# Supplementary material for: Tailored cobalt-salen complexes enable electrocatalytic intramolecular allylic C–H functionalizations
Source: Nat Commun. 2021 Jun 18;12:3745. doi: 10.1038/s41467-021-24125-5 (PMC8213807; doi:10.1038/s41467-021-24125-5)
Supplement: Supplementary file 1 — Supplementary Information [file 41467_2021_24125_MOESM1_ESM.pdf]

Tailored cobalt salen complexes enable electrocatalytic intramolecular allylic C–H functionalizations

Chen-Yan Cai, Zheng-Jian Wu, Ji-Ying Liu, Ming Chen, Jinshuai Song & Hai-Chao Xu

Supplementary Table 1 Additioal optimization of conditions for allylic C–H amination[a]

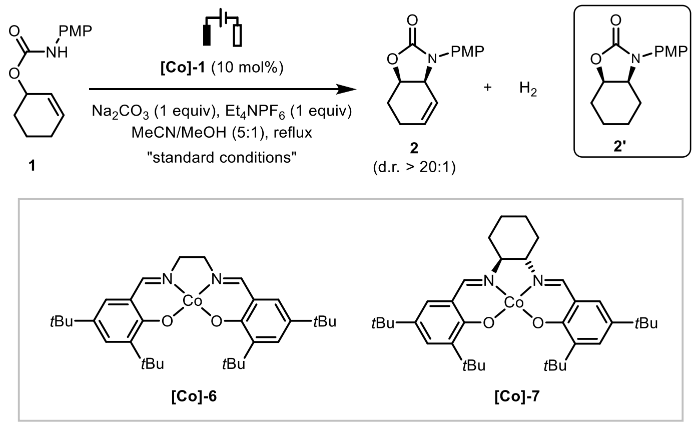

Entry	Deviation from standard conditions	Yield of **2** (%)[b]
1	No Et_4NPF_6	64 (12)
2	Et_4NBF_4 instead of Et_4NPF_6	87
3	$LiClO_4$ instead of Et_4NPF_6	70 (25)
4	NaOPiv instead of Na_2CO_3	39 (54)
5	K_2CO_3 instead of Na_2CO_3	85
6	$NaHCO_3$ instead of Na_2CO_3	82
7	K_2HPO_4•$3H_2O$ instead of Na_2CO_3	83
8	LiOMe instead of Na_2CO_3	39 (4)
9	Cs_2CO_3 instead of Na_2CO_3	0[d]
10	Stainless steel plate (1 cm x 1 cm) as cathode	75 (21)
11	Graphite plate (1 cm x 1 cm) as anode	0 (70)
12	MeCN as solvent	56
13	MeOH as solvent	2 (95)
14	**[Co]-5** as catalyst	25[c]
15	**[Co]-6** as catalyst	25[e]
16	**[Co]-7** as catalyst	24[e]
17	No electricity	0 (>95)

[a]Reaction conditions: RVC (1 cm x 1 cm x 1.2 cm), Pt plate cathode (1 cm x 1 cm), **1** (0.2 mmol), MeCN (5 mL), MeOH (1 mL), Et_4NPF_6 (0.2 mmol), Na_2CO_3 (0.2 mmol), 10 mA, 4 h (7.5 F mol^{-1}). [b]Determined by ^1H NMR analysis using 1,3,5-trimethoxybenzene as the internal standard. Unreacted **1** was shown in brackets. [c]**2'** was formed in 4% yield. [d]Compound **1** underwent methanolysis to give MeOCONHPMP in 60% yield. [e]**2'** was formed in 9% yield.

Supplementary Table 2 Optimization of conditions for allylic C–H alkylation[a]

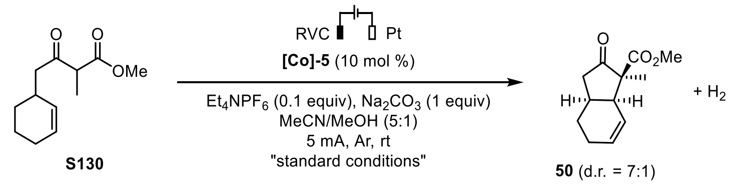

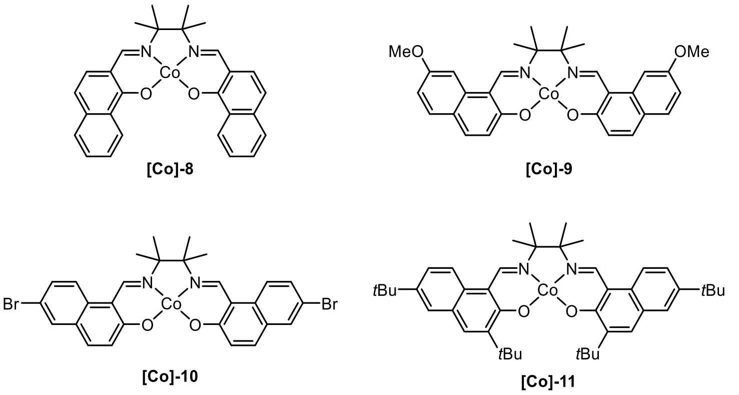

Entry	Deviation from standard conditions	Yield of **50** (%)[b]
1	None	71[c]
2	No **[Co]-5**	5 (9)
3	no electricity	0 (80)
4	No Na_2CO_3	46 (22)
5	$MeCN/H_2O$ (5:1) instead of MeCN/MeOH (5:1)	30 (54)
6	MeCN as solvent	16 (31)
7	MeOH as solvent	39 (6)
8	2 mA	32 (16)
9	10 mA	46 (19)
10	**[Co]-1** as catalyst,	62
11	**[Co]-6** as catalyst	16 (24)
12	**[Co]-7** as catalyst	15 (19)
13	**[Co]-8** as catalyst	68
14	**[Co]-9** as catalyst	62
15	**[Co]-10** as catalyst	17 (17)
16	**[Co]-11** as catalyst	23 (8)

[a]Reaction conditions: RVC (1 cm x 1 cm x 1.2 cm), Pt plate cathode (1 cm x 1 cm), **S130** (0.3 mmol), MeCN (5 mL), MeOH (1 mL), Et_4NPF_6 (0.03 mmol), Na_2CO_3 (0.3 mmol), 5 mA, 4 h (2.5 F mol^{-1}). [b]Determined by ^1H NMR analysis using 1,3,5-trimethoxybenzene as the internal standard. Unreacted **S130** was shown in brackets. [c]Isolated yield.

Supplementary Table 3 Additional experiments with stoichiometric [Co^{III}][a]

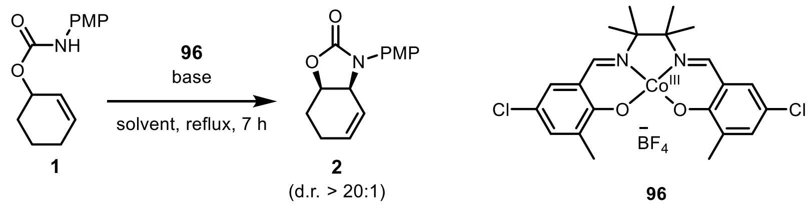

Entry	**96**	Base	Solvent	**2**	Unreacted **1**
1	1 equiv	Na_2CO_3 (1 equiv)	MeCN/MeOH (5:1)	0%	95%
2	2 equiv	Cs_2CO_3 (1 equiv)	MeCN/MeOH (5:1)	0%	90%
3	1 equiv	Cs_2CO_3 (1 equiv)	MeCN	8%	80%

[a]Reactions were conducted with 0.05 mmol of **1**.

Supplementary Table 4 Reaction in deuterated solvent

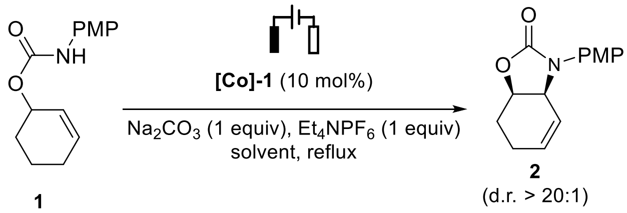

Entry	Solvent	**2**
1	$MeCN/CD_3OD$ (5:1)	86%
2	$CD_3CN/MeOH$ (5:1)	85%
3	CD_3CN/CD_3OD	86%

M06-2X/def2-TZVPP//M06-2X/6-31G(d)

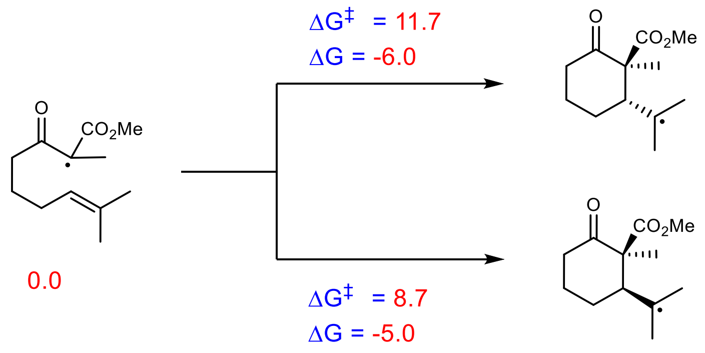

Supplementary Figure 1. DFT calculated energetics (kcal mol^{-1}). Geometries optimization calculations were carried out by a meta-GGA hybrid functional M06-2X[1] with 6-31G* basis set[2,3] for all atoms. Vibrational frequencies were calculated analytically at the same level to check the stationary point or transition state as minima or saddle point, and to obtain the thermodynamic corrections. A large basis set of def2-TZVPP[4] was employed for single energy correction. The M06-2X functional has good performance in thermochemistry calculations[5], and the basis set def2-TZVPP presents convergent results[6] in previous benchmark studies. The changes in Gibbs free energy are reported in the content (relative energies labeled in red). All the theoretical calculations were performed in Gaussian16 package[7].

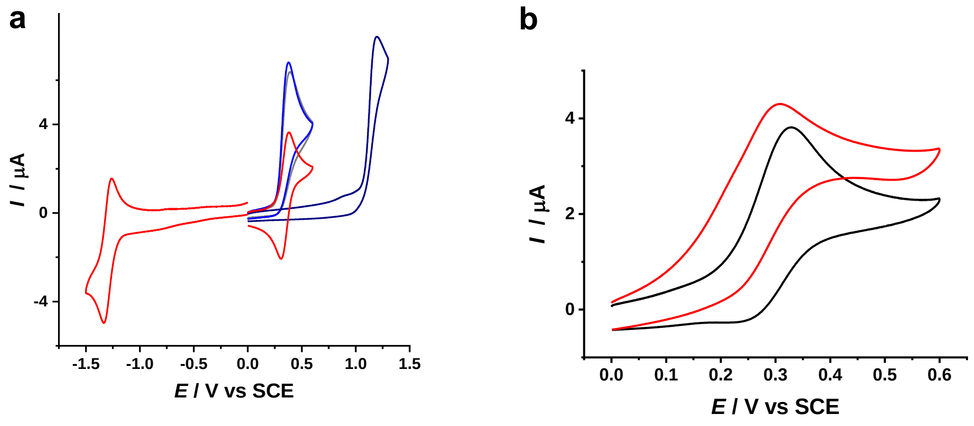

Supplementary Figure 2. Cyclic voltammograms. The cyclic voltammograms were recorded under argon in an electrolyte of Et_4NPF_6 (0.1 M) in MeCN/MeOH (5:1) using a glassy carbon disk working electrode (diameter, 1 mm), a Pt wire auxiliary electrode and a SCE reference electrode. The scan rate was 100 mV/s. **a**, Red: **[Co]-1** (1.5 mM). Navy: **1** (1 mM), Na_2CO_3 (1 mM). Gray: **1** (3 mM), Cs_2CO_3 (3 mM). Blue: **1** (3 mM), NaOMe (3 mM). **b**, Black: **[Co]-1** (1.5 mM), **1** (10 mM), NaOMe (1.5 mM). Red: **[Co]-1** (1.5 mM), **1** (10 mM), NaOMe (1.5 mM), tested at 60 °C.

Supplementary Methods

General considerations

Anhydrous acetonitrile and methanol were obtained from distillation under argon from calcium hydride and magnesium chips, respectively. Anhydrous tetrahydrofuran was purchased from Energy and used without further purification. Et_4NPF_6 was purified via recrystallization from ethanol/H_2O. Na_2CO_3 was purchased from Aldrich. The other commercially available reagents were used without purification. Flash column chromatography was performed with silica gel (200–300 mesh). Cyclic voltammograms were recorded on a CHI 760E potentiostat. NMR spectra were recorded on Bruker AV-500 and Bruker AV-600 instruments. Data were reported as chemical shifts in ppm relative to TMS (0.00 ppm) for ^1H and $CDCl_3$ (77.2 ppm) for ^{13}C. The abbreviations used for explaining the multiplicities were as follows: s = singlet, d = doublet, t = triplet, q = quartet, m = multiplet, br = broad. Infrared spectra were recorded on a Nicolet AVATER FTIR330 spectrometer. High resolution mass spectra (ESI) were recorded by the instrumentation center of Department of Chemistry, Xiamen University, on a Micromass QTOF2 Quadruple/Time-of-Flight Tandem mass spectrometer. The reticulated vitreous carbon (100 pores per inch) can be obtained from Goodfellow. The Co-complexes were prepared according to reported procedures[8].

Procedures for the Electrolysis

General procedure for allylic C–H amination. The substrate (0.2 mmol, 1 equiv), Et_4NPF_6 (0.2 mmol, 1 equiv), Na_2CO_3 (0.2 mmol, 1 equiv) and **[Co]-1** (0.02 mmol, 0.1 equiv) were placed in a 10 mL three-necked round-bottom flask. The flask was equipped with a condenser, a reticulated vitreous carbon (RVC) anode (100 PPI, 1 cm x 1 cm x 1.2 cm), and a platinum plate (1 cm x 1 cm x 0.1 cm) cathode (Supplementary Fig. 3a). The flask was flushed with argon. MeCN (5 mL) and MeOH (1 mL) were added. The electrolysis was carried out at reflux using a constant current of 10 mA until complete consumption of the substrate (monitored by TLC or ^1H NMR). The reaction mixture was cooled to rt. H_2O (30 mL) and EtOAc (30 ml) were added. The phases were separated, and the aqueous phase was extracted twice with EtOAc. The combined organic solution was dried over anhydrous $MgSO_4$, filtered, and concentrated under reduced pressure. The residue was chromatographed through silica gel eluting with EtOAc/hexanes to give the product.

Gram scale synthesis of 2. The electrolysis was conducted in a 1-L beaker-type cell with a RVC (100 PPI, 5 cm x 8 cm x 1.2 cm) anode, a Pt plate cathode (5 cm x 5 cm x 0.1 cm), and a constant current of 400 mA (Supplementary Fig. 3b). The reaction mixture consisted **[Co]-1** (2.87 g, 6.00 mmol), **1** (14.5 g, 58.7 mmol), Na_2CO_3 (6.36 g, 60.0 mmol), Et_4NPF_6 (16.5 g, 60.0 mmol), MeOH (140 mL) and MeCN (700 mL). The reaction time was 42 h. The reaction mixture was cooled to rt and concentrated under reduced pressure. The residue was diluted

with EtOAc and H_2O. The phases were separated, and the aqueous phase was extracted twice with EtOAc. The combined organic solution was dried over anhydrous $MgSO_4$, filtered, and concentrated under reduced pressure. The residue was chromatographed through silica gel eluting with EtOAc/hexanes to give the product as light yellow solid (71% yield, 10.3 g).

a b c

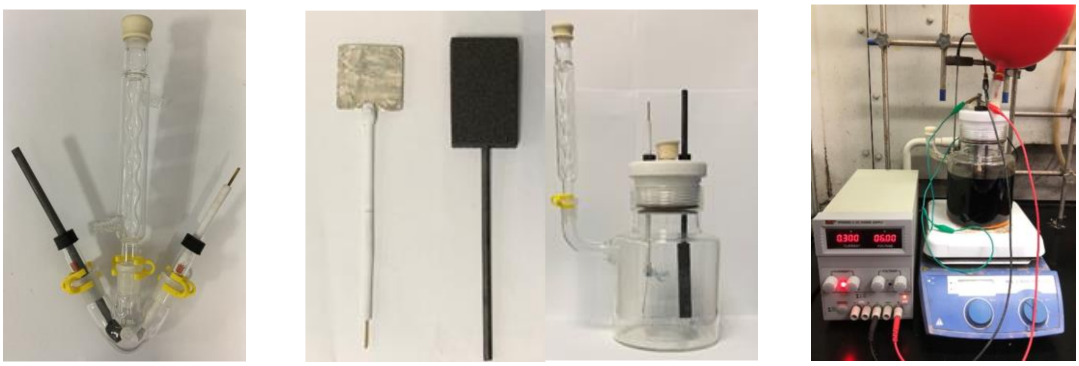

Supplementary Figure 3. Electrolysis setups. a, Setup for milligram scale reactions. **b**, Setup for the gram scale synthesis of **2**. **c**, Setup for the gram scale synthesis of **63**.

General procedure for allylic C–H alkylation. A 10 mL three-necked round bottom flask was charged with **[Co]-5** (0.03 mmol, 10 mol%), Et_4NPF_6 (0.03 mmol, 0.1 equiv) and Na_2CO_3 (0.3 mmol, 1 equiv). The flask was equipped with a reticulated vitreous carbon anode (100 PPI, 1 cm x 1 cm x 1.2 cm) and a platinum plate (1 cm x 1 cm) cathode. The flask was flushed with argon. The substrate (0.3 mmol, 1 equiv), MeCN (5 mL) and MeOH (1 mL) were added. The electrolysis was carried out at rt or reflux using a constant current of 5 mA until complete consumption of the substrate (monitored by TLC or ^{1}H NMR). The reaction mixture was concentrated under reduced pressure. The residue was chromatographed through silica gel eluting with EtOAc/hexanes to give the desired product.

Gram scale synthesis of 63. The electrolysis was conducted in a 1-L beaker-type cell equipped with two RVC plates as anode (100 PPI, 1.2 cm x 5 cm x 6 cm) and a platinum plate (5 cm x 5 cm x 0.1 cm) cathode (Supplementary Fig. 3c). The three electrodes were placed in parallel with the Pt plate cathode sandwiched between two RVC anodes. The cell was charged with Co(II) salen complex **[Co]-5** (2.53 g, 5.26 mmol, 10 mol%), Et_4NPF_6 (1.45 g, 5.26 mmol, 0.1 equiv) and Na_2CO_3 (5.58 g, 52.6 mmol, 1 equiv). MeCN (700 mL) and MeOH (140 mL) were added. The mixture was stirred at rt and bubbled with argon for 1 h. Compound **83** (14.0 g, 52.6 mmol, 1.0 equiv) was added in one portion. The constant current (300 mA) electrolysis was carried out at rt for 10.3 h (2.2 F mol^{-1}). The reaction mixture was concentrated under reduced pressure. The residue was purified by flash column chromatography on silica gel eluting with hexane/EtOAc (30:1) to afford **64** as a pale-yellow

oil (12.2 g, 88% yield).

Characterization data for the electrolysis products

Compounds **4**[9], **16**[9], **21**[10], **35**[9], **51**[11], **54**[11], **59**[11] have been reported. All the diastereomeric ratio values were determined by crude ^{1}H NMR spectroscopy.

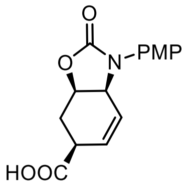

3-(4-Methoxyphenyl)-2-oxo-2,3,3a,6,7,7a-hexahydrobenzo[*d*]oxazole-6-carboxylic acid (3). Light yellow solid; Yield = 64%; Electricity = 8.8 F mol^{-1}; ^{1}H NMR (600 MHz, DMSO-d_6) δ 12.55 (brs, 1H), 7.40–7.34 (m, 2H), 7.01–6.95 (m, 2H), 6.11 (ddd, J = 10.2, 3.6, 1.2 Hz, 1H), 5.70 (ddd, J = 10.2, 3.3, 2.3 Hz, 1H), 4.96–4.90 (m, 1H), 4.88–4.84 (m, 1H), 3.76 (s, 3H), 3.25–3.20 (m, 1H), 2.21 (ddd, J = 13.5, 5.7, 4.2 Hz, 1H), 2.06 (dt, J = 13.5, 7.6 Hz, 1H); ^{13}C NMR (151 MHz, DMSO-d_6) δ 174.5, 157.6, 155.6, 131.6, 130.6, 124.9, 123.1, 115.2, 72.1, 56.2, 53.8, 38.3, 28.2; IR (neat, cm^{-1}): 3418, 1652, 1025, 998, 765; ESI HRMS *m/z* (M+Na)$^+$ calcd 312.0842, obsd 312.0845.

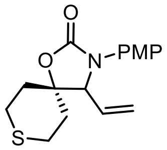

3-(4-Methoxyphenyl)-4-vinyl-1-oxa-8-thia-3-azaspiro[4.5]decan-2-one (5). White solid; Yield = 55%; Electricity = 6.5 F mol^{-1}; ^{1}H NMR (500 MHz, $CDCl_3$) δ 7.27–7.23 (m, 2H), 6.90–6.84 (m, 2H), 5.70 (ddd, J = 16.9, 10.2, 8.5 Hz, 1H), 5.44–5.27 (m, 2H), 4.25 (d, J = 8.5 Hz, 1H), 3.78 (s, 3H), 3.17, 3.09 (AB of ABX_2, 2H, J_{AB} = 13.2 Hz, J_{AX} = 2.7 Hz, J_{BX} = 2.7 Hz), 2.57–2.45 (m, 2H), 2.32–2.25 (m, 1H), 2.24–2.17 (m, 1H), 1.99, 1.82 (AB of ABX_2, 2H, J_{AB} = 13.6 Hz, J_{AX} = 3.7 Hz, J_{BX} = 3.7 Hz); ^{13}C NMR (151 MHz, $CDCl_3$) δ 157.4, 154.9, 132.2, 129.9, 124.4, 122.2, 114.4, 80.1, 70.8, 55.6, 37.9, 33.9, 24.4, 24.2; IR (neat, cm^{-1}): 2919, 1750, 1514, 1247, 1142, 831, 605; ESI HRMS *m/z* (M+Na)$^+$ calcd 328.0978, obsd 328.0983.

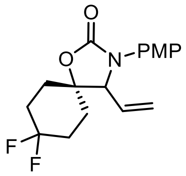

8,8-Difluoro-3-(4-methoxyphenyl)-4-vinyl-1-oxa-3-azaspiro[4.5]decan-2-one (6). Light yellow solid; Yield = 92%; Electricity = 10.3 F mol^{-1}; ^{1}H NMR (600 MHz, $CDCl_3$) δ 7.32–7.26 (m, 2H), 6.93–6.87 (m, 2H), 5.73 (ddd, J = 17.0, 10.2, 8.6 Hz, 1H), 5.43–5.29 (m, 2H), 4.37 (d, J = 8.6 Hz, 1H), 3.80 (s, 3H), 2.37–2.18 (m, 2H), 2.18–2.07 (m, 3H), 2.04 (ddt, J =

14.2, 4.9, 2.9 Hz, 1H), 1.99–1.90 (m, 1H), 1.81–1.72 (m, 1H); ^{13}C NMR (151 MHz, $CDCl_3$) δ 157.5, 154.9, 132.2, 129.8, 124.4, 122.4 (dd, $J_{C\text{-}F}$ = 244.1, 238.4 Hz), 122.3, 114.4, 79.6 (d, *J* = 1.4 Hz), 69.2 (d, $J_{C\text{-}F}$ = 2.4 Hz), 55.6, 33.3 (d, $J_{C\text{-}F}$ = 9.8 Hz), 29.9 (t, $J_{C\text{-}F}$ = 47.5 Hz), 29.6 (t, $J_{C\text{-}F}$ = 47.5 Hz), 28.8 (d, $J_{C\text{-}F}$ = 9.8 Hz); ^{19}F NMR (565 MHz, $CDCl_3$) δ −93.2 (d, *J* = 238.4 Hz), -104.1 (d, *J* = 238.1 Hz); IR (neat, cm^{-1}): 2948, 1752, 1514, 1249, 988, 832; ESI HRMS *m/z* $(M+Na)^+$ calcd 346.1225, obsd 346.1227.

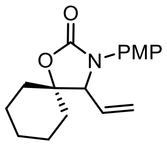

3-(4-Methoxyphenyl)-4-vinyl-1-oxa-3-azaspiro[4.5]decan-2-one (7). Light yellow oil; Yield = 82%; Electricity = 11.5 F mol^{-1}; ^{1}H NMR (600 MHz, $CDCl_3$) δ 7.32–7.28 (m, 2H), 6.90–6.83 (m, 2H), 5.73 (ddd, *J* = 17.0, 10.2, 8.6 Hz, 1H), 5.36–5.22 (m, 2H), 4.26 (d, *J* = 8.6 Hz, 1H), 3.78 (s, 3H), 2.00–1.93 (m, 1H), 1.91–1.85 (m, 1H), 1.85–1.77 (m, 1H), 1.76–1.68 (m, 1H), 1.68–1.56 (m, 4H), 1.48 (ddd, *J* = 13.4, 11.7, 4.2 Hz, 1H), 1.34–1.24 (m, 1H); ^{13}C NMR (151 MHz, $CDCl_3$) δ 157.1, 155.5, 133.0, 130.5, 124.1, 121.3, 114.3, 81.8, 70.0, 55.6, 36.8, 32.2, 25.1, 22.2, 21.9; IR (neat, cm^{-1}): 2936, 2862, 1747, 1514, 1248, 830; ESI HRMS *m/z* $(M+Na)^+$ calcd 310.1414, obsd 310.1400.

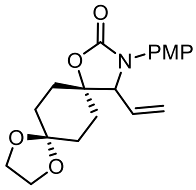

3-(4-Methoxyphenyl)-4-vinyl-1,9,12-trioxa-3-azadispiro$[4.2.4^{8}.2^{5}]$tetradecan-2-one (8). Colorless oil; Yield = 58%; Electricity = 6.5 F mol^{-1}; ^{1}H NMR (500 MHz, $CDCl_3$) δ 7.32–7.27 (m, 2H), 6.90–6.85 (m, 2H), 5.72 (ddd, *J* = 16.9, 10.2, 8.6 Hz, 1H), 5.38–5.27 (m, 2H), 4.31 (d, *J* = 8.6 Hz, 1H), 4.01–3.96 (m, 2H), 3.96–3.91 (m, 2H), 3.78 (s, 3H), 2.13–2.02 (m, 2H), 2.01–1.89 (m, 3H), 1.80–1.75 (m, 1H), 1.75–1.67 (m, 2H); ^{13}C NMR (151 MHz, $CDCl_3$) δ 157.3, 155.3, 132.7, 130.2, 124.3, 121.7, 114.3, 107.8, 80.6, 69.4, 64.7, 64.5, 55.6, 34.6, 30.8, 30.5, 29.8; IR (neat, cm^{-1}): 2957, 1750, 1514, 1248, 1090, 832, 756; ESI HRMS *m/z* $(M+Na)^+$ calcd 368.1468, obsd 368.1470.

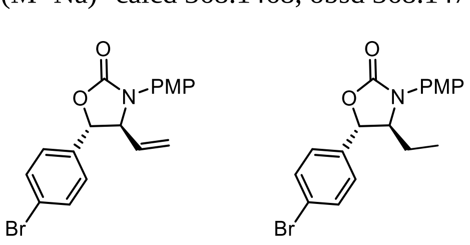

9 17:1 **9'**

5-(4-Bromophenyl)-3-(4-methoxyphenyl)-4-vinyloxazolidin-2-one (9). Light yellow solid; Isolated as a 17:1 mixture of **9** and **9'**; Yield = 67%; Electricity = 5.2 F mol^{-1}; ^{1}H NMR (500

MHz, $CDCl_3$) δ 7.60–7.52 (m, 2H), 7.33–7.27 (m, 2H), 7.28–7.20 (m, 2H), 6.91–6.85 (m, 2H), 5.84 (ddd, J = 17.0, 10.2, 8.3 Hz, 1H), 5.35 (d, J = 10.2 Hz, 1H), 5.24 (d, J = 17.2 Hz, 1H), 5.18 (d, J = 7.4 Hz, 1H), 4.48 (dd, J = 8.3, 7.4 Hz, 1H), 3.78 (s, 3H); ^{13}C NMR (126 MHz, $CDCl_3$) δ 157.6, 155.5, 136.4, 134.2, 132.3, 129.5, 127.5, 124.6, 123.3, 122.2, 114.4, 79.6, 68.8, 55.6; IR (neat, cm^{-1}): 3439, 1755, 1637, 1533, 1247, 1008, 826; ESI HRMS *m/z* $(M+Na)^+$ calcd 396.0206, obsd 396.0215.

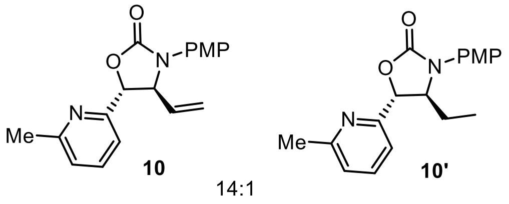

3-(4-Methoxyphenyl)-5-(6-methylpyridin-2-yl)-4-vinyloxazolidin-2-one (10). Brown oil; Isolated as a 14:1 mixture of **10** and **10'**; Yield = 72%; Electricity = 8.1 F mol^{-1}; ^{1}H NMR (500 MHz, $CDCl_3$) δ 7.65–7.60 (m, 1H), 7.38–7.31 (m, 3H), 7.14 (d, J = 7.8 Hz, 1H), 6.87 (d, J = 9.0 Hz, 2H), 6.01 (ddd, J = 17.3, 10.3, 7.4 Hz, 1H), 5.38–5.32 (m, 2H), 5.26 (d, J = 5.0 Hz, 1H), 4.99 (dd, J = 7.4, 5.1 Hz, 1H), 3.78 (s, 3H), 2.56 (s, 3H); ^{13}C NMR (151 MHz, $CDCl_3$) δ 158.9, 157.2, 156.2, 155.4, 137.4, 135.0, 130.1, 123.8, 123.5, 120.5, 117.9, 114.3, 80.0, 65.6, 55.6, 24.6; IR (neat, cm^{-1}): 2934, 1754, 1514, 1248, 831, 752; ESI HRMS *m/z* $(M+Na)^+$ calcd 333.1210, obsd 333.1217.

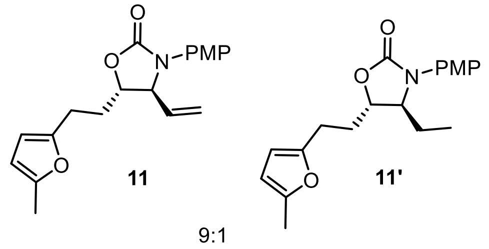

3-(4-Methoxyphenyl)-5-(2-(5-methylfuran-2-yl)ethyl)-4-vinyloxazolidin-2-one (11). Light yellow oil; Isolated as a 9:1 mixture of **11** and **11'**; Yield = 43%; Electricity = 6.8 F mol^{-1}; ^{1}H NMR (600 MHz, $CDCl_3$) δ 7.27–7.23 (m, 2H), 6.90–6.85 (m, 2H), 5.93–5.90 (m, 1H), 5.87–5.84 (m, 1H), 5.72 (ddd, J = 17.1, 10.1, 8.1 Hz, 1H), 5.34–5.26 (m, 2H), 4.31 (dd, J = 8.1, 6.9 Hz, 1H), 4.26 (td, J = 7.1, 4.9 Hz, 1H), 3.78 (s, 3H), 2.88 (dt, J = 14.8, 6.8 Hz, 1H), 2.77 (dt, J = 15.5, 7.9 Hz, 1H), 2.25 (s, 3H), 2.14–2.08 (m, 2H); ^{13}C NMR (151 MHz, $CDCl_3$) δ 157.4, 155.8, 152.2, 151.0, 135.0, 129.9, 124.3, 121.1, 114.4, 106.5, 106.1, 78.3, 66.2, 55.6, 32.4, 23.8, 13.7; IR (neat, cm^{-1}): 3445, 2921, 1750, 1514, 1249, 838, 560; ESI HRMS *m/z* $(M+Na)^+$ calcd 350.1363, obsd 350.1368.

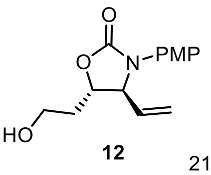

12

21:1

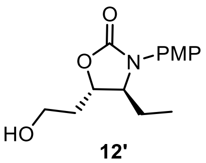

12'

5-(2-Hydroxyethyl)-3-(4-methoxyphenyl)-4-vinyloxazolidin-2-one (12). Light yellow oil; Isolated as a 21:1 mixture of **12** and **12'**; Yield = 55%; Electricity = 9.6 F mol^{-1}; ^{1}H NMR (500 MHz, $CDCl_3$) δ 7.29–7.24 (m, 2H), 6.91–6.86 (m, 2H), 5.74 (ddd, *J* = 17.4, 10.2, 7.5 Hz, 1H), 5.37–5.26 (m, 2H), 4.50–4.38 (m, 2H), 3.90–3.84 (m, 2H), 3.78 (s, 3H), 2.08–1.98 (m, 2H), 1.97–1.79 (m, 1H); ^{13}C NMR (126 MHz, $CDCl_3$) δ 157.5, 156.0, 134.7, 129.8, 124.5, 121.2, 114.4, 76.9, 66.4, 58.5, 55.6, 36.2; IR (neat, cm^{-1}): 3425, 2937, 2838, 1743, 1514, 1399, 1249, 832, 759; ESI HRMS *m/z* (M+Na)$^+$ calcd 286.1050, obsd 286.1056.

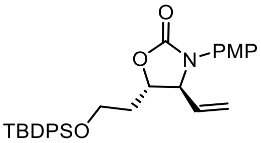

5-(2-((*tert*-Butyldiphenylsilyl)oxy)ethyl)-3-(4-methoxyphenyl)-4-vinyloxazolidin-2-one (13). Light yellow oil; Yield = 65%; Electricity = 8.5 F mol^{-1}; ^{1}H NMR (500 MHz, $CDCl_3$) δ 7.68–7.62 (m, 4H), 7.45–7.36 (m, 6H), 7.28–7.23 (m, 2H), 6.89–6.84 (m, 2H), 5.75 (ddd, *J* = 17.7, 10.1, 8.1 Hz, 1H), 5.33–5.24 (m, 2H), 4.54–4.48 (m, 1H), 4.45–4.38 (m, 1H), 3.95–3.83 (m, 2H), 3.77 (s, 3H), 2.03–1.97 (m, 2H), 1.06 (s, 9H); ^{13}C NMR (126 MHz, $CDCl_3$) δ 157.3, 155.9, 135.7, 135.0, 133.6, 133.4, 130.1, 130.0, 127.9 (2C), 124.2, 120.9, 114.3, 76.3, 66.1, 59.6, 55.6, 36.5, 27.0, 19.3; IR (neat, cm^{-1}): 2956, 2929, 1754, 1514, 1250, 1111, 703, 505; ESI HRMS *m/z* (M+Na)$^+$ calcd 524.2228, obsd 524.2233.

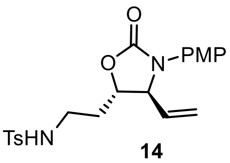

14

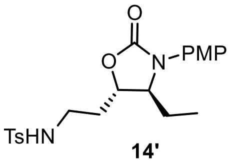

14'

8:1

***N*-(2-(3-(4-Methoxyphenyl)-2-oxo-4-vinyloxazolidin-5-yl)ethyl)-4-methylbenzenesulfonamide (14).** Light yellow oil; Isolated as a 8:1 mixture of **14** and **14'**; Yield = 49%; Electricity = 11.8 F mol^{-1}; ^{1}H NMR (500 MHz, $CDCl_3$) δ 7.79–7.71 (m, 2H), 7.33–7.28 (m, 2H), 7.24–7.20 (m, 2H), 6.88–6.83 (m, 2H), 5.67 (ddd, *J* = 17.1, 10.1, 7.9 Hz, 1H), 5.34–5.23 (m, 2H), 5.17 (t, *J* = 6.3 Hz, 1H), 4.35–4.23 (m, 2H), 3.78 (s, 3H), 3.19–3.06 (m, 2H), 2.42 (s, 3H), 2.10–1.99 (m, 1H), 1.98–1.89 (m, 1H); ^{13}C NMR (126 MHz, $CDCl_3$) δ 157.6, 155.6, 143.7, 136.8, 134.3, 130.0, 129.6, 127.3, 124.5, 121.6, 114.4, 76.9, 66.3, 55.6, 39.6, 33.7, 21.7; IR (neat, cm^{-1}): 3259, 2924, 1735, 1514, 1182, 1159, 1093, 832, 552; ESI HRMS *m/z* (M+Na)$^+$ calcd 439.1298, obsd 439.1308.

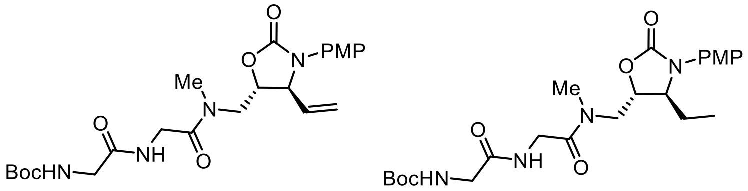

15 **15'**

8:1

***tert*-Butyl (2-((2-(((3-(4-methoxyphenyl)-2-oxo-4-vinyloxazolidin-5-yl)methyl)(methyl) amino)-2-oxoethyl)amino)-2-oxoethyl)carbamate (15).** Light yellow oil; Isolated as a 8:1 mixture of **15** and **15'**; Yield = 72%; Electricity = 5.6 F mol^{-1}; ^{1}H NMR (500 MHz, $CDCl_3$) δ 7.27–7.22 (m, 2H), 7.04 (brs, 1H), 6.90–6.86 (m, 2H), 5.73 (ddd, J = 17.6, 10.2, 7.8 Hz, 1H), 5.40–5.30 (m, 3H), 4.51–4.45 (m, 1H), 4.45–4.39 (m, 1H), 4.12–4.08 (m, 2H), 3.97 (dd, J = 14.5, 3.5 Hz, 1H), 3.90–3.83 (m, 2H), 3.78 (s, 3H), 3.62 (dd, J = 14.4, 6.4 Hz, 1H), 3.14 (s, 3H), 1.46 (s, 9H); ^{13}C NMR (126 MHz, $CDCl_3$) δ 169.6, 169.2, 157.5, 156.1, 155.2, 134.1, 129.4, 124.3, 121.6, 114.4, 80.3, 77.7, 63.4, 55.6, 50.0, 44.2, 41.5, 36.4, 28.4; IR (neat, cm^{-1}): 3346, 2929, 1751, 1651, 1515, 1249, 1169, 833; ESI HRMS m/z $(M+Na)^+$ calcd 499.2163, obsd 499.2172.

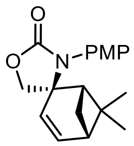

(1*S*,2*R*,5*S*)-3'-(4-Methoxyphenyl)-6,6-dimethylspiro[bicyclo[3.1.1]heptane-2,4'-oxazolidin]-3-en-2'-one (17). The stereochemistry was determined using NOE experiment. Light yellow solid; Yield = 90%; Electricity = 9.3 F mol^{-1}; ^{1}H NMR (600 MHz, $CDCl_3$) δ 7.10–7.06 (m, 2H), 6.90–6.85 (m, 2H), 6.42 (dd, J = 8.6, 6.6 Hz, 1H), 5.77 (dd, J = 8.8, 2.2 Hz, 1H), 4.42, 4.26 (AB, 2H, J_{AB} = 9.0 Hz), 3.79 (s, 3H), 2.45 (td, J = 5.8, 2.3 Hz, 1H), 2.07–1.98 (m, 2H), 1.33 (s, 3H), 0.94 (s, 3H), 0.76 (d, J = 9.4 Hz, 1H); ^{13}C NMR (151 MHz, $CDCl_3$) δ 159.5, 157.3, 141.4, 130.9, 129.0, 125.3, 114.6, 73.0, 68.2, 55.5, 51.8, 47.3, 40.8, 30.7, 26.8, 23.8; IR (neat, cm^{-1}): 2987, 2942, 1754, 1514, 1250, 1109, 831, 760; ESI HRMS m/z $(M+Na)^+$ calcd 322.1414, obsd 322.1421.

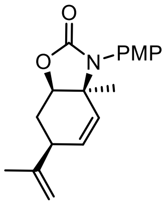

(3a*S*,6*S*,7a*R*)-3-(4-Methoxyphenyl)-3a-methyl-6-(prop-1-en-2-yl)-3a,6,7,7a-tetrahydro benzo[*d*]oxazol-2(3*H*)-one (18). Colorless oil; Yield = 88%; Electricity = 6.5 F mol^{-1}; ^{1}H NMR (600 MHz, $CDCl_3$) δ 7.18–7.13 (m, 2H), 6.96–6.92 (m, 2H), 5.89 (ddd, J = 10.1, 2.7, 0.9 Hz, 1H), 5.45 (dd, J = 10.1, 2.5 Hz, 1H), 4.91–4.86 (m, 1H), 4.84–4.81 (m, 1H), 4.49 (dd,

J = 9.7, 4.7 Hz, 1H), 3.83 (s, 3H), 2.89–2.78 (m, 1H), 2.18 (dtd, *J* = 13.2, 4.8, 1.0 Hz, 1H), 1.97 (dt, *J* = 13.1, 9.7 Hz, 1H), 1.84–1.78 (m, 3H), 1.37 (s, 3H); ^{13}C NMR (151 MHz, $CDCl_3$) δ 159.4, 156.3, 146.5, 133.0, 130.6, 127.7, 127.3, 114.6, 111.8, 79.5, 60.0, 55.6, 40.3, 30.8, 25.6, 20.7; IR (neat, cm^{-1}): 3418, 2967, 1751, 1514, 1249, 832, 529; ESI HRMS *m/z* $(M+Na)^+$ calcd 322.1414, obsd 322.1424.

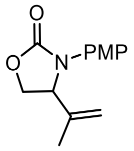

3-(4-Methoxyphenyl)-4-(prop-1-en-2-yl)oxazolidin-2-one (19). Light yellow oil; Yield = 78%; Electricity = 5.0 F mol^{-1}; ^{1}H NMR (500 MHz, $CDCl_3$) δ 7.44–7.28 (m, 2H), 6.96–6.77 (m, 2H), 5.05 (s, 1H), 5.00 (s, 1H), 4.82 (dd, *J* = 9.1, 5.7 Hz, 1H), 4.54 (t, *J* = 9.0 Hz, 1H), 4.19–4.02 (m, 1H), 3.78 (s, 3H), 1.68 (s, 3H); ^{13}C NMR (151 MHz, $CDCl_3$) δ 156.2, 141.4, 130.2, 122.7, 116.7, 114.3, 66.1, 62.7, 55.5, 16.4; IR (neat, cm^{-1}): 3418, 2935, 1754, 1556, 1285, 819, 705; ESI HRMS *m/z* $(M+Na)^+$ calcd 256.0944, obsd 256.0949.

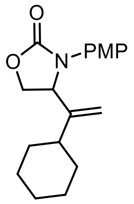

4-(1-Cyclohexylvinyl)-3-(4-methoxyphenyl)oxazolidin-2-one (20). Colorless oil; Yield = 63%; Electricity = 3.7 F mol^{-1}; ^{1}H NMR (500 MHz, $CDCl_3$) δ 7.37–7.30 (m, 2H), 6.89–6.84 (m, 2H), 5.04 (s, 1H), 5.02 (d, *J* = 0.9 Hz, 1H), 4.80 (dd, *J* = 8.9, 5.7 Hz, 1H), 4.56 (t, *J* = 8.7 Hz, 1H), 4.05 (dd, *J* = 8.4, 5.7 Hz, 1H), 3.78 (s, 3H), 1.86 (tt, *J* = 11.6, 3.0 Hz, 1H), 1.82–1.75 (m, 2H), 1.75–1.67 (m, 2H), 1.48–1.42 (m, 1H), 1.34–1.09 (m, 5H); ^{13}C NMR (126 MHz, $CDCl_3$) δ 156.7, 156.4, 150.8, 130.6, 122.5, 114.2, 112.6, 68.0, 61.1, 55.6, 40.9, 33.8, 32.9, 26.8, 26.7, 26.1; IR (neat, cm^{-1}): 2925, 1751, 1514, 1248, 828, 756; ESI HRMS *m/z* $(M+Na)^+$ calcd 324.1570, obsd 324.1578.

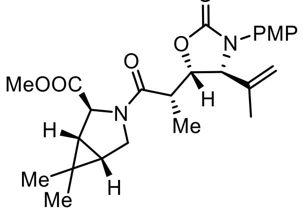

Methyl 3-(2-(3-(4-methoxyphenyl)-2-oxo-4-(prop-1-en-2-yl)oxazolidin-5-yl)propanoyl)-6,6-dimethyl-3-azabicyclo[3.1.0]hexane-2-carboxylate (22). Light yellow oil; Yield = 91%; Isolated as a 15:1 mixture of diastereomers and only the major isomer was shown. Electricity = 9.9 F mol^{-1}; ^{1}H NMR (500 MHz, $CDCl_3$) δ 7.51–7.45 (m, 2H), 6.91–6.87 (m, 2H), 5.00 (s, 1H), 4.94–4.90 (m, 1H), 4.68 (d, *J* = 2.5 Hz, 1H), 4.42 (s, 1H), 4.38 (dd, *J* = 8.9, 2.6 Hz, 1H),

3.91 (dd, J = 10.0, 5.0 Hz, 1H), 3.78 (s, 3H), 3.70 (s, 3H), 3.51 (d, J = 10.1 Hz, 1H), 2.89 (dq, J = 8.8, 6.8 Hz, 1H), 1.72–1.66 (m, 1H), 1.51–1.43 (m, 2H), 1.30 (d, J = 6.8 Hz, 3H), 1.06 (s, 3H), 0.97 (s, 3H); ^{13}C NMR (126 MHz, $CDCl_3$) δ 172.2, 171.4, 156.7, 155.0, 141.3, 130.5, 122.3, 116.0, 114.3, 79.8, 65.7, 59.4, 55.5, 52.6, 47.5, 42.8, 30.6, 27.3, 26.3, 19.7, 17.2, 13.8, 12.7; IR (neat, cm^{-1}): 3489, 2953, 2875, 1754, 1514, 1249, 1209, 1034, 831; ESI HRMS *m/z* $(M+Na)^+$ calcd 479.2153, obsd 479.2157.

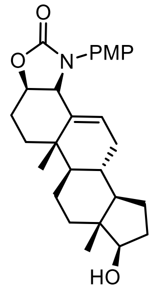

(3a*R*,5a*S*,5b*R*,7a*R*,8*R*,10a*R*,10b*S*,12b*R*)-8-Hydroxy-1-(4-methoxyphenyl)-5a,7a-dimethyl -1,3a,4,5,5a,5b,6,7,7a,8,9,10,10a,10b,11,12b-hexadecahydro-2*H*-cyclopenta[7,8] phenanthro[1,2-*d*]oxazol-2-one (23). Light yellow solid; Yield = 93%; Electricity = 4.7 F mol^{-1}; ^{1}H NMR (600 MHz, $CDCl_3$) δ 7.27–7.22 (m, 2H), 6.91–6.84 (m, 2H), 5.71 (dd, J = 4.7, 2.7 Hz, 1H), 4.78 (ddd, J = 8.8, 5.8, 4.6 Hz, 1H), 4.72 (d, J = 8.7 Hz, 1H), 3.79 (s, 3H), 3.62 (t, J = 8.6 Hz, 1H), 2.12–1.98 (m, 2H), 1.92–1.82 (m, 2H), 1.79–1.68 (m, 2H), 1.64–1.50 (m, 4H), 1.53–1.41 (m, 3H), 1.41–1.32 (m, 1H), 1.25 (ddq, J = 18.6, 12.3, 6.1 Hz, 1H), 1.07 (td, J = 12.9, 4.0 Hz, 1H), 1.00–0.92 (m, 2H), 0.90 (s, 3H), 0.70 (s, 3H); ^{13}C NMR (151 MHz, $CDCl_3$) δ 157.1, 157.0, 135.7, 133.0, 130.4, 124.7, 114.1, 81.7, 74.2, 64.2, 55.5, 51.7, 47.7, 42.9, 36.5(2C), 31.6(2C), 30.6, 29.9, 25.3, 23.3, 21.8, 20.4, 11.1; IR (neat, cm^{-1}):3421, 2949, 1743, 1614, 1248, 1031, 829, 734; ESI HRMS *m/z* $(M+Na)^+$ calcd 460.2458, obsd 460.2465.

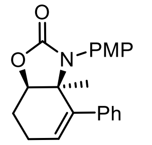

3-(4-Methoxyphenyl)-3a-methyl-4-phenyl-3a,6,7,7a-tetrahydrobenzo[*d*]oxazol-2(3*H*)-one (24). Light yellow solid; Yield = 77%; Electricity = 7.5 F mol^{-1}; ^{1}H NMR (600 MHz, $CDCl_3$) δ 7.15–7.12 (m, 1H), 7.11–7.06 (m, 2H), 6.89–6.83 (m, 2H), 6.78–6.73 (m, 2H), 6.66–6.61 (m, 2H), 5.93 (dd, J = 6.0, 2.8 Hz, 1H), 4.61 (t, J = 3.5 Hz, 1H), 3.71 (s, 3H), 2.51 (dddd, J = 17.5, 11.3, 5.1, 2.8 Hz, 1H), 2.35–2.28 (m, 1H), 2.28–2.20 (m, 1H), 1.95 (dddd, J = 14.3, 11.2, 5.6, 3.1 Hz, 1H), 1.63 (s, 3H); ^{13}C NMR (151 MHz, $CDCl_3$) δ 158.8, 157.6, 140.7, 140.3, 132.1, 130.5, 129.7, 128.6, 127.8, 127.2, 114.0, 82.4, 63.5, 55.6, 25.4, 24.5, 20.7; IR (neat, cm^{-1}): 2933, 1750, 1513, 1249, 765, 704; ESI HRMS *m/z* $(M+Na)^+$ calcd 358.1414, obsd 358.1421.

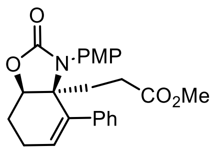

Methyl 3-(3-(4-methoxyphenyl)-2-oxo-4-phenyl-2,3,7,7a-tetrahydrobenzo[*d*]oxazol-3a(6*H*)-yl)propanoate (25). Light yellow oil; Yield = 95%; Electricity = 7.5 F mol^{-1}; ^{1}H NMR (500 MHz, $CDCl_3$) δ 7.16–7.11 (m, 1H), 7.11–7.03 (m, 2H), 6.86–6.78 (m, 2H), 6.72–6.64 (m, 4H), 6.16 (dd, *J* = 6.2, 3.2 Hz, 1H), 4.75–4.64 (m, 1H), 3.74 (s, 3H), 3.67 (s, 3H), 2.54–2.45 (m, 1H), 2.44–2.34 (m, 3H), 2.31–2.24 (m, 2H), 2.24–2.16 (m, 1H), 1.90–1.80 (m, 1H); ^{13}C NMR (126 MHz, $CDCl_3$) δ 172.9, 159.2, 157.4, 140.1, 138.9, 135.5, 130.8, 129.3, 128.3, 128.0, 127.3, 114.4, 79.7, 65.5, 55.6, 52.1, 32.5, 29.1, 26.7, 20.6; IR (neat, cm^{-1}): 3455, 2952, 1748, 1514, 1250, 1175, 831, 763; ESI HRMS *m/z* $(M+Na)^+$ calcd 430.1625, obsd 430.1639.

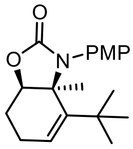

4-(*tert*-Butyl)-3-(4-methoxyphenyl)-3a-methyl-3a,6,7,7a-tetrahydrobenzo[*d*]oxazol-2(3*H*)-one (26). Colorless oil; Yield = 53%; Electricity = 8.9 F mol^{-1}; ^{1}H NMR (500 MHz, $CDCl_3$) δ 7.09–7.01 (m, 2H), 6.90–6.84 (m, 2H), 6.33 (dd, *J* = 7.9, 3.3 Hz, 1H), 4.50–4.43 (m, 1H), 3.79 (s, 3H), 2.38 (ddt, *J* = 15.9, 12.1, 3.7 Hz, 1H), 2.21–2.13 (m, 1H), 2.12–2.04 (m, 1H), 1.84 (s, 3H), 1.55 (ddt, *J* = 14.0, 12.2, 4.7 Hz, 1H), 0.81 (s, 9H); ^{13}C NMR (151 MHz, $CDCl_3$) δ 159.7, 157.8, 145.9, 131.6, 131.4, 129.6, 114.5, 84.1, 65.7, 55.6, 36.7, 31.9, 28.8, 27.2, 21.1; IR (neat, cm^{-1}): 3444, 2955, 1748, 1502, 1257, 762; ESI HRMS *m/z* $(M+Na)^+$ calcd 338.1727, obsd 338.1739.

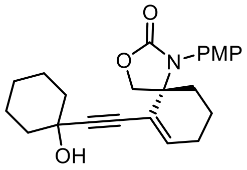

6-((1-Hydroxycyclohexyl)ethynyl)-1-(4-methoxyphenyl)-3-oxa-1-azaspiro[4.5]dec-6-en-2-one (27). Light yellow foam solid; Yield = 54%; Electricity = 4.6 F mol^{-1}; ^{1}H NMR (600 MHz, $CDCl_3$) δ 7.35–7.31 (m, 2H), 6.92–6.88 (m, 2H), 6.31 (dd, *J* = 4.9, 3.5 Hz, 1H), 4.49, 4.21 (ABq, J_{AB} = 8.5 Hz, 2H), 3.80 (s, 3H), 2.48 (brs, 1H), 2.08–2.00 (m, 1H), 2.00–1.92 (m, 3H), 1.91–1.87 (m, 1H), 1.86–1.80 (m, 1H), 1.72–1.66 (m, 2H), 1.64–1.58 (m, 2H), 1.55–1.45 (m, 5H), 1.24–1.19 (m, 1H); ^{13}C NMR (151 MHz, $CDCl_3$) δ 159.1, 157.7, 139.7, 129.7, 128.2, 124.0, 114.6, 95.6, 81.3, 74.3, 69.1, 64.0, 55.6, 39.9 (2C), 33.3, 29.8, 25.3, 25.2, 23.4, 19.0; IR (neat, cm^{-1}): 3421, 2934, 1735, 1514, 1249, 836; ESI HRMS *m/z* $(M+Na)^+$ calcd 404.1832, obsd 404.1837.

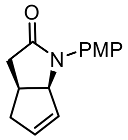

1-(4-Methoxyphenyl)-3,3a,4,6a-tetrahydrocyclopenta[*b*]pyrrol-2(1*H*)-one (28). Light yellow oil; Yield = 91%; Electricity = 10.3 F mol^{-1}; ^{1}H NMR (600 MHz, $CDCl_3$) δ 7.46–7.38 (m, 2H), 6.96–6.87 (m, 2H), 5.97 (dtd, *J* = 5.9, 2.3, 1.0 Hz, 1H), 5.84–5.78 (m, 1H), 5.09–5.01 (m, 1H), 3.80 (s, 3H), 3.06 (dddd, *J* = 13.6, 8.7, 7.3, 3.1 Hz, 1H), 2.88 (dd, *J* = 17.5, 10.3 Hz, 1H), 2.78 (ddtd, *J* = 17.2, 8.5, 2.3, 1.4 Hz, 1H), 2.39 (dd, *J* = 17.5, 6.0 Hz, 1H), 2.35–2.27 (m, 1H); ^{13}C NMR (151 MHz, $CDCl_3$) δ 173.4, 157.3, 134.9, 131.8, 129.0, 124.3, 114.4, 71.2, 55.6, 39.8 (2C), 32.4; IR (neat, cm^{-1}): 3410, 1693, 1632, 1514, 1249, 832; ESI HRMS *m/z* (M+Na)$^+$ calcd 252.0995, obsd 252.0972.

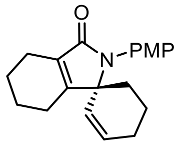

2'-(4-Methoxyphenyl)-4',5',6',7'-tetrahydrospiro[cyclohexane-1,1'-isoindol]-2-en-3'(2'*H*)-one (29). Colorless oil; Yield = 55%; Electricity = 9.7 F mol^{-1}; ^{1}H NMR (600 MHz, $CDCl_3$) δ 7.20–7.14 (m, 2H), 6.91–6.86 (m, 2H), 6.08 (dt, *J* = 10.0, 3.9 Hz, 1H), 5.34 (dt, *J* = 10.0, 2.1 Hz, 1H), 3.80 (s, 3H), 2.38–2.30 (m, 1H), 2.30–2.16 (m, 3H), 1.97–1.86 (m, 2H), 1.87–1.71 (m, 4H), 1.69–1.58 (m, 3H), 1.35–1.27 (m, 1H); ^{13}C NMR (151 MHz, $CDCl_3$) δ 171.1, 158.6, 158.2, 132.9, 130.4, 130.1, 128.3, 114.3, 67.9, 55.5, 30.7, 23.8, 23.2, 22.7, 22.2, 20.6, 19.4; IR (neat, cm^{-1}): 3444, 2933, 1686, 1512, 1247, 844; ESI HRMS *m/z* (M+Na)$^+$ calcd 332.1621, obsd 332.1614.

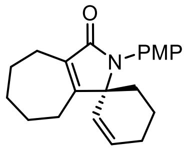

2-(4-Methoxyphenyl)-5,6,7,8-tetrahydro-2*H*-spiro[cyclohepta[*c*]pyrrole-1,1'-cyclohexan]-2'-en-3(4*H*)-one (30). Light yellow solid; Yield = 65%; Electricity = 13.0 F mol^{-1}; ^{1}H NMR (600 MHz, $CDCl_3$) δ 7.17–7.10 (m, 2H), 6.91–6.85 (m, 2H), 6.13 (dt, *J* = 10.0, 4.0 Hz, 1H), 5.38–5.30 (m, 1H), 3.80 (s, 3H), 2.54 (ddd, *J* = 16.2, 8.1, 2.9 Hz, 1H), 2.43 (ddd, *J* = 16.2, 9.1, 3.0 Hz, 1H), 2.33–2.25 (m, 2H), 1.92–1.84 (m, 3H), 1.83–1.79 (m, 1H), 1.79–1.71 (m, 2H), 1.70–1.63 (m, 2H), 1.60–1.47 (m, 3H), 1.06–0.95 (m, 1H); ^{13}C NMR (151 MHz, $CDCl_3$) δ 171.5, 161.1, 158.7, 133.8, 133.7, 130.5, 130.3, 127.1, 114.3, 67.2, 55.5, 31.5, 31.1, 28.2, 27.4, 27.1, 24.7, 23.9, 18.7; IR (neat, cm^{-1}): 3425, 2924, 1684, 1512, 596, 579; ESI HRMS *m/z* (M+Na)$^+$ calcd 346.1778, obsd 346.1782.

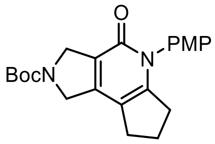

***tert*-Butyl 5-(4-methoxyphenyl)-4-oxo-3,4,5,6,7,8-hexahydrocyclopenta[*b*]pyrrolo[3,4-d]pyridine-2(1*H*)-carboxylate (31).** Light yellow oil; Yield = 89%; Isolated as a 1:1 mixture of rotary isomers; Electricity = 7.1 F mol^{-1}; ^{1}H NMR (500 MHz, acetone-d_6) δ 7.25–7.19 (m, 2H), 7.08–7.02 (m, 2H), 4.64–4.57 (m, 1H), 4.57–4.50 (m, 1H), 4.44–4.34 (m, 2H), 3.89–3.83 (m, 3H), 2.78 (t, *J* = 7.5 Hz, 2H), 2.54 (t, *J* = 7.6 Hz, 2H), 2.10–2.03 (m, 2H), 1.54–1.48 (m, 9H); ^{13}C NMR (126 MHz, acetone-d_6) δ 161.2, 155.4 (2C), 152.9, 152.8, 148.1, 147.6, 133.2, 130.3, 124.4, 124.0, 115.9, 115.2, 115.1, 80.7 (2C), 56.6, 54.0, 53.8, 52.7, 52.5, 34.4, 30.0, 29.4, 23.9; IR (neat, cm^{-1}): 3440, 1659, 1510, 1396, 1250, 1172, 842; ESI HRMS *m/z* (M+Na)$^{+}$ calcd 405.1785, obsd 305.1791.

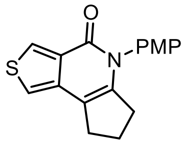

5-(4-Methoxyphenyl)-5,6,7,8-tetrahydro-4*H*-cyclopenta[*b*]thieno[3,4-d]pyridin-4-one (32). Light yellow oil; Yield = 85%; Electricity = 4.2 F mol^{-1}; ^{1}H NMR (600 MHz, $CDCl_3$) δ 8.38 (d, *J* = 3.2 Hz, 1H), 7.20–7.15 (m, 2H), 7.13 (d, *J* = 3.2 Hz, 1H), 7.00–6.96 (m, 2H), 3.84 (s, 3H), 2.94–2.85 (m, 2H), 2.46 (tt, *J* = 8.2, 2.0 Hz, 2H), 2.12–2.05 (m, 2H); ^{13}C NMR (151 MHz, $CDCl_3$) δ 160.4, 159.4, 141.0, 137.1, 131.8, 131.3, 130.3, 129.3, 114.7, 114.2, 111.8, 55.6, 33.2, 29.8, 21.9; IR (neat, cm^{-1}): 3440, 1651, 1509, 1248, 537; ESI HRMS *m/z* (M+Na)$^{+}$ calcd 320.0716, obsd 320.0724.

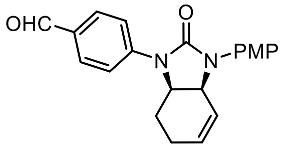

4-(3-(4-Methoxyphenyl)-2-oxo-2,3,3a,6,7,7a-hexahydro-1*H*-benzo[*d*]imidazol-1-yl)benzaldehyde (33). Light yellow solid; Yield = 62%; Electricity = 8.1 F mol^{-1}; ^{1}H NMR (500 MHz, $CDCl_3$) δ 9.92 (s, 1H), 7.93–7.83 (m, 2H), 7.81–7.73 (m, 2H), 7.36–7.28 (m, 2H), 7.04–6.86 (m, 2H), 6.14–6.05 (m, 1H), 5.83–5.66 (m, 1H), 4.65–4.58 (m, 1H), 4.54 (td, *J* = 8.3, 7.9, 3.4 Hz, 1H), 3.82 (s, 3H), 2.19–2.00 (m, 3H), 1.94–1.83 (m, 1H); ^{13}C NMR (126 MHz, $CDCl_3$) δ 191.2, 157.7, 155.5, 144.6, 132.5, 131.2, 131.1, 130.5, 125.6, 122.2, 119.4, 114.6, 55.7, 53.0, 52.5, 22.9, 21.9; IR (neat, cm^{-1}): 3417, 1694, 1509, 1377, 830, 599; ESI HRMS *m/z* (M+Na)$^{+}$ calcd 371.1366, obsd 371.1380.

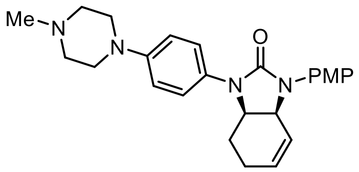

1-(4-Methoxyphenyl)-3-(4-(4-methylpiperazin-1-yl)phenyl)-1,3,3a,4,5,7a-hexahydro-2*H*-benzo[*d*]imidazol-2-one (34). Light yellow oil; Yield = 59%; Electricity = 5.2 F mol^{-1}; ^{1}H NMR (500 MHz, $CDCl_3$) δ 7.43–7.37 (m, 2H), 7.33–7.28 (m, 2H), 6.97–6.92 (m, 2H), 6.92–6.87 (m, 2H), 6.01 (dt, *J* = 9.6, 3.6 Hz, 1H), 5.72 (ddt, *J* = 10.3, 3.5, 1.8 Hz, 1H), 4.62 (ddd, *J* = 7.9, 3.0, 1.5 Hz, 1H), 4.40 (ddt, *J* = 9.9, 6.3, 3.3 Hz, 1H), 3.79 (s, 3H), 3.23–3.18 (m, 4H), 2.59 (t, *J* = 5.0 Hz, 4H), 2.36 (s, 3H), 2.15–2.05 (m, 1H), 1.96–1.80 (m, 3H); ^{13}C NMR (126 MHz, $CDCl_3$) δ 156.8, 156.5, 148.7, 131.9, 131.8, 130.4, 124.3, 123.9, 122.5, 116.6, 114.3, 55.6, 55.2, 53.5, 52.7, 49.5, 46.3, 22.9, 20.9; IR (neat, cm^{-1}): 2927, 2848, 1698, 1510, 1269, 826; ESI HRMS *m/z* $(M+H)^+$ calcd 419.2442, obsd 419.2447.

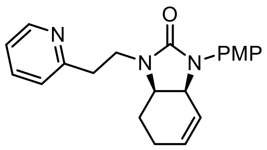

1-(4-Methoxyphenyl)-3-(2-(pyridin-2-yl)ethyl)-1,3,3a,4,5,7a-hexahydro-2*H*-benzo[*d*]imidazol-2-one (36). Light yellow oil; Yield = 86%; Electricity = 4.7 F mol^{-1}; ^{1}H NMR (500 MHz, $CDCl_3$) δ 8.52 (ddd, *J* = 4.8, 1.9, 0.9 Hz, 1H), 7.65–7.52 (m, 1H), 7.35–7.30 (m, 2H), 7.28–7.25 (m, 1H), 7.13 (ddd, *J* = 7.5, 4.9, 1.2 Hz, 1H), 6.92–6.84 (m, 2H), 5.97–5.90 (m, 1H), 5.63 (ddt, *J* = 9.1, 2.8, 1.6 Hz, 1H), 4.42 (ddd, *J* = 7.9, 3.0, 1.5 Hz, 1H), 3.91–3.85 (m, 1H), 3.82–3.77 (m, 4H), 3.46 (ddd, *J* = 14.3, 8.5, 6.0 Hz, 1H), 3.16–3.00 (m, 2H), 2.09–1.98 (m, 1H), 1.96–1.81 (m, 3H); ^{13}C NMR (126 MHz, $CDCl_3$) δ 159.5, 158.6, 156.3, 149.3, 136.7, 132.0, 131.6, 123.7, 123.6, 122.5, 121.6, 114.3, 55.6, 52.8, 52.2, 41.4, 36.6, 22.7, 20.8; IR (neat, cm^{-1}): 3431, 2929, 1689, 1513, 1433, 1247, 1181, 844, 754; ESI HRMS *m/z* $(M+Na)^+$ calcd 372.1682, obsd 372.1690.

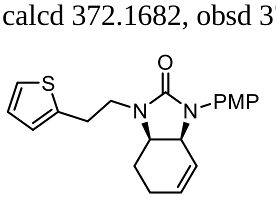

1-(4-Methoxyphenyl)-3-(2-(thiophen-2-yl)ethyl)-1,3,3a,4,5,7a-hexahydro-2*H*-benzo[*d*]imidazol-2-one (37). Light yellow oil; Yield = 47%; Electricity = 4.8 F mol^{-1}; ^{1}H NMR (500 MHz, $CDCl_3$) δ 7.35–7.29 (m, 2H), 7.15 (dd, *J* = 5.2, 1.2 Hz, 1H), 6.93 (dd, *J* = 5.1, 3.4 Hz, 1H), 6.91–6.85 (m, 3H), 5.96 (dtd, *J* = 10.2, 3.9, 1.1 Hz, 1H), 5.66 (ddt, *J* = 10.1, 3.6, 2.0 Hz, 1H), 4.44 (ddd, *J* = 8.1, 3.2, 1.6 Hz, 1H), 3.85–3.73 (m, 5H), 3.29 (ddd, *J* = 14.2, 8.6, 6.2 Hz, 1H), 3.18 (ddd, *J* = 14.8, 8.8, 6.2 Hz, 1H), 3.08 (ddd, *J* = 14.6, 8.3, 6.2 Hz, 1H), 2.14–2.05 (m, 1H), 1.98–1.90 (m, 1H), 1.90–1.78 (m, 2H); ^{13}C NMR (126 MHz, $CDCl_3$) δ 158.6, 156.5,

141.7, 132.0, 131.6, 127.1, 125.4, 123.9, 122.6, 114.4, 55.7, 52.9, 52.5, 43.2, 28.6, 22.9, 21.0; IR (neat, cm^{-1}): 3424, 2925, 2850, 1694, 1613, 1431, 1246, 828; ESI HRMS *m/z* (M+Na)$^+$ calcd 377.1294, obsd 377.1295.

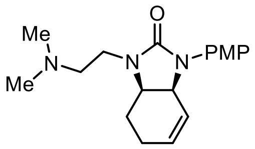

3-(2-(Dimethylamino)ethyl)-1-(4-methoxyphenyl)-1,3,3a,4,5,7a-hexahydro-2*H*-benzo[*d*]imidazol-2-one (38). Light yellow oil; Yield = 63%; Electricity = 11.1 F mol^{-1}; ^{1}H NMR (500 MHz, $CDCl_3$) δ 7.37–7.31 (m, 2H), 6.90–6.85 (m, 2H), 6.00–5.93 (m, 1H), 5.70–5.63 (m, 1H), 4.50 (ddt, *J* = 8.0, 3.1, 1.5 Hz, 1H), 3.95 (ddd, *J* = 7.9, 6.2, 3.8 Hz, 1H), 3.78 (s, 3H), 3.67 (ddd, *J* = 14.5, 8.0, 6.8 Hz, 1H), 3.13 (ddd, *J* = 14.3, 7.7, 5.5 Hz, 1H), 2.55–2.43 (m, 2H), 2.28 (s, 6H), 2.19–2.11 (m, 1H), 2.02–1.94 (m, 1H), 1.92–1.83 (m, 2H); ^{13}C NMR (126 MHz, $CDCl_3$) δ 158.6, 156.3, 132.1, 131.5, 123.6, 122.7, 114.3, 57.4, 55.6, 52.8, 52.2, 45.8, 39.3, 22.9, 21.0; IR (neat, cm^{-1}): 3385, 2934, 2834, 2776, 1694, 1513, 1430, 1245, 1035, 829; ESI HRMS *m/z* (M+H)$^+$ calcd 316.2020, obsd 316.2024.

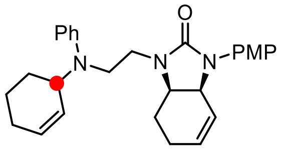

3-(2-(Cyclohex-2-en-1-yl(phenyl)amino)ethyl)-1-(4-methoxyphenyl)-1,3,3a,4,5,7a-hexahydro-2*H*-benzo[*d*]imidazol-2-one (39). The cyclization afforded only cis-fused products. But the title compound was isolated as a 1:1 mixture of diastereomers due to the presence of a stereocenter at the carbon marked with a red dot. Light yellow oil; Yield = 65%; Electricity = 6.5 F mol^{-1}; ^{1}H NMR (500 MHz, $CDCl_3$) δ 7.37–7.30 (m, 2H), 7.25–7.21 (m, 2H), 6.97–6.91 (m, 2H), 6.91–6.86 (m, 2H), 6.73–6.67 (m, 1H), 5.98–5.88 (m, 2H), 5.70–5.55 (m, 2H), 4.46 (ddt, *J* = 7.9, 3.1, 1.5 Hz, 1H), 4.43–4.34 (m, 1H), 3.94 (ddd, *J* = 7.9, 5.9, 3.9 Hz, 0.5H), 3.88 (ddd, *J* = 7.9, 5.4, 4.0 Hz, 0.5H), 3.78 (s, 3H), 3.63 (ddd, *J* = 16.1, 10.2, 4.7 Hz, 0.5H), 3.55–3.26 (m, 3H), 3.15 (ddd, *J* = 13.8, 9.7, 5.8 Hz, 0.5H), 2.19–2.05 (m, 2H), 2.04–1.89 (m, 3H), 1.87–1.77 (m, 3H), 1.70–1.56 (m, 2H); ^{13}C NMR (126 MHz, $CDCl_3$) δ 159.1, 158.8, 156.5 (2C), 148.5 (2C), 131.9, 131.4 (2C), 131.3, 131.2, 129.6 (2C), 129.5, 124.0 (2C), 122.7 (2C), 117.0, 116.9, 114.4, 113.9, 113.7, 55.8 (2C), 55.6, 53.3, 53.2 (2C), 52.7, 44.3, 43.7, 41.0, 40.2, 27.0, 26.8, 25.1 (2C), 23.4, 23.0, 21.8, 20.9, 20.8; IR (neat, cm^{-1}): 2927, 1698, 1512, 1429, 1246, 1181, 829, 749; ESI HRMS *m/z* (M+H)$^+$ calcd 444.2646, obsd 444.2658.

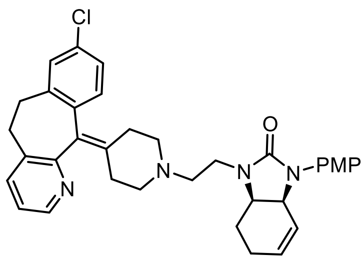

3-(2-(4-(8-Chloro-5,6-dihydro-11*H*-benzo[5,6]cyclohepta[1,2-*b*]pyridin-11-ylidene)piperidin-1-yl)ethyl)-1-(4-methoxyphenyl)-1,3,3a,4,5,7a-hexahydro-2*H*-benzo[*d*]imidazol-2-one (40). The cyclization afforded only cis-fused products. But the title compound was isolated as a 1:1 mixture of diastereomers due to the axial chiral tetrasubstituted alkene. Light yellow foamy solid; Yield = 61%; Electricity = 4.7 F mol^{-1}; ^{1}H NMR (500 MHz, $CDCl_3$) δ 8.46–8.34 (m, 1H), 7.43–7.40 (m, 1H), 7.36–7.32 (m, 2H), 7.15–7.10 (m, 3H), 7.08–7.05 (m, 1H), 6.91–6.85 (m, 2H), 5.95 (dt, *J* = 10.2, 3.9 Hz, 1H), 5.64 (dt, *J* = 10.5, 2.3 Hz, 1H), 4.53–4.42 (m, 1H), 4.05–3.93 (m, 1H), 3.78 (s, 3H), 3.70–3.56 (m, 1H), 3.45–3.30 (m, 2H), 3.21–3.09 (m, 1H), 2.88–2.72 (m, 4H), 2.63–2.55 (m, 1H), 2.55–2.46 (m, 2H), 2.41–2.29 (m, 3H), 2.23–2.09 (m, 3H), 1.99–1.92 (m, 1H), 1.92–1.84 (m, 2H); ^{13}C NMR (126 MHz, $CDCl_3$) δ 158.7, 157.7 (2C), 156.3, 156.2, 146.7, 139.7 (2C), 138.9 (2C), 137.9 (2C), 137.4 (2C), 133.5 (2C), 132.8, 132.7, 132.1, 131.5, 131.0, 130.9, 129.1, 129.0, 126.1, 123.6, 123.5, 122.6, 122.2, 114.3, 56.2, 56.1, 55.6, 55.3 (2C), 55.1, 55.0, 52.9 (2C), 52.5, 52.3, 38.9, 38.8, 31.9, 31.6, 31.3, 31.2, 31.0 (2C), 22.9(2C), 20.9; IR (neat, cm^{-1}): 3438, 3350, 1515, 1240, 878; ESI HRMS *m/z* (M+H)$^+$ calcd 581.2678, obsd 581.2693.

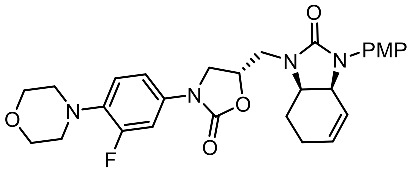

(5*S*)-3-(3-Fluoro-4-morpholinophenyl)-5-((3-(4-methoxyphenyl)-2-oxo-2,3,3a,6,7,7a-hexahydro-1H-benzo[*d*]imidazol-1-yl)methyl)oxazolidin-2-one (41). The cyclization afforded only cis-fused products. But the title compound was isolated as an isolated as a 5.6:1 mixture of diastereomers due to the sterocenter at the cyclic carbamate. Light yellow foamy solid; Yield = 49%; Electricity = 5.3 F mol^{-1}; ^{1}H NMR (500 MHz, $CDCl_3$) δ 7.51–7.43 (m, 1H), 7.33–7.28 (m, 0.3H), 7.16–7.11 (m, 1.7H), 7.11–7.04 (m, 1H), 6.91–6.81 (m, 3H), 6.04–5.94 (m, 1H), 5.68–5.56 (m, 1H), 4.91–4.78 (m, 1H), 4.54 (ddd, *J* = 7.9, 3.2, 1.6 Hz, 0.2H), 4.38 (ddd, *J* = 7.8, 3.3, 1.6 Hz, 1.8H), 4.10–4.05 (m, 1H), 4.03–3.92 (m, 1.7H), 3.89–3.83 (m, 4.3H), 3.80–3.75 (m, 4H), 3.56 (dd, *J* = 15.2, 3.6 Hz, 0.8H), 3.40–3.32 (m, 0.2H), 3.06–3.01 (m, 4H), 2.26–1.98 (m, 3H), 1.79–1.68 (m, 1H); ^{13}C NMR (126 MHz, $CDCl_3$) δ 159.5, 157.1, 155.60 (d, $J_{C\text{-}F}$ = 246.5 Hz), 154.6, 136.5 (d, $J_{C\text{-}F}$ = 9.0 Hz), 133.22 (d, $J_{C\text{-}F}$ = 10.4 Hz), 132.1, 131.1, 124.9, 122.1, 118.9 (d, $J_{C\text{-}F}$ = 4.2 Hz), 114.4, 114.1 (d, $J_{C\text{-}F}$ = 3.2 Hz), 107.7 (d, $J_{C\text{-}F}$ =

26.3 Hz), 73.0, 67.1, 55.6, 54.2, 53.2, 51.1, 51.1, 47.8, 43.7, 22.6, 21.5; ^{19}F NMR (471 MHz, $CDCl_3$) δ −120.3 (2F); IR (neat, cm^{-1}): 3406, 2956, 2923, 2851, 1751, 1514, 1245, 844; ESI HRMS *m/z* (M+Na)$^+$ calcd 545.2171, obsd 545.2177.

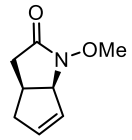

1-Methoxy-3,3a,4,6a-tetrahydrocyclopenta[*b*]pyrrol-2(1*H*)-one (42). Light yellow oil; Yield = 80%; Electricity = 4.7 F mol^{-1}; ^{1}H NMR (500 MHz, $CDCl_3$) δ 6.03–5.97 (m, 1H), 5.97–5.91 (m, 1H), 4.69 (d, *J* = 7.9 Hz, 1H), 3.83 (s, 3H), 2.99–2.89 (m, 1H), 2.82–2.73 (m, 1H), 2.66 (dd, *J* = 17.4, 10.3 Hz, 1H), 2.30–2.20 (m, 1H), 2.13 (dd, *J* = 17.4, 5.0 Hz, 1H); ^{13}C NMR (126 MHz, $CDCl_3$) δ 169.2, 135.3, 128.4, 67.1, 63.0, 40.3, 35.7, 30.7; IR (neat, cm^{-1}): 2673, 1686, 843, 559; ESI HRMS *m/z* (M+Na)$^+$ calcd 252.0995, obsd 252.0972.

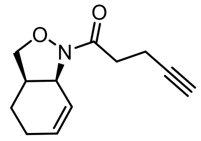

1-(3a,4,5,7a-Tetrahydrobenzo[*c*]isoxazol-1(3*H*)-yl)pent-4-yn-1-one (43). Colorless oil; Yield = 76%; Electricity = 4.7 F mol^{-1}; ^{1}H NMR (500 MHz, $CDCl_3$) δ 6.02–5.96 (m, 1H), 5.94–5.88 (m, 1H), 4.60 (ddt, *J* = 7.6, 3.6, 1.8 Hz, 1H), 3.96 (dd, *J* = 7.9, 6.1 Hz, 1H), 3.87 (dd, *J* = 7.9, 3.7 Hz, 1H), 2.81–2.72 (m, 1H), 2.72–2.65 (m, 2H), 2.59–2.45 (m, 2H), 2.16–2.07 (m, 1H), 2.04–1.95 (m, 2H), 1.89–1.81 (m, 1H), 1.69–1.59 (m, 1H); ^{13}C NMR (126 MHz, $CDCl_3$) δ 171.5, 130.6, 124.4, 83.6, 73.9, 68.7, 53.7, 39.2, 32.2, 22.8, 21.9, 13.9; IR (neat, cm^{-1}): 3455, 3293, 2926, 1644, 1435, 845; ESI HRMS *m/z* (M+Na)$^+$ calcd 228.0995, obsd 228.1001.

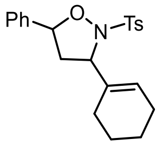

3-(Cyclohex-1-en-1-yl)-5-phenyl-2-tosylisoxazolidine (44). White solid; Yield = 60%; Isolated as a 3:2 mixture of diastereomers; Electricity = 2.8 F mol^{-1}; ^{1}H NMR (500 MHz, $CDCl_3$) δ 7.91–7.86 (m, 1.2H), 7.84–7.80 (m, 0.8H), 7.34–7.24 (m, 7H), 5.91–5.87 (m, 0.4H), 5.87–5.81 (m, 0.6H), 5.11 (td, *J* = 11.6, 11.2, 5.7 Hz, 1H), 4.78 (t, *J* = 8.2 Hz, 0.6H), 4.68 (d, *J* = 8.2 Hz, 0.4H), 2.71 (ddd, *J* = 12.4, 8.1, 5.8 Hz, 0.6H), 2.48–2.38 (m, 3.4H), 2.28–2.13 (m, 1.6H), 2.14–1.99 (m, 3.4H), 1.74–1.51 (m, 4H); ^{13}C NMR (126 MHz, $CDCl_3$) δ 145.0, 144.9, 137.4, 136.9, 135.4, 134.6, 133.6, 132.5, 129.8 (2C), 129.6, 129.4, 128.8, 128.7 (2C), 128.6, 127.5, 127.1, 124.8, 124.1, 83.3, 83.1, 65.8, 65.5, 42.5, 40.5, 26.0, 25.2, 25.1, 24.2, 22.7, 22.5, 22.4, 21.8 (2C); IR (neat, cm^{-1}): 2927, 1332, 1163, 699, 575; ESI HRMS *m/z* (M+Na)$^+$ calcd 406.1447, obsd 406.1455.

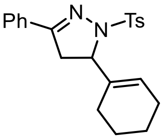

5-(Cyclohex-1-en-1-yl)-3-phenyl-1-tosyl-4,5-dihydro-1*H*-pyrazole (45). Colorless foamy solid; Yield = 82%; Electricity = 9.3 F mol^{-1}; ^{1}H NMR (500 MHz, $CDCl_3$) δ 7.82–7.77 (m, 2H), 7.69–7.63 (m, 2H), 7.43–7.33 (m, 3H), 7.30–7.26 (m, 2H), 5.78 (td, *J* = 3.8, 1.8 Hz, 1H), 4.26 (dd, *J* = 11.0, 9.8 Hz, 1H), 3.09, 2.97 (ABX, 2H, J_{AB} = 17.1 Hz, J_{AX} = 11.0 Hz, J_{BX} = 9.8 Hz), 2.38 (s, 3H), 2.18–2.07 (m, 2H), 2.07–1.98 (m, 1H), 1.96–1.87 (m, 1H), 1.68–1.60 (m, 3H), 1.59–1.49 (m, 1H); ^{13}C NMR (126 MHz, $CDCl_3$) δ 157.2, 144.1, 135.3, 132.9, 131.1, 130.5, 129.6, 128.7, 126.9, 126.1, 68.3, 39.2, 25.3, 23.5, 22.5, 22.4, 21.7; IR (neat, cm^{-1}): 2927, 1597, 1447, 1359, 1170, 1092, 693, 597; ESI HRMS *m/z* (M+Na)$^+$ calcd 403.1451, obsd 403.1449.

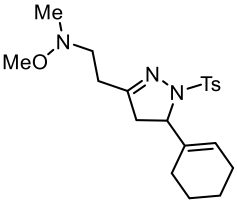

***N*-(2-(5-(Cyclohex-1-en-1-yl)-1-tosyl-4,5-dihydro-1*H*-pyrazol-3-yl)ethyl)-*N*,*O*-dimethylhydroxylamine (46).** Light yellow oil; Yield = 72%; Electricity = 9.3 F mol^{-1}; ^{1}H NMR (500 MHz, $CDCl_3$) δ 7.76–7.70 (m, 2H), 7.31–7.28 (m, 2H), 5.77–5.63 (m, 1H), 4.08 (t, *J* = 10.3 Hz, 1H), 3.37 (s, 3H), 2.84–2.69 (m, 2H), 2.68–2.58 (m, 2H), 2.55–2.49 (m, 5H), 2.42 (s, 3H), 2.16–2.05 (m, 2H), 2.03–1.95 (m, 1H), 1.91–1.83 (m, 1H), 1.67–1.58 (m, 3H), 1.57–1.48 (m, 1H); ^{13}C NMR (126 MHz, $CDCl_3$) δ 161.3, 143.9, 135.4, 132.8, 129.4, 128.8, 125.7, 67.5, 60.1, 57.1, 45.1, 41.8, 28.5, 25.2, 23.5, 22.4 (2C), 21.7; IR (neat, cm^{-1}): 2929, 1359, 1169, 817, 695, 547; ESI HRMS *m/z* (M+Na)$^+$ calcd 414.1822, obsd 414.1832.

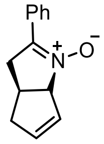

2-Phenyl-3,3a,4,6a-tetrahydrocyclopenta[*b*]pyrrole 1-oxide (47). Brown solid; Yield = 72%; Electricity = 2.8 F mol^{-1}; ^{1}H NMR (500 MHz, $CDCl_3$) δ 8.40–8.22 (m, 2H), 7.56–7.34 (m, 3H), 6.32–6.16 (m, 1H), 6.06 (dd, *J* = 5.5, 2.6 Hz, 1H), 5.29–5.12 (m, 1H), 3.70–3.35 (m, 1H), 3.25–3.00 (m, 1H), 2.97–2.71 (m, 2H), 2.50–2.23 (m, 1H); ^{13}C NMR (126 MHz, $CDCl_3$) δ 136.0, 130.4, 129.4, 128.5, 128.0, 127.4, 86.6, 41.5, 38.7, 31.1; IR (neat, cm^{-1}): 3380, 2923, 1375, 761, 692; ESI HRMS *m/z* (M+Na)$^+$ calcd 222.0889, obsd 222.0898.

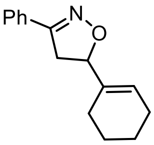

5-(Cyclohex-1-en-1-yl)-3-phenyl-4,5-dihydroisoxazole (48). Light yellow oil; Yield = 84%; Electricity = 3.7 F mol^{-1}; ^{1}H NMR (500 MHz, $CDCl_3$) δ 7.62–7.55 (m, 2H), 7.35–7.28 (m, 3H), 5.74 (td, *J* = 3.7, 1.8 Hz, 1H), 5.01 (dd, *J* = 11.0, 8.7 Hz, 1H), 3.30, 3.09 (ABX, 2H, J_{AB} = 16.6 Hz, J_{AX} = 11.0 Hz, J_{BX} = 8.8 Hz), 2.07–1.92 (m, 3H), 1.91–1.81 (m, 1H), 1.66–1.52 (m, 3H), 1.51–1.41 (m, 1H); ^{13}C NMR (126 MHz, $CDCl_3$) δ 156.4, 135.6, 130.0 (2C), 128.8, 126.8, 126.1, 85.7, 38.6, 25.2, 22.9, 22.4; IR (neat, cm^{-1}): 2926, 1446, 1356, 913, 759, 692; ESI HRMS *m/z* (M+Na)$^+$ calcd 250.1202, obsd 250.1212.

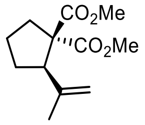

Dimethyl 2-(prop-1-en-2-yl)cyclopentane-1,1-dicarboxylate (49). Yiled = 43%; Colorless oil; Electricity =2.4 F mol^{-1}; ^{1}H NMR (400 MHz, $CDCl_3$) δ 4.83–4.80 (m, 1H), 4.76 (s, 1H), 3.73 (s, 3H), 3.62 (s, 3H), 3.35 (dd, *J* = 9.6, 6.9 Hz, 1H), 2.61 (ddd, *J* = 13.8, 9.6, 8.1 Hz, 1H), 2.08–2.00 (m, 1H), 1.92–1.82 (m, 2H), 1.81–1.76 (m, 1H), 1.75 (s, 3H), 1.61–1.52 (m, 1H); ^{13}C NMR (101 MHz, $CDCl_3$) δ 173.5, 171.6, 145.4, 112.4, 64.5, 52.7, 52.2, 52.1, 35.4, 30.9, 23.7, 23.2; IR (neat, cm^{-1}): 2954, 2876, 1732, 1644, 1435, 1265, 1158, 805; ESI HRMS *m/z* (M+Na)$^+$ calcd 249.1097, obsd 249.1096.

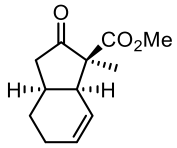

Methyl 1-methyl-2-oxo-2,3,3a,4,5,7a-hexahydro-1*H*-indene-1-carboxylate (50). Yield = 71%; Isolated as a 7:1 mixture of diastereomers; Colorless oil; Electricity = 2.5 F mol^{-1}; Spectrum of major diastereoisomer: ^{1}H NMR (500 MHz, $CDCl_3$) δ 5.93–5.88 (m, 1H), 5.62–5.56 (m, 1H), 3.71 (s, 3H), 3.24–3.19 (m, 1H), 2.67 (dd, *J* = 18.2, 8.7 Hz, 1H), 2.61–2.54 (m, 1H), 2.20 (dd, *J* = 18.2, 5.5 Hz, 1H), 2.09–2.04 (m, 2H), 1.78–1.69 (m, 1H), 1.51–1.43 (m, 1H), 1.26 (s, 3H); (2 stereoisomers) ^{13}C NMR (126 MHz, $CDCl_3$) δ 216.0, 215.4, 173.4, 172.3, 129.9, 129.2, 124.7, 124.2, 59.7, 59.0, 52.8, 52.0, 47.5, 43.8, 42.8, 42.3, 31.5, 31.4, 24.8, 24.4, 22.5, 22.2, 21.4, 17.6; IR (neat, cm^{-1}): 2959, 2872, 1740, 1715, 1456, 1260, 750; ESI HRMS *m/z* (M+Na)$^+$ calcd 231.0992, obsd 231.0991.

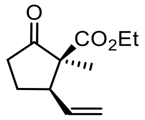

Ethyl 1-methyl-2-oxo-5-vinylcyclopentane-1-carboxylate (52). Yield = 60%; Isolated as a 2:1 mixture of diastereomers; Colorless oil; Electricity = 2.5 F mol^{-1}; ^{1}H NMR (400 MHz, $CDCl_3$) δ 5.82–5.71 (m, 1.5H), 5.23–5.09 (m, 3H), 4.26–4.03 (m, 3H), 3.35 (dt, *J* = 12.0, 7.0 Hz, 0.5H), 2.67–2.54 (m, 2H), 2.48–2.41 (m, 1H), 2.36–2.27 (m, 1H), 2.21–2.04 (m, 2.5H), 1.88–1.75 (m, 0.5H), 1.29 (s, 3H), 1.26 (t, *J* = 7.1 Hz, 1.5H), 1.24 (t, *J* = 8.1 Hz, 3H), 1.15 (s,

1.5H); ^{13}C NMR (101 MHz, $CDCl_3$) δ 215.9, 215.1, 172.1, 170.3, 136.0, 135.7, 117.5, 61.5, 61.2, 59.9, 59.5, 53.2, 48.8, 37.6, 37.3, 25.6, 24.8, 18.4, 14.3 (2C), 14.1; IR (neat, cm^{-1}): 2982, 2935, 1753, 1731, 1456, 1376, 1234, 921; ESI HRMS *m/z* $(M+Na)^+$ calcd 219.0992, obsd 219.0991.

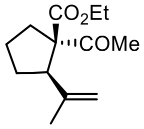

Ethyl 1-acetyl-2-(prop-1-en-2-yl) cyclopentane-1-carboxylate (53). Yield = 84%; Isolated as a 2.5:1 mixture of diastereomers; Colorless oil; Electricity = 2.4 F mol^{-1}; ^1H NMR (400 MHz, $CDCl_3$) δ 4.85 (p, *J* = 1.5 Hz, 0.4H), 4.82–4.80 (m, 0.4H), 4.78 (p, *J* = 1.6 Hz, 1H), 4.75–4.72 (m, 1H), 4.25–4.01 (m, 2.8H), 3.47–3.38 (m, 1.4H), 2.63–2.53 (m, 1.4H), 2.18 (s, 3H), 2.09 (s, 1.2H), 2.01–1.78 (m, 5.6H), 1.72–1.69 (m, 4.2H), 1.58–1.41 (m, 1.4H), 1.27 (t, *J* = 7.1 Hz, 1.2H), 1.23 (t, *J* = 7.2 Hz, 3H); ^{13}C NMR (101 MHz, $CDCl_3$) δ 204.4, 203.9, 173.7, 172.0, 145.6, 145.0, 114.2, 112.1, 71.1, 70.6, 61.6, 61.2, 52.1, 49.7, 34.0, 33.7, 31.3, 31.0, 29.1, 27.3, 23.7, 23.6, 23.3, 22.3, 14.1 (2C); IR (neat, cm^{-1}): 2962, 2875, 1712, 1644, 1449, 1355, 1248, 1153, 894; ESI HRMS *m/z* $(M+Na)^+$ calcd 247.1305, obsd 247.1303.

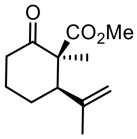

Methyl 1-methyl-2-oxo-6-(prop-1-en-2-yl)cyclohexane-1-carboxylate (55). Yield = 79%; Colorless oil; Electricity = 2.5 F mol^{-1}; ^1H NMR (400 MHz, $CDCl_3$) δ 4.90–4.85 (m, 1H), 4.76 (s, 1H), 3.67 (s, 3H), 3.00 (td, *J* = 13.7, 6.8 Hz, 1H), 2.50–2.34 (m, 2H), 2.24 (dd, *J* = 13.1, 3.1 Hz, 1H), 2.15–2.07 (m, 1H), 1.69 (s, 3H), 1.73–1.62 (m, 2H), 1.32 (s, 3H); ^{13}C NMR (101 MHz, $CDCl_3$) δ 207.9, 171.6, 144.5, 114.8, 60.5, 57.0, 52.2, 39.6, 27.1, 25.3, 21.2, 19.2; IR (neat, cm^{-1}): 2951, 2869, 1739, 1709, 1453, 1219, 900; ESI HRMS *m/z* $(M+Na)^+$ calcd 233.1148, obsd 233.1148.

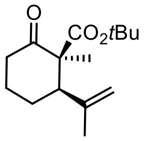

***tert*-Butyl 1-methyl-2-oxo-6-(prop-1-en-2-yl)cyclohexane-1-carboxylate (56).** Yield = 56%; Colorless oil; Electricity = 2.8 F mol^{-1}; ^1H NMR (400 MHz, $CDCl_3$) δ 4.88 (s, 1H), 4.79 (s, 1H), 2.98–2.84 (m, 1H), 2.52–2.35 (m, 2H), 2.20 (dd, *J* = 13.0, 3.1 Hz, 1H), 2.15–2.04 (m, 1H), 1.77 (s, 3H), 1.71–1.61 (m, 2H), 1.44 (s, 9H), 1.27 (s, 3H); ^{13}C NMR (101 MHz, $CDCl_3$) δ 208.6, 170.3, 144.8, 114.7, 82.1, 61.4, 56.6, 39.5, 28.1, 27.3, 25.3, 22.0, 19.8; IR (neat, cm^{-1}): 2938, 2869, 1705, 1640, 1248, 847; ESI HRMS *m/z* $(M+Na)^+$ calcd 275.1618, obsd 275.1618.

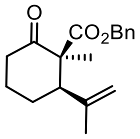

Benzyl 1-methyl-2-oxo-6-(prop-1-en-2-yl)cyclohexane-1-carboxylate (57). Yield = 61%; Colorless oil; Electricity = 2.8 F mol^{-1}; ^{1}H NMR (500 MHz, $CDCl_3$) δ 7.37–7.29 (m, 5H), 5.11, 5.07 (ABq, J_{AB} = 12.4 Hz, 2H), 4.86–4.82 (m, 1H), 4.73 (s, 1H), 2.99–2.88 (m, 1H), 2.46–2.36 (m, 2H), 2.24 (dd, J = 13.0, 3.1 Hz, 1H), 2.11–2.04 (m, 1H), 1.68–1.61 (m, 5H), 1.35 (s, 3H); ^{13}C NMR (101 MHz, $CDCl_3$) δ 173.5, 171.6, 145.4, 112.4, 64.5, 52.7, 52.2, 52.1, 35.4, 30.9, 23.7, 23.2; IR (neat, cm^{-1}): 2942, 2869, 1709, 1455, 1208, 698; ESI HRMS *m/z* (M+Na)$^+$ calcd 219.0992, obsd 219.0991.

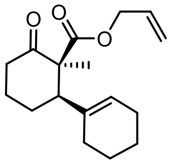

Allyl 2-methyl-3-oxo-[1,1'-bi(cyclohexan)]-1'-ene-2-carboxylate (58). Yield = 54%; Colorless oil; Electricity = 2.5 F mol^{-1}; ^{1}H NMR (400 MHz, $CDCl_3$) δ 5.89 (ddt, J = 17.1, 10.4, 5.7 Hz, 1H), 5.50–5.44 (m, 1H), 5.31 (dq, J = 17.1, 1.6 Hz, 1H), 5.24 (dq, J = 10.4, 1.3 Hz, 1H), 4.59–4.49 (m, 2H), 3.00 (td, J = 13.9, 6.7 Hz, 1H), 2.47–2.34 (m, 2H), 2.15–1.93 (m, 5H), 1.84–1.73 (m, 1H), 1.70–1.50 (m, 6H), 1.31 (s, 3H); ^{13}C NMR (101 MHz, $CDCl_3$) δ 208.6, 171.1, 136.9, 131.7, 125.9, 118.8, 65.8, 61.1, 57.8, 39.8, 27.5, 26.9, 25.5, 25.4, 23.2, 22.5, 19.4; IR (neat, cm^{-1}): 2941, 1741, 1717, 1453, 1359, 1196, 936; ESI HRMS *m/z* (M+H)$^+$ calcd 277.1798, obsd 277.1801.

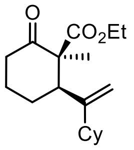

Ethyl 2-(1-cyclohexylvinyl)-1-methyl-6-oxocyclohexane-1-carboxylate (60). Yield = 56%; Colorless oil; Electricity = 2.5 F mol^{-1}; ^{1}H NMR (400 MHz, $CDCl_3$) δ 4.88 (s, 1H), 4.82 (s, 1H), 4.11 (2q, J = 7.1 Hz, 2H), 3.04 (ddd, J = 14.5, 13.4, 7.0 Hz, 1H), 2.48–2.38 (m, 2H), 2.15 (dd, J = 13.0, 3.2 Hz, 1H), 2.12–2.05 (m, 1H), 1.81–1.61 (m, 9H), 1.29–1.16 (m, 10H); ^{13}C NMR (101 MHz, $CDCl_3$) δ 208.6, 170.9, 155.4, 110.2, 61.9, 61.2, 55.5, 45.9, 39.5, 33.9, 32.6, 28.8, 27.2, 26.9, 26.4, 25.4, 19.3, 14.1; IR (neat, cm^{-1}): 2927, 2853, 1738, 1707, 1448, 1219, 1094; ESI HRMS *m/z* (M+Na)$^+$ calcd 315.1931, obsd 315.1930.

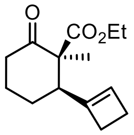

Ethyl 2-(cyclobut-1-en-1-yl)-1-methyl-6-oxocyclohexane-1-carboxylate (61). Yield = 61%; Colorless oil; Electricity = 2.5 F mol^{-1}; ^{1}H NMR (400 MHz, $CDCl_3$) δ 5.79 (s, 1H), 4.13 (q, J

= 7.1 Hz, 2H), 2.78 (td, J = 14.0, 6.4 Hz, 1H), 2.57 (dt, J = 13.1, 3.2 Hz, 1H), 2.52–2.41 (m, 2H), 2.36–2.31 (m, 2H), 2.26–2.16 (m, 2H), 2.15–2.05 (m, 1H), 1.83–1.77 (m, 1H), 1.72–1.60 (m, 1H), 1.39 (s, 3H), 1.25 (t, J = 7.1 Hz, 3H); ^{13}C NMR (101 MHz, $CDCl_3$) δ 208.0, 170.9, 148.8, 130.3, 61.2, 60.2, 50.3, 39.8, 32.0, 26.8, 26.2, 25.1, 19.6, 14.1; IR (neat, cm^{-1}): 3501, 2941, 2872, 1715, 1450, 803; ESI HRMS *m/z* $(M+Na)^+$ calcd 259.1305, obsd 259.1305.

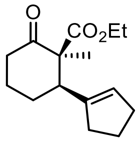

Ethyl 2-(cyclopent-1-en-1-yl)-1-methyl-6-oxocyclohexane-1-carboxylate (62). Yield = 66%; Colorless oil; Electricity = 2.5 F mol^{-1}; ^{1}H NMR (400 MHz, $CDCl_3$) δ 5.50–5.44 (m, 1H), 4.11 (2q, J = 7.1 Hz, 2H), 2.92 (ddd, J = 14.5, 13.5, 6.8 Hz, 1H), 2.48–2.40 (m, 2H), 2.39–2.24 (m, 4H), 2.22–2.13 (m, 1H), 2.13–2.05 (m, 1H), 1.86–1.76 (m, 2H), 1.72–1.61 (m, 2H), 1.31 (s, 3H), 1.25 (t, J = 7.1 Hz, 3H); ^{13}C NMR (101 MHz, $CDCl_3$) δ 208.4, 171.2, 143.4, 128.3, 61.2, 60.6, 51.4, 39.7, 34.1, 32.1, 27.4, 25.3, 23.9, 19.6, 14.1; IR (neat, cm^{-1}): 2939, 1713, 1450, 1247, 1019; ESI HRMS *m/z* $(M+Na)^+$ calcd 273.1461, obsd 273.1470.

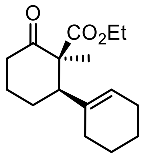

Ethyl 2-methyl-3-oxo-[1,1'-bi(cyclohexan)]-1'-ene-2-carboxylate (63). Yield = 78%; Colorless oil; Electricity = 2.5 F mol^{-1}; ^{1}H NMR (400 MHz, $CDCl_3$) δ 5.50–5.43 (m, 1H), 4.18–4.03 (m, 2H), 3.00 (ddd, J = 14.4, 13.3, 6.7 Hz, 1H), 2.48–2.33 (m, 2H), 2.13–1.93 (m, 5H), 1.84–1.74 (m, 1H), 1.71–1.50 (m, 6H), 1.28 (s, 3H), 1.26 (t, J = 7.1 Hz, 3H); ^{13}C NMR (101 MHz, $CDCl_3$) δ 208.7, 171.4, 136.9, 125.7, 61.2, 60.9, 57.7, 39.7, 27.4, 26.8, 25.4 (2C), 23.2, 22.5, 19.3, 14.1; IR (neat, cm^{-1}): 3416, 2938, 2868, 1708, 1449, 1215, 857; ESI HRMS *m/z* $(M+Na)^+$ calcd 287.1618, obsd 287.1618.

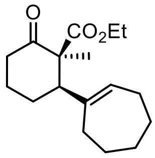

Ethyl 2-(cyclohept-1-en-1-yl)-1-methyl-6-oxocyclohexane-1-carboxylate (64). Yield = 60%; Colorless oil; Electricity = 2.5 F mol^{-1}; ^{1}H NMR (400 MHz, $CDCl_3$) δ 5.63 (t, J = 6.6 Hz, 1H), 4.14 (dq, J = 10.8, 7.1 Hz, 1H), 4.05 (dq, J = 10.8, 7.1 Hz, 1H), 3.04 (ddd, J = 14.6, 13.3, 6.9 Hz, 1H), 2.49–2.40 (m, 1H), 2.39–2.27 (m, 1H), 2.20–2.04 (m, 6H), 1.80–1.59 (m, 4H), 1.54–1.45 (m, 2H), 1.43–1.33 (m, 2H), 1.27 (s, 3H), 1.26 (t, J = 7.2 Hz, 3H); ^{13}C NMR (101 MHz, $CDCl_3$) δ 208.9, 171.3, 143.6, 131.3, 61.2, 60.7, 59.4, 40.0, 33.0, 30.7, 28.5, 27.3, 26.9, 26.7, 25.2, 19.4, 14.1; IR (neat, cm^{-1}): 2925, 2852, 1737, 1706, 1448, 1214; ESI HRMS *m/z* $(M+Na)^+$ calcd 301.1774, obsd 301.1775.

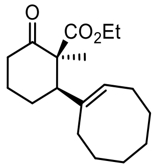

Ethyl (*E*)-2-(cyclooct-1-en-1-yl)-1-methyl-6-oxocyclohexane-1-carboxylate (65). Yield = 56%; Colorless oil; Electricity = 2.5 F mol^{-1}; ^{1}H NMR (400 MHz, $CDCl_3$) δ 5.44 (t, *J* = 8.2 Hz, 1H), 4.10 (2q, *J* = 7.2 Hz, 2H), 3.03 (td, *J* = 14.1, 6.7 Hz, 1H), 2.47–2.34 (m, 2H), 2.24–2.14 (m, 2H), 2.13–2.03 (m, 4H), 1.72–1.57 (m, 2H), 1.53–1.41 (m, 8H), 1.29 (s, 3H), 1.25 (t, *J* = 7.2 Hz, 3H); ^{13}C NMR (101 MHz, $CDCl_3$) δ 208.8, 171.2, 139.5, 128.5, 61.5, 61.1, 57.3, 39.8, 29.6, 29.5, 27.9, 26.8, 26.6, 26.2, 25.4, 19.3, 14.1; IR (neat, cm^{-1}): 2925, 2856, 1737, 1706, 1448, 1225, 1093; ESI HRMS *m/z* (M+Na)$^{+}$ calcd 315.1931, obsd 315.1931.

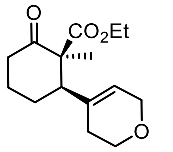

Ethyl 2-(3,6-dihydro-2H-pyran-4-yl)-1-methyl-6-oxocyclohexane-1-carboxylate (66). Yield = 75%; Colorless oil; Electricity = 2.5 F mol^{-1}; ^{1}H NMR (400 MHz, $CDCl_3$) δ 5.54–5.48 (m, 1H), 4.20–4.05 (m, 4H), 3.74 (t, *J* = 5.3 Hz, 2H), 2.93 (ddd, *J* = 14.5, 13.5, 6.9 Hz, 1H), 2.49–2.36 (m, 2H), 2.19–2.07 (m, 3H), 2.04–1.96 (m, 1H), 1.73–1.60 (m, 2H), 1.32 (s, 3H), 1.26 (t, *J* = 7.1 Hz, 3H); ^{13}C NMR (101 MHz, $CDCl_3$) δ 207.9, 171.2, 135.0, 124.5, 65.5, 64.5, 61.4, 60.8, 56.7, 39.6, 27.7, 26.7, 25.3, 19.4, 14.1; IR (neat, cm^{-1}): 2939, 1716, 1450, 1222, 1086; ESI HRMS *m/z* (M+Na)$^{+}$ calcd 289.1410, obsd 289.1411.

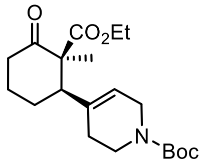

***tert*-Butyl 4-(2-(ethoxycarbonyl)-2-methyl-3-oxocyclohexyl)-3,6-dihydropyridine-1(2*H*)-carboxylate (67).** Yield = 77%; Pale yellow oil; Electricity = 2.6 F mol^{-1}; ^{1}H NMR (500 MHz, $CDCl_3$) δ 5.46 (s, 1H), 4.17–4.05 (m, 2H), 3.94–3.81 (m, 2H), 3.48–3.38 (m, 2H), 3.04–2.84 (m, 1H), 2.49–2.33 (m, 2H), 2.21–2.07 (m, 3H), 2.04–1.95 (m, 1H), 1.71–1.61 (m, 2H), 1.47 (s, 9H), 1.30 (s, 3H), 1.26 (t, *J* = 7.2 Hz, 3H); ^{13}C NMR (126 MHz, $CDCl_3$) δ 207.8, 171.1, 154.9, 135.9, 122.5, 79.6, 61.3, 60.6, 56.6, 43.6, 39.9, 39.6, 28.5, 27.4, 26.6, 25.2, 19.4, 14.0; IR (neat, cm^{-1}): 2978, 2938, 2869, 1703, 1417, 1174, 863; ESI HRMS *m/z* (M+Na)$^{+}$ calcd 388.2094, obsd 388.2099.

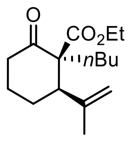

Ethyl 1-butyl-2-oxo-6-(prop-1-en-2-yl)cyclohexane-1-carboxylate (68). Yield = 45%; Isolated as a 6:1 mixture of diastereomers; Colorless oil; Only the major diastereoisomer has been showed; Electricity = 2.4 F mol^{-1}; ^{1}H NMR (400 MHz, $CDCl_3$) δ 4.91–4.88 (m, 1H), 4.77 (s, 1H), 4.16–4.07 (m, 2H), 2.78 (ddd, *J* = 15.5, 12.2, 6.6 Hz, 1H), 2.60 (dd, *J* = 12.2, 3.5 Hz, 1H), 2.51–2.42 (m, 1H), 2.28–2.16 (m, 1H), 2.10–1.95 (m, 2H), 1.83–1.62 (m, 6H), 1.37–1.23 (m, 6H), 1.11–0.99 (m, 1H), 0.89 (t, *J* = 7.1 Hz, 3H); ^{13}C NMR (101 MHz, $CDCl_3$) δ 208.6, 171.4, 144.7, 114.7, 63.9, 61.1, 50.7, 40.2, 32.0, 26.9, 26.8, 23.8, 23.5, 21.7, 14.1 (2C); IR (neat, cm^{-1}): 2926, 1752, 1732, 1435, 1258, 1083; ESI HRMS *m/z* (M+Na)$^{+}$ calcd 289.1774, obsd 289.1798.

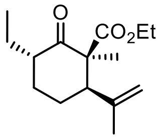

Ethyl 3-ethyl-1-methyl-2-oxo-6-(prop-1-en-2-yl)cyclohexane-1-carboxylate (69). Yield = 41%; Colorless oil; Electricity = 2.8 F mol^{-1}; ^{1}H NMR (400 MHz, $CDCl_3$) δ 4.89–4.86 (m, 1H), 4.78 (s, 1H), 4.19–4.02 (m, 2H), 2.90–2.80 (m, 1H), 2.50 (qd, *J* = 13.3, 3.9 Hz, 1H), 2.21–2.13 (m, 2H), 1.86–1.76 (m, 1H), 1.74 (s, 3H), 1.71–1.62 (m, 1H), 1.36–1.27 (m, 5H), 1.24 (t, *J* = 7.1 Hz, 3H), 0.89 (t, *J* = 7.4 Hz, 3H); ^{13}C NMR (101 MHz, $CDCl_3$) δ 208.7, 171.6, 144.7, 115.0, 61.2, 61.0, 58.0, 49.5, 32.6, 27.6, 22.9, 21.7, 19.5, 14.1, 11.8; IR (neat, cm^{-1}): 2937, 1716, 1455, 1379, 1209; ESI HRMS *m/z* (M+Na)$^{+}$ calcd 275.1618, obsd 275.1618.

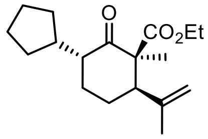

Ethyl 3-cyclopentyl-1-methyl-2-oxo-6-(prop-1-en-2-yl)cyclohexane-1-carboxylate (70). Yield = 67%; Colorless oil; Electricity = 2.5 F mol^{-1}; ^{1}H NMR (400 MHz, $CDCl_3$) δ 4.90–4.86 (m, 1H), 4.79 (s, 1H), 4.18–4.04 (m, 2H), 2.76 (ddd, *J* = 13.0, 8.5, 5.6 Hz, 1H), 2.47 (qd, *J* = 13.3, 4.1 Hz, 1H), 2.21–1.96 (m, 4H), 1.78–1.44 (m, 9H), 1.38–1.28 (m, 4H), 1.24 (t, *J* = 7.2 Hz, 3H), 1.14–1.03 (m, 1H), 1.01–0.91 (m, 1H); ^{13}C NMR (101 MHz, $CDCl_3$) δ 208.7, 171.7, 144.7, 115.1, 61.4, 61.1, 58.1, 54.2, 39.7, 32.0, 31.5, 30.2, 27.8, 25.6, 25.4, 21.9, 19.5, 14.1; IR (neat, cm^{-1}): 2949, 2868, 1716, 1451, 1379, 1210, 808; ESI HRMS *m/z* (M+Na)$^{+}$ calcd 315.1931, obsd 315.1930.

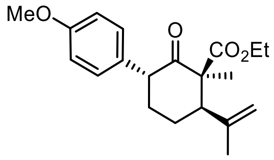

Ethyl 3-(4-methoxyphenyl)-1-methyl-2-oxo-6-(prop-1-en-2-yl)cyclohexane-1-carboxylate (71). Yield = 43%; Colorless oil; Electricity = 2.5 F mol^{-1}; ^{1}H NMR (400 MHz, $CDCl_3$) δ 7.07–7.02 (m, 2H), 6.89–6.84 (m, 2H), 4.94–4.89 (m, 1H), 4.82 (s, 1H), 4.35 (dd, *J* = 13.4,

5.8 Hz, 1H), 4.23–4.09 (m, 2H), 3.79 (s, 3H), 2.63 (qd, *J* = 13.2, 3.8 Hz, 1H), 2.35 (dd, *J* = 13.2, 3.4 Hz, 1H), 2.32–2.25 (m, 1H), 1.95 (qd, *J* = 13.1, 3.8 Hz, 1H), 1.81–1.73 (m, 4H), 1.35 (2s, 3H), 1.28 (2t, *J* = 7.1 Hz, 3H); ^{13}C NMR (101 MHz, $CDCl_3$) δ 206.6, 171.4, 158.6, 144.5, 131.3, 129.8, 115.1, 113.9, 61.4, 61.2, 57.8, 55.4, 53.9, 34.1, 27.7, 21.6, 19.6, 14.2; IR (neat, cm^{-1}): 2933, 1709, 1515, 1249, 1102, 831; ESI HRMS *m/z* (M+Na)$^+$ calcd 353.1723, obsd 353.1726.

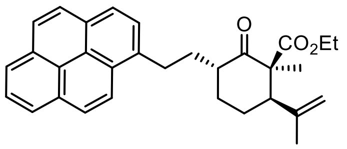

Ethyl 2-(1-cyclohexylvinyl)-1-methyl-6-oxocyclohexane-1-carboxylate (72). Yield = 66%; Yellow oil; Electricity = 2.7 F mol^{-1}; The stereochemistry of **72** was determined by 2D-NOESY analysis. ^{1}H NMR (400 MHz, $CDCl_3$) δ 8.46 (d, *J* = 9.2 Hz, 1H), 8.20–8.09 (m, 4H), 8.05–7.97 (m, 3H), 7.87 (d, *J* = 7.8 Hz, 1H), 4.96–4.90 (m, 1H), 4.83 (s, 1H), 4.13–3.99 (m, 2H), 3.49 (ddd, *J* = 13.4, 10.7, 5.4 Hz, 1H), 3.30–3.14 (m, 2H), 2.55 (qd, *J* = 13.2, 3.9 Hz, 1H), 2.42–2.31 (m, 1H), 2.27–2.18 (m, 2H), 1.78 (s, 3H), 1.76–1.64 (m, 2H), 1.52–1.41 (m, 4H), 1.15 (t, *J* = 7.2 Hz, 3H); ^{13}C NMR (101 MHz, $CDCl_3$) δ 208.7, 171.3, 144.5, 137.0, 131.5, 131.1, 129.9, 128.9, 127.6, 127.4, 127.3, 126.7, 125.9, 125.2, 125.1, 124.9 (2C), 124.8, 123.8, 115.1, 61.3, 61.2, 58.1, 47.7, 33.5, 32.7, 31.4, 27.6, 21.7, 19.5, 14.0; IR (neat, cm^{-1}): 3040, 2938, 1713, 1455, 1210, 844; ESI HRMS *m/z* (M+Na)$^+$ calcd 475.2244, obsd 475.2249.

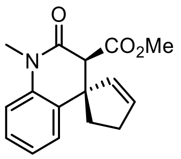

Methyl 1'-methyl-2'-oxo-2',3'-dihydro-1'*H*-spiro[cyclopentane-1,4'-quinolin]-2-ene-3'-carboxylate (73). Yield = 73%; Isolated as a 1:1 mixture of diastereomers; Pale yellow oil; Electricity = 2.5 F mol^{-1}; ^{1}H NMR (400 MHz, $CDCl_3$) δ 7.32–7.23 (m, 2H), 7.17–7.11 (m, 2H), 7.08–7.01 (m, 4H), 6.15 (dt, *J* = 5.6, 2.4 Hz, 1H), 6.06 (dt, *J* = 5.6, 2.4 Hz, 1H), 5.87 (dt, *J* = 5.6, 2.1 Hz, 1H), 5.59 (dt, *J* = 5.6, 2.1 Hz, 1H), 3.71 (s, 3H), 3.69 (s, 1H), 3.55 (s, 3H), 3.54 (s, 1H), 3.44 (s, 3H), 3.41 (s, 3H), 2.80 (ddd, *J* = 13.3, 9.2, 5.3 Hz, 1H), 2.63–2.35 (m, 4H), 2.16 (ddd, *J* = 13.1, 8.4, 4.9 Hz, 1H), 1.86–1.76 (m, 2H); ^{13}C NMR (101 MHz, $CDCl_3$) δ 168.9, 168.3, 166.1, 165.6, 139.0, 138.6, 135.6, 135.2, 134.3, 132.3, 131.9, 127.9, 127.8, 125.3, 124.5, 123.7, 123.6, 115.3, 115.1, 58.1, 56.6, 54.2, 53.6, 52.3, 52.1, 38.3, 32.7, 32.3, 31.2, 30.1, 30.0; IR (neat, cm^{-1}): 2950, 2852, 1738, 1678, 1600, 1367, 757; ESI HRMS *m/z* (M+Na)$^+$ calcd 294.1101, obsd 294.1099.

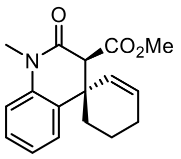

Methyl 1'-methyl-2'-oxo-2',3'-dihydro-1'*H*-spiro[cyclohexane-1,4'-quinolin]-2-ene-3'-carboxylate (74). Yield = 80%; Isolated as a 1:0.7 mixture of diastereomers; Pale yellow oil; Electricity = 2.5 F mol^{-1}; ^{1}H NMR (400 MHz, $CDCl_3$) δ 7.33–7.24 (m, 2.4H), 7.20 (dd, *J* = 7.6, 1.6 Hz, 1H), 7.10–7.02 (m, 3.4H), 6.21–6.11 (m, 1.7H), 5.63–5.57 (m, 1H), 5.45–5.40 (m, 0.7H), 3.75 (s, 2.1H), 3.62 (s, 0.7H), 3.59 (s, 1H), 3.57 (s, 3H), 3.43 (s, 3H), 3.42 (s, 2.1H), 2.63–2.54 (m, 0.7H), 2.13–2.06 (m, 3.4H), 1.79–1.28 (m, 6.1H); ^{13}C NMR (101 MHz, $CDCl_3$) δ 168.7, 168.2, 165.7, 165.0, 138.9, 138.6, 132.9, 132.7, 132.4, 131.1, 128.2, 128.0, 127.8, 127.6, 127.5 (2C), 123.3, 122.9, 115.5, 115.0, 59.0, 56.9, 52.3, 52.0, 41.6, 41.0, 34.6, 30.0, 29.9, 27.0, 25.0 (2C), 18.1, 18.0; IR (neat, cm^{-1}): 3020, 2933, 1739, 1678, 1599, 1459, 1368, 1157, 759; ESI HRMS *m/z* $(M+Na)^+$ calcd 308.1257, obsd 308.1257.

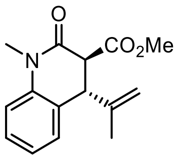

Methyl 1-methyl-2-oxo-4-(prop-1-en-2-yl)-1,2,3,4-tetrahydroquinoline-3-carboxylate (75). Yield = 31%; Yellow oil; Electricity = 2.5 F mol^{-1}; ^{1}H NMR (400 MHz, $CDCl_3$) δ 7.33–7.26 (m, 1H), 7.17–7.12 (m, 1H), 7.09–7.01 (m, 2H), 5.03–4.97 (m, 1H), 4.76 (s, 1H), 4.00 (d, *J* = 7.7 Hz, 1H), 3.76 (d, *J* = 7.7 Hz, 1H), 3.69 (s, 3H), 3.39 (s, 3H), 1.77 (s, 3H); ^{13}C NMR (101 MHz, $CDCl_3$) δ 169.5, 165.9, 142.1, 139.6, 128.4, 128.3, 125.6, 123.6, 115.6, 115.2, 52.8, 52.3, 46.7, 30.0, 19.9; IR (neat, cm^{-1}): 2942, 1745, 1678, 1601, 1370, 757; ESI HRMS *m/z* $(M+Na)^+$ calcd 282.1101, obsd 282.1101.

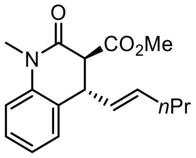

Methyl (*E*)-1-methyl-2-oxo-4-(pent-1-en-1-yl)-1,2,3,4-tetrahydroquinoline-3-carboxylate (76). Yield = 42%; Yellow oil; Electricity = 2.5 F mol^{-1}; ^{1}H NMR (500 MHz, $CDCl_3$) δ 7.31 (td, *J* = 7.5, 1.5 Hz, 1H), 7.23 (dt, *J* = 7.5, 1.3 Hz, 1H), 7.09 (td, *J* = 7.5, 1.1 Hz, 1H), 7.03 (dd, *J* = 8.2, 1.1 Hz, 1H), 5.59 (dtd, *J* = 15.3, 6.9, 0.9 Hz, 1H), 5.42 (ddt, *J* = 15.3, 7.8, 1.3 Hz, 1H), 3.98 (t, *J* = 8.4 Hz, 1H), 3.72 (s, 3H), 3.56 (d, *J* = 9.0 Hz, 1H), 3.41 (s, 3H), 2.07–2.01 (m, 2H), 1.42–1.37 (m, 2H), 0.90 (t, *J* = 7.4 Hz, 3H); ^{13}C NMR (101 MHz, $CDCl_3$) δ 169.5, 166.0, 139.3, 135.5, 128.2, 127.9, 127.6, 127.0, 123.6, 115.1, 54.2, 52.5, 42.6, 34.6, 30.0, 22.5, 13.7; IR (neat, cm^{-1}): 2952, 1742, 1674, 1603, 1360, 759; ESI HRMS *m/z* $(M+Na)^+$ calcd 310.1414, obsd 310.1417.

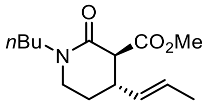

Methyl 1-butyl-2-oxo-4-((*E*)-prop-1-en-1-yl)piperidine-3-carboxylate (77). Yield = 42%; Isolated as a 7:1 mixture of diastereomers; Yellow oil; Electricity = 2.5 F mol^{-1}; Only the

major diastereoisomer has been showed; ^{1}H NMR (500 MHz, $CDCl_3$) δ 5.53 (dq, *J* = 15.2, 6.4 Hz, 1H), 5.30 (ddq, *J* = 15.2, 7.5, 1.6 Hz, 1H), 3.73 (s, 3H), 3.46–3.34 (m, 2H), 3.33–3.25 (m, 2H), 3.17 (d, *J* = 10.3 Hz, 1H), 2.85–2.77 (m, 1H), 1.98–1.92 (m, 1H), 1.68–1.62 (m, 4H), 1.56–1.49 (m, 2H), 1.32 (p, *J* = 7.3 Hz, 2H), 0.92 (t, *J* = 7.3 Hz, 3H); ^{13}C NMR (101 MHz, $CDCl_3$) δ 171.1, 165.4, 131.2, 126.9, 55.5, 52.3, 47.3, 46.6, 39.8, 29.1, 28.2, 20.2, 18.1, 14.0; IR (neat, cm^{-1}): 2929, 1743, 1716, 1644, 1455, 1156; ESI HRMS *m/z* $(M+Na)^+$ calcd 276.1570, obsd 276.1566.

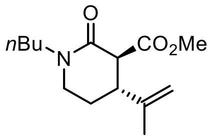

Methyl 1-butyl-2-oxo-4-(prop-1-en-2-yl)piperidine-3-carboxylate (78). Yield = 39%; Isolated as a 7:1 mixture of diastereomers; Yellow oil; Electricity = 2.5 F mol^{-1}; Only the major diastereoisomer has been showed; ^{1}H NMR (400 MHz, $CDCl_3$) δ 4.82 (s, 1H), 4.79 (s, 1H), 3.72 (s, 3H), 3.46–3.38 (m, 3H), 3.33–3.25 (m, 2H), 2.85 (td, *J* = 11.1, 3.1 Hz, 1H), 1.95 (dd, *J* = 13.5, 3.9 Hz, 1H), 1.79–1.71 (m, 4H), 1.57–1.48 (m, 2H), 1.36–1.28 (m, 2H), 0.92 (t, *J* = 7.3 Hz, 3H); ^{13}C NMR (101 MHz, $CDCl_3$) δ 171.1, 165.7, 144.6, 112.4, 53.9, 52.4, 47.3, 46.7, 43.9, 29.1, 27.1, 20.2, 19.6, 14.0; IR (neat, cm^{-1}): 2956, 1744, 1645, 1435, 1157; ESI HRMS *m/z* $(M+Na)^+$ calcd 276.1570, obsd 276.1564.

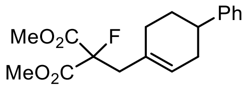

Dimethyl 2-fluoro-2-((1,2,3,6-tetrahydro-[1,1'-biphenyl]-4-yl)methyl)malonate (81). Yield = 72%; Colorless oil; Electricity = 4.6 F mol^{-1}; ^{1}H NMR (400 MHz, $CDCl_3$) δ 7.33–7.26 (m, 2H), 7.20 (d, *J* = 7.4 Hz, 3H), 5.73–5.59 (m, 1H), 3.84 (d, *J* = 4.8 Hz, 6H), 2.98–2.90 (m, 1H), 2.85 (s, 1H), 2.72 (ddt, *J* = 14.5, 7.8, 2.8 Hz, 1H), 2.37–2.06 (m, 4H), 1.92 (ddt, *J* = 12.8, 5.1, 2.6 Hz, 1H), 1.82–1.65 (m, 1H); ^{13}C NMR (101 MHz, $CDCl_3$) δ 166.7, 166.5, 146.9, 130.9, 128.529, 127.5, 127.0, 126.2, 95.6 (d, $J_{C\text{-}F}$ = 201.5 Hz), 53.5, 53.4, 42.2 (d, $J_{C\text{-}F}$ = 20.4 Hz), 39.7, 33.8, 30.1, 30.1; ^{19}F NMR (376 MHz, $CDCl_3$) δ -163.8; IR (neat, cm^{-1}): 2922, 1755, 1436, 1266, 701.

Synthesis and Characterization of Substrates

General Procedure 1 (GP 1):

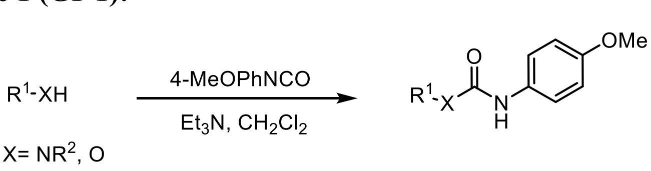

To a solution of the alcohol or amine (1 equiv) in CH_2Cl_2 (0.1 M) was added 4-methoxyphenyl isocyanate (1.2 equiv) at rt, followed by Et_3N (2.0 equiv). The resulting reaction mixture was stirred at rt until complete consumption of the starting material (monitored by TLC). The

solvent was removed under reduced pressure. The residue was chromatographed through silica gel eluting with EtOAc/hexanes to give the product.

General Procedure 2 (GP 2):

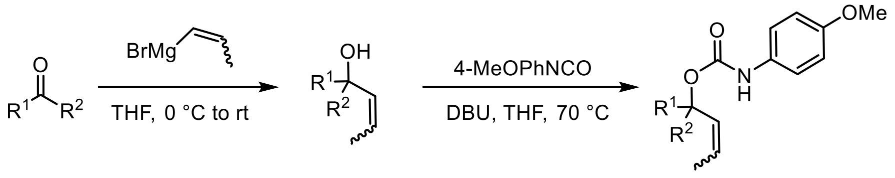

Step 1: To a solution of prop-1-en-1-ylmagnesium bromide in THF (0.5 M, 1.2 equiv) under argon atmosphere at 0 °C was added the corresponding ketone (1.0 equiv) dropwise over 10 min. Upon complete addition, the reaction mixture was stirred at rt. The completion of the reaction was monitored by TLC. Saturated aqueous NH_4Cl was added to quench the reaction. The resulting mixture was extracted with EtOAc. The combined organic solution was washed with brine, dried over anhydrous $MgSO_4$, filtered, and concentrated under reduced pressure to afford the corresponding alcohol. The crude product was used in the following step without purification.

Step 2: To a solution of the alcohol (1.0 equiv) in THF (0.1 M) was added 4-methoxyphenyl isocyanate (2.0 equiv) at rt, followed by DBU (3.0 equiv). The resulting reaction mixture was stirred at 70 °C until complete consumption of the starting material (monitored by TLC). The solvent was removed under reduced pressure. The residue was chromatographed through silica gel eluting with EtOAc/hexanes to give the product.

General Procedure 3 (GP 3):

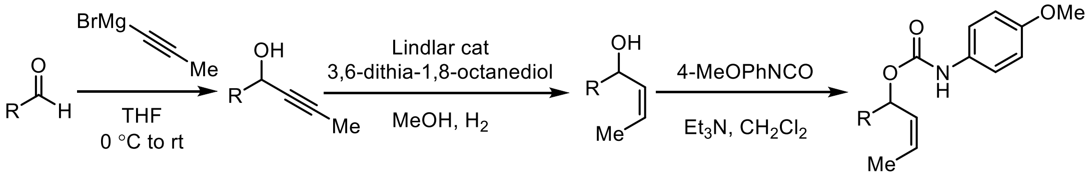

Step 1: To a solution of 1-propynylmagnesium bromide in THF (0.5 M, 1.2 equiv) under argon atmosphere at 0 °C was added the corresponding aldehyde (1.0 equiv) dropwise over 10 min. Upon complete addition, the reaction mixture was stirred at rt. The completion of the reaction was monitored by TLC. Saturated aqueous NH_4Cl was added to quench the reaction. The resulting mixture was extracted with EtOAc. The combined organic solution was washed with brine, dried over anhydrous $MgSO_4$, filtered, and concentrated under reduced pressure to afford the corresponding alcohol. The crude product was used in the following step without purification.

Step 2: The corresponding alcohol was dissolved in MeOH (0.6 M) under argon atmosphere. 3,6-Dithia-1,8-octanediol (0.017 equiv) and Lindlar catalyst (5% Pd on $CaCO_3$ poisoned with

Pb, 0.05 equiv) were added. The reaction mixture was then stirred under hydrogen atmosphere (balloon). The completion of the reaction was monitored by TLC. The reaction mixture was filtered through a pad of celite and concentrated under reduced pressure. The residue was chromatographed through silica gel eluting with EtOAc/hexanes to give the product.

Step 3: The corresponding carbamate was prepared starting from alcohol by following the GP 1.

General Procedure 4 (GP 4):

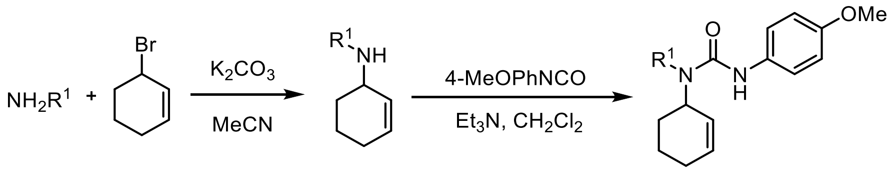

Step 1: To a solution of amine (2.7 equiv) in CH_3CN (1 M), was added K_2CO_3 (1.0 equiv), followed by 3-bromocyclohexene (1.0 equiv). The resulting reaction mixture was stirred for 12 h at rt. Solvent was removed under reduced pressure. The residue was chromatographed through silica gel eluting with EtOAc/hexanes to afford the product.

Step 2: The corresponding urea was prepared starting from amine by following the GP 1.

General Procedure 5 (GP 5):

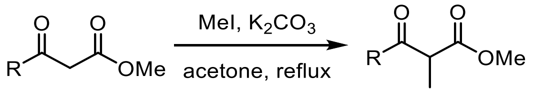

To a mixture of the corresponding *β*-keto ester (1.0 equiv) and K_2CO_3 (0.95 equiv) in acetone (0.2 M) was added iodomethane (5.0 equiv) at rt. The mixture was stirred under reflux for 24 h. H_2O was added to quench the reaction. The mixture was extracted with EtOAc. The combined organic layer was dried over anhydrous Na_2SO_4, filtered, and concentrated under reduced pressure. The residue was purified by flash column chromatography on silica gel eluting with EtOAc/hexanes to give the product.

General Procedure 6 (GP 6):

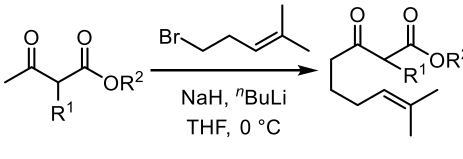

To a suspension of NaH (1.3 equiv) in THF (0.3 M) was added the corresponding *β*-keto ester (1.0 equiv) dropwise at 0 °C and stirred at the same temperature for 15 min. *n*BuLi (1.3 equiv) was added dropwise via a syringe. The resulting mixture was stirred for 30 min. The alkenyl bromide was then added slowly. The mixture was warmed to rt and stirred until complete

consumption of the β-keto ester (monitored by TLC). The mixture was cooled to 0 °C and treated with a saturated NH_4Cl solution cautiously. The mixture was extracted with Et_2O. The combined organic solution was washed with brine, dried over anhydrous Na_2SO_4, filtered, and concentrated under reduced pressure. The residue was purified by flash column chromatography on silica gel eluting with EtOAc/hexanes to give the product.

General Procedure 7 (GP 7):

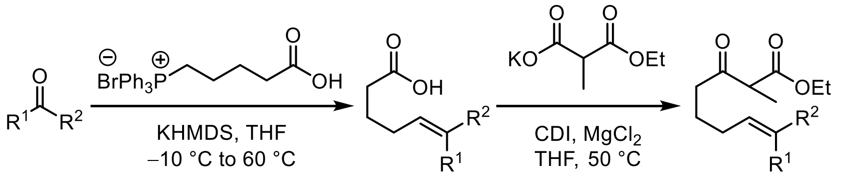

Step 1: T a mixture of 4-(carboxybutyl)triphenylphosphonium bromide (1.1 equiv) in THF (0.5 M) at −10 °C was added KHMDS (2.3 equiv) was added dropwise via a syringe. The resultant orange mixture was stirred at ambient temperature for 1 h. The corresponding ketone (1.0 equiv) was added slowly. The mixture was stirred at 60 °C for 10 h. Cool the mixture to 0 °C and H_2O was added slowly to quench the reaction. The mixture was washed with Et_2O. The aqueous solution was acidified with 2 N HCl to pH 1 and extracted with Et_2O. The combined organic layer was washed with brine, dried over anhydrous $MgSO_4$ and concentrated under reduced pressure to give the crude carboxylic acid, which was used for next step without further purification.

Step 2: The corresponding carboxylic acid (1.0 equiv) was dissolved in anhydrous THF (0.4 M), followed by CDI (1.5 equiv) in one portion. After stirred for 2 h at 50 °C, the mixture was added $MgCl_2$ (2.0 equiv) and potassium 3-ethoxy-2-methyl-3-oxo-propanoate (2.0 equiv). The mixture was stirred at 50 °C for 12 h. Cool the mixture to room temperature and a saturated NH_4Cl solution was added slowly. The mixture was extracted with Et_2O. The combined organic solution was washed with brine, dried over anhydrous Na_2SO_4, filtered, and concentrated under reduced pressure. The residue was purified by flash column chromatography on silica gel with EtOAc/hexanes to give the product.

General Procedure 8 (GP 8):

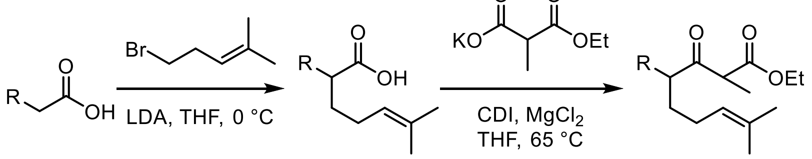

Diisopropyl amine (2.2 equiv) was dissolved in THF (1 M). The solution was cooled to 0 °C. *n*BuLi (2.1 equiv) was added dropwise. The resulting solution was stirred for 30 min at the same temperature and then cooled to −78 °C. The carboxylic acid (1.0 equiv) was added

dropwise. The mixture was warmed to rt and stirred overnight. The reaction mixture was cooled to 0 °C and quenched with H_2O. The mixture was extracted with Et_2O. The aqueous phase was acidified to pH 1 and extracted with CH_2Cl_2. The combined organic layer was dried over anhydrous $MgSO_4$, filtered and concentrated under reduced pressure to afford the crude alkenyl carboxylic acid, which was used for next step without further purification. GP 7, step 2 was followed to prepare the ketoester but under a higher temperature of 65 °C.

General Procedure 9 (GP 9):

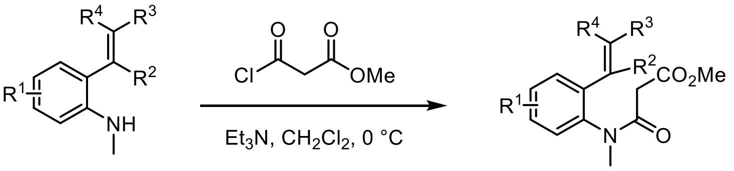

A solution of aniline (1.0 equiv) and Et_3N (1.5 equiv) in anhydrous CH_2Cl_2 (0.3 M) was cooled to 0 °C. Methyl malonyl chloride (1.2 equiv) was added dropwise. The mixture was then allowed to warm to rt and stirred until complete consumption of the corresponding aniline (monitored by TLC). The solvent was removed under reduced pressure. EtOAc was added and the mixture was filtered. The filtrate was concentrated under reduce pressure. The residue was purified by flash column chromatography on silica gel with EtOAc/hexanes to give the product.

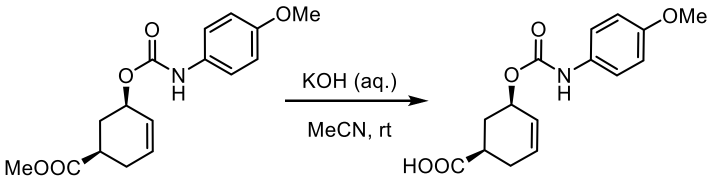

S1 **S2**

5-(((4-Methoxyphenyl)carbamoyl)oxy)cyclohex-3-ene-1-carboxylic acid (S2). To a solution of **S1**[12] (0.31 g, 1.0 mmol, 1.0 equiv) in MeCN (5 mL) was added KOH (0.2 M in H_2O, 5 mL, 1.0 equiv). The resulting reaction mixture was stirred at rt for 6 h and then extracted with EtOAc. The aqueous solution was acidified with HCl (1 M) to pH 4. The solid was filtered, washed with H_2O and dried to give the title compound in 81% yield (0.24 g). White solid; ^{1}H NMR (600 MHz, acetone-d_6) δ 10.81 (brs, 1H), 8.48 (brs, 1H), 7.59–7.39 (m, 2H), 6.94–6.85 (m, 2H), 5.91 (ddt, J = 9.7, 4.7, 2.3 Hz, 1H), 5.79–5.70 (m, 1H), 5.47–5.34 (m, 1H), 3.80 (s, 3H), 2.83 (dddd, J = 13.1, 10.5, 5.6, 2.9 Hz, 1H), 2.54–2.46 (m, 1H), 2.42–2.34 (m, 1H), 2.33–2.22 (m, 1H), 1.68 (td, J = 12.5, 9.9 Hz, 1H); ^{13}C NMR (151 MHz, acetone-d_6) δ 176.2, 157.2, 155.0, 134.0, 130.3, 129.3, 121.5, 115.5, 71.3, 56.4, 39.2, 32.8, 28.9; IR (neat, cm^{-1}): 3306, 2921, 1694, 1514, 1244, 1030, 827; ESI HRMS m/z $(M+Na)^+$ calcd 314.0999, obsd 314.1005.

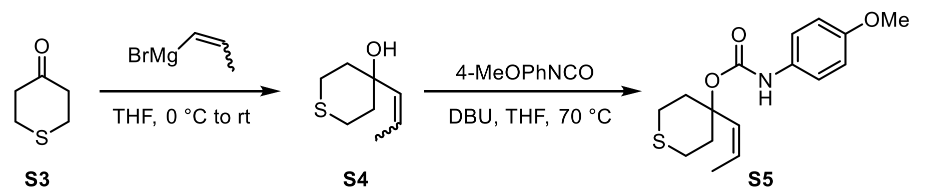

S3 **S4** **S5**

(*Z*)-4-(Prop-1-en-1-yl)tetrahydro-2*H*-thiopyran-4-yl (4-methoxyphenyl)carbamate (S5). The title compound (0.15 g, 51% yield in two steps) was obtained as a *Z*-isomer and white solid starting from **S3** (0.11 g, 0.96 mmol) by following the GP 2. ^{1}H NMR (600 MHz, $CDCl_3$) δ 7.39–7.21 (m, 2H), 6.89–6.81 (m, 2H), 6.70 (brs, 1H), 5.61–5.49 (m, 2H), 3.77 (s, 3H), 3.01–2.86 (m, 2H), 2.68–2.57 (m, 2H), 2.53–2.41 (m, 2H), 2.07–1.96 (m, 2H), 1.85–1.72 (m, 3H); ^{13}C NMR (151 MHz, $CDCl_3$) δ 155.9, 152.1, 133.3, 131.2, 126.4, 120.5, 114.3, 79.8, 55.6, 37.0, 24.1, 14.3; IR (neat, cm^{-1}): 3326, 2921, 1700, 1515, 1226, 1196, 1031, 829; ESI HRMS *m/z* (M+Na)$^+$ calcd 330.1134, obsd 330.1147.

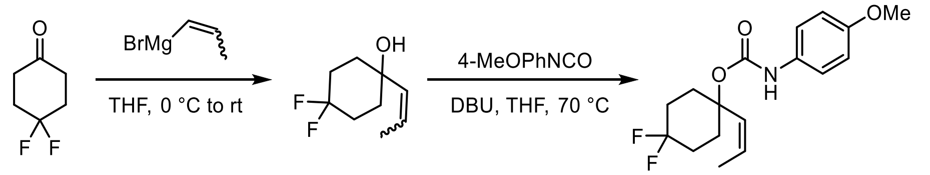

S6 **S7** **S8**

(*Z*)-4,4-Difluoro-1-(prop-1-en-1-yl)cyclohexyl (4-methoxyphenyl)carbamate (S8). The title compound (0.17 g, 49% yield in two steps) was obtained as a *Z*-isomer and white solid starting from **S6** (0.13 g, 1.0 mmol) by following the GP 2. ^{1}H NMR (600 MHz, $CDCl_3$) δ 7.40–7.22 (m, 2H), 6.91–6.76 (m, 2H), 6.63 (brs, 1H), 5.68–5.50 (m, 2H), 3.77 (s, 3H), 2.56–2.44 (m, 2H), 2.14–1.94 (m, 4H), 1.94–1.85 (m, 2H), 1.84–1.75 (m, 3H); ^{13}C NMR (151 MHz, $CDCl_3$) δ 156.0, 152.2, 132.0, 131.1, 127.3, 122.8 (t, $J_{C\text{-}F}$ = 241.6 Hz), 120.6, 114.4, 79.4, 55.6, 32.5 (d, $J_{C\text{-}F}$ = 8.3 Hz), 30.0 (t, $J_{C\text{-}F}$ = 24.6 Hz), 14.3; ^{19}F NMR (565 MHz, $CDCl_3$) δ −93.6 (d, J = 235.0 Hz), −102.0 (d, J = 236.0 Hz); IR (neat, cm^{-1}): 3328, 2943, 1701, 1515, 1217, 1032, 975, 829; ESI HRMS *m/z* (M+Na)$^+$ calcd 348.1382, obsd 348.1388.

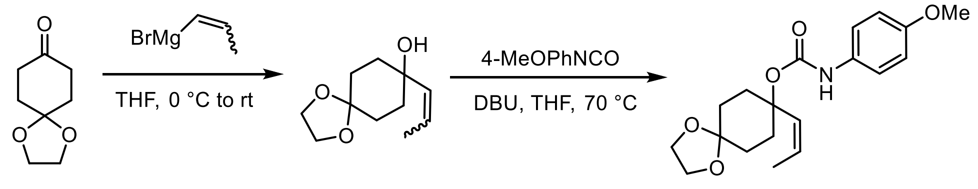

S9 **S10** **S11**

(*Z*)-8-(Prop-1-en-1-yl)-1,4-dioxaspiro[4.5]decan-8-yl (4-methoxyphenyl)carbamate (S11). The title compound (0.23 g, 61% yield in two steps) was obtained as a *Z*-isomer and white solid starting from **S9** (0.16 g, 1.0 mmol) by following the GP 2. ^{1}H NMR (600 MHz, $CDCl_3$) δ 7.33–7.29 (m, 2H), 6.86–6.80 (m, 2H), 6.71 (brs, 1H), 5.65–5.60 (m, 1H), 5.60–5.52 (m, 1H), 4.00–3.92 (m, 4H), 3.77 (s, 3H), 2.49–2.35 (m, 2H), 2.02–1.91 (m, 2H), 1.89–1.82 (m, 2H), 1.82–1.75 (m, 3H), 1.70–1.61 (m, 2H); ^{13}C NMR (151 MHz, $CDCl_3$) δ 155.8, 152.5, 132.9, 131.4, 126.4, 120.5, 114.2, 108.0, 80.3, 64.4, 64.3, 55.6, 33.6, 30.8, 14.2; IR (neat, cm^{-1}): 3322, 2955,

1702, 1515, 1214, 1033, 829, 725; ESI HRMS *m/z* (M+Na)+ calcd 370.1625, obsd 370.1631.

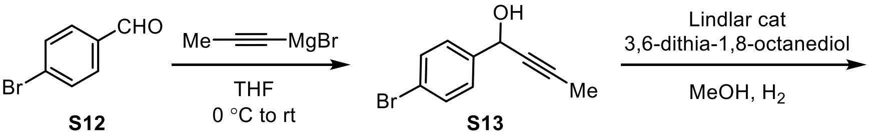

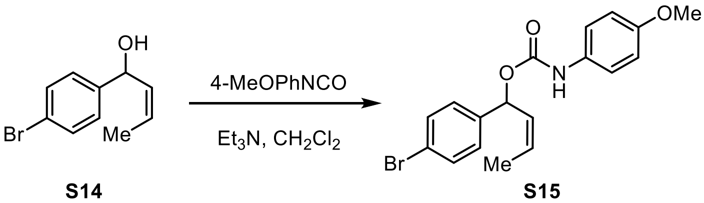

(*Z*)-1-(4-Bromophenyl)but-2-en-1-yl (4-methoxyphenyl)carbamate (S15). The title compound (1.3 g, 89% yield in three steps) was prepared as a white solid starting from **S12** (0.74 g, 4.0 mmol) by following the GP 3. ^{1}H NMR (500 MHz, $CDCl_3$) δ 7.51–7.45 (m, 2H), 7.30–7.25 (m, 4H), 6.88–6.79 (m, 2H), 6.61–6.45 (m, 2H), 5.75 (dqd, *J* = 10.8, 6.9, 1.1 Hz, 1H), 5.63 (ddq, *J* = 10.7, 8.9, 1.7 Hz, 1H), 3.77 (s, 3H), 1.82 (dd, *J* = 6.9, 1.7 Hz, 3H); ^{13}C NMR (126 MHz, $CDCl_3$) δ 156.2, 153.2, 139.5, 131.9, 130.9, 128.9, 128.6, 128.4, 122.0, 120.8, 114.4, 71.7, 55.6, 13.7; IR (neat, cm^{-1}): 3416, 1701, 1514, 1220, 1033, 827, 523; ESI HRMS *m/z* (M+Na)+ calcd 398.0362, obsd 398.0373.

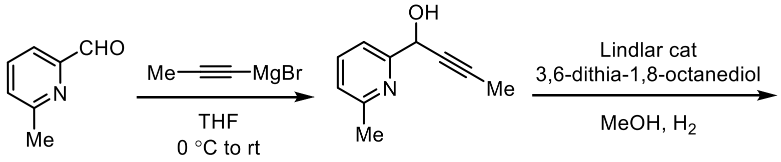

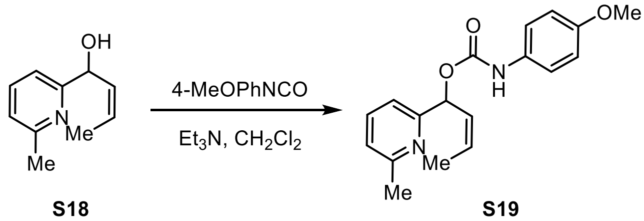

(*Z*)-1-(6-Methylpyridin-2-yl)but-2-en-1-yl (4-methoxyphenyl)carbamate (S19). The title compound (0.79 g, 59% yield in three steps) was prepared as a white solid starting from **S16** (0.48 g, 4.0 mmol) by following the GP 3. ^{1}H NMR (600 MHz, $CDCl_3$) δ 7.59–7.46 (m, 1H), 7.31–7.24 (m, 2H), 7.15 (d, *J* = 7.7 Hz, 1H), 7.05 (d, *J* = 7.7 Hz, 1H), 6.96–6.84 (m, 1H), 6.84–6.76 (m, 2H), 6.56 (d, *J* = 7.5 Hz, 1H), 5.92–5.70 (m, 2H), 3.75 (s, 3H), 2.55 (s, 3H), 1.87 (s, 3H); ^{13}C NMR (151 MHz, $CDCl_3$) δ 158.7, 158.5, 156.0, 137.1, 131.2, 129.6, 128.1 (2C), 122.6, 120.6, 118.6, 114.3, 73.1, 55.6, 24.7, 13.9; IR (neat, cm^{-1}): 3319, 1719, 1514, 1220, 1032, 829, 779; ESI HRMS *m/z* (M+Na)+ calcd 335.1366, obsd 335.1373.

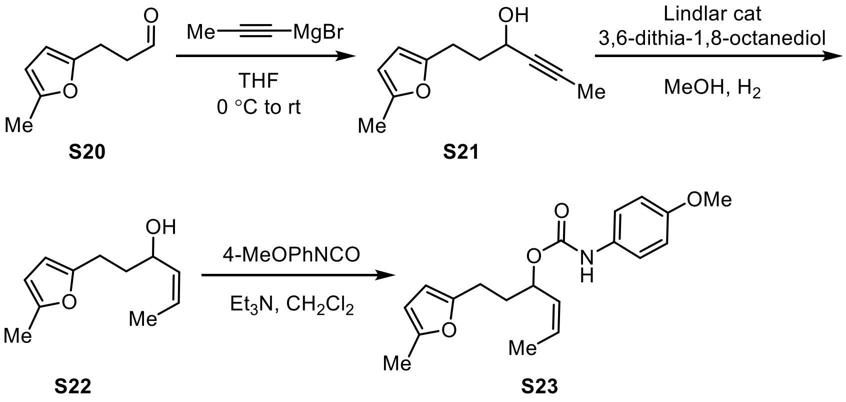

(*Z*)-1-(5-Methylfuran-2-yl)hex-4-en-3-yl (4-methoxyphenyl)carbamate (S23). The title compound (1.2 g, 89% yield in three steps) was prepared as a colorless oil starting from **S20** (0.55 g, 4.0 mmol) by following the GP 3. ^{1}H NMR (500 MHz, $CDCl_3$) δ 7.32–7.18 (m, 2H), 6.88–6.76 (m, 2H), 6.59 (brs, 1H), 5.89–5.85 (m, 1H), 5.85–5.79 (m, 1H), 5.72–5.62 (m, 1H), 5.62–5.54 (m, 1H), 5.43–5.33 (m, 1H), 3.75 (s, 3H), 2.63 (t, *J* = 7.1 Hz, 2H), 2.23 (s, 3H), 2.11–1.99 (m, 1H), 1.93–1.80 (m, 1H), 1.73 (d, *J* = 6.9 Hz, 3H); ^{13}C NMR (151 MHz, $CDCl_3$) δ 155.9, 153.6, 153.4, 150.5, 131.2, 129.0, 128.8, 120.6, 114.3, 106.0, 105.7, 70.5, 55.6, 33.4, 24.0, 13.6 (2C); IR (neat, cm^{-1}): 3327, 2922, 1702, 1515, 1219, 1033, 829, 784; ESI HRMS *m/z* (M+Na)$^+$ calcd 352.1519, obsd 352.1526.

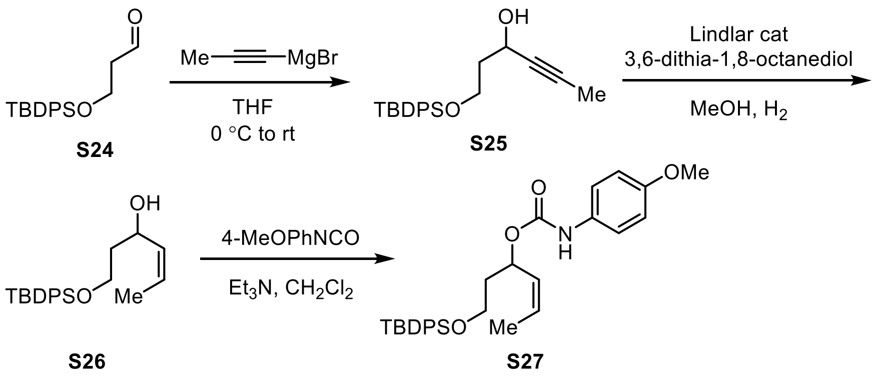

(*Z*)-1-((*tert*-Butyldiphenylsilyl)oxy)hex-4-en-3-yl (4-methoxyphenyl)carbamate (S27). The title compound (3.06 g, 61% yield in three steps) was prepared as a colorless oil starting from **S24**[13] (3.13 g, 10.0 mmol) by following the GP 3. ^{1}H NMR (500 MHz, $CDCl_3$) δ 7.69–7.65 (m, 4H), 7.40–7.30 (m, 6H), 7.29–7.20 (m, 2H), 6.88–6.77 (m, 2H), 6.47 (brs, 1H), 5.88–5.77 (m, 1H), 5.62 (dq, *J* = 10.8, 6.9 Hz, 1H), 5.42–5.29 (m, 1H), 3.77–3.68 (m, 5H), 1.95 (ddt, *J* = 13.6, 7.9, 5.7 Hz, 1H), 1.83–1.75 (m, 4H), 1.06 (s, 9H); ^{13}C NMR (126 MHz, $CDCl_3$) δ 155.9, 153.5, 135.7 (2C), 134.9, 133.9, 133.7, 131.3, 129.7, 129.4, 128.3, 127.8 (2C), 120.6, 114.3, 68.5, 60.0, 55.6, 37.8, 27.0, 19.3, 13.6; IR (neat, cm^{-1}): 3334, 2931, 1729, 1515, 1431, 1219, 1032, 826, 505; ESI HRMS *m/z* (M+Na)$^+$ calcd 526.2384, obsd 526.2399.

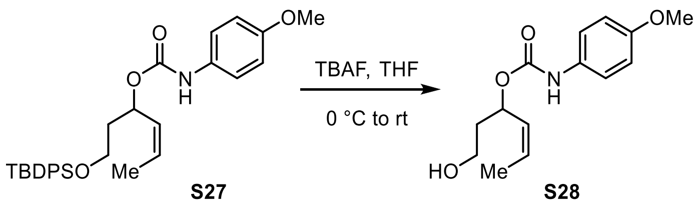

(*Z*)-1-Hydroxyhex-4-en-3-yl (4-methoxyphenyl)carbamate (S28). To a solution of **S27** (2.5 g, 5.0 mmol, 1.0 equiv) in THF (30 mL) at 0 °C was added dropwise a solution of TBAF·3H_2O (3.2 g, 10 mmol, 2.0 equiv) in THF (10 mL). The reaction mixture was stirred for 8 h under rt, diluted with EtOAc, and quenched with saturated $NaHCO_3$. The layers were separated and the aqueous layer was extracted twice with EtOAc. The combined organic solution was washed with brine, dried over anhydrous $MgSO_4$, filtered and concentrated under reduced pressure. The residue was chromatographed through silica gel eluting with EtOAc/hexanes to afford the title compound as a colorless oil in 75% yield (0.99 g). ^{1}H NMR (500 MHz, $CDCl_3$) δ 7.32–7.21 (m, 2H), 7.07 (brs, 1H), 6.84–6.78 (m, 2H), 5.75 (td, *J* = 8.6, 5.0 Hz, 1H), 5.63 (dqd, *J* = 10.7, 6.9, 1.1 Hz, 1H), 5.42 (ddq, *J* = 10.7, 8.9, 1.8 Hz, 1H), 3.75 (s, 3H), 3.71–3.63 (m, 2H), 3.06 (brs, 1H), 1.93–1.77 (m, 2H), 1.73 (dd, *J* = 6.9, 1.8 Hz, 3H); ^{13}C NMR (126 MHz, $CDCl_3$) δ 156.0, 154.3, 131.1, 129.0, 128.4, 120.8, 114.3, 68.4, 58.6, 55.5, 38.1, 13.5; IR (neat, cm^{-1}): 3317, 1701, 1515, 1229, 1032, 829; ESI HRMS *m/z* $(M+Na)^+$ calcd 288.1206, obsd 288.1213.

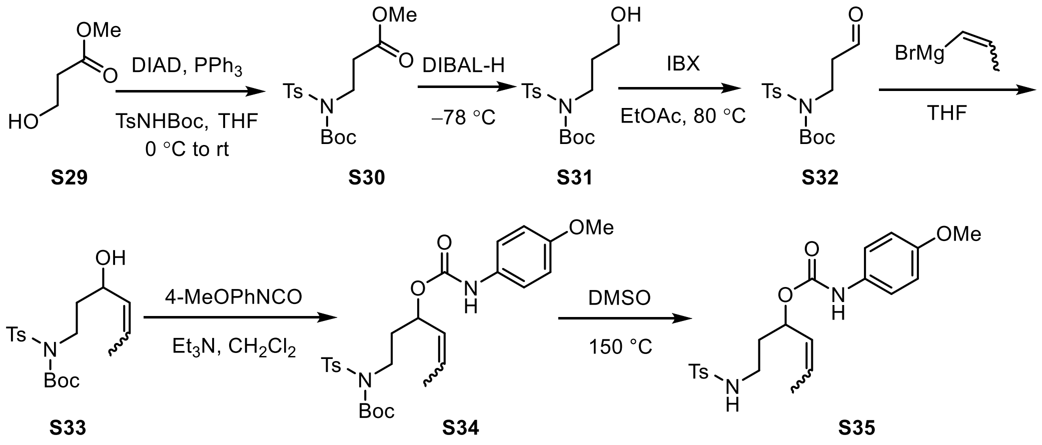

Methyl 3-((*N*-(*tert*-butoxycarbonyl)-4-methylphenyl)sulfonamido)propanoate (S30). Diisopropyl azodicarboxylate (DIAD, 0.76 mL, 4.3 mmol, 1.3 equiv) was added dropwise to a solution of **S29** (5.0 g, 48 mmol, 1.0 equiv), *N*-(*tert*-butoxycarbonyl)-*p*-toluenesulfonamide (14.3 g, 52.8 mmol, 1.10 equiv) and PPh_3 (13.8 g, 52.8 mmol, 1.10 equiv) in THF (100 mL) at 0 °C. The reaction mixture was stirred at rt for 12 h and then concentrated under reduced pressure. The residue was chromatographed through silica gel eluting with EtOAc/hexanes to give the title compound as a white solid (7.2 g, 42%). The product was directly used without further purification.

***tert*-Butyl (3-hydroxypropyl)(tosyl)carbamate (S31).** A solution of **S30** (3.6 g, 10 mmol, 1.0 equiv) in THF (30 mL) was cooled to –78 °C, and DIBAL-H (1.0 N solution in hexane; 30 mL, 30 mmol, 3.0 equiv) was added dropwise. The solution was stirred for 45 min at –78 °C. Then it

was slowly warmed to 0 °C, quenched sequentially by H_2O (1.2 mL), NaOH (15%, 1.2 mL) and H_2O (3 mL). Then it was warmed to rt and stirred for 15 min, $MgSO_4$ was added then stirred for another 15 min, filtered and concentrated under reduced pressure to give the product in quantitative yield. The product was directly used without further purification.

***tert*-Butyl (3-oxopropyl)(tosyl)carbamate (S32).** To a solution of **S31** (3.3 g, 10 mmol, 1.0 equiv) in EtOAc (50 mL) was added IBX (8.4 g, 30 mmol, 3.0 equiv). The mixture was warmed to 80 °C and stirred for 5 h. The reaction mixture was cooled to rt, filtered and concentrated under reduced pressure to give the title compound in quantitative yield. The product was directly used without further purification.

***tert*-Butyl (3-(((4-methoxyphenyl)carbamoyl)oxy)hex-4-en-1-yl)(tosyl)carbamate (S34).** The title compound (1.8 g, 35% yield in two steps) was prepared as a light-yellow oil starting from **S32** (3.3 g, 10 mmol) by following the GP 2 and GP 1. *Z/E* = 8:1; ^{1}H NMR (500 MHz, $CDCl_3$) δ 9.13 (brs, 1H), 7.76–7.70 (m, 2H), 7.37–7.29 (m, 4H), 6.88–6.81 (m, 2H), 5.79 (ddt, *J* = 15.6, 7.0, 6.0 Hz, 0.2H), 5.65 (dq, *J* = 10.3, 7.0 Hz, 0.8H), 5.50–4.94 (m, 2H), 3.81–3.64 (m, 5H), 2.41 (s, 3H), 2.06 (dtt, *J* = 13.8, 6.6, 5.3 Hz, 1H), 2.00–1.92 (m, 1H), 1.72 (dd, *J* = 7.0, 1.7 Hz, 2.7H), 1.69 (dd, *J* = 6.5, 1.7 Hz, 0.3H), 1.47 (s, 9H); Only the major isomer was shown; ^{13}C NMR (126 MHz, $CDCl_3$) δ 156.7, 152.9, 150.1, 145.1, 135.8, 130.4, 130.2, 128.8, 128.3, 127.0, 122.2, 114.3, 81.8, 70.8, 55.5, 42.4, 34.4, 27.8, 21.6, 13.5; IR (neat, cm^{-1}): 3358, 2978, 1739, 1514, 1354, 1279, 1156, 670; ESI HRMS *m/z* $(M+Na)^+$ calcd 541.1979, obsd 541.1987.

1-((4-Methylphenyl)sulfonamido)hex-4-en-3-yl (4-methoxyphenyl)carbamate (S35). A solution of **S34** (1.8 g, 3.5 mmol, 1.0 equiv) in anhydrous DMSO (40 mL) was heated at 150 °C for 1 h. The reaction mixture was cooled to rt and then poured into Et_2O (120 mL)/H_2O (40 mL). The layers were separated, and the aqueous layer was extracted twice with Et_2O. The combined organic solution was washed with H_2O and brine, dried over $MgSO_4$, filtered, and concentrated under reduced pressure. The crude product was purified by silica gel to give the title compound as a light-yellow oil in 25% yield (0.35 g). *Z/E* = 8:1; ^{1}H NMR (500 MHz, $CDCl_3$) δ 7.75–7.69 (m, 2H), 7.28–7.17 (m, 4H), 6.98 (brs, 1H), 6.83–6.78 (m, 2H), 5.73–5.50 (m, 3H), 5.40–5.24 (m, 1H), 3.76 (s, 3H), 3.11–2.99 (m, 1H), 2.99–2.87 (m, 1H), 2.35 (s, 3H), 1.87–1.70 (m, 2H), 1.69–1.59 (m, 3H); Only the major isomer was shown. ^{13}C NMR (126 MHz, $CDCl_3$) δ 156.0, 153.7, 143.4, 136.9, 131.0, 129.8, 128.8, 128.4, 127.1, 120.7, 114.2, 68.7, 55.6, 39.6, 34.8, 21.5, 13.5; IR (neat, cm^{-1}): 3293, 2933, 1701, 1514, 1224, 1159, 829; ESI HRMS *m/z* $(M+Na)^+$ calcd 441.1455, obsd 441.1466.

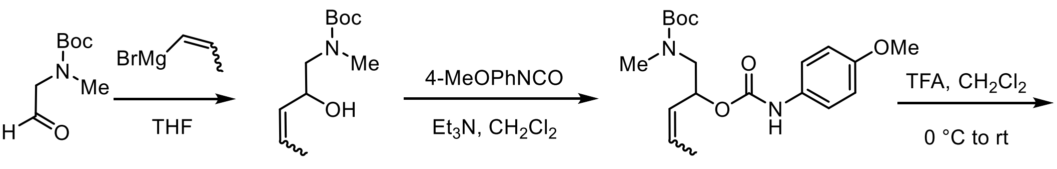

S36 **S37** **S38**

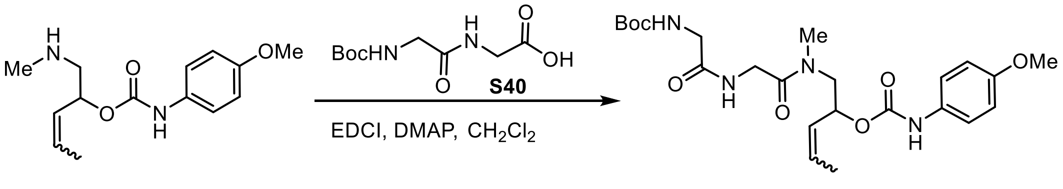

S39 **S41**

***tert*-Butyl (2-(((4-methoxyphenyl)carbamoyl)oxy)pent-3-en-1-yl)(methyl)carbamate (S38).** The title compound (1.9 g, 77% yield in two steps) was prepared as a light-yellow oil starting from **S36** (1.2 g, 7.0 mmol) by following the GP 2 and the GP 1. Isolated as 3:2 mixture of rotary isomers with 6:1 *Z*/*E*. ^{1}H NMR (500 MHz, $CDCl_3$) δ 7.36–7.24 (m, 2H), 7.04–6.94 (m, 0.4H), 6.90 (brs, 0.6H), 6.85–6.79 (m, 2H), 5.86–5.75 (m, 1H), 5.74–5.65 (m, 1H), 5.43–5.28 (m, 1H), 3.76 (s, 3H), 3.61–3.51 (m, 0.4H), 3.48–3.38 (m, 0.6H), 3.35–3.19 (m, 1H), 2.95–2.88 (m, 3H), 1.77 (dd, *J* = 7.0, 1.8 Hz, 2.6H), 1.68 (dd, *J* = 7.0, 1.8 Hz, 0.4H), 1.51–1.39 (m, 9H); ^{13}C NMR (126 MHz, $CDCl_3$) δ 156.0, 155.6, 153.3, 131.2, 130.4, 129.8, 127.0, 120.7, 114.2, 79.9, 79.6, 69.6, 68.5, 55.5, 52.4, 51.8, 36.1, 35.4, 28.5, 13.6, 13.4; IR (neat, cm^{-1}): 3310, 2929, 1701, 1515, 1393, 1219, 1156, 1034, 829; ESI HRMS *m/z* $(M+Na)^+$ calcd 387.1890, obsd 387.1901.

1-(Methylamino)pent-3-en-2-yl (4-methoxyphenyl)carbamate (S39). S38 (1.1 g, 3.0 mmol) was dissolved in CH_2Cl_2 (3.5 mL) and treated with trifluoroacetic acid (3.5 mL) at rt. The mixture was stirred at rt for 1 h, concentrated under reduced pressure, the residues diluted with CH_2Cl_2, and basified by saturated $NaHCO_3$. The layers were separated, and the aqueous layer was extracted twice with CH_2Cl_2. The combined organic solution was washed with brine, dried over anhydrous $MgSO_4$, and concentrated under reduced pressure. The product was directly used without further purification.

2,2,11-Trimethyl-4,7,10-trioxo-3-oxa-5,8,11-triazahexadec-14-en-13-yl (4-methoxyphenyl) carbamate (S41). To a solution of **S40** (0.30 g, 1.3 mmol, 1.0 equiv) in CH_2Cl_2 (20 mL) was added EDCI (0.41 g, 2.2 mmol, 1.5 equiv), followed by **S39** (0.57 g, 2.2 mmol, 1.5 equiv) and DMAP (20 mg, 0.14 mmol, 0.10 equiv). The resulting reaction mixture was stirred at rt for 8 h, diluted with EtOAc, and quenched with HCl (1 N, 15 mL). The layers were separated, and the aqueous layer was extracted twice with EtOAc. The combined organic solution was washed with brine, dried over anhydrous $MgSO_4$, and concentrated under reduced pressure. The residue was chromatographed through silica gel eluting with CH_2Cl_2/MeOH to afford **S41** in 89% yield (0.55 g, white foamy solid), as a 3:2 mixture of rotary isomers with 10:1 *Z*/*E*. ^{1}H NMR (500 MHz, $CDCl_3$) δ 8.88 (brs, 0.06H), 8.33 (brs, 0.6H), 7.54–7.32 (m, 2H), 7.29 (brs, 0.6H), 7.22

(brs, 0.4H), 6.87–6.75 (m, 2H), 6.00–5.62 (m, 2H), 5.52 (brs, 1H), 5.43–5.29 (m, 1H), 4.87 (d, *J* = 14.5 Hz, 0.6H), 4.09–3.99 (m, 1H), 3.98–3.89 (m, 0.6H), 3.88–3.69 (m, 5.4H), 3.62 (dd, *J* = 15.4, 9.8 Hz, 0.6H), 3.37 (d, *J* = 16.3 Hz, 0.4H), 3.17–3.06 (m, 0.6H), 3.04–2.93 (m, 3H), 1.81 (dd, *J* = 7.0, 1.8 Hz, 1.6H), 1.77 (dd, *J* = 7.0, 1.8 Hz, 1.1H), 1.73 (dd, *J* = 6.6, 1.6 Hz, 0.2H), 1.71–1.68 (m, 0.1H), 1.48–1.40 (m, 9H); ^{13}C NMR (126 MHz, $CDCl_3$) δ 170.6, 169.9, 169.3, 168.8, 156.2, 155.6, 153.1, 131.7, 131.2, 130.4, 130.2, 126.3, 126.1, 121.8, 120.6, 120.3, 114.3, 114.2, 114.1, 80.2, 67.3, 55.6, 55.5 (2C), 52.1, 51.5, 44.2, 44.1, 41.4 (2C), 40.9, 35.4, 34.2, 34.1, 28.4, 17.9, 14.3, 13.7, 13.6; IR (neat, cm^{-1}): 3295, 1718, 1651, 1513, 1221, 1031, 830; ESI HRMS *m/z* (M+Na)$^+$ calcd 501.2320, obsd 501.2331.

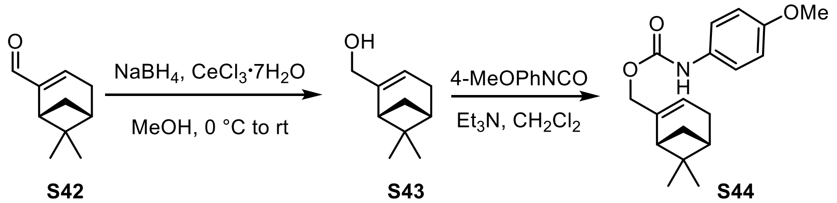

S42 S43 S44

((1*R*,5*S*)-6,6-Dimethylbicyclo[3.1.1]hept-2-en-2-yl)methyl (4-methoxyphenyl)carbamate (S44). The title compound was prepared by the following procedure described for the synthesis of **S66** and obtained as a light yellow oil in 55% yield in two steps. ^{1}H NMR (600 MHz, $CDCl_3$) δ 7.34–7.26 (m, 2H), 6.87–6.81 (m, 2H), 6.55 (brs, 1H), 5.59 (tt, *J* = 3.3, 1.6 Hz, 1H), 4.61–4.53 (m, 1H), 4.50 (d, *J* = 12.6 Hz, 1H), 3.78 (s, 3H), 2.41 (dt, *J* = 8.7, 5.6 Hz, 1H), 2.35–2.29 (m, 1H), 2.28–2.22 (m, 1H), 2.17 (td, *J* = 5.6, 1.5 Hz, 1H), 2.13–2.08 (m, 1H), 1.29 (s, 3H), 1.20 (d, *J* = 8.7 Hz, 1H), 0.84 (s, 3H); ^{13}C NMR (151 MHz, $CDCl_3$) δ 156.1, 154.1, 143.4, 131.2, 121.6, 120.8, 114.4, 67.9, 55.7, 43.6, 40.9, 38.2, 31.7, 31.4, 26.3, 21.2; IR (neat, cm^{-1}): 3320, 2935, 1701, 1514, 1219, 1035, 828, 521; ESI HRMS *m/z* (M+Na)$^+$ calcd 324.1570, obsd 324.1575.

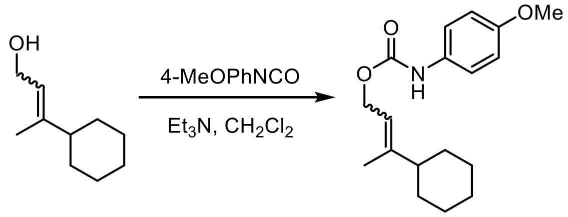

S45 S46

3-Cyclohexylbut-2-en-1-yl (4-methoxyphenyl)carbamate (S46). The title compound (1.3 g, 86%) was prepared as a colorless oil starting from **S45**[14] (0.80 g, 5.0 mmol) by following the GP 1. *E*:*Z* = 10:1; ^{1}H NMR (500 MHz, $CDCl_3$) δ 7.27 (d, *J* = 12.4 Hz, 2H), 6.86–6.78 (m, 2H), 6.66 (s, 0.9H), 6.60 (s, 0.1H), 5.35 (ddt, *J* = 6.9, 5.8, 1.3 Hz, 0.9H), 5.30 (td, *J* = 7.2, 1.6 Hz, 0.1H), 4.68 (d, *J* = 7.0 Hz, 2H), 3.77 (s, 3H), 1.92–1.83 (m, 1H), 1.79–1.73 (m, 2H), 1.73–1.70 (m, 1H), 1.68 (s, 3H), 1.67–1.63 (m, 1H), 1.34–1.24 (m, 2H), 1.24–1.20 (m, 1H), 1.20–1.17 (m, 1H), 1.17–1.11 (m, 2H); Only the *E* configuration has showed; ^{13}C NMR (126 MHz, $CDCl_3$) δ 156.1, 154.2, 147.5, 131.3, 120.8, 116.8, 114.4, 62.3, 55.7, 47.3, 31.8, 26.8, 26.5, 15.0; IR (neat, cm^{-1}):

3320, 2927, 1701, 1515, 1224, 1034, 828, 767; ESI HRMS *m/z* $(M+Na)^+$ calcd 326.1727, obsd 326.1728.

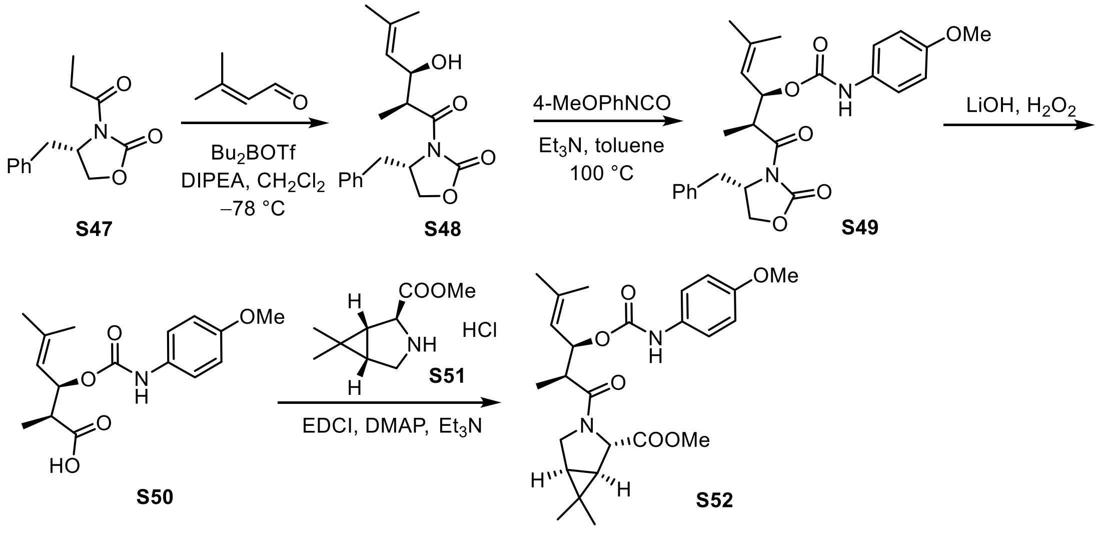

(*S*)-4-Benzyl-3-((2*S*,3*R*)-3-hydroxy-2,5-dimethylhex-4-enoyl)oxazolidin-2-one (S48). To a solution of **S47** (4.5 g, 18 mmol) in CH_2Cl_2 (50 mL) at 0 °C was added dibutylboron triflate (1 M in CH_2Cl_2, 20 mL, 1.1 equiv) dropwise. The resulting reaction mixture was stirred for 15 min at 0 °C. DIPEA (3.5 mL, 22 mmol, 1.1 equiv) was added slowly. After being stirred for 30 min at 0 °C, the reaction mixture was cooled to −78 °C. A solution of 3-methylbut-2-enal (1.7 g, 20 mmol, 1.1 equiv) in CH_2Cl_2 (20 mL) was added dropwise over 20 min. The resulting mixture was warmed to ambient temperature and stirred for 12 h. The reaction was quenched with 10 mL of pH 7 buffer solution (Na_2HPO_4/NaH_2PO_4), followed by H_2O_2 (30%, 10 mL) and MeOH (20 mL). The resulting solution was stirred for 2 h. The residue was diluted with CH_2Cl_2 and washed with saturated $NaHCO_3$ and then brine. The organic phase was dried over $MgSO_4$ and concentrated. The residue was chromatographed through silica gel eluting with EtOAc/hexanes to give the product in 84% (4.8 g) yield as a light-yellow oil. ^1H NMR (500 MHz, $CDCl_3$) δ 7.36–7.31 (m, 2H), 7.30–7.25 (m, 1H), 7.23–7.19 (m, 2H), 5.27 (dp, J = 8.6, 1.5 Hz, 1H), 4.73–4.64 (m, 2H), 4.25–4.14 (m, 2H), 3.88 (qd, J = 7.0, 4.5 Hz, 1H), 3.25, 2.79 (ABX, 2H, J_{AB} = 13.4 Hz, J_{AX} = 3.4 Hz, J_{BX} = 9.4 Hz), 2.56 (d, J = 2.8 Hz, 1H), 1.74 (d, J = 1.5 Hz, 3H), 1.70 (d, J = 1.4 Hz, 3H), 1.29 (d, J = 7.0 Hz, 3H); ^{13}C NMR (126 MHz, $CDCl_3$) δ 176.6, 153.3, 136.8, 135.3, 129.6, 129.1, 127.5, 124.3, 69.3, 66.3, 55.4, 43.2, 38.0, 26.0, 18.5, 12.0; IR (neat, cm^{-1}): 3429, 2975, 1779, 1697, 1386, 1211, 703; ESI HRMS *m/z* $(M+Na)^+$ calcd 340.1519, obsd 340.1521.

(2*S*,3*R*)-1-((*S*)-4-Benzyl-2-oxooxazolidin-3-yl)-2,5-dimethyl-1-oxohex-4-en-3-yl (4-methoxyphenyl)carbamate (S49). To a solution of **S48** (0.96 g, 3.1 mmol) in toluene (10 mL) was added 4-methoxyphenyl isocyanate (0.46 mL, 3.7 mmol, 1.2 equiv) at rt, followed by Et_3N (0.87 mL, 6.2 mmol, 2.0 equiv). The resulting reaction mixture was stirred at 100 °C until complete consumption of the starting material (monitored by TLC). The solvent was removed

under reduced pressure. The residue was chromatographed through silica gel eluting with EtOAc/hexanes to give the product as a light yellow solid (1.2 g, 80%). ^{1}H NMR (500 MHz, $CDCl_3$) δ 7.35–7.29 (m, 2H), 7.29–7.23 (m, 3H), 7.22–7.17 (m, 2H), 6.87–6.79 (m, 2H), 6.46 (brs, 1H), 5.83 (dd, J = 9.2, 5.9 Hz, 1H), 5.24 (dp, J = 9.2, 1.5 Hz, 1H), 4.58 (tt, J = 10.0, 3.0 Hz, 1H), 4.27–4.17 (m, 1H), 4.17–4.06 (m, 2H), 3.77 (s, 3H), 3.27, 2.77 (AB of ABX, 2H, J_{AB} = 13.3 Hz, J_{AX} = 3.4 Hz, J_{BX} = 9.7 Hz), 1.80 (d, J = 1.4 Hz, 3H), 1.74 (d, J = 1.4 Hz, 3H), 1.30 (d, J = 6.9 Hz, 3H); ^{13}C NMR (126 MHz, $CDCl_3$) δ 173.9, 156.2, 153.6, 139.2, 135.5, 131.2, 129.6, 129.2, 129.1, 127.5, 122.2, 121.0, 114.4, 72.4, 66.4, 55.9, 55.7, 42.5, 38.1, 26.1, 18.8, 12.2; IR (neat, cm^{-1}): 3343, 2972, 1779, 1705, 1515, 1386, 1216, 1029, 829, 703; ESI HRMS *m/z* $(M+Na)^+$ calcd 489.1996, obsd 489.2007.

(2*S*,3*R*)-3-(((4-Methoxyphenyl)carbamoyl)oxy)-2,5-dimethylhex-4-enoic acid (S50). To a solution of **S49** (0.84 g, 1.8 mmol, 1.0 equiv) in THF (20 mL) and H_2O (5 mL) was added lithium hydroxide monohydrate (0.15 g, 3.6 mmol, 2.0 equiv) and H_2O_2 (30%, 0.74 mL). The reaction mixture was stirred for 2 h at rt. THF was removed under reduced pressure. The excess H_2O_2 was quenched by addition of saturated aqueous $Na_2S_2O_3$ (10 mL). The reaction mixture was extracted with Et_2O to remove any neutral organic impurities. The aqueous layer was acidified with HCl (1 N) to pH 2 and then extracted three times with CH_2Cl_2. The combined organic solution was washed with brine, dried over anhydrous Na_2SO_4, filtered and concentrated under reduced pressure to afford **S50** as a light-yellow oil (0.42 g, 76% yield). The crude product was used in the following step without purification.

Methyl (1*R*,2*S*,5*S*)-3-((2*S*,3*R*)-3-(((4-methoxyphenyl)carbamoyl)oxy)-2,5-dimethylhex-4-enoyl)-6,6-dimethyl-3-azabicyclo[3.1.0]hexane-2-carboxylate (S52). To a solution of **S50** (0.42 g, 1.4 mmol, 1.0 equiv) in CH_2Cl_2 (20 mL) was added EDCI (0.39 g, 2.0 mmol, 1.5 equiv), followed by **S51** (0.42 g, 2.0 mmol, 1.5 equiv) and DMAP (20 mg, 0.14 mmol, 0.10 equiv). The resulting reaction mixture was stirred at rt for 8 h, diluted with EtOAc, and quenched with HCl (1 N, 15 mL). The layers were separated, and the aqueous layer was extracted twice with EtOAc. The combined organic solution was washed with brine, dried over anhydrous $MgSO_4$, filtered, and concentrated under reduced pressure. The residue was chromatographed through silica gel eluting with CH_2Cl_2/MeOH to afford **S52** as a white solid and a 7:3 mixture of rotary isomers (63% yield, 0.40 g). ^{1}H NMR (500 MHz, $CDCl_3$) δ 7.39–7.14 (m, 2.7H), 6.83 (d, J = 8.5 Hz, 2H), 6.44 (brs, 0.3H), 5.62–5.56 (m, 0.7H), 5.56–5.50 (m, 0.3H), 5.43 (d, J = 9.5 Hz, 0.7H), 5.12 (d, J = 9.6 Hz, 0.3H), 4.44 (s, 0.7H), 4.38 (s, 0.3H), 3.88 (dd, J = 10.1, 5.1 Hz, 0.7H), 3.82–3.70 (m, 6.6H), 3.51 (d, J = 10.0 Hz, 0.7H), 3.05–2.90 (m, 0.7H), 2.65–2.52 (m, 0.3H), 1.75 (d, J = 7.6 Hz, 6H), 1.54–1.37 (m, 2H), 1.14–1.01 (m, 6H), 0.98–0.92 (m, 3H); ^{13}C NMR (126 MHz, $CDCl_3$) δ 172.7, 172.5, 172.3, 172.2, 156.0, 155.8, 153.5, 153.3, 139.9, 139.2, 131.7, 131.2, 121.3, 120.6, 120.4, 114.4, 114.3, 74.4, 73.3, 60.5, 59.3, 55.6, 52.8, 52.5, 47.5, 46.8, 43.3, 41.6, 32.7, 30.8, 27.5, 26.3 (2C), 26.2, 26.1 (2C), 19.8, 19.6, 18.7, 18.6, 14.4, 13.2, 12.7, 12.6;

IR (neat, cm^{-1}): 3421, 3256, 1737, 1629, 1549, 1224, 1034, 827, 736; ESI HRMS *m/z* (M+Na)$^+$ calcd 481.2309, obsd 481.2320.

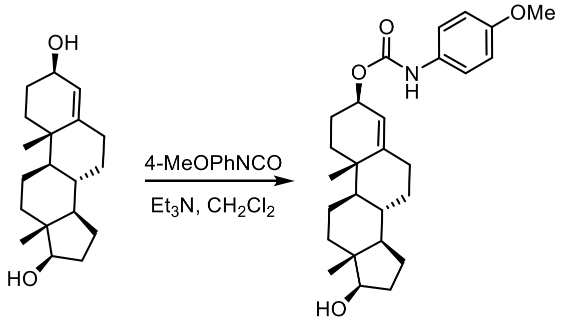

S53 **S54**

(3*S*,8*R*,9*S*,10*R*,13*S*,14*S*,17*S*)-17-Hydroxy-10,13-dimethyl-2,3,6,7,8,9,10,11,12,13,14,15,16, 17-tetradecahydro-1*H*-cyclopenta[*a*]phenanthren-3-yl (4-methoxyphenyl)carbamate (S54). The title compound (0.34 g, 35%) was prepared as a white solid starting from **S53**[15] (0.67 g, 2.0 mmol) by following the GP 1. ^{1}H NMR (500 MHz, $CDCl_3$) δ 7.32–7.27 (m, 2H), 6.88–6.80 (m, 2H), 6.54 (brs, 1H), 5.32 (d, *J* = 2.3 Hz, 1H), 5.22 (t, *J* = 8.1 Hz, 1H), 3.78 (s, 3H), 3.63 (t, *J* = 8.7 Hz, 1H), 2.27–2.15 (m, 1H), 2.04 (dt, *J* = 14.5, 6.8 Hz, 2H), 1.82 (dt, *J* = 12.5, 3.4 Hz, 1H), 1.79–1.72 (m, 2H), 1.66–1.52 (m, 4H), 1.50–1.41 (m, 2H), 1.40–1.30 (m, 2H), 1.26 (td, *J* = 12.3, 5.9 Hz, 1H), 1.12–1.00 (m, 4H), 0.98–0.81 (m, 2H), 0.81–0.72 (m, 4H); ^{13}C NMR (151 MHz, $CDCl_3$) δ 156.0, 153.9, 149.4, 131.3, 120.7, 119.6, 114.4, 81.9, 71.6, 55.7, 54.5, 50.8, 43.0, 37.6, 36.7, 36.1, 35.2, 32.6, 32.3, 30.6, 25.6, 23.5, 20.7, 19.1, 11.2; IR (neat, cm^{-1}): 3331, 2923, 1713, 1504, 1226, 830; ESI HRMS *m/z* (M+Na)$^+$ calcd 462.2615, obsd 462.2624.

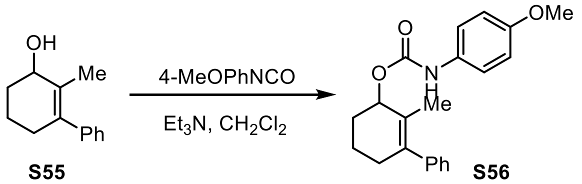

S55 **S56**

2-Methyl-3,4,5,6-tetrahydro-[1,1'-biphenyl]-3-yl (4-methoxyphenyl)carbamate (S56). The title compound (0.69 g, 79%) was prepared as a white solid starting from **S55**[16] (0.56 g, 3.0 mmol) by following the GP 1. ^{1}H NMR (600 MHz, $CDCl_3$) δ 7.47–7.32 (m, 4H), 7.32–7.25 (m, 1H), 7.23–7.15 (m, 2H), 6.95–6.84 (m, 2H), 6.73 (brs, 1H), 5.39 (t, *J* = 4.2 Hz, 1H), 3.82 (s, 3H), 2.43–2.34 (m, 1H), 2.33–2.24 (m, 1H), 2.06 (ddt, *J* = 13.3, 6.7, 3.3 Hz, 1H), 1.98–1.90 (m, 1H), 1.86 (tdd, *J* = 11.9, 5.5, 2.9 Hz, 1H), 1.82–1.75 (m, 1H), 1.71–1.63 (m, 3H); ^{13}C NMR (151 MHz, $CDCl_3$) δ 156.0, 154.2, 143.1, 139.2, 131.4, 128.3, 128.1, 127.2, 126.7, 120.6, 114.4, 72.6, 55.6, 32.2, 29.2, 19.0, 17.7; IR (neat, cm^{-1}): 3319, 2932, 1699, 1514, 1223, 1033, 828; ESI HRMS *m/z* (M+Na)$^+$ calcd 360.1570, obsd 360.1578.

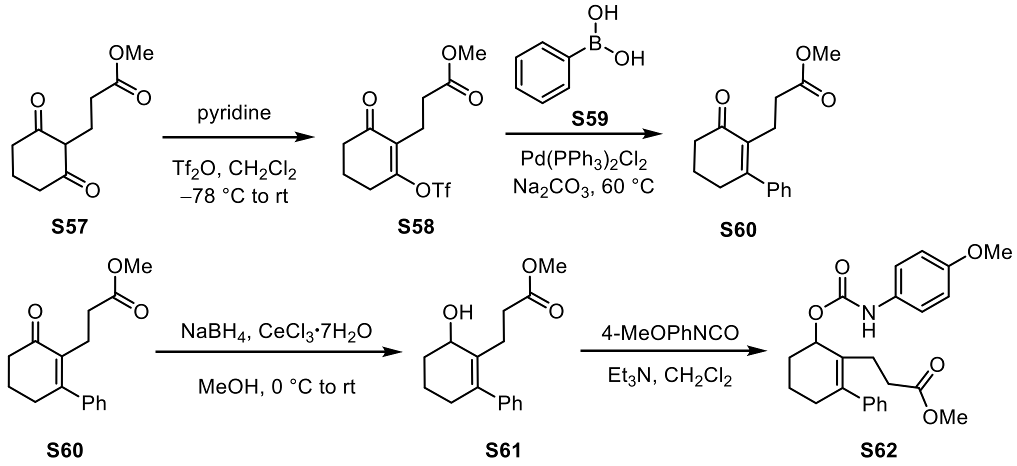

Methyl 3-(6-oxo-2-(((trifluoromethyl)sulfonyl)oxy)cyclohex-1-en-1-yl)propanoate (S58). To a solution of **S57**[17] (0.50 g, 2.5 mmol, 1.0 equiv) and pyridine (0.40 mL, 5.0 mmol, 2.0 equiv) in CH_2Cl_2 (10 mL) at –78 °C was slowly added trifluoromethanesulfonic anhydride (0.50 mL, 3.0 mmol, 1.2 equiv). The reaction mixture was stirred at the same temperature for 10 min and at rt for 4 h. The reaction was quenched with HCl (1 N, 5 mL). The resulting mixture was extracted with Et_2O. The combined organic layers were washed with 10 mL of a saturated $NaHCO_3$ solution, followed by 10 mL of brine. The resulting organic phase was dried over Na_2SO_4, filtered, and concentrated in vacuo to afford the title compound as a brown oil in 84% yield (0.69 g). This compound was used in the following step without purification.

Methyl 3-(3-oxo-3,4,5,6-tetrahydro-[1,1'-biphenyl]-2-yl)propanoate (S60). S58 (0.69 g, 2.1 mmol), **S59** (0.30 g, 2.5 mmol, 1.2 equiv), $Pd(PPh_3)_2Cl_2$ (0.20 g, 0.20 mmol, 0.10 equiv) and Na_2CO_3 (1.1 g, 11 mmol, 5.0 equiv) were dissolved in a mixture of 1,4-dioxane/H_2O (2.5:1, 14 mL). The resulting mixture was deoxygenated with a stream of argon for 10 min, then heated to 60 °C until completion of the reaction (monitored by ^{1}H NMR). The mixture was cooled down to rt and quenched with saturated NH_4Cl. The reaction mixture was extracted with EtOAc. The combined organic solution was washed with H_2O and brine, dried over anhydrous Na_2SO_4, filtered and evaporated under reduced pressure. The residue was purified by silica gel column chromatography to give the desired **S59** as a light yellow oil (0.41 g, 74%). This compound was used in the following step without purification.

Methyl 3-(3-(((4-methoxyphenyl)carbamoyl)oxy)-3,4,5,6-tetrahydro-[1,1'-biphenyl]-2-yl) propanoate (S62). The title compound was prepared by following the procedure described for the synthesis of **S66**. White solid; 44% yield in two steps; ^{1}H NMR (500 MHz, $CDCl_3$) δ 7.37–7.28 (m, 4H), 7.28–7.22 (m, 1H), 7.14–7.06 (m, 2H), 6.90–6.81 (m, 2H), 6.66 (brs, 1H), 5.48–5.34 (m, 1H), 3.78 (s, 3H), 3.55 (s, 3H), 2.43–2.35 (m, 1H), 2.34–2.18 (m, 5H), 2.07–1.99 (m, 1H), 1.93–1.76 (m, 2H), 1.77–1.68 (m, 1H); ^{13}C NMR (126 MHz, $CDCl_3$) δ 173.6, 156.1, 153.9, 142.8, 141.9, 131.3, 129.5, 128.5, 127.7, 127.0, 120.7, 114.4, 70.1, 55.7, 51.6, 33.3, 33.0, 29.2, 26.2, 18.6; IR (neat, cm^{-1}): 3343, 2947, 1721, 1514, 1219, 1031, 763, 703; ESI HRMS *m/z*

(M+Na)+ calcd 432.1781, obsd 432.1792.

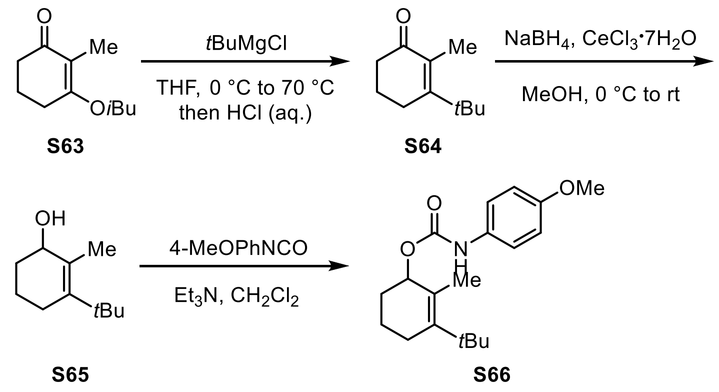

3-(*tert*-Butyl)-2-methylcyclohex-2-en-1-one (S64). To a solution of **S63**[18] (1.5 g, 8.0 mmol, 1.0 equiv) in THF (10 mL) at 0 °C was added *t*BuMgCl (1 M in THF, 1.2 equiv) dropwise over 10 min. Upon complete addition, the reaction mixture was warmed to 70 °C and stirred for 12 h. The mixture was quenched by HCl (10% aq., 10 mL) and extracted by EtOAc. The organic layer was washed by saturated $NaHCO_3$ and brine and concentrated under reduced pressure. The residue was chromatographed through silica gel eluting with EtOAc/hexanes to give the product in 21% yield (0.30 g). The product was used in the following step without purification.

3-(*tert*-Butyl)-2-methylcyclohex-2-en-1-ol (S65). To a solution of $CeCl_3 \cdot 7H_2O$ (0.70 g, 1.9 mmol, 1.1 equiv) and **S64** (0.28 g, 1.7 mmol, 1.0 equiv) in MeOH (15 mL) at 0 °C was added $NaBH_4$ (0.20 g, 5.1 mmol, 3.0 equiv) in several portions. The resulting reaction mixture was stirred at rt for 1 h, quenched with H_2O, and extracted three times with EtOAc. The combined organic solution was dried over $MgSO_4$ and concentrated under reduced pressure to afford the title compound as a colorless oil in 70% yield (0.17 g). This compound was used in the following step without purification.

3-(*tert*-Butyl)-2-methylcyclohex-2-en-1-yl (4-methoxyphenyl)carbamate (S66). The title compound (0.15 g, 48%) was prepared as a white solid starting from **S65** (0.17 g, 1.0 mmol) by following the GP 1. ^{1}H NMR (600 MHz, $CDCl_3$) δ 7.37–7.27 (m, 2H), 6.89–6.79 (m, 2H), 6.59 (brs, 1H), 5.10 (t, *J* = 4.0 Hz, 1H), 3.77 (s, 3H), 2.16 (dt, *J* = 18.4, 4.8 Hz, 1H), 2.02–1.92 (m, 1H), 1.90–1.78 (m, 4H), 1.68–1.49 (m, 3H), 1.17 (s, 9H); ^{13}C NMR (151 MHz, $CDCl_3$) δ 155.9, 154.3, 144.9, 131.5, 125.0, 120.4, 114.4, 75.4, 55.6, 36.2, 30.2, 29.0, 28.1, 19.7, 19.1; IR (neat, cm^{-1}): 3324, 2947, 1698, 1515, 1220, 1030, 828; ESI HRMS *m/z* $(M+Na)^+$ calcd 340.1883, obsd 340.1893.

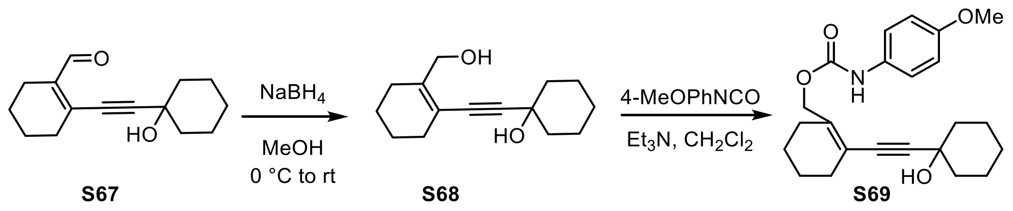

1-((2-(Hydroxymethyl)cyclohex-1-en-1-yl)ethynyl)cyclohexan-1-ol (S68). To a solution of

S67[19] (0.80 g, 3.4 mmol, 1.0 equiv) in MeOH (15 mL) at 0 °C was added $NaBH_4$ (0.20 g, 20 mmol, 6.0 equiv) in several portions. The resulting reaction mixture was stirred at rt for 1 h, quenched with H_2O (10 mL), and extracted three times with EtOAc. The combined organic solution was dried over $MgSO_4$ and concentrated under reduced pressure to afford the title compound as a colorless oil in 70% yield (0.56 g). This compound was used in the following step without purification.

(2-((1-Hydroxycyclohexyl)ethynyl)cyclohex-1-en-1-yl)methyl (4-methoxyphenyl)carbamate (S69). The title compound (0.54 g, 59%) was prepared as a colorless oil starting from **S68** (0.56 g, 2.4 mmol) by following the GP 1. ^{1}H NMR (500 MHz, $CDCl_3$) δ 7.34–7.27 (m, 2H), 6.90–6.78 (m, 2H), 6.69 (brs, 1H), 4.88 (s, 2H), 3.77 (s, 3H), 2.58 (brs, 1H), 2.30–2.07 (m, 4H), 1.94–1.85 (m, 2H), 1.73–1.47 (m, 11H), 1.27–1.16 (m, 1H); ^{13}C NMR (151 MHz, $CDCl_3$) δ 156.0, 154.2, 139.0, 131.2, 120.7, 119.8, 114.4, 97.1, 83.2, 69.2, 67.0, 55.6, 40.2, 30.4, 27.2, 25.4, 23.6, 22.2, 22.1; IR (neat, cm^{-1}): 3313, 2933, 1701, 1514, 1226, 1054, 829; ESI HRMS *m/z* (M+Na)$^+$ calcd 406.1989, obsd 406.1997.

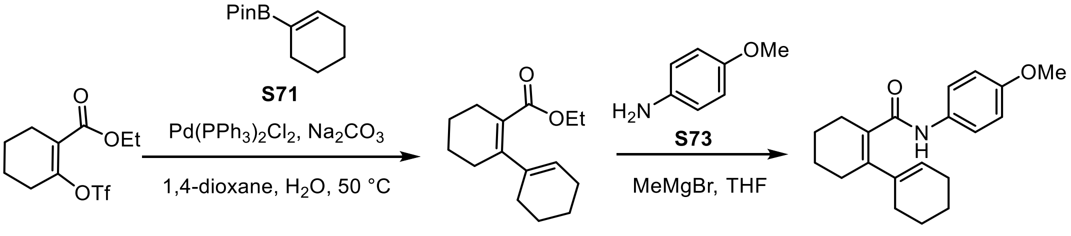

S70 S72 S74

***N*-(4-Methoxyphenyl)-[1,1'-bi(cyclohexane)]-1,1'-diene-2-carboxamide (S74).** The title compound was obtained as a light-yellow solid (0.72 g, 46% yield in two steps) starting from **S70**[20] (1.5 g, 5.0 mmol, 1.0 equiv) by following the procedure described for the synthesis of **S77**. ^{1}H NMR (500 MHz, $CDCl_3$) δ 7.74 (brs, 1H), 7.46–7.35 (m, 2H), 6.89–6.81 (m, 2H), 5.78–5.64 (m, 1H), 3.78 (s, 3H), 2.45–2.34 (m, 2H), 2.26–2.13 (m, 2H), 2.13–2.03 (m, 4H), 1.67–1.62 (m, 4H), 1.62–1.57 (m, 2H), 1.57–1.50 (m, 2H); ^{13}C NMR (126 MHz, $CDCl_3$) δ 168.8, 156.3, 143.5, 141.0, 131.7, 130.0, 125.5, 121.1, 114.3, 55.6, 31.2, 28.7, 26.4, 25.5, 23.0, 22.7, 22.3, 22.1; IR (neat, cm^{-1}): 3313, 2923, 1651, 1511, 1235, 827; ESI HRMS *m/z* (M+Na)$^+$ calcd 334.1778, obsd 334.1784.

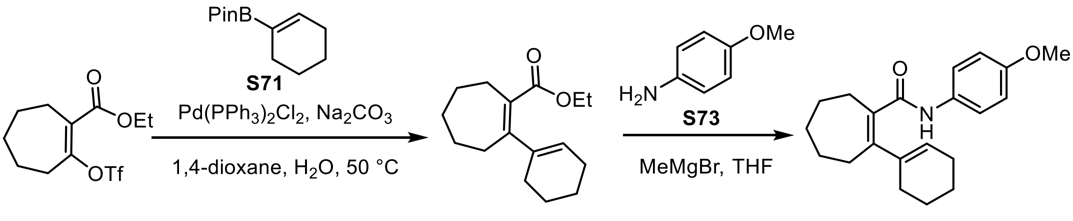

S75 S76 S77

Ethyl 2-(cyclohex-1-en-1-yl)cyclohept-1-ene-1-carboxylate (S76). S71 (5.0 g, 24 mmol, 1.2 equiv), **S75**[20] (6.0 g, 20 mmol, 1.0 equiv), $Pd(PPh_3)_2Cl_2$ (0.70 g, 0.10 mmol, 0.050 equiv) and Na_2CO_3 (8.5 g, 80 mmol, 4.0 equiv) were dissolved in a mixture of 1,4-dioxane/H_2O (2.5:1, 56

mL). The resulting mixture was deoxygenated with a stream of argon for 10 min, then heated to 50 °C until completion of the reaction (monitored by ^{1}H NMR). The mixture was cooled to rt and quenched with saturated NH_4Cl (10 mL). The reaction mixture was extracted with EtOAc. The combined organic solution was washed with H_2O and brine, dried over anhydrous Na_2SO_4, filtered and evaporated under reduced pressure. The residue was purified by silica gel column chromatography to give the desired **S76** as a colorless oil (3.3 g, 66%). The product was directly used without further purification.

2-(Cyclohex-1-en-1-yl)-*N*-(4-methoxyphenyl)cyclohept-1-ene-1-carboxamide (S77). To a solution of CH_3MgBr (3 M in Et_2O, 1.3 mL, 2.0 equiv) in Et_2O (20 mL) at 0 °C was added a solution of **S73** (0.49 g, 4.0 mmol, 2.0 equiv) in Et_2O (20 mL) dropwise. Upon complete addition, the reaction mixture was stirred for 10 min at 0 °C before dropwise addition of **S76** (0.47 g, 2.0 mmol, 1.0 equiv). The reaction mixture was warmed to ambient temperature and stirred for 1 h. Saturated aqueous NH_4Cl was added to quench the reaction. The resulting mixture was extracted with EtOAc. The combined organic solution was washed with brine, dried over anhydrous $MgSO_4$, filtered, and concentrated under reduced pressure. The residue was chromatographed through silica gel eluting with EtOAc/hexanes to afford **S77** as a brown solid in 65% yield (0.55 g). ^{1}H NMR (600 MHz, $CDCl_3$) δ 7.48 (brs, 1H), 7.42–7.35 (m, 2H), 6.87–6.82 (m, 2H), 5.66 (tt, J = 3.9, 1.7 Hz, 1H), 3.78 (s, 3H), 2.55–2.48 (m, 2H), 2.37–2.29 (m, 2H), 2.15–2.06 (m, 2H), 2.06–1.99 (m, 2H), 1.82–1.76 (m, 2H), 1.64–1.49 (m, 8H); ^{13}C NMR (151 MHz, $CDCl_3$) δ 169.9, 156.3, 149.5, 141.3, 136.0, 131.7, 124.7, 121.2, 114.3, 55.6, 34.8, 32.6, 30.6, 28.3, 27.0, 26.4, 25.4, 22.9, 22.1; IR (neat, cm^{-1}): 3313, 2923, 1651, 1511, 1235, 827; ESI HRMS *m/z* $(M+Na)^+$ calcd 348.1934, obsd 348.1939.

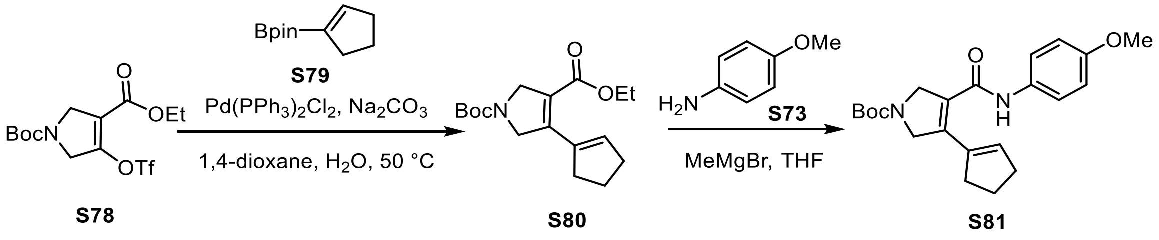

1-(*tert*-Butyl) 3-ethyl 4-(cyclopent-1-en-1-yl)-2,5-dihydro-1*H*-pyrrole-1,3-dicarboxylate (S80). The title compound was obtained as a colorless oil (0.75 g, 85%) starting from **S78** (1.1 g, 3.0 mmol, 1.0 equiv) by following the procedure described for the synthesis of **S76**. The compound exists as a mixture of rotary isomers; ^{1}H NMR (600 MHz, $CDCl_3$) δ 6.19–6.04 (m, 1H), 4.46–4.40 (m, 2H), 4.40–4.34 (m, 2H), 4.26–4.17 (m, 2H), 2.68–2.58 (m, 2H), 2.48–2.41 (m, 2H), 1.95–1.87 (m, 2H), 1.48 (s, 9H), 1.34–1.26 (m, 3H); ^{13}C NMR (151 MHz, $CDCl_3$) δ 164.2, 164.1, 154.1, 154.0, 142.0, 141.8, 136.9, 136.8, 136.7, 136.4, 122.9, 122.6, 80.0 (2C), 60.8, 60.7, 56.8, 56.7, 55.1, 55.0, 34.2, 33.2 (2C), 28.7, 23.9, 14.4, 14.3; IR (neat, cm^{-1}): 3379, 2978, 1713, 1397, 1255, 1152, 771; ESI HRMS *m/z* $(M+Na)^+$ calcd 330.1676, obsd 330.1690.

***tert*-Butyl 3-(cyclopent-1-en-1-yl)-4-((4-methoxyphenyl)carbamoyl)-2,5-dihydro-1*H*-pyrrole-1-carboxylate (S81).** The title compound was obtained as a light-yellow oil (0.54 g, 70% yield) starting from **S80** (0.59 g, 2.0 mmol, 1.0 equiv) by following the procedure described for the synthesis of **S77**. The compound exists as a 3:2 mixture of rotary isomers. ^{1}H NMR (500 MHz, $CDCl_3$) δ 7.95 (brs, 0.6H), 7.79 (brs, 0.4H), 7.51–7.39 (m, 2H), 6.95–6.80 (m, 2H), 6.01–5.86 (m, 1H), 4.58–4.42 (m, 2H), 4.40–4.26 (m, 2H), 3.79 (s, 3H), 2.62–2.53 (m, 2H), 2.50–2.39 (m, 2H), 1.92 (tt, J = 7.5 Hz, 2H), 1.53–1.43 (m, 9H); ^{13}C NMR (126 MHz, $CDCl_3$) δ 163.2, 156.7, 154.2, 154.0, 136.7, 136.6, 135.3, 135.0, 134.8, 134.4, 130.8 (2C), 128.1, 127.9, 121.7, 121.6, 114.4, 80.1, 80.0, 56.1, 56.0, 55.6, 55.5, 55.3, 33.8, 33.1 (2C), 28.6, 23.8; IR (neat, cm^{-1}): 3281, 2975, 1699, 1511, 1247, 830; ESI HRMS *m/z* $(M+Na)^+$ calcd 407.1941, obsd 407.1948.

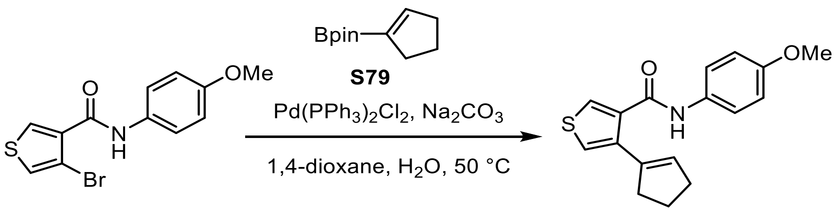

S82 S83

4-(Cyclopent-1-en-1-yl)-*N*-(4-methoxyphenyl)thiophene-3-carboxamide (S83). The title compound was obtained as a white solid (0.48 g, 80% yield) starting from **S82**[21] (0.62 g, 2.0 mmol, 1.0 equiv) by following the procedure described for the synthesis of **S76**. ^{1}H NMR (500 MHz, $CDCl_3$) δ 7.87 (d, J = 3.7 Hz, 2H), 7.54–7.40 (m, 2H), 7.11 (d, J = 3.1 Hz, 1H), 6.94–6.84 (m, 2H), 6.07–6.02 (m, 1H), 3.80 (s, 3H), 2.75–2.63 (m, 2H), 2.59– 2.48 (m, 2H), 2.08–1.95 (m, 2H); ^{13}C NMR (126 MHz, $CDCl_3$) δ 162.8, 156.7, 138.1, 137.1, 136.6, 131.3, 131.0, 130.5, 123.5, 121.6, 114.5, 55.7, 36.5, 33.8, 23.6; IR (neat, cm^{-1}): 3269, 2951, 1651, 1510, 1242, 1035, 829; ESI HRMS *m/z* $(M+Na)^+$ calcd 322.0872, obsd 322.0876.

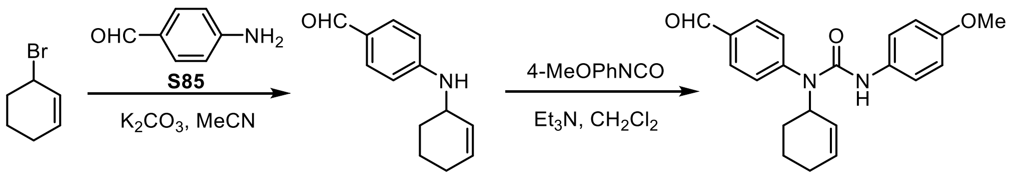

S84 S86 S87

1-(Cyclohex-2-en-1-yl)-1-(4-formylphenyl)-3-(4-methoxyphenyl)urea (S87). The title compound (0.64 g, 18% yield in two steps) was prepared as a light-yellow solid starting from **S84** (1.2 mL, 10 mmol) by following the GP 4. ^{1}H NMR (500 MHz, $CDCl_3$) δ 10.02 (s, 1H), 7.98–7.90 (m, 2H), 7.50–7.43 (m, 2H), 7.22–7.13 (m, 2H), 6.83–6.74 (m, 2H), 5.93 (brs, 1H), 5.80 (ddt, J = 9.9, 4.7, 2.5 Hz, 1H), 5.75–5.69 (m, 1H), 5.35–5.28 (m, 1H), 3.75 (s, 3H), 2.04–1.81 (m, 3H), 1.69–1.58 (m, 2H), 1.50–1.41 (m, 1H); ^{13}C NMR (126 MHz, $CDCl_3$) δ 191.1, 156.0, 154.3, 145.2, 135.7, 131.8, 131.1, 131.0, 130.7, 129.2, 121.9, 114.1, 55.6, 53.4, 28.3, 24.5, 21.5; IR (neat, cm^{-1}): 3354, 2936, 1664, 1599, 1513, 1242, 829, 556; ESI HRMS *m/z*

(M+Na)$^+$ calcd 373.1523, obsd 373.1533.

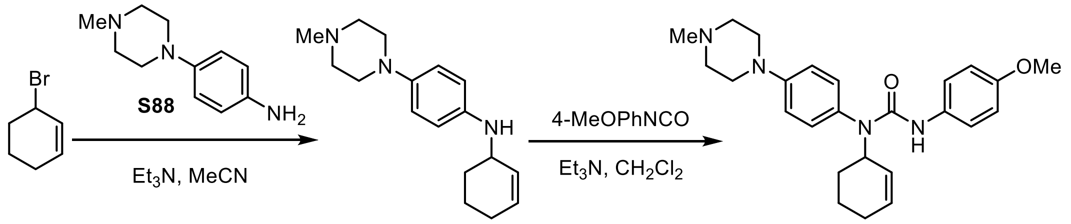

S84 S89 S90

1-(Cyclohex-2-en-1-yl)-3-(4-methoxyphenyl)-1-(4-(4-methylpiperazin-1-yl)phenyl)urea (S90). The title compound was obtained as a colorless oil (3.4 g, 80% yield in two steps) starting from **S88** (1.9 g, 10 mmol, 1.0 equiv) by following the procedure described for the synthesis of **S99**. ^{1}H NMR (500 MHz, $CDCl_3$) δ 7.18–7.14 (m, 2H), 7.13–7.08 (m, 2H), 6.95–6.90 (m, 2H), 6.78–6.74 (m, 2H), 5.92 (brs, 1H), 5.74–5.65 (m, 2H), 5.33–5.24 (m, 1H), 3.73 (s, 3H), 3.30–3.24 (m, 4H), 2.61–2.55 (m, 4H), 2.36 (s, 3H), 1.97–1.75 (m, 3H), 1.65–1.56 (m, 2H), 1.47–1.36 (m, 1H); ^{13}C NMR (126 MHz, $CDCl_3$) δ 155.5, 155.4, 150.9, 132.4, 131.6, 129.8 (2C), 129.1, 121.5, 116.1, 114.0, 55.6, 55.1, 52.2, 48.4, 46.2, 28.3, 24.5, 21.5; IR (neat, cm^{-1}): 3420, 2936, 1666, 1513, 1240, 827, 557; ESI HRMS *m/z* (M+Na)$^+$ calcd 443.2417, obsd 443.2423.

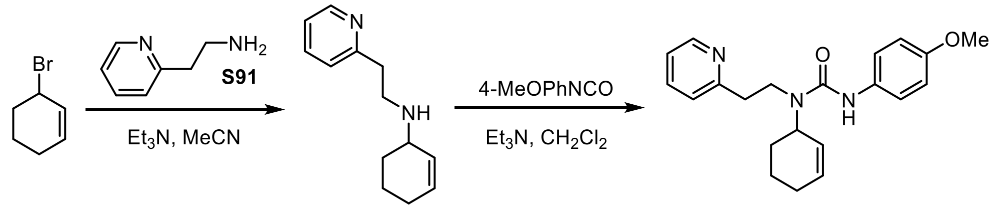

S84 S92 S93

1-(Cyclohex-2-en-1-yl)-3-(4-methoxyphenyl)-1-(2-(pyridin-2-yl)ethyl)urea (S93). The title compound was obtained as a colorless oil (2.4 g, 67% yield in two steps) starting from **S91** (1.2 g, 10 mmol, 1.0 equiv) by following the procedure described for the synthesis of **S99**. ^{1}H NMR (500 MHz, $CDCl_3$) δ 8.71 (brs, 1H), 8.63–8.54 (m, 1H), 7.68–7.56 (m, 1H), 7.49–7.40 (m, 2H), 7.20–7.14 (m, 2H), 6.88–6.80 (m, 2H), 6.02–5.84 (m, 1H), 5.56 (dtt, J = 10.1, 2.6, 1.4 Hz, 1H), 5.02–4.76 (m, 1H), 3.76 (s, 3H), 3.75–3.69 (m, 1H), 3.62 (dt, J = 15.6, 6.8 Hz, 1H), 3.21–3.04 (m, 2H), 2.14–1.93 (m, 3H), 1.87–1.77 (m, 1H), 1.72–1.53 (m, 2H); ^{13}C NMR (126 MHz, $CDCl_3$) δ 159.5, 155.9, 155.2, 149.2, 137.0, 133.7, 131.4, 129.8, 123.6, 121.9, 121.6, 114.0, 55.6 (2C), 52.7, 43.9, 39.6, 28.2, 24.8, 21.8; IR (neat, cm^{-1}): 3294, 2934, 1639, 1511, 1241, 828; ESI HRMS *m/z* (M+Na)$^+$ calcd 374.1839, obsd 374.1848.

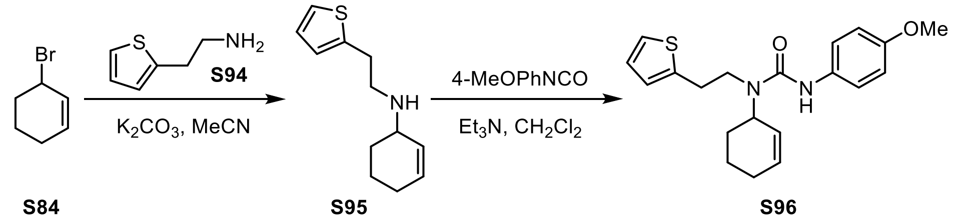

S84 S95 S96

1-(Cyclohex-2-en-1-yl)-3-(4-methoxyphenyl)-1-(2-(thiophen-2-yl)ethyl)urea (S96). The title compound (1.1 g, 91% yield in two steps) was prepared as a white solid starting from **S84** (0.81 g, 5.0 mmol) by following the GP 4. ^{1}H NMR (500 MHz, $CDCl_3$) δ 7.22–7.17 (m, 2H), 7.16 (dd, J = 5.1, 1.2 Hz, 1H), 6.95 (dd, J = 5.1, 3.4 Hz, 1H), 6.90–6.86 (m, 1H), 6.83–6.77 (m, 2H), 6.51 (brs, 1H), 6.04–5.93 (m, 1H), 5.72–5.56 (m, 1H), 4.47–4.22 (m, 1H), 3.83–3.68 (m, 4H), 3.40 (ddd, J = 14.6, 8.5, 6.5 Hz, 1H), 3.22–3.02 (m, 2H), 2.07 (ddt, J = 8.4, 6.2, 3.5 Hz, 2H), 2.01–1.93 (m, 1H), 1.85 (dt, J = 12.7, 3.7 Hz, 1H), 1.75 (tdd, J = 12.7, 10.3, 2.8 Hz, 1H), 1.69–1.57 (m, 1H); ^{13}C NMR (126 MHz, $CDCl_3$) δ 155.7 (2C), 141.7, 132.5, 132.2, 129.7, 127.2, 125.5, 124.0, 121.8, 114.2, 55.6, 55.2, 48.8, 30.6, 27.8, 24.7, 21.9; IR (neat, cm^{-1}): 3377, 2934, 1633, 1511, 1275, 826; ESI HRMS *m/z* $(M+Na)^+$ calcd 379.1451, obsd 379.1460.

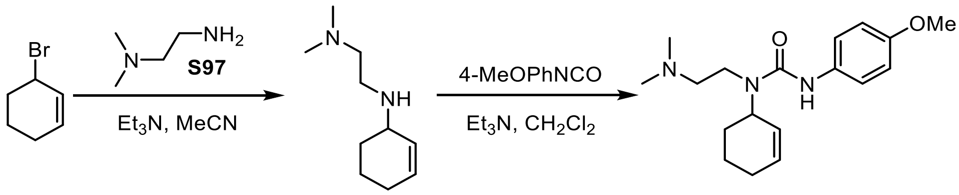

S84 **S98** **S99**

***N^1*-(Cyclohex-2-en-1-yl)-*N^2*,*N^2*-dimethylethane-1,2-diamine (S98).** To a solution of **S97** (0.44 g, 5.0 mmol, 1.0 equiv) in CH_3CN (1 M), was added Et_3N (1.4 mL, 10 mmol, 2.0 equiv), followed by **S84** (0.60 mL, 5.3 mmol, 1.1 equiv). The resulting reaction mixture was stirred for 12 h at rt. Solvent was removed under reduced pressure. The residue was chromatographed through silica gel eluting with EtOAc/hexanes to afford the product as a light-yellow oil in 44% yield (0.70 g), which was directly used in the following step without further purification.

1-(Cyclohex-2-en-1-yl)-1-(2-(dimethylamino)ethyl)-3-(4-methoxyphenyl)urea (S99). The title compound (0.39 g, 56%) was prepared starting from **S98** (0.37 g, 2.2 mmol, 1.0 equiv) by following the GP 1. White solid; ^{1}H NMR (500 MHz, $CDCl_3$) δ 10.80 (brs, 1H), 7.31–7.27 (m, 2H), 6.85–6.79 (m, 2H), 5.88 (ddt, J = 10.2, 6.6, 3.2 Hz, 1H), 5.49 (dt, J = 10.2, 2.0 Hz, 1H), 5.03–4.93 (m, 1H), 3.76 (s, 3H), 3.32–3.15 (m, 2H), 2.63–2.49 (m, 2H), 2.37 (s, 6H), 2.04–1.94 (m, 3H), 1.84–1.75 (m, 1H), 1.67 (tddd, J = 12.9, 9.6, 7.6, 2.9 Hz, 1H), 1.39 (tdd, J = 12.8, 10.1, 3.2 Hz, 1H); ^{13}C NMR (126 MHz, $CDCl_3$) δ 158.0, 154.7, 134.6, 131.5, 129.7, 120.4, 114.2, 63.1, 55.7, 52.5, 45.6, 42.2, 28.1, 24.8, 21.8; IR (neat, cm^{-1}): 2934, 2858, 1664, 1511, 1459, 1237, 1038, 827; ESI HRMS *m/z* $(M+H)^+$ calcd 318.2176, obsd 318.2182.

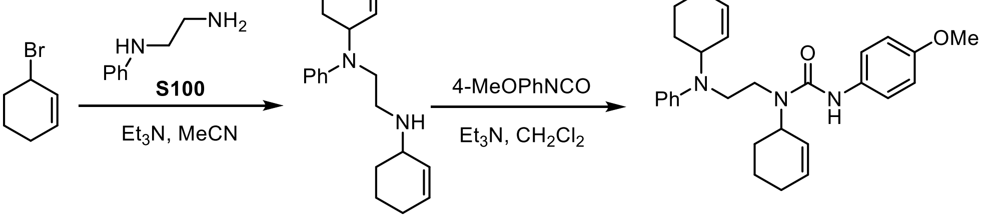

S84 **S101** **S102**

1-(Cyclohex-2-en-1-yl)-1-(2-(cyclohex-2-en-1-yl(phenyl)amino)ethyl)-3-(4-methoxyphenyl)urea (S102). The title compound was obtained as a colorless oil (0.53 g, 12% yield in two steps) starting from **S100** (1.4 g, 10 mmol, 1.0 equiv) by following the procedure described for the synthesis of **S99**. The compound was isolated as a 1:1 mixture of diastereisomers. ^{1}H NMR (500 MHz, $CDCl_3$) δ 7.94–7.64 (m, 1H), 7.33–7.25 (m, 2H), 7.21–7.05 (m, 2H), 7.01–6.94 (m, 2H), 6.86–6.80 (m, 1H), 6.79–6.73 (m, 2H), 6.01–5.87 (m, 2H), 5.70 (ddd, *J* = 11.8, 5.8, 3.9 Hz, 1H), 5.65 (dt, *J* = 10.4, 1.9 Hz, 0.5H), 5.57 (dt, *J* = 10.2, 1.9 Hz, 0.5H), 4.94–4.67 (m, 1H), 4.50–4.39 (m, 1H), 3.77–3.70 (m, 3H), 3.50–3.27 (m, 4H), 2.11–1.94 (m, 5H), 1.92–1.75 (m, 3H), 1.72–1.49 (m, 4H); ^{13}C NMR (126 MHz, $CDCl_3$) δ 156.9, 156.8, 155.4, 155.3, 148.1, 148.0, 132.9, 132.9, 132.0, 131.9, 131.8, 131.7, 129.7, 129.4, 129.2, 129.1, 121.5, 121.5, 119.1, 119.0, 116.0, 115.8, 114.1, 57.5, 57.3, 55.6 (2C), 53.5 (2C), 47.9, 47.8, 44.0, 43.9, 28.4, 27.7, 26.1, 26.0, 25.0, 24.8, 21.9, 21.7 (2C), 21.6; IR (neat, cm^{-1}): 3321, 2934, 1152, 1242, 827, 749; ESI HRMS *m/z* $(M+H)^+$ calcd 446.2802, obsd 446.2811.

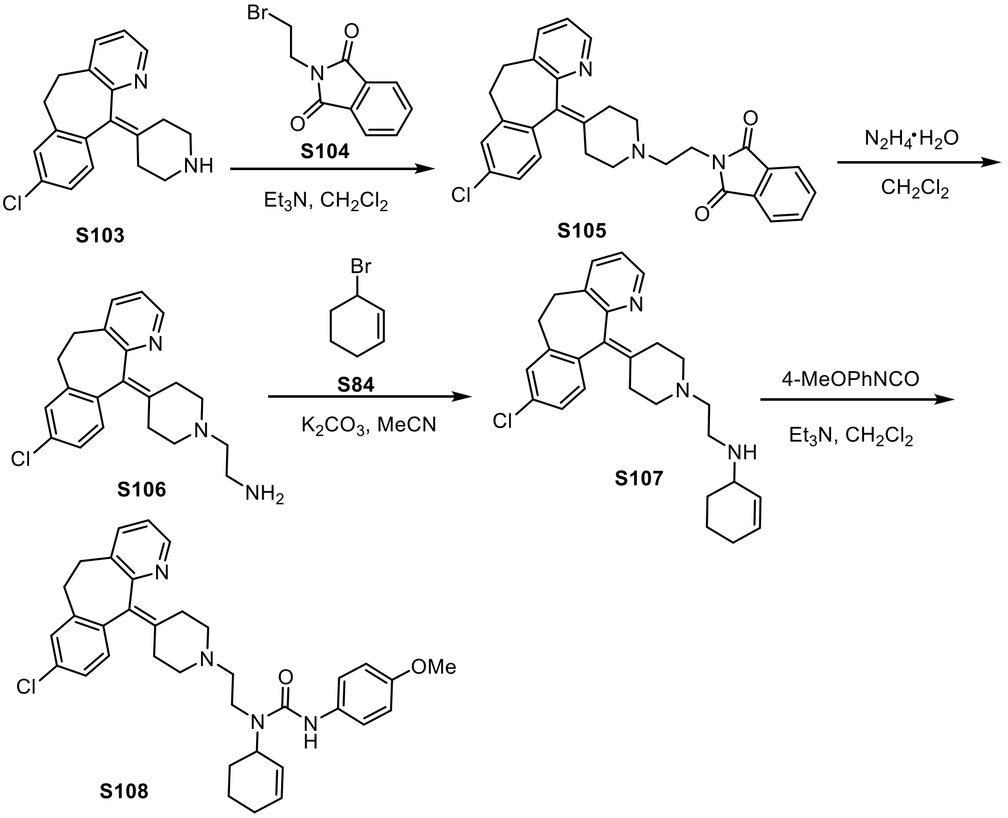

2-(2-(4-(8-Chloro-5,6-dihydro-11*H*-benzo[5,6]cyclohepta[1,2-*b*]pyridin-11-ylidene)piperidin-1-yl)ethyl)isoindoline-1,3-dione (S105). To a solution of **S103** (5.0 g, 16 mmol, 1.0 equiv) in CH_2Cl_2 (1 M), was added Et_3N (4.5 mL, 32 mmol, 2.0 equiv), followed by **S104** (4.1 g, 16 mmol, 1.0 equiv). The resulting reaction mixture was stirred for 12 h at rt. Solvent was removed under reduced pressure. The residue was chromatographed through silica gel eluting with EtOAc/hexanes to afford the product as a light yellow foamy solid in 45% yield (3.5 g) which was directly used without further purification.

2-(4-(8-Chloro-5,6-dihydro-11*H*-benzo[5,6]cyclohepta[1,2-*b*]pyridin-11-ylidene)piperidin-1-yl)ethan-1-amine (S106). To a solution of **S105** (3.5 g, 7.2 mmol, 1.0 equiv) in CH_2Cl_2 (0.1 M) were added hydrazine monohydrate (1.3 mL, 22 mmol, 3.0 equiv). After 9 h, the mixture was filtered through Celite and concentrated in vacuo. The product was directly used without further purification.

1-(2-(4-(8-Chloro-5,6-dihydro-11*H*-benzo[5,6]cyclohepta[1,2-*b*]pyridin-11-ylidene)piperidin-1-yl)ethyl)-1-(cyclohex-2-en-1-yl)-3-(4-methoxyphenyl)urea (S108). The title compound (1.6 g, 64% yield in two steps) was prepared starting from **S106** (1.5 g, 4.3 mmol) by following the GP 4 and isolated as as a white foamy and a 1:1 mixture of diastereomers. ^{1}H NMR (500 MHz, $CDCl_3$) δ 10.00 (brs, 1H), 8.39 (dd, *J* = 4.8, 1.7 Hz, 1H), 7.43 (dd, *J* = 7.7, 1.7 Hz, 1H), 7.40–7.33 (m, 2H), 7.16–7.13 (m, 1H), 7.12–7.10 (m, 2H), 7.08 (dd, *J* = 7.7, 4.8 Hz, 1H), 6.89–6.83 (m, 2H), 5.92–5.82 (m, 1H), 5.54–5.41 (m, 1H), 5.01–4.90 (m, 1H), 3.79 (s, 3H), 3.45–3.21 (m, 4H), 2.95–2.75 (m, 4H), 2.63–2.53 (m, 3H), 2.48–2.36 (m, 3H), 2.33–2.24 (m, 2H), 2.01–1.94 (m, 3H), 1.80–1.74 (m, 1H), 1.71–1.58 (m, 1H), 1.44–1.35 (m, 1H); ^{13}C NMR (126 MHz, $CDCl_3$) δ 157.9, 157.2, 155.2, 146.7, 139.6, 137.6 (2C), 137.5, 133.6 (2C), 133.4, 132.9, 131.4, 130.7, 129.6 (2C), 129.0, 126.1, 122.3, 122.0, 114.2, 61.5, 55.9 (2C), 55.8 (2C), 55.5, 52.5, 42.2, 31.8, 31.5, 30.8, 30.5, 28.0 (2C), 24.7, 21.7; IR (neat, cm^{-1}): 3439, 1654, 1511, 1238, 827; ESI HRMS *m/z* $(M+H)^+$ calcd 583.2834, obsd 583.2842.

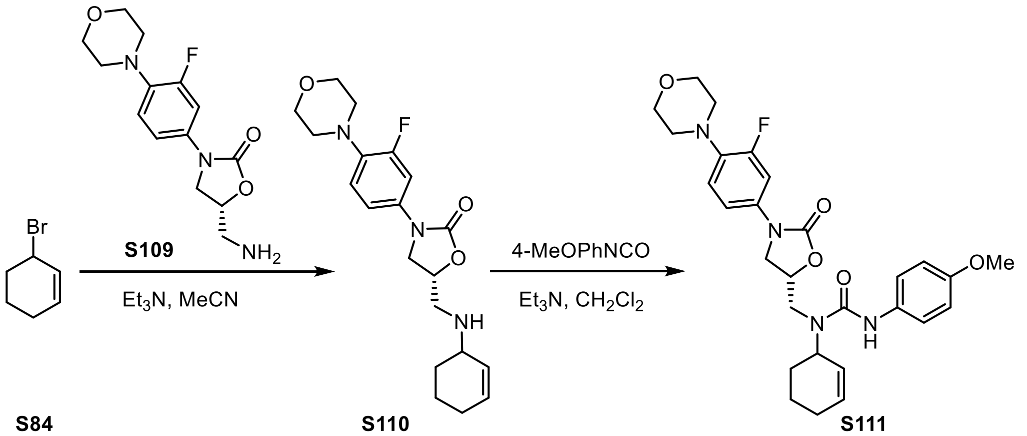

1-(Cyclohex-2-en-1-yl)-1-(((*S*)-3-(3-fluoro-4-morpholinophenyl)-2-oxooxazolidin-5-yl)methyl)-3-(4-methoxyphenyl)urea (S111). The title compound was obtained as a red foamy solid (1.1 g, 21% yield in two steps) and a 5.7:1 mixture of diastereomers starting from **S109** (3.0 g, 10 mmol, 1.0 equiv) by following the procedure described for the synthesis of **S99**. ^{1}H NMR (500 MHz, $CDCl_3$) δ 7.48–7.42 (m, 1H), 7.25–7.22 (m, 0.3H), 7.22–7.18 (m, 1.7H), 7.12–7.05 (m, 2H), 6.94–6.86 (m, 1H), 6.85–6.79 (m, 2H), 6.16–6.04 (m, 1H), 5.86 (d, *J* = 10.4 Hz, 0.2H), 5.81 (dt, *J* = 10.1, 1.9 Hz, 0.8H), 4.91–4.79 (m, 1H), 4.34–4.19 (m, 1H), 4.04–3.96 (m, 1.2H), 3.92–3.82 (m, 5.8H), 3.78–3.75 (m, 3H), 3.63 (dd, *J* = 15.2, 3.0 Hz, 0.8H), 3.38 (dd, *J* = 15.0, 7.1 Hz, 0.2H), 3.11–2.97 (m, 4H), 2.18–2.03 (m, 3H), 1.91–1.76 (m, 2H), 1.71–1.59 (m, 1H); Only the major isomer was shown. ^{13}C NMR (126 MHz, $CDCl_3$) δ 156.4, 156.0, 155.5 (d, $J_{C\text{-}F}$ =

246.2 Hz), 154.5, 136.4 (d, $J_{C\text{-}F}$ = 9.0 Hz), 133.3 (d, $J_{C\text{-}F}$ = 10.3 Hz), 133.2, 131.9, 129.4, 121.9, 118.9 (d, $J_{C\text{-}F}$ = 4.1 Hz), 114.2, 114.0 (d, $J_{C\text{-}F}$ = 3.2 Hz), 107.6 (d, $J_{C\text{-}F}$ = 26.2 Hz), 73.5, 67.0, 57.3, 55.6, 51.1 (2C), 50.3, 48.1, 27.0, 24.6, 21.7; ^{19}F NMR (471 MHz, $CDCl_3$) δ −120.3, −120.4; IR (neat, cm^{-1}): 2954, 1752, 1515, 1240, 1116, 751; ESI HRMS *m/z* $(M+Na)^+$ calcd 547.2327, obsd 547.2335.

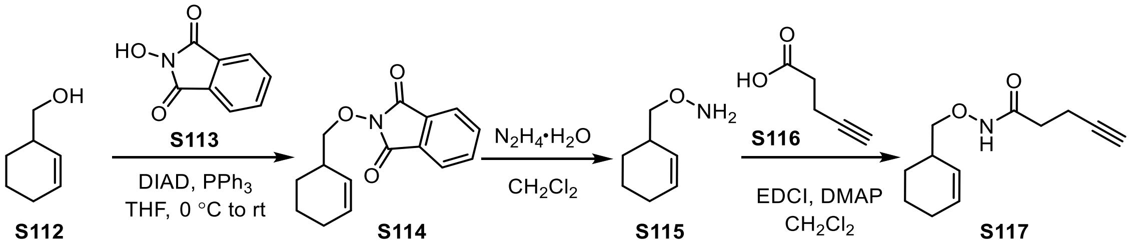

2-(Cyclohex-2-en-1-ylmethoxy)isoindoline-1,3-dione (S114). Diisopropyl azodicarboxylate (DIAD, 3.5 mL, 18 mmol, 1.2 equiv) was added dropwise to a solution of **S112** (1.7 g, 15 mmol, 1.0 equiv), **S113** (2.9 g, 18 mmol, 1.2 equiv) and PPh_3 (4.7 g, 18 mmol, 1.2 equiv) in THF (50 mL) at 0 °C. The reaction mixture was stirred at rt for 5 h and then concentrated under reduced pressure. The residue was chromatographed through silica gel eluting with EtOAc/hexanes to give the title compound as a white solid (3.0 g, 78%). ^{1}H NMR (500 MHz, $CDCl_3$) δ 7.85–7.81 (m, 2H), 7.78–7.73 (m, 2H), 5.84 (dtd, *J* = 9.9, 3.7, 2.3 Hz, 1H), 5.75–5.69 (m, 1H), 4.08 (d, *J* = 6.9 Hz, 2H), 2.68–2.55 (m, 1H), 2.08–1.97 (m, 2H), 1.97–1.88 (m, 1H), 1.80–1.72 (m, 1H), 1.67–1.52 (m, 2H); ^{13}C NMR (126 MHz, $CDCl_3$) δ 163.6, 134.5, 129.9, 129.1, 126.5, 123.6, 82.0, 34.7, 25.5, 25.2, 20.6; IR (neat, cm^{-1}): 3417, 2932, 1788, 1729, 1187, 993, 877; ESI HRMS *m/z* $(M+Na)^+$ calcd 280.0944, obsd 280.0955.

***O*-(Cyclohex-2-en-1-ylmethyl)hydroxylamine (S115).** To a solution of **S114** (3.0 g, 12 mmol, 1.0 equiv) in CH_2Cl_2 (0.1 M) were added hydrazine monohydrate (1.6 mL, 27 mmol, 2.3 equiv). After 2 h, the mixture was filtered through Celite and concentrated in vacuo. The product was directly used without further purification.

***N*-(Cyclohex-2-en-1-ylmethoxy)pent-4-ynamide (S117).** The title compound was obtained as a colorless oil (0.24 g, 59%) and a 4:1 mixture of rotary isomers starting from **S116** (0.20 g, 2.0 mmol, 1.0 equiv) by following the procedure described for the synthesis of **S52**. ^{1}H NMR (500 MHz, $CDCl_3$) δ 9.75 (brs, 0.8H), 8.78 (brs, 0.2H), 5.88–5.71 (m, 1H), 5.69–5.49 (m, 1H), 3.87–3.70 (m, 2H), 2.76–2.61 (m, 0.4H), 2.58–2.45 (m, 3H), 2.43–2.28 (m, 1.6H), 2.04–1.94 (m, 3H), 1.86–1.77 (m, 1H), 1.76–1.65 (m, 1H), 1.62–1.48 (m, 1H), 1.46–1.36 (m, 1H); ^{13}C NMR (126 MHz, $CDCl_3$) δ 175.3, 169.1, 129.8, 129.2, 127.2, 126.6, 82.6, 81.5, 80.5, 69.6, 69.0, 34.4, 32.2, 30.7, 29.7, 25.7, 25.2, 20.7, 14.8, 13.4; IR (neat, cm^{-1}): 3292, 2930, 1660, 1435, 1072, 637; ESI HRMS *m/z* $(M+Na)^+$ calcd 230.1151, obsd 230.1160.

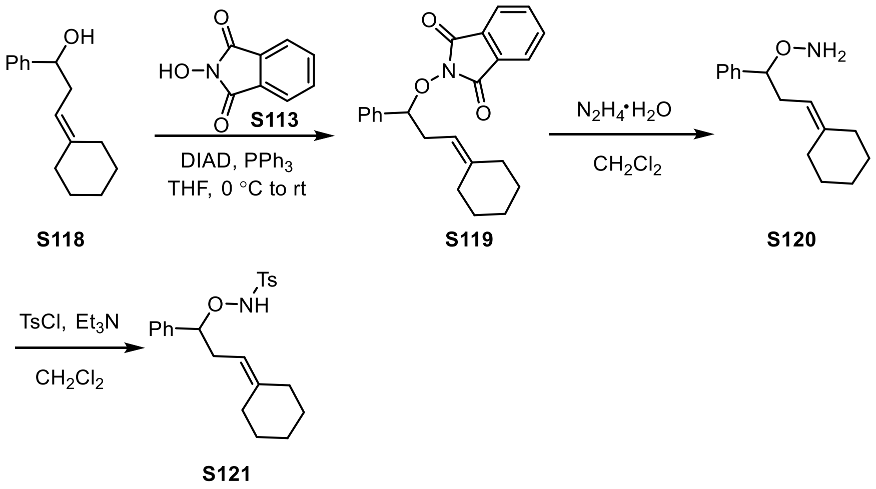

***O*-(3-Cyclohexylidene-1-phenylpropyl)hydroxylamine (S120).** The title compound was obtained as a light-yellow oil (0.44 g, 83% yield in two steps) starting from **S118**[22] (0.50 g, 2.3 mmol, 1.0 equiv) by following the procedure described for the synthesis of **S115**. The product was directly used in the following step without further purification.

***N*-(3-Cyclohexylidene-1-phenylpropoxy)-4-methylbenzenesulfonamide (S121).** The hydroxylamine **S120** was dissolved in CH_2Cl_2 (10 mL) and treated with pyridine (0.46 mL, 5.7 mmol, 3.0 equiv) and *p*-TsCl (0.55 g, 2.9 mmol, 1.5 equiv). The reaction mixture was stirred at rt until complete consumption of starting material (monitored by TLC). The mixture was quenched with H_2O and extracted with CH_2Cl_2. The combined organic solution was washed with HCl (1 N, 20 mL) and brine (20 mL), dried over Na_2SO_4 and concentrated in vacuo. The residue was chromatographed through silica gel to afford **S121** as a white solid in 42% yield (0.31 g). ^{1}H NMR (500 MHz, $CDCl_3$) δ 7.84–7.76 (m, 2H), 7.34–7.30 (m, 3H), 7.30–7.26 (m, 2H), 7.25–7.21 (m, 2H), 6.72–6.64 (m, 1H), 5.06–4.99 (m, 1H), 4.92 (t, *J* = 7.0 Hz, 1H), 2.57 (ddd, *J* = 14.7, 7.3, 7.3 Hz, 1H), 2.45 (s, 3H), 2.37 (ddd, *J* = 14.3, 7.0, 7.0 Hz, 1H), 2.10–2.02 (m, 2H), 2.02–1.91 (m, 2H), 1.54–1.35 (m, 5H), 1.32–1.22 (m, 1H); ^{13}C NMR (126 MHz, $CDCl_3$) δ 144.9, 142.5, 140.2, 134.1, 129.8, 128.9, 128.5, 128.3, 127.4, 115.9, 89.0, 37.3, 33.3, 29.0, 28.7, 27.6, 27.0, 21.9; IR (neat, cm^{-1}): 3220, 2926, 1340, 1167, 813; ESI HRMS *m/z* $(M+Na)^+$ calcd 408.1604, obsd 408.1610.

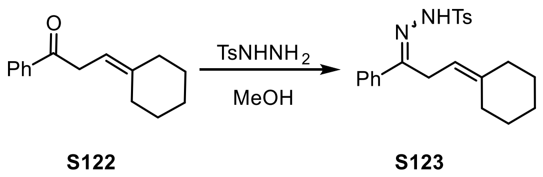

***N'*-(3-Cyclohexylidene-1-phenylpropylidene)-4-methylbenzenesulfonohydrazide (S123).** To a stirred solution of 4-methylbenzenesulfonohydrazide (0.64 g, 3.4 mmol, 1.5 equiv) in MeOH (4 mL), **S122** (0.49 g, 2.3 mmol, 1.0 equiv) was added at rt. The mixture was stirred until the reaction was completed as monitored by TLC. After removal of the solvent under vacuum, the residue was purified by flash column chromatography on silica gel to give the title

compound (0.56 g, 63% yield). Light yellow solid; *Z:E* = 1:9; ^{1}H NMR (500 MHz, $CDCl_3$) δ 7.96 (brs, 0.9H), 7.88–7.82 (m, 1.8H), 7.82–7.76 (m, 0.2H), 7.65–7.60 (m, 1.8H), 7.41–7.37 (m, 0.3H), 7.35–7.31 (m, 2.8H), 7.31–7.26 (m, 2H), 7.05–7.00 (m, 0.2H), 4.97 (t, *J* = 7.5 Hz, 0.1H), 4.82 (t, *J* = 6.9 Hz, 0.9H), 3.34 (d, *J* = 6.9 Hz, 1.8H), 3.17 (d, *J* = 7.4 Hz, 0.2H), 2.43 (s, 0.3H), 2.39 (s, 2.7H), 2.29–2.23 (m, 1.8H), 2.09–2.02 (m, 1.8H), 2.01–1.95 (m, 0.2H), 1.91–1.86 (m, 0.2H), 1.63–1.49 (m, 5.7H), 1.46–1.38 (m, 0.3H); ^{13}C NMR (126 MHz, $CDCl_3$) δ 154.9, 145.5, 144.1, 137.0, 135.7, 129.7, 129.6, 129.5, 128.5, 128.1 (2C), 127.1, 126.6, 112.6, 37.1, 29.0, 28.8, 28.5, 27.4, 27.3, 27.0, 26.8, 26.6, 21.7; IR (neat, cm^{-1}): 3423, 2932, 1168, 692, 569; ESI HRMS *m/z* $(M+Na)^+$ calcd 405.1607, obsd 405.1620.

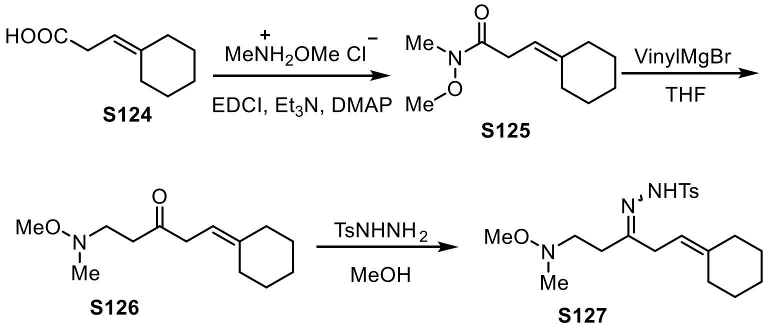

3-Cyclohexylidene-*N*-methoxy-*N*-methylpropanamide (**S125**). To a solution of **S124** (2.3 g, 15 mmol, 1.0 equiv) in CH_2Cl_2 (20 mL) was added Et_3N (3.4 mL, 24 mmol, 1.6 equiv) and EDCI (5.2 g, 27 mmol, 1.8 equiv), followed by *N,O*-dimethylhydroxylamine hydrochloride (2.3 g, 24 mmol, 1.6 equiv) and DMAP (0.20 g, 1.5 mmol, 0.10 equiv). The resulting reaction mixture was stirred at rt for 12 h, diluted with EtOAc, and quenched with HCl (1 N, 15 mL). The layers were separated, and the aqueous layer was extracted twice with EtOAc. The combined organic solution was washed with brine, dried over anhydrous $MgSO_4$, and concentrated under reduced pressure. The residue was chromatographed through silica gel to afford **S125** as a colorless oil in 52% yield (1.5 g).

1-Cyclohexylidene-5-(methoxy(methyl)amino)pentan-3-one (S126). To a solution of vinylmagnesium bromide in THF (1.0 M, 4.1 mL, 4.1 mmol, 1.2 equiv) at 0 °C was added **S125** (0.68 g, 3.4 mmol, 1.0 equiv) dropwise over 10 min. The reaction mixture was stirred at 0 °C until completion (monitored by TLC). H_2O was added to quench the reaction. The resulting mixture was extracted with EtOAc. The combined organic solution was washed with brine, dried over anhydrous $MgSO_4$, filtered, and concentrated under reduced pressure to afford the title compound (0.67 g, 87% yield). The crude product was used in the following step without purification.

***N'*-(1-Cyclohexylidene-5-(methoxy(methyl)amino)pentan-3-ylidene)-4-methylbenzenesulfonohydrazide (S127).** The title compound was obtained as a light-yellow oil (1.0 g, 85%) and a 1:2.5 mixture of *Z*/*E* isomer starting from **S126** (0.68 g, 3.0 mmol, 1.0 equiv) by following the

procedure described for the synthesis of **S123**.; ^{1}H NMR (500 MHz, $CDCl_3$) δ 10.67 (brs, 0.7H), 7.85–7.80 (m, 1.4H), 7.80–7.76 (m, 0.6H), 7.66 (brs, 0.3H), 7.32–7.26 (m, 2H), 5.01 (tt, *J* = 7.6, 1.2 Hz, 0.7H), 4.82 (tt, *J* = 7.2, 1.3 Hz, 0.3H), 3.63 (s, 2.1H), 3.34 (s, 0.9H), 2.92–2.59 (m, 4H), 2.56–2.30 (m, 8H), 2.21–1.99 (m, 4H), 1.60–1.34 (m, 6H); ^{13}C NMR (126 MHz, $CDCl_3$) δ 159.1, 158.6, 145.4, 144.0, 143.8, 143.4, 136.7, 135.7, 129.5, 129.4, 128.2, 128.0, 115.1, 112.4, 59.9, 59.8, 58.8, 56.9, 45.2, 44.3, 37.3, 37.2, 36.8, 35.6, 29.4, 28.9, 28.8, 28.7, 28.5, 28.3, 27.8, 27.3, 26.8, 26.7, 21.7 (2C); IR (neat, cm^{-1}): 3447, 2932, 1636, 1167, 1036, 686, 569; ESI HRMS *m/z* $(M+H)^+$ calcd 394.2159, obsd 394.2174.

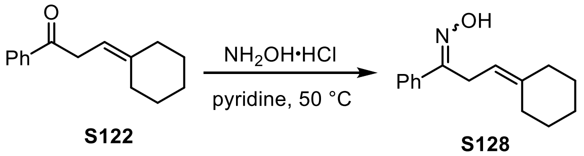

S122 S128

3-Cyclohexylidene-1-phenylpropan-1-one oxime (S128). To a solution of **S122** (0.43 g, 2.0 mmol, 1.0 equiv) in pyridine (3 mL) was added hydroxylamine hydrochloride (0.70 g, 10 mmol, 5.0 equiv). The reaction mixture was stirred at 50 °C for 3 h, diluted with EtOAc, and quenched with HCl (1 N, 15 mL). The layers were separated, and the aqueous layer was extracted twice with EtOAc. The combined organic solution was washed with brine, dried over anhydrous $MgSO_4$, and concentrated under reduced pressure. The residue was chromatographed through silica gel to afford **S128** as a white solid in 78% yield (0.32 g). ^{1}H NMR (500 MHz, $CDCl_3$) δ 8.92 (brs, 1H), 7.64–7.57 (m, 2H), 7.40–7.33 (m, 3H), 5.12 (tt, *J* = 7.2, 1.3 Hz, 1H), 3.55 (d, *J* = 7.1 Hz, 2H), 2.29–2.21 (m, 2H), 2.09–1.99 (m, 2H), 1.59–1.42 (m, 6H); ^{13}C NMR (126 MHz, $CDCl_3$) δ 158.7, 142.3, 136.1, 129.3, 128.6, 126.7, 114.9, 37.2, 29.1, 28.6, 27.7, 27.0, 25.3; IR (neat, cm^{-1}): 3352, 2931, 1447, 915, 762; ESI HRMS *m/z* $(M+H)^+$ calcd 230.1539, obsd 230.1547.

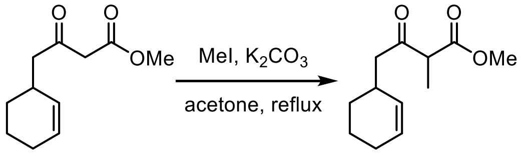

S129 S130

Methyl 4-(cyclohex-2-en-1-yl)-2-methyl-3-oxobutanoate (S130). The title compound was prepared from the known compound **S130**[23] as a pale-yellow oil (59% yield) by following the GP 5. The compound was obtained as a 1:1 mixture of diastereomers and contained traces of enol form; Pale yellow oil; ^{1}H NMR (500 MHz, $CDCl_3$) δ 5.72–5.67 (m, 2H), 5.50–5.44 (m, 2H), 3.73 (2s, 6H), 3.53 (2q, *J* = 7.2 Hz, 2H), 2.71–2.64 (m, 2H), 2.59–2.53 (m, 2H), 2.49 (dd, *J* = 17.0, 7.2 Hz, 1H), 2.48 (dd, *J* = 17.0, 7.6 Hz, 1H), 1.99–1.94 (m, 4H), 1.83–1.76 (m, 2H), 1.71–1.63 (m, 2H), 1.60–1.51 (m, 2H), 1.34 (2d, *J* = 7.2 Hz, 6H), 1.23–1.16 (m, 2H); ^{13}C NMR (126 MHz, $CDCl_3$) δ 205.1, 205.0, 171.0 (2C), 130.3 (2C), 128.2(2C), 53.2, 53.1, 52.4, 47.7, 47.6, 30.9, 30.8, 28.9, 28.8, 25.1 (2C), 21.1, 21.0, 12.8 (2C); IR (neat, cm^{-1}): 3428, 2928,

1748, 1716, 1452, 1204, 724; ESI HRMS *m/z* $(M+Na)^+$ calcd 233.1148, obsd 233.1138.

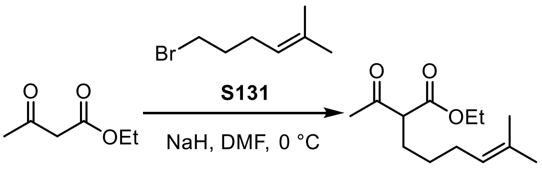

S132

Ethyl 2-acetyl-7-methyloct-6-enoate (S132). To a suspension of NaH (60% dispersion in mineral oil, 0.32 g, 8.0 mmol, 1.0 equiv) in DMF (25 mL) was added ethyl acetoacetate (0.98 mL, 8.0 mmol, 1.0 equiv) dropwise at 0 °C. The mixture was stirred for 10 min and the alkenyl bromide **S131**[24] (0.71 g, 4.0 mmol, 1.0 equiv) was added dropwise using a syringe. The mixture was warmed to rt and stirred for 12 h. H_2O (60 mL) was added to quench the reaction. The mixture was extracted twice with Et_2O. The combined organic solution was washed with brine, dried over anhydrous Na_2SO_4, filtered, and concentrated under reduced pressure. The residue was purified by flash column chromatography on silica gel to afford **S132** as a colorless oil (0.28 g, 30% yield, < 10% of enol form). ^{1}H NMR (400 MHz, $CDCl_3$) δ 5.16–5.03 (m, 1.1H), 4.24–4.15 (m, 2.2H), 3.40 (t, *J* = 7.4 Hz, 1H), 2.22 (s, 3H), 2.03–1.96 (m, 2.5H), 1.92–1.76 (m, 2.2H), 1.68 (2s, 3.3H), 1.59 (2s, 3.3H), 1.42–1.24 (m, 5.5H); ^{13}C NMR (101 MHz, $CDCl_3$) (major form) δ 203.5, 170.0, 132.3, 123.9, 61.4, 60.0, 28.9, 28.0, 27.8, 27.7, 25.8, 17.8, 14.3; IR (neat, cm^{-1}): 3444, 2980, 2953, 1738, 1715, 1447, 1368, 1151, 1026; ESI HRMS *m/z* $(M+Na)^+$ calcd 249.1461, obsd 249.1463.

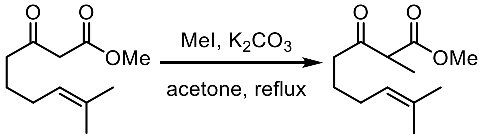

S133 **S134**

Methyl 2,8-dimethyl-3-oxonon-7-enoate (S134). The title compound was prepared as a colorless oil (37% yield) from the known compound **S133**[25] by following the GP 5. The compound contained traces of enol form. ^{1}H NMR (400 MHz, $CDCl_3$) δ 5.07 (t, *J* = 7.0 Hz, 1H), 3.73 (s, 3H), 3.53 (q, *J* = 7.1 Hz, 1H), 2.61–2.44 (m, 2H), 1.98 (q, *J* = 7.3 Hz, 2H), 1.69 (s, 3H), 1.67–1.58 (m, 2H), 1.59 (s, 3H), 1.33 (d, *J* = 7.1 Hz, 3H); ^{13}C NMR (101 MHz, $CDCl_3$) δ 206.0, 171.2, 132.6, 123.7, 52.8, 52.5, 40.9, 27.3, 25.8, 23.8, 17.8, 13.0; IR (neat, cm^{-1}): 2923, 1751, 1732, 1435, 1238, 1084; ESI HRMS *m/z* $(M+Na)^+$ calcd 235.1305, obsd 235.1306.

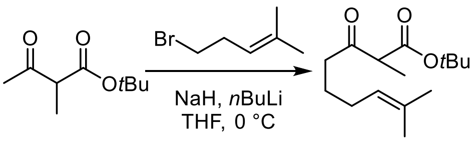

S135 **S136**

***tert*-Butyl 2,8-dimethyl-3-oxonon-7-enoate (S136).** The title compound was prepared as a

colorless oil (32% yield) from commercially available **S135** and 5-bromo-2-methylpent-2-ene by following the GP 6. ^{1}H NMR (400 MHz, $CDCl_3$) δ 5.11–5.04 (m, 1H), 3.41 (q, *J* = 7.1 Hz, 1H), 2.63–2.41 (m, 2H), 1.99 (q, *J* = 7.1 Hz, 2H), 1.68 (s, 3H), 1.67–1.59 (m, 2H), 1.59 (s, 3H), 1.45 (s, 9H), 1.27 (d, *J* = 7.1 Hz, 3H); ^{13}C NMR (101 MHz, $CDCl_3$) δ 206.5, 170.0, 132.5, 123.8, 81.7, 54.0, 40.8, 28.0, 27.4, 25.8, 23.9, 17.8, 12.8; IR (neat, cm^{-1}): 2979, 2935, 1740, 1715, 1456, 1369, 1555, 846; ESI HRMS *m/z* (M+Na)$^+$ calcd 277.1774, obsd 277.1776.

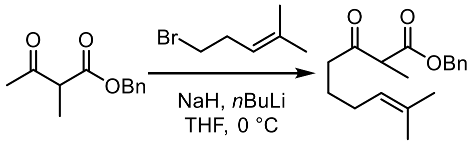

S137 **S138**

Benzyl 2,8-dimethyl-3-oxonon-7-enoate (S138). The title compound was prepared as a colorless oil (18% yield) from commercially available **S137** and 5-bromo-2-methylpent-2-ene by following the GP 6. ^{1}H NMR (400 MHz, $CDCl_3$) δ 7.38–7.31 (m, 5H), 5.16 (s, 2H), 5.07–4.99 (m, 1H), 3.55 (q, *J* = 7.2 Hz, 1H), 2.51 (dt, *J* = 15.7, 6.5 Hz, 1H), 2.43 (dt, *J* = 15.7, 6.5 Hz, 1H), 1.92 (q, *J* = 7.4 Hz, 2H), 1.67 (s, 3H), 1.60–1.55 (m, 2H), 1.56 (s, 3H), 1.34 (d, *J* = 7.2 Hz, 3H); ^{13}C NMR (101 MHz, $CDCl_3$) δ 205.8, 170.6, 135.6, 132.6, 128.7, 128.5, 128.4, 123.7, 67.1, 53.0, 41.0, 27.3, 25.8, 23.8, 17.8, 13.0. IR (neat, cm^{-1}): 2938, 1746, 1736, 1455, 1377, 1184, 698; ESI HRMS *m/z* (M+Na)$^+$ calcd 311.1618, obsd 311.1611.

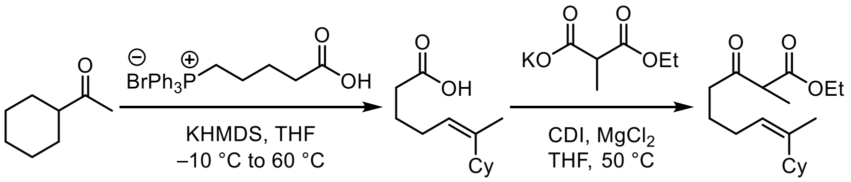

S139 **S140**

Ethyl (*Z*)-8-cyclohexyl-2-methyl-3-oxonon-7-enoate (S140). The title compound was prepared as a colorless oil (57% yield in two steps) from 1-cyclohexylethan-1-one by following the GP 7. *Z*/*E* = 1:0.6; Colorless oil; ^{1}H NMR (400 MHz, $CDCl_3$) δ 5.10–5.05 (m, 0.6H), 5.03–4.97 (m, 1H), 4.19 (2q, *J* = 7.2 Hz, 3.2H), 3.51 (2q, *J* = 7.2 Hz, 1.6H), 2.62–2.45 (m, 3.2H), 2.39–2.31 (m, 1H), 2.01 (p, *J* = 7.1 Hz, 3.2H), 1.88–1.72 (m, 4.8H), 1.71–1.59 (m, 10.2H), 1.56 (s, 1.6H), 1.48–1.43 (m, 1.6H), 1.36–1.24 (m, 16H); ^{13}C NMR (101 MHz, $CDCl_3$) δ 206.2, 206.1, 170.8, 141.8, 141.5, 123.2, 121.5, 61.4, 53.0, 47.5, 41.0, 39.9, 32.2, 31.3, 27.1, 26.9, 26.8, 26.6, 26.4, 24.2, 23.9, 19.8, 14.5, 14.2, 12.9; IR (neat, cm^{-1}): 2927, 2853, 1743, 1716, 1450, 1377, 1191; ESI HRMS *m/z* (M+Na)$^+$ calcd 317.2087, obsd 317.2089.

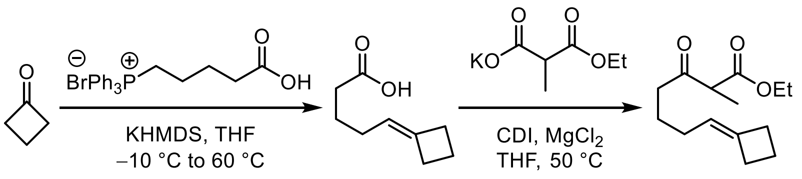

S141 **S142**

Ethyl 7-cyclobutylidene-2-methyl-3-oxoheptanoate (S142). The title compound was prepared as a colorless oil (52% yield in two steps) from cyclobutanone by following GP 7. The compound contained < 10% of enol form. ^{1}H NMR (400 MHz, $CDCl_3$) δ 5.03–4.96 (m, 1H), 4.19 (2q, J = 7.2 Hz, 2H), 3.51 (q, J = 7.1 Hz, 1H), 2.66–2.44 (m, 6H), 1.97–1.84 (m, 4H), 1.62 (p, J = 7.1 Hz, 2H), 1.33 (d, J = 7.1 Hz, 3H), 1.27 (t, J = 7.1 Hz, 3H); ^{13}C NMR (101 MHz, $CDCl_3$) δ 206.1, 170.8, 141.2, 119.4, 61.4, 53.0, 40.9, 31.0, 29.4, 27.3, 23.7, 17.2, 14.2, 12.9; IR (neat, cm^{-1}): 2982, 2942, 1740, 1716, 1456, 1372, 1192; ESI HRMS *m/z* $(M+Na)^+$ calcd 261.1461, obsd 261.1464.

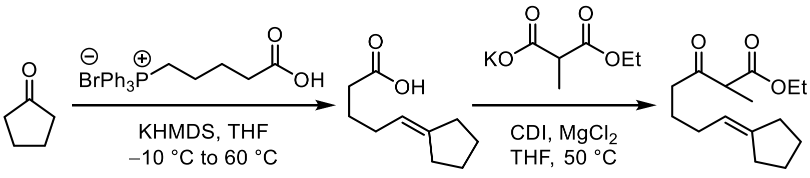

S143 **S144**

Ethyl 7-cycloheptylidene-2-methyl-3-oxoheptanoate (S144). The title compound was prepared as a colorless oil (33% yield in two steps) from cyclopentanone by following the GP 7. The compound contained traces of enol form. ^{1}H NMR (400 MHz, $CDCl_3$) δ 5.23–5.15 (m, 1H), 4.19 (q, J = 7.1 Hz, 2H), 3.51 (q, J = 7.1 Hz, 1H), 2.62–2.45 (m, 2H), 2.24–2.11 (m, 4H), 2.01–1.94 (m, 2H), 1.69–1.56 (m, 6H), 1.33 (d, J = 7.1 Hz, 3H), 1.27 (t, J = 7.1 Hz, 3H); ^{13}C NMR (101 MHz, $CDCl_3$) δ 206.2, 170.8, 144.4, 119.1, 61.4, 53.0, 41.0, 33.7, 29.0, 28.8, 26.5 (2C), 23.7, 14.2, 12.9; IR (neat, cm^{-1}): 2942, 1743, 1716, 1453, 1192, 1022; ESI HRMS *m/z* $(M+Na)^+$ calcd 275.1618, obsd 275.1619.

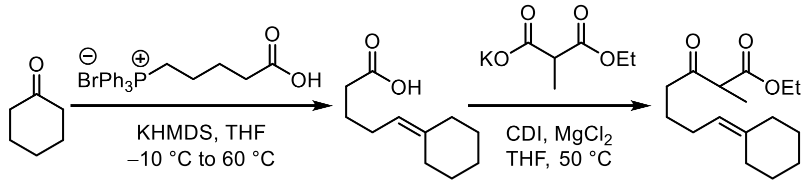

S145 **83**

Ethyl 7-cyclohexylidene-2-methyl-3-oxoheptanoate (83). The title compound was prepared as a colorless oil (57% yield in two steps) from cyclohexanone by following the GP 7. The compound contained traces of enol form. ^{1}H NMR (400 MHz, $CDCl_3$) δ 5.02 (t, J = 7.3 Hz, 1H), 4.18 (q, J = 7.1 Hz, 2H), 3.51 (q, J = 7.1 Hz, 1H), 2.61–2.44 (m, 2H), 2.13–2.03 (m, 4H), 1.99 (q, J = 7.3 Hz, 2H), 1.62 (p, J = 7.1 Hz, 2H), 1.57–1.43 (m, 6H), 1.33 (d, J = 7.1 Hz, 3H), 1.27 (t, J = 7.1 Hz, 3H); ^{13}C NMR (101 MHz, $CDCl_3$) δ 206.2, 170.8, 140.9, 120.3, 61.4, 53.0,

40.9, 37.3, 28.8, 28.0, 27.1, 26.4, 24.1, 14.2, 12.9; IR (neat, cm^{-1}): 2930, 2855, 1739, 1715, 1448, 1189; ESI HRMS *m/z* $(M+Na)^+$ calcd 289.1774, obsd 289.1776.

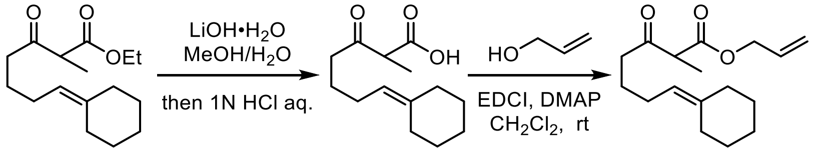

Allyl 7-cyclohexylidene-2-methyl-3-oxoheptanoate (S147). To a solution of **83** (0.58 g, 2.0 mmol, 1.0 equiv) in MeOH (1.5 mL) and H_2O (1.5 mL) was added LiOH•H_2O (0.63 g, 15 mmol, 7.5 equiv). The reaction mixture was stirred for 12 h, treated with H_2O (15 mL), and washed twice with Et_2O. The aqueous phase was acidified to pH 1 and extracted three times with CH_2Cl_2. The combined organic layer was dried over anhydrous $MgSO_4$, filtered and concentrated under reduced pressure to afford the crude carboxylic acid **S146**, which was then dissolved in anhydrous CH_2Cl_2 (8.0 mL). DMAP (39 mg, 0.32 mmol, 16 mol%), EDCI (0.61 g, 3.2 mmol, 1.6 equiv) and allyl alcohol (0.19 g, 3.2 mmol, 1.6 equiv) were added. The resulting mixture was stirred at rt for 3.5 h and diluted with CH_2Cl_2. The organic layer was washed twice with H_2O, dried over anhydrous Na_2SO_4, filtered, and concentrated under reduced pressure. The residue was purified by flash column chromatography on silica gel to afford **S147** as a colorless oil (0.27 g, 50% yield in two steps, < 10% of enol form). ^1H NMR (500 MHz, $CDCl_3$) δ 5.90 (ddt, *J* = 16.8, 10.4, 5.8 Hz, 1H), 5.32 (dq, *J* = 16.8, 1.6 Hz, 1H), 5.27–5.23 (m, 1H), 5.02 (t, *J* = 7.4 Hz, 1H), 4.62 (d, *J* = 5.8 Hz, 2H), 3.55 (q, *J* = 7.1 Hz, 1H), 2.57 (dt, *J* = 17.4, 7.3 Hz, 1H), 2.49 (dt, *J* = 17.4, 7.3 Hz, 1H), 2.11–2.03 (m, 4H), 1.99 (q, *J* = 7.3 Hz, 2H), 1.62 (p, *J* = 7.1 Hz, 2H), 1.56–1.46 (m, 6H), 1.35 (d, *J* = 7.1 Hz, 3H); ^{13}C NMR (126 MHz, $CDCl_3$) δ 206.0, 170.4, 140.9, 131.7, 120.3, 118.9, 66.0, 52.9, 40.9, 37.3, 28.8, 28.0, 27.0, 26.4, 24.1, 13.0; IR (neat, cm^{-1}): 2928, 2854, 1744, 1717, 1449, 1187, ; ESI HRMS *m/z* $(M+Na)^+$ calcd 301.1774, obsd 301.1776.

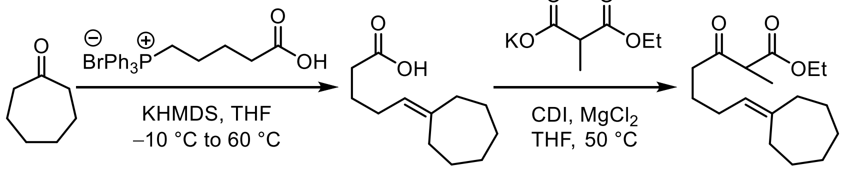

Ethyl 7-cyclopentylidene-2-methyl-3-oxoheptanoate (S149). The title compound was prepared as a colorless oil (66% yield in two steps) from cycloheptanone by following the GP 7. The compound contained traces of enol form. ^1H NMR (400 MHz, $CDCl_3$) δ 5.10–5.05 (m, 1H), 4.18 (2q, *J* = 7.1 Hz, 2H), 3.51 (q, *J* = 7.1 Hz, 1H), 2.63–2.44 (m, 2H), 2.22–2.16 (m, 4H), 1.97 (q, *J* = 7.2 Hz, 2H), 1.68–1.60 (m, 2H), 1.58–1.47 (m, 8H), 1.33 (d, *J* = 7.1 Hz, 3H), 1.27 (t, *J* = 7.1 Hz, 3H); ^{13}C NMR (101 MHz, $CDCl_3$) δ 206.2, 170.8, 142.4, 124.0, 61.4, 53.0, 41.0, 38.0, 30.2, 30.1, 29.5, 29.2, 27.3, 27.0, 23.8, 14.2, 12.9; IR (neat, cm^{-1}): 3450, 2925,

2853, 1740, 1716, 1455, 1376, 1190; ESI HRMS *m/z* $(M+Na)^+$ calcd 303.1931, obsd 303.1932.

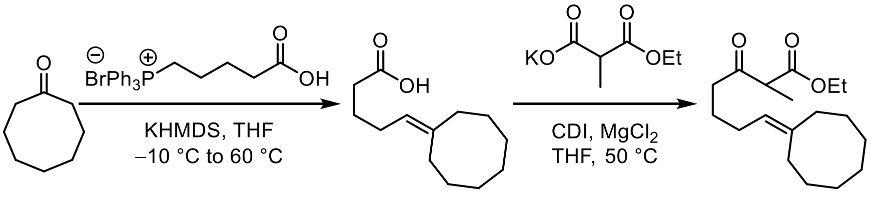

S150 **S151**

Ethyl 7-cyclooctylidene-2-methyl-3-oxoheptanoate (S151). The title compound was prepared as a colorless oil (55% yield in two steps) from cyclooctanone by following the GP 7. The compound contained < 10% of enol form. ^{1}H NMR (400 MHz, $CDCl_3$) δ 5.11 (t, *J* = 7.2 Hz, 1H), 4.19 (q, *J* = 7.2 Hz, 2H), 3.51 (q, *J* = 7.2 Hz, 1H), 2.64–2.45 (m, 2H), 2.18–2.10 (m, 4H), 2.01 (q, *J* = 7.3 Hz, 2H), 1.69–1.56 (m, 6H), 1.51–1.46 (m, 6H), 1.33 (2d, *J* = 7.2 Hz, 3H), 1.27 (2t, *J* = 7.2 Hz, 3H); ^{13}C NMR (101 MHz, $CDCl_3$) δ 206.1, 170.8, 142.1, 124.4, 61.4, 53.0, 41.1, 37.9, 29.3, 27.4, 27.2 (2C), 26.5, 26.4, 26.3, 23.8, 14.2, 12.9; IR (neat, cm^{-1}): 3445, 2927, 2855, 1739, 1715, 1455, 1375, 1189; ESI HRMS *m/z* $(M+Na)^+$ calcd 317.2087, obsd 317.2089.

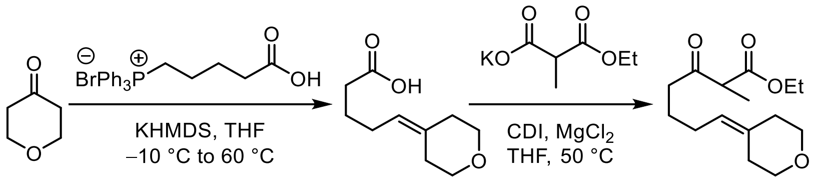

S152 **S153**

Ethyl 2-methyl-3-oxo-7-(tetrahydro-4*H*-pyran-4-ylidene)heptanoate (S153). The title compound was prepared as a colorless oil (67% yield in two steps) from tetrahydro-4*H*-pyran-4-one by following the GP 7. The compound contained traces of enol form. ^{1}H NMR (400 MHz, $CDCl_3$) δ 5.15 (t, *J* = 7.3 Hz, 1H), 4.19 (q, *J* = 7.2 Hz, 2H), 3.65 (2t, *J* = 5.3 Hz, 4H), 3.51 (q, *J* = 7.1 Hz, 1H), 2.58 (dt, *J* = 17.4, 7.3 Hz, 1H), 2.50 (dt, *J* = 17.4, 7.3 Hz, 1H), 2.25 (t, *J* = 5.3 Hz, 2H), 2.20 (t, *J* = 5.3 Hz, 2H), 2.01 (q, *J* = 7.3 Hz, 2H), 1.64 (p, *J* = 7.3 Hz, 2H), 1.33 (d, *J* = 7.1 Hz, 3H), 1.27 (t, *J* = 7.2 Hz, 3H); ^{13}C NMR (101 MHz, $CDCl_3$) δ 205.9, 170.7, 135.4, 122.4, 69.8, 68.9, 61.4, 53.0, 40.7, 37.1, 29.8, 26.2, 23.9, 14.2, 12.9; IR (neat, cm^{-1}): 2954, 2849, 1741, 1715, 1455, 1374, 1191, 1100, 852; ESI HRMS *m/z* $(M+Na)^+$ calcd 291.1567, obsd 291.1567.

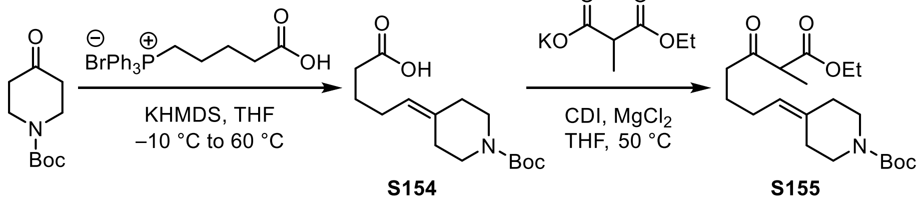

S154 **S155**

***tert*-Butyl 4-(7-ethoxy-6-methyl-5,7-dioxoheptylidene)piperidine-1-carboxylate (S155).**

The title compound was prepared as a colorless oil (65% in two steps) from *tert*-butyl 4-oxopiperidine-1-carboxylate by following the GP 7. ^{1}H NMR (500 MHz, $CDCl_3$) δ 5.18 (t, *J* = 7.5 Hz, 1H), 4.18 (q, *J* = 7.2 Hz, 2H), 3.50 (q, *J* = 7.1 Hz, 1H), 3.38 (q, *J* = 6.1 Hz, 4H), 2.58 (dt, *J* = 17.5, 7.3 Hz, 1H), 2.49 (dt, *J* = 17.4, 7.3 Hz, 1H), 2.18 (t, *J* = 5.9 Hz, 2H), 2.12 (t, *J* = 5.9 Hz, 2H), 2.02 (q, *J* = 7.3 Hz, 2H), 1.64 (p, *J* = 7.3 Hz, 2H), 1.47 (s, 9H), 1.33 (d, *J* = 7.1 Hz, 3H), 1.27 (t, *J* = 7.2 Hz, 3H); ^{13}C NMR (126 MHz, $CDCl_3$) δ 205.9, 170.7, 154.9, 136.2, 123.1, 79.5, 61.4, 53.0, 40.7, 36.0, 28.6, 28.4, 26.4, 23.9, 14.2, 12.9; IR (neat, cm^{-1}): 2978, 2939, 1744, 1698, 1420, 1366, 1236, 1170, 1116, 768; ESI HRMS *m/z* $(M+Na)^+$ calcd 390.2251, obsd 390.2256.

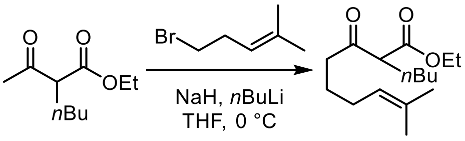

S156

Ethyl 2-butyl-8-methyl-3-oxonon-7-enoate (S156). The title compound was prepared as a yellow oil (50% yield) from ethyl 2-acetylhexanoate by following the GP 6. The compound contained < 10% of enol form. ^{1}H NMR (500 MHz, $CDCl_3$) δ 5.10–5.04 (m, 1H), 4.18 (2q, *J* = 7.1 Hz, 2H), 3.41 (t, *J* = 7.4 Hz, 1H), 2.54 (dt, *J* = 17.4, 7.2 Hz, 1H), 2.46 (dt, *J* = 17.4, 7.2 Hz, 1H), 1.98 (q, *J* = 7.2 Hz, 2H), 1.88–1.79 (m, 2H), 1.68 (s, 3H), 1.65–1.58 (m, 5H), 1.35–1.29 (m, 4H), 1.26 (t, *J* = 7.1 Hz, 3H), 0.89 (t, *J* = 7.1 Hz, 3H); ^{13}C NMR (126 MHz, $CDCl_3$) δ 205.7, 170.2, 132.6, 123.8, 61.3, 59.4, 41.4, 29.8, 28.1, 27.4, 25.9, 23.8, 22.6, 17.9, 14.3, 14.0; IR (neat, cm^{-1}): 2959, 2873, 1743, 1716, 1456, 1368, 1179; ESI HRMS *m/z* $(M+Na)^+$ calcd 291.1931, obsd 291.1932.

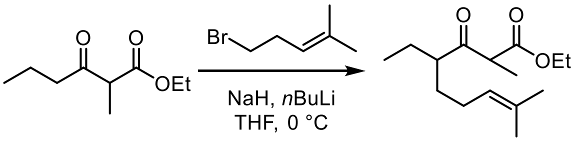

S157

Ethyl 4-ethyl-2,8-dimethyl-3-oxonon-7-enoate (S157). The title compound was prepared as a colorless oil (34% yield) and obtained as a 1:0.6 mixture of diastereoisomers from ethyl 2-methyl-3-oxohexanoate by following the GP 6. ^{1}H NMR (400 MHz, $CDCl_3$) δ 5.10–5.03 (m, 1.6H), 4.25–4.08 (m, 3.2H), 3.64 (q, *J* = 7.1 Hz, 1.6H), 2.71–2.62 (m, 1.6H), 2.52–2.33 (m, 1H), 1.99–1.86 (m, 3.2H), 1.76–1.36 (m, 15H), 1.32 (d, *J* = 7.1 Hz, 3H), 1.31 (d, *J* = 7.1 Hz, 1.8H), 1.26 (t, *J* = 7.1 Hz, 4.8H), 0.86 (2t, *J* = 7.4 Hz, 4.8H); ^{13}C NMR (101 MHz, $CDCl_3$) δ 209.2, 209.1, 170.5, 170.4, 132.5, 132.3, 123.9, 123.8, 61.4, 52.5, 52.4, 31.2, 30.7, 25.9, 25.8, 25.8, 24.4, 24.0, 17.8 (2C), 14.2, 13.0 (2C), 11.8, 11.7; IR (neat, cm^{-1}): 2967, 2937, 1743, 1713, 1457, 1378, 1195; ESI HRMS *m/z* $(M+Na)^+$ calcd 277.1774, obsd 277.1774.

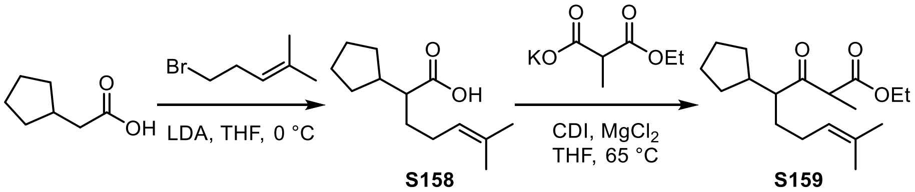

Ethyl 4-cyclopentyl-2,8-dimethyl-3-oxonon-7-enoate (S159). The title compound was prepared as a colorless oil (79% yield in two steps) and a 1:1 mixture of diastereoisomers from 2-cyclopentylacetic acid by following the GP 8. The compound contained < 10% of enol form. ^{1}H NMR (400 MHz, $CDCl_3$) δ 5.10–4.99 (m, 1H), 4.25–4.11 (m, 2H), 3.65 (q, J = 7.0 Hz, 1H), 2.67–2.58 (m, 1H), 2.14–1.43 (m, 11H), 1.67 (s, 3H), 1.57 (s, 3H), 1.31 (2d, J = 7.0 Hz, 3H), 1.26 (t, J = 7.1 Hz, 3H), 1.19–1.02 (m, 2H); ^{13}C NMR (101 MHz, $CDCl_3$) δ 209.3 (2C), 170.3 (2C), 132.4, 132.1, 124.1, 123.9, 61.3 (2C), 56.3, 56.2, 53.6, 53.0, 42.3, 41.5, 31.4, 31.2, 30.5, 30.4, 30.3, 30.2, 25.8 (2C), 25.7, 25.2 (2C), 24.9, 24.8, 17.8 (2C), 14.2, 13.0 (2C); IR (neat, cm^{-1}): 2954, 2871, 1745, 1712, 1452, 1376, 1193; ESI HRMS m/z $(M+Na)^+$ calcd 317.2087, obsd 317.2093.

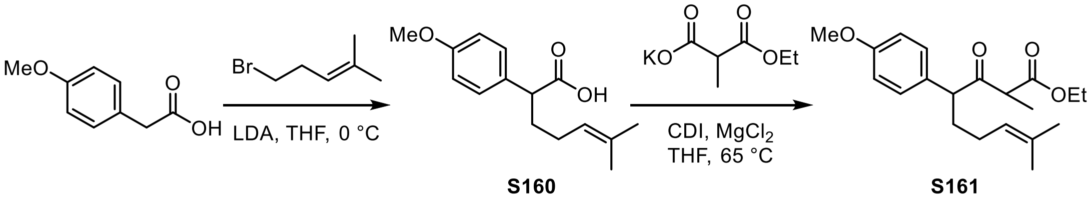

Ethyl 4-(4-methoxyphenyl)-2,8-dimethyl-3-oxonon-7-enoate (S161). The title compound was prepared as a colorless oil (55% yield in two steps) from 2-(4-methoxyphenyl)acetic acid by following the GP 8. The compound was isolated as a 1:0.15 mixture of diastereoisomers and contained traces of enol form. Only the major diastereoisomer was shown. ^{1}H NMR (400 MHz, $CDCl_3$) δ 7.15–7.08 (m, 2H), 6.88–6.83 (m, 2H), 5.10–5.02 (m, 1H), 4.13 (2q, J = 7.2 Hz, 2H), 3.86 (t, J = 7.3 Hz, 1H), 3.79 (s, 3H), 3.57 (q, J = 7.0 Hz, 1H), 2.09–1.99 (m, 1H), 1.90–1.80 (m, 2H), 1.75–1.65 (m, 1H), 1.67 (s, 3H), 1.51 (s, 3H), 1.25 (t, J = 7.3 Hz, 3H), 1.14 (d, J = 7.0 Hz, 3H); ^{13}C NMR (101 MHz, $CDCl_3$) δ 205.5, 170.5, 159.0, 132.3, 130.1, 129.7, 123.8, 114.4, 61.4, 57.1, 55.3, 51.2, 32.4, 25.8, 25.6, 17.8, 14.2, 13.0; IR (neat, cm^{-1}): 2938, 1744, 1715, 1609, 1511, 1455, 1251, 826; ESI HRMS m/z $(M+Na)^+$ calcd 355.1880, obsd 355.1882.

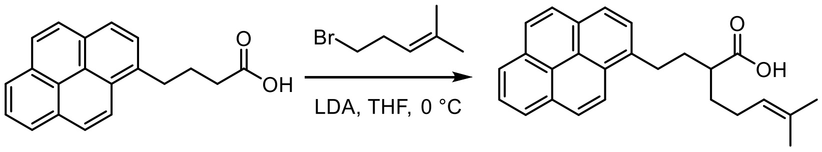

S162

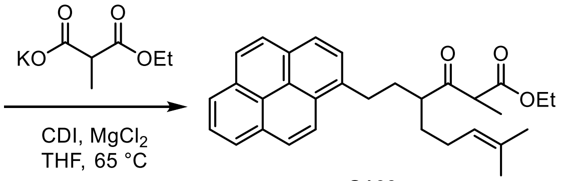

S163

Ethyl 2,8-dimethyl-3-oxo-4-(2-(pyren-1-yl)ethyl)non-7-enoate (S163). The title compound was prepared as a pale yellow oil (46% yield in two steps) from 4-(pyren-1-yl)butanoic acid by following the GP 8. The compound was isolated as a 1:0.8 mixture of diastereoisomers and contained 14% of enol forms. ^{1}H NMR (400 MHz, $CDCl_3$) δ 8.28 (d, J = 9.2 Hz, 1H), 8.21 (d, J = 9.0 Hz, 0.8H), 8.16–7.93 (m, 12.6H), 7.82 (d, J = 7.8 Hz, 1H), 7.80 (d, J = 7.8 Hz, 0.8H), 5.11–5.03 (m, 1.8H), 4.18–4.11 (m, 3.6H), 3.72 (q, J = 7.1 Hz, 1H), 3.67 (q, J = 7.2 Hz, 0.8H), 3.39–3.08 (m, 3.6H), 3.01–2.76 (m, 1.8H), 2.32–2.13 (m, 1.8H), 2.03–1.76 (m, 9H), 1.68 (2s, 5.4H), 1.57 (s, 3H), 1.56 (s, 2.4H), 1.37 (d, J = 7.1 Hz, 3H), 1.36 (d, J = 7.1 Hz, 2.4H), 1.21 (2t, J = 7.1 Hz, 5.4H); ^{13}C NMR (101 MHz, $CDCl_3$) δ 209.1, 208.9, 170.5, 170.4, 136.4, 136.0, 132.9, 132.6, 131.5, 131.1, 131.0, 130.1 (2C), 128.8, 128.7, 127.7, 127.6, 127.2 (2C), 126.9, 126.8, 126.0 (2C), 125.2, 125.1 (3C), 125.0 (2C), 124.9 (2C), 123.6, 123.5 (2C), 123.3, 61.5, 61.4, 52.3, 51.1, 50.9, 33.1, 32.8, 31.7, 31.5, 31.4, 31.3, 25.8 (2C), 17.9, 17.8, 14.3, 14.2, 13.3, 13.1; IR (neat, cm^{-1}): 3041, 2936, 1743, 1711, 1455, 1376, 845; ESI HRMS m/z $(M+Na)^+$ calcd 477.2400, obsd 477.2409.

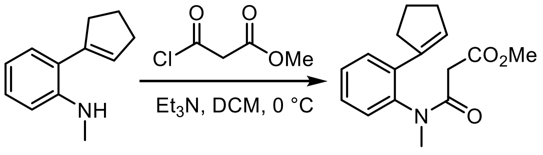

S164 **S165**

Methyl 3-((2-(cyclopent-1-en-1-yl)phenyl)(methyl)amino)-3-oxopropanoate (S165). The title compound was prepared as a pale-yellow oil (83% yield) from **S164**[26] by following the GP 9. ^{1}H NMR (400 MHz, $CDCl_3$) δ 7.43–7.37 (m, 1H), 7.36–7.26 (m, 2H), 7.20–7.15 (m, 1H), 6.02–5.96 (m, 1H), 3.66 (s, 3H), 3.20 (s, 3H), 3.18, 3.09 (ABq, J_{AB} = 15.6 Hz, 2H), 2.68–2.61 (m, 2H), 2.55–2.48 (m, 2H), 1.98 (p, J = 7.6 Hz, 2H); ^{13}C NMR (101 MHz, $CDCl_3$) δ 168.2, 166.3, 140.2, 139.8, 135.7, 131.7, 129.9, 128.8 (2C), 128.3, 52.3, 41.3, 36.5, 35.3, 33.9, 23.6; IR (neat, cm^{-1}): 3479, 2951, 2845, 1747, 1667, 1489, 1436, 1381, 1156, 771, 570; ESI HRMS m/z $(M+Na)^+$ calcd 296.1257, obsd 296.1256.

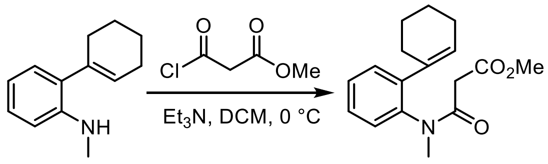

Methyl 3-(methyl(2',3',4',5'-tetrahydro-[1,1'-biphenyl]-2-yl)amino)-3-oxopropanoate (S167). The title compound was prepared as a white solid (80% yield) from **S166**[26] by following the GP 9. ^{1}H NMR (400 MHz, $CDCl_3$) δ 7.36–7.22 (m, 3H), 7.18–7.12 (m, 1H), 5.76–5.69 (m, 1H), 3.68 (s, 3H), 3.24, 3.15 (ABq, J_{AB} = 15.5 Hz, 2H), 3.21 (s, 3H), 2.22–2.13 (m, 4H), 1.75–1.62 (m, 4H); ^{13}C NMR (101 MHz, $CDCl_3$) δ 168.3, 166.4, 142.4, 140.4, 136.5, 130.5, 128.8, 128.7, 128.3, 128.2, 52.3, 41.3, 37.2, 28.9, 25.8, 23.1, 21.9; IR (neat, cm^{-1}): 2933, 2857, 1748, 1663, 1486, 1436, 1380, 1156, 760; ESI HRMS *m/z* (M+Na)$^+$ calcd 310.1414, obsd 310.1413.

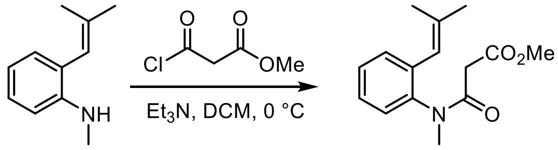

Methyl 3-(methyl(2-(2-methylprop-1-en-1-yl)phenyl)amino)-3-oxopropanoate (S169). The title compound was prepared as a colorless oil (77% yield) from **S168**[26] by following the GP 9. ^{1}H NMR (400 MHz, $CDCl_3$) δ 7.35–7.32 (m, 2H), 7.31–7.25 (m, 1H), 7.20–7.16 (m, 1H), 6.11–6.07 (m, 1H), 3.65 (s, 3H), 3.12, 3.06 (ABq, J_{AB} = 15.6 Hz, 2H), 1.91 (d, J = 1.5 Hz, 3H), 1.81 (d, J = 1.5 Hz, 3H); ^{13}C NMR (101 MHz, $CDCl_3$) δ 168.1, 166.3, 141.4, 138.6, 136.5, 131.4, 128.3, 128.0, 127.9, 120.3, 52.2, 41.1, 36.4, 26.6, 19.4; IR (neat, cm^{-1}): 2952, 1747, 1667, 1485, 1435, 1380, 1158, 773; ESI HRMS *m/z* (M+Na)$^+$ calcd 284.1257, obsd 284.1256.

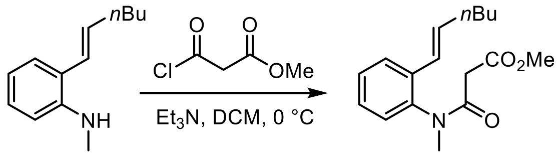

Methyl (*E*)-3-((2-(hex-1-en-1-yl)phenyl)(methyl)amino)-3-oxopropanoate (S171). The title compound was prepared as a yellow oil (81% yield) from **S170**[26] by following the GP 9. ^{1}H NMR (400 MHz, $CDCl_3$) δ 7.58 (dd, J = 7.6, 1.5 Hz, 1H), 7.33 (td, J = 7.6, 1.5 Hz, 1H), 7.26 (td, J = 7.6, 1.5 Hz, 1H), 7.15 (dd, J = 7.6, 1.5 Hz, 1H), 6.38–6.25 (m, 2H), 3.65 (s, 3H), 3.23 (s, 3H), 3.12 (s, 2H), 2.27–2.20 (m, 2H), 1.50–1.32 (m, 4H), 0.93 (t, J = 7.2 Hz, 3H); ^{13}C NMR (101 MHz, $CDCl_3$) δ 168.0, 166.4, 140.0, 135.6, 135.5, 129.0, 128.3, 126.7, 123.9, 52.2, 41.3, 36.7, 33.1, 31.5, 22.3, 14.0; IR (neat, cm^{-1}): 2956, 2928, 1749, 1668, 1380, 1156; ESI HRMS *m/z* (M+Na)$^+$ calcd 312.1570, obsd 312.1571.

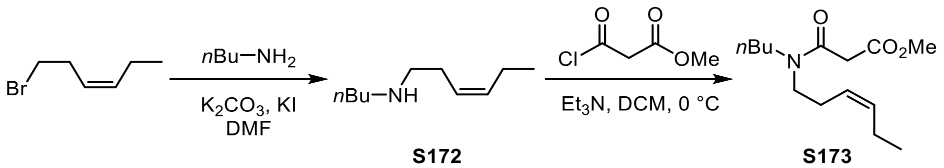

S172 **S173**

Methyl (*Z*)-3-(butyl(hex-3-en-1-yl)amino)-3-oxopropanoate (S173). A solution of butylamine (3.0 mL, 30 mmol, 1.5 equiv) in DMF (40 mL) was treated with KI (0.33 g, 2.0 mmol, 10 mol%), K_2CO_3 (2.8 g, 20 mmol, 1.0 equiv) and (*Z*)-1-bromohex-3-ene (3.2 g, 20 mmol, 1.0 equiv). The mixture stirred at rt for 12 h. The reaction mixture was treated with H_2O (40 mL) and extracted three times with Et_2O. The combined organic layer was washed with brine, dried with anhydrous Na_2SO_4, filtered, and concentrated under reduced pressure to afford the crude amine **S172**. **S172** was dissolved in anhydrous CH_2Cl_2 (35 mL). Et_3N (5.5 mL, 40 mmol, 2.0 equiv) was added. The mixture was cooled to 0 °C and treated with methyl malonyl chloride (2.6 mL, 24 mmol, 1.2 equiv). The mixture was allowed to warm to rt and stirred until complete consumption of **S172** (monitored by TLC). The solvent was removed under reduced pressure. EtOAc (40 mL) was added and the mixture was filtered. The filtrate was concentrated under reduced pressure. The residue was purified by flash column chromatography on silica gel to afford **S173** as a yellow oil (2.1 g, 50% yield). The title compound was isolated as a 1:1 mixture of rotamers. ^{1}H NMR (500 MHz, $CDCl_3$) δ 5.56–5.42 (m, 2H), 5.34–5.23 (m, 2H), 3.75 (s, 3H), 3.74 (s, 3H), 3.46 (s, 2H), 3.43 (s, 2H), 3.37–3.31 (m, 4H), 3.26–3.20 (m, 4H), 2.34–2.28 (m, 4H), 2.06 (h, J = 7.4 Hz, 4H), 1.59–1.51 (m, 4H), 1.36–1.29 (m, 4H), 1.00–0.91 (m, 12H); ^{13}C NMR (101 MHz, $CDCl_3$) δ 168.4 (2C), 165.6, 135.2, 134.2, 125.1, 123.9, 52.5, 48.9, 48.3, 46.2, 45.8, 41.3, 41.2, 31.2, 29.8, 29.7, 26.8, 25.6, 20.8, 20.7, 20.3, 20.2, 14.5, 14.3, 14.0, 13.9; IR (neat, cm^{-1}): 2961, 2875, 1746, 1651, 1436, 1255, 1020; ESI HRMS *m/z* $(M+Na)^+$ calcd 278.1727, obsd 278.1728.

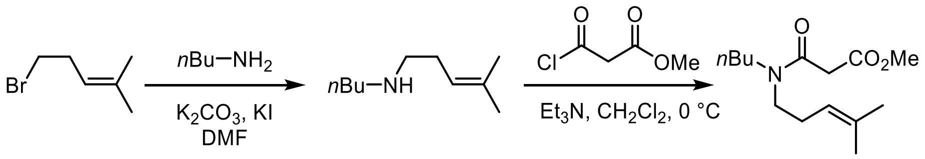

S174 **S175**

Methyl 3-(butyl(4-methylpent-3-en-1-yl)amino)-3-oxopropanoate (S175). The title compound was prepared as a pale-yellow oil (37% yield) from 5-bromo-2-methylpent-2-ene by following the procedure described for the synthesis of **S173**. The compound was isolated as a 1:0.9 mixture of rotamers. ^{1}H NMR (400 MHz, $CDCl_3$) δ 5.11–5.04 (m, 1.9H), 3.75 (s, 3H), 3.74 (s, 2.7H), 3.45 (s, 2H), 3.43 (s, 1.8H), 3.37–3.27 (m, 3.8H), 3.25–3.19 (m, 3.8H), 2.25 (2q, J = 7.5 Hz, 3.8H), 1.71 (s, 3H), 1.69 (s, 2.7H), 1.63 (2s, 5.7H), 1.59–1.50 (m, 3.8H), 1.37–1.27 (m, 3.8H), 0.95 (t, J = 7.3 Hz, 2.7H), 0.92 (t, J = 7.3 Hz, 3H); ^{13}C NMR (101 MHz, $CDCl_3$) δ 168.3 (2C), 165.5 (2C), 135.1, 134.0, 120.6, 119.6, 52.4, 48.7, 48.2, 46.2, 45.6, 41.1, 41.0, 31.1, 29.6, 27.5, 26.3, 25.7, 20.2, 20.1, 17.8 (2C), 13.9, 13.8; IR (neat, cm^{-1}): 2946,

1748, 1633, 1224, 1048; ESI HRMS *m/z* $(M+H)^+$ calcd 256.1907, obsd 256.1918.

Mechanstic Studies

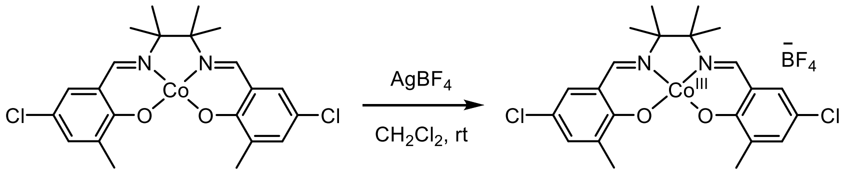

***N,N'*-bis(3-Methyl-5-chlorosalicylidene)-1,2-dimethyldiaminato cobalt(III) tetrafluoroborate (95).** The title compound was obtained as a dark green solid in 41% yield (0.12 g) according to the literature procedure[27]. A 20-mL vial was charged with **[Co]-1** (0.23 g, 0.50 mmol, 1.0 equiv). CH_2Cl_2 (5 mL) was added, followed by $AgBF_4$ (0.11 g, 0.55 mmol, 1.1 equiv). The reaction flask was wrapped in aluminum foil. After being stirred for 12 h at rt, the dark green reaction mixture was filtered through Celite and concentrated. The resulting solid was taken up in hexane (30 mL) and collected on a fritted filter. The dark green solid was washed with additional hexane (50 mL) until the filtrate was clear. ^1H NMR (500 MHz, DMSO-d_6) δ 8.22–8.16 (m, 2H), 7.68–7.63 (m, 2H), 7.43–7.36 (m, 2H), 2.62 (s, 6H), 1.47 (s, 12H); ^{13}C NMR (126 MHz, DMSO-d_6) δ 167.1, 162.7, 134.7, 133.7, 132.3, 120.1, 118.4, 76.1, 27.2, 17.8; ^{19}F NMR (471 MHz, DMSO-d_6) δ −148.3 (2F); IR (neat, cm^{-1}): 3734, 2929, 1623, 1435, 1308, 1057, 786, 410; ESI HRMS *m/z* $(C_{22}H_{24}Cl_2CoN_2O_2)^+$ calcd 477.0547, obsd 477.0551.

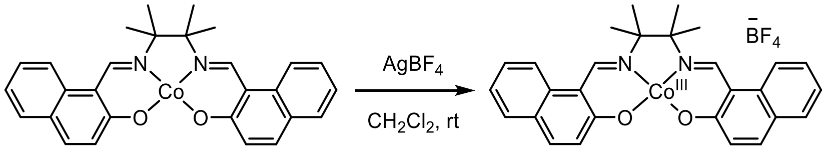

***N,N'*-bis(methanylylidene)-*N,N'*-bis(naphthalen-2-ol)-1,2-dimethyldiaminato cobalt(III) tetrafluoroborate (S176):** The title compound was obtained as a green solid (47% yield) according to the procedure described for the synthesis of **95**. ^1H NMR (500 MHz, DMSO-d_6) δ 8.75 (s, 2H), 8.26 (d, J = 8.5 Hz, 2H), 8.01 (d, J = 9.1 Hz, 2H), 7.88 (d, J = 7.8 Hz, 2H), 7.78 (d, J = 9.0 Hz, 2H), 7.62 (t, J = 7.6 Hz, 2H), 7.29 (t, J = 7.4 Hz, 2H), 1.66 (s, 12H); ^{13}C NMR (126 MHz, DMSO-d_6) δ 166.5, 160.7, 136.3, 134.9, 129.7, 128.7, 128.0, 125.6, 123.6, 121.5, 110.0, 76.9, 27.7; ^{19}F NMR (471 MHz, DMSO-d_6) δ −148.2 (2F); ESI HRMS *m/z* $(C_{28}H_{26}CoN_2O_2)^+$ calcd 481.1321, obsd 481.1320.

Reaction of 1 with stoichiometric Co^{III} complex 95. A 5 mL round-bottomed flask was charged with **1** (0.05 mmol, 1.0 equiv), **95** (0.15 mmol, 3.0 equiv) and the corresponding base (0.10 mmol, 2.0 equiv). The flask was then equipped with a condenser and flushed with argon.

MeCN (2 mL) was added. The reaction was quenched by H_2O (1 mL) after 7 h. The phases were separated, and the aqueous phase was extracted with EtOAc. The combined organic solution was dried over anhydrous $MgSO_4$, filtered, and concentrated under reduced pressure. The yield were determined by ^{1}H NMR analysis using 1,3,5-trimethoxybenzene as the internal standard.

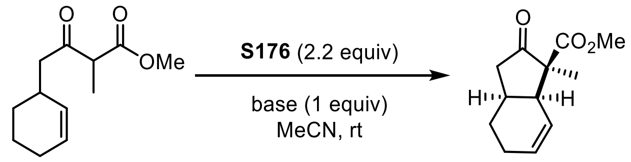

S130

50, with Na_2CO_3: trace
with Cs_2CO_3: 27%

Reaction of S130 with stoichiometric Co^{III} complex S176. A 10 mL schlenk tube was charged with **S130** (0.050 mmol, 1.0 equiv), **S176** (0.12 mmol, 2.2 equiv) and the corresponding base (0.050 mmol, 1.0 equiv) and flushed with argon. MeCN (1 mL) was added. The reaction mixture was stirred for 14 h (for Na_2CO_3) or 10 h (for Cs_2CO_3) at rt. EtOAc (5 mL) was added and the mixture was filtered. The organic solution was concentrated under reduced pressure. The yields were determined by ^{1}H NMR analysis using 1,3,5-trimethoxybenzene as the internal standard.

Coordinates and energies

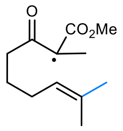

C	-0.855352	2.424171	-0.966452
C	-1.702555	1.181870	-0.913511
C	1.316168	1.380884	1.713336
O	-1.075292	0.039732	2.120846
C	-0.717213	-1.906586	0.818287
C	-0.327968	-0.529467	1.332876
C	0.945007	0.139735	0.978320
C	-1.268412	0.022174	-1.422317
C	-1.981951	-1.305207	-1.332098
C	-2.072602	-1.863493	0.097653
C	-3.009996	1.330415	-0.186068
C	1.867396	-0.368846	-0.054695
O	1.658385	-1.267880	-0.844566
O	3.023475	0.324742	-0.051607
C	3.966807	-0.083279	-1.039915
H	-1.349620	3.218571	-1.539095
H	-0.697330	2.818128	0.046535
H	0.122917	2.229259	-1.417957
H	0.483201	1.702516	2.337313
H	2.195151	1.198085	2.344953
H	1.601129	2.176406	1.017309
H	-0.817727	-2.524370	1.718777
H	0.051787	-2.341346	0.181549
H	-0.293117	0.009229	-1.905822
H	-2.997863	-1.225213	-1.741293
H	-1.450248	-2.030151	-1.957690
H	-2.755358	-1.259111	0.704257
H	-2.489778	-2.875644	0.056990
H	-3.720317	0.538629	-0.438557
H	-2.834029	1.293450	0.898233
H	-3.475574	2.295983	-0.412891
H	4.832182	0.564038	-0.906635
H	4.240947	-1.130749	-0.895241
H	3.545131	0.035301	-2.040613

Energies (0K) = -694.033335669

Energies (0K) + ZPE = -693.737935

Enthalpies (298K) = -693.719644

Free Energies (298K) = -693.784393

Transition state

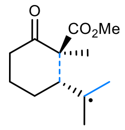

C	-1.615057	2.725621	-0.365979
C	-1.735124	1.249789	-0.598735
C	0.401780	1.259602	1.652604
O	-1.188773	-0.922294	2.113192
C	-0.453241	-2.342384	0.357584
C	-0.396764	-1.085827	1.193902
C	0.481055	0.036495	0.782464
C	-0.647463	0.512134	-1.039855
C	-0.746742	-0.851325	-1.713488
C	-1.289833	-2.031789	-0.896450
C	-3.068802	0.634879	-0.288688
C	1.761566	-0.215720	0.089564
O	2.115384	-1.235003	-0.472118
O	2.540549	0.890554	0.087960
C	3.783189	0.744375	-0.593394
H	-2.332757	3.263283	-1.000427
H	-1.860489	2.987097	0.671521
H	-0.612698	3.099980	-0.587845
H	-0.621474	1.391397	2.006664
H	1.041761	1.135373	2.534723
H	0.739084	2.152343	1.123443
H	-0.952131	-3.110130	0.954010
H	0.542885	-2.675899	0.068657
H	0.190263	1.118964	-1.387863
H	-1.373725	-0.727035	-2.607630
H	0.251702	-1.119964	-2.071127

H	-2.323641	-1.852279	-0.587310
H	-1.305598	-2.915229	-1.543427
H	-3.406392	-0.031308	-1.090474
H	-3.014732	0.040930	0.634080
H	-3.829641	1.406923	-0.145448
H	4.287592	1.704745	-0.495777
H	4.377847	-0.051424	-0.139477
H	3.617484	0.501088	-1.645725

Energies (0K) = -694.018565210

Energies (0K) + ZPE = -693.722981

Enthalpies (298K) = -693.705781

Free Energies (298K) = -693.766550

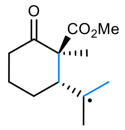

C	-2.675373	2.290407	-0.215359
C	-1.970279	1.054297	0.248376
C	0.307468	0.325565	2.053444
O	-0.203226	-2.292024	1.415841
C	-0.309181	-2.102451	-0.966983
C	-0.081356	-1.586945	0.440558
C	0.314761	-0.103798	0.582260
C	-0.587979	0.813110	-0.298690
C	-0.615000	0.281754	-1.751700
C	-1.154467	-1.146135	-1.821069
C	-2.837024	0.005780	0.876395
C	1.752377	0.003206	0.072351
O	2.433691	-0.917702	-0.310834
O	2.203880	1.266208	0.122919
C	3.556879	1.438702	-0.300800
H	-1.974488	3.095541	-0.459847
H	-3.282604	2.102614	-1.117048
H	-3.369390	2.660563	0.549307
H	-0.681182	0.169289	2.489020
H	1.021320	-0.266979	2.630951
H	0.568063	1.384005	2.131297
H	-0.759366	-3.095083	-0.887861
H	0.684126	-2.213183	-1.418884
H	-0.088881	1.790681	-0.319421
H	-1.226649	0.951822	-2.366400
H	0.401971	0.310968	-2.170317
H	-2.194676	-1.160518	-1.475293
H	-1.164044	-1.498783	-2.857447
H	-3.627886	-0.332271	0.186723
H	-2.292843	-0.881196	1.207284
H	-3.358805	0.417624	1.751474
H	3.770646	2.499542	-0.181271
H	4.226367	0.836136	0.316158
H	3.668614	1.136421	-1.344524

Energies (0K) = -694.047128234

Energies (0K) + ZPE = -693.750398

Enthalpies (298K) = -693.732670

Free Energies (298K) = -693.796312

Transition state

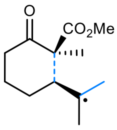

C	2.140105	1.394098	-1.018661
C	1.181389	1.657839	0.104858
C	-0.017241	-0.534351	2.186181
O	-2.651753	-0.554932	1.568191
C	-2.544229	-0.482234	-0.804886
C	-1.940235	-0.602459	0.575301
C	-0.464479	-0.590668	0.752442
C	-0.179851	1.447341	-0.022053
C	-0.870626	1.367507	-1.368770
C	-2.344017	0.965018	-1.290017
C	1.787254	2.130764	1.389485
C	0.414739	-1.257719	-0.225384
O	0.135680	-1.586345	-1.363646
O	1.662406	-1.433305	0.268699
C	2.569402	-2.084732	-0.615357

H	1.739905	0.689717	-1.753468
H	2.379864	2.325599	-1.550984
H	3.084492	0.993103	-0.633483
H	-0.587031	0.225712	2.725751
H	-0.224530	-1.491633	2.679728
H	1.050115	-0.335248	2.267935
H	-3.610095	-0.703226	-0.711008
H	-2.076762	-1.175079	-1.506035
H	-0.801199	1.822322	0.794634
H	-0.344473	0.670351	-2.029535
H	-0.795163	2.359410	-1.834911
H	-2.801199	1.078904	-2.278051
H	-2.876822	1.644178	-0.611380
H	2.347093	3.061861	1.226428
H	1.033224	2.312324	2.159320
H	2.508601	1.397206	1.773749
H	3.508404	-2.164866	-0.068748
H	2.195196	-3.074853	-0.885234
H	2.706443	-1.501552	-1.529482

Energies (0K) = -694.023934584

Energies (0K) + ZPE = -693.728277

Enthalpies (298K) = -693.711141

Free Energies (298K) = -693.771302

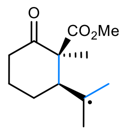

C	2.228512	1.117918	-1.030352
C	1.298342	1.618359	0.030869
C	0.099708	-0.281893	2.100734
O	-2.460203	-0.944433	1.595968
C	-2.637453	-0.263099	-0.684479
C	-1.897572	-0.537381	0.605232
C	-0.384190	-0.218005	0.653443
C	-0.152540	1.233409	0.059694
C	-0.856472	1.421218	-1.294446
C	-2.358114	1.161652	-1.182894
C	1.900385	2.449697	1.117038
C	0.353855	-1.206857	-0.241341
O	-0.019447	-1.578259	-1.332013
O	1.518027	-1.605224	0.289536
C	2.287946	-2.476617	-0.538688
H	2.694972	1.952229	-1.574630
H	3.053803	0.538501	-0.589092
H	1.731736	0.480200	-1.768259
H	-0.469860	0.430219	2.704302
H	-0.062457	-1.275743	2.521681
H	1.161938	-0.041322	2.165192
H	-3.700390	-0.431700	-0.496189
H	-2.287418	-0.980257	-1.434091
H	-0.657543	1.895926	0.779476
H	-0.426742	0.747191	-2.043188
H	-0.673113	2.446204	-1.635910
H	-2.843915	1.311688	-2.152127
H	-2.803311	1.885655	-0.487879
H	2.406681	3.336435	0.707607
H	1.151121	2.791674	1.837063
H	2.668891	1.893657	1.676648
H	3.165365	-2.742417	0.048423
H	1.708906	-3.364568	-0.798638
H	2.579876	-1.960653	-1.457173

Energies (0K) = -694.050217006

Energies (0K) + ZPE = -693.752546

Enthalpies (298K) = -693.735402

Free Energies (298K) = -693.795694

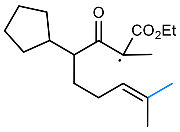

C	-4.161030	-0.339914	-0.008123
C	-2.999703	-0.223454	-0.960182
C	-1.668112	-0.311072	2.709289
O	-0.500427	-2.440296	1.639257
C	0.898261	-1.631844	-0.060750

C	-0.149275	-1.424326	1.047147
C	-0.807012	-0.172800	1.500147
C	-2.125783	-1.227248	-1.109245
C	-0.886962	-1.272908	-1.968045
C	0.212082	-2.148301	-1.347818
C	-2.923575	1.093352	-1.681739
C	1.989936	-0.579406	-0.291405
C	2.500782	0.072103	1.007689
C	3.955112	0.524095	0.719908
C	4.292305	-0.035382	-0.680336
C	3.279159	-1.164876	-0.889979
C	-0.800142	1.162991	0.871952
O	-1.312750	2.138849	1.383885
O	-0.189553	1.209235	-0.321463
C	1.214896	3.193096	-0.297543
C	0.001359	2.508220	-0.899307
H	-4.088523	0.430639	0.771661
H	-5.115246	-0.177945	-0.524162
H	-4.193177	-1.320038	0.475985
H	-2.541977	-0.929257	2.465697
H	-1.135327	-0.845447	3.500485
H	-2.001986	0.664467	3.059192
H	1.428399	-2.504961	0.343471
H	-2.318022	-2.139230	-0.539452
H	-1.141154	-1.711884	-2.943392
H	-0.505793	-0.266360	-2.166323
H	-0.223997	-3.127488	-1.113874
H	0.995837	-2.326627	-2.092828
H	-3.834647	1.249978	-2.272506
H	-2.871751	1.918822	-0.959011
H	-2.067692	1.161708	-2.355958
H	1.641697	0.198504	-0.977065
H	1.863652	0.896966	1.341963
H	2.490444	-0.680168	1.808110
H	4.637770	0.119638	1.472759
H	4.060528	1.612659	0.754381
H	4.133322	0.736449	-1.443055
H	5.330523	-0.367158	-0.766812
H	3.580333	-2.053270	-0.317326
H	3.180372	-1.460870	-1.939081
H	1.361589	4.171255	-0.764517
H	1.071186	3.342790	0.776047
H	2.117069	2.594008	-0.457877
H	0.138305	2.315171	-1.966733
H	-0.903675	3.101391	-0.753163

Energies (0K) = -928.588901095

Energies (0K) + ZPE = -928.140383

Enthalpies (298K) = -928.116549

Free Energies (298K) = -928.191932

Transition state

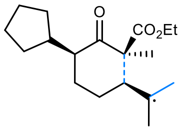

C	-4.347113	-0.587587	-0.283382
C	-3.037649	-0.461011	-0.999027
C	-2.085247	-0.618628	2.214094
O	0.406130	-1.606992	2.430615
C	1.127338	-1.615380	0.182470
C	0.190157	-1.170813	1.310352
C	-1.072435	-0.377049	1.127057
C	-1.969655	-1.279561	-0.696873
C	-0.747658	-1.413606	-1.581787
C	0.349353	-2.280772	-0.965036
C	-2.956114	0.666182	-1.983962
C	2.152964	-0.578231	-0.299713
C	2.786267	0.243508	0.826905
C	3.993763	0.913086	0.154391
C	4.442681	-0.074000	-0.958618
C	3.389740	-1.203001	-0.965866
C	-1.093544	1.001507	0.607756
O	-2.004920	1.783249	0.794841
O	-0.020748	1.311012	-0.150116
C	0.506076	3.627823	0.372335

C	0.029616	2.642748	-0.678844
H	-4.548816	0.322606	0.297620
H	-5.170410	-0.694131	-1.001922
H	-4.365179	-1.444665	0.394606
H	-2.276034	-1.688536	2.336623
H	-1.697030	-0.252505	3.170764
H	-3.013481	-0.091251	1.997472
H	1.712098	-2.397333	0.683512
H	-2.201901	-2.163127	-0.098536
H	-1.066417	-1.869573	-2.528764
H	-0.340858	-0.428497	-1.833205
H	-0.098854	-3.215244	-0.602168
H	1.066774	-2.566287	-1.742624
H	-3.680709	0.516308	-2.795336
H	-3.218103	1.610058	-1.489001
H	-1.965842	0.775786	-2.431339
H	1.690877	0.110505	-1.015966
H	2.087982	0.957086	1.274904
H	3.112137	-0.440178	1.622757
H	4.794472	1.138609	0.863600
H	3.684649	1.865521	-0.291728
H	4.474956	0.430781	-1.928942
H	5.445874	-0.468590	-0.776332
H	3.734025	-2.046585	-0.353355
H	3.197051	-1.588059	-1.972113
H	0.593606	4.627866	-0.062183
H	-0.206148	3.668966	1.199415
H	1.486163	3.330901	0.757030
H	0.733338	2.576439	-1.512913
H	-0.959155	2.913691	-1.058109

Energies (0K) = -928.577374949

Energies (0K) + ZPE = -928.130399

Enthalpies (298K) = -928.106680

Free Energies (298K) = -928.183543

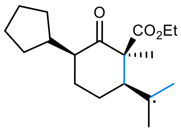

C	-4.365760	-0.958044	-0.342291
C	-2.998142	-0.739491	-0.904455
C	-2.153913	-0.417150	2.153831
O	0.582923	-0.280440	2.463050
C	1.166425	-1.518879	0.524995
C	0.211562	-0.695636	1.386897
C	-1.235361	-0.360581	0.930724
C	-1.783506	-1.298724	-0.213440
C	-0.681560	-1.734443	-1.186826
C	0.432255	-2.459810	-0.437500
C	-2.893660	0.244095	-2.029346
C	2.179550	-0.615205	-0.207573
C	2.872713	0.435306	0.667367
C	4.057200	0.862578	-0.199443
C	4.586485	-0.467772	-0.766116
C	3.354177	-1.409767	-0.829792
C	-1.239934	1.074069	0.410238
O	-2.096094	1.889571	0.643095
O	-0.190762	1.311743	-0.399544
C	0.392645	3.656678	-0.045491
C	-0.135896	2.612798	-1.010823
H	-4.750837	-0.048798	0.147286
H	-5.084204	-1.205809	-1.136040
H	-4.383929	-1.764506	0.397554
H	-2.202638	-1.441609	2.535623
H	-1.769401	0.229885	2.943037
H	-3.157709	-0.084570	1.886141
H	1.746033	-2.102104	1.253028
H	-2.105921	-2.209210	0.315419
H	-1.127362	-2.393812	-1.940031
H	-0.271758	-0.870179	-1.721154
H	-0.004411	-3.297445	0.122242
H	1.153111	-2.895721	-1.137766
H	-3.538556	-0.051957	-2.868439

H	-3.234431	1.242416	-1.713576
H	-1.875475	0.348916	-2.414619
H	1.659581	-0.081332	-1.010961
H	2.206763	1.256503	0.945863
H	3.223208	-0.028919	1.598625
H	4.819293	1.423663	0.348481
H	3.697591	1.504474	-1.014277
H	5.068788	-0.343645	-1.739320
H	5.338397	-0.883011	-0.087722
H	3.537712	-2.323815	-0.251907
H	3.131145	-1.723390	-1.853900
H	0.448411	4.628080	-0.545542
H	-0.272623	3.746815	0.815677
H	1.394759	3.389970	0.300853
H	0.530920	2.480890	-1.866051
H	-1.136681	2.871103	-1.367070

Energies (0K) = -928.610144115

Energies (0K) + ZPE = -928.160993

Enthalpies (298K) = -928.137405

Free Energies (298K) = -928.214045

Transition state

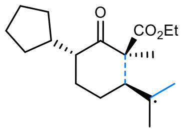

C	-3.993992	-1.923997	0.346907
C	-3.025688	-1.302426	-0.611453
C	-1.630132	-0.759054	2.305721
O	0.959091	-1.318220	1.911035
C	1.313853	-0.483512	-0.287990
C	0.492766	-0.672943	0.981064
C	-0.935720	-0.265514	1.065912
C	-1.670379	-1.570739	-0.544660
C	-0.711516	-1.252257	-1.675176
C	0.762302	-1.471911	-1.333637
C	-3.625464	-0.321521	-1.573142
C	2.796594	-0.712424	-0.002288
C	3.701463	-0.570858	-1.239227
C	5.064708	-0.208926	-0.646611
C	4.701048	0.816291	0.433377
C	3.374851	0.295890	1.031242
C	-1.495202	0.996271	0.537337
O	-2.596899	1.409312	0.843092
O	-0.713649	1.638237	-0.358523
C	-1.076750	4.010430	0.055380
C	-1.253316	2.850635	-0.906855
H	-4.497663	-1.147236	0.937885
H	-4.780098	-2.462146	-0.199198
H	-3.508868	-2.622390	1.032768
H	-1.490762	-1.836530	2.423829
H	-1.182619	-0.288631	3.189137
H	-2.689937	-0.509392	2.282625
H	1.175545	0.531765	-0.671515
H	-1.384036	-2.426530	0.070830
H	-0.975591	-1.894350	-2.526396
H	-0.844965	-0.218992	-2.012109
H	0.913851	-2.496215	-0.963501
H	1.354046	-1.376476	-2.250593
H	-4.078396	0.510499	-1.018619
H	-2.901279	0.091604	-2.277873
H	-4.427538	-0.799429	-2.150412
H	2.909854	-1.725039	0.402015
H	3.725732	-1.472661	-1.858976
H	3.345987	0.257048	-1.870968
H	5.774183	0.173614	-1.386258
H	5.510553	-1.099046	-0.185230
H	5.484631	0.944248	1.185284
H	4.543622	1.792871	-0.039316
H	2.670908	1.116485	1.213642
H	3.528461	-0.199372	1.992754
H	-1.423624	4.938517	-0.408224
H	-1.656765	3.837004	0.964135
H	-0.022331	4.127548	0.319834
H	-0.689981	3.005754	-1.829594

H -2.307838 2.692246 -1.146204

Energies (0K) = -928.586003429

Energies (0K) + ZPE = -928.138432

Enthalpies (298K) = -928.114977

Free Energies (298K) = -928.190865

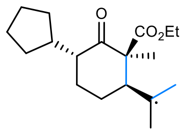

C	-4.084845	-2.127326	0.141010
C	-3.089434	-1.256585	-0.556882
C	-1.763189	-0.765632	2.191584
O	0.910287	-0.703208	2.079589
C	1.271809	-0.491362	-0.277671
C	0.422468	-0.514168	0.987503
C	-1.118992	-0.398777	0.855904
C	-1.618992	-1.389573	-0.272741
C	-0.753640	-1.355551	-1.542195
C	0.724862	-1.572702	-1.228938
C	-3.637979	-0.112275	-1.352459
C	2.756486	-0.652009	0.039373
C	3.650174	-0.760818	-1.209738
C	5.065416	-0.329028	-0.751181
C	4.884739	0.276519	0.662147
C	3.379640	0.532154	0.789895
C	-1.525928	1.036840	0.525534
O	-2.292605	1.692369	1.187159
O	-0.966141	1.496680	-0.609334
C	-0.558870	3.869321	-0.234504
C	-1.343109	2.826244	-1.007783
H	-4.686775	-1.560795	0.869394
H	-4.800793	-2.559620	-0.572212
H	-3.604598	-2.948871	0.681019
H	-1.507822	-1.795162	2.455741
H	-1.402216	-0.111121	2.985589
H	-2.848111	-0.663654	2.126923
H	1.115564	0.480395	-0.766142
H	-1.463395	-2.382219	0.176163
H	-1.115335	-2.141533	-2.215133
H	-0.873101	-0.402962	-2.066596
H	0.871975	-2.561614	-0.770469
H	1.308610	-1.563399	-2.155008
H	-4.015525	0.690330	-0.697494
H	-2.896122	0.335051	-2.020456
H	-4.489392	-0.438961	-1.962894
H	2.886480	-1.551386	0.658083
H	3.639236	-1.762013	-1.650626
H	3.274465	-0.069784	-1.976812
H	5.487406	0.403825	-1.444779
H	5.755957	-1.176680	-0.731837
H	5.191352	-0.449304	1.423450
H	5.485257	1.177363	0.815884
H	3.109468	1.463948	0.270718
H	3.035069	0.605998	1.823146
H	-0.805367	4.869713	-0.601693
H	-0.806314	3.818461	0.827731
H	0.515794	3.709851	-0.360053
H	-1.118305	2.862561	-2.075708
H	-2.419491	2.943225	-0.858407

Energies (0K) = -928.612476476

Energies (0K) + ZPE = -928.163025

Enthalpies (298K) = -928.139422

Free Energies (298K) = -928.215959

NMR spectra

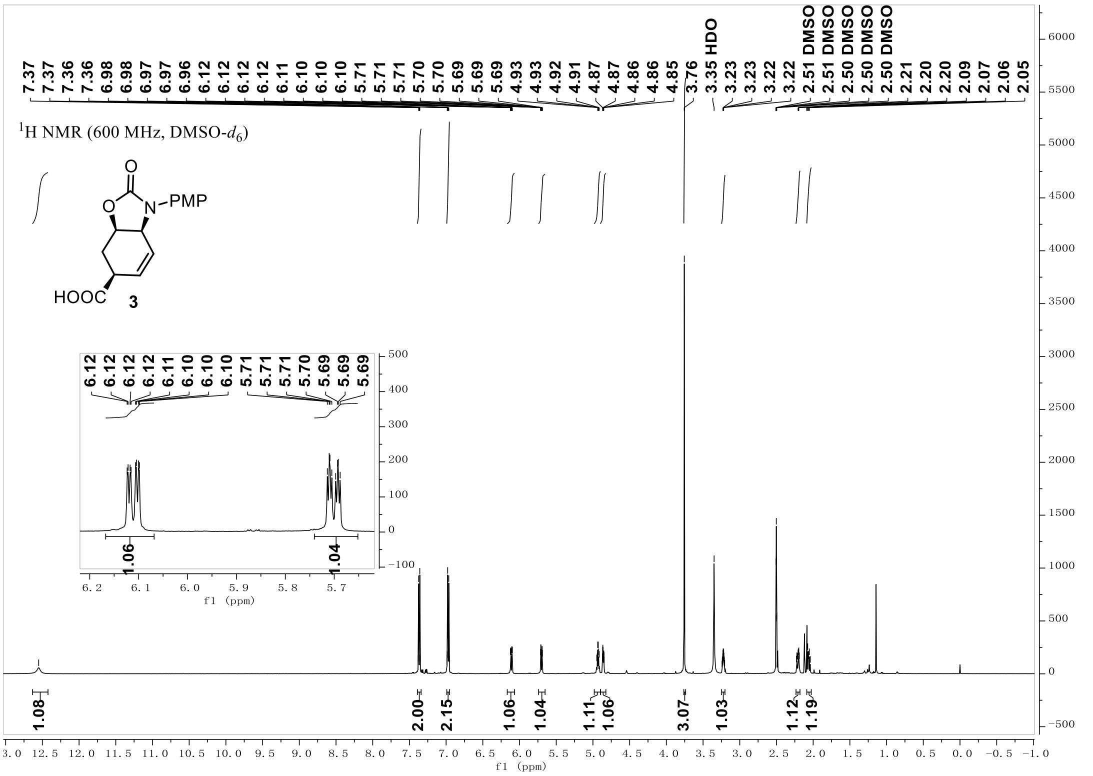

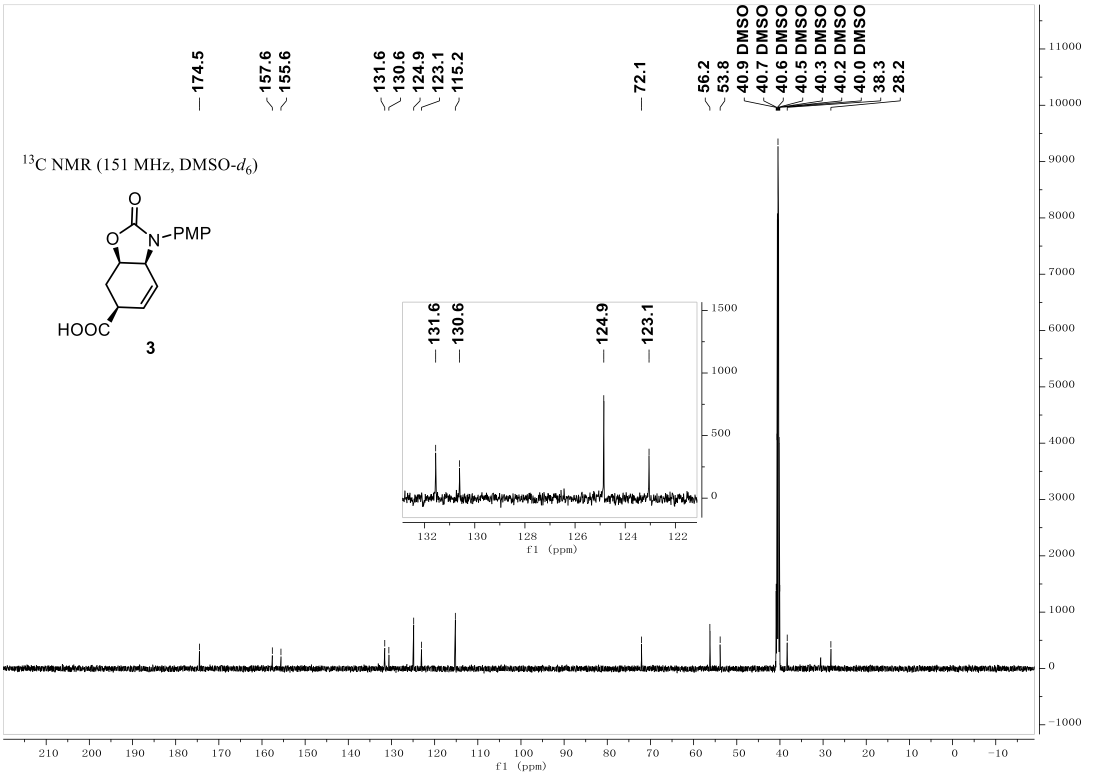

Supplementary Figure 4. ¹H NMR and ¹³C NMR spectra of compound 3.

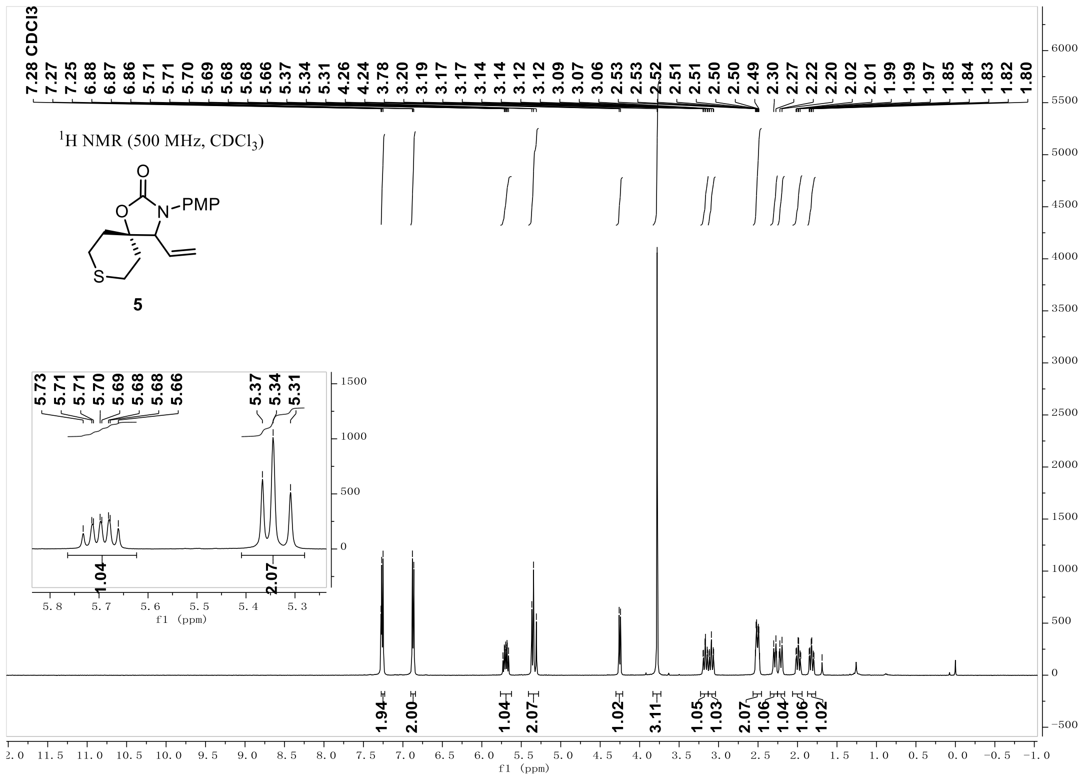

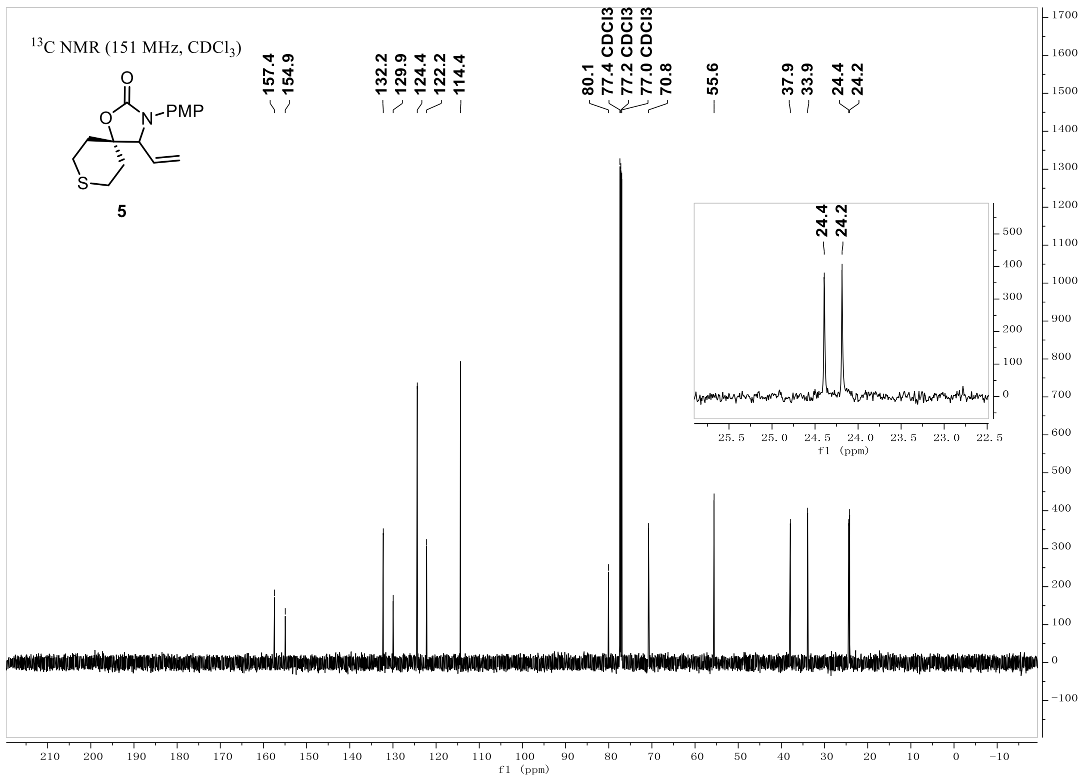

Supplementary Figure 5. ^{1}H NMR and ^{13}C NMR spectra of compound 5.

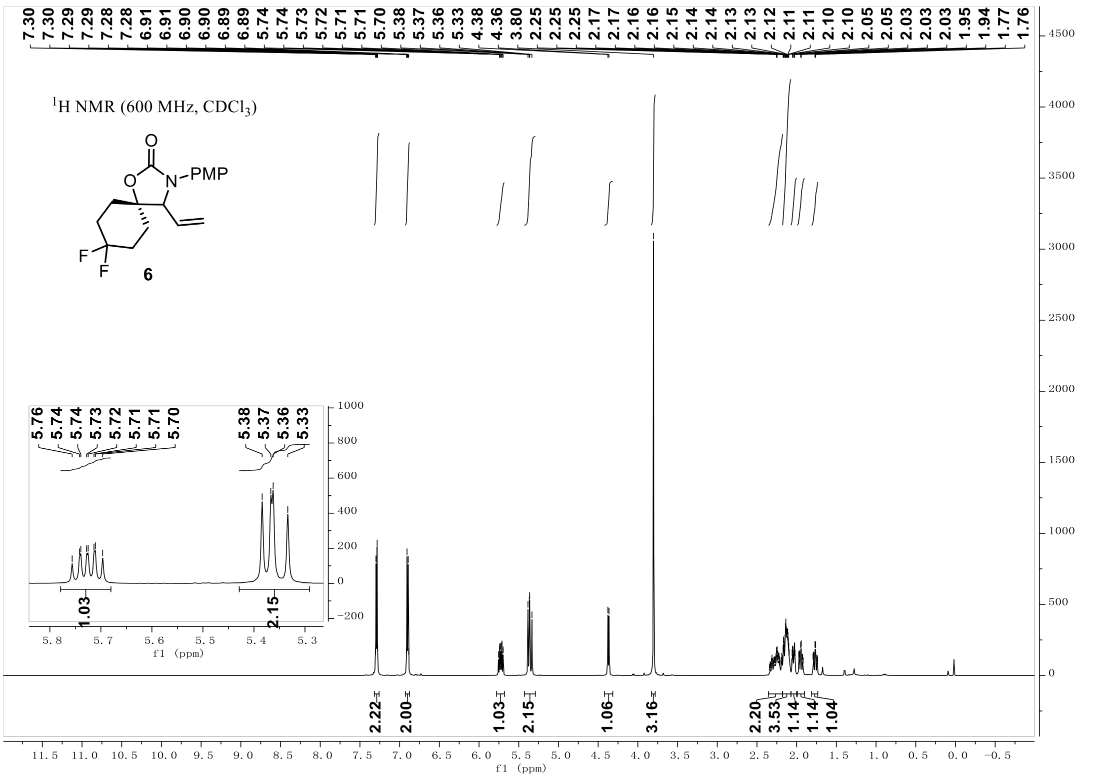

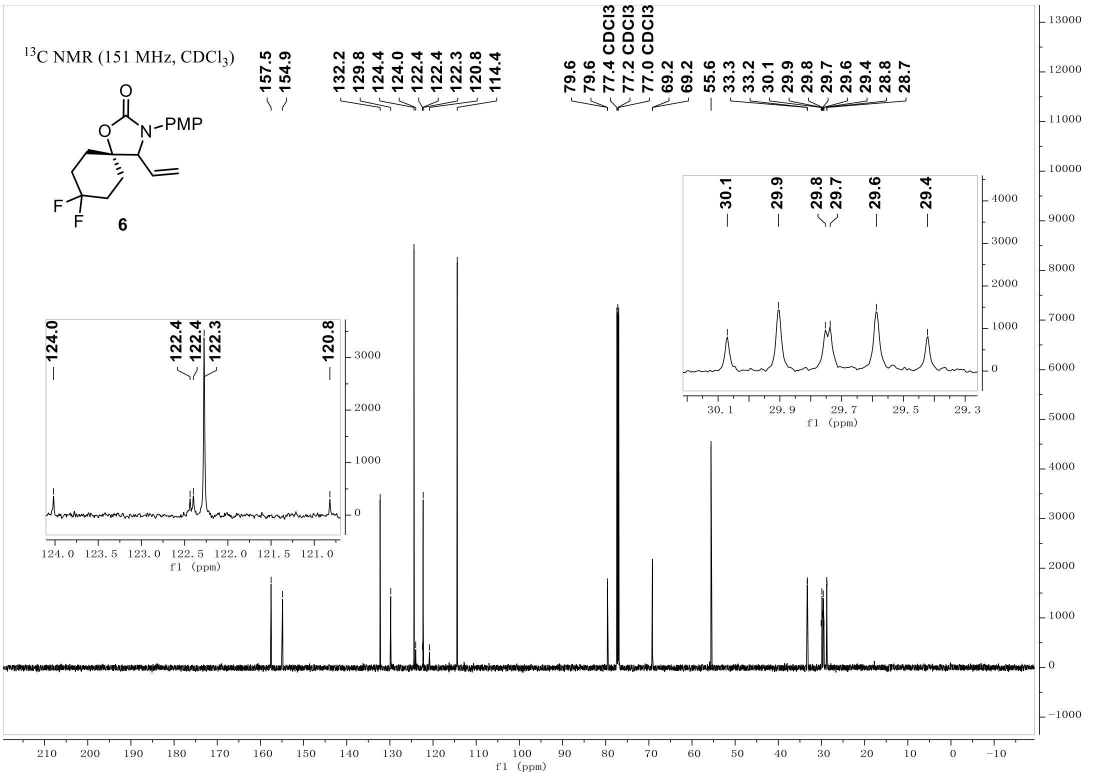

Supplementary Figure 6. ^{1}H NMR and ^{13}C NMR spectra of compound 6.

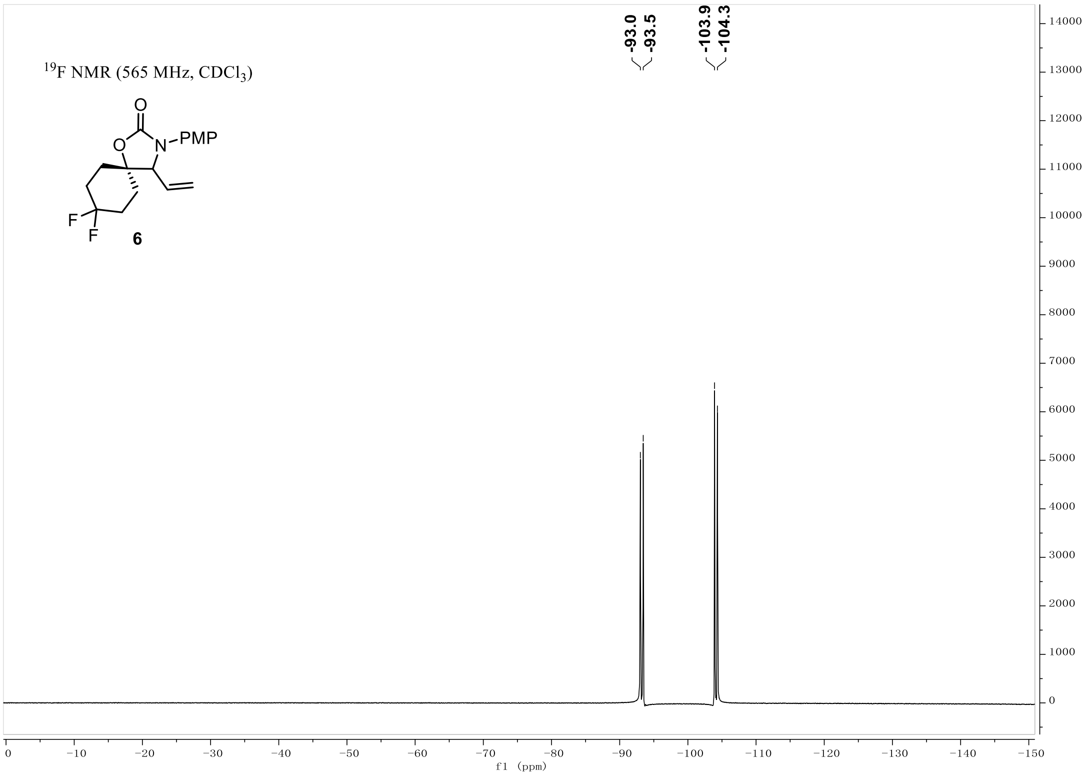

Supplementary Figure 7. ^{19}F NMR spectra of compound 6.

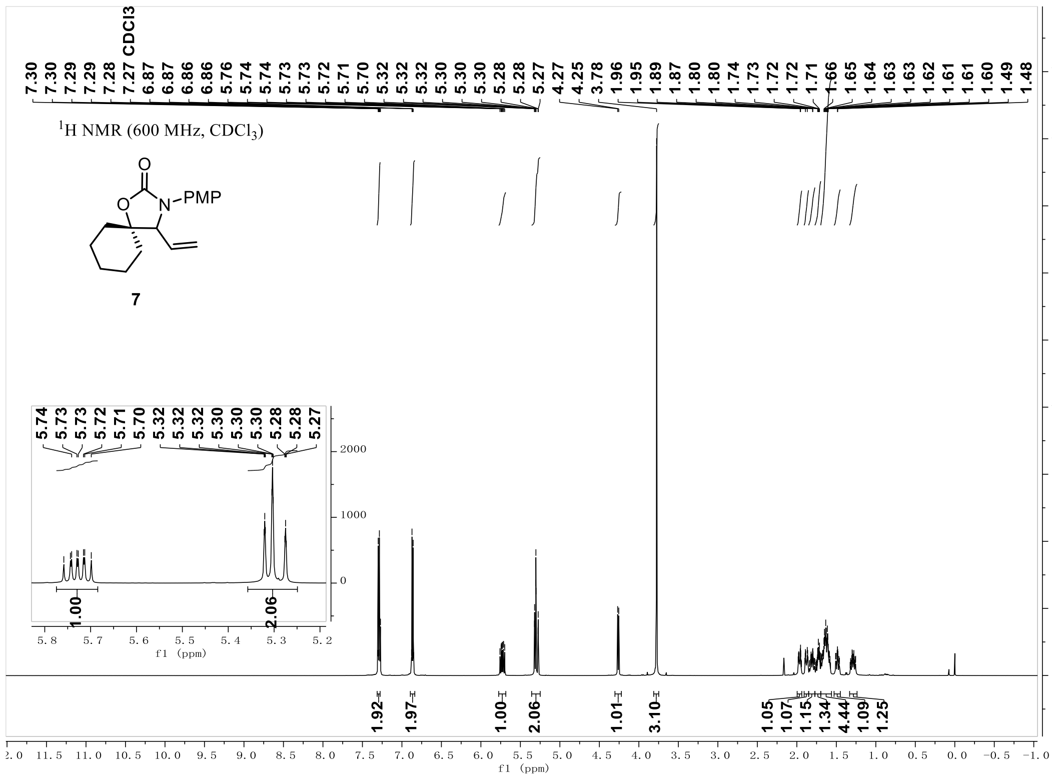

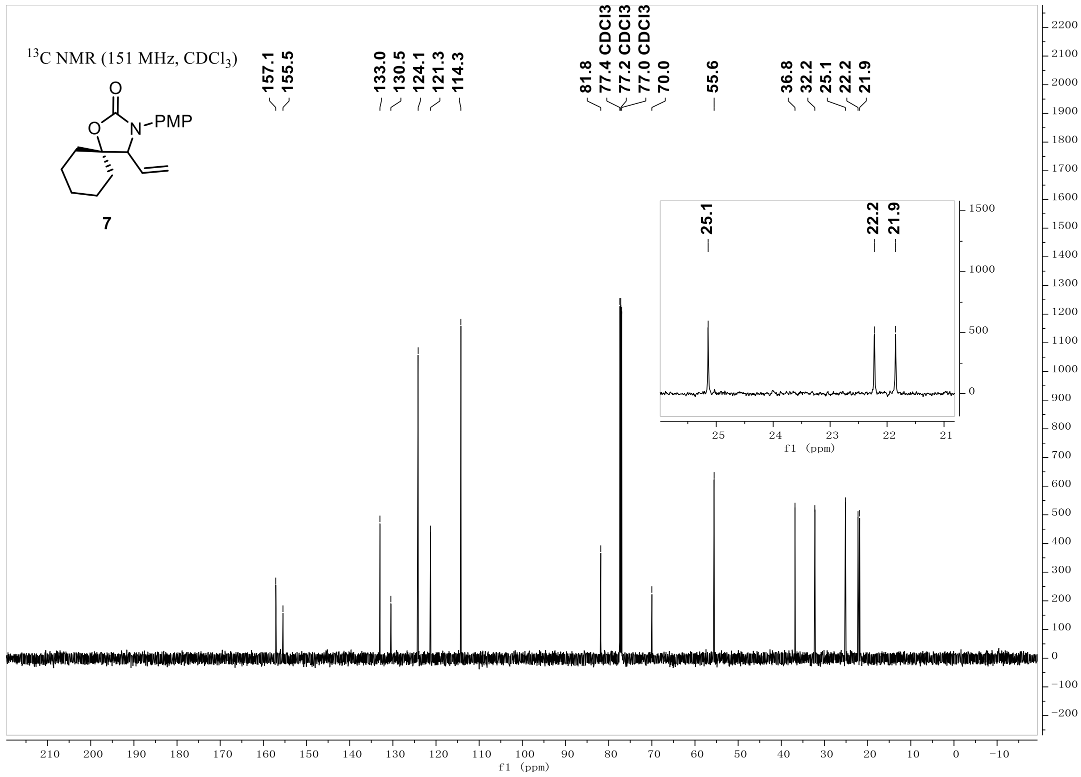

Supplementary Figure 8. ^{1}H NMR and ^{13}C NMR spectra of compound 7.

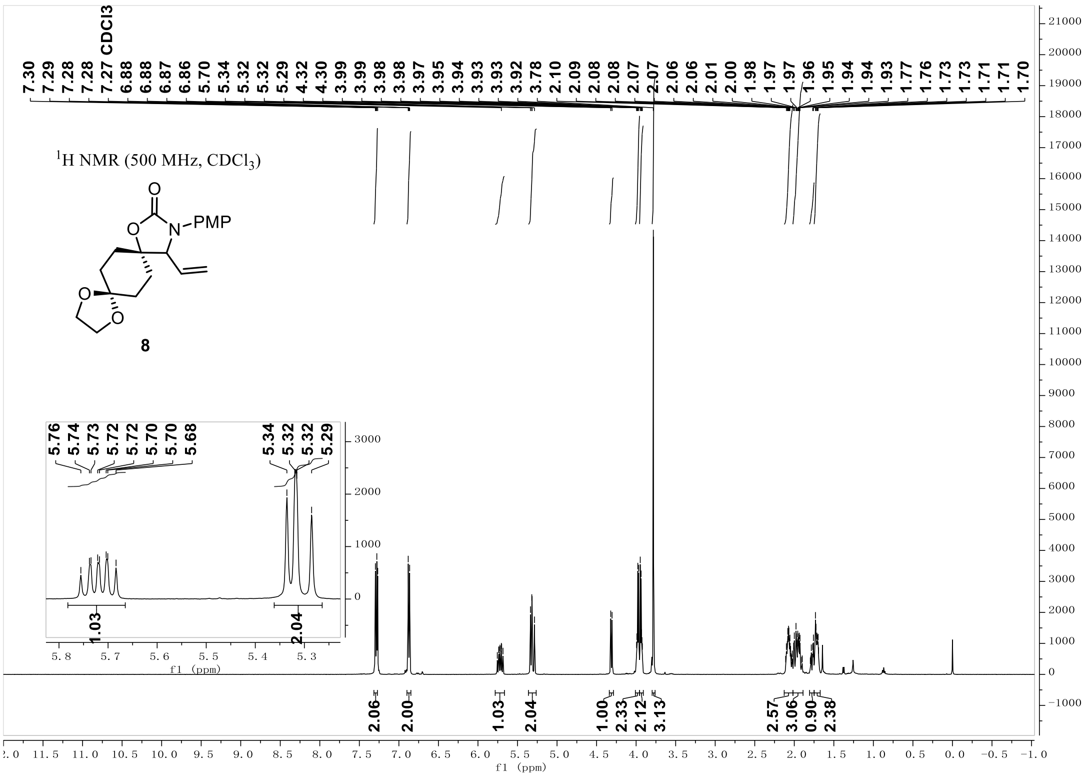

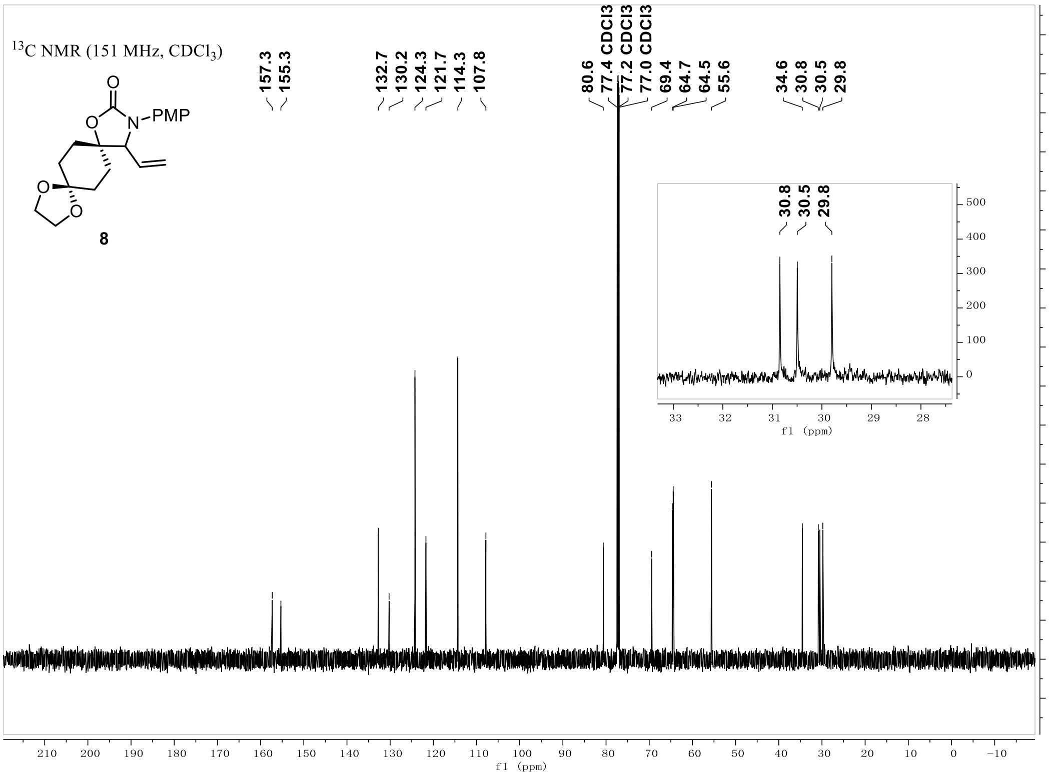

Supplementary Figure 9. ^{1}H NMR and ^{13}C NMR spectra of compound 8.

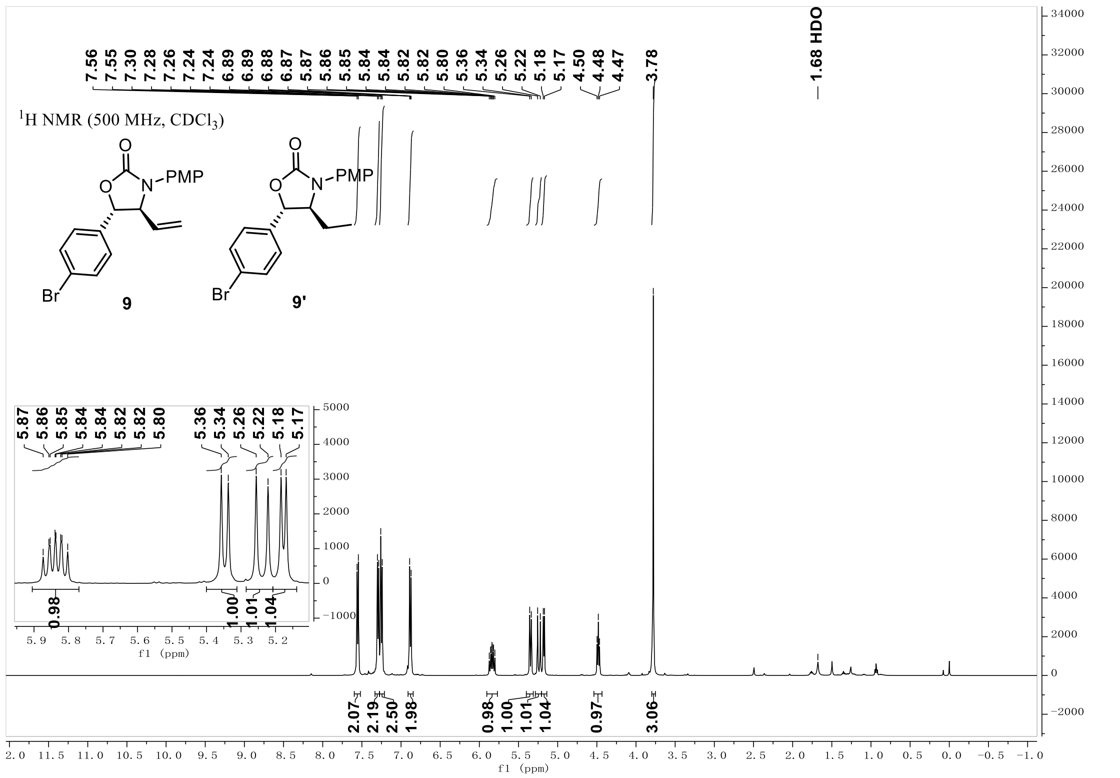

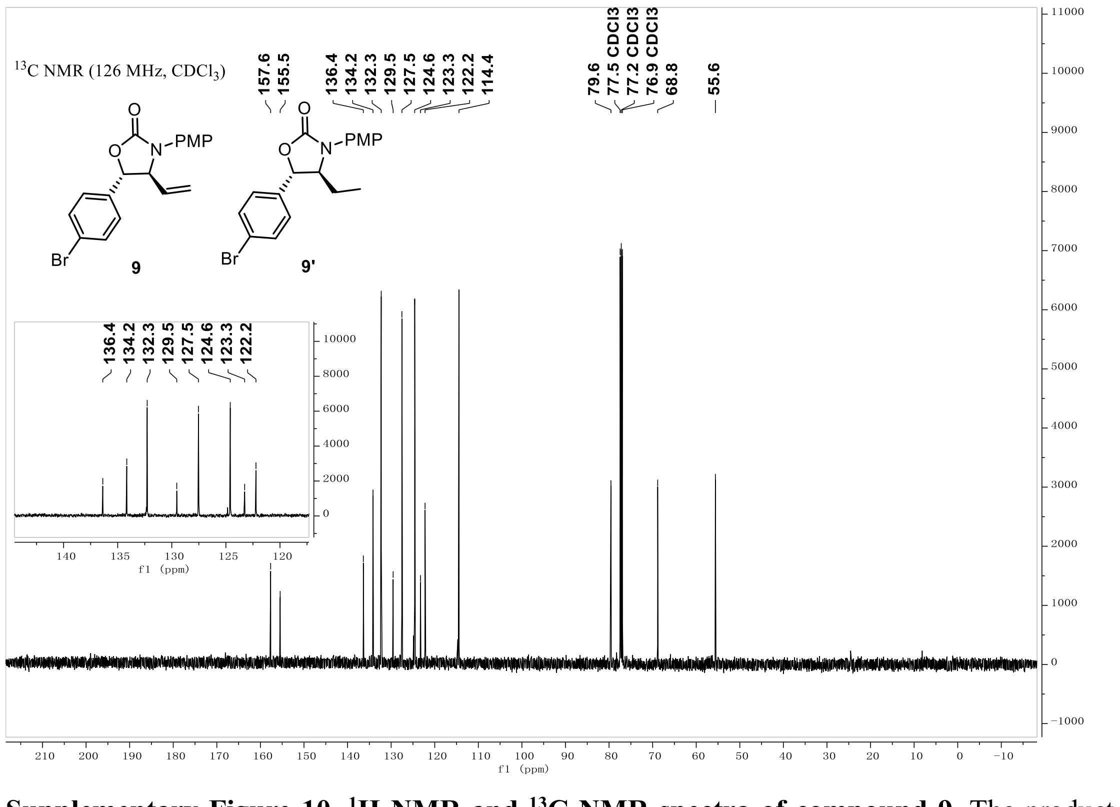

Supplementary Figure 10. ^{1}H NMR and ^{13}C NMR spectra of compound 9. The product was isolated as a mixture of **9** and **9'** in a ratio of 17:1.

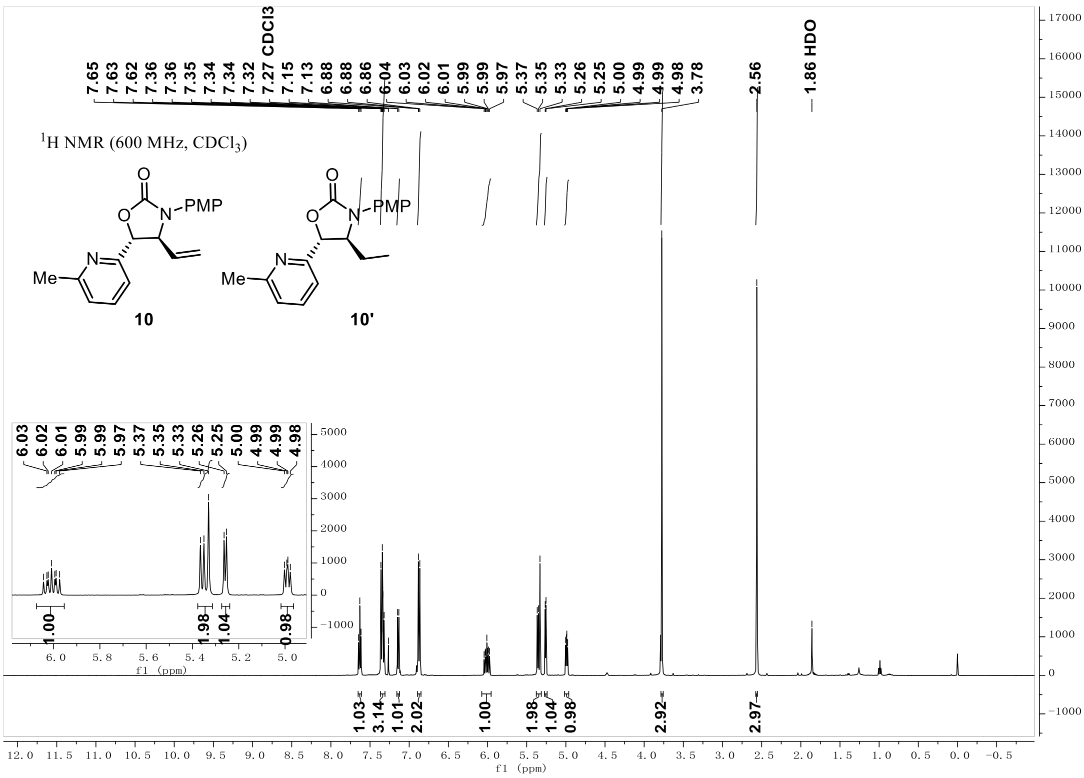

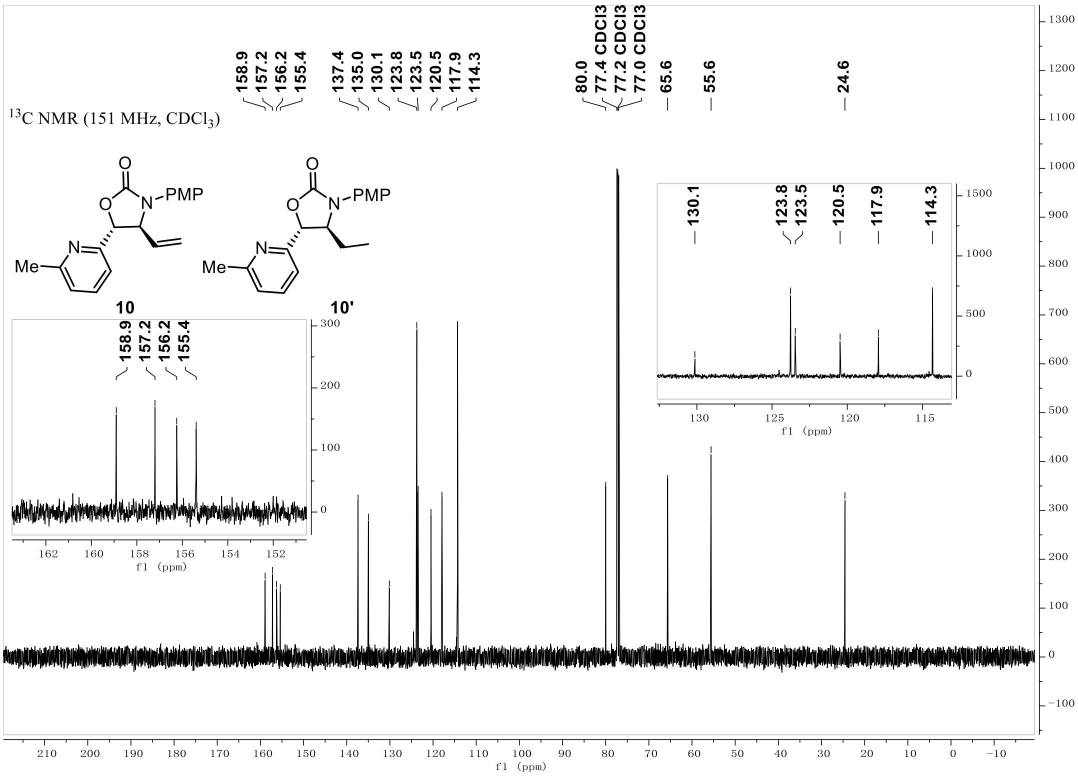

Supplementary Figure 11. ¹H NMR and ¹³C NMR spectra of compound 10. The product was isolated as a mixture of **10** and **10'** in a ratio of 14:1.

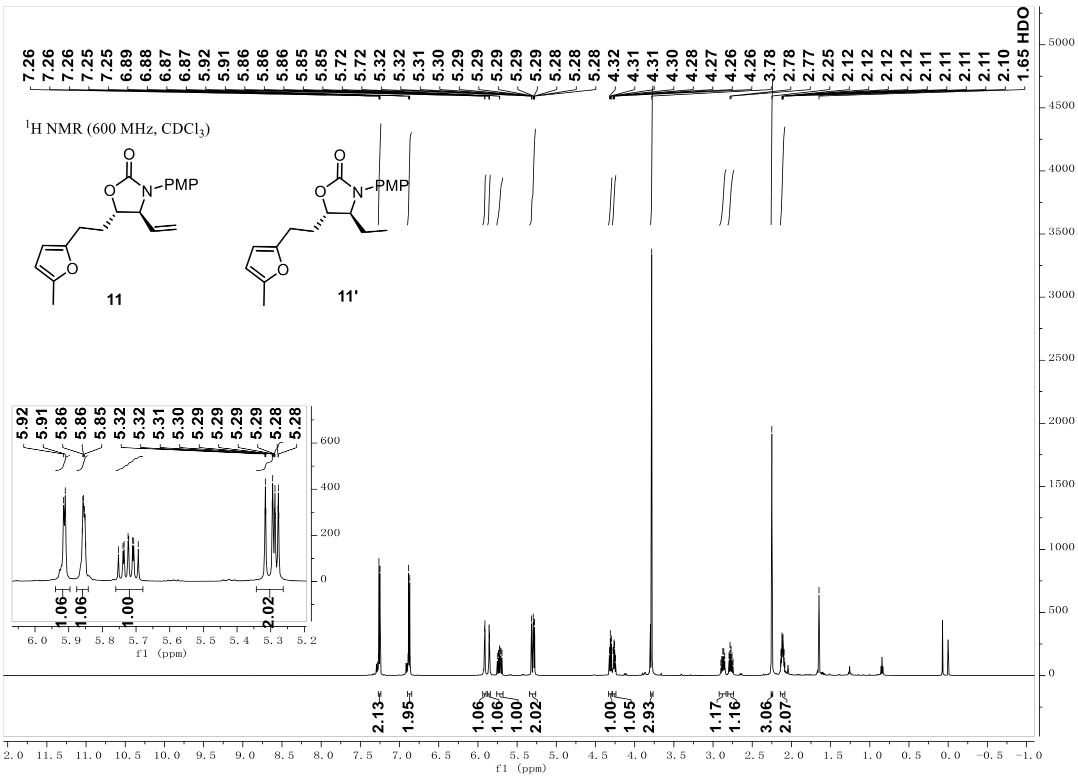

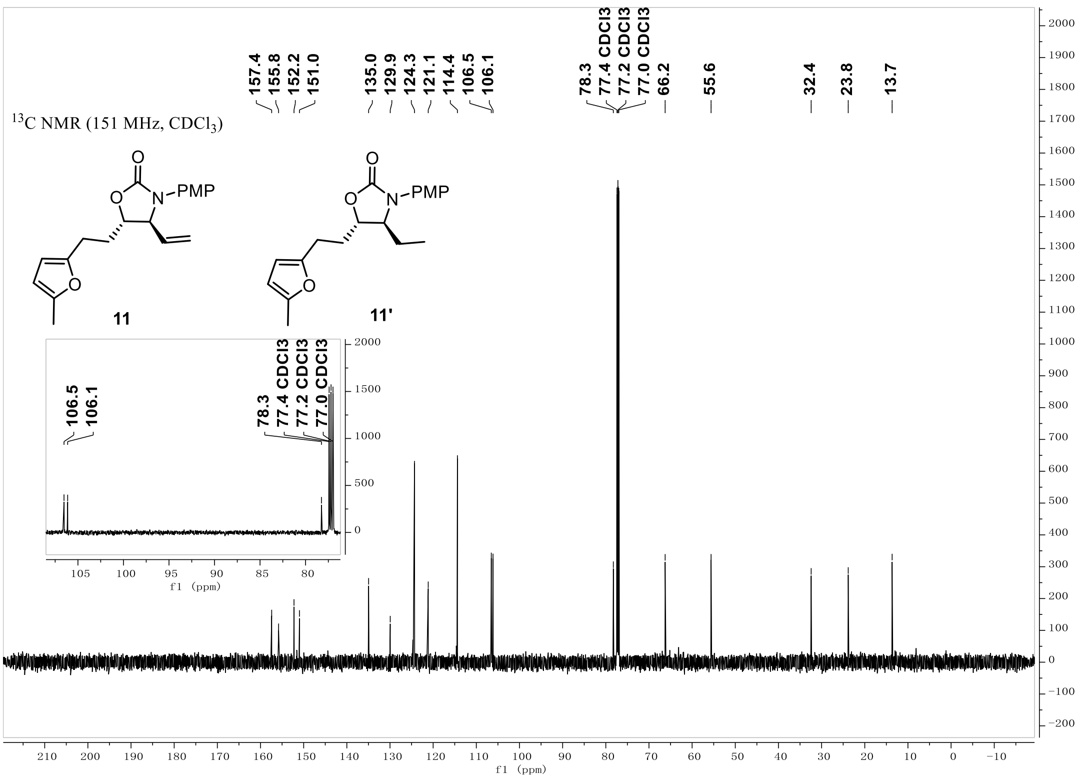

Supplementary Figure 12. ^{1}H NMR and ^{13}C NMR spectra of compound 11. The product was isolated as a mixture of **11** and **11'** in a ratio of 9:1.

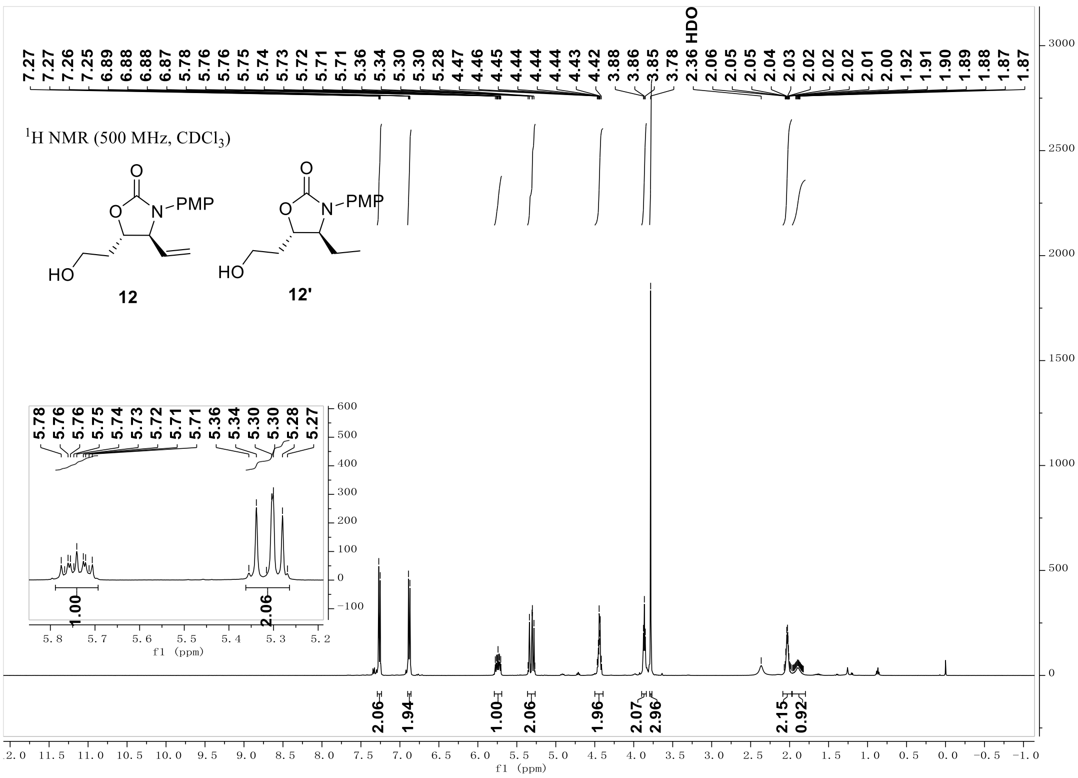

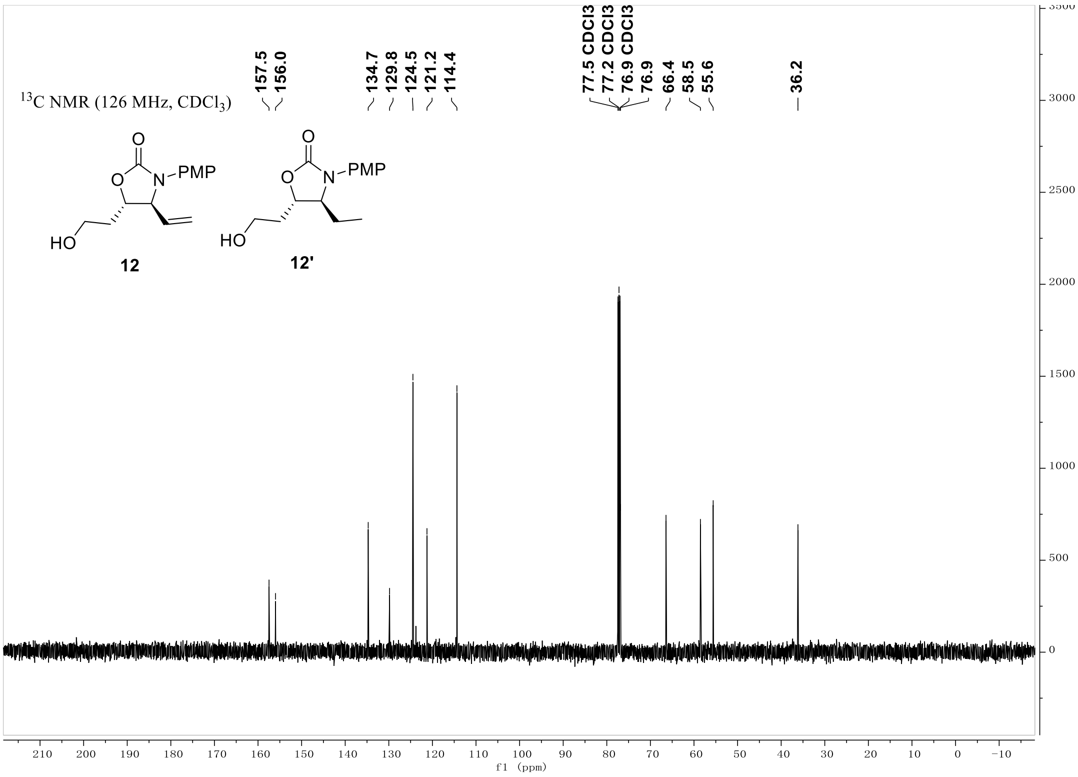

Supplementary Figure 13. ^{1}H NMR and ^{13}C NMR spectra of compound 12. The product was isolated as a mixture of **12** and **12'** in a ratio of 21:1.

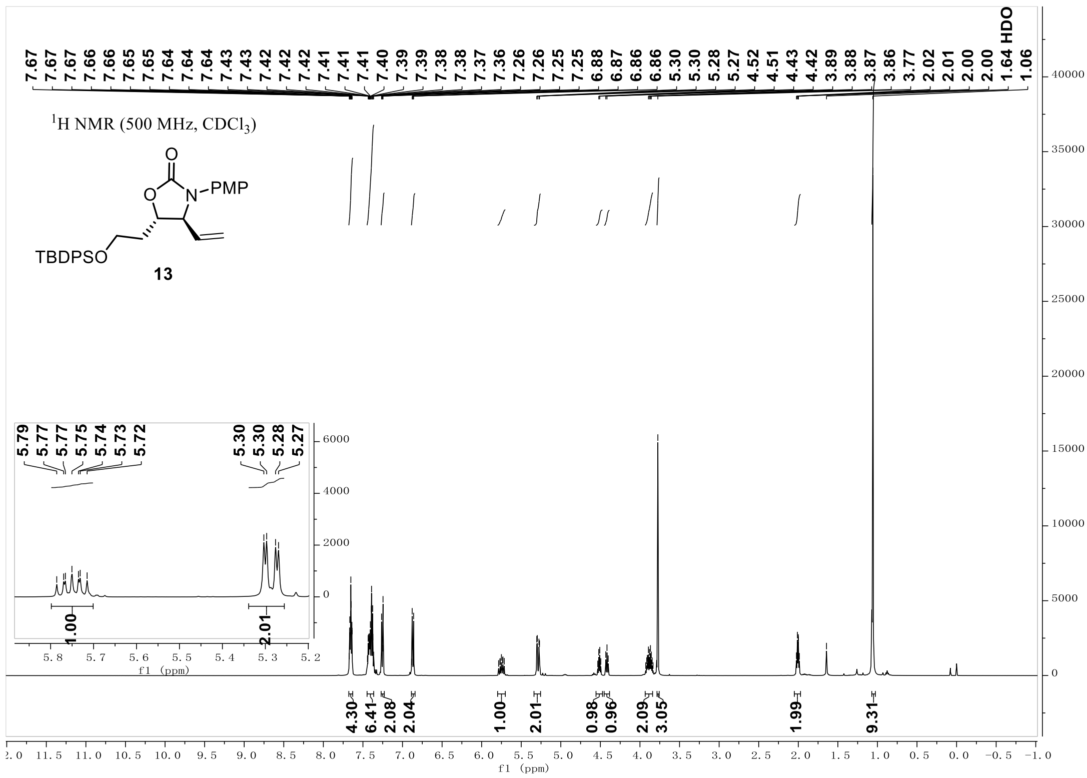

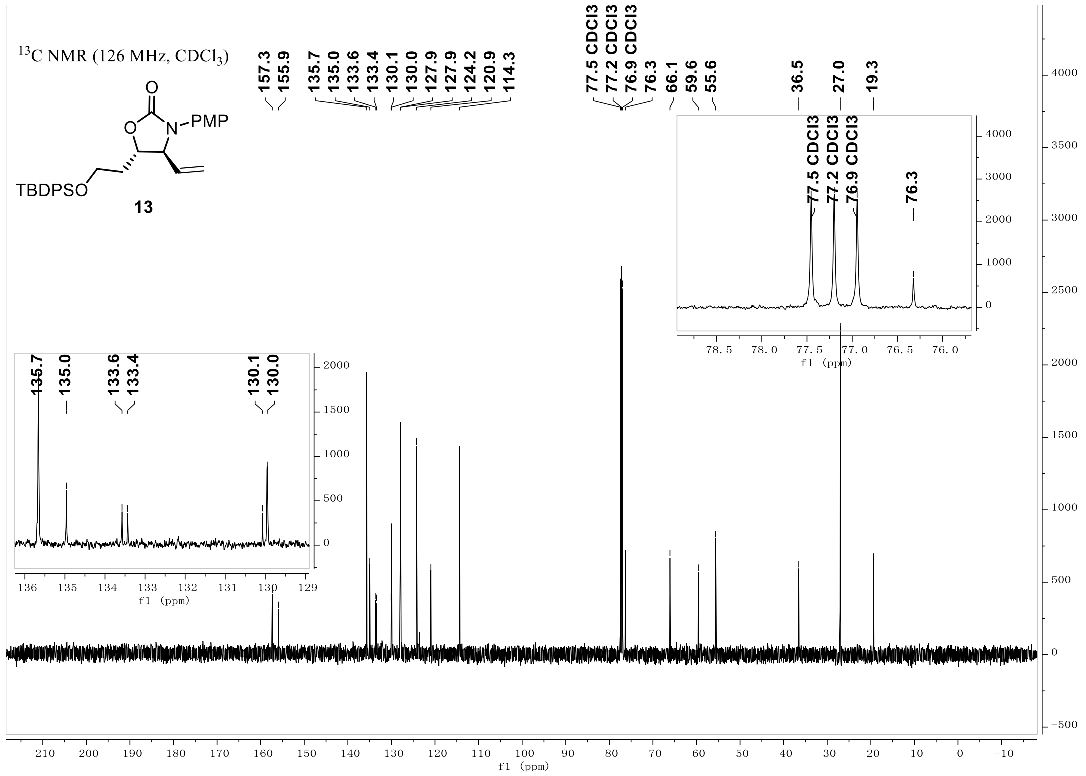

Supplementary Figure 14. ^{1}H NMR and ^{13}C NMR spectra of compound 13.

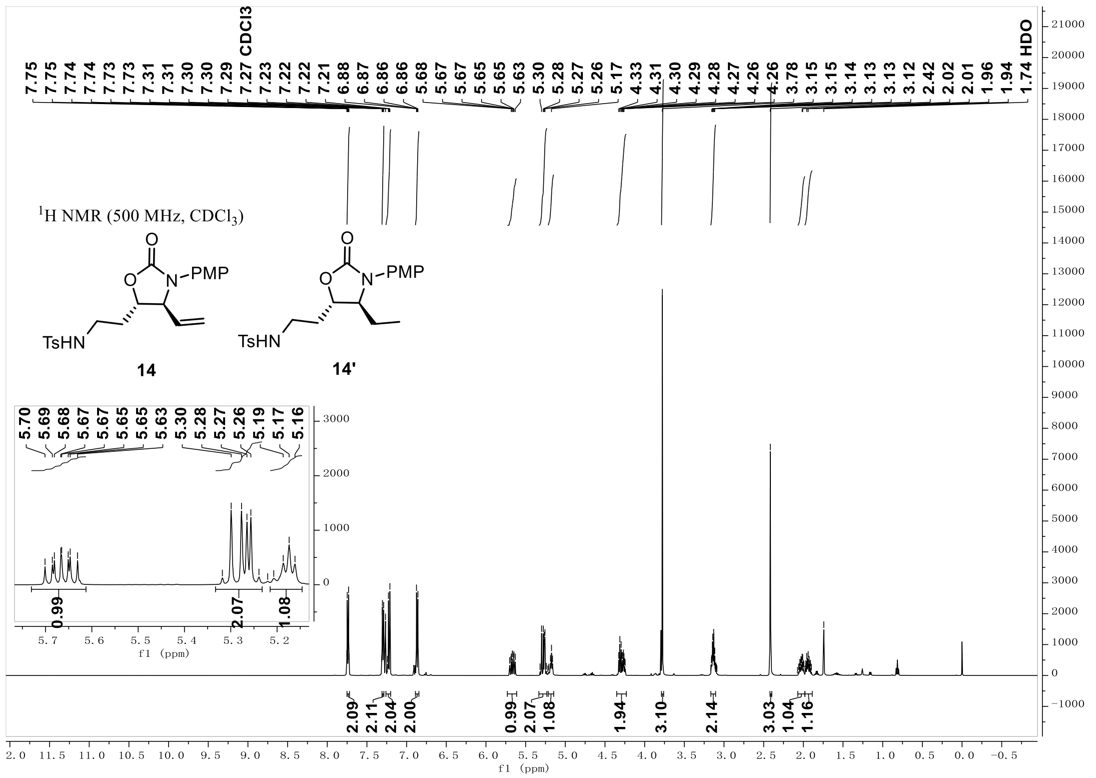

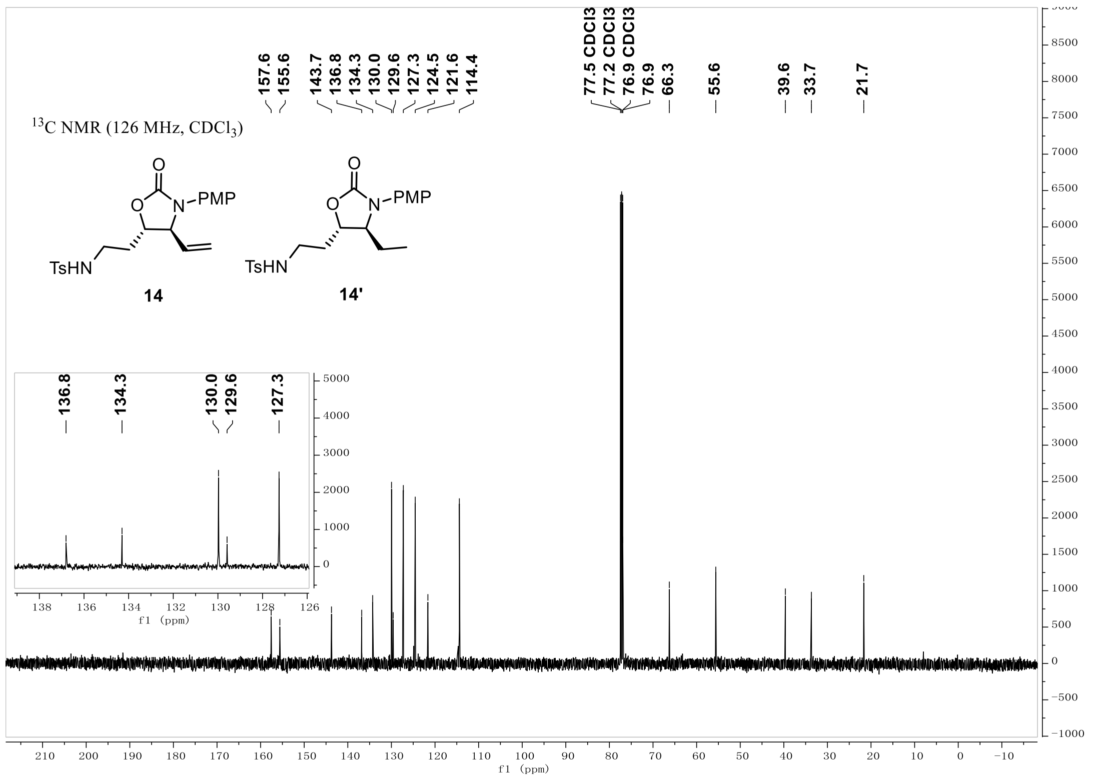

Supplementary Figure 15. ^{1}H NMR and ^{13}C NMR spectra of compound 14. The product was isolated as a mixture of **14** and **14'** in a ratio of 8:1.

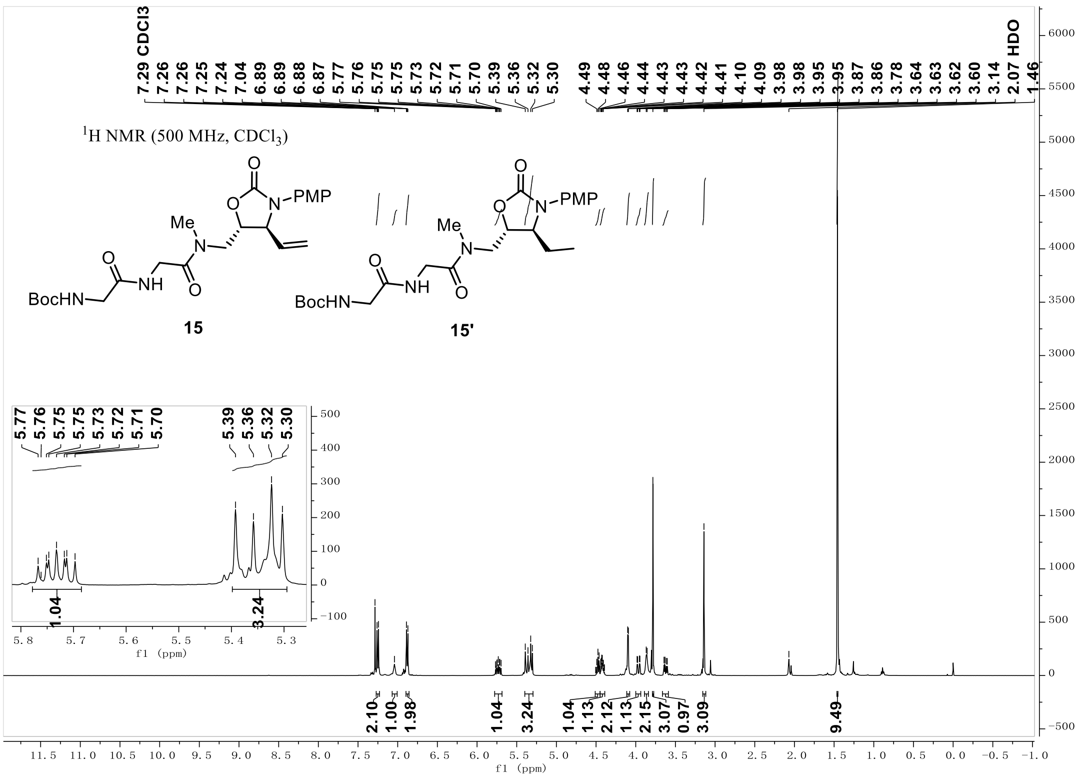

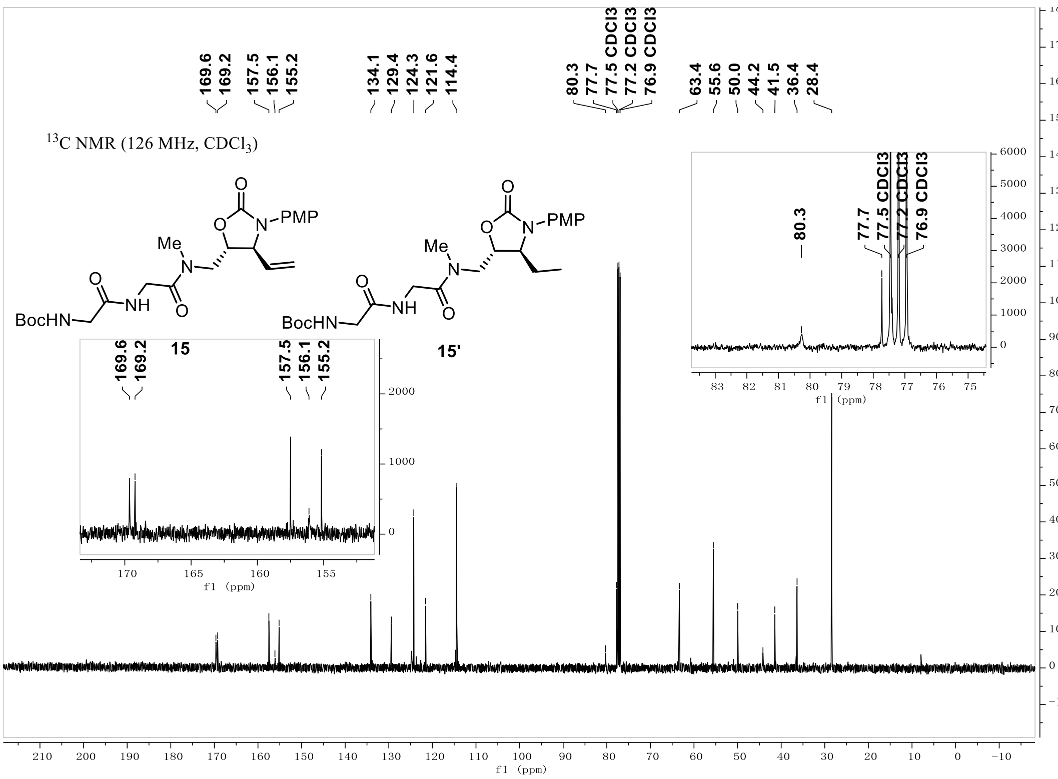

Supplementary Figure 16. ^{1}H NMR and ^{13}C NMR spectra of compound 15. The product was isolated as a mixture of **15** and **15'** in a ratio of 8:1.

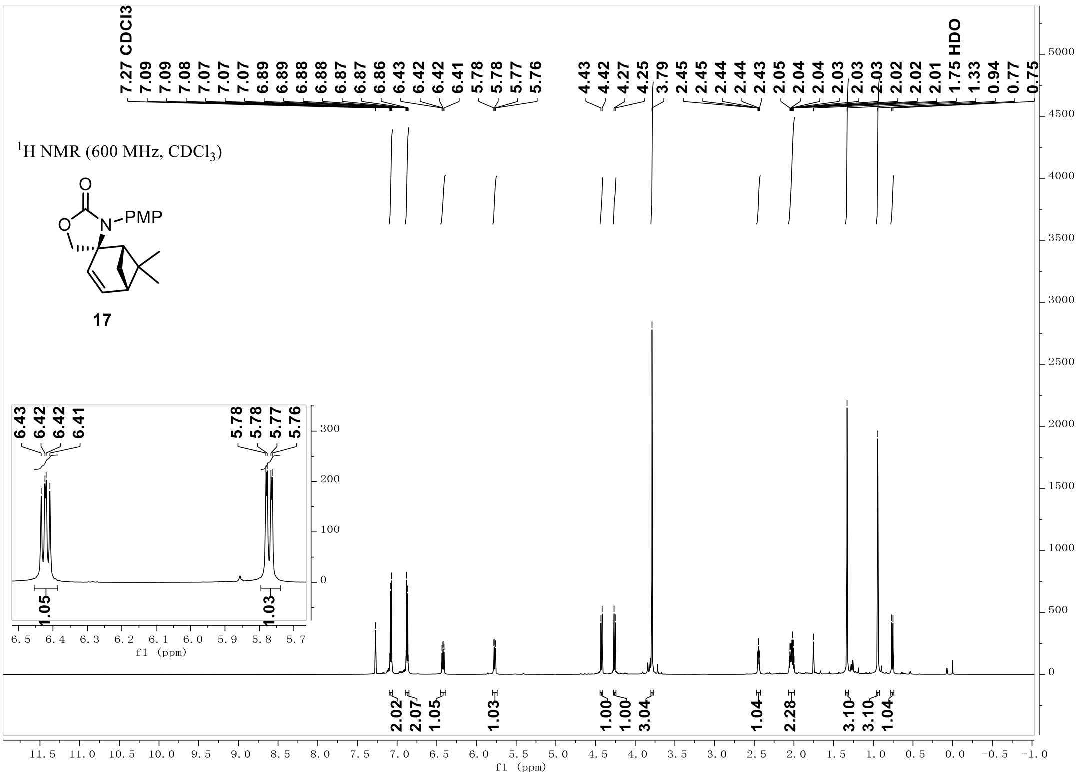

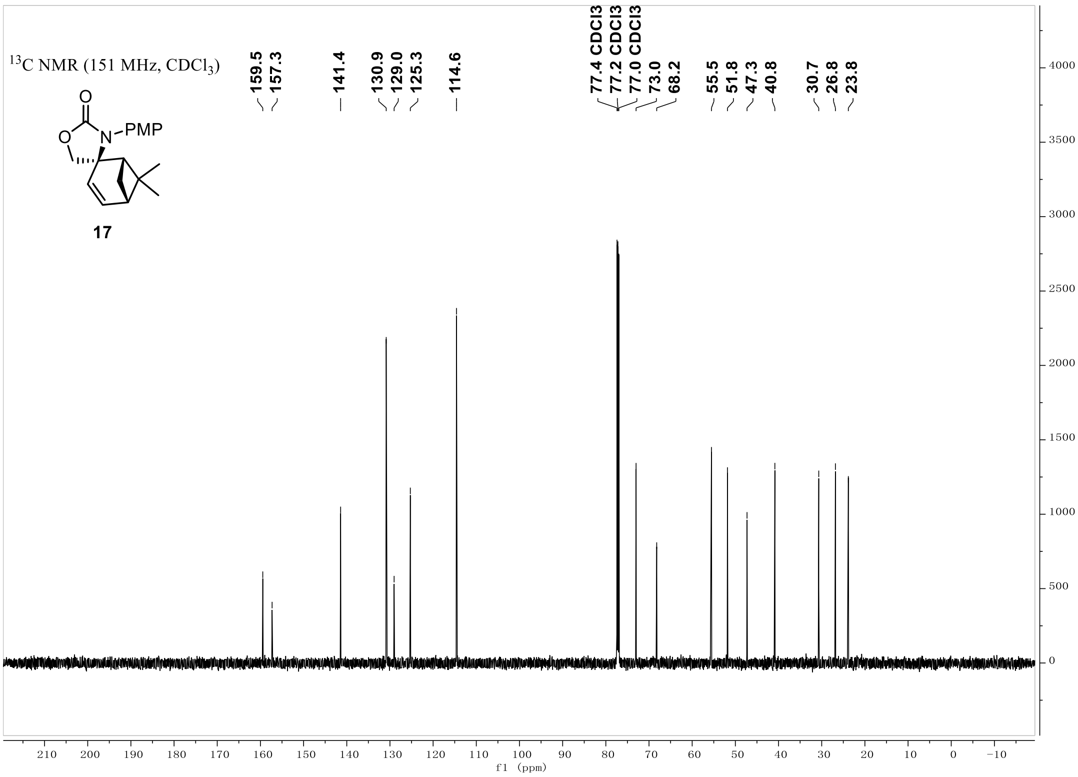

Supplementary Figure 17. ^{1}H NMR and ^{13}C NMR spectra of compound 17.

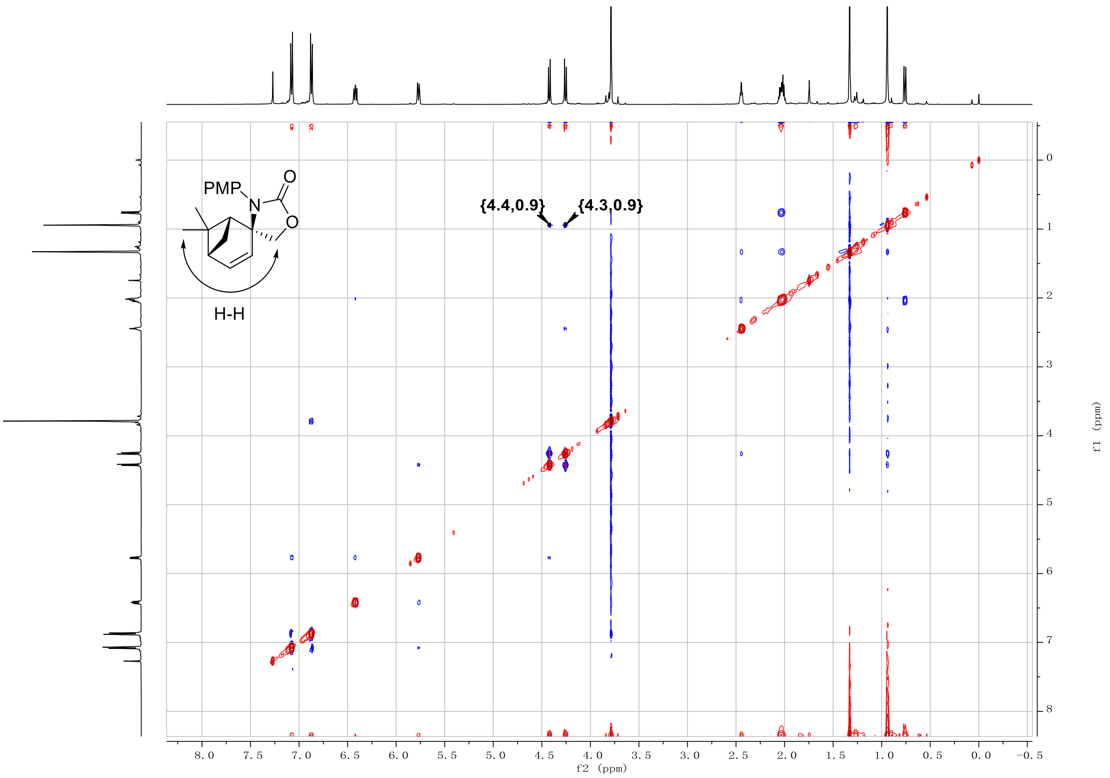

Supplementary Figure 18. 2D NOESY spectra of compound 17.

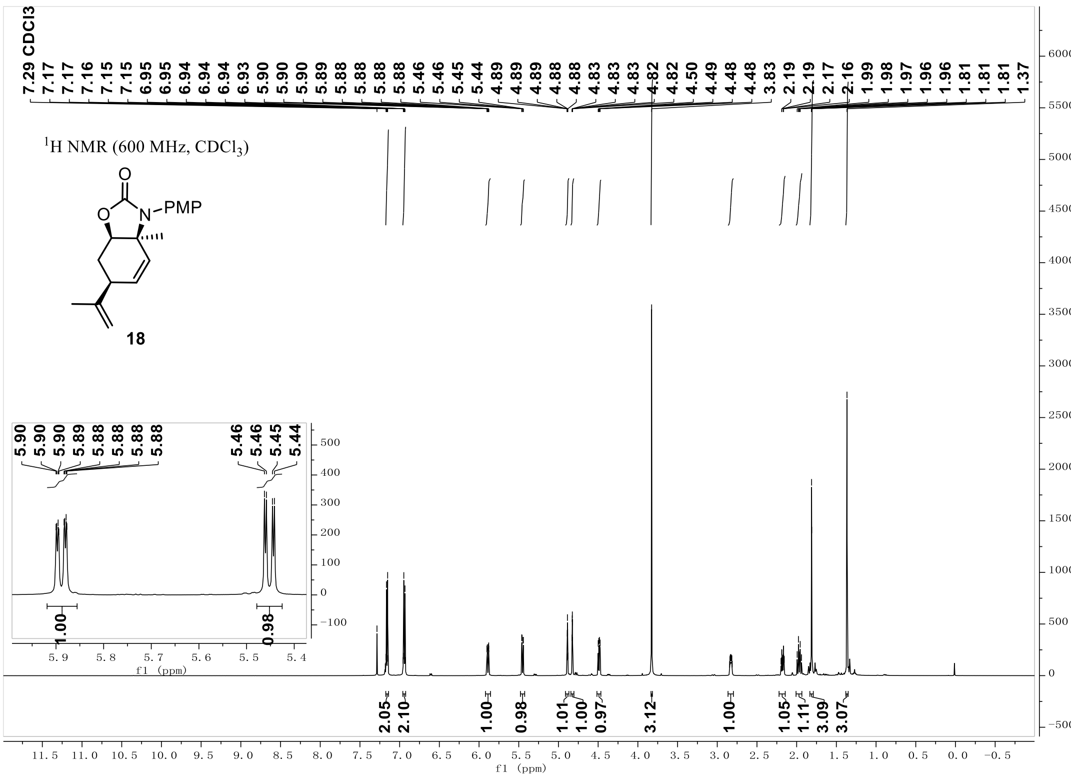

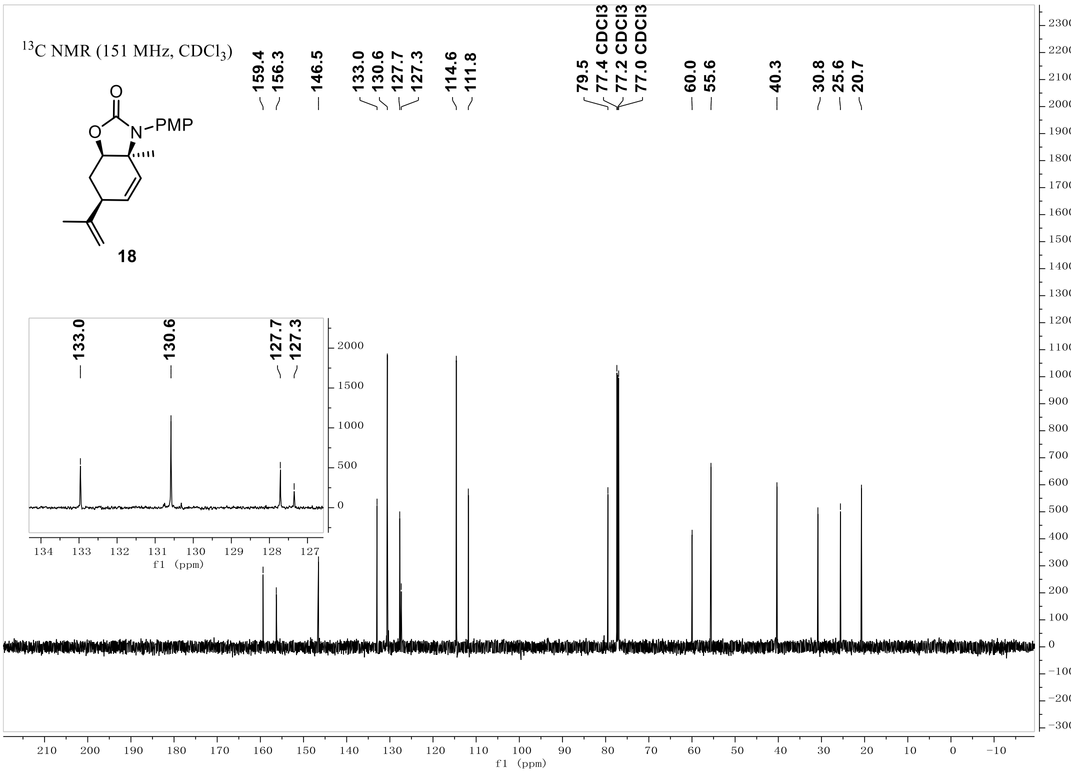

Supplementary Figure 19. ^{1}H NMR and ^{13}C NMR spectra of compound 18.

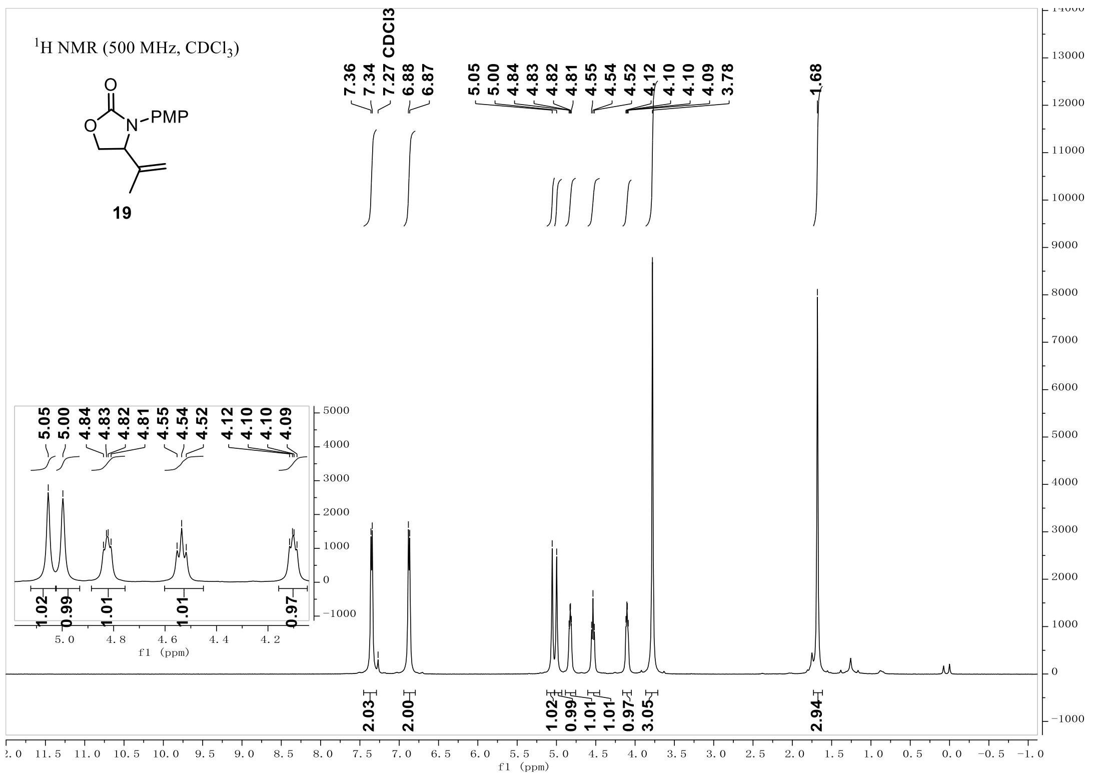

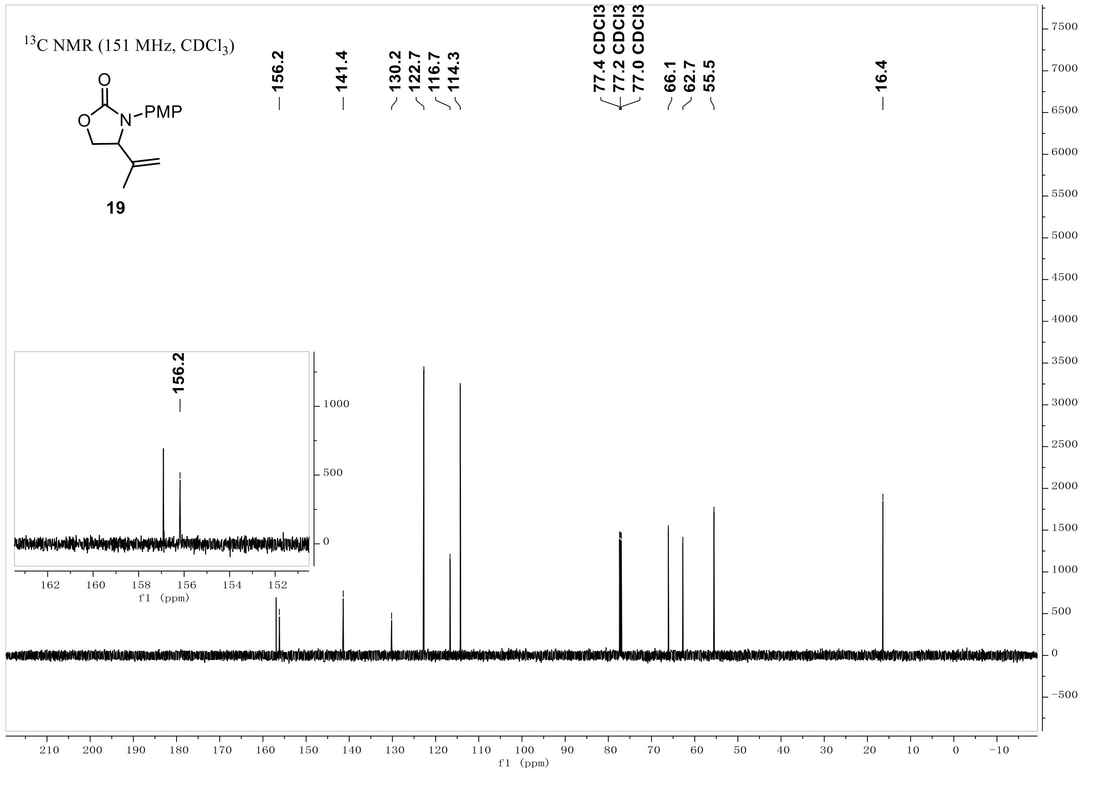

Supplementary Figure 20. ^{1}H NMR and ^{13}C NMR spectra of compound 19.

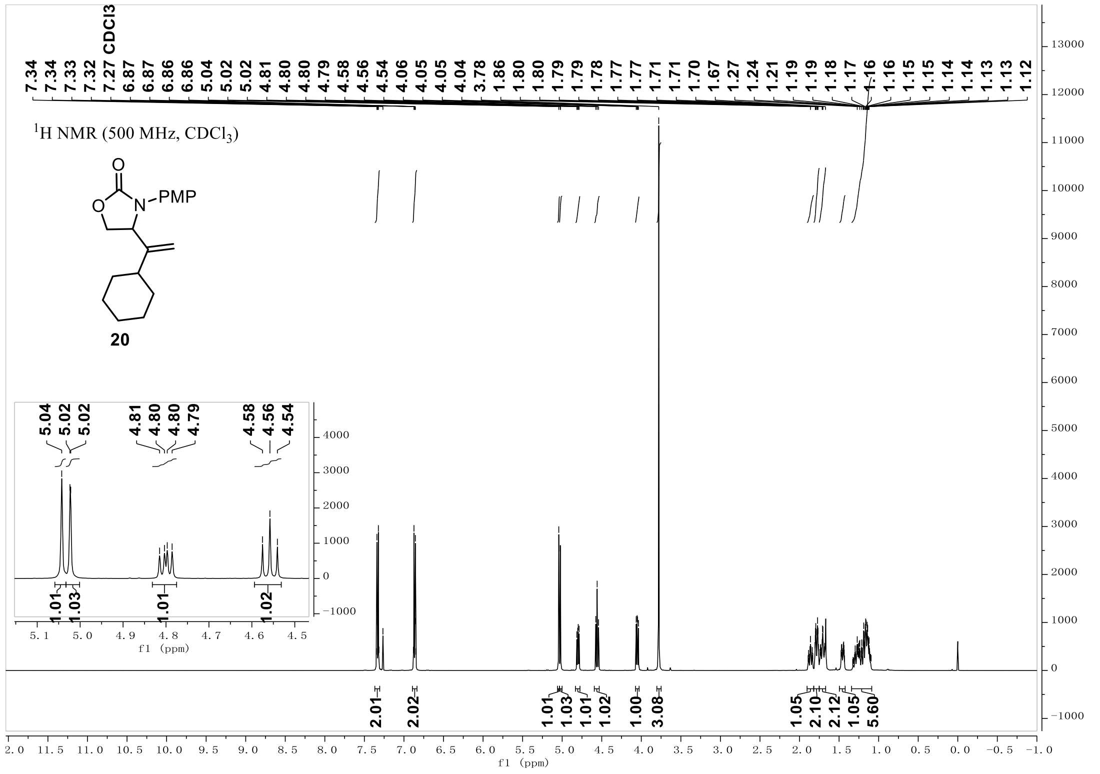

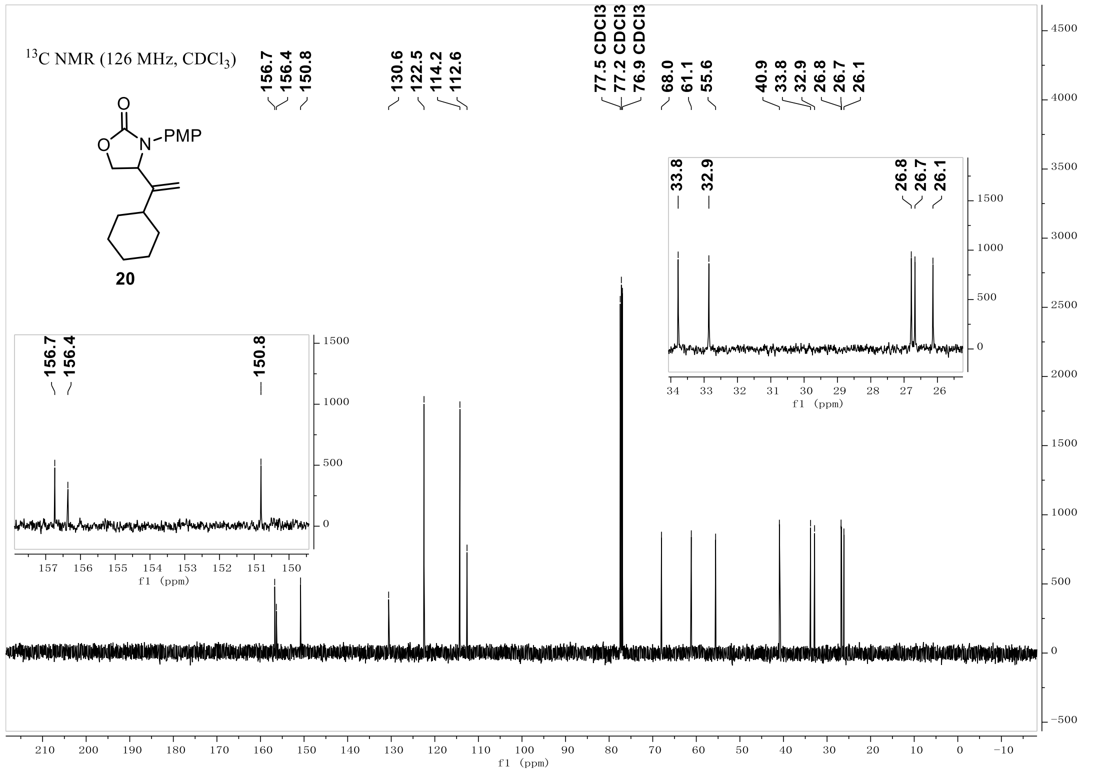

Supplementary Figure 21. ^{1}H NMR and ^{13}C NMR spectra of compound 20.

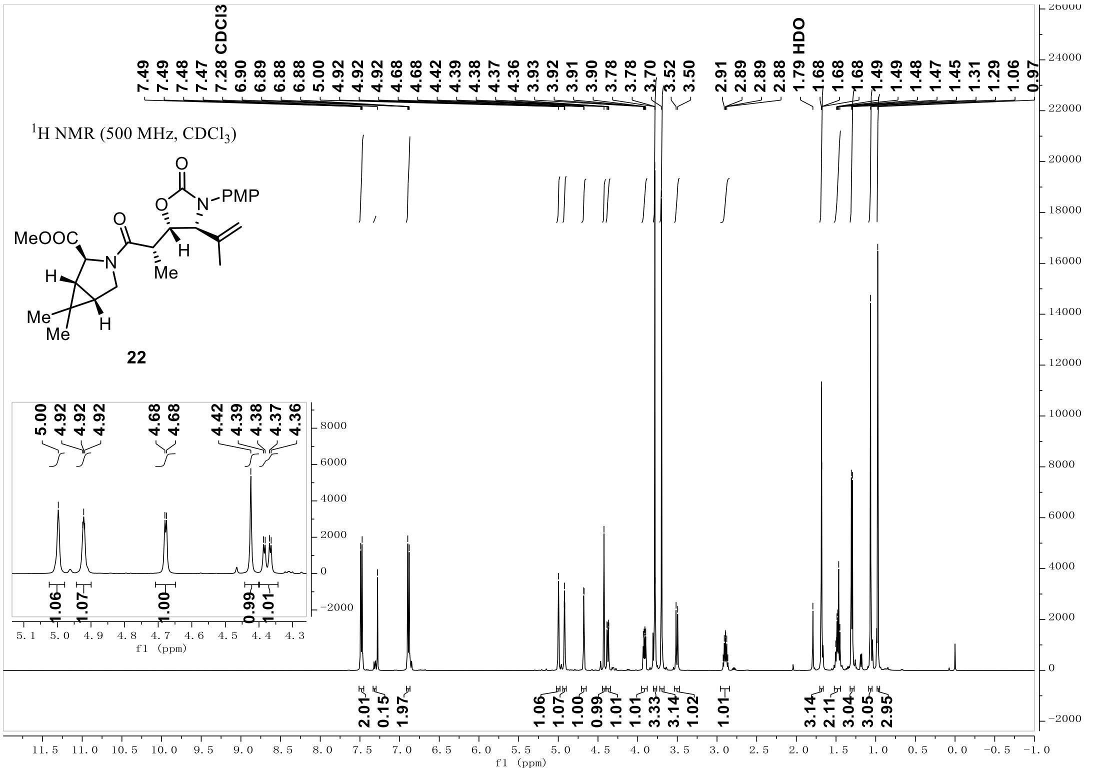

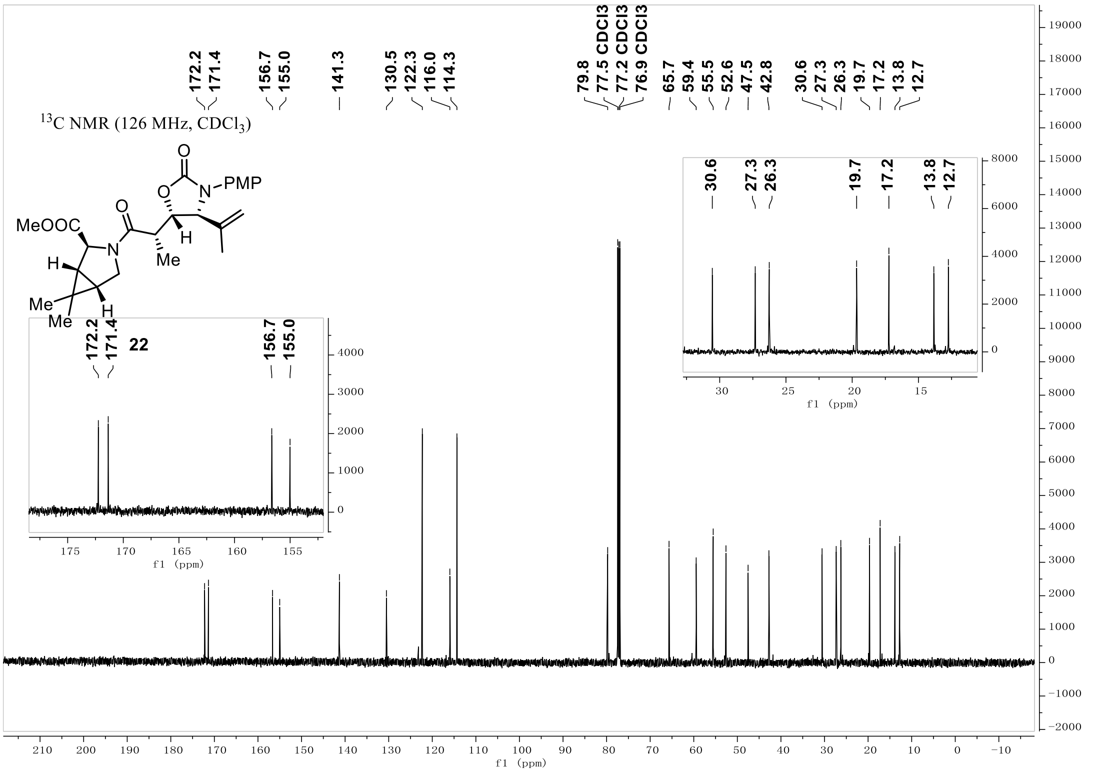

Supplementary Figure 22. ^{1}H NMR and ^{13}C NMR spectra of compound 22. The product was isolated as a 15:1 mixture of diastereomers.

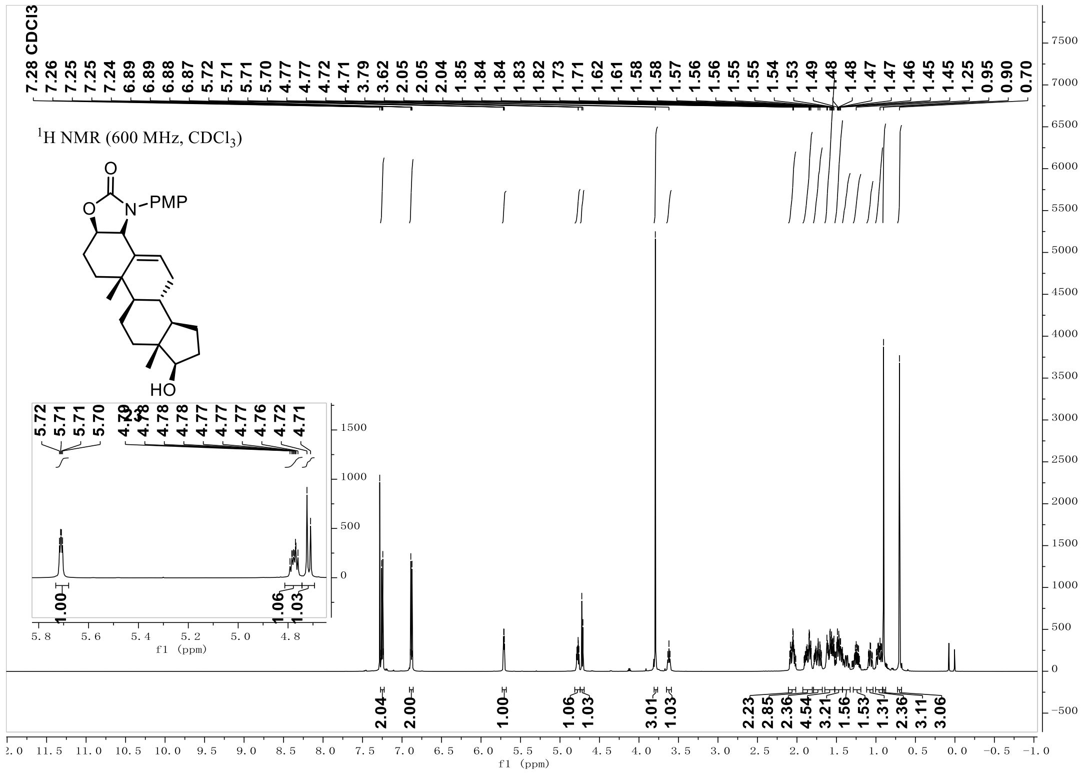

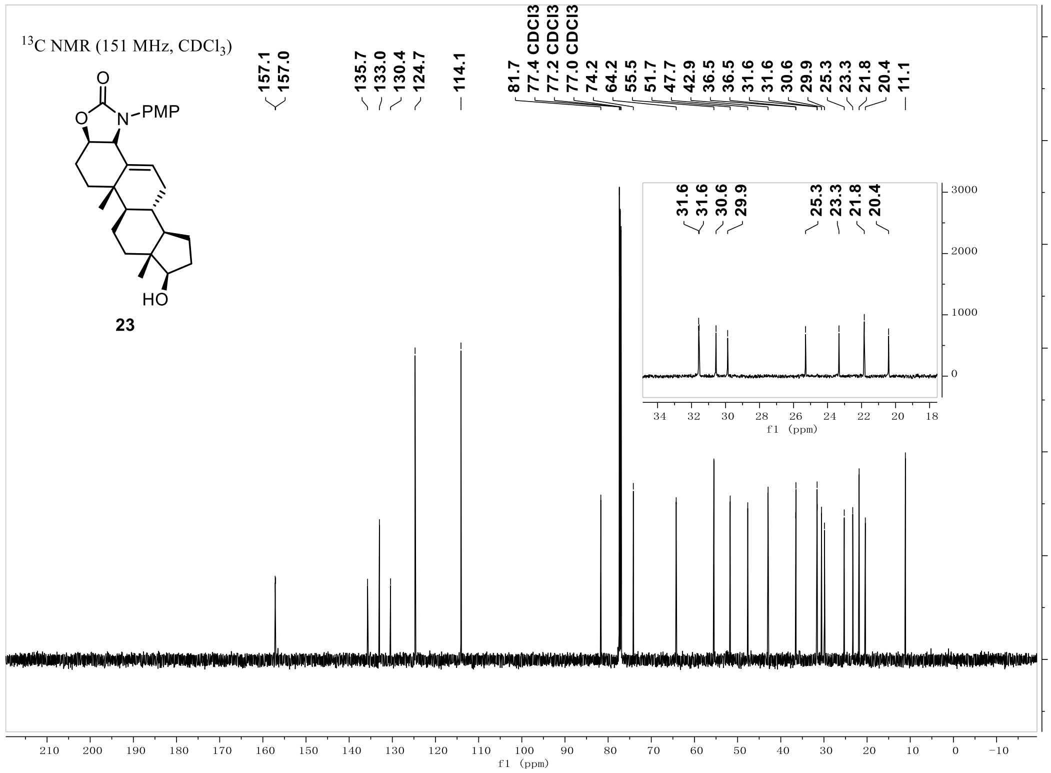

Supplementary Figure 23. ^{1}H NMR and ^{13}C NMR spectra of compound 23.

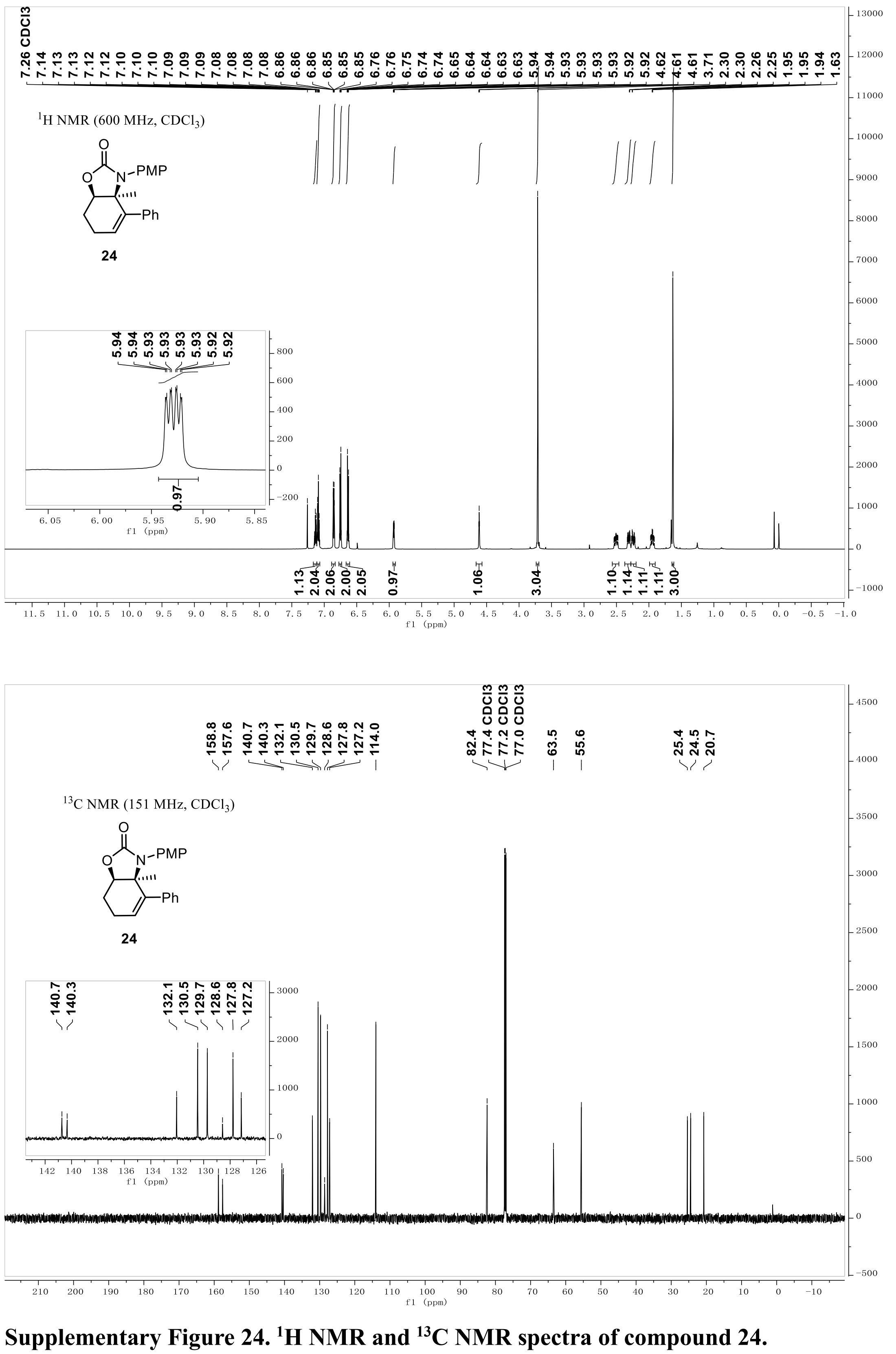

Supplementary Figure 24. ^{1}H NMR and ^{13}C NMR spectra of compound 24.

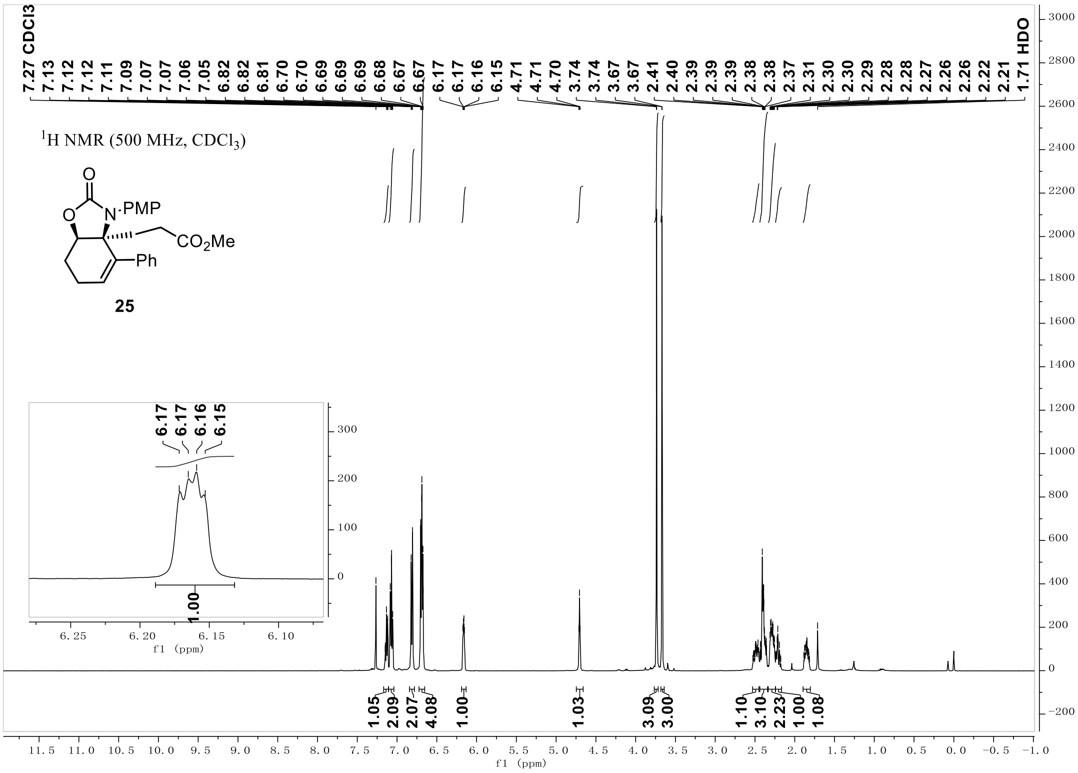

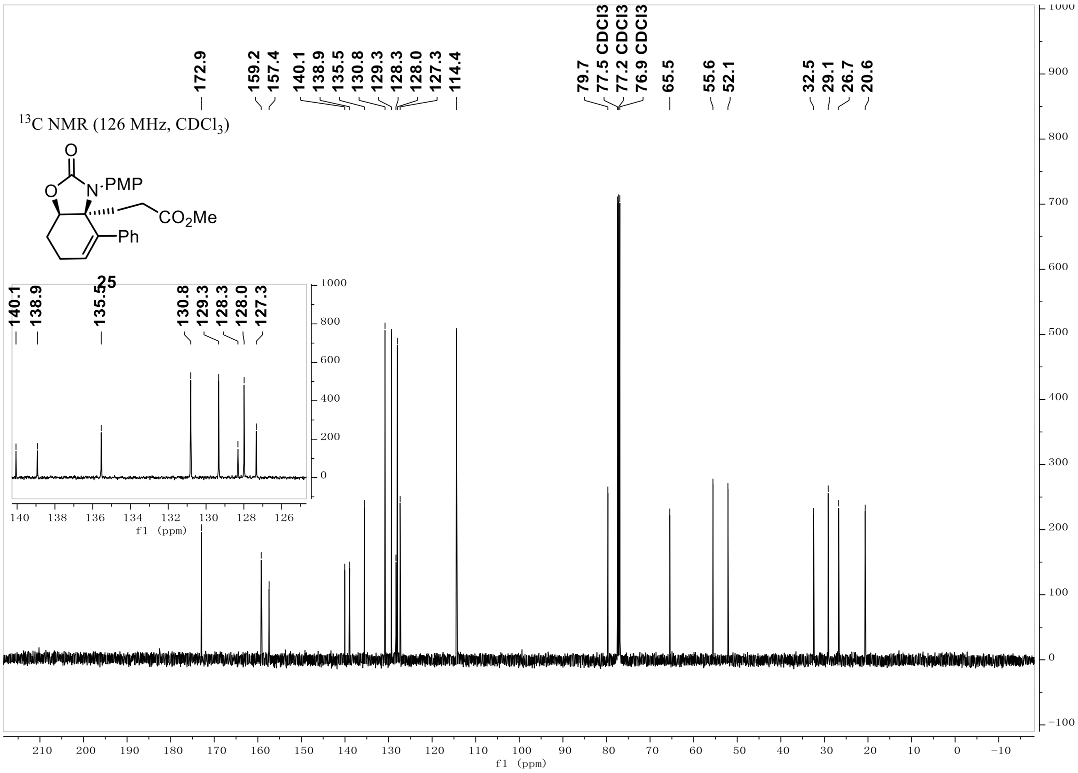

Supplementary Figure 25. ^{1}H NMR and ^{13}C NMR spectra of compound 25.

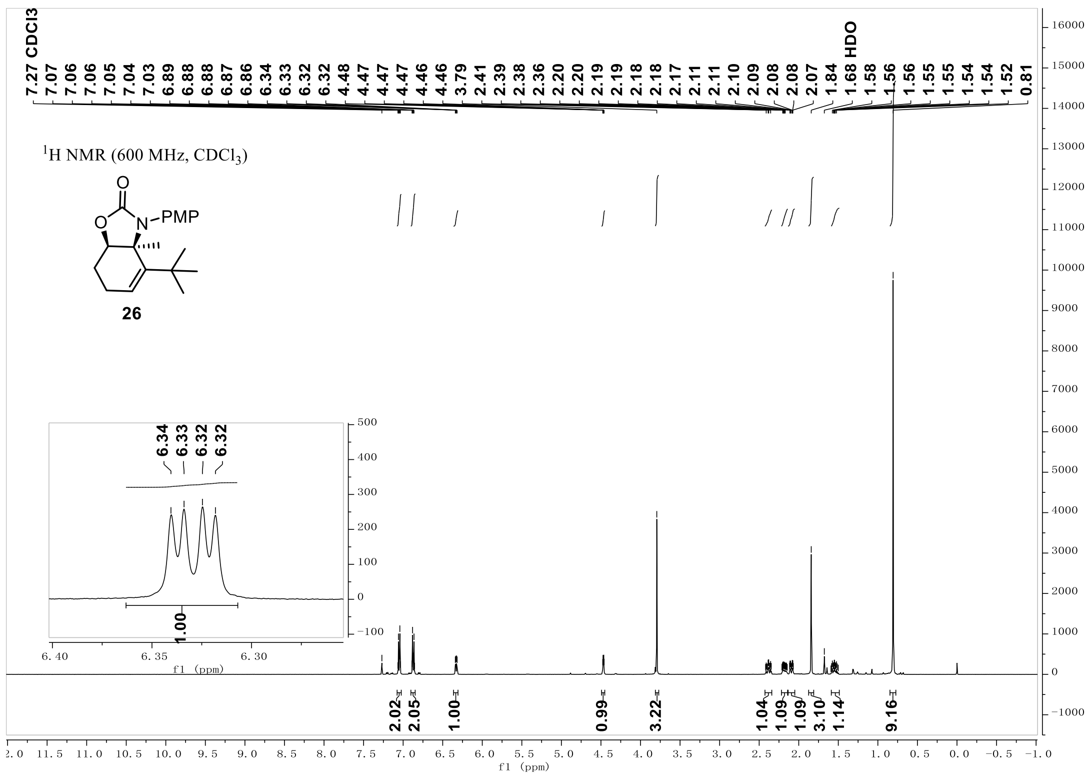

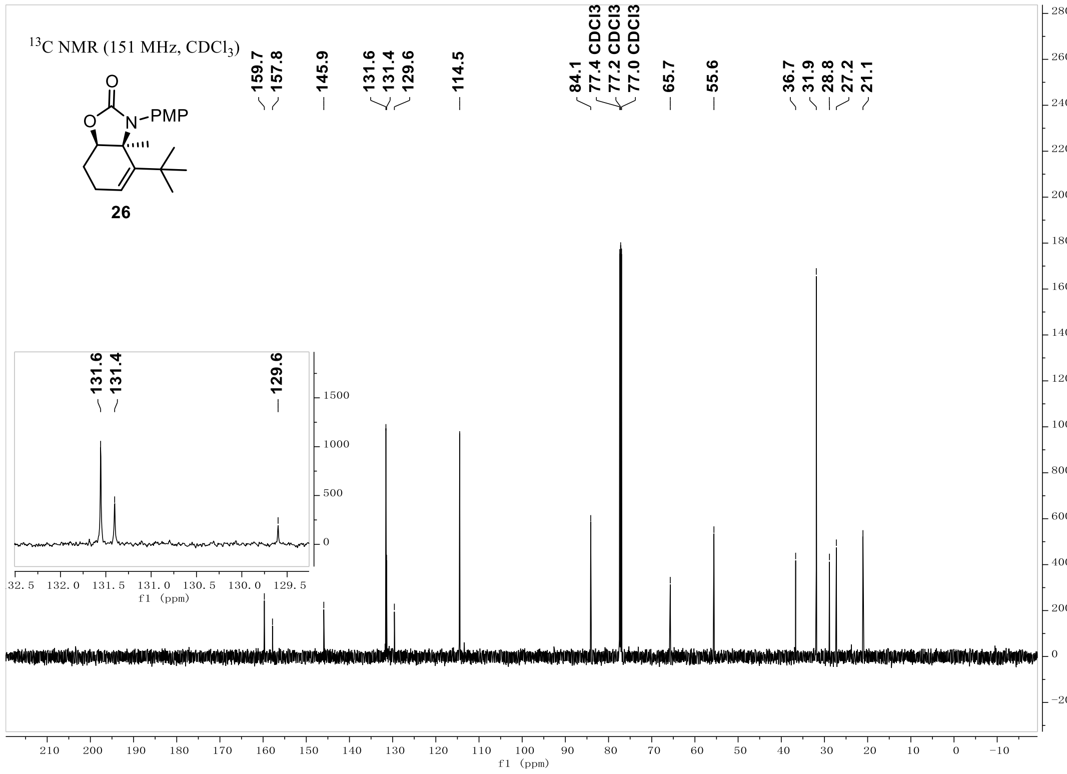

Supplementary Figure 26. ^{1}H NMR and ^{13}C NMR spectra of compound 26.

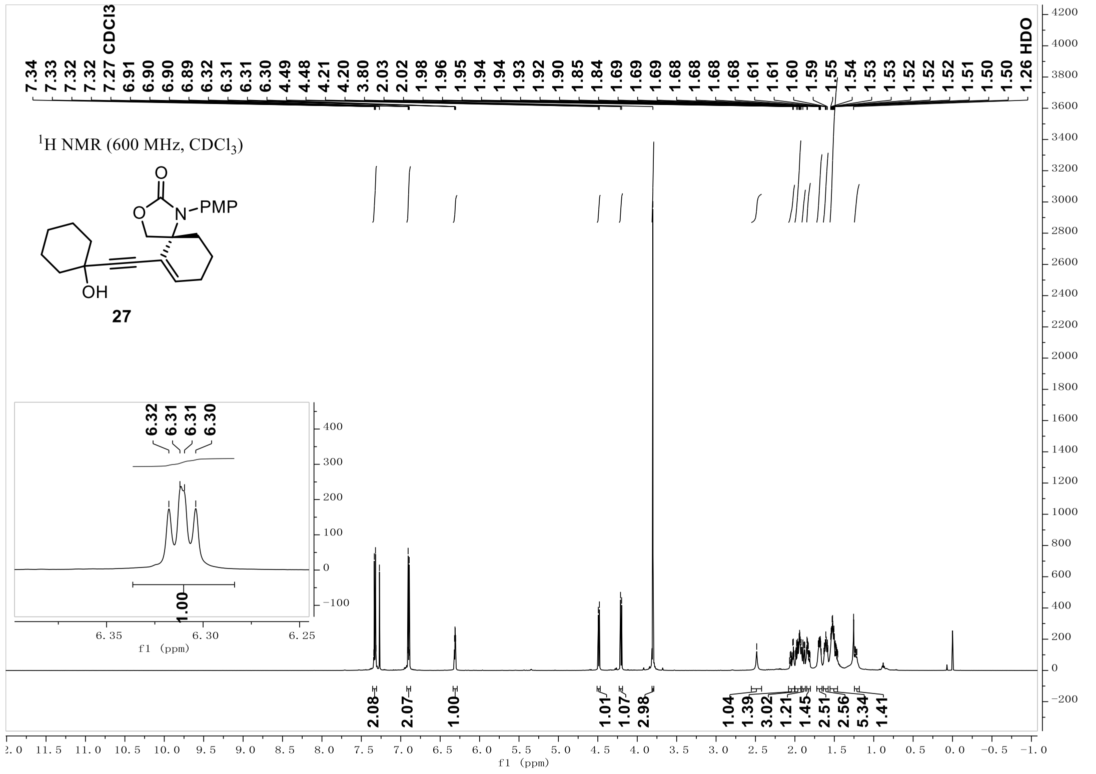

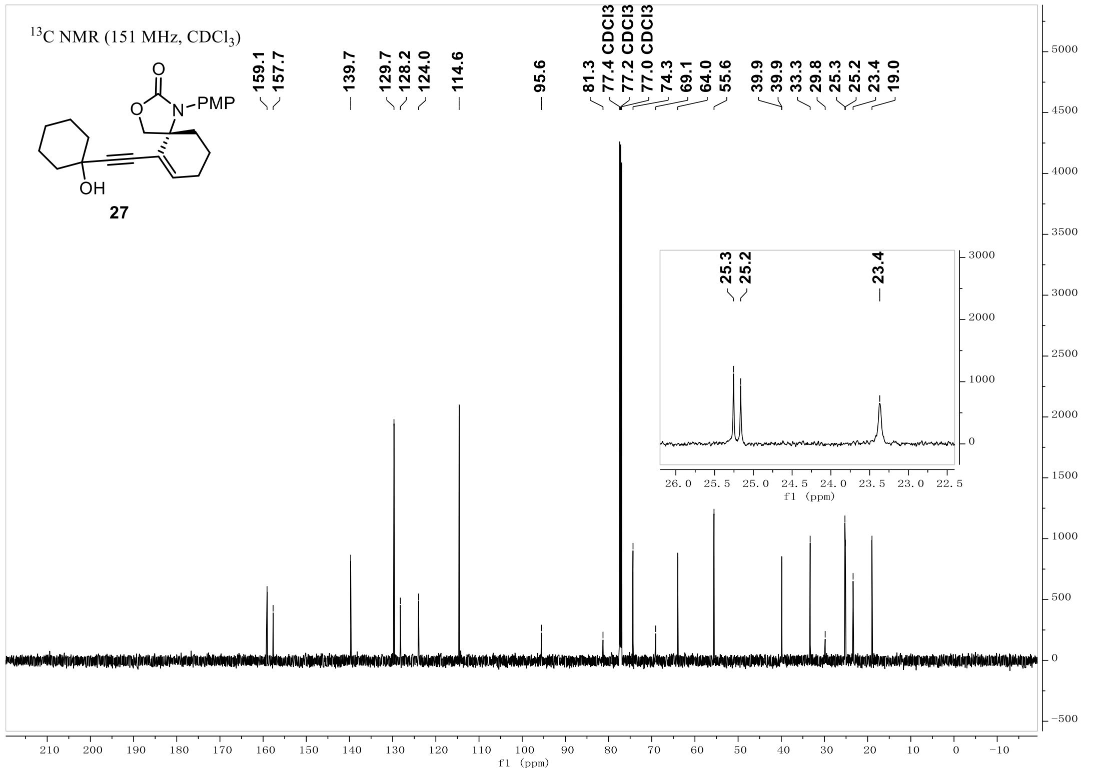

Supplementary Figure 27. ^{1}H NMR and ^{13}C NMR spectra of compound 27.

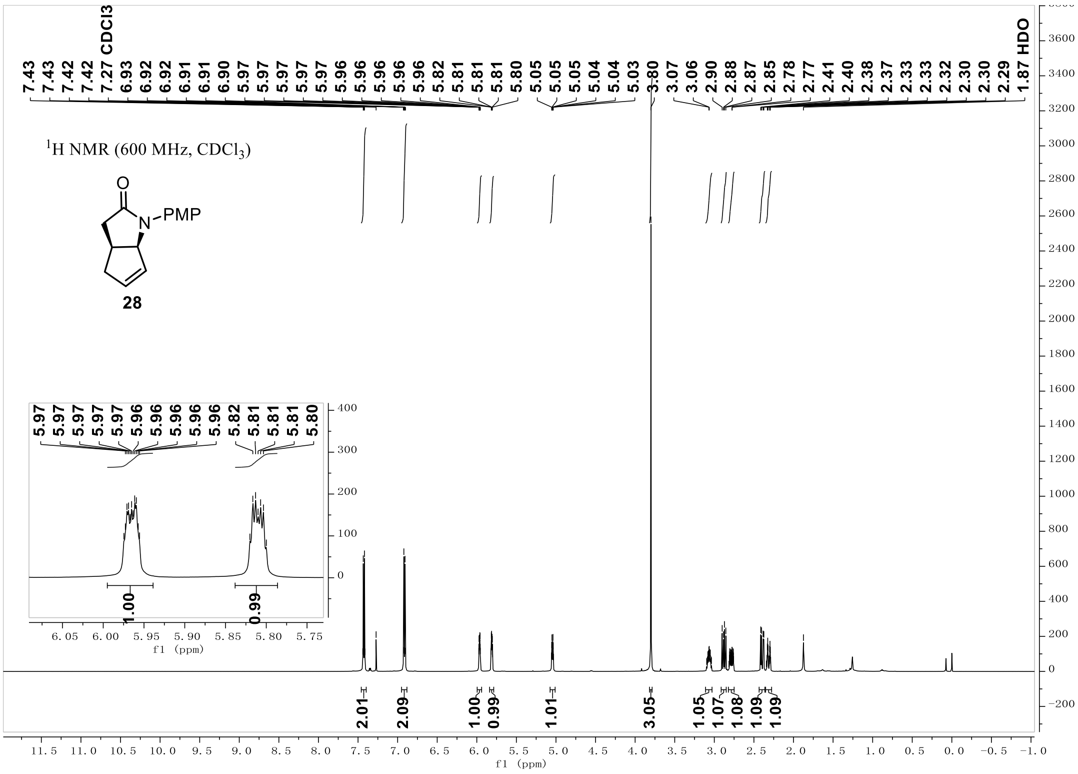

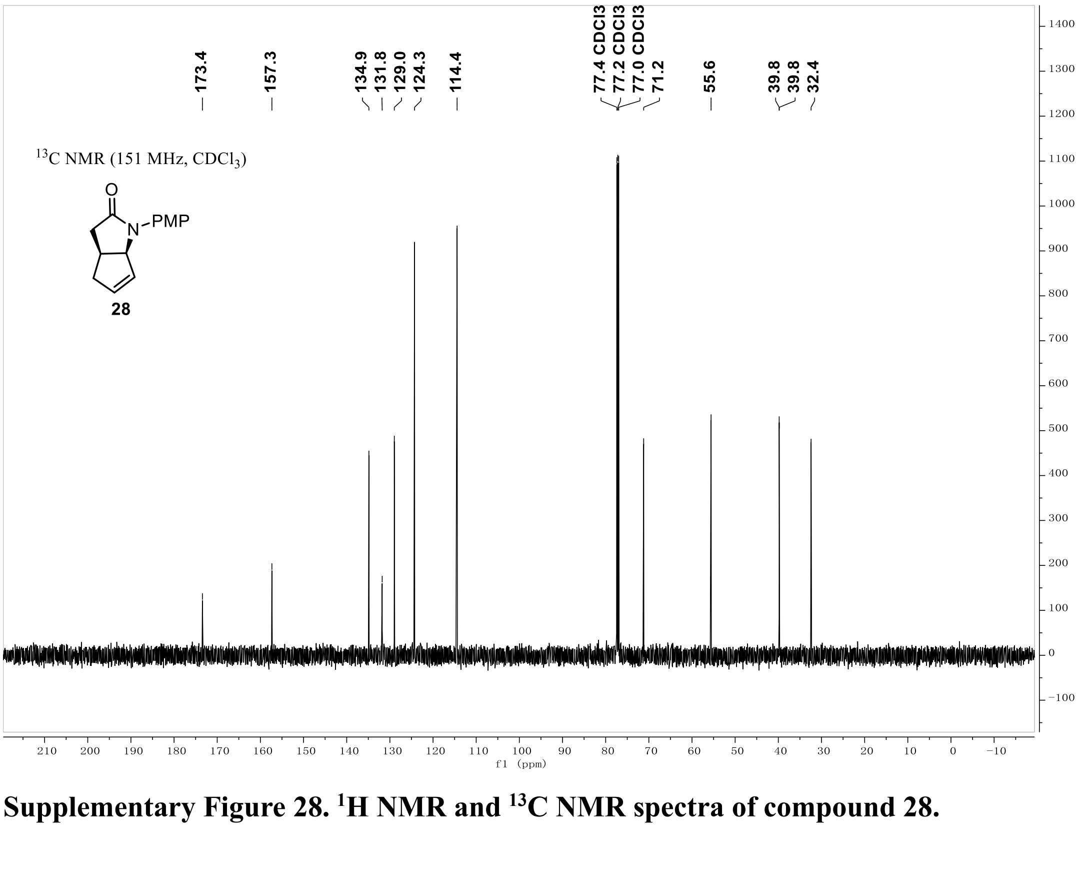

Supplementary Figure 28. ^{1}H NMR and ^{13}C NMR spectra of compound 28.

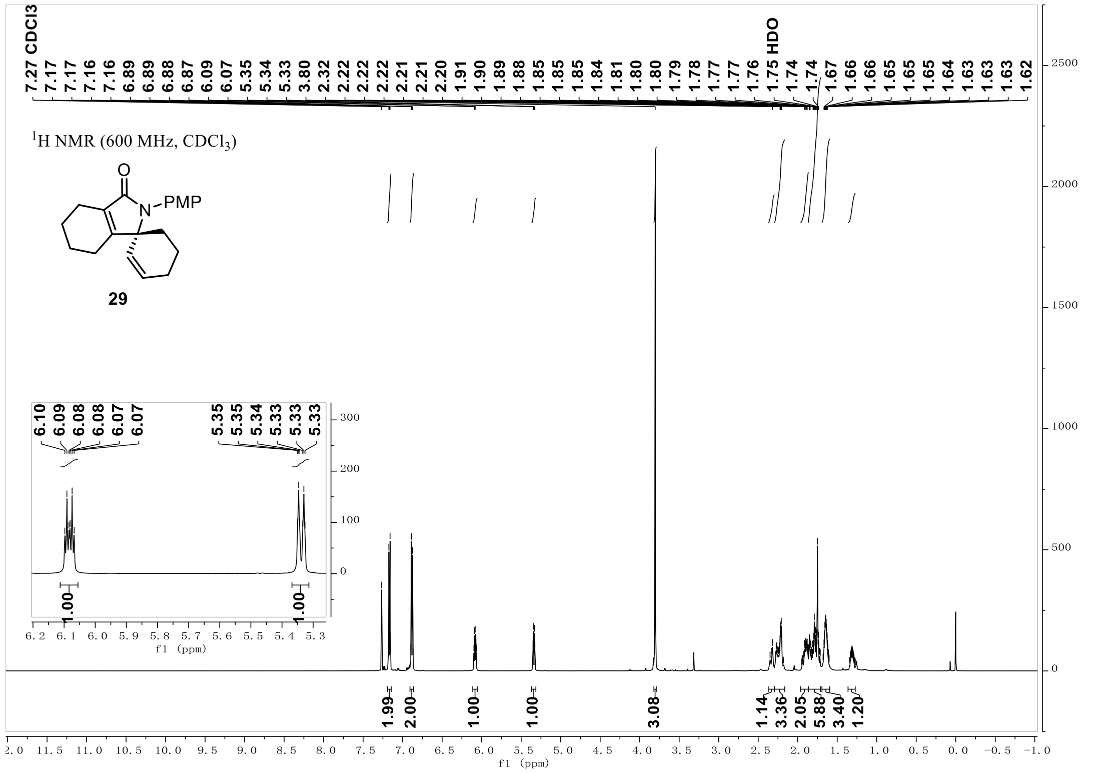

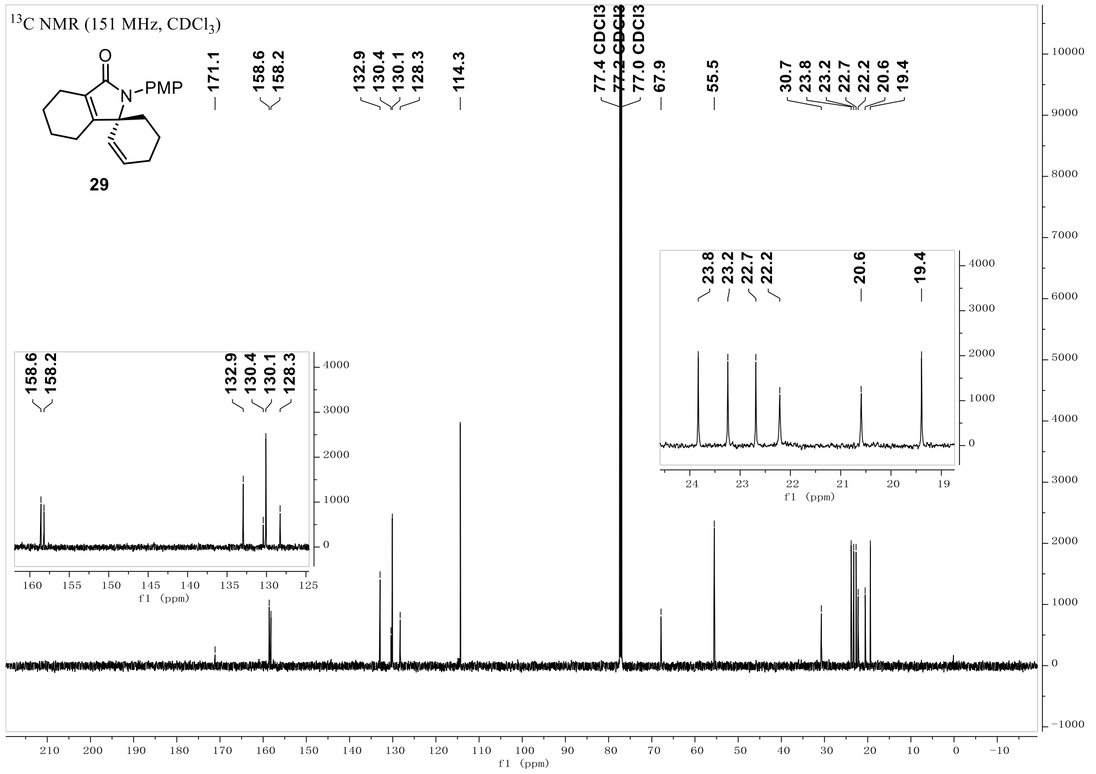

Supplementary Figure 29. ^{1}H NMR and ^{13}C NMR spectra of compound 29.

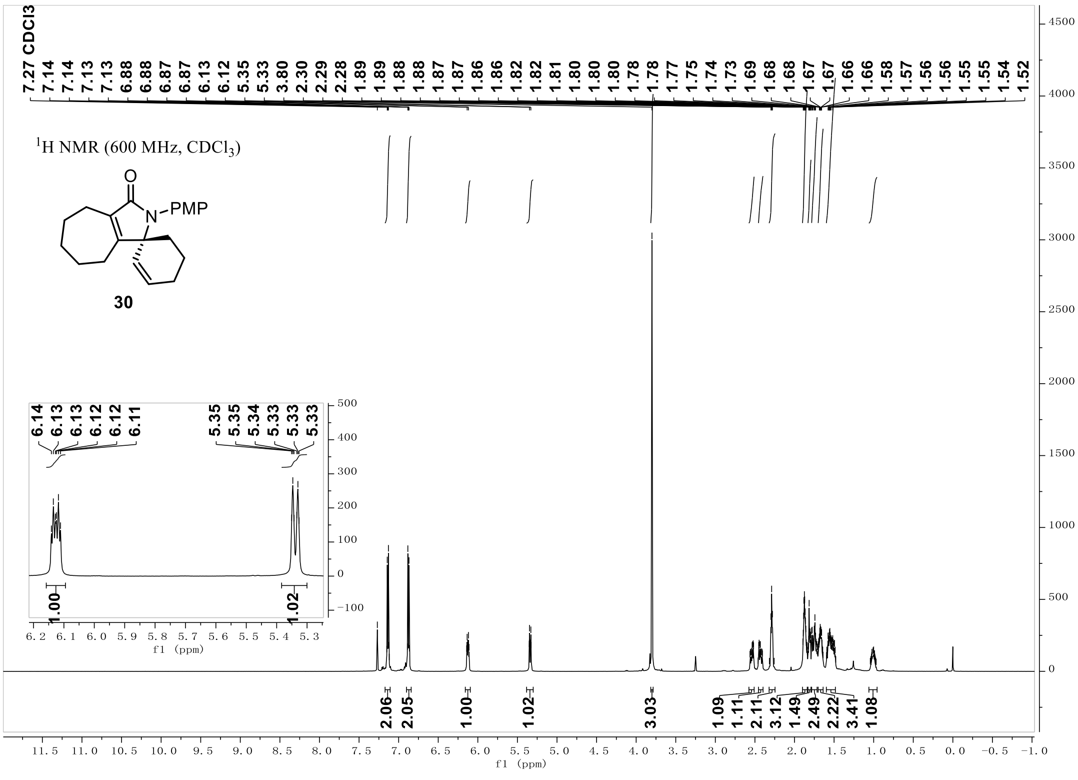

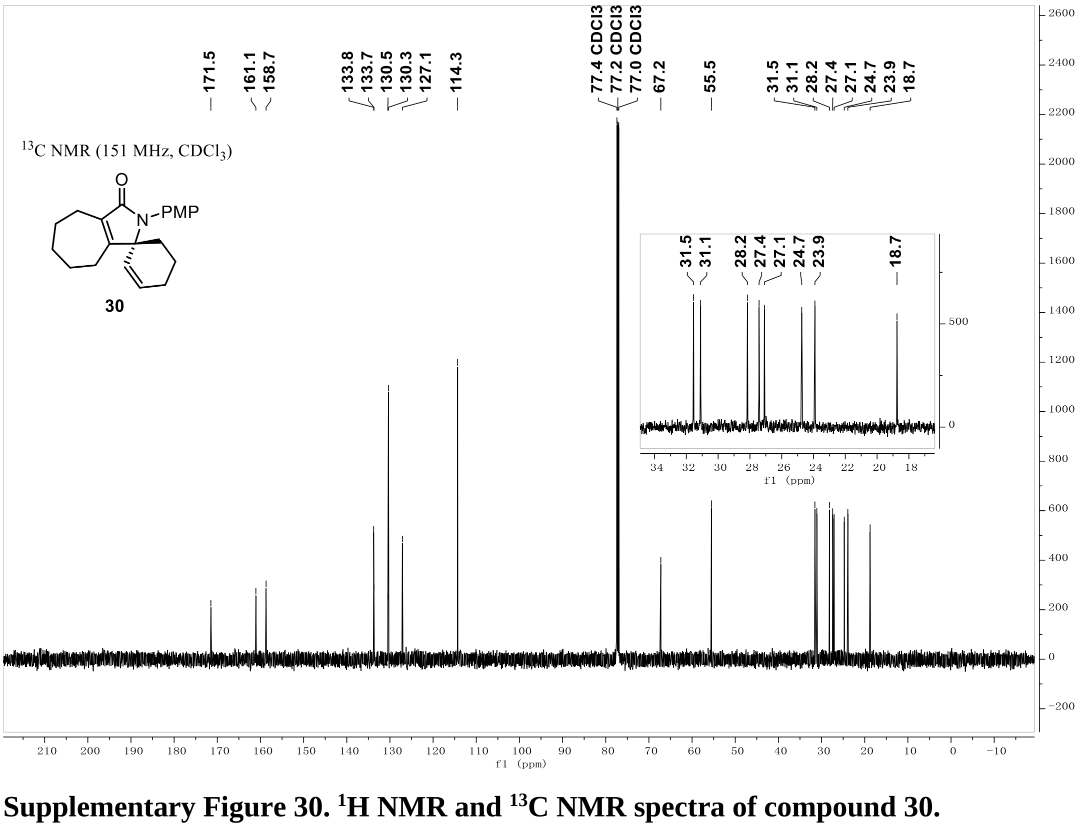

Supplementary Figure 30. ^{1}H NMR and ^{13}C NMR spectra of compound 30.

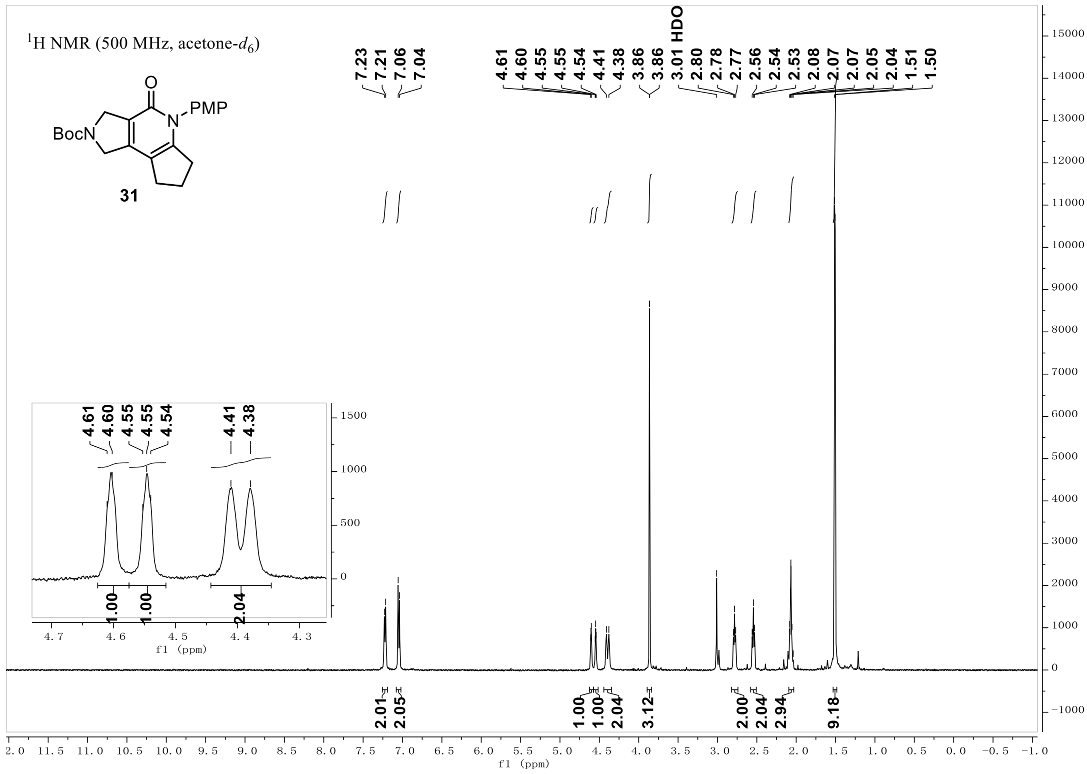

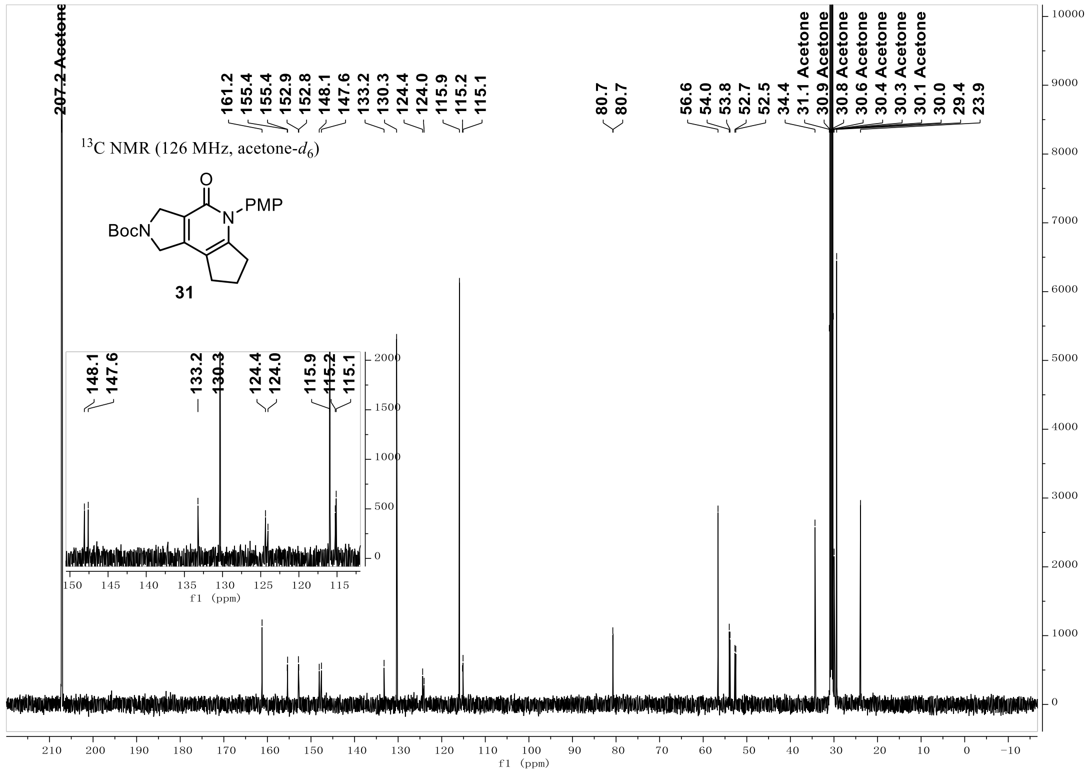

Supplementary Figure 31. ^{1}H NMR and ^{13}C NMR spectra of compound 31.

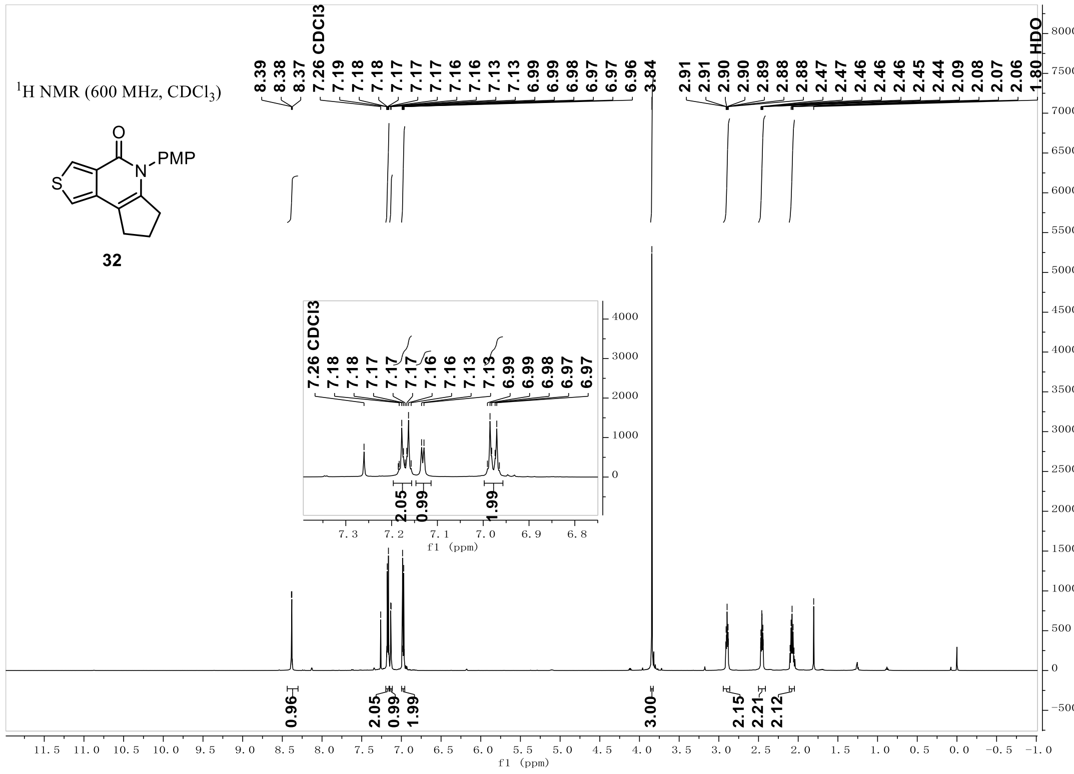

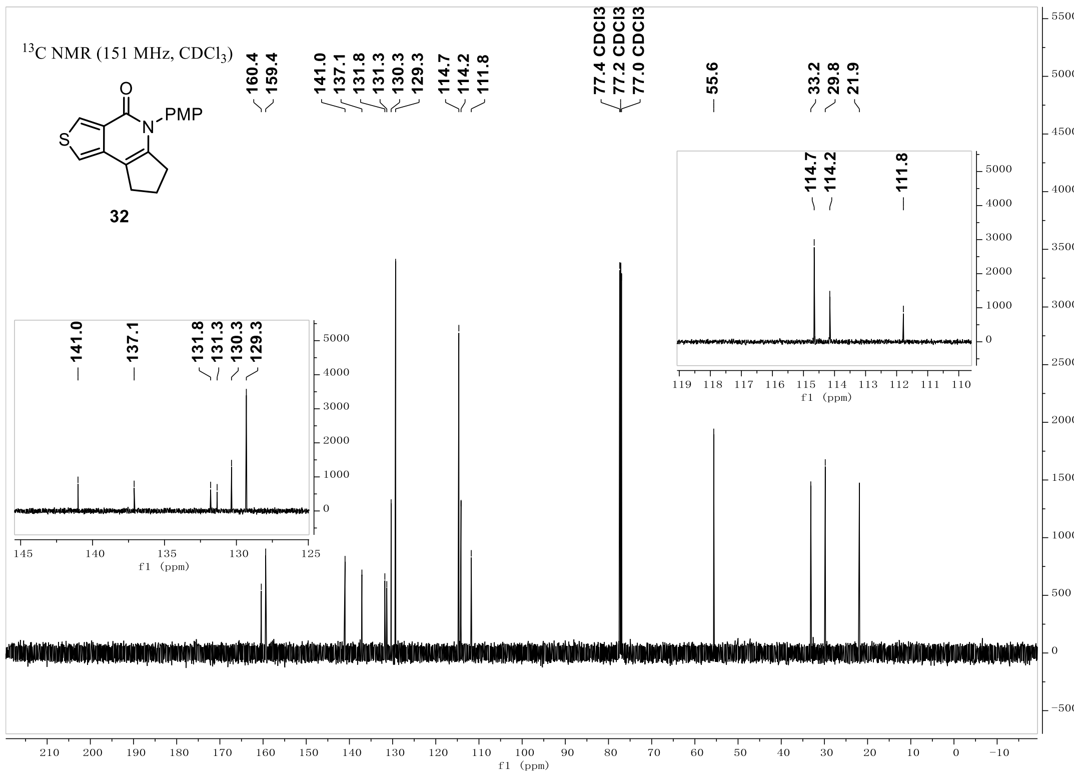

Supplementary Figure 32. ^{1}H NMR and ^{13}C NMR spectra of compound 32.

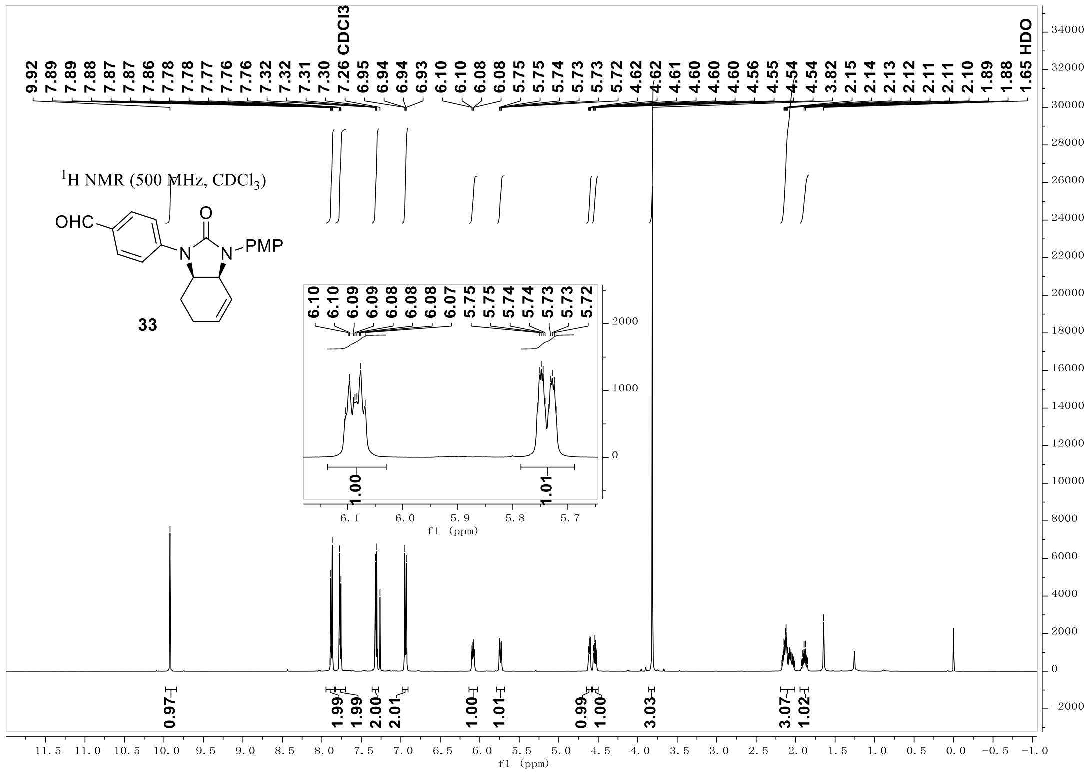

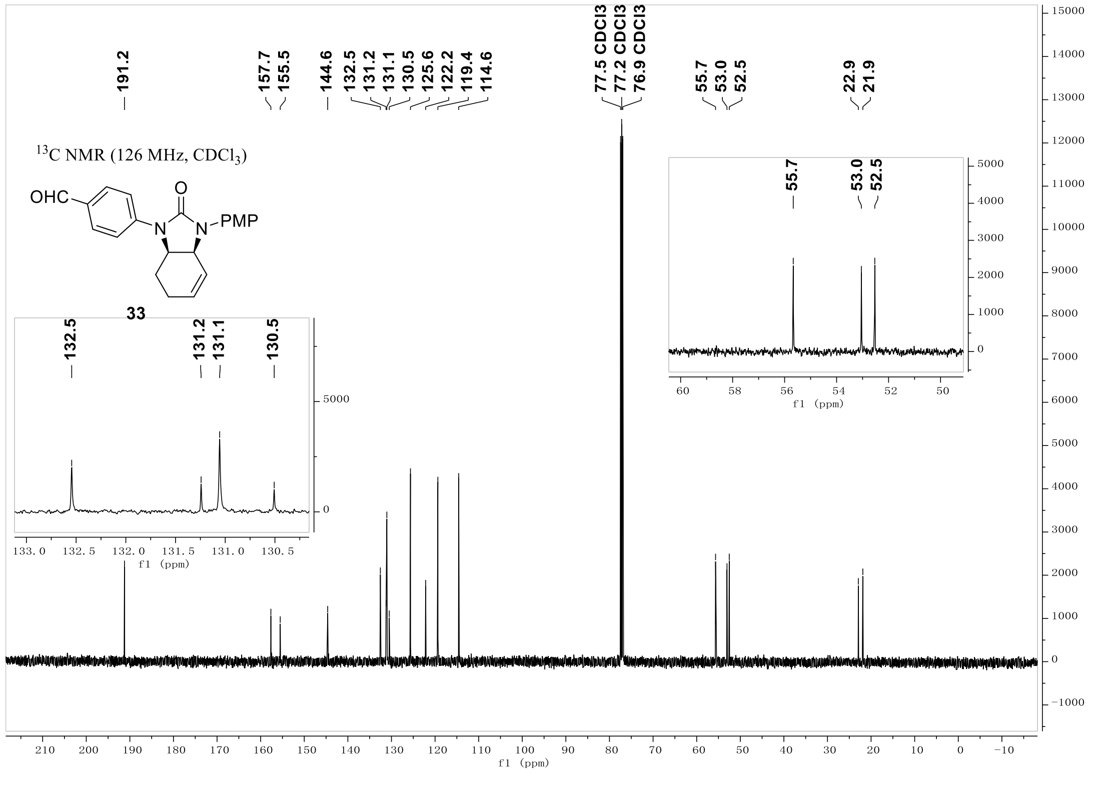

Supplementary Figure 33. ^{1}H NMR and ^{13}C NMR spectra of compound 33.

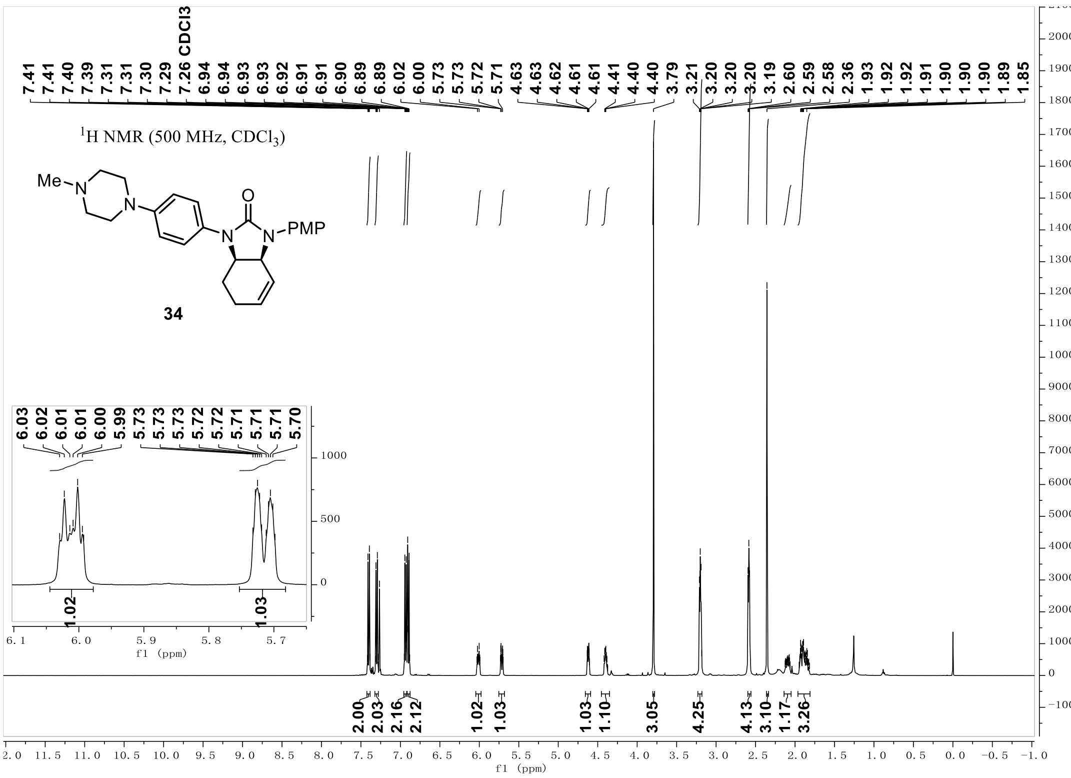

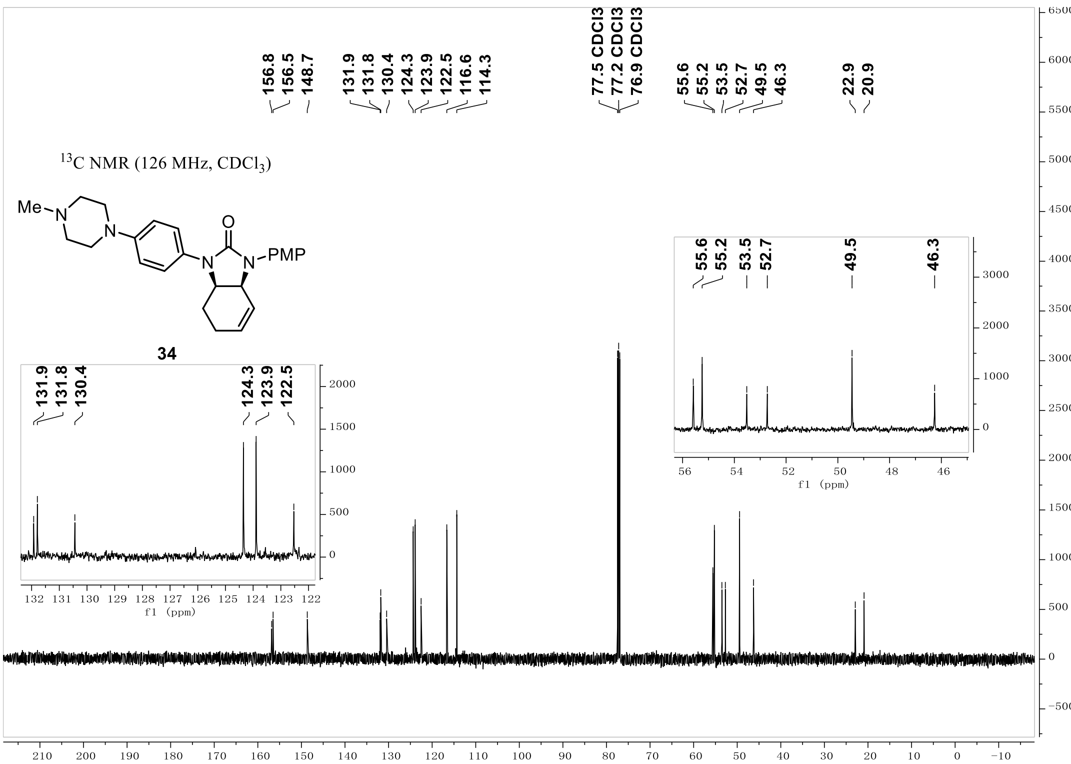

Supplementary Figure 34. ^{1}H NMR and ^{13}C NMR spectra of compound 34.

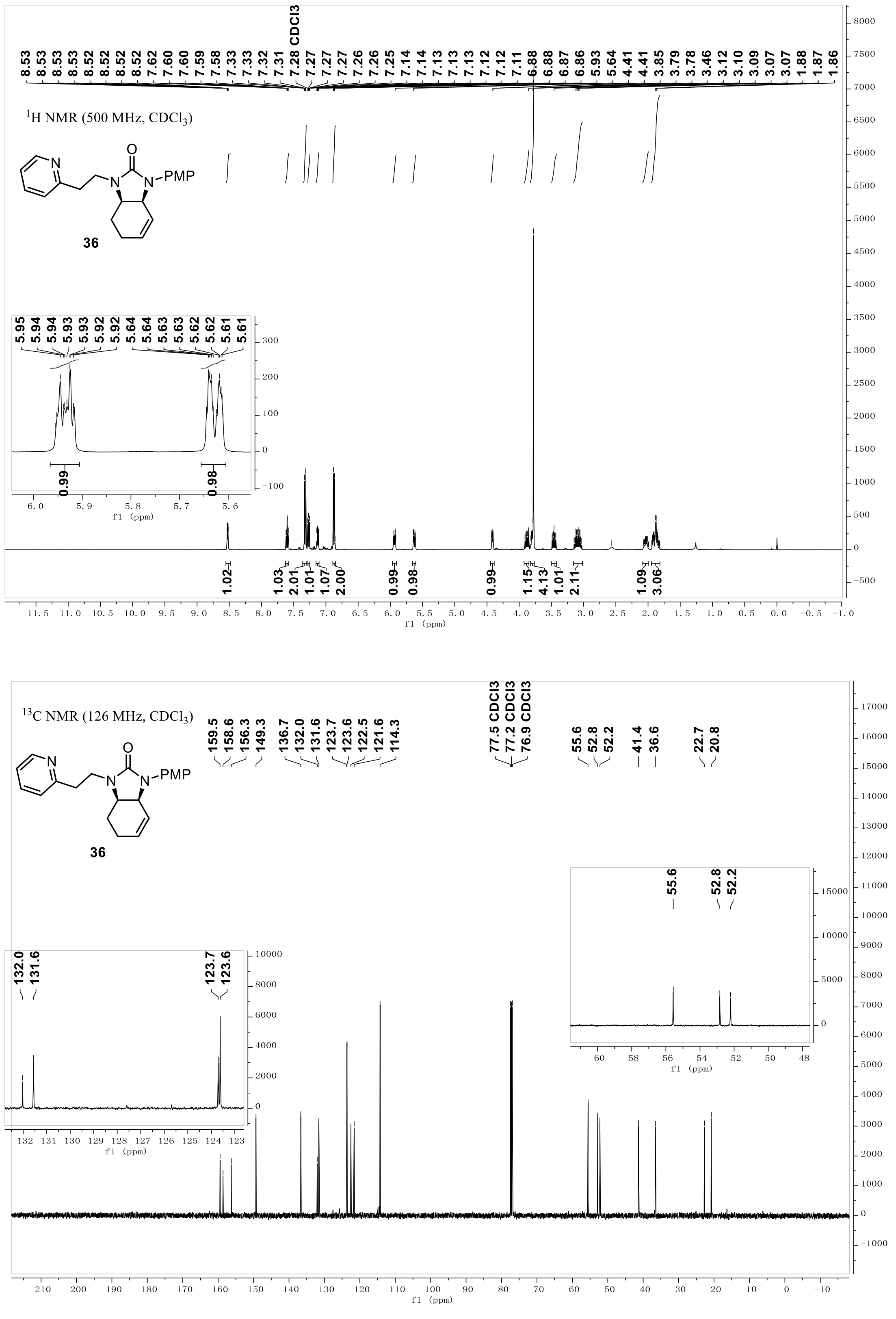

Supplementary Figure 35. ^{1}**H NMR and** ^{13}**C NMR spectra of compound 36.**

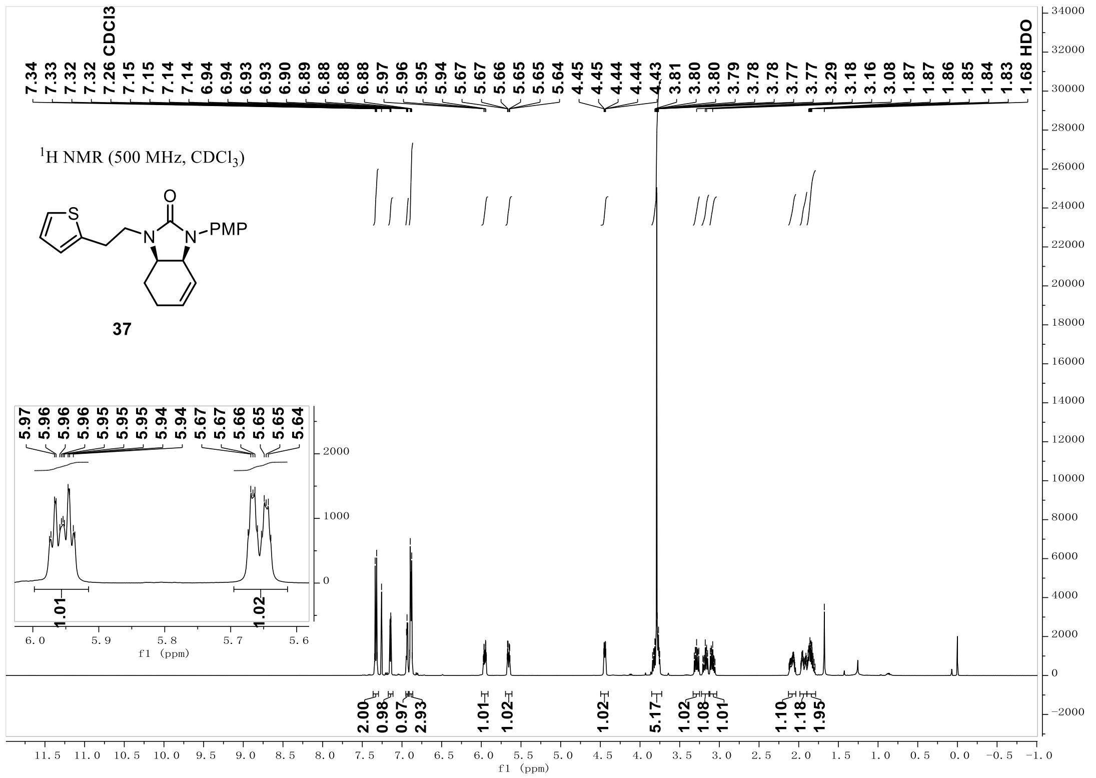

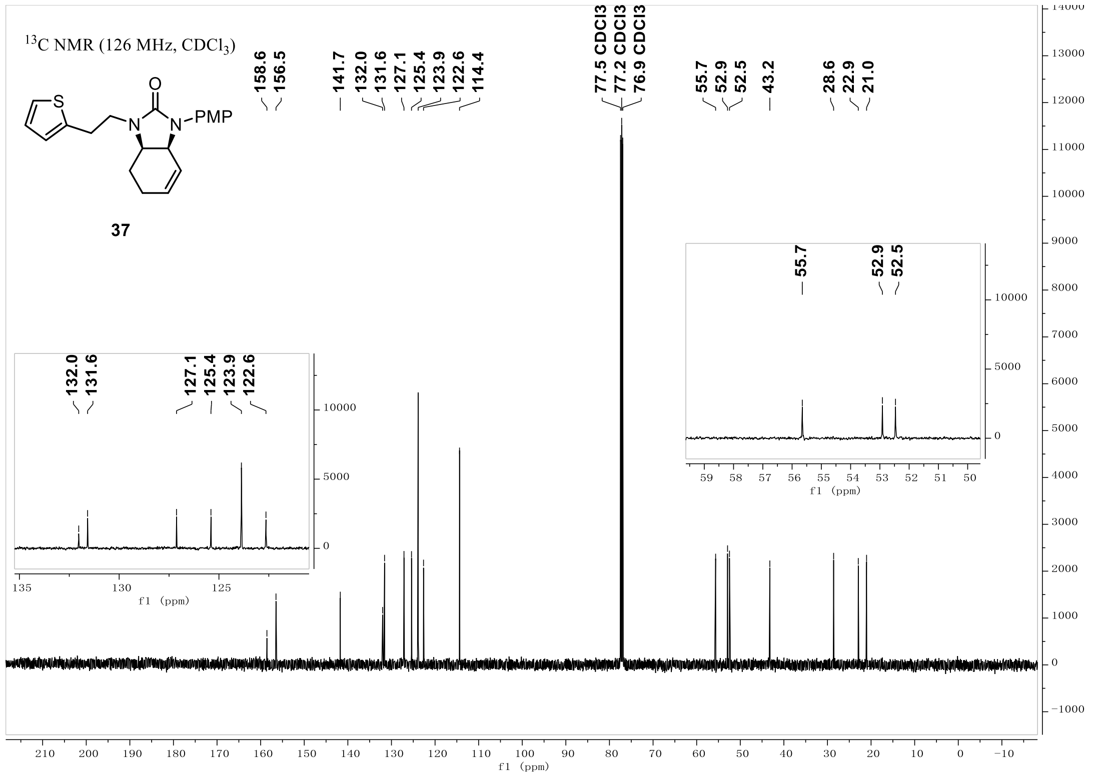

Supplementary Figure 36. ^{1}H NMR and ^{13}C NMR spectra of compound 37.

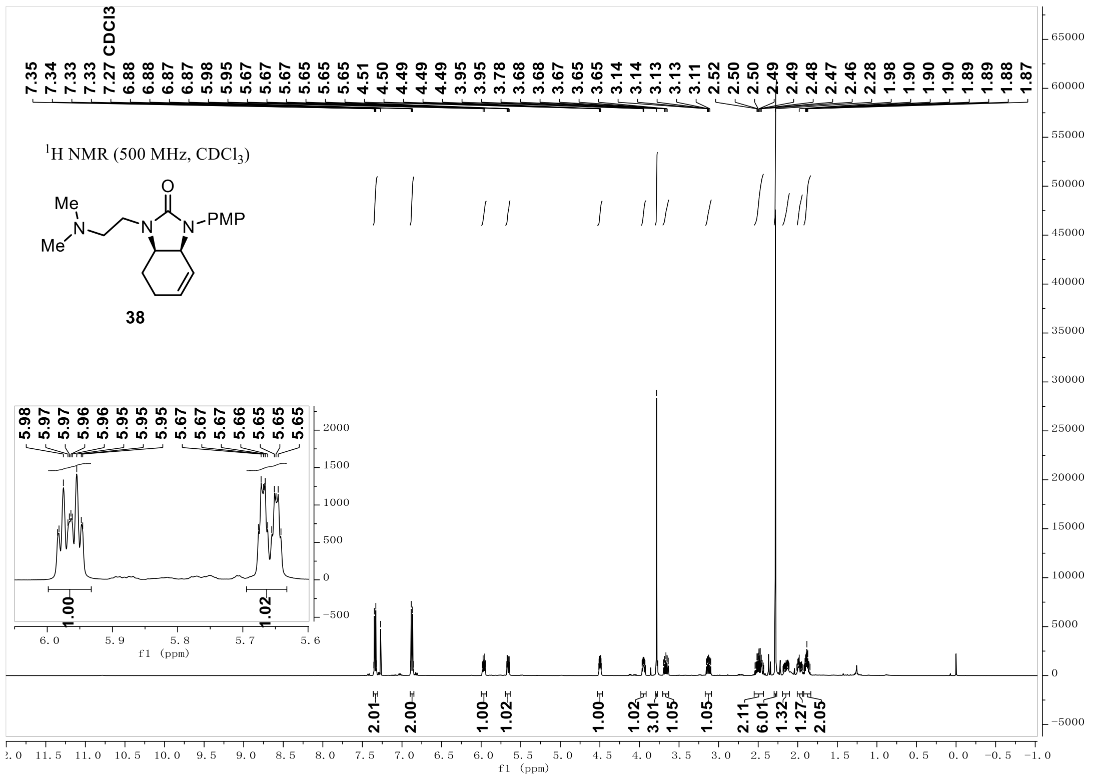

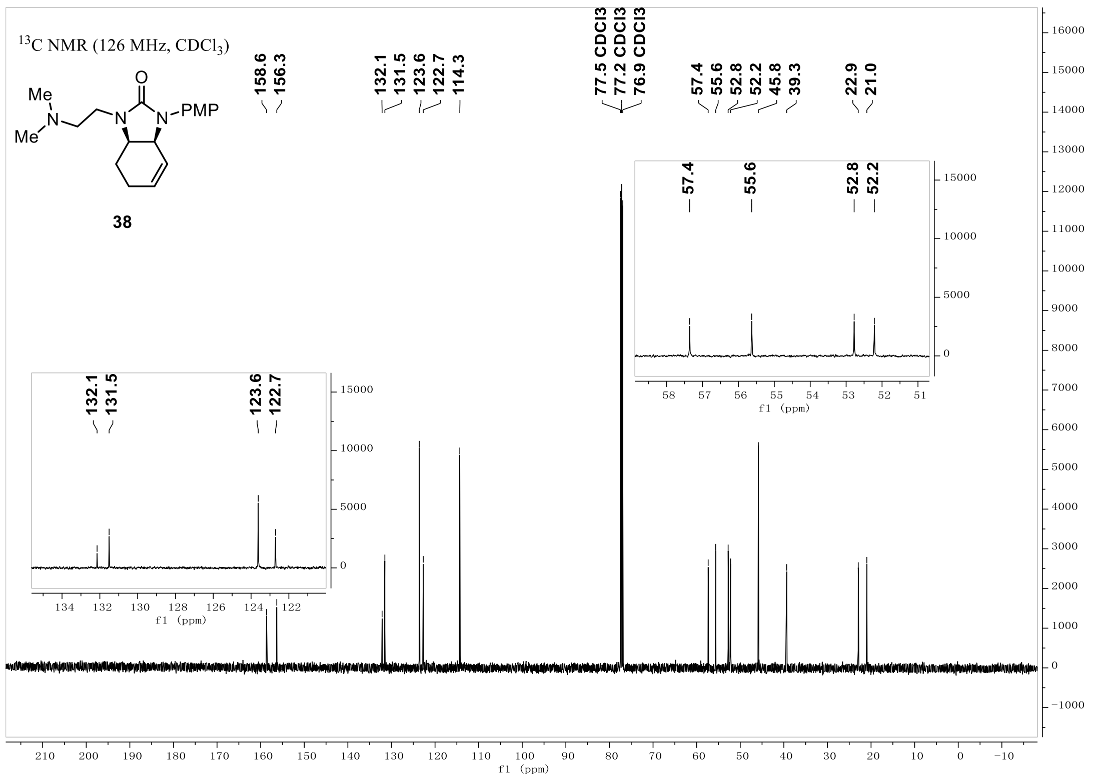

Supplementary Figure 37. ^{1}H NMR and ^{13}C NMR spectra of compound 38.

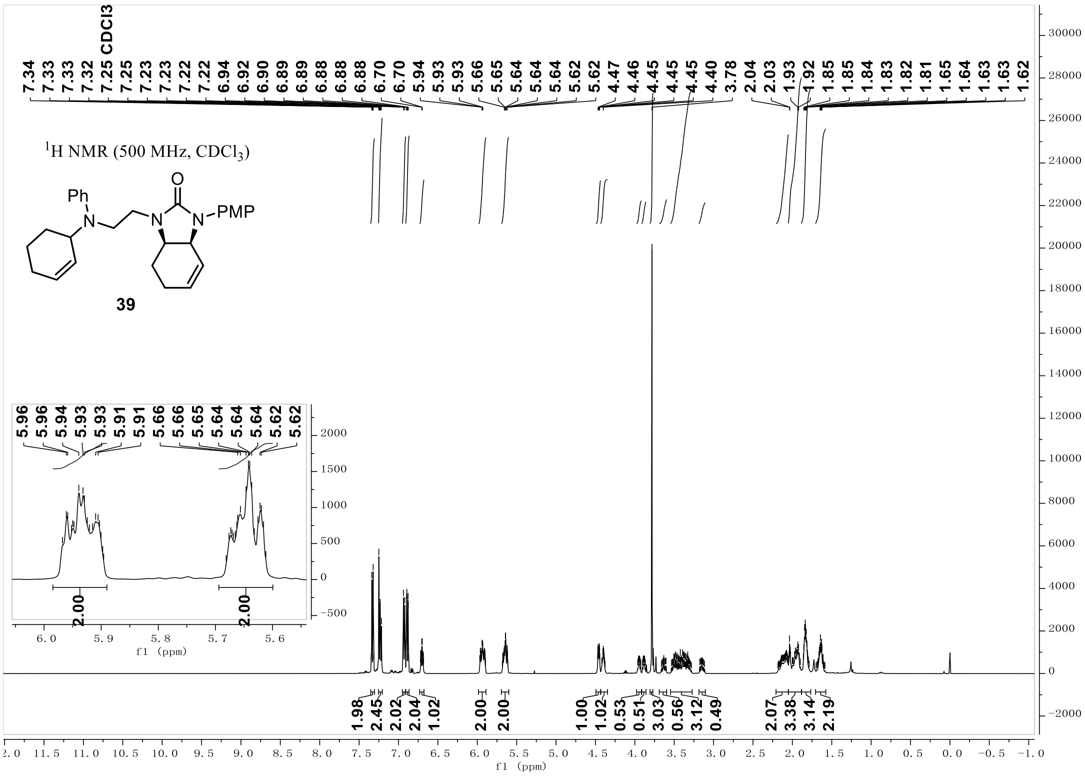

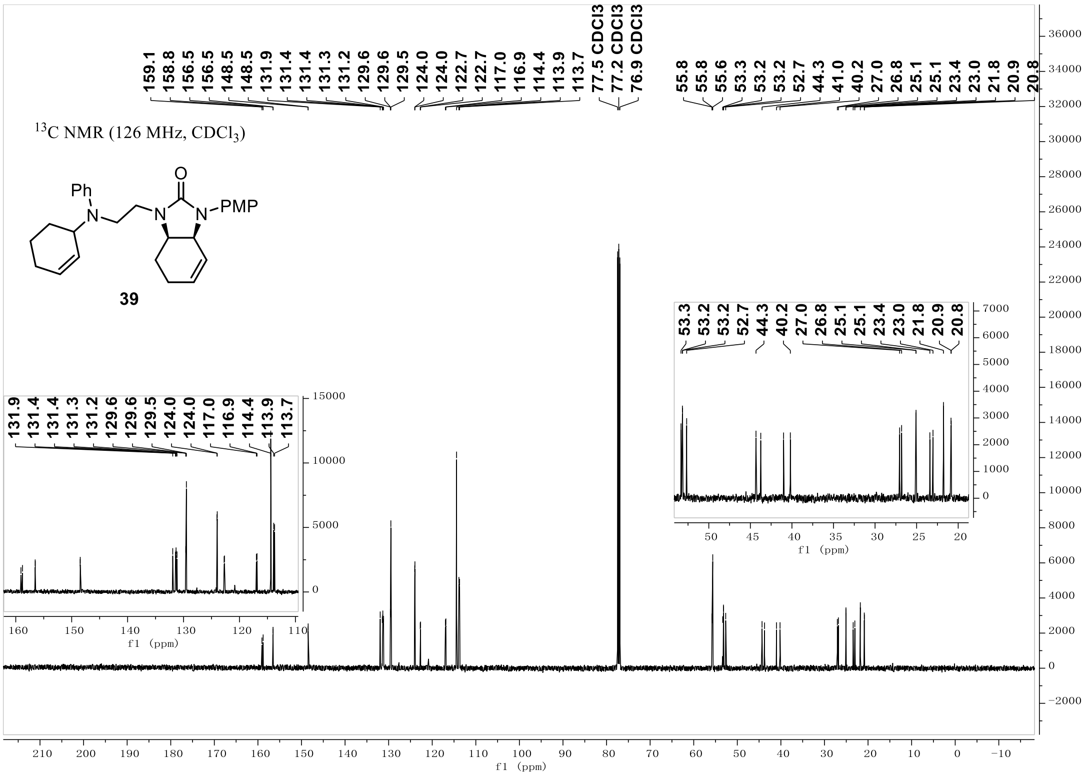

Supplementary Figure 38. ^{1}H NMR and ^{13}C NMR spectra of compound 39. The title compound was isolated as a 1:1 mixture of diastereomers due to the presence of a stereocenter.

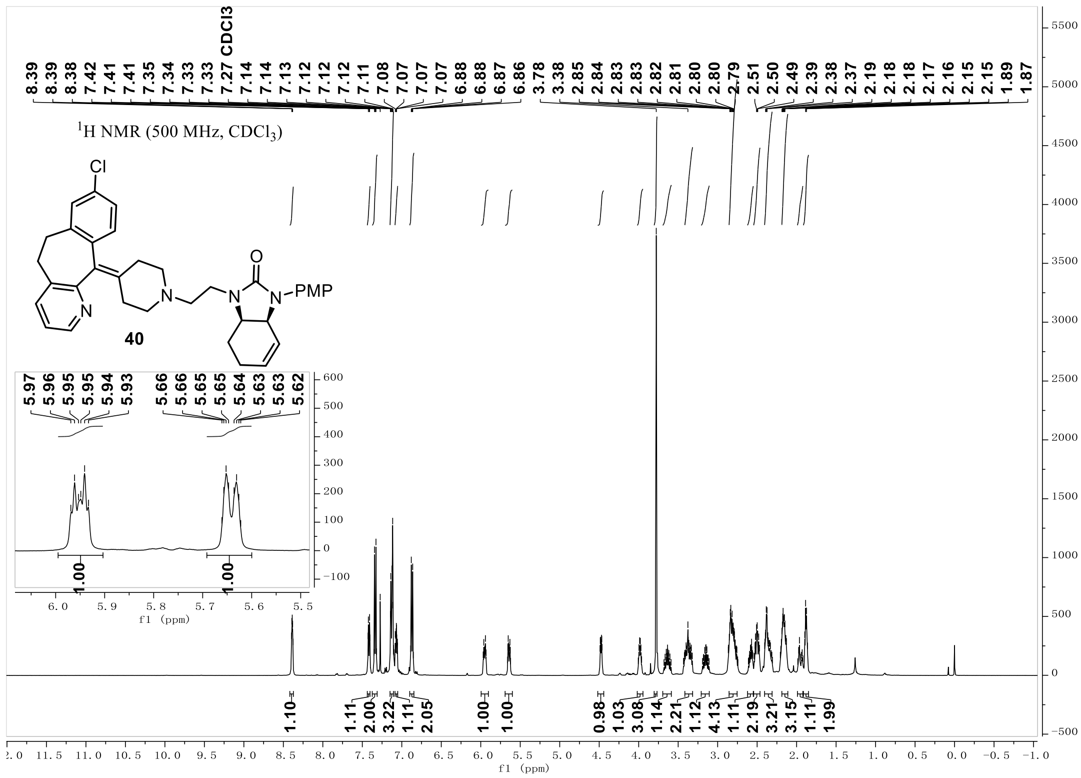

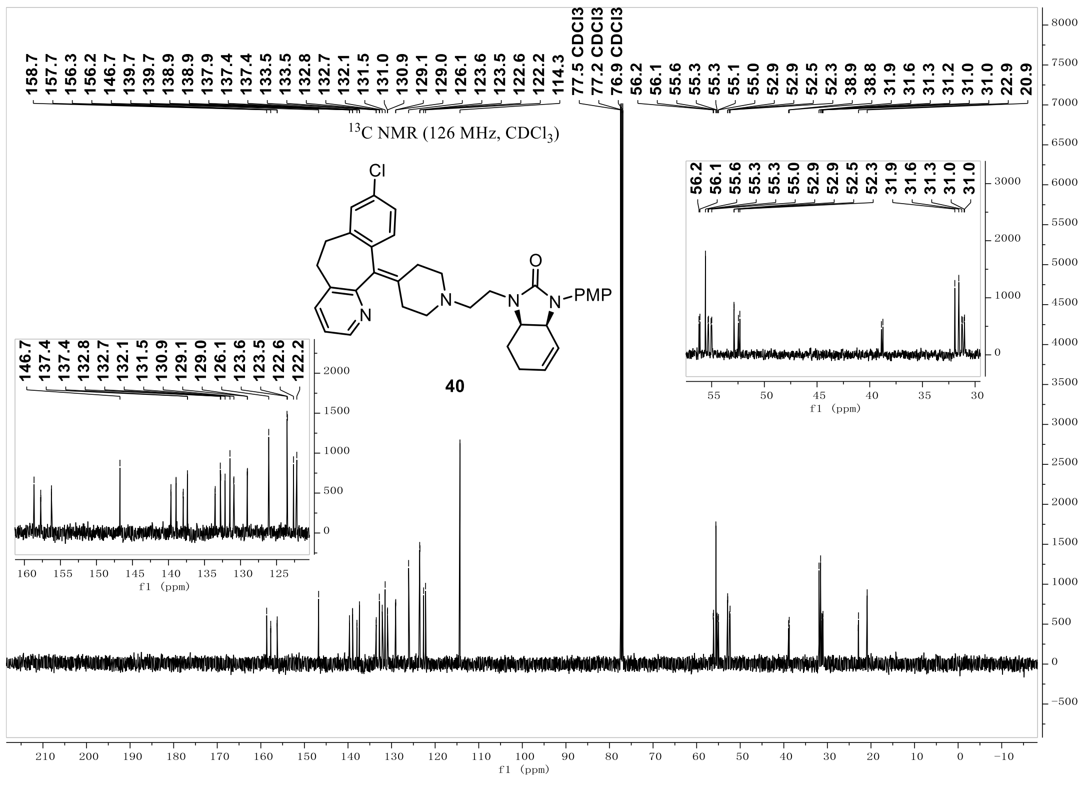

Supplementary Figure 39. ^{1}H NMR and ^{13}C NMR spectra of compound 40. The title compound was isolated as a 1:1 mixture of diastereomers due to the axial chiral tetrasubstituted alkene.

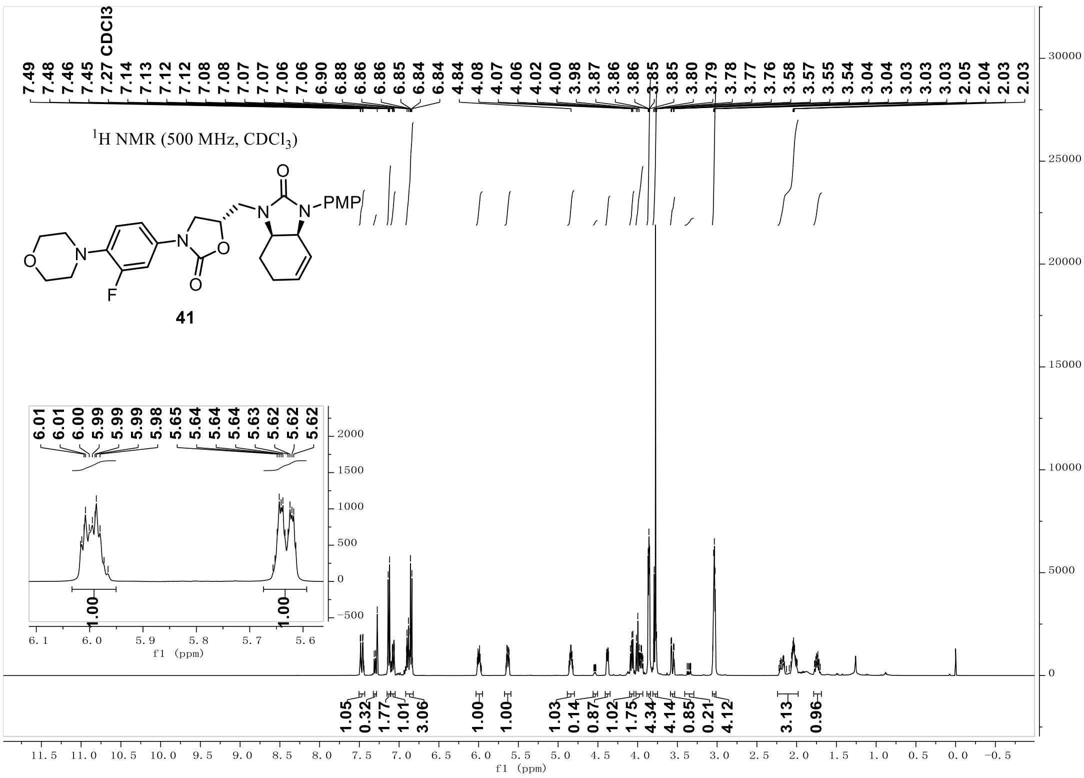

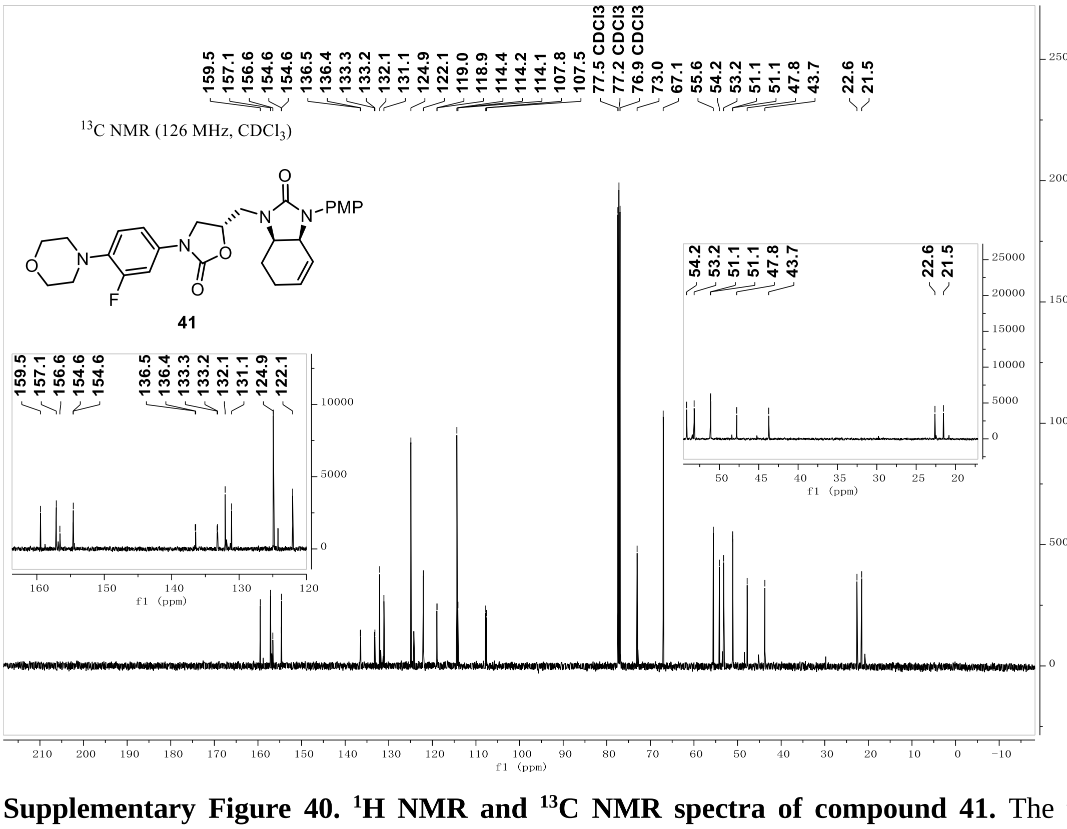

Supplementary Figure 40. ^{1}H NMR and ^{13}C NMR spectra of compound 41. The title compound was isolated as an isolated as a 5.6:1 mixture of diastereomers due to the sterocenter at the cyclic carbamate.

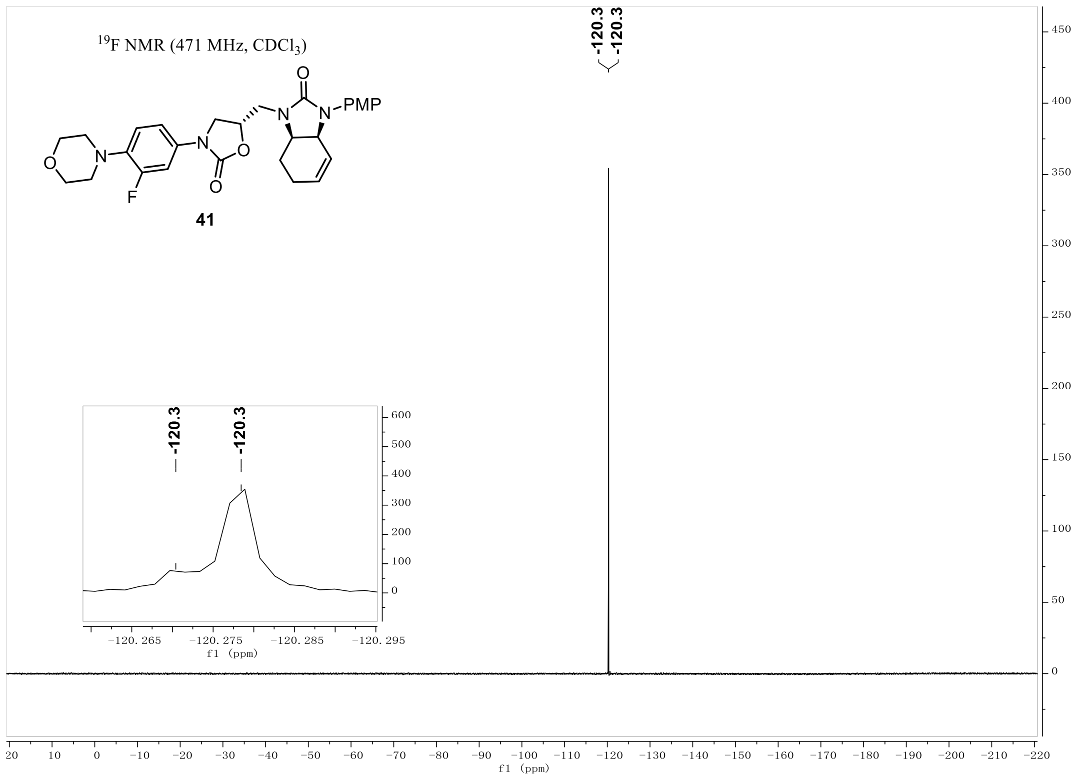

Supplementary Figure 41. ^{19}F NMR spectra of compound 41. The title compound was isolated as an isolated as a 5.6:1 mixture of diastereomers due to the sterocenter at the cyclic carbamate.

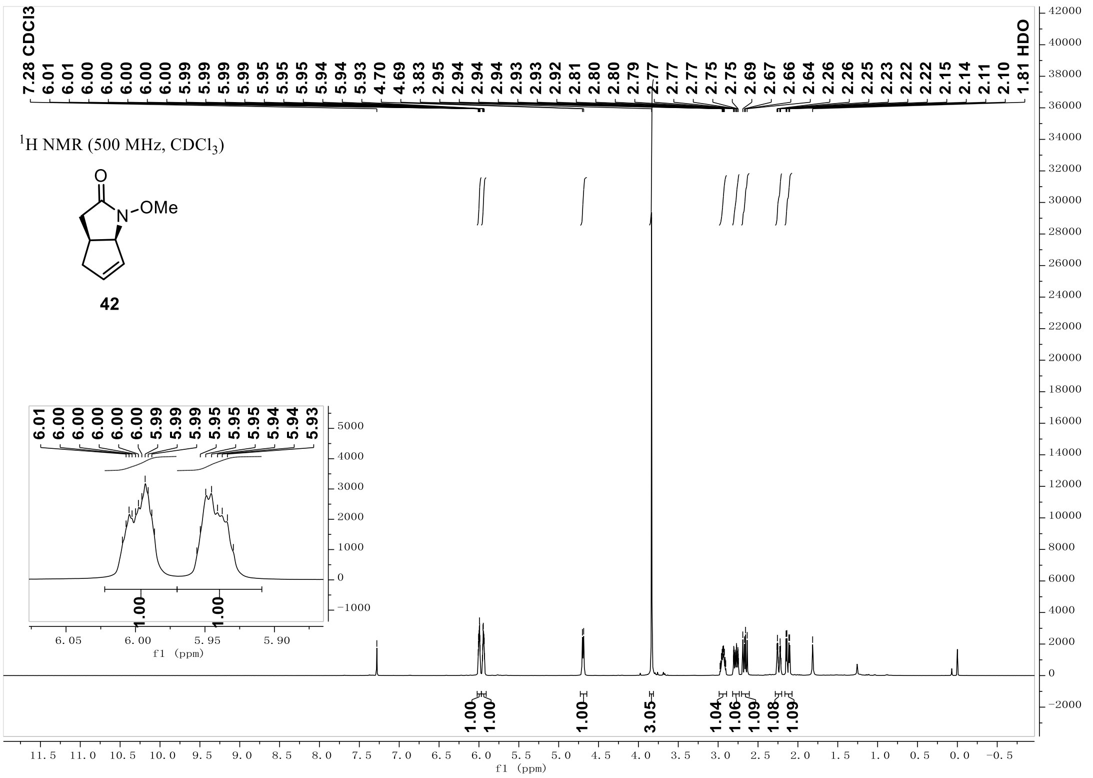

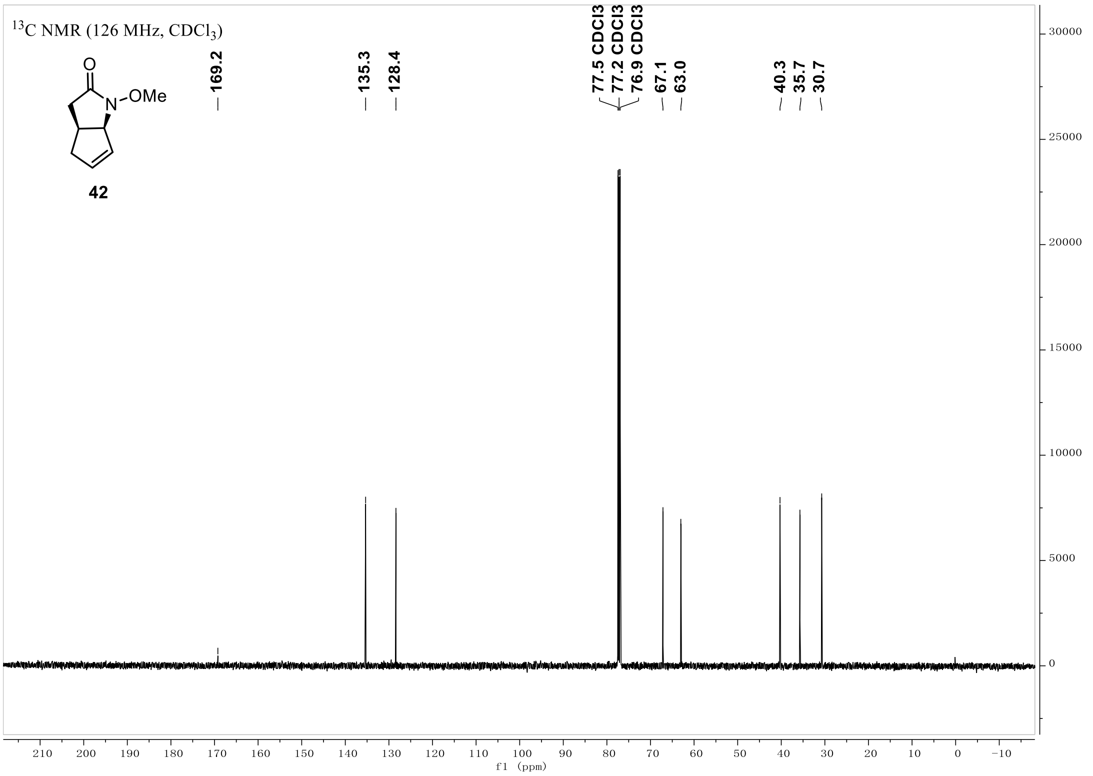

Supplementary Figure 42. ^{1}H NMR and ^{13}C NMR spectra of compound 42.

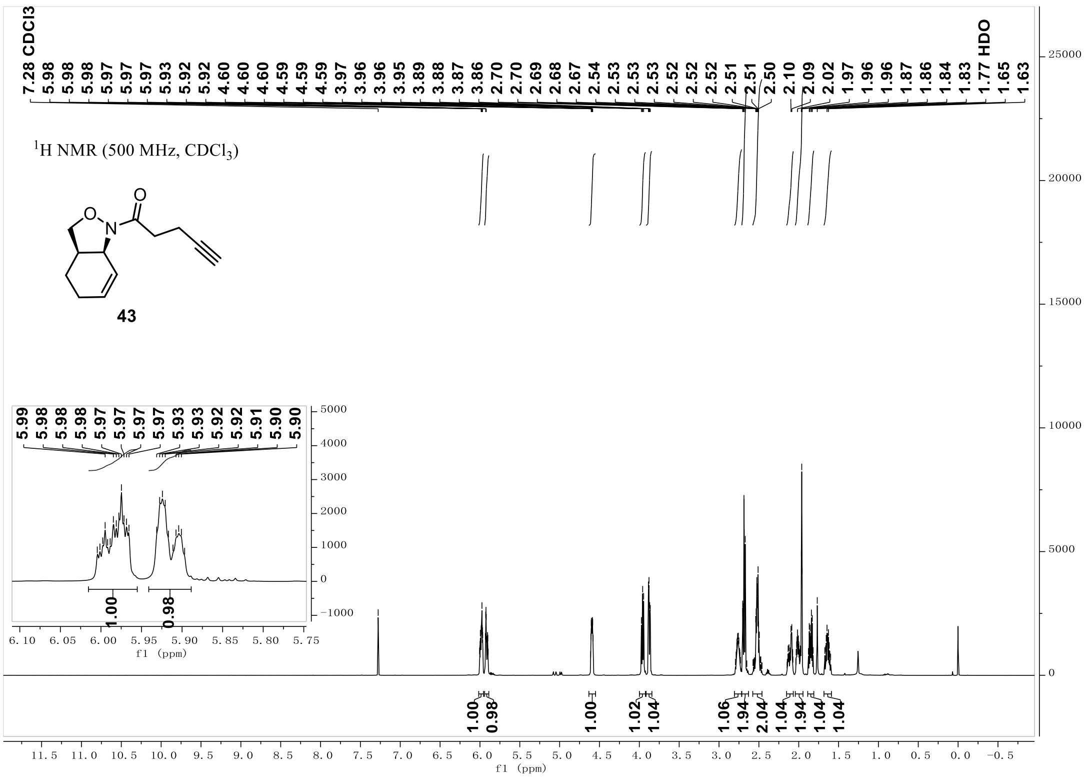

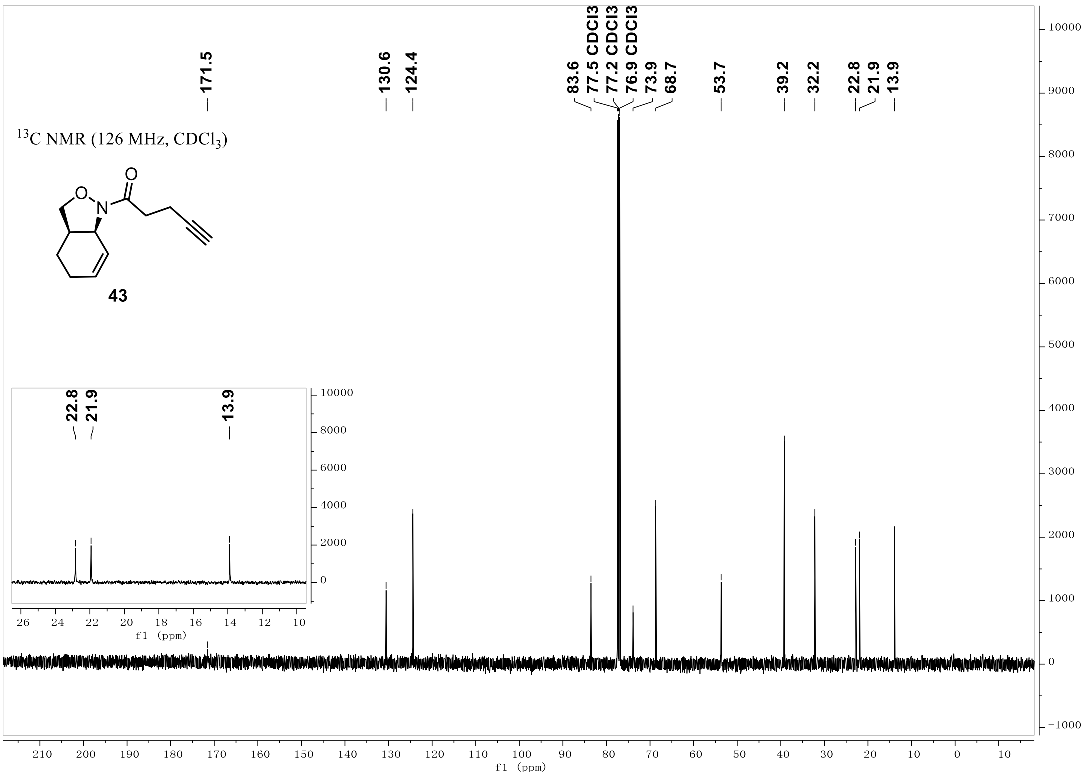

Supplementary Figure 43. ^{1}H NMR and ^{13}C NMR spectra of compound 43.

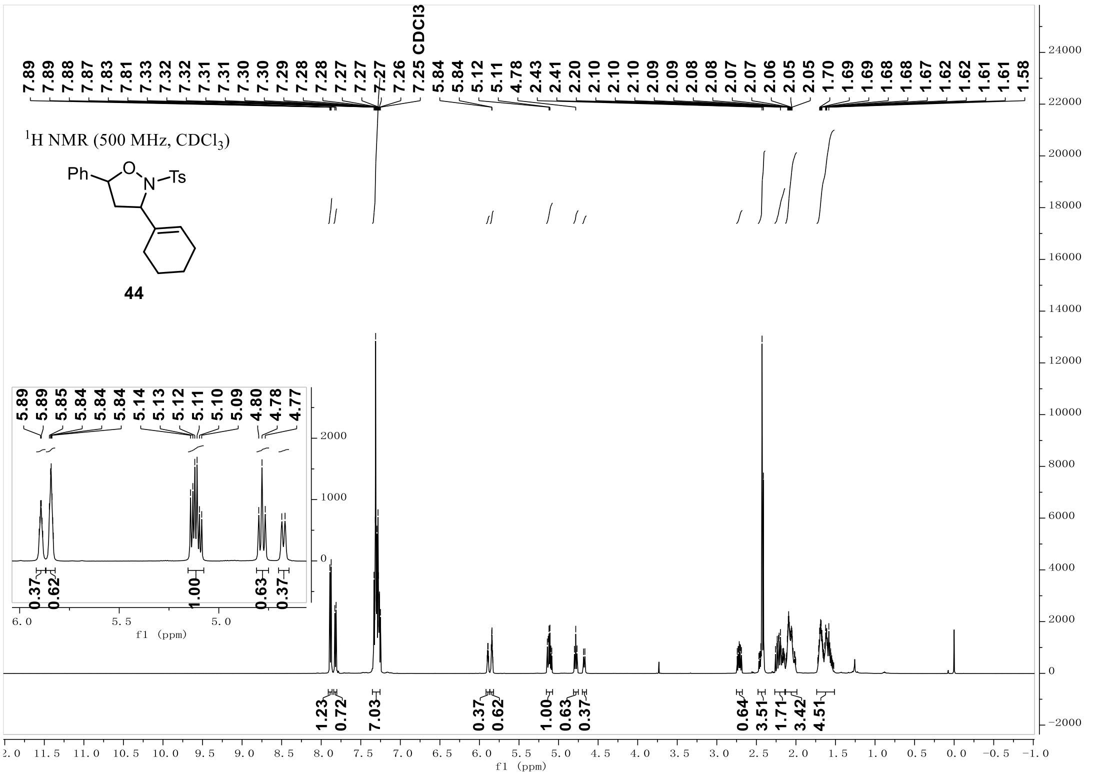

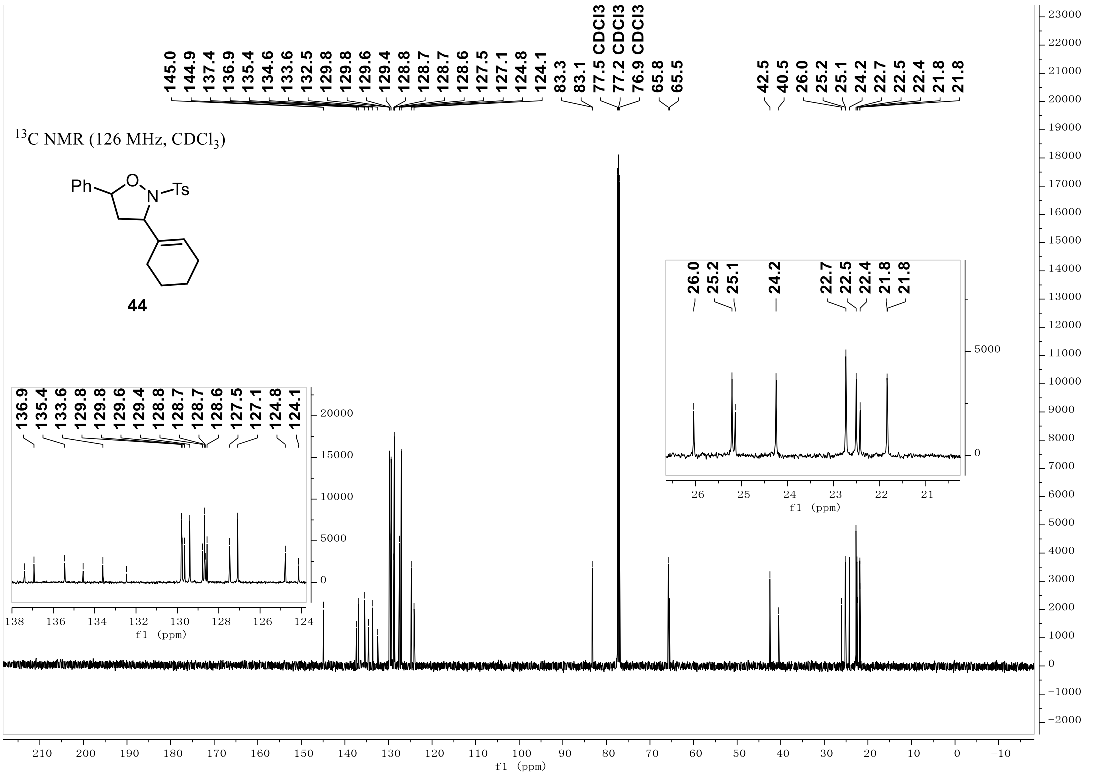

Supplementary Figure 44. ^{1}H NMR and ^{13}C NMR spectra of compound 44. The product was isolated as a 3:2 mixture of diastereomers.

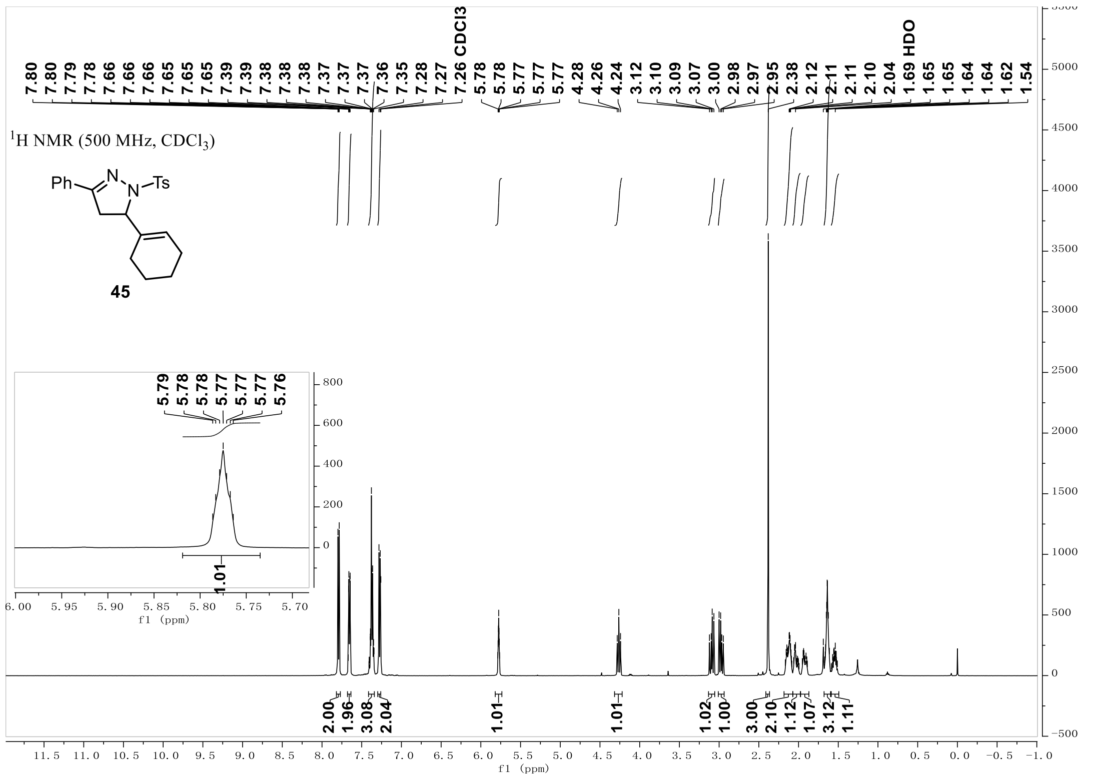

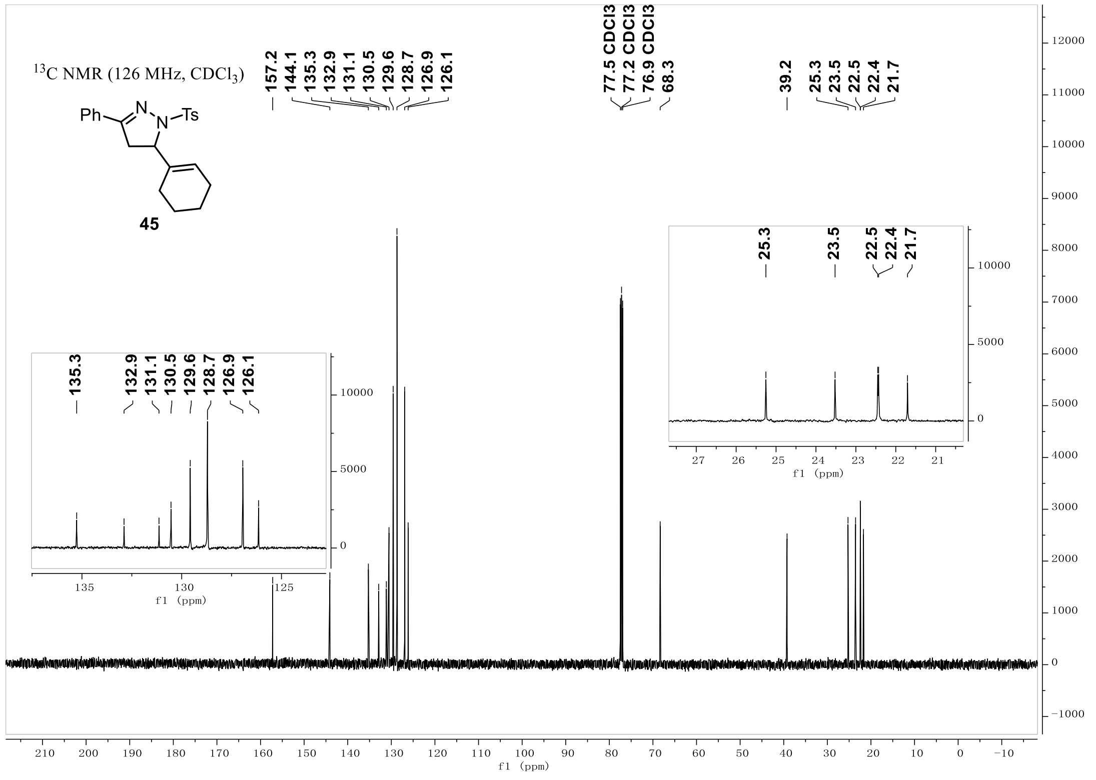

Supplementary Figure 45. ^{1}H NMR and ^{13}C NMR spectra of compound 45.

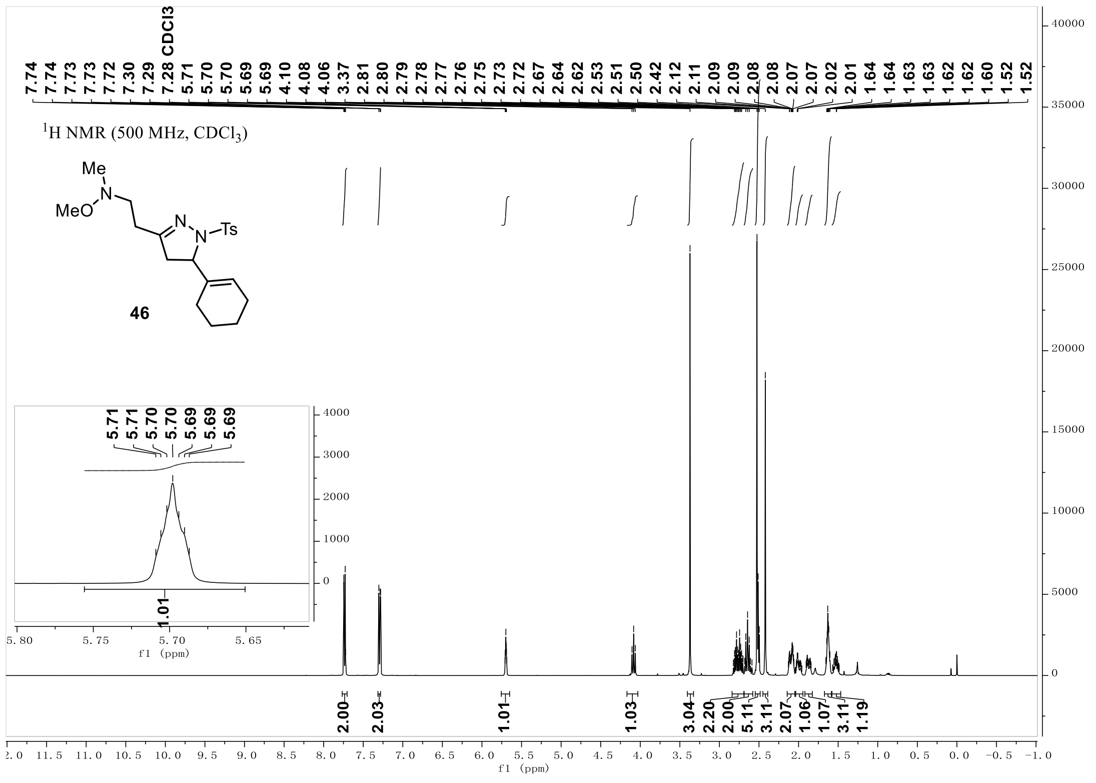

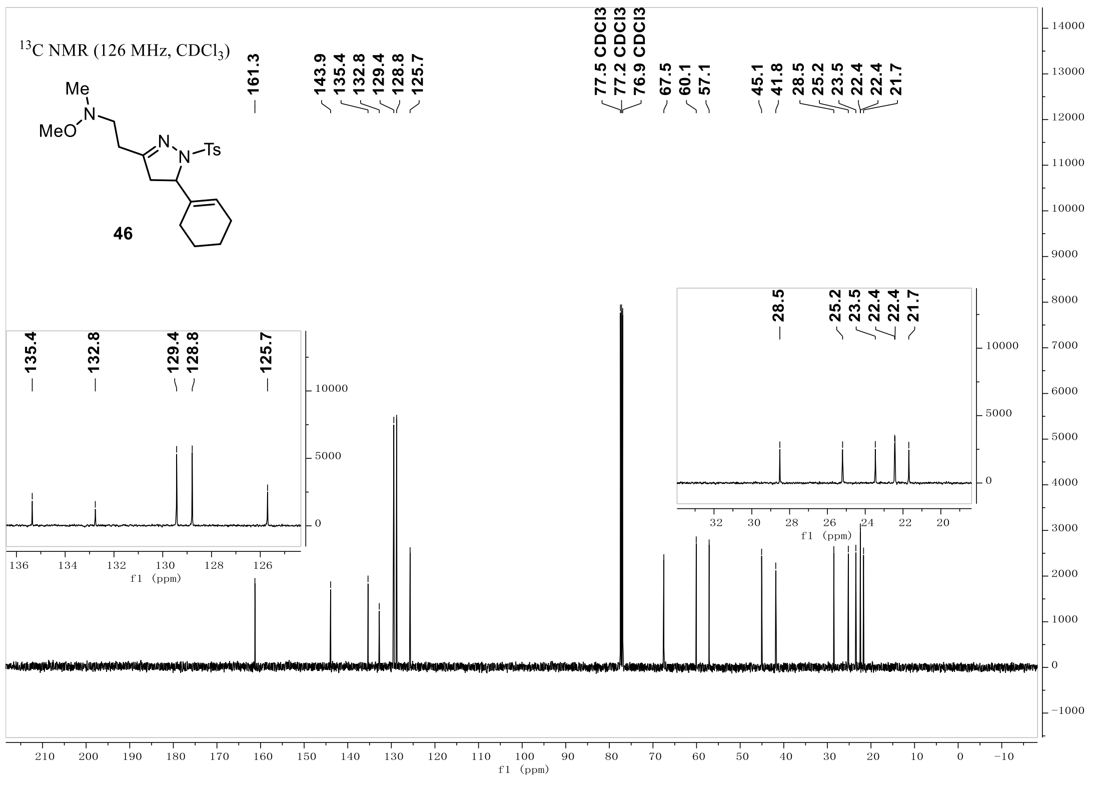

Supplementary Figure 46. ^{1}H NMR and ^{13}C NMR spectra of compound 46.

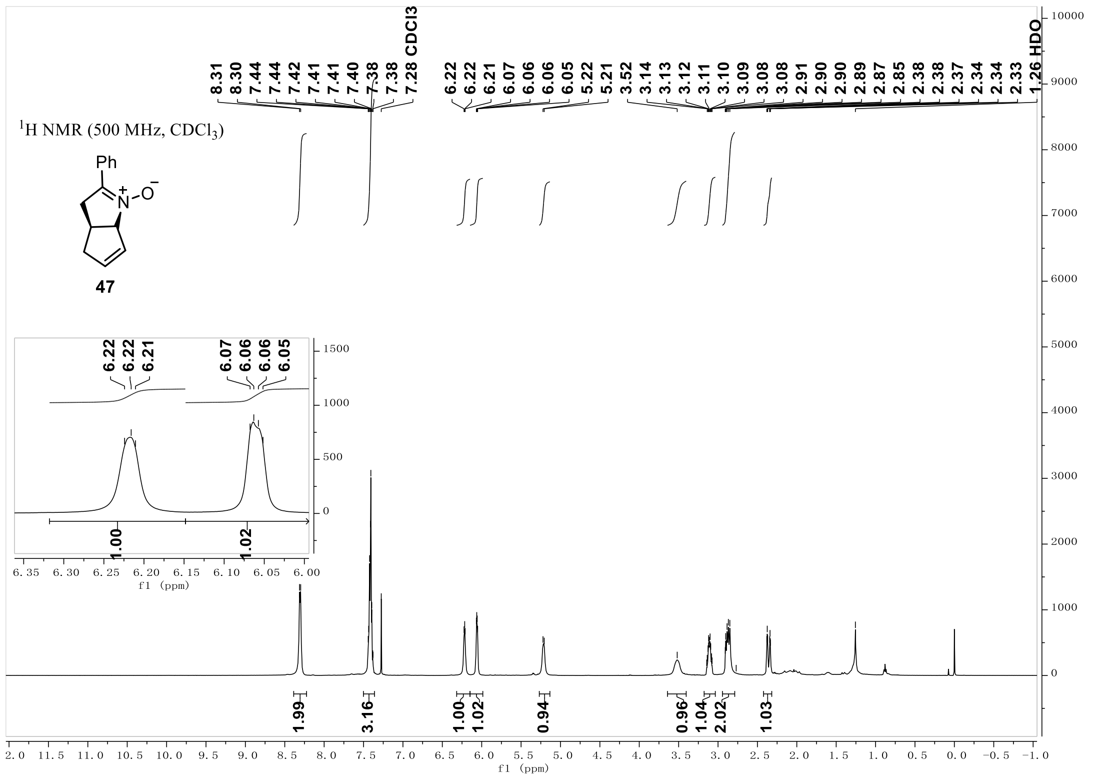

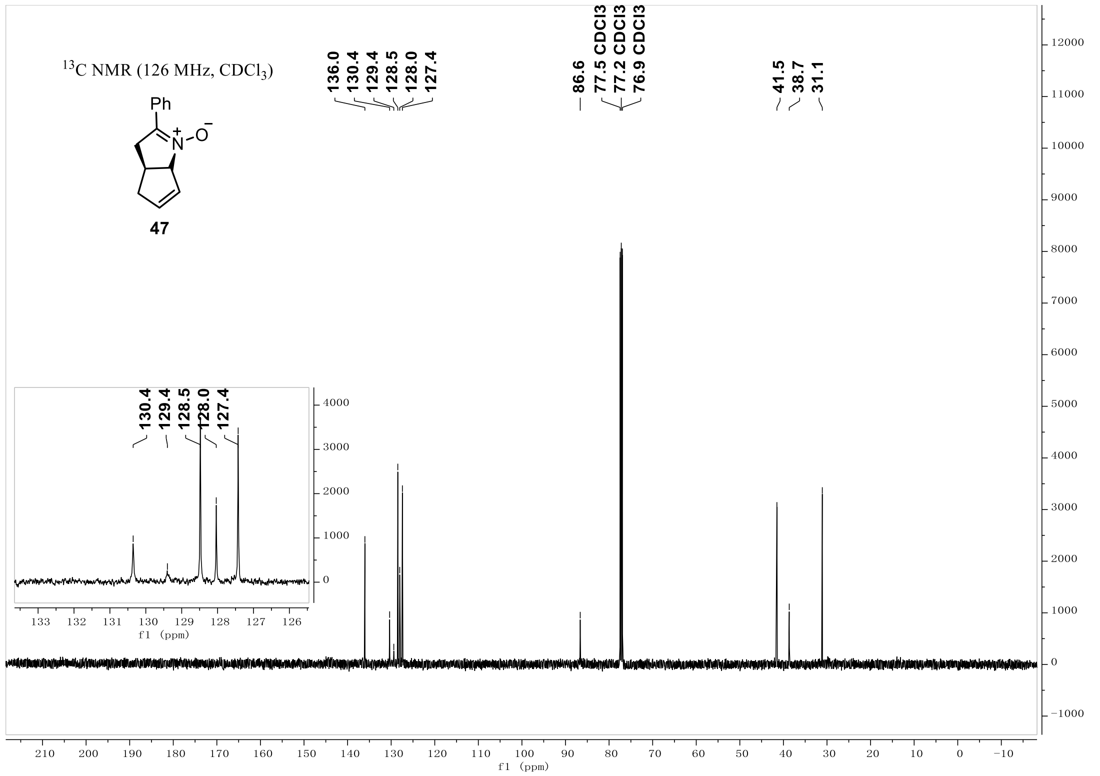

Supplementary Figure 47. ^{1}H NMR and ^{13}C NMR spectra of compound 47.

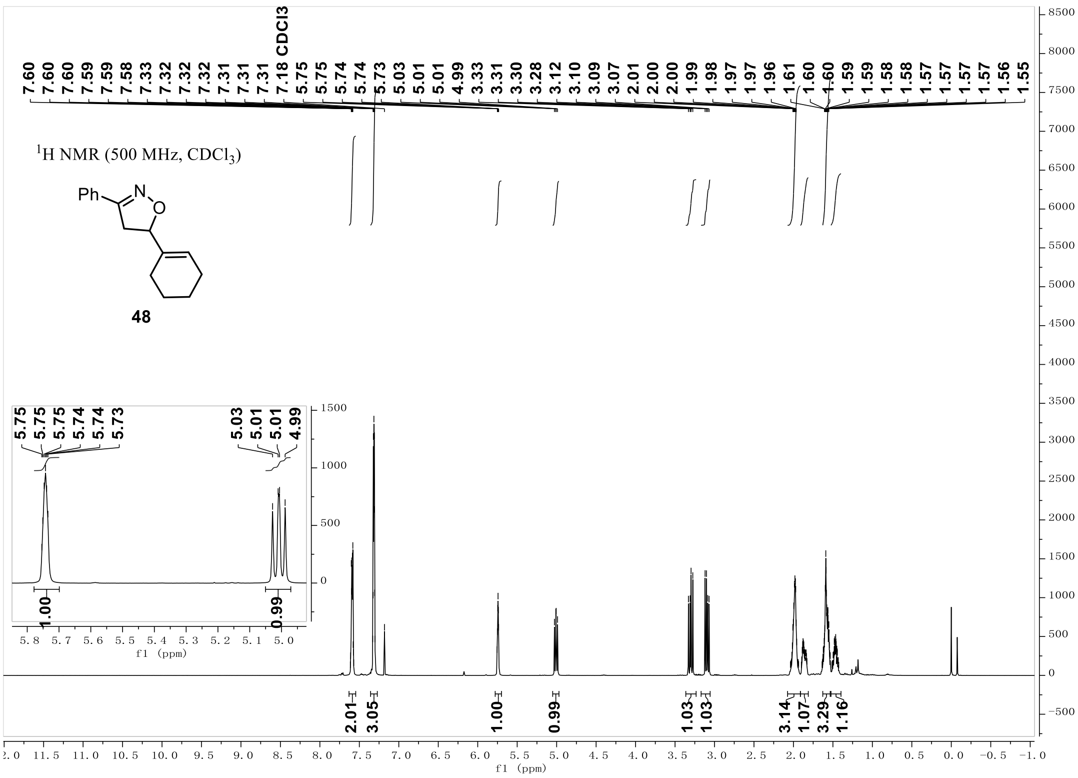

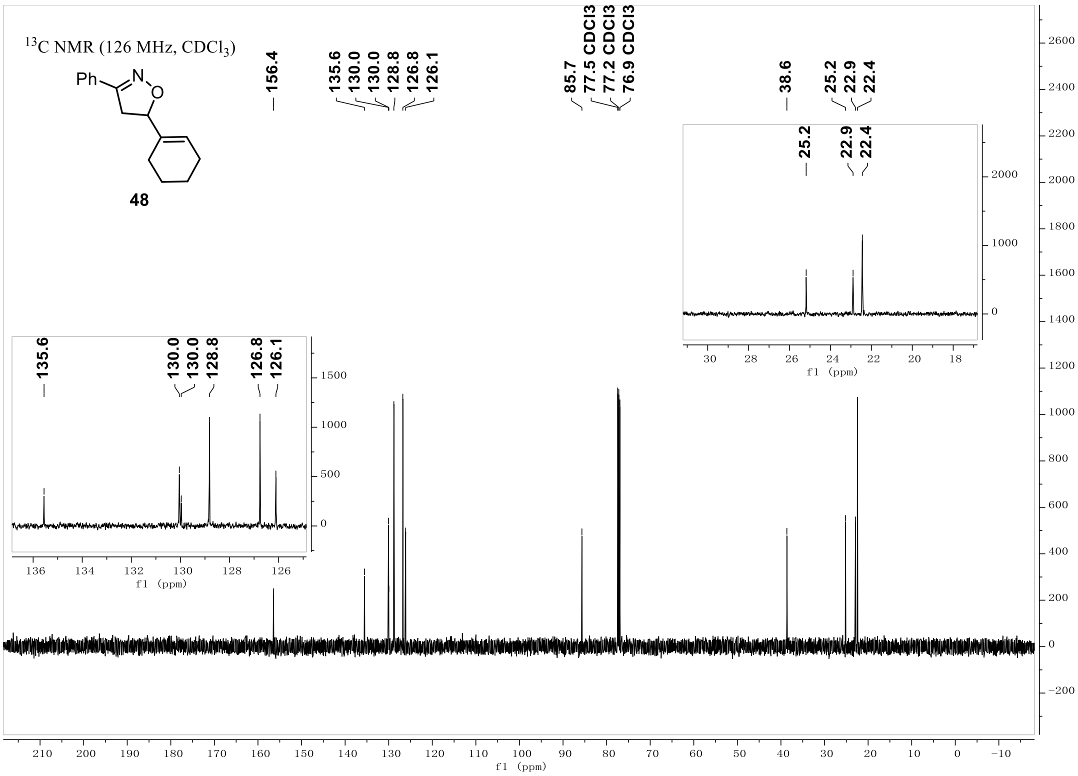

Supplementary Figure 48. ^{1}H NMR and ^{13}C NMR spectra of compound 48.

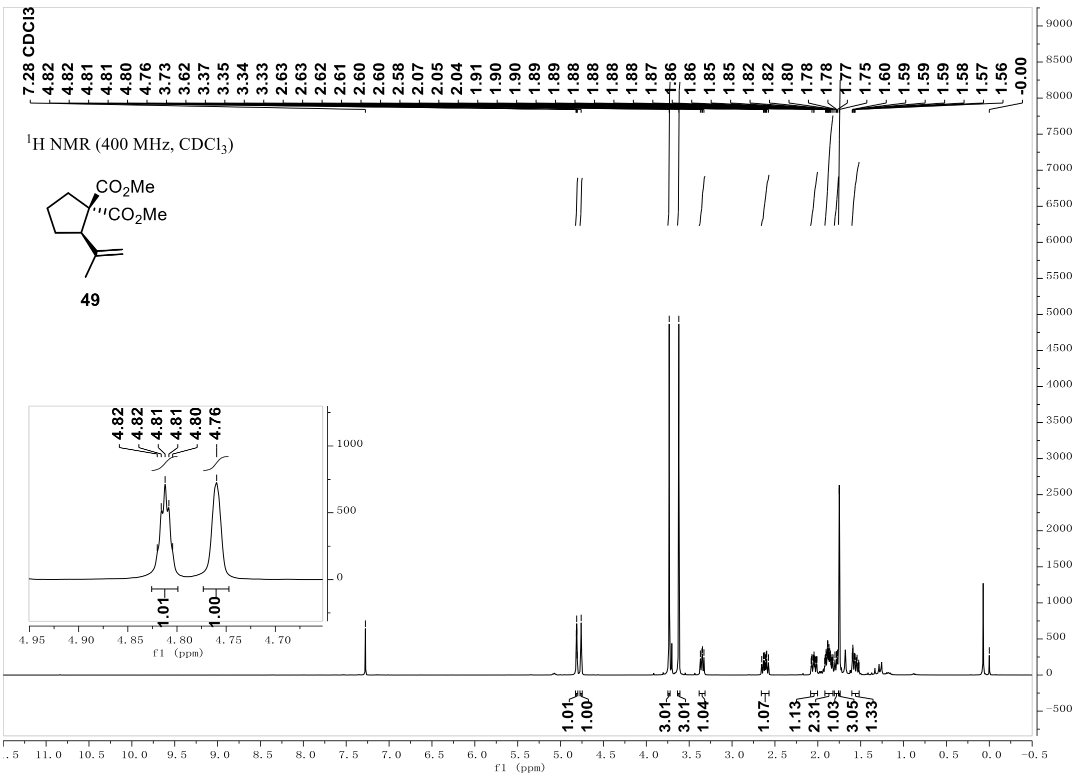

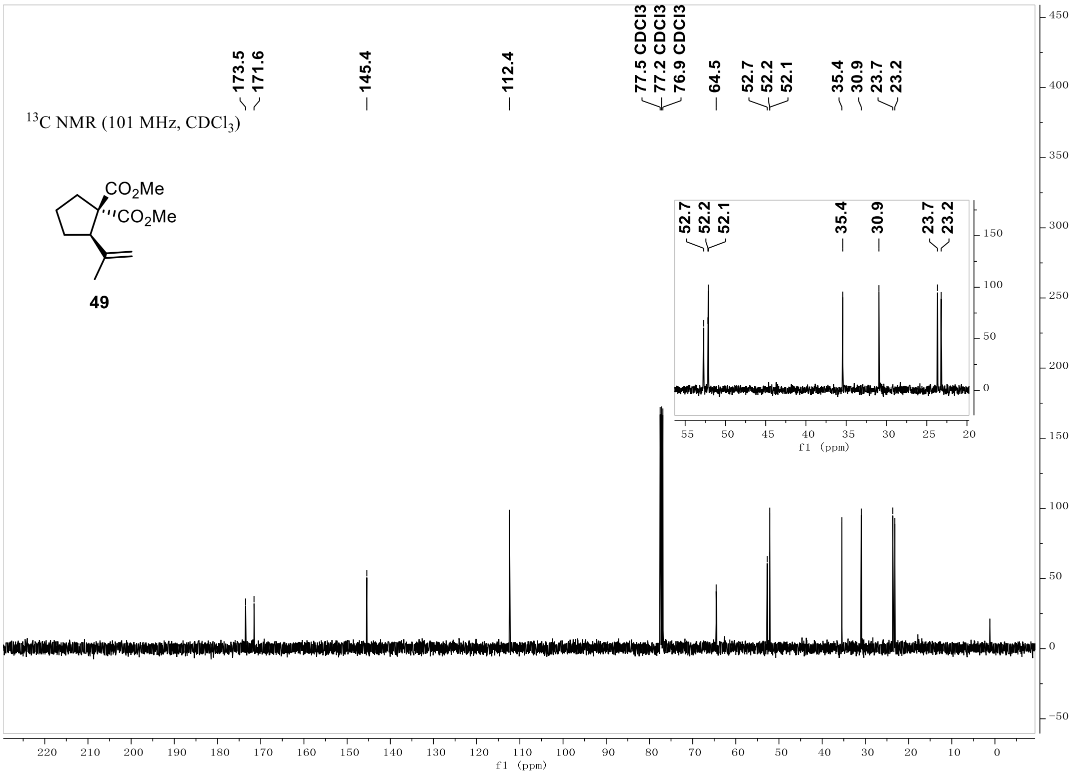

Supplementary Figure 49. ^{1}H NMR and ^{13}C NMR spectra of compound 49.

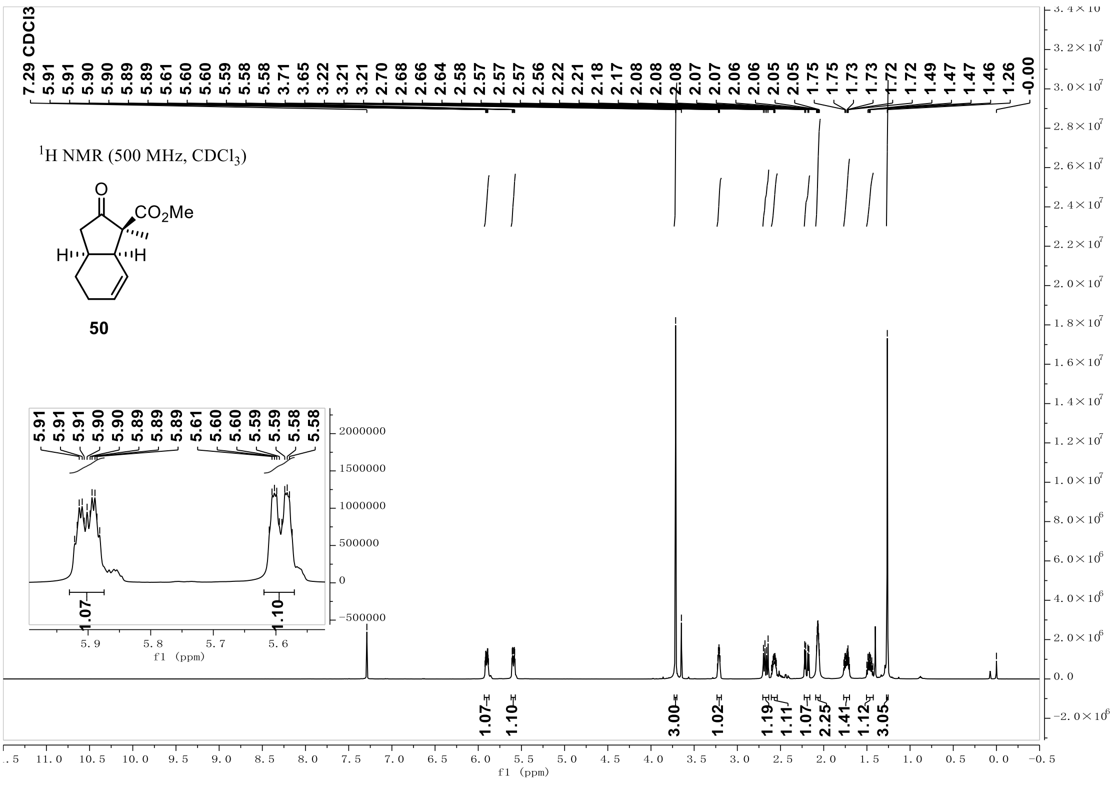

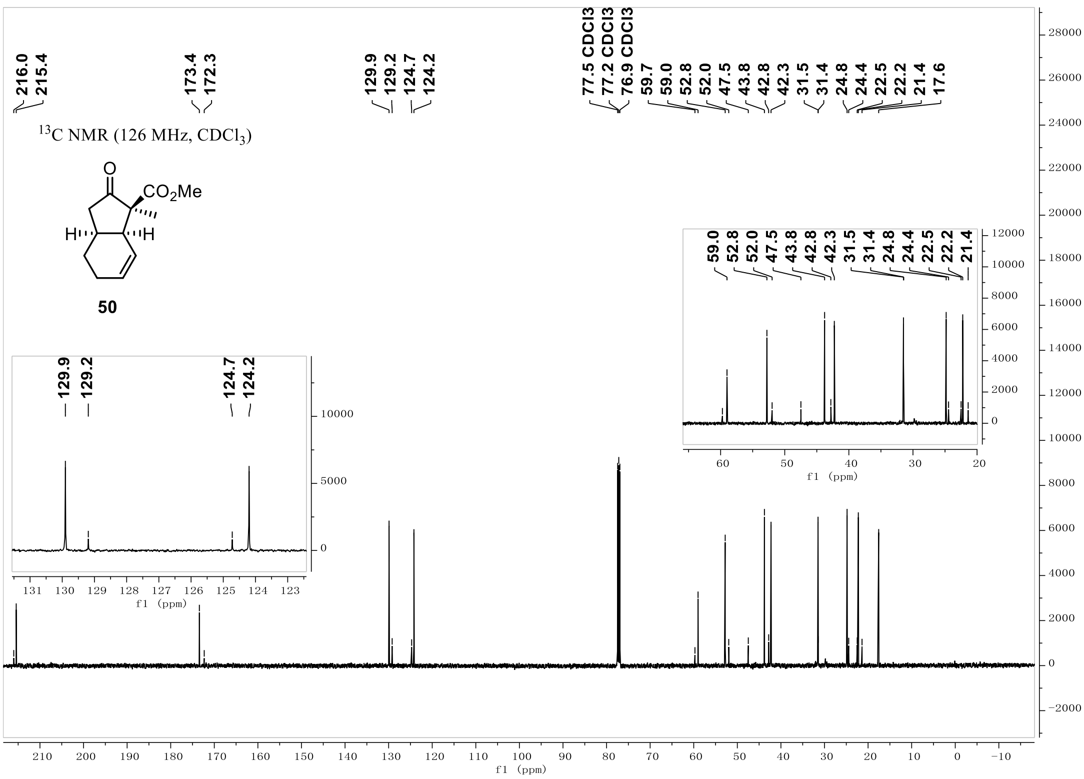

Supplementary Figure 50. ^{1}H NMR and ^{13}C NMR spectra of compound 50. The product was isolated as a 7:1 mixture of diastereomers.

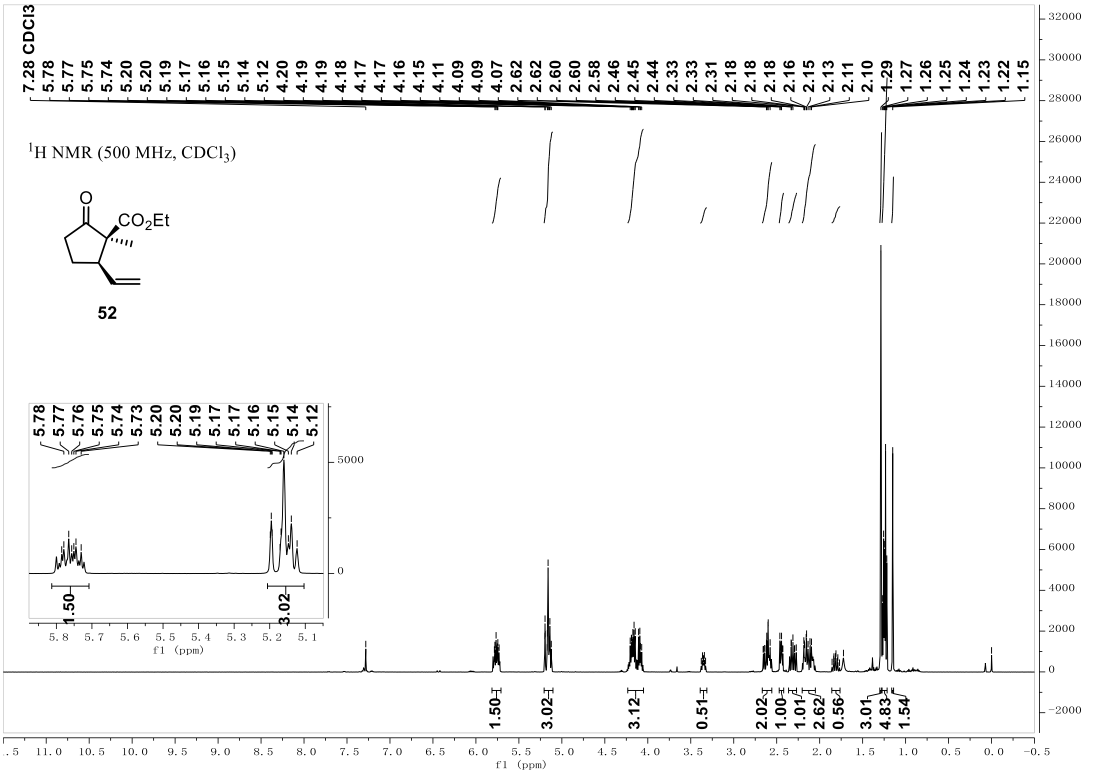

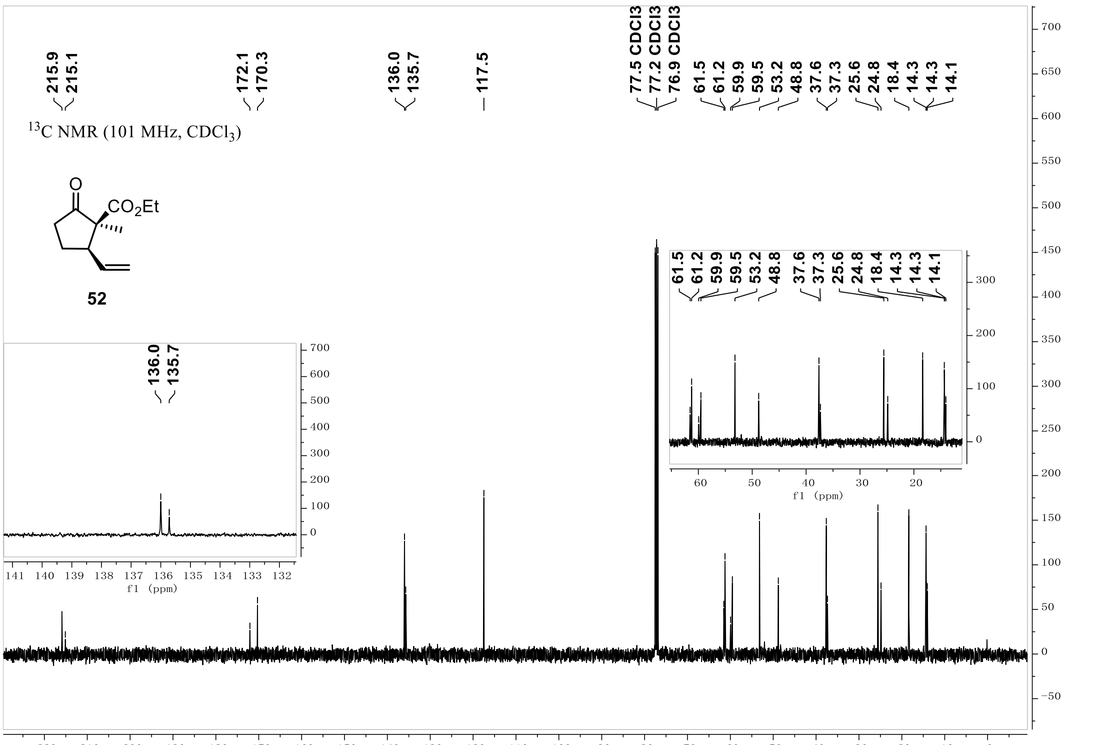

Supplementary Figure 51. ^1H NMR and ^{13}C NMR spectra of compound 52. The product was isolated as a 2:1 mixture of diastereomers.

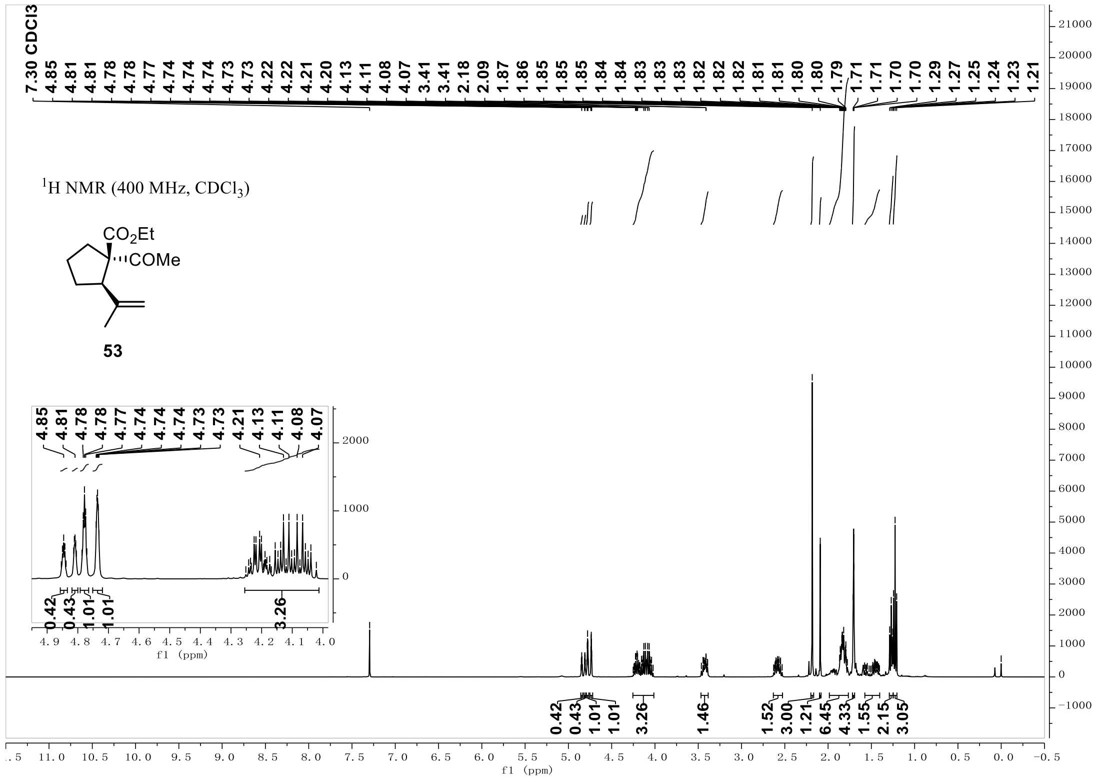

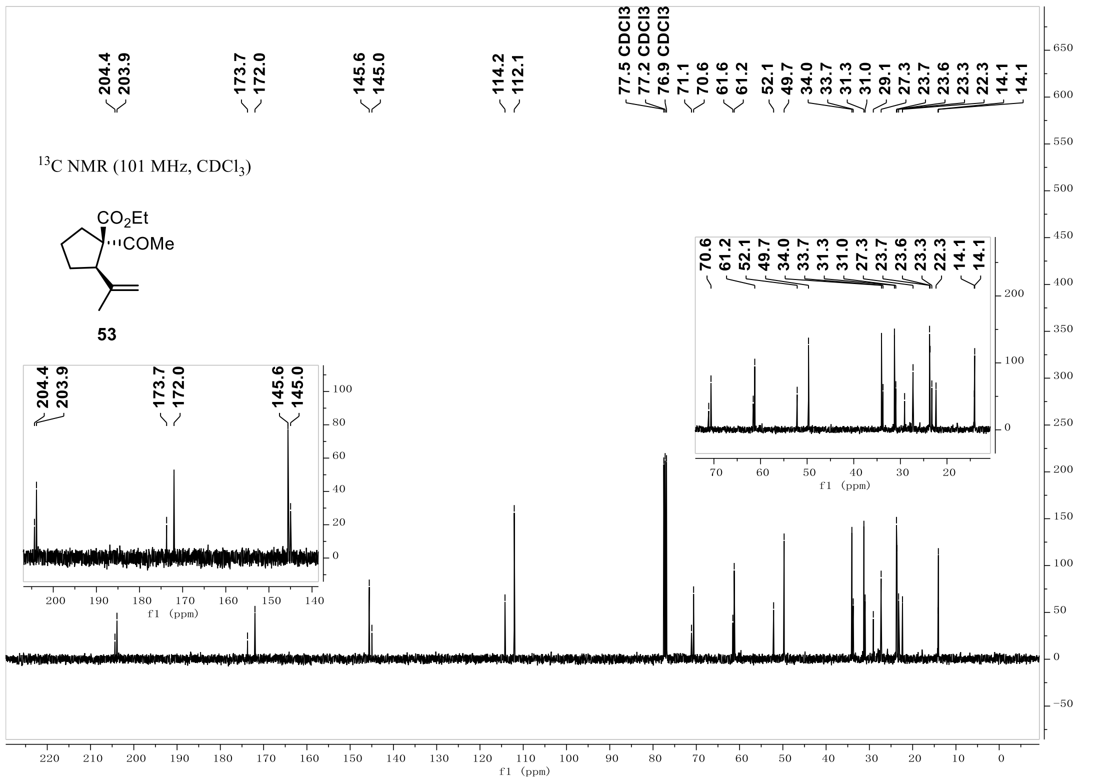

Supplementary Figure 52. ^{1}H NMR and ^{13}C NMR spectra of compound 53. The product was isolated as a 2.5:1 mixture of diastereomers.

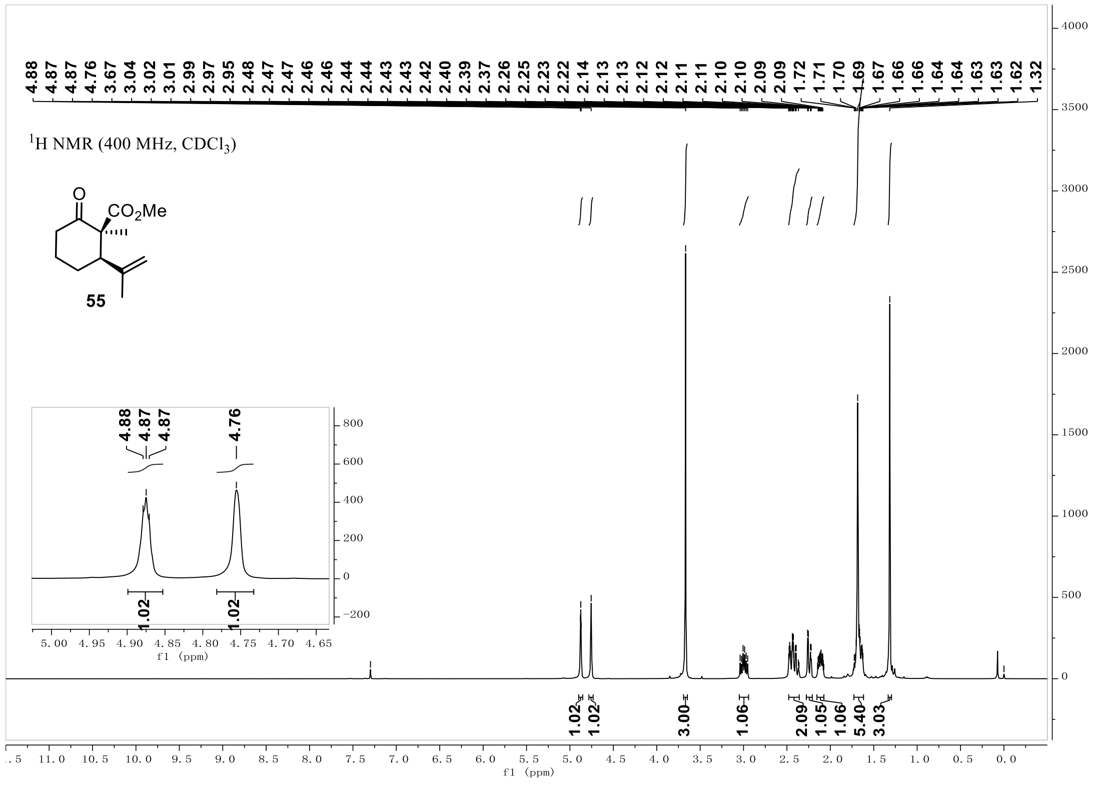

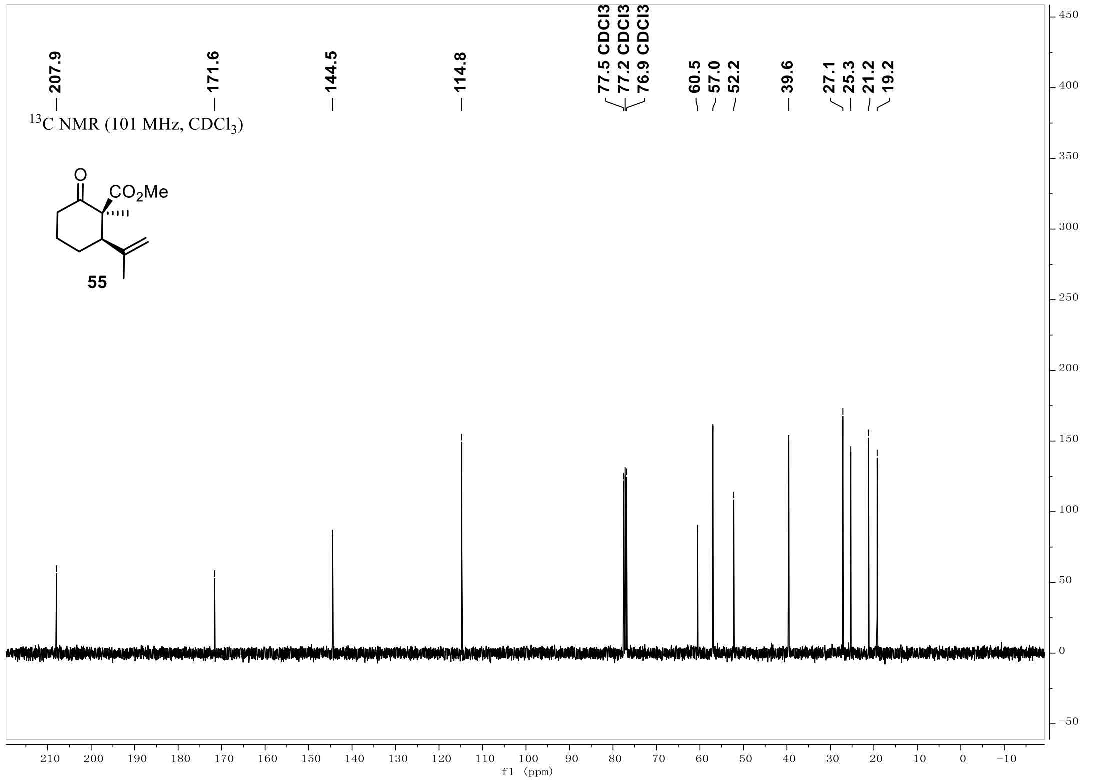

Supplementary Figure 53. ^{1}H NMR and ^{13}C NMR spectra of compound 55.

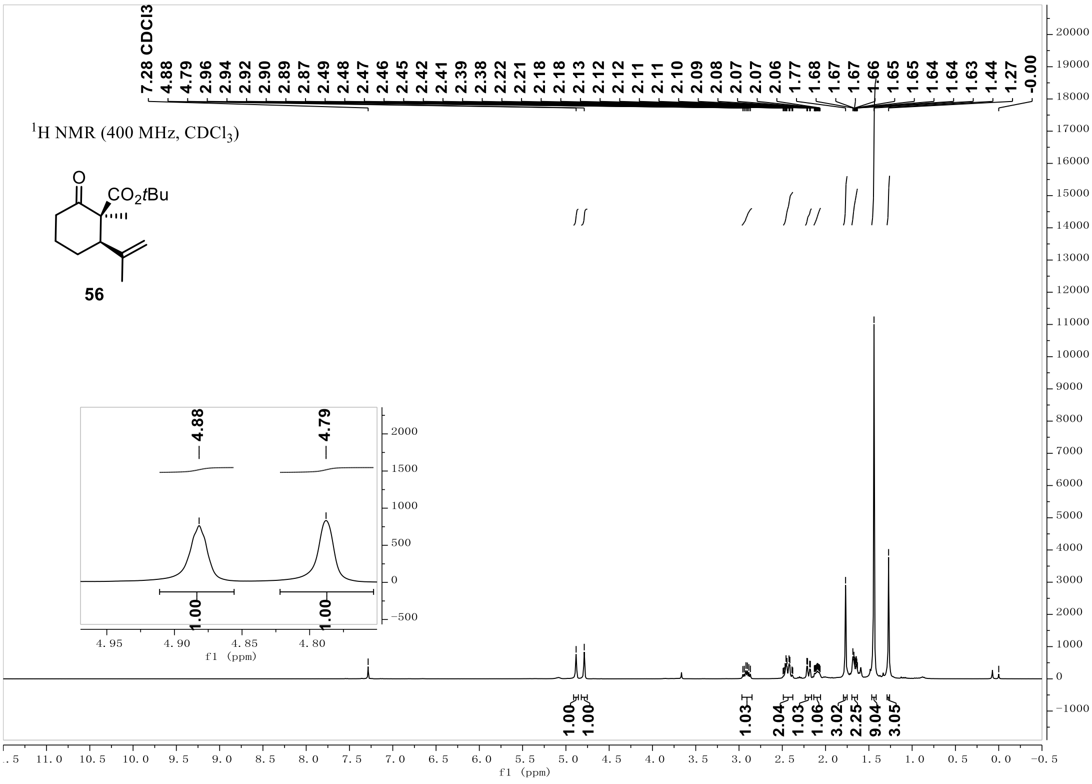

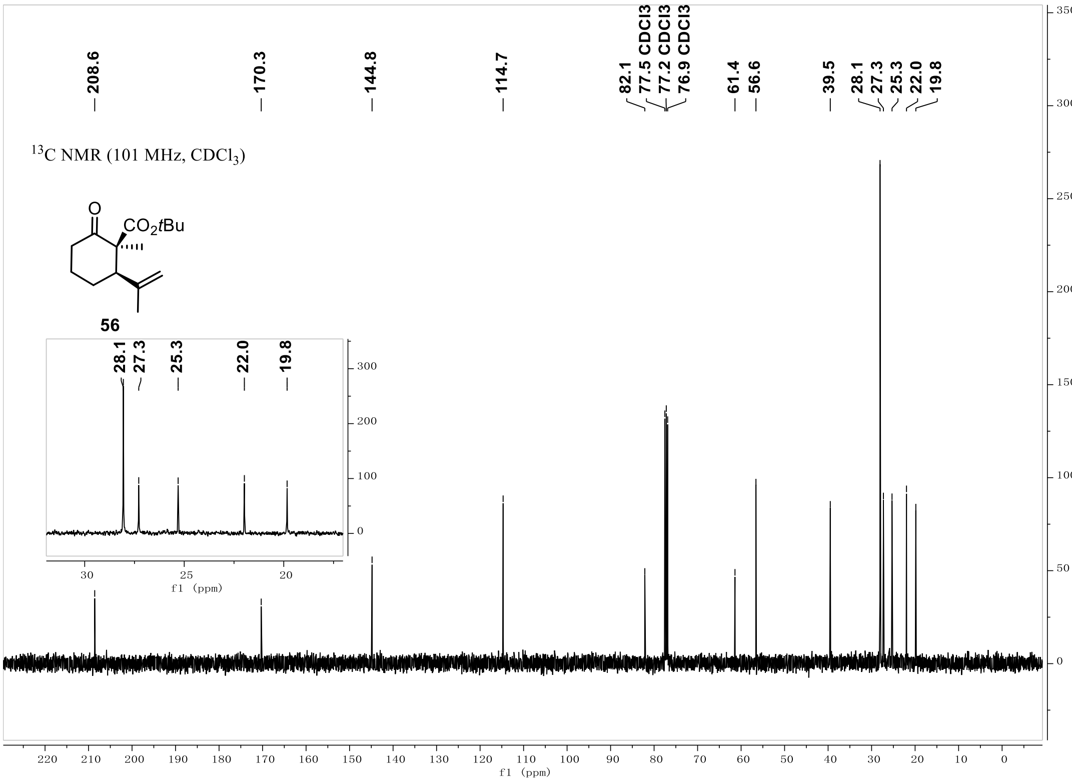

Supplementary Figure 54. ^{1}H NMR and ^{13}C NMR spectra of compound 56.

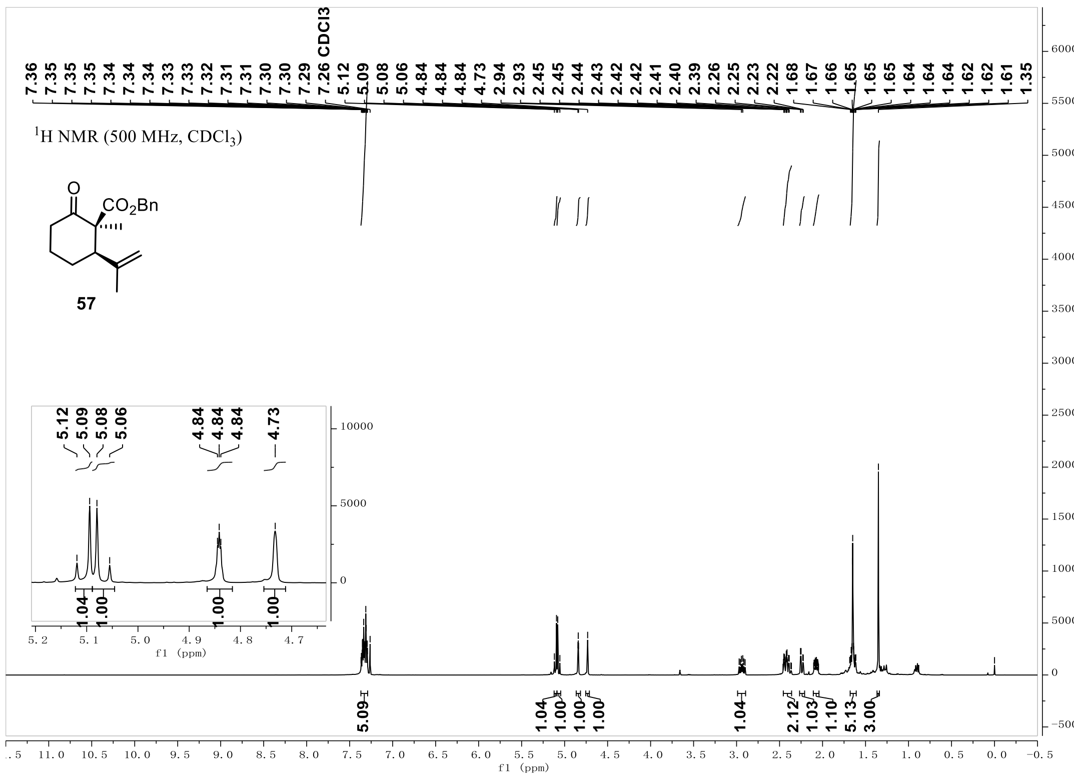

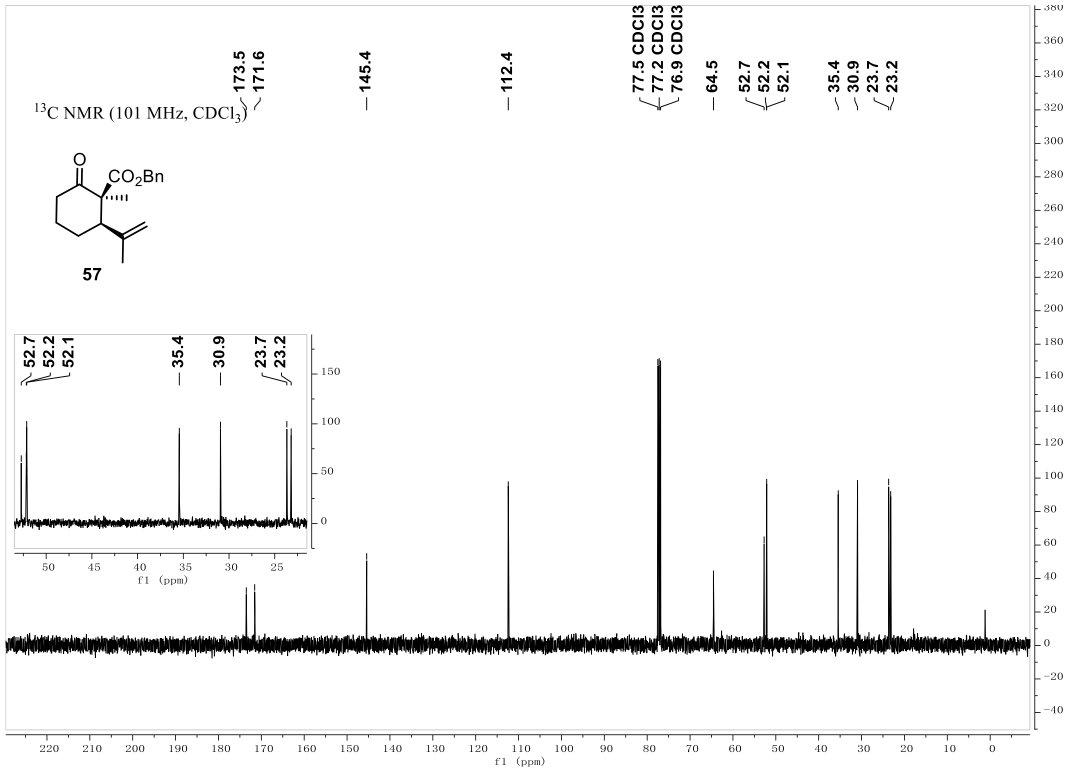

Supplementary Figure 55. ^{1}H NMR and ^{13}C NMR spectra of compound 57.

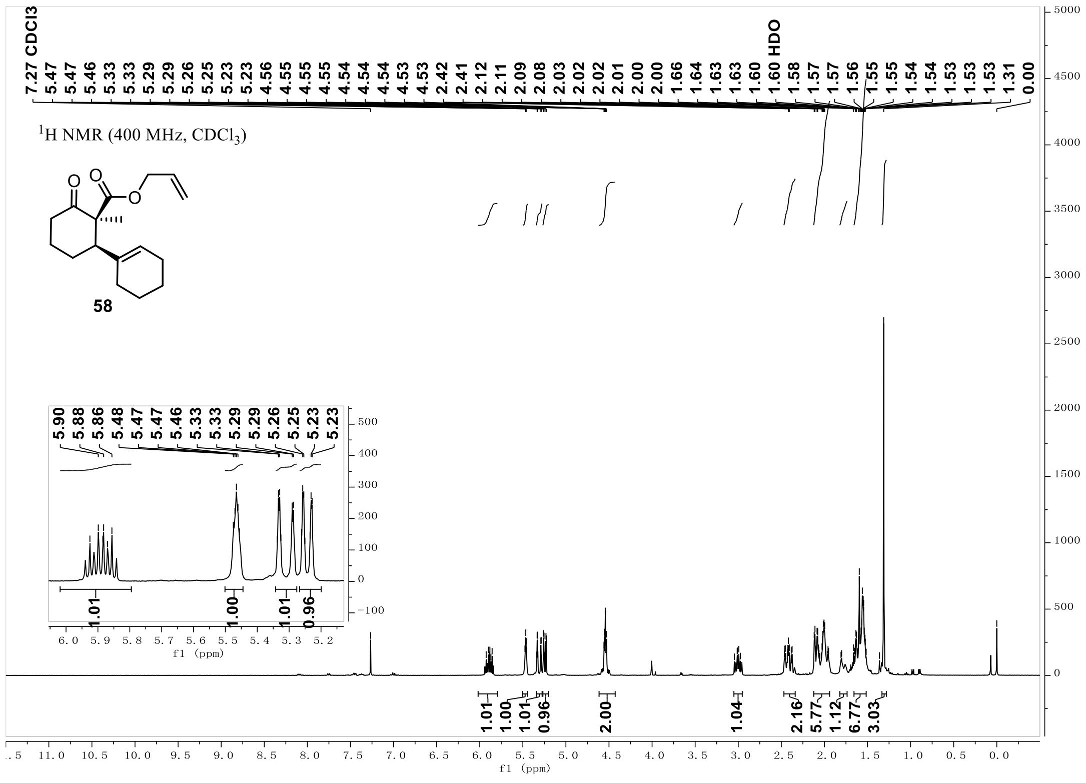

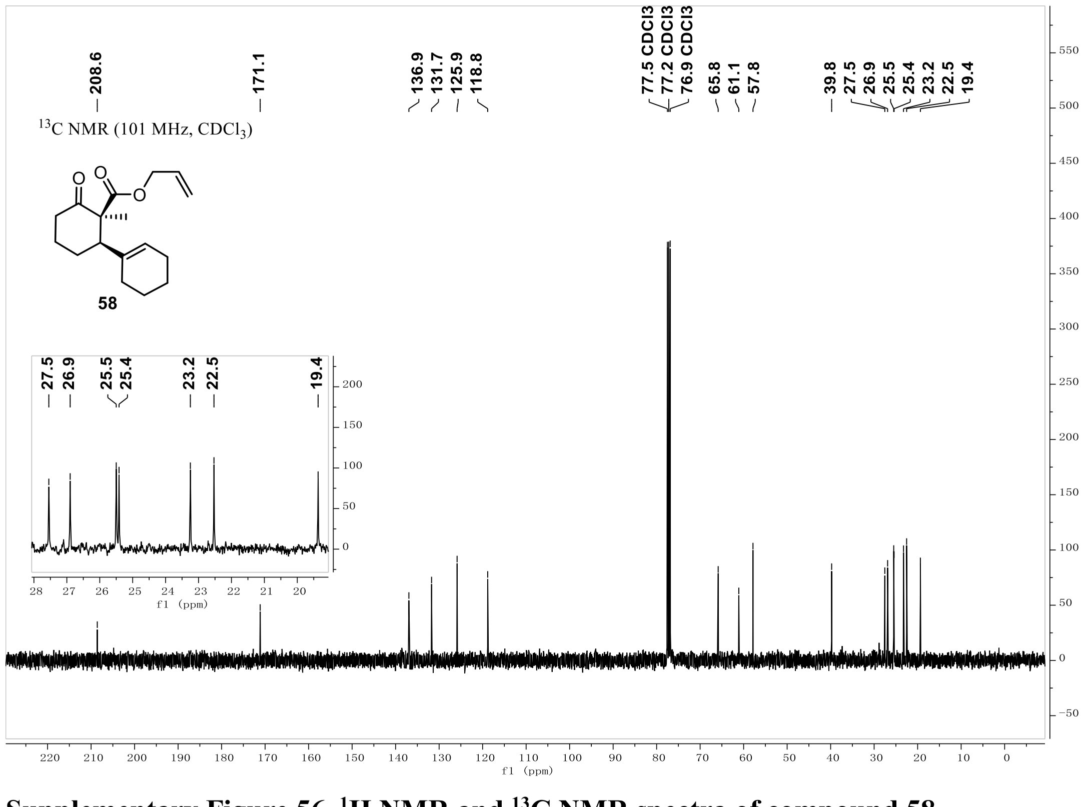

Supplementary Figure 56. ^{1}H NMR and ^{13}C NMR spectra of compound 58.

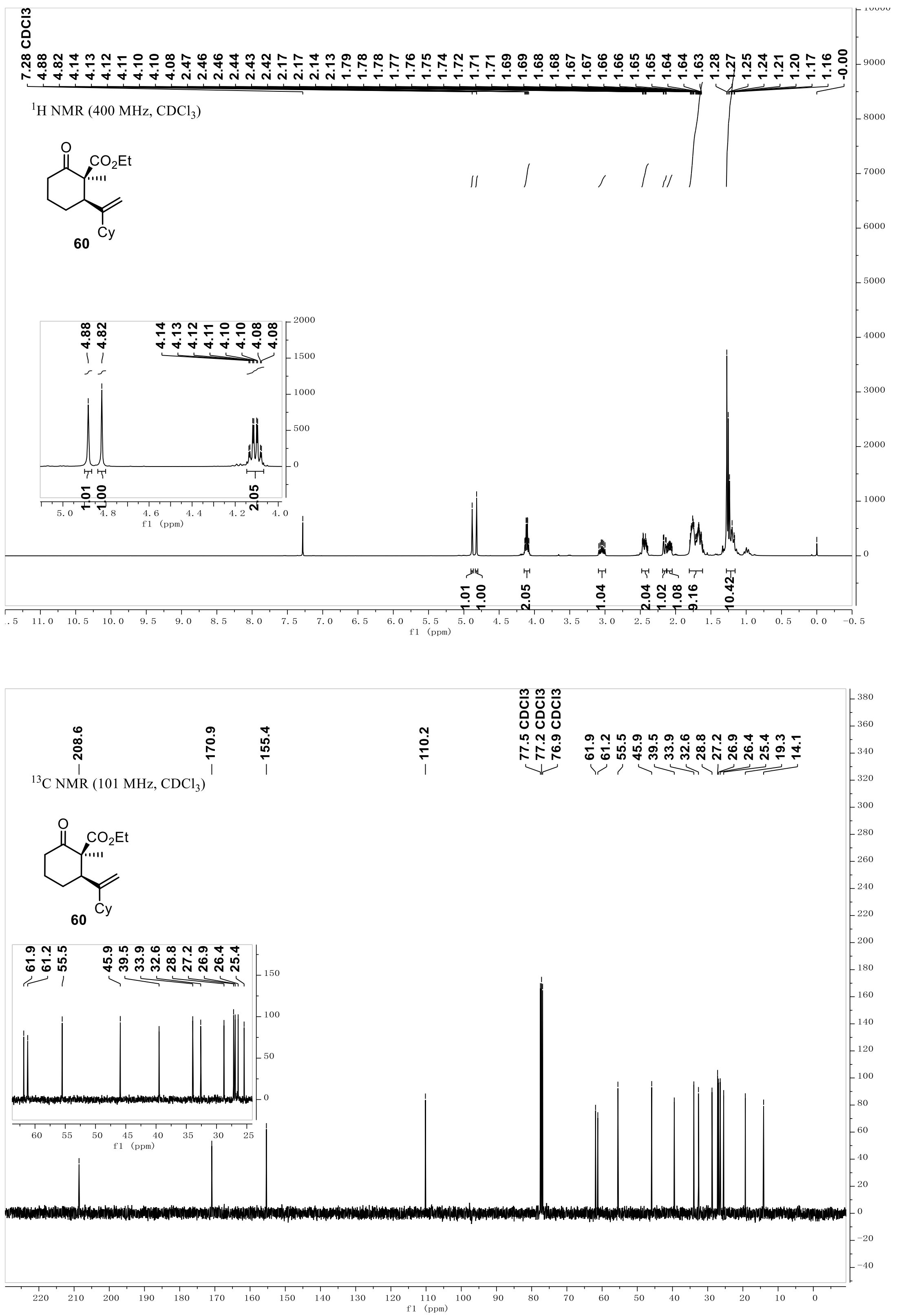

Supplementary Figure 57. ^{1}H NMR and ^{13}C NMR spectra of compound 60.

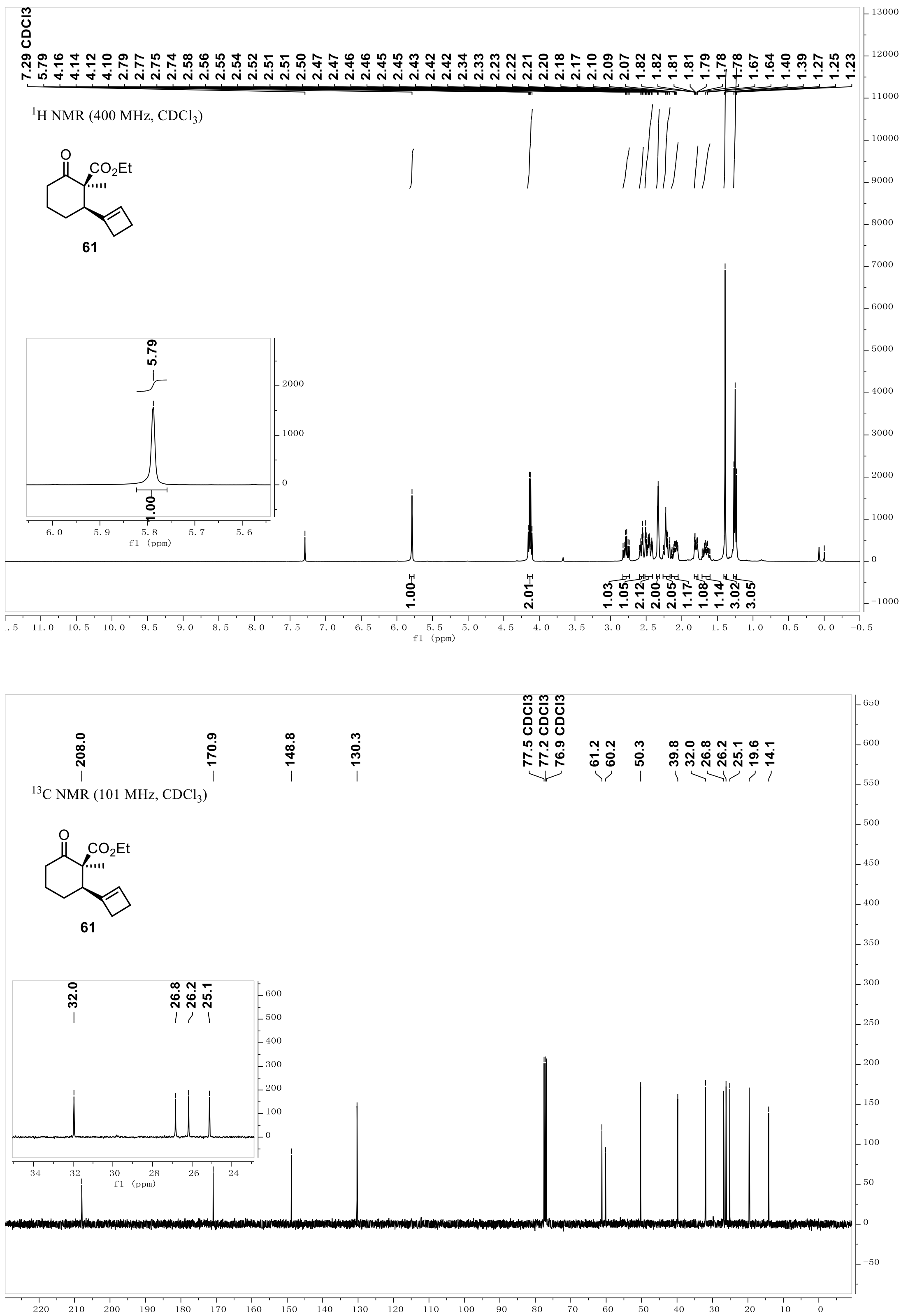

Supplementary Figure 58. ^{1}H **NMR and** ^{13}C **NMR spectra of compound 61.**

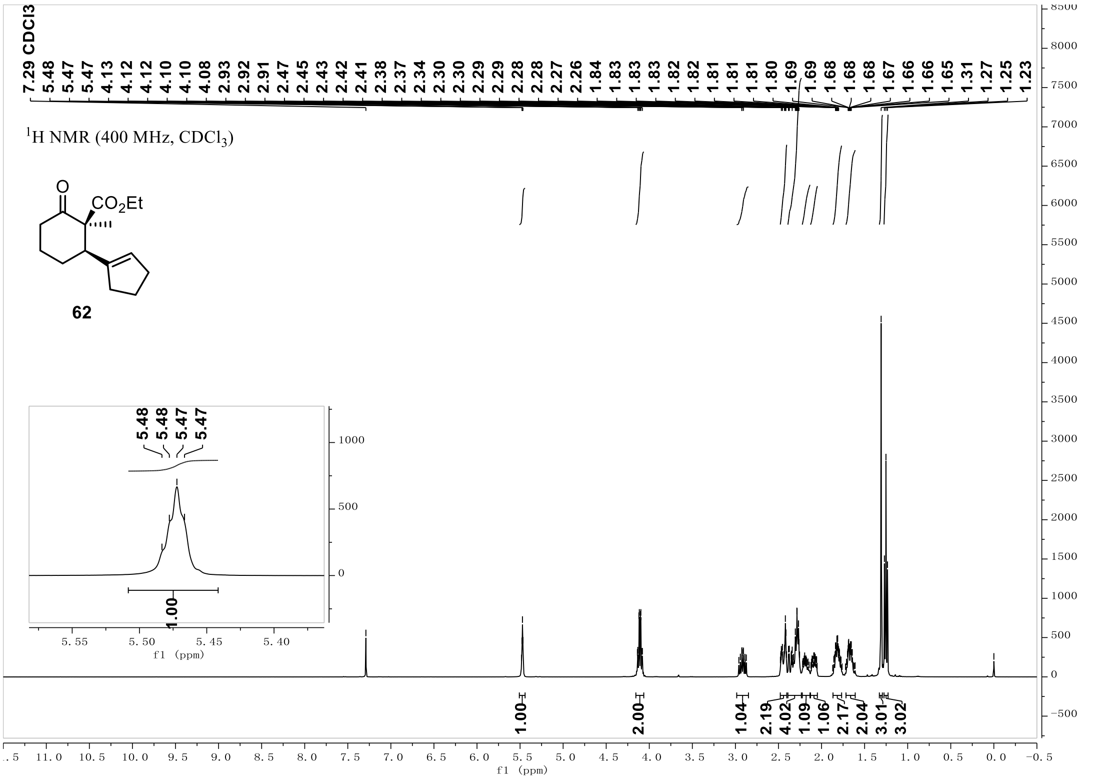

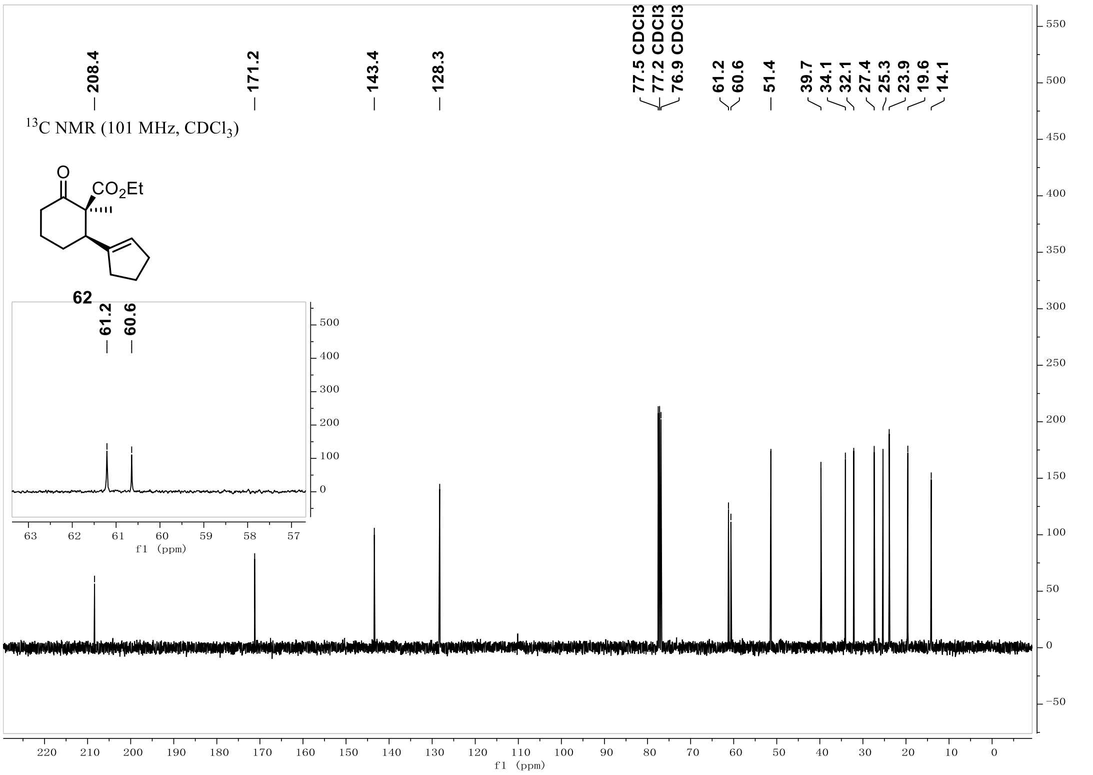

Supplementary Figure 59. ^{1}H NMR and ^{13}C NMR spectra of compound 62.

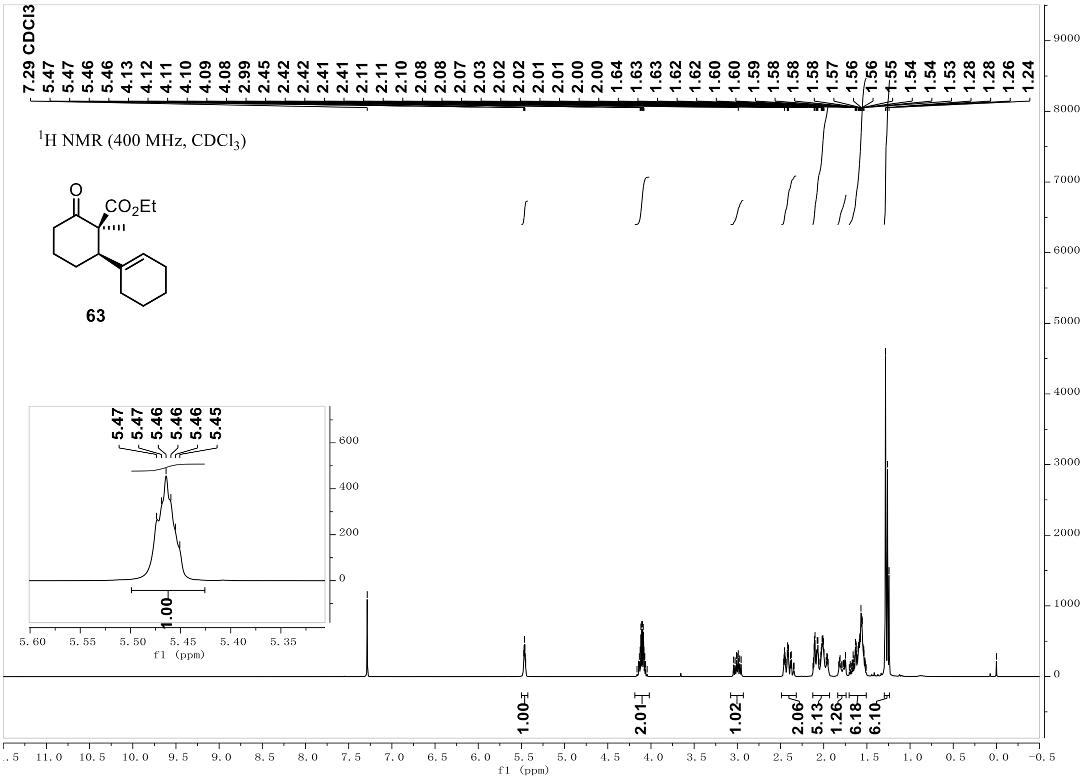

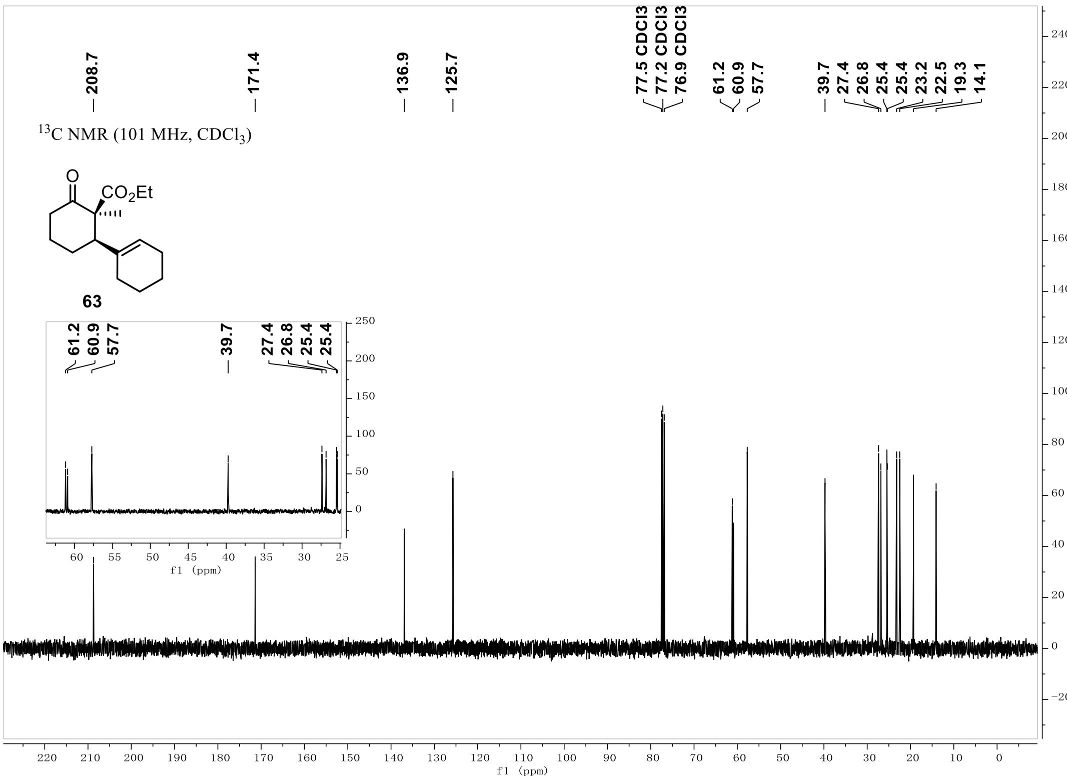

Supplementary Figure 60. ^{1}H NMR and ^{13}C NMR spectra of compound 63.

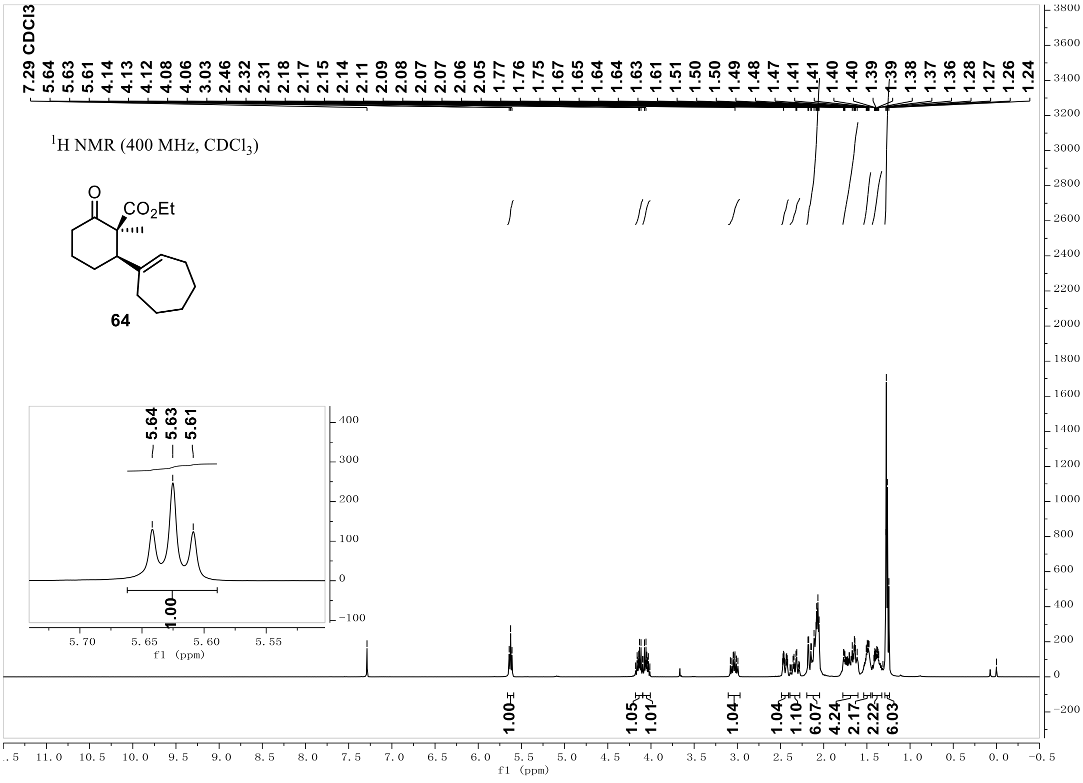

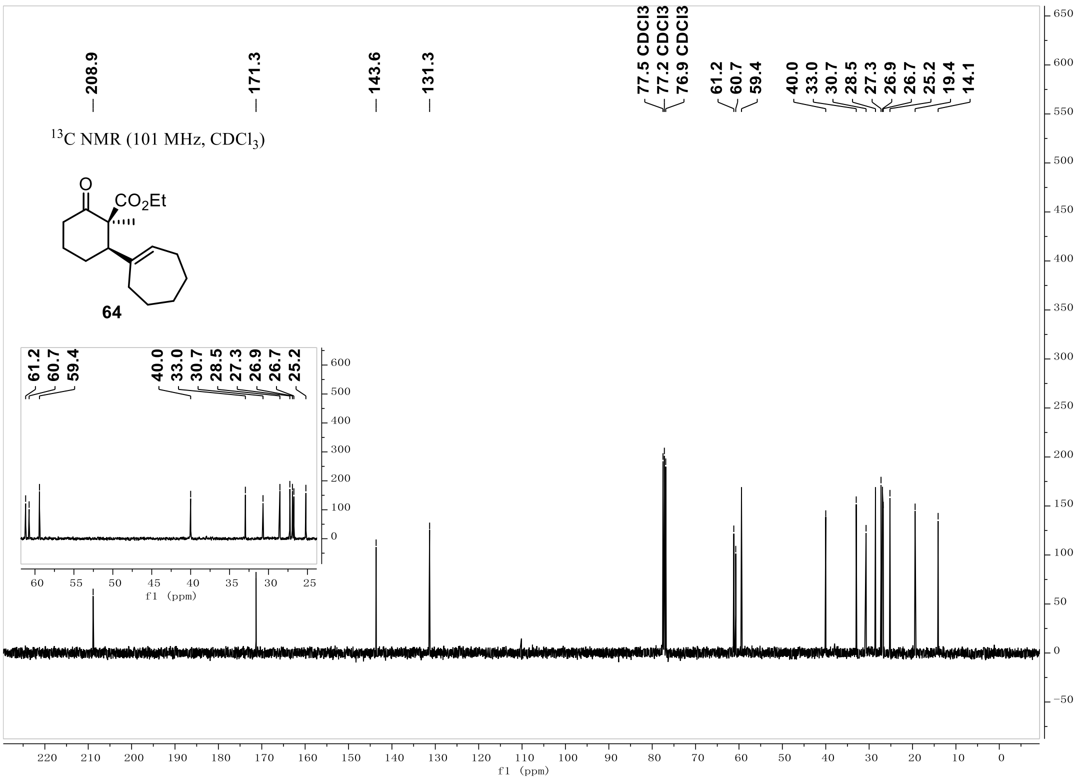

Supplementary Figure 61. ^{1}H NMR and ^{13}C NMR spectra of compound 64.

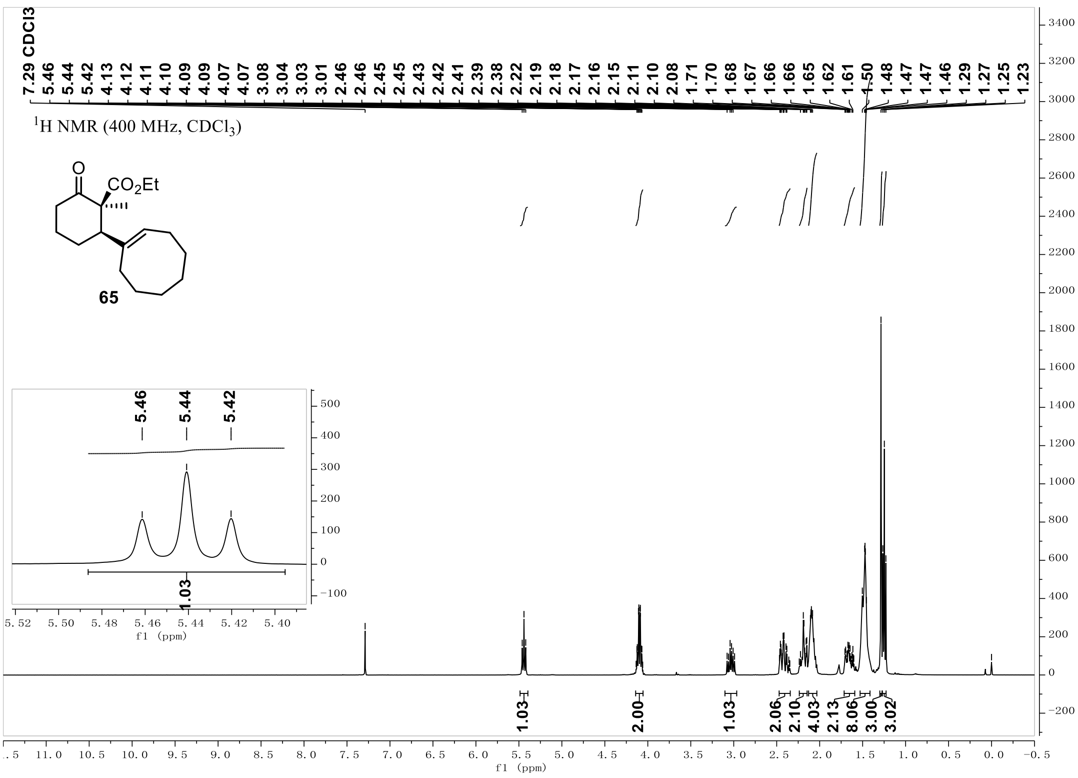

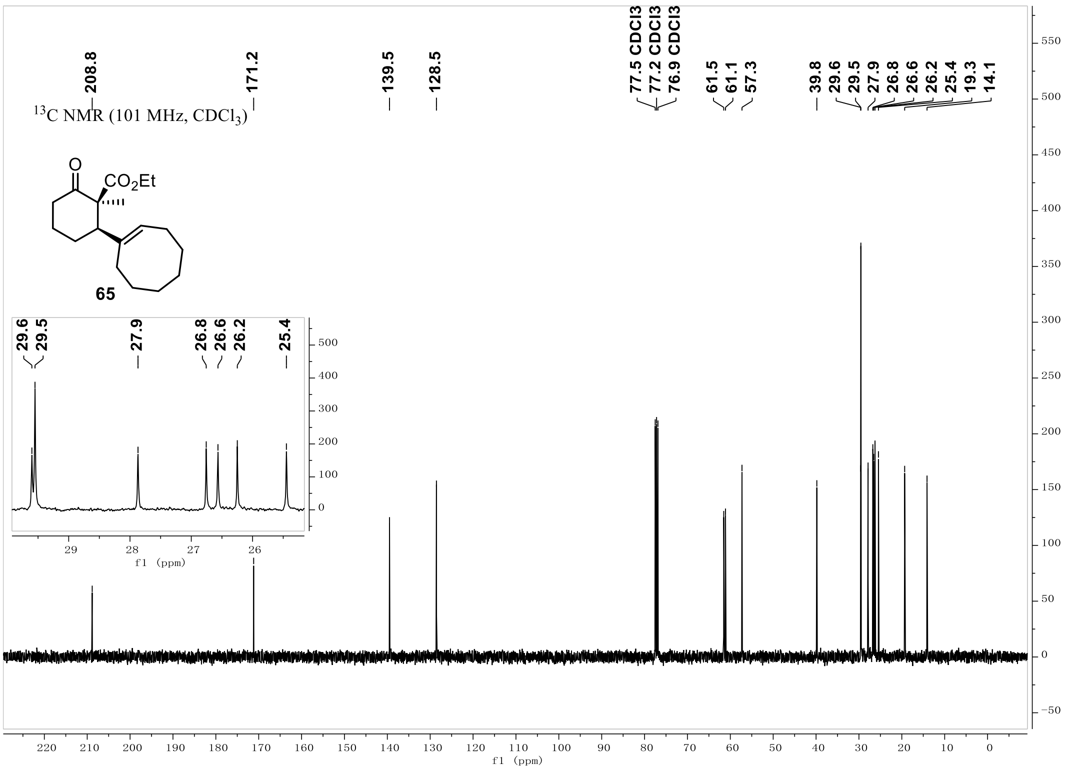

Supplementary Figure 62. ^{1}H NMR and ^{13}C NMR spectra of compound 65.

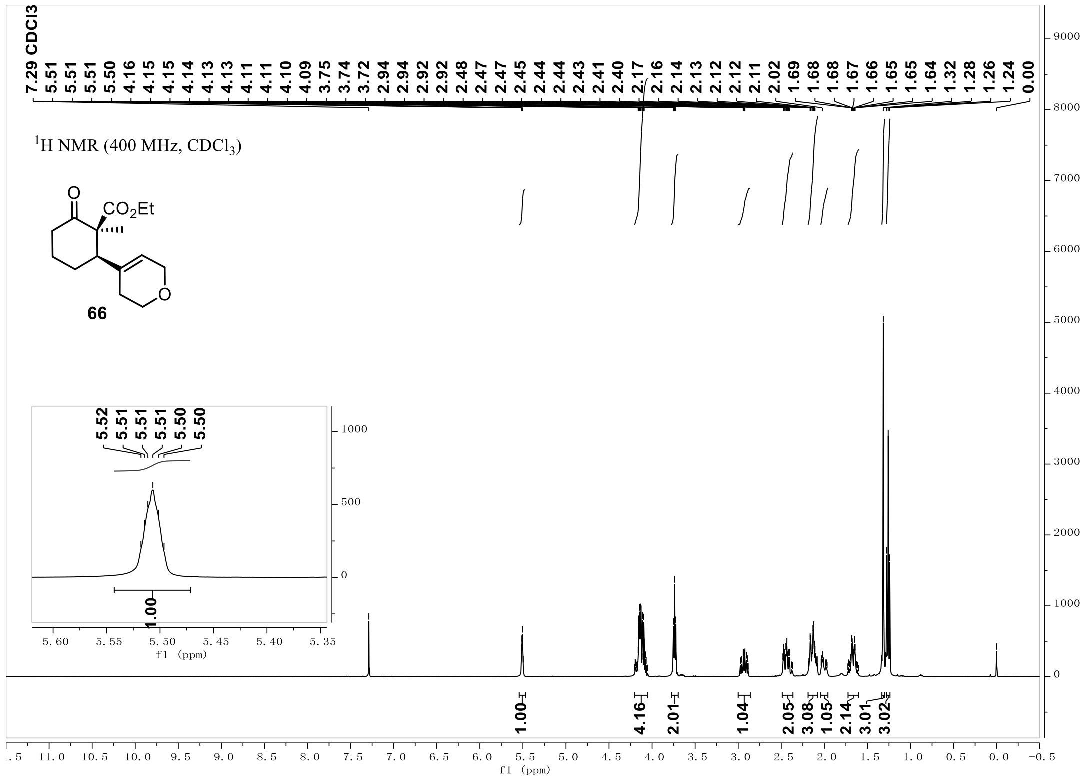

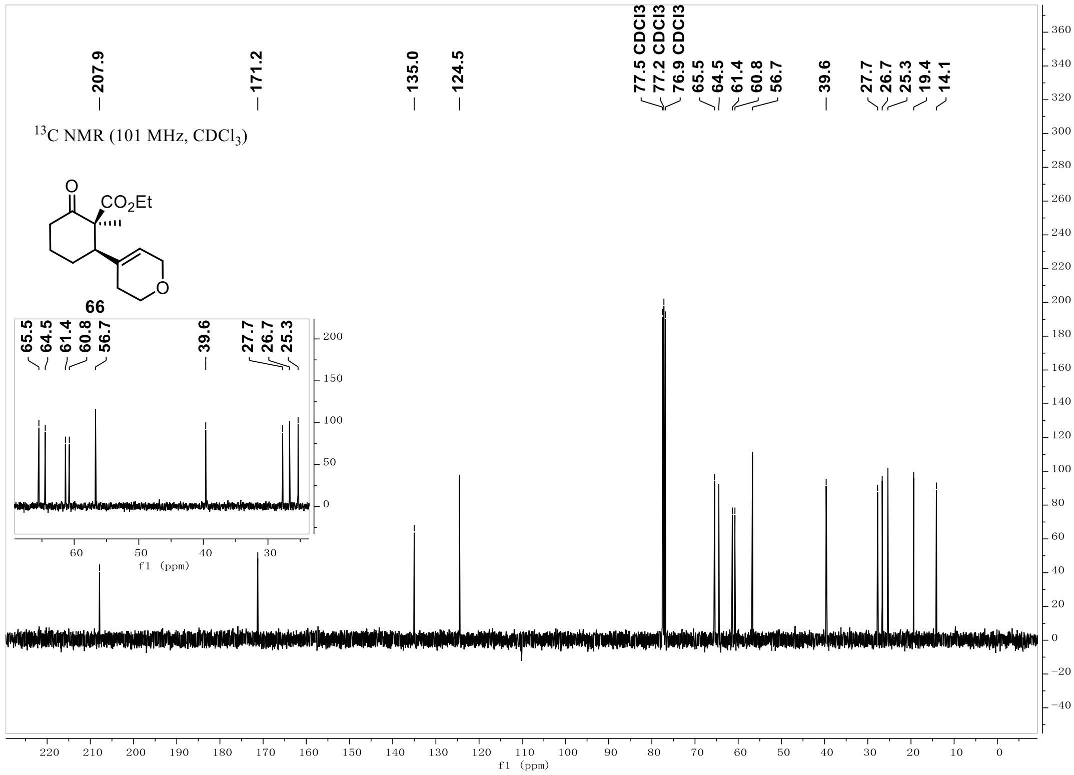

Supplementary Figure 63. ^{1}H NMR and ^{13}C NMR spectra of compound 66.

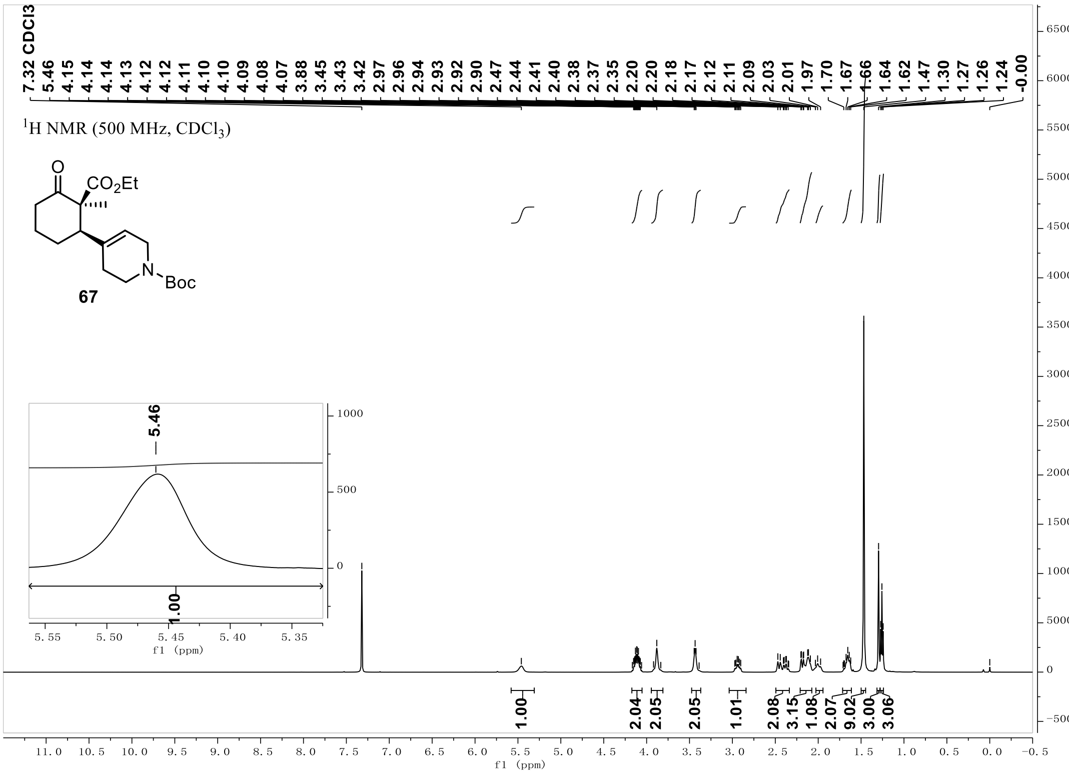

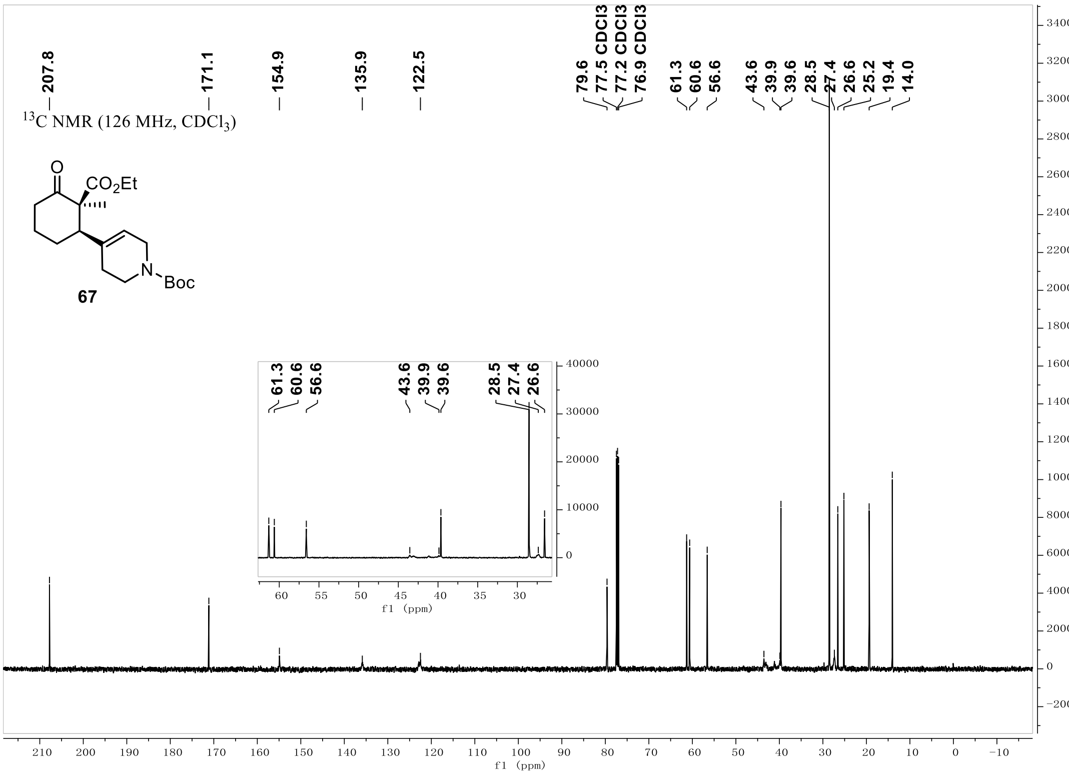

Supplementary Figure 64. ^{1}H NMR and ^{13}C NMR spectra of compound 67.

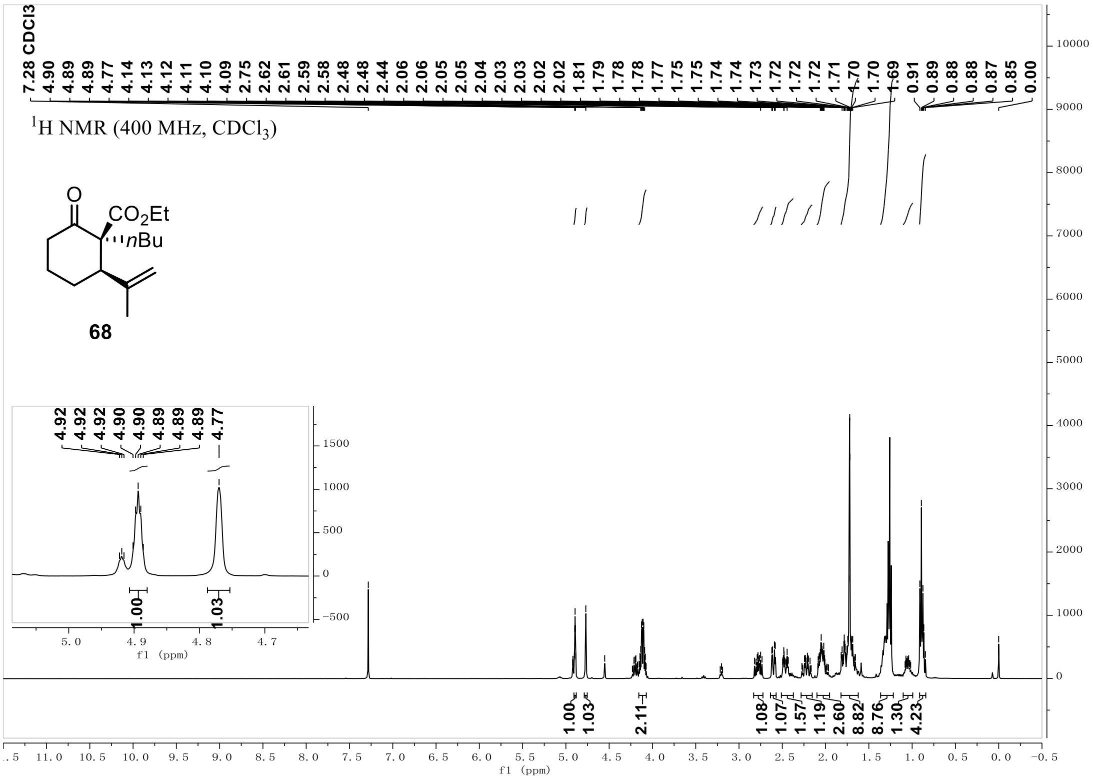

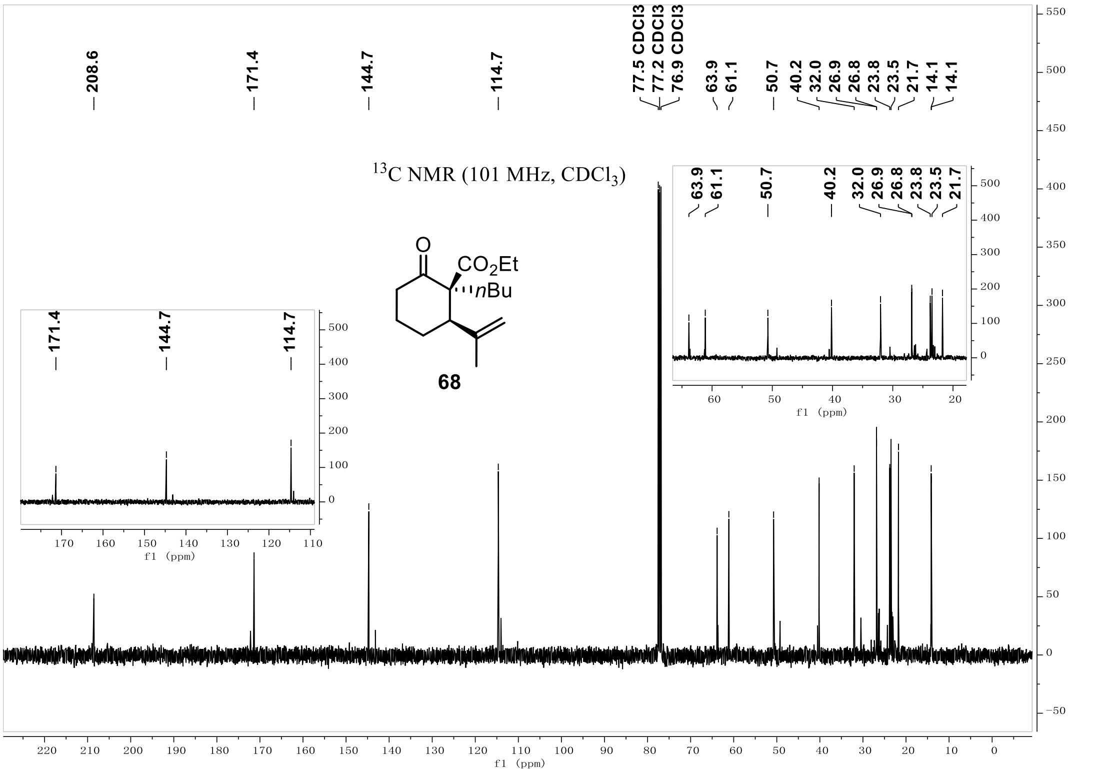

Supplementary Figure 65. ^{1}H NMR and ^{13}C NMR spectra of compound 68. The product was isolated as a 6:1 mixture of diastereomers.

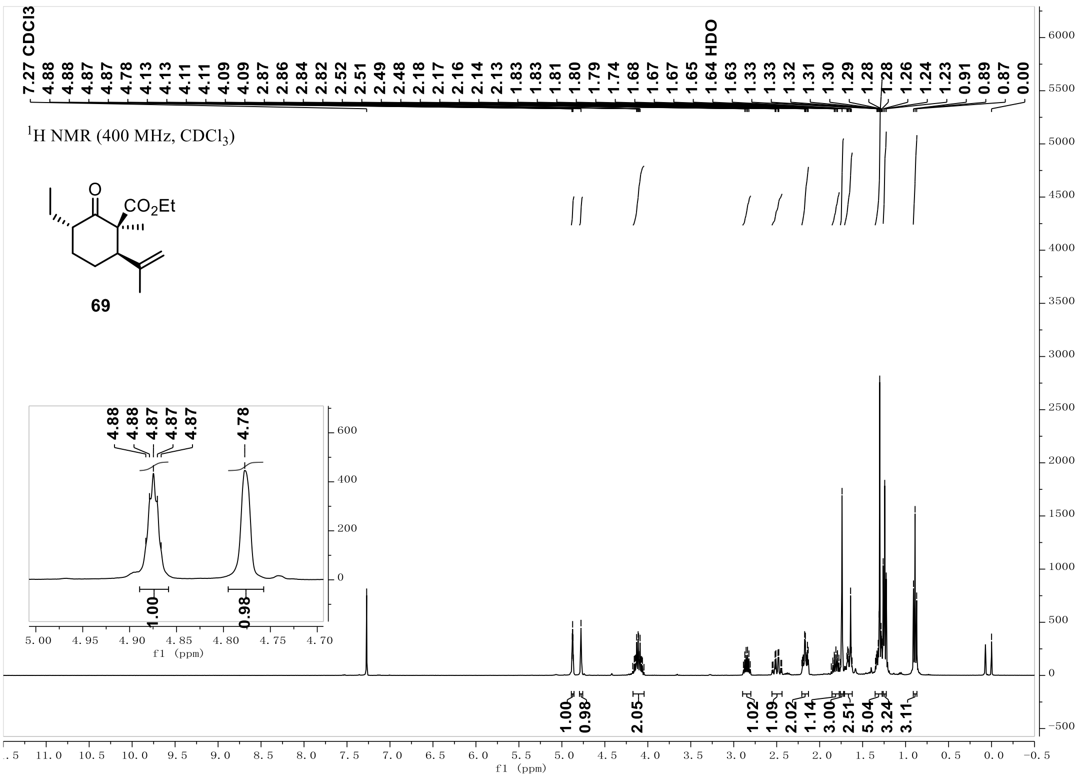

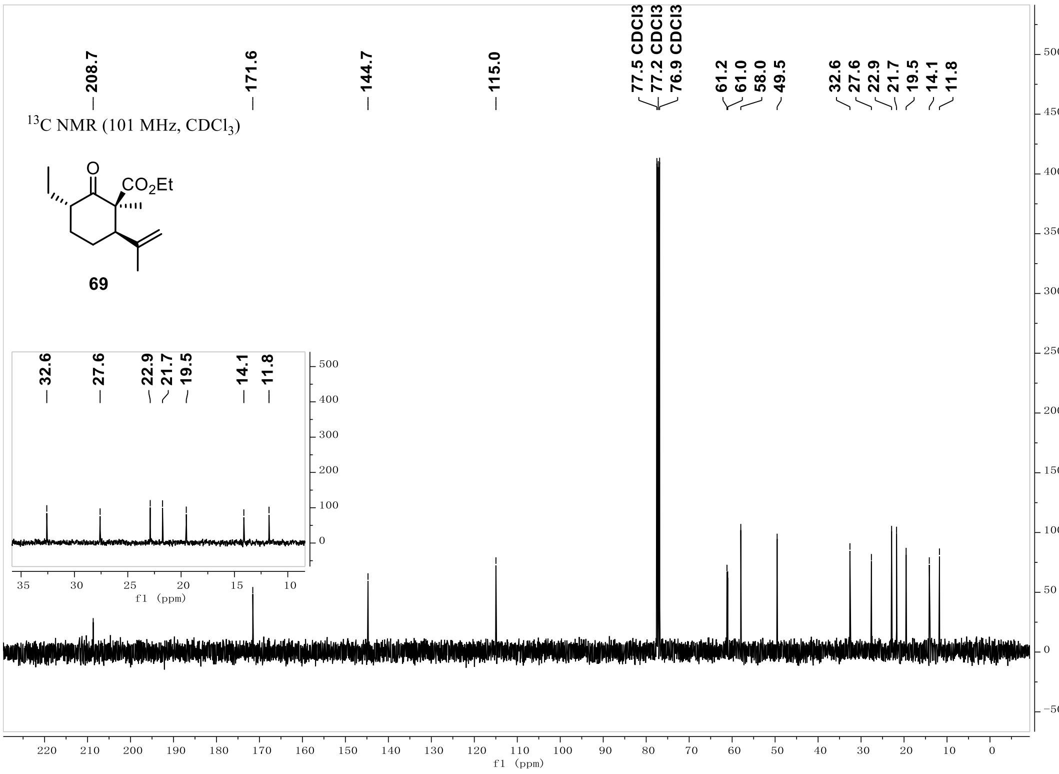

Supplementary Figure 66. ^{1}H NMR and ^{13}C NMR spectra of compound 69.

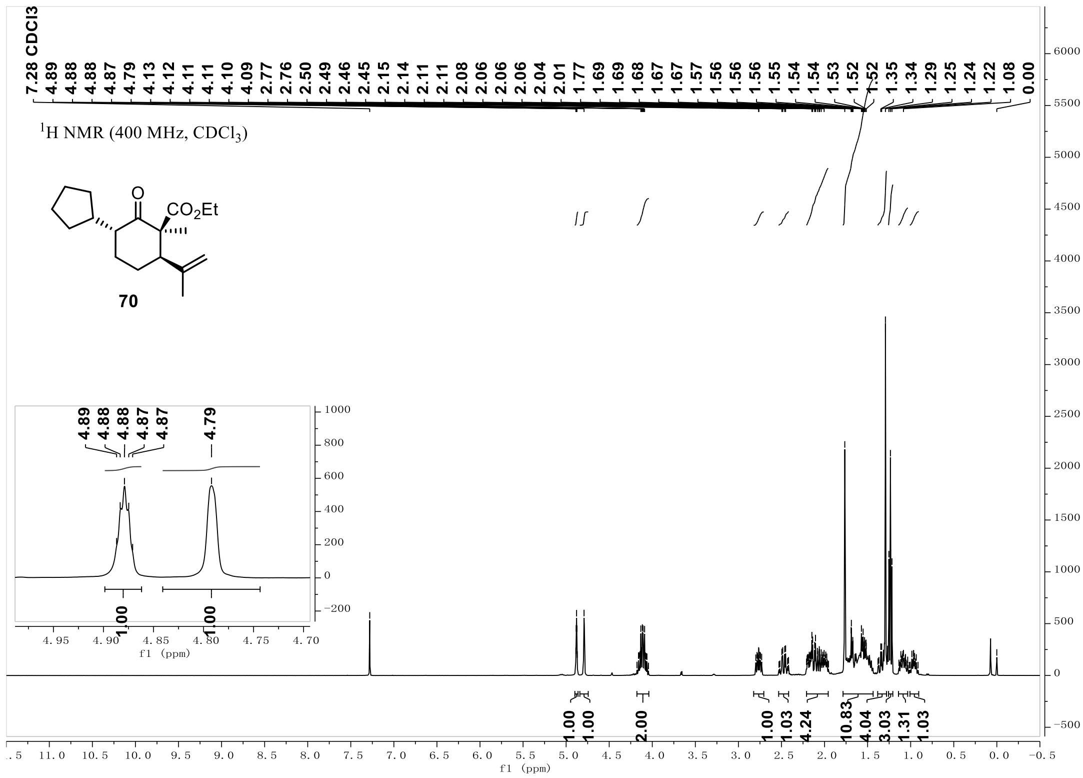

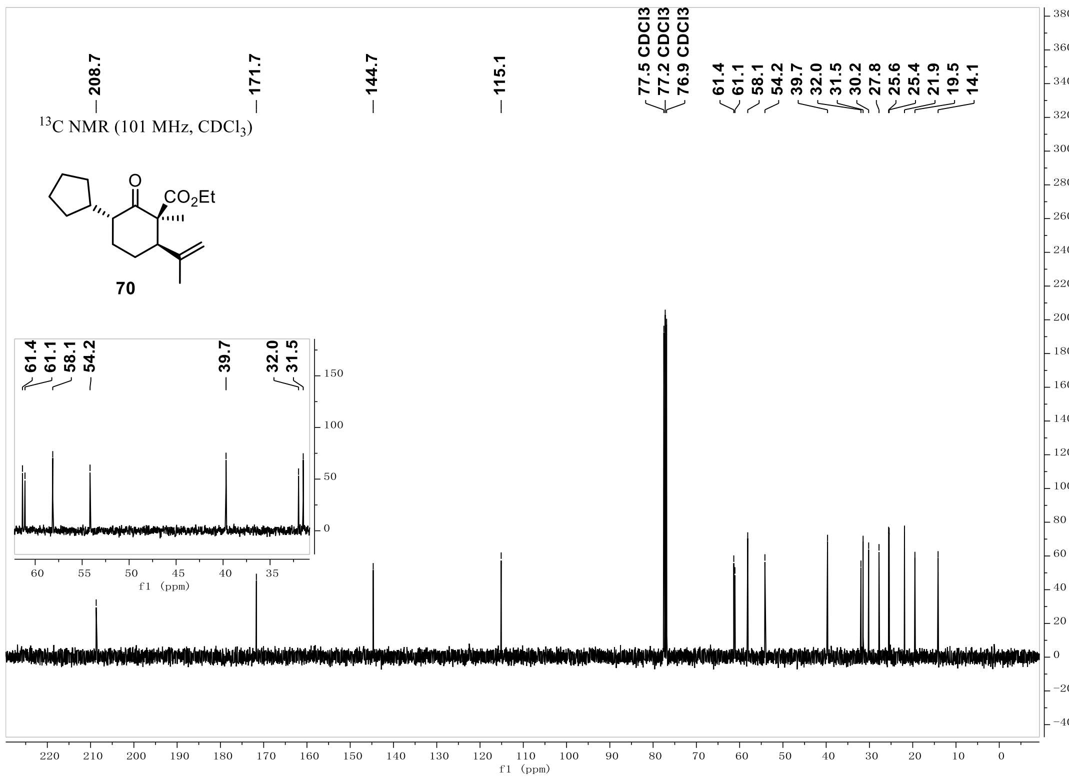

Supplementary Figure 67. ^{1}H NMR and ^{13}C NMR spectra of compound 70.

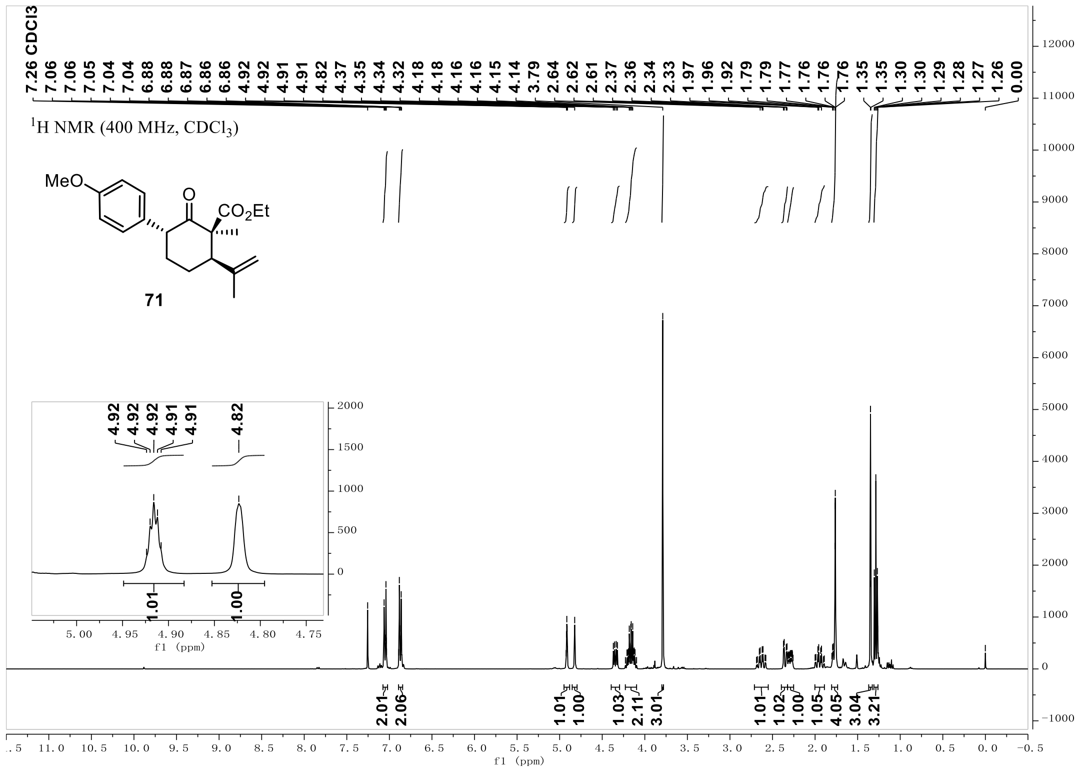

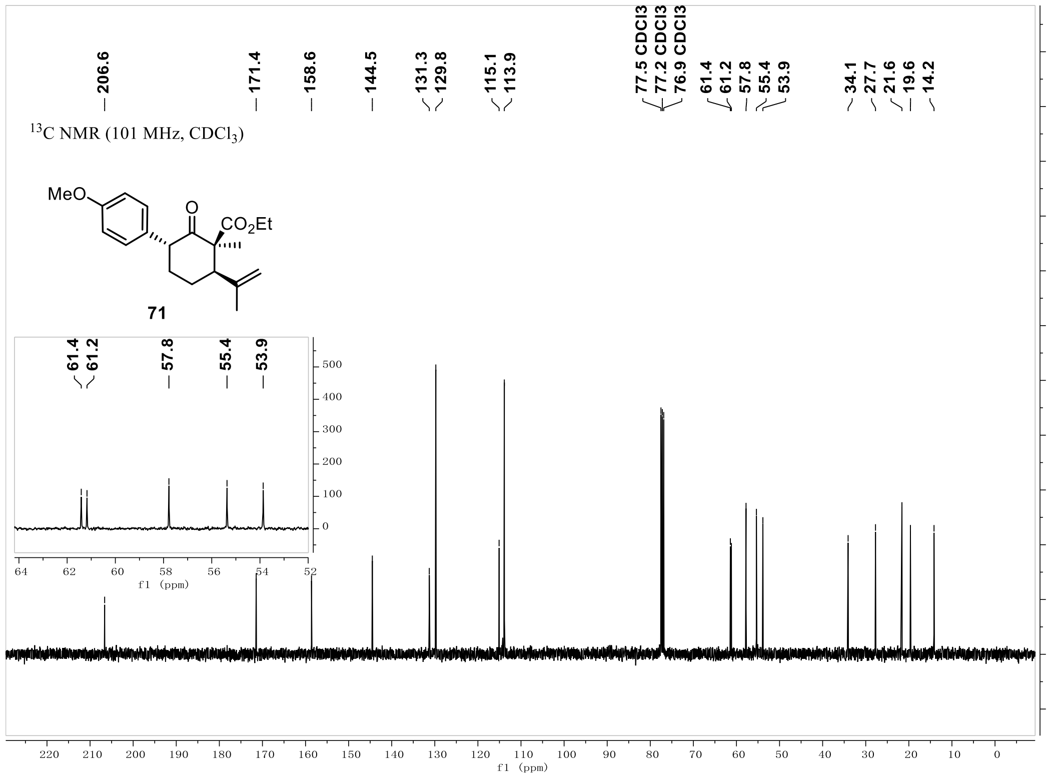

Supplementary Figure 68. ^{1}H NMR and ^{13}C NMR spectra of compound 71.

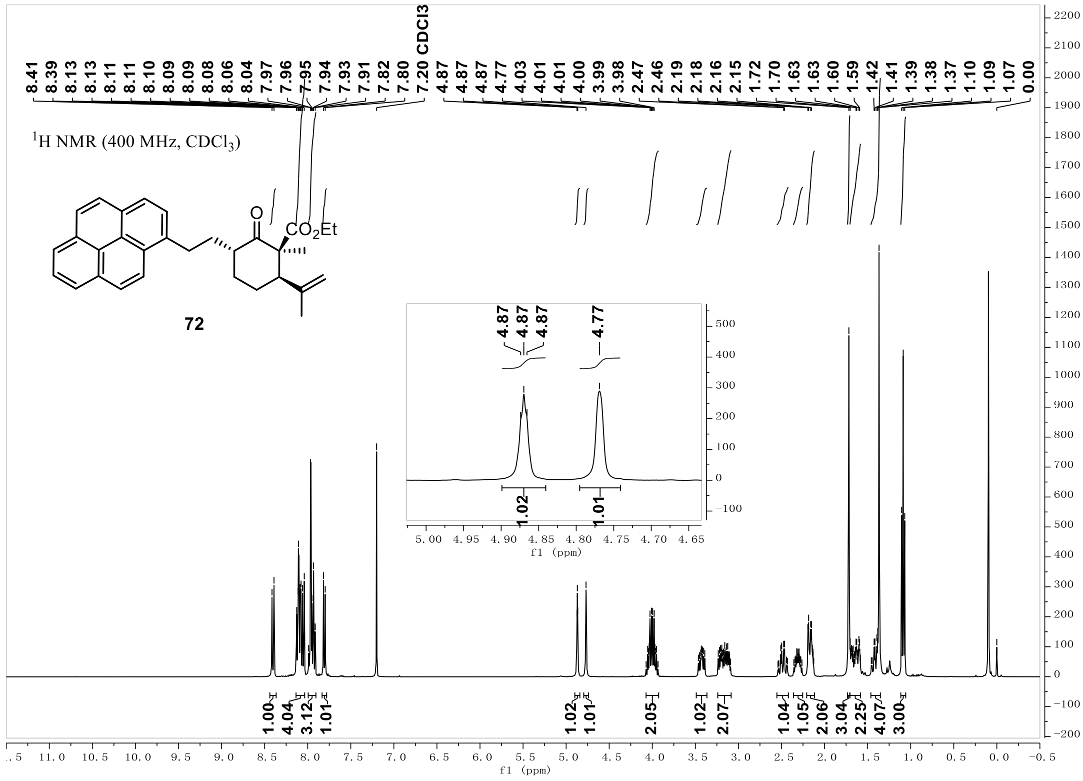

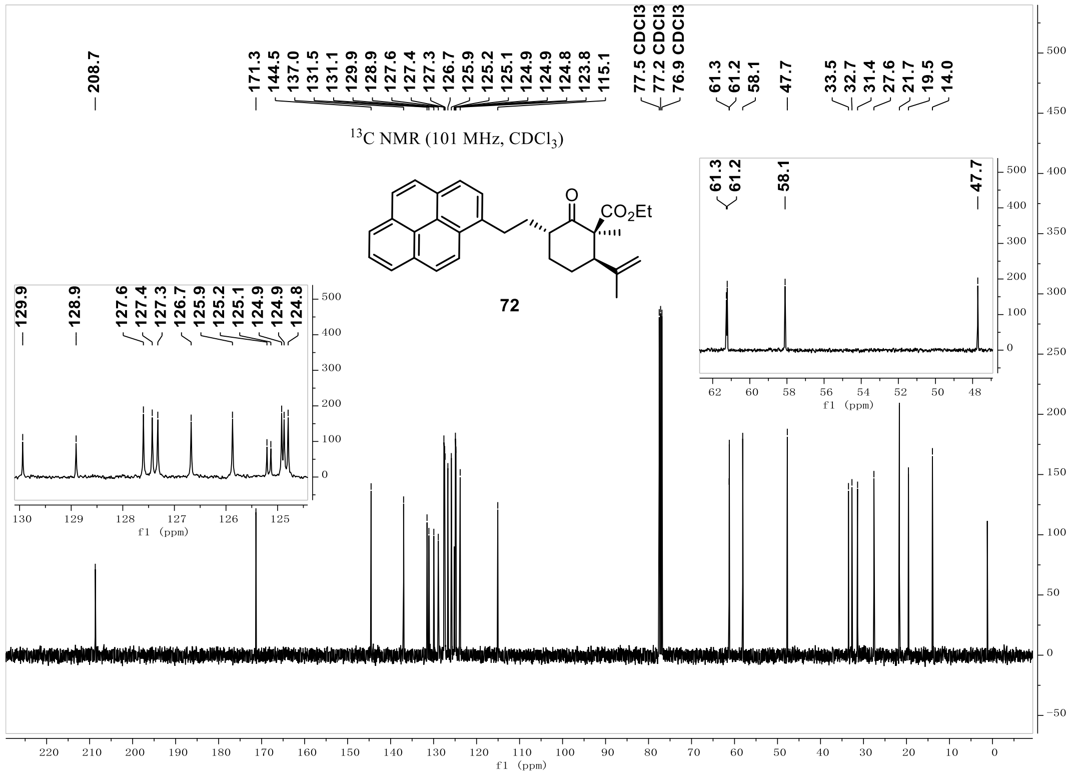

Supplementary Figure 69. ^{1}H NMR and ^{13}C NMR spectra of compound 72.

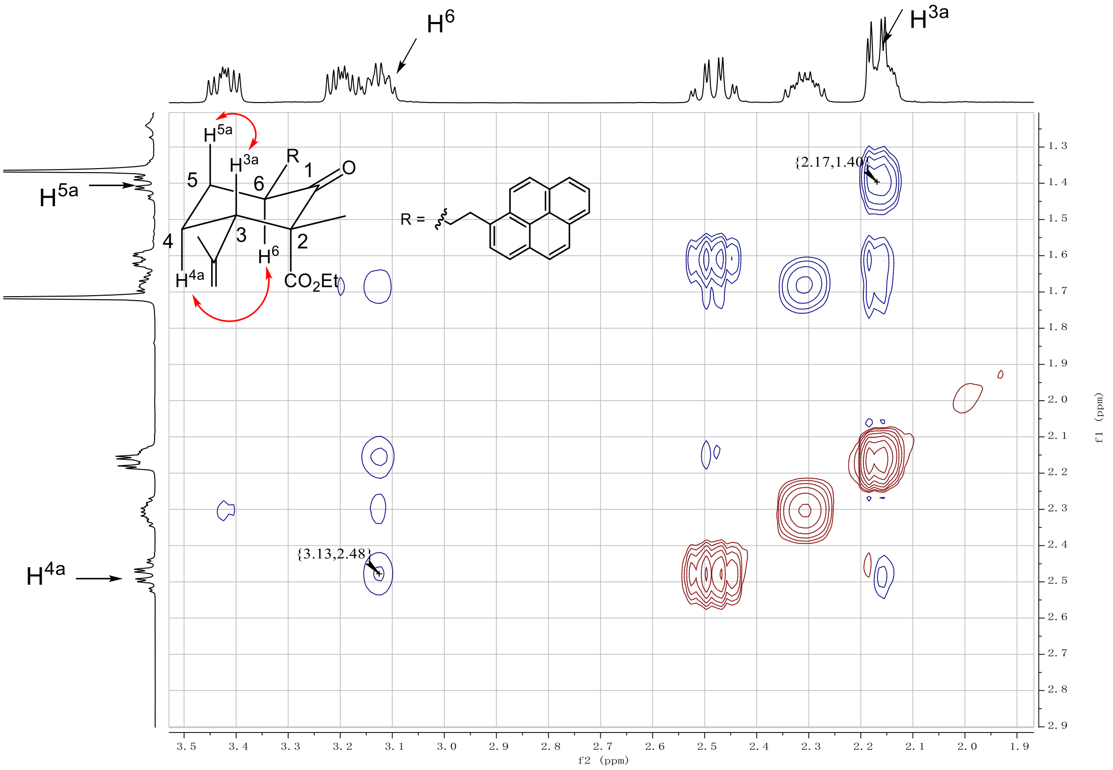

Supplementary Figure 70. 2D-NOESY spectra of compound 72.

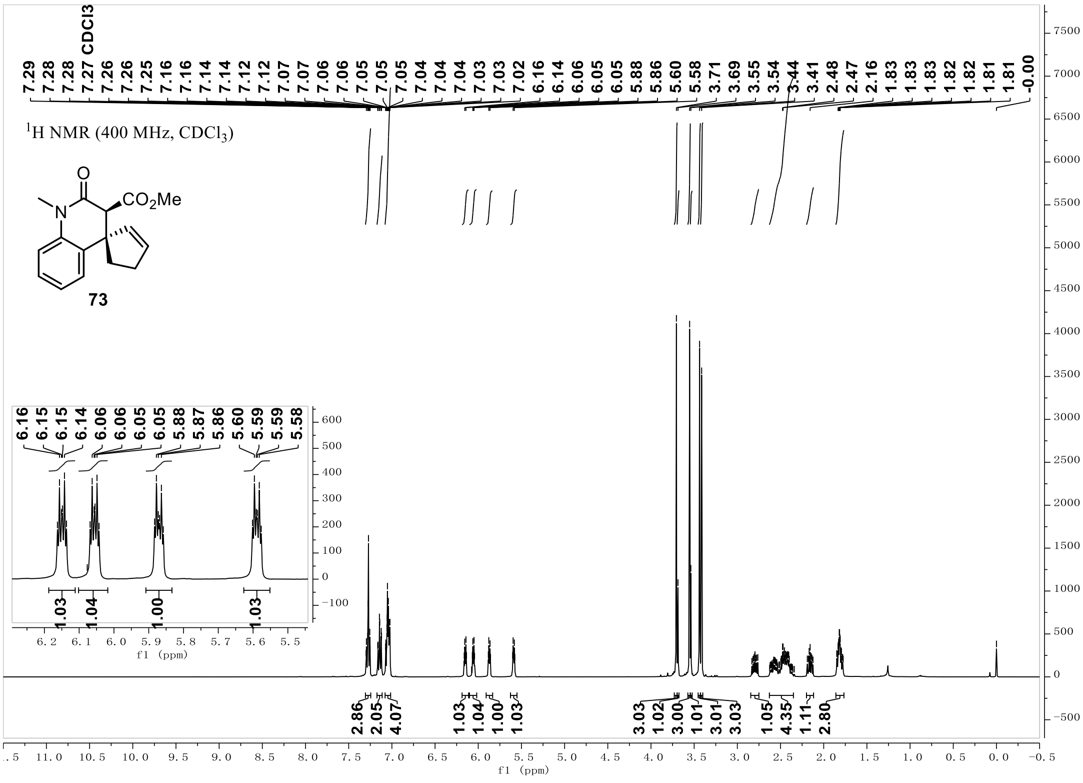

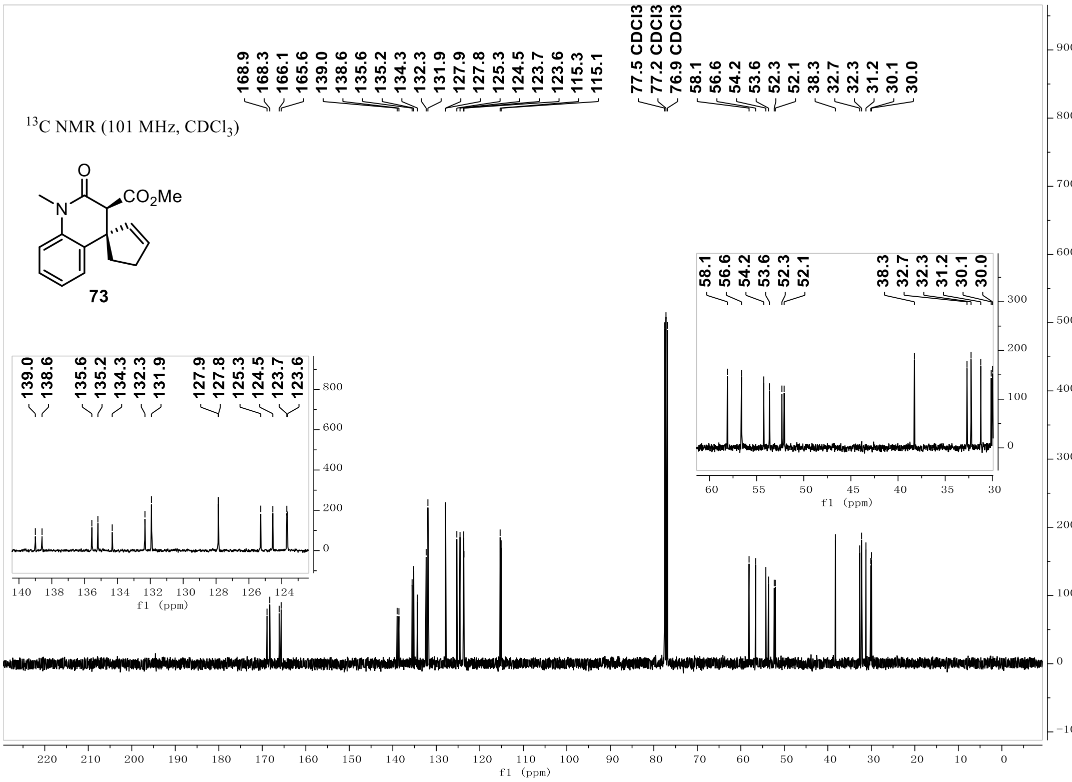

Supplementary Figure 71. ^{1}H NMR and ^{13}C NMR spectra of compound 73. The product was isolated as a 1:1 mixture of diastereomers.

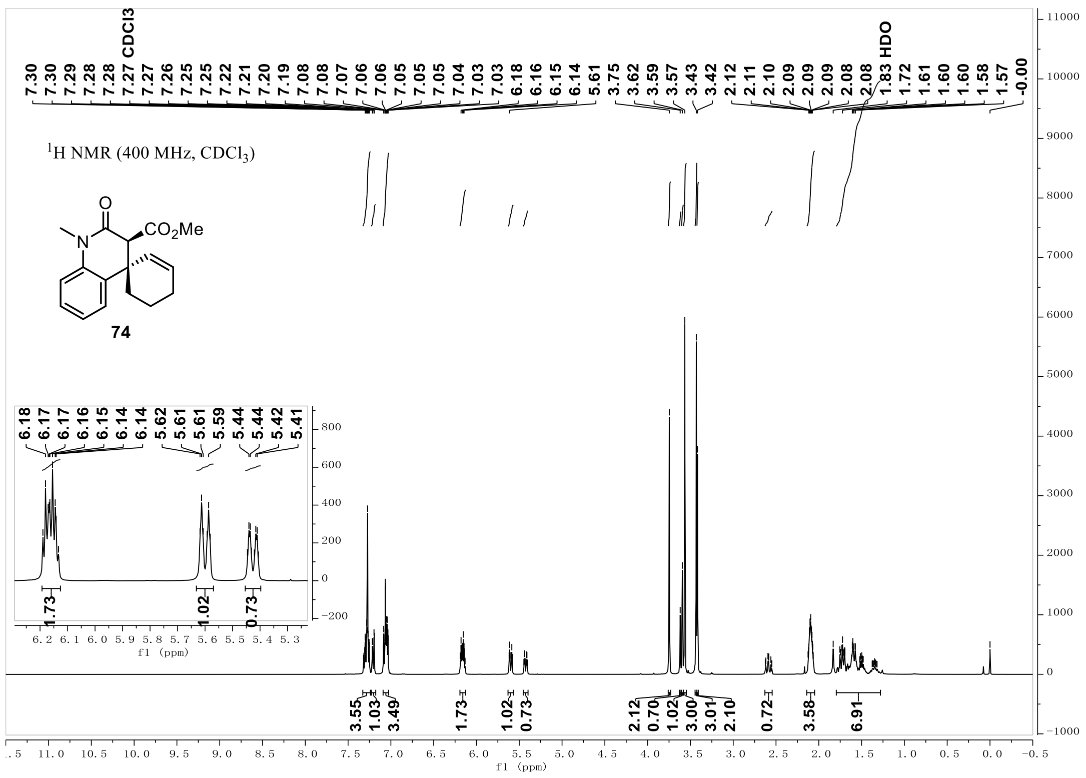

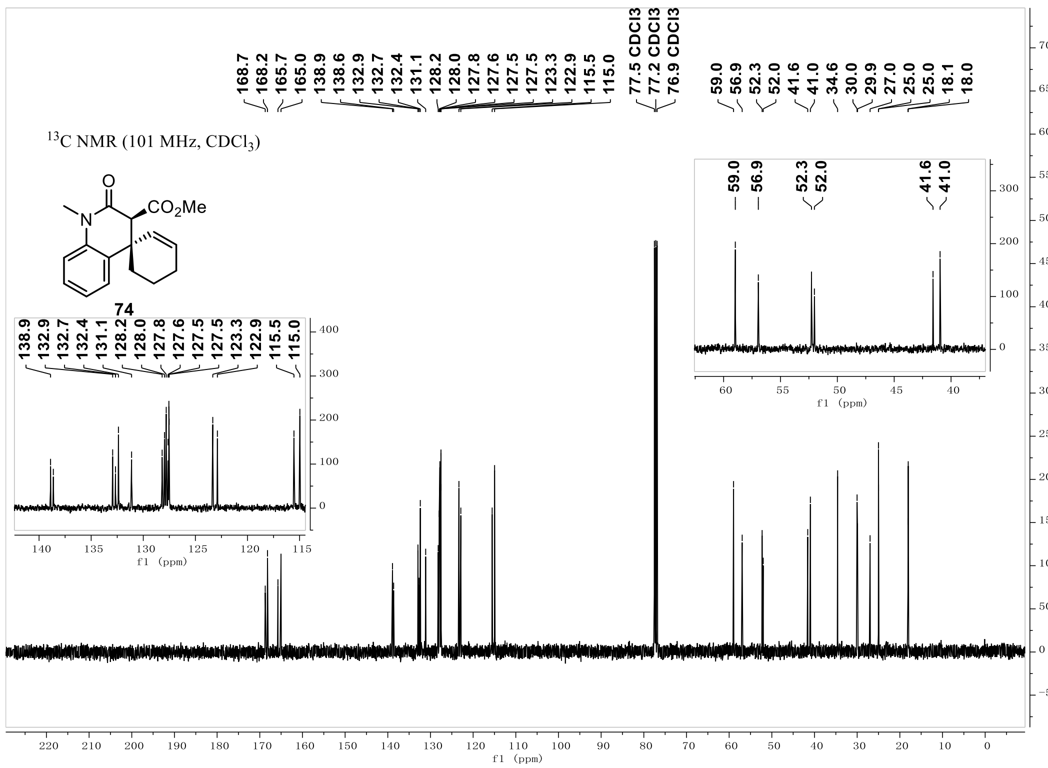

Supplementary Figure 72. ^{1}H NMR and ^{13}C NMR spectra of compound 74. The product was isolated as a 1:0.7 mixture of diastereomers.

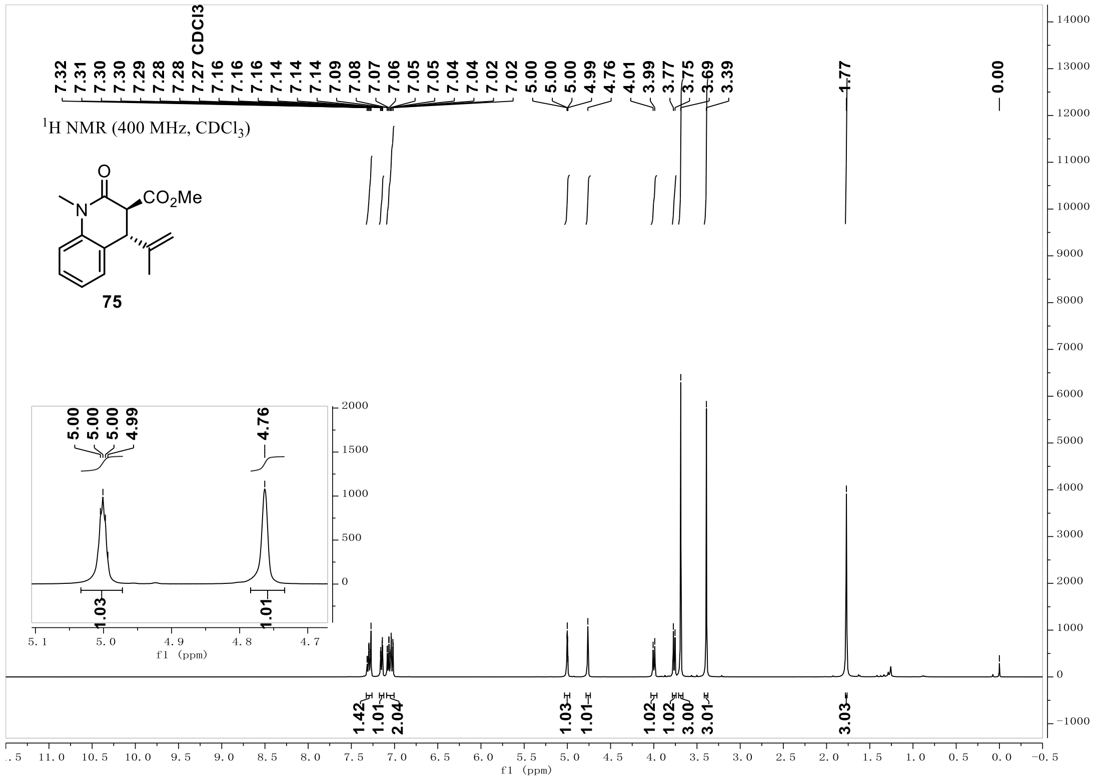

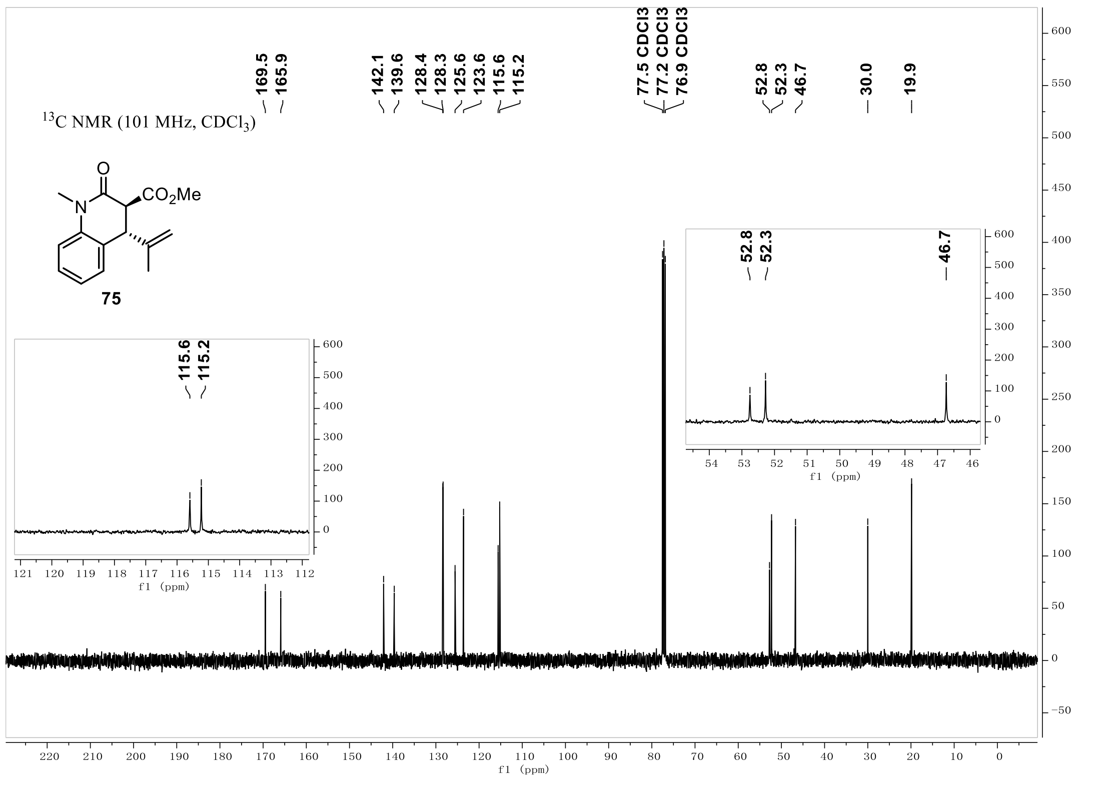

Supplementary Figure 73. ^{1}H NMR and ^{13}C NMR spectra of compound 75.

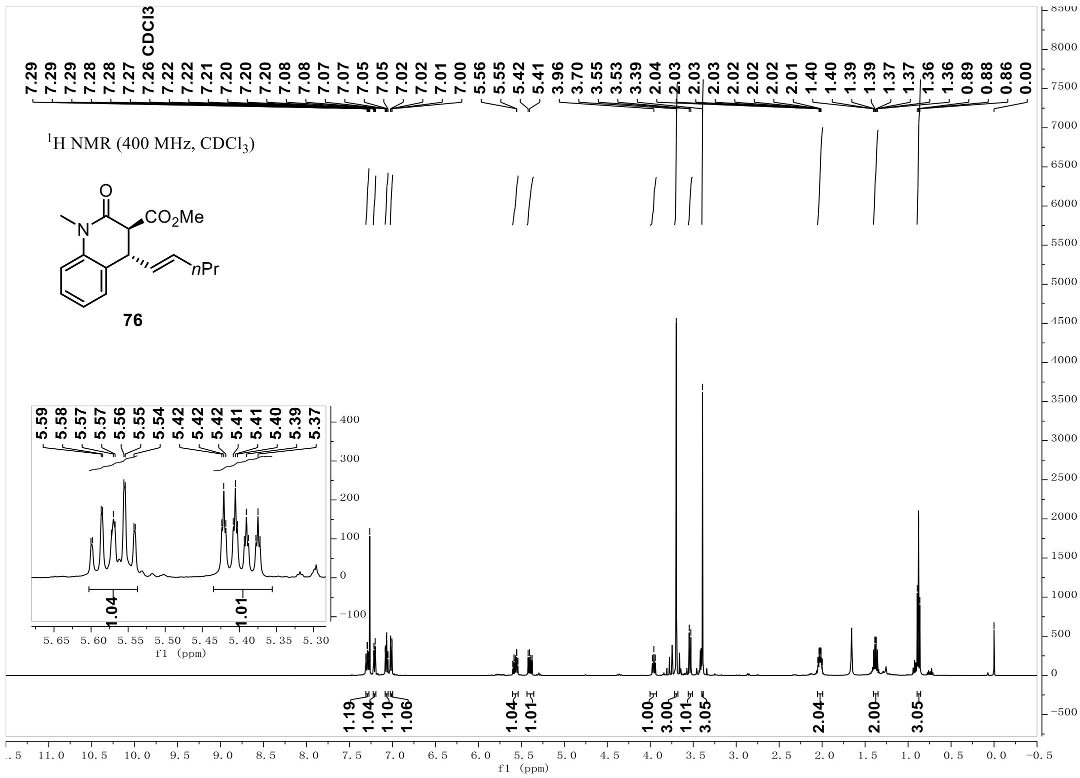

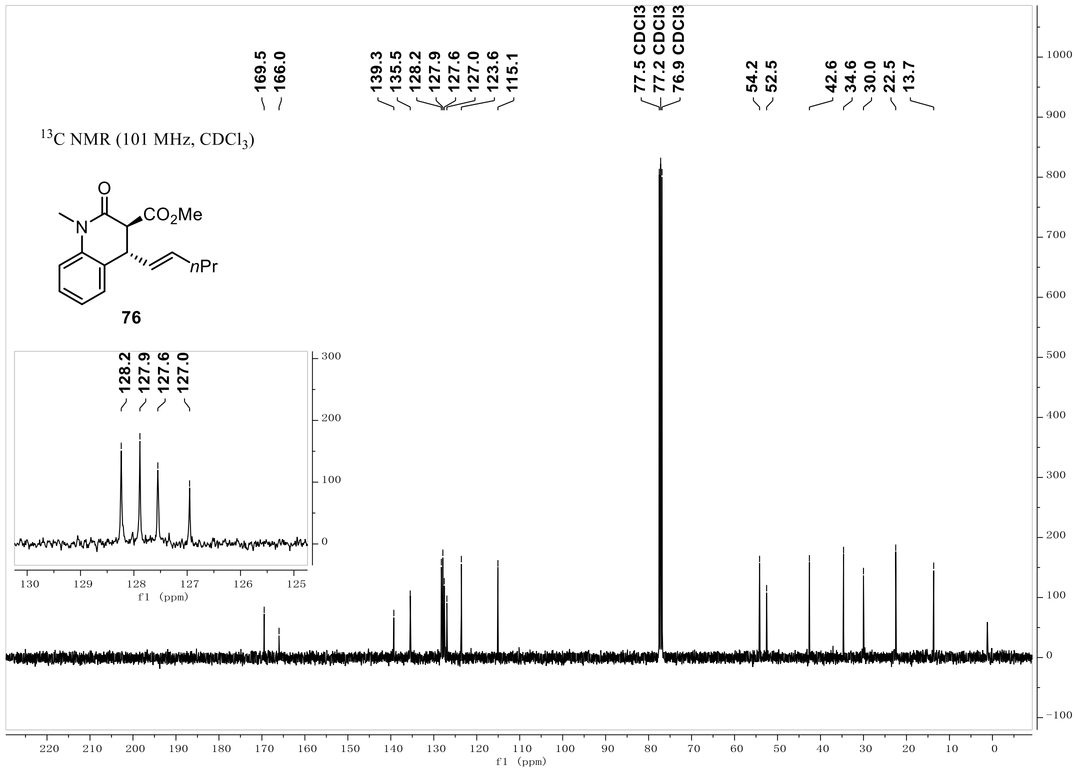

Supplementary Figure 74. ^{1}H NMR and ^{13}C NMR spectra of compound 76.

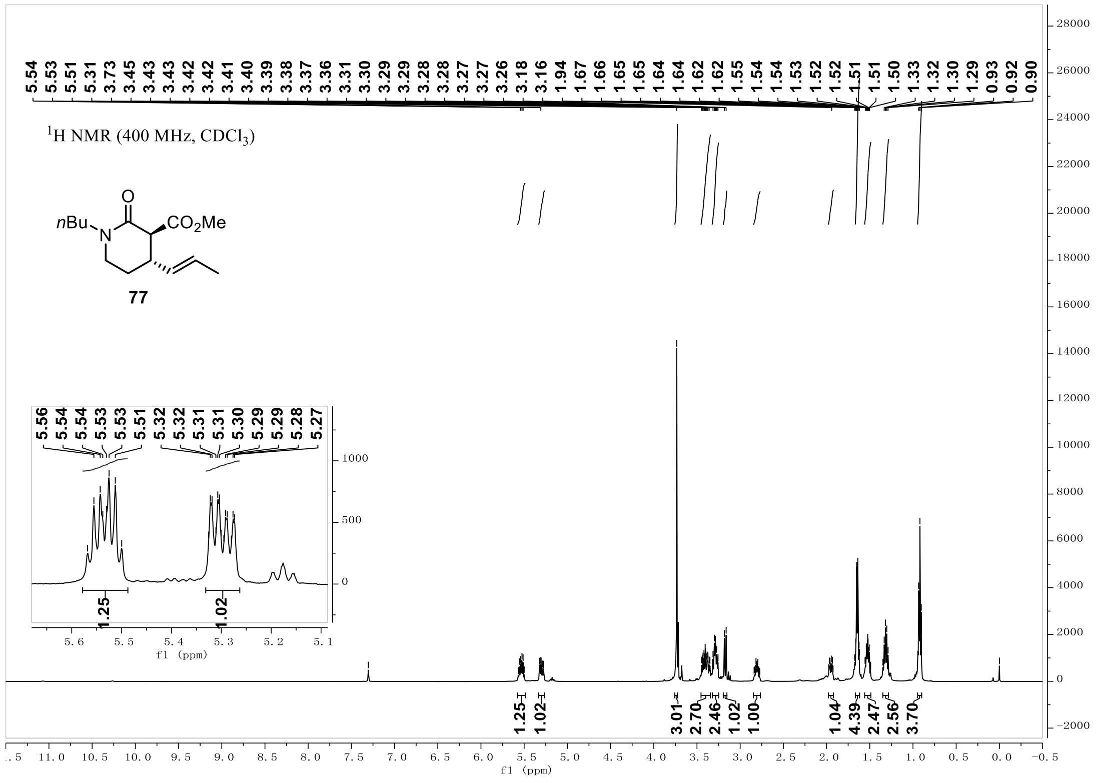

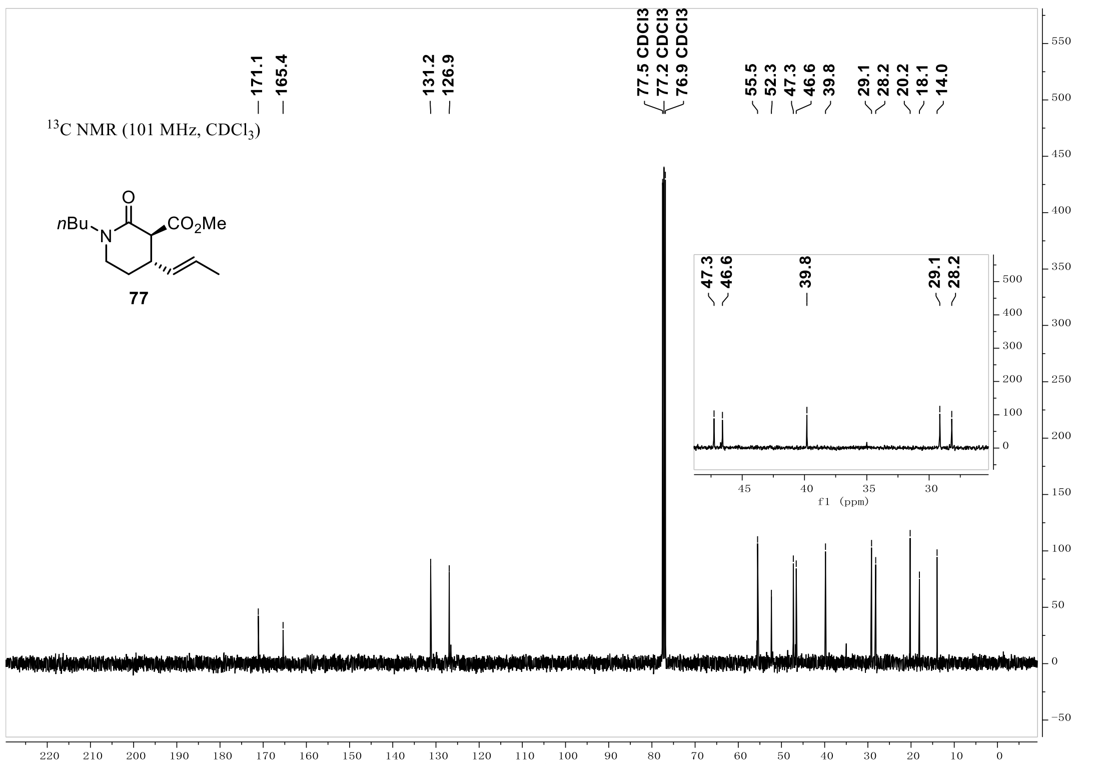

Supplementary Figure 75. ^{1}H NMR and ^{13}C NMR spectra of compound 77. The product was isolated as a 7:1 mixture of diastereomers.

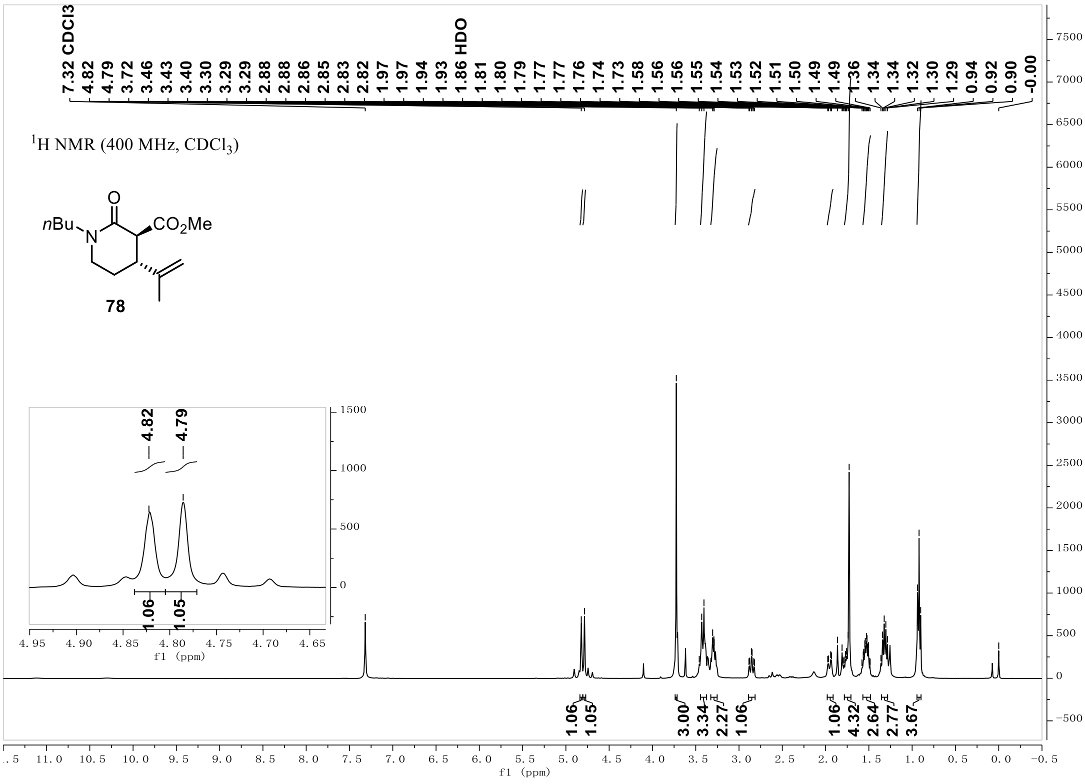

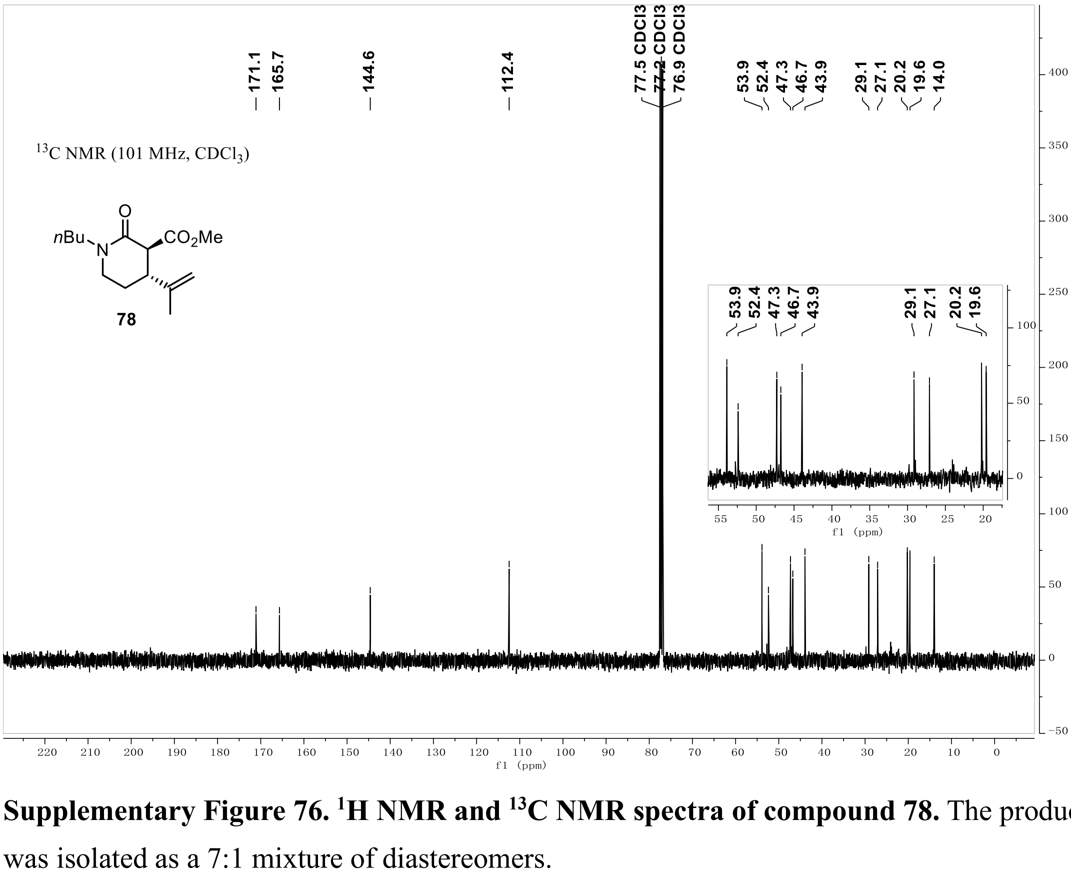

Supplementary Figure 76. ^{1}H NMR and ^{13}C NMR spectra of compound 78. The product was isolated as a 7:1 mixture of diastereomers.

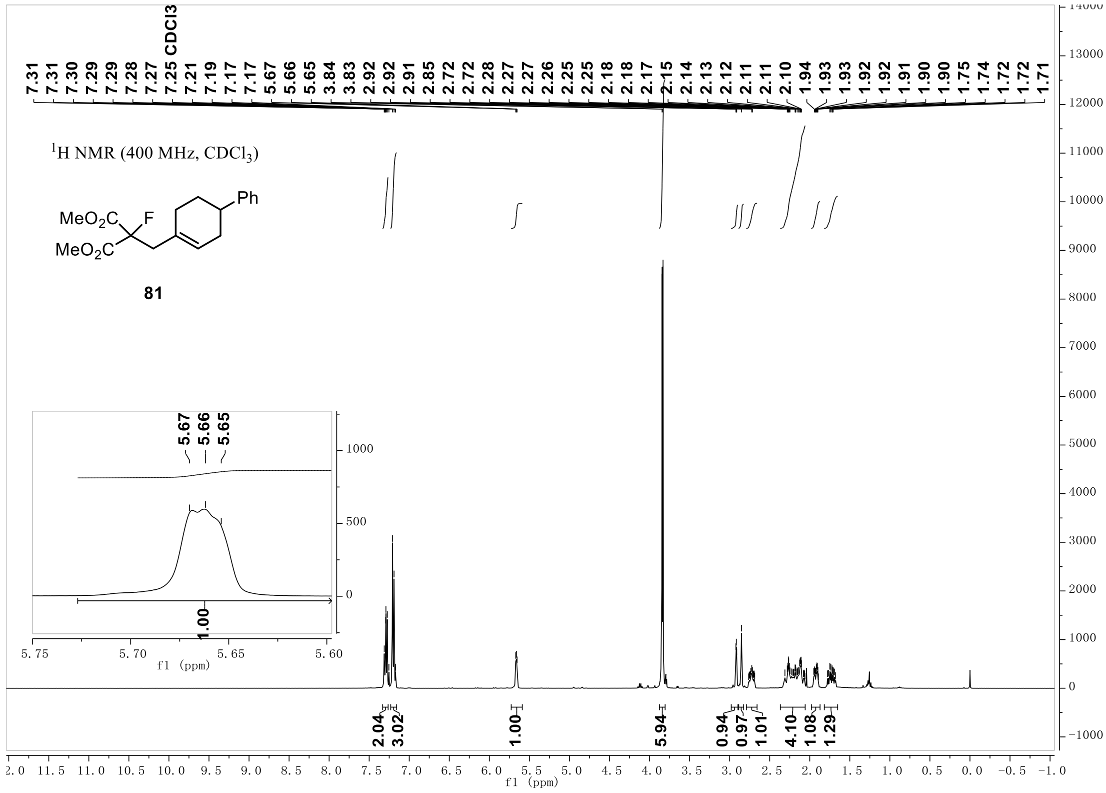

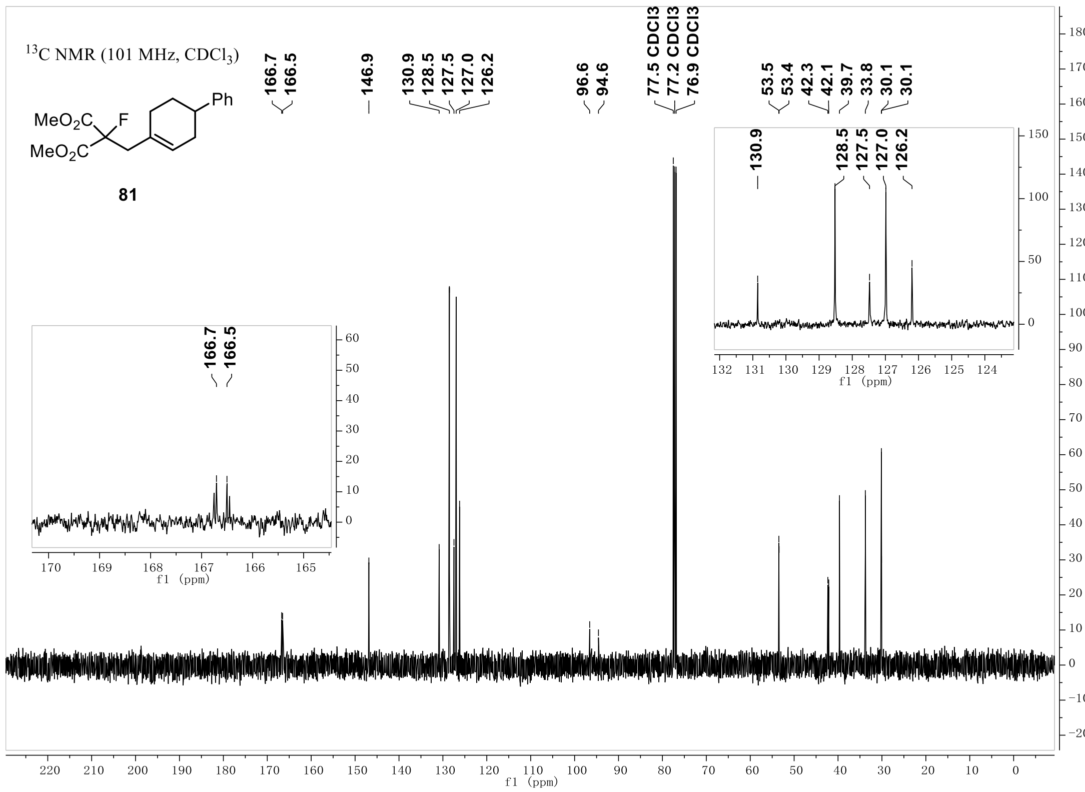

Supplementary Figure 77. ^{1}H NMR and ^{13}C NMR spectra of compound 81.

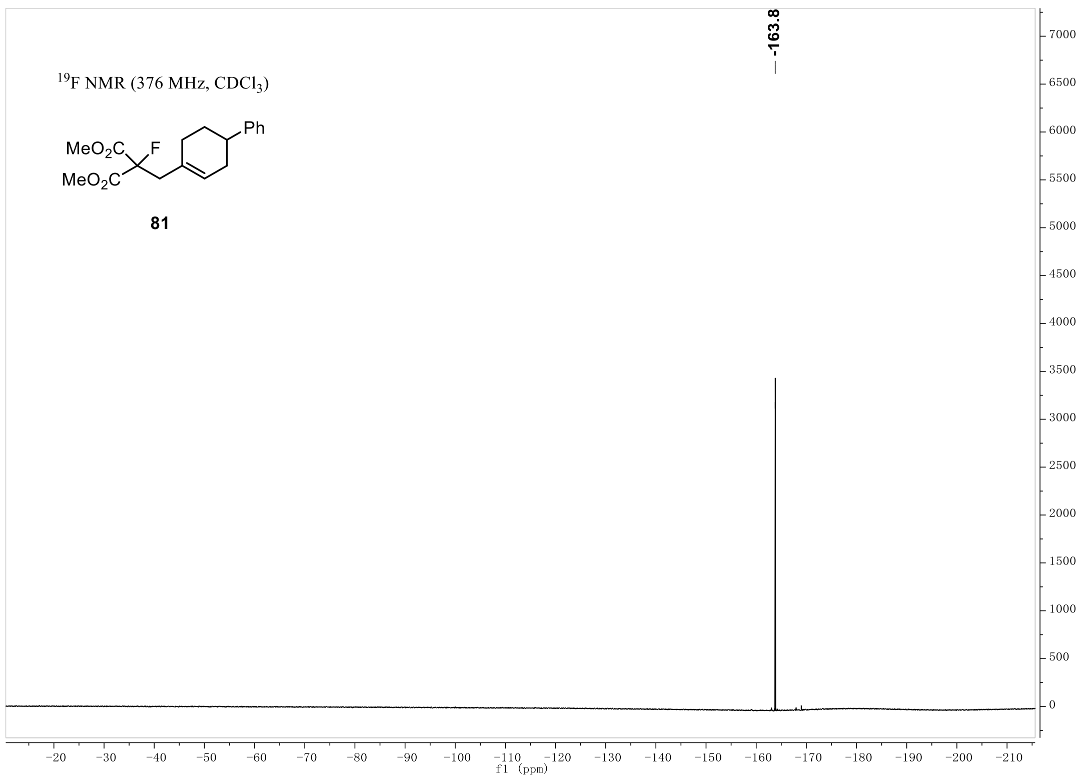

Supplementary Figure 78. ^{19}F NMR spectra of compound 81.

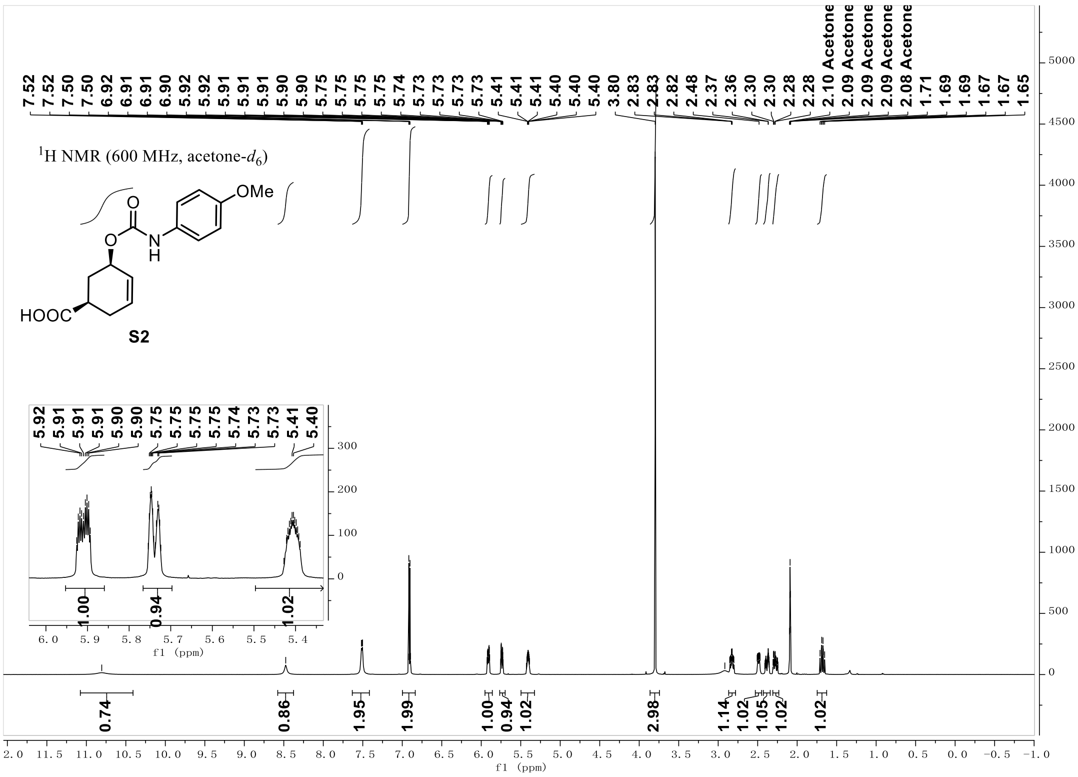

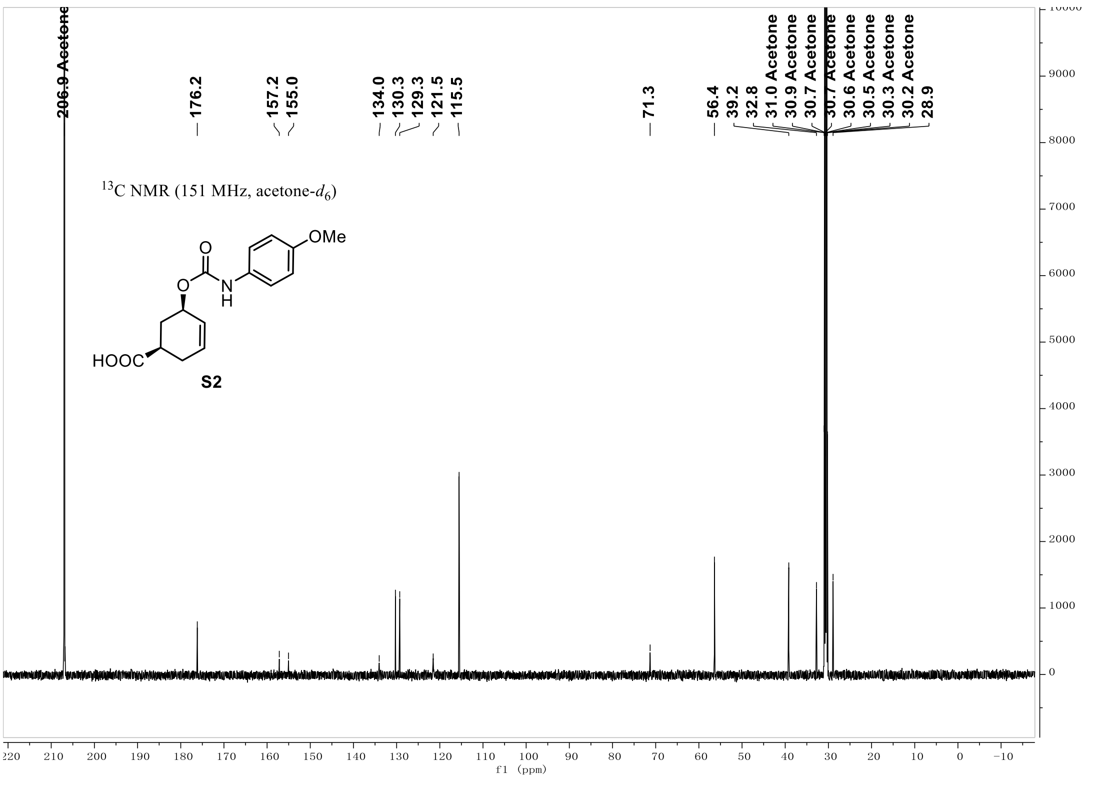

Supplementary Figure 79. ^{1}H NMR and ^{13}C NMR spectra of compound S2.

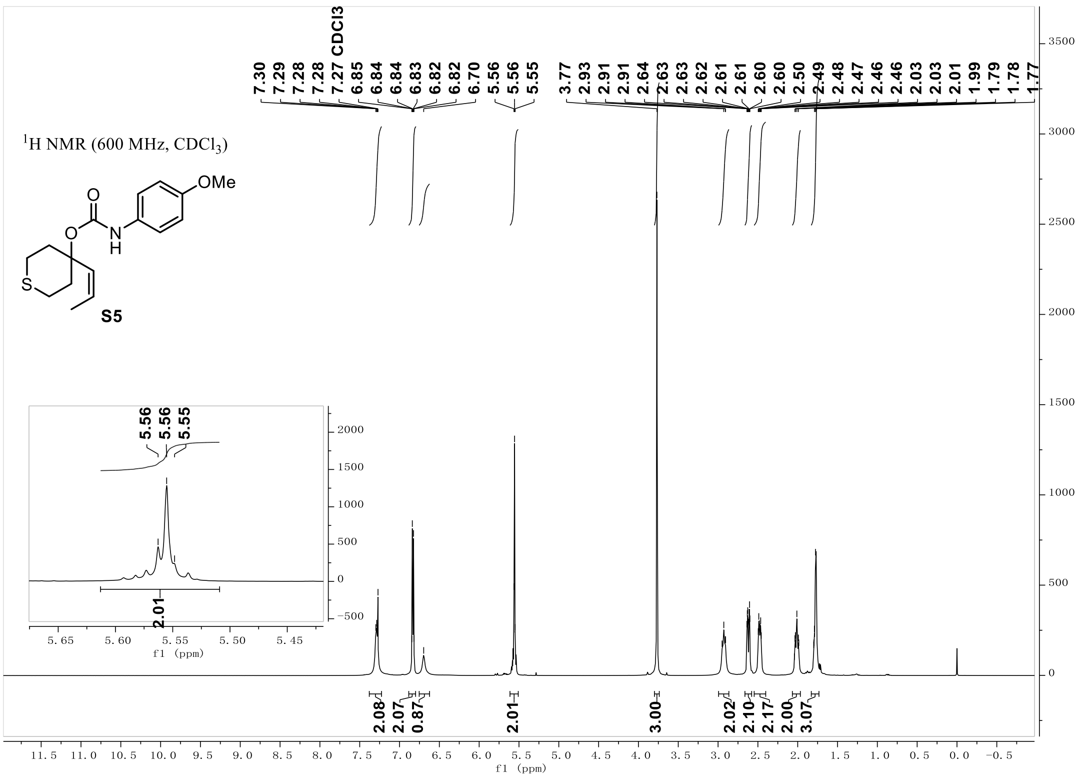

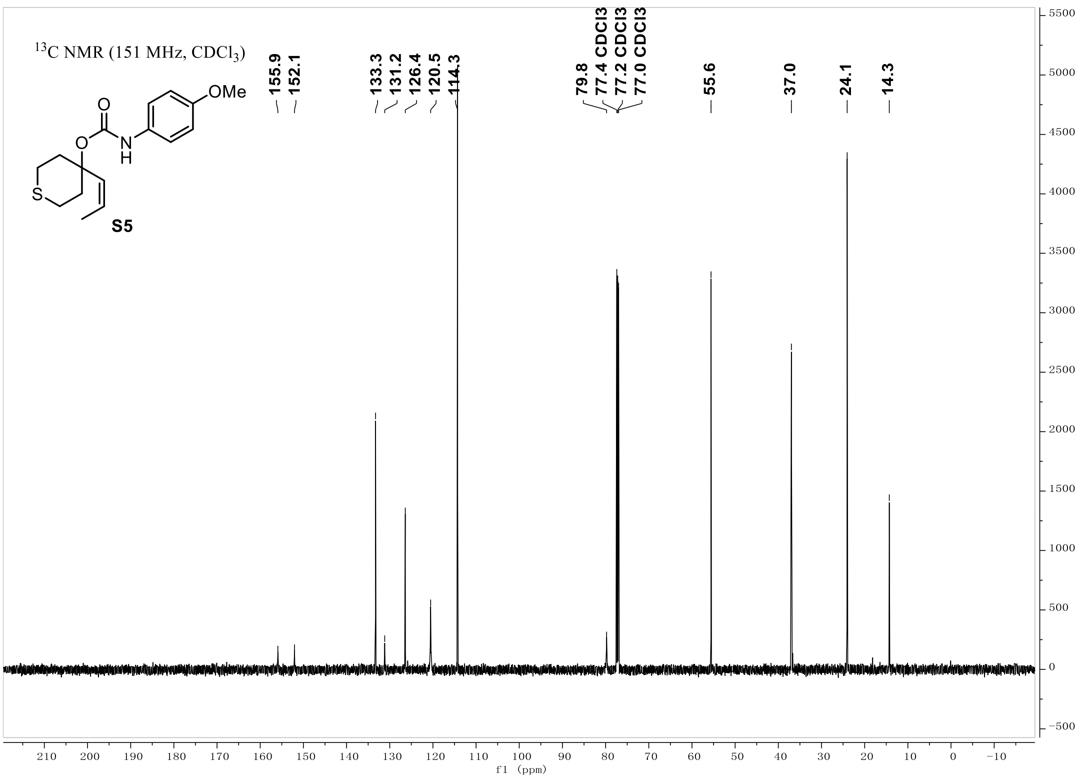

Supplementary Figure 80. ^{1}H NMR and ^{13}C NMR spectra of compound S5.

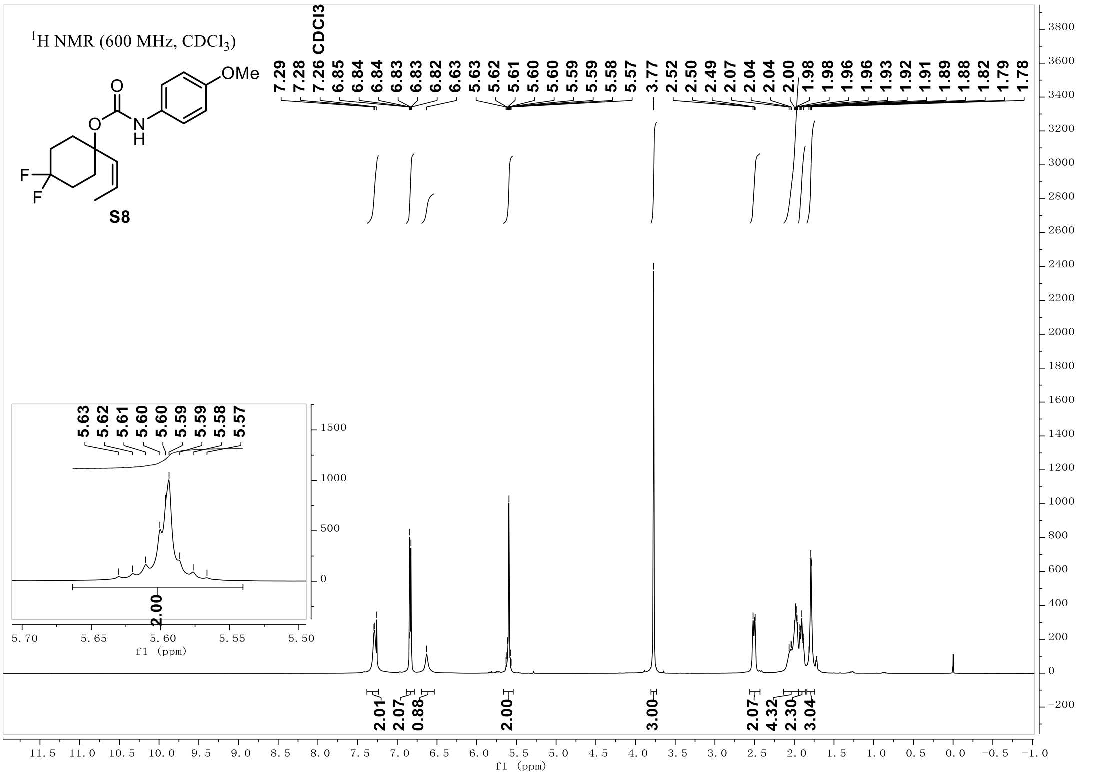

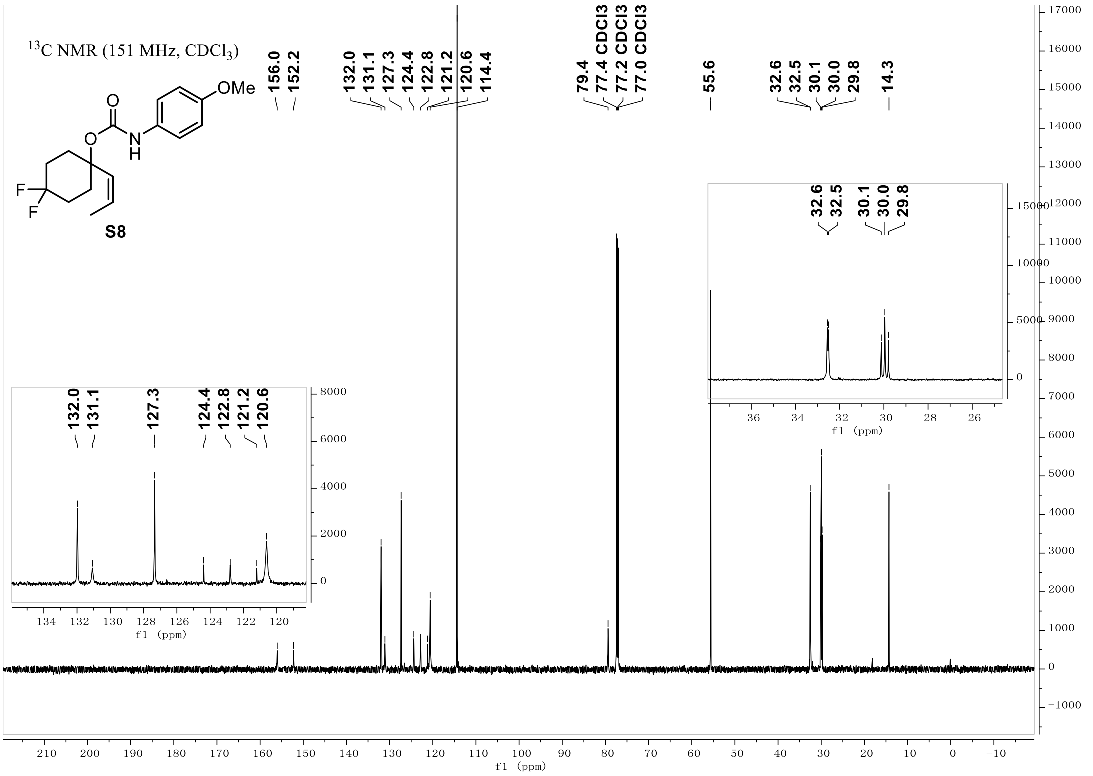

Supplementary Figure 81. ^{1}H NMR and ^{13}C NMR spectra of compound S8.

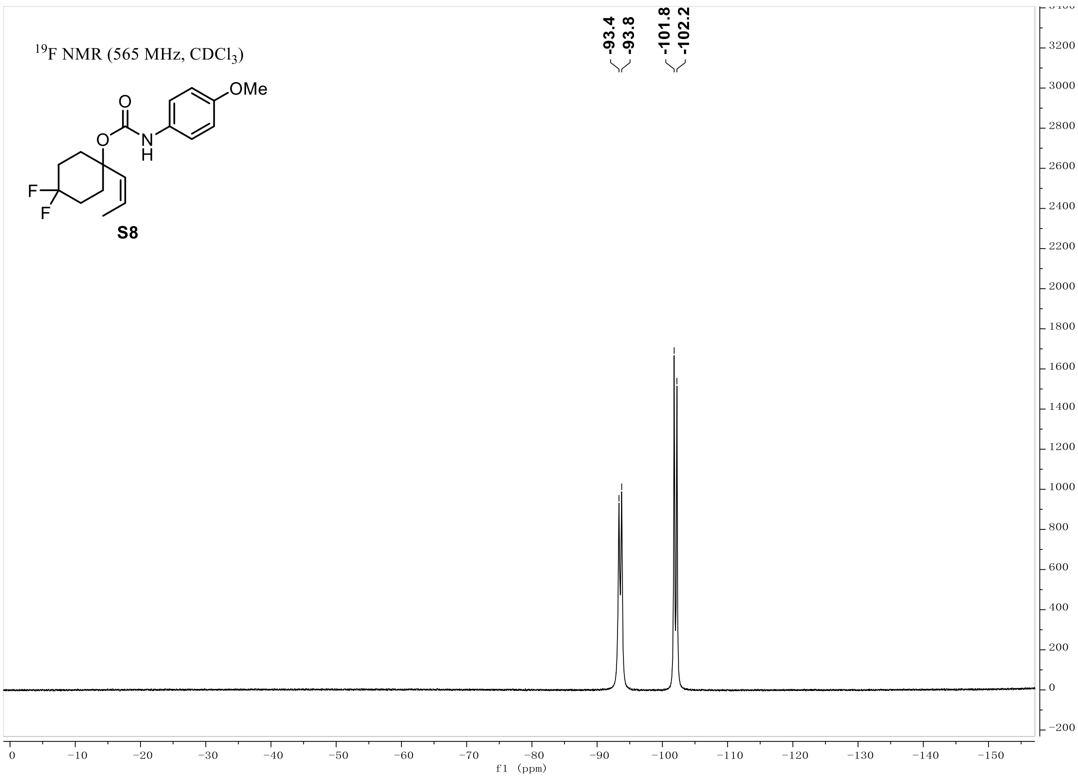

Supplementary Figure 82. ^{19}F NMR spectra of compound S8.

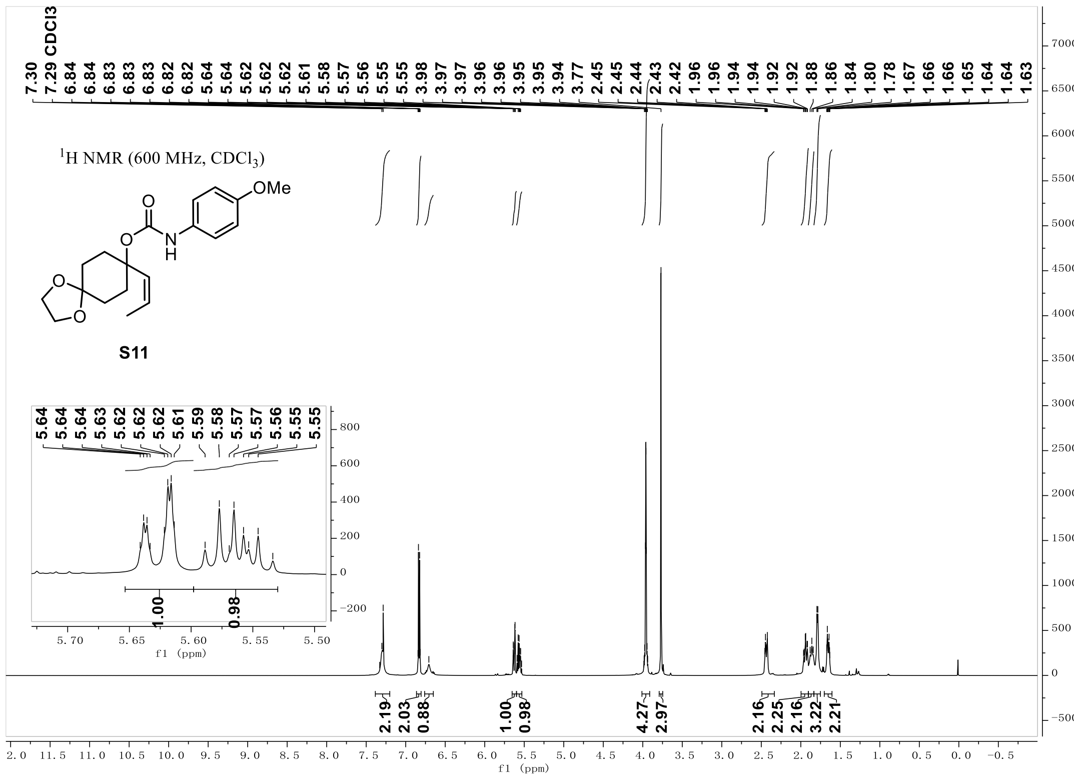

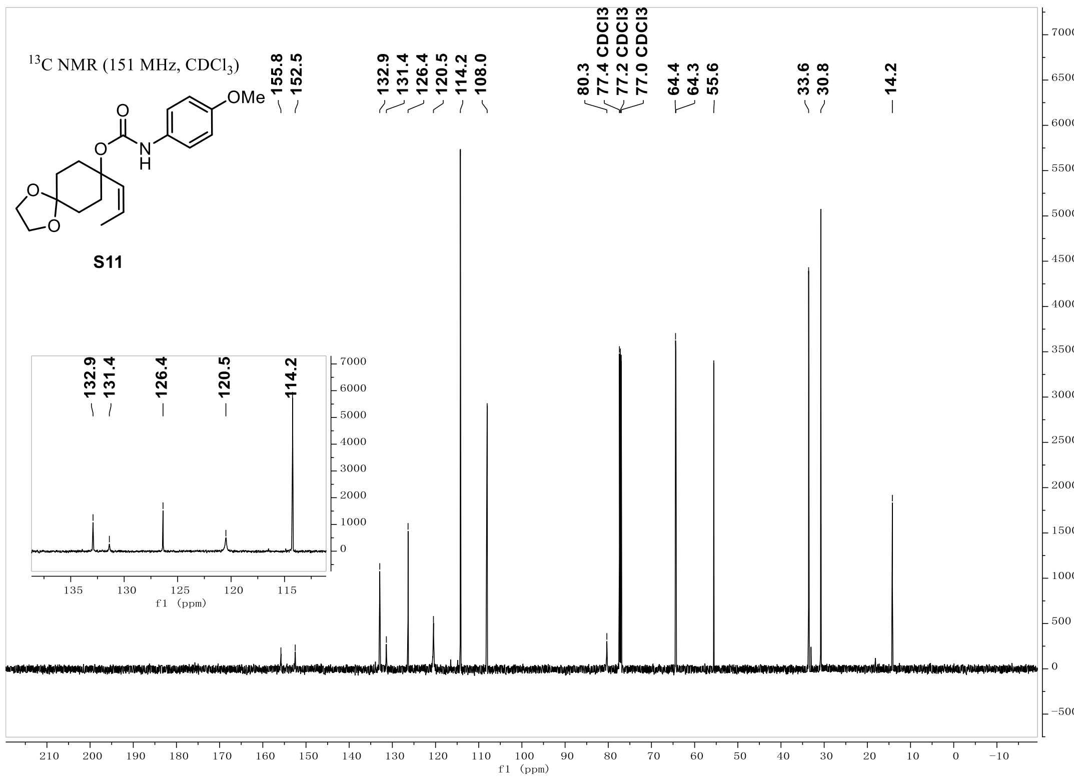

Supplementary Figure 83. ^{1}H NMR and ^{13}C NMR spectra of compound S11.

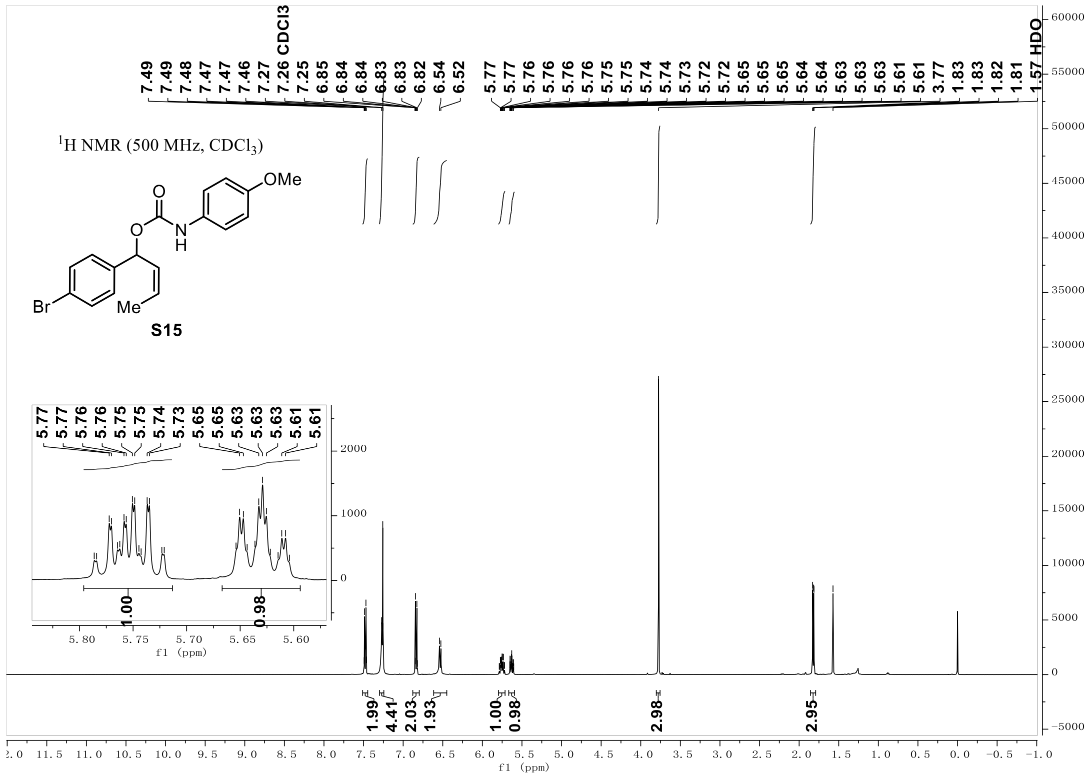

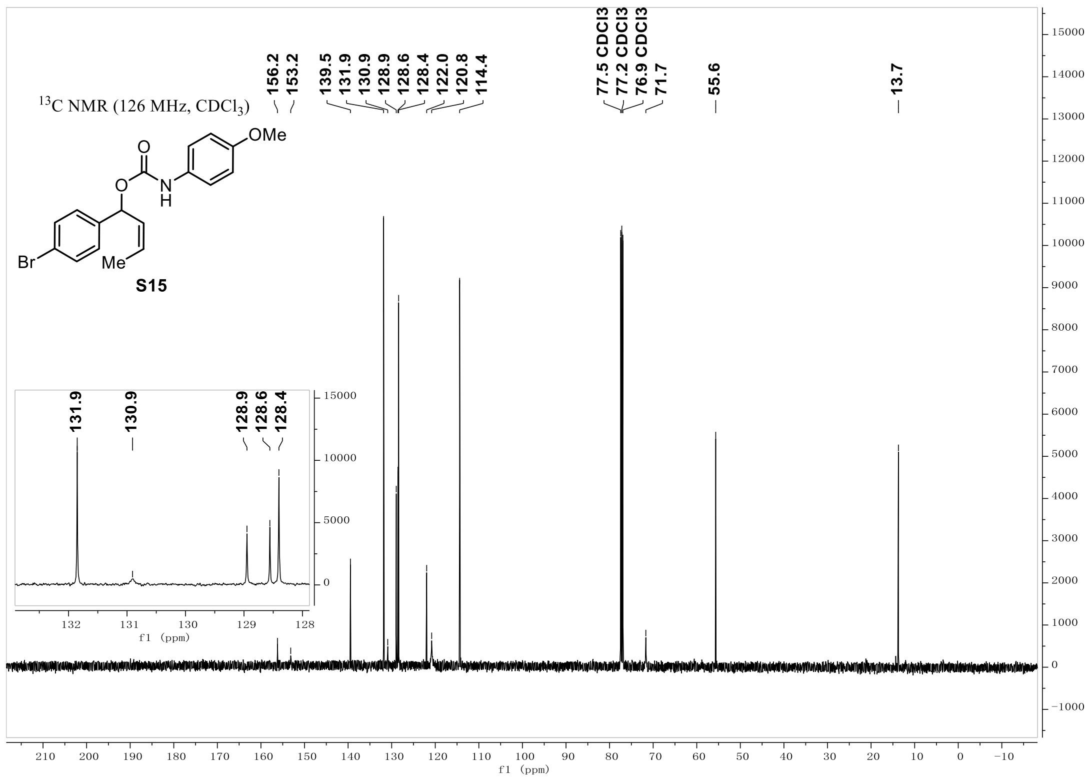

Supplementary Figure 84. ^{1}H NMR and ^{13}C NMR spectra of compound S15.

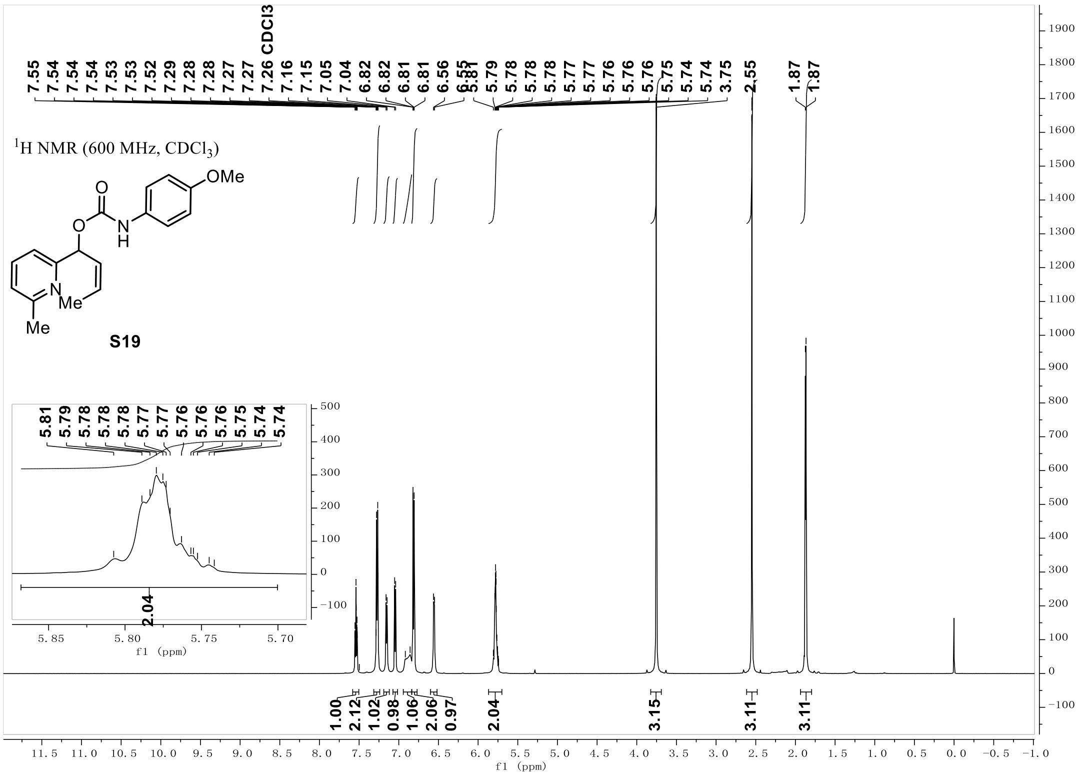

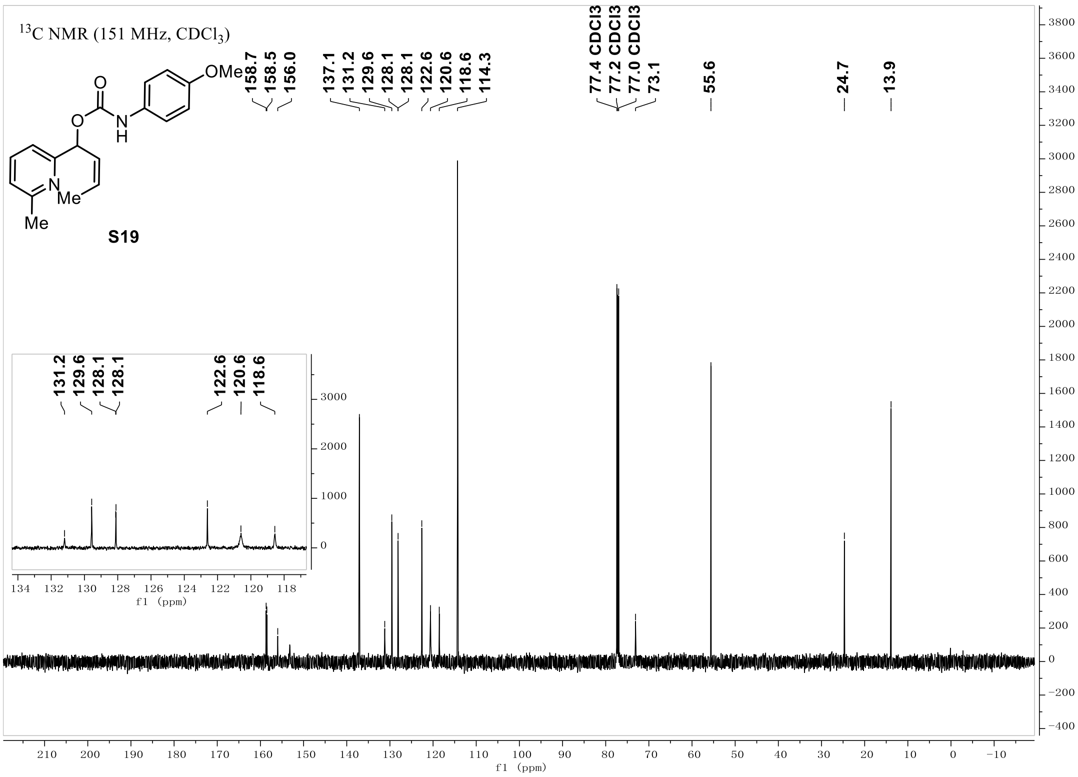

Supplementary Figure 85. ^{1}H NMR and ^{13}C NMR spectra of compound S19.

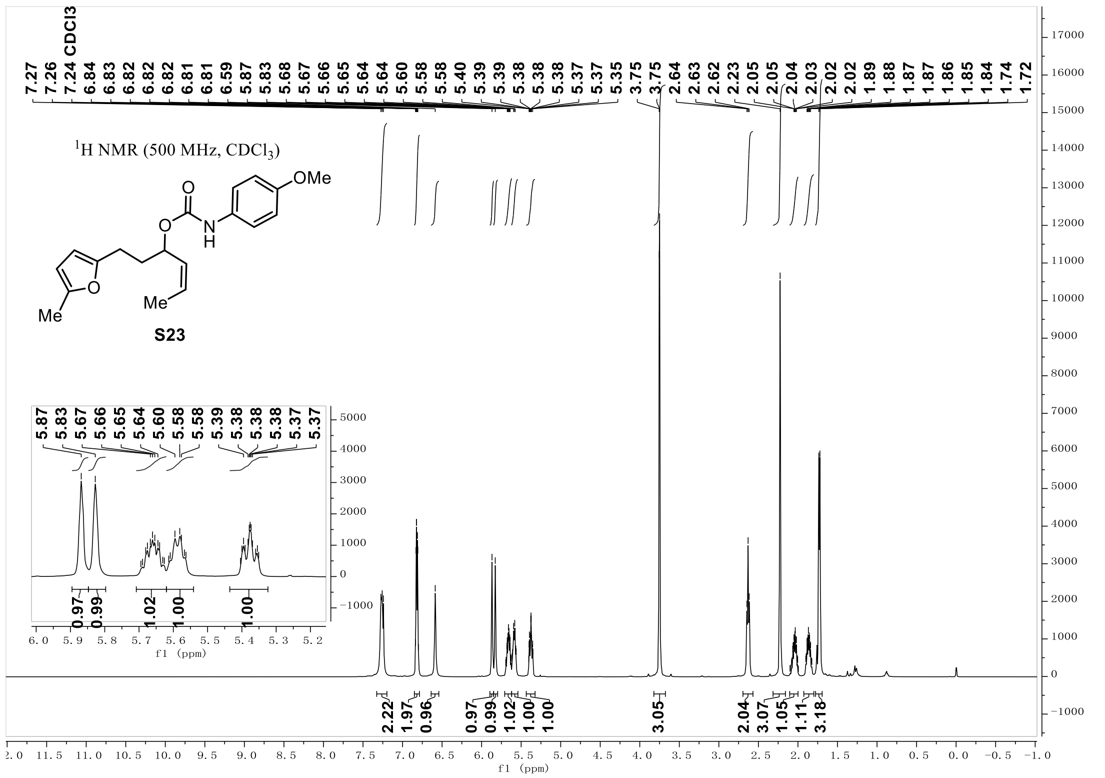

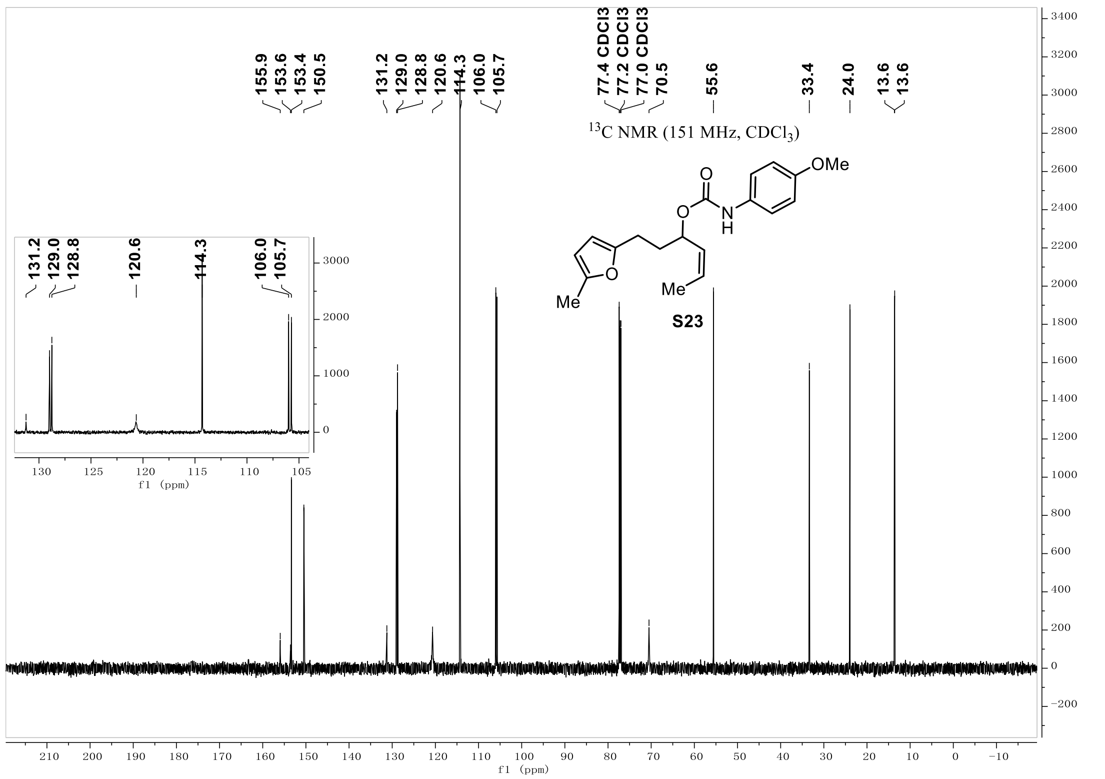

Supplementary Figure 86. ^{1}H NMR and ^{13}C NMR spectra of compound S23.

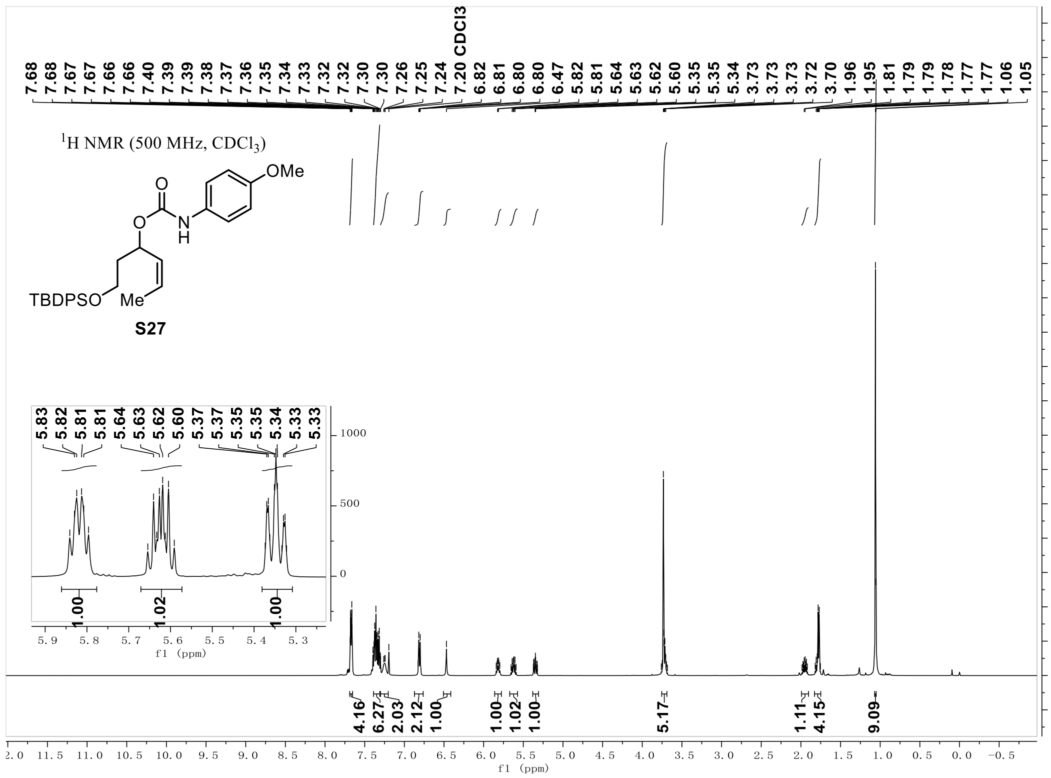

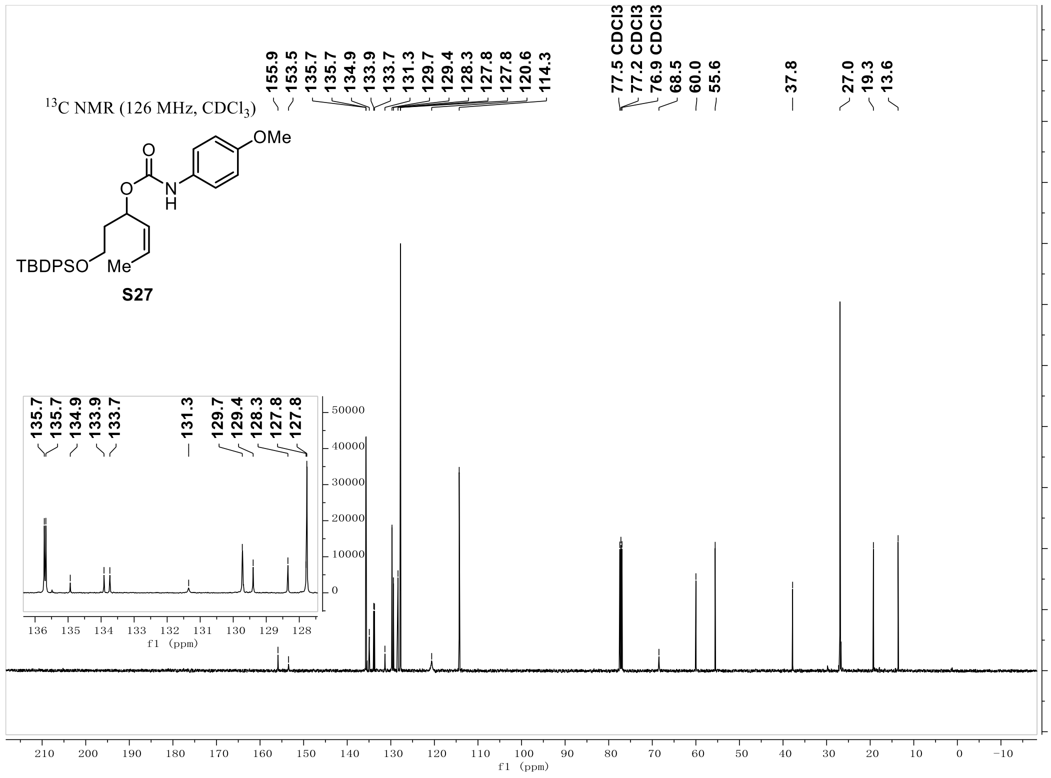

Supplementary Figure 87. ^{1}H NMR and ^{13}C NMR spectra of compound S27.

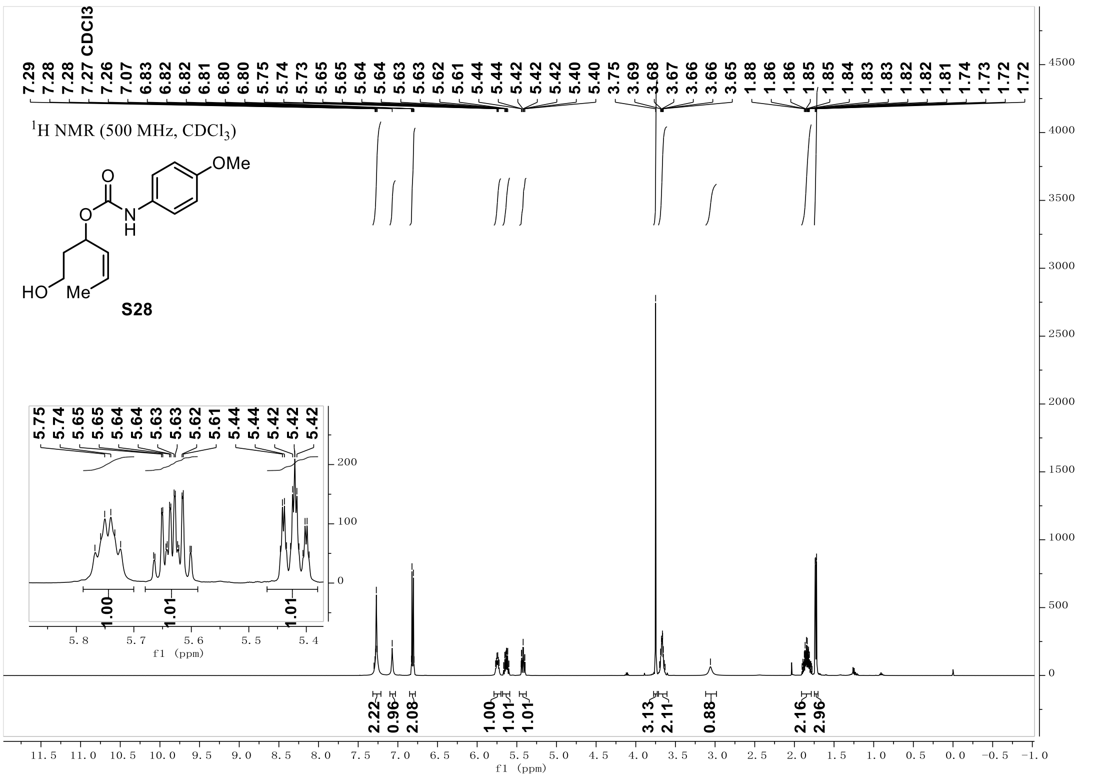

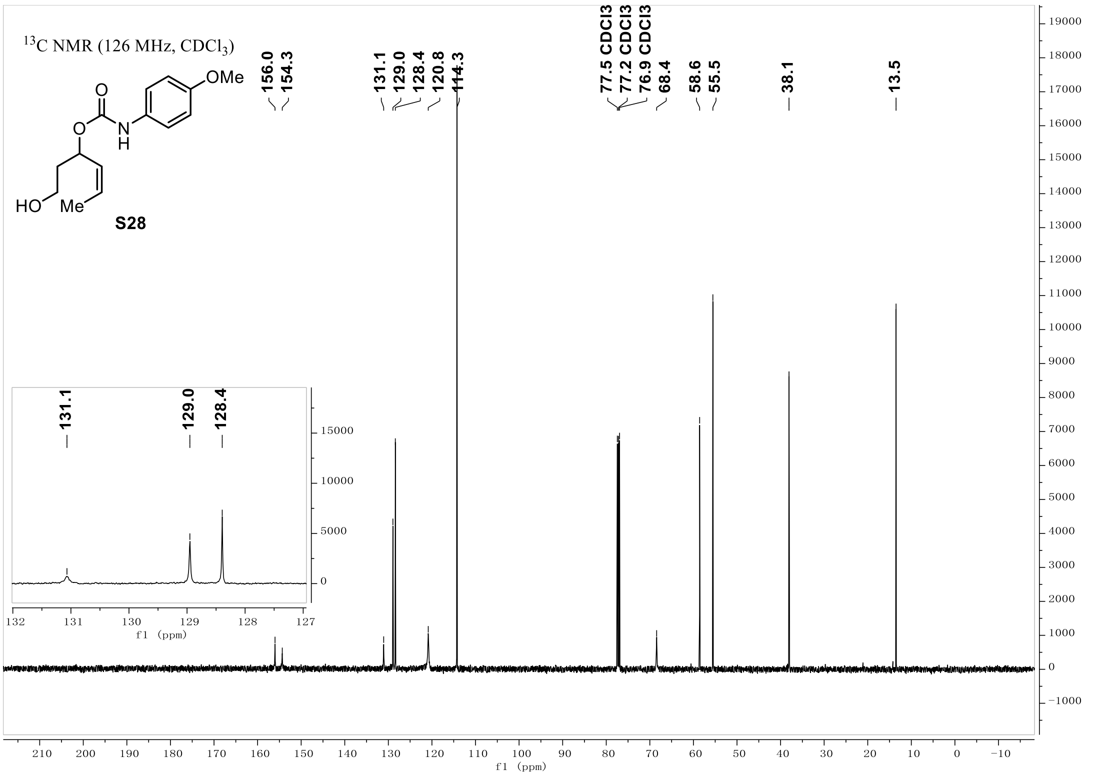

Supplementary Figure 88. ^{1}H NMR and ^{13}C NMR spectra of compound S28.

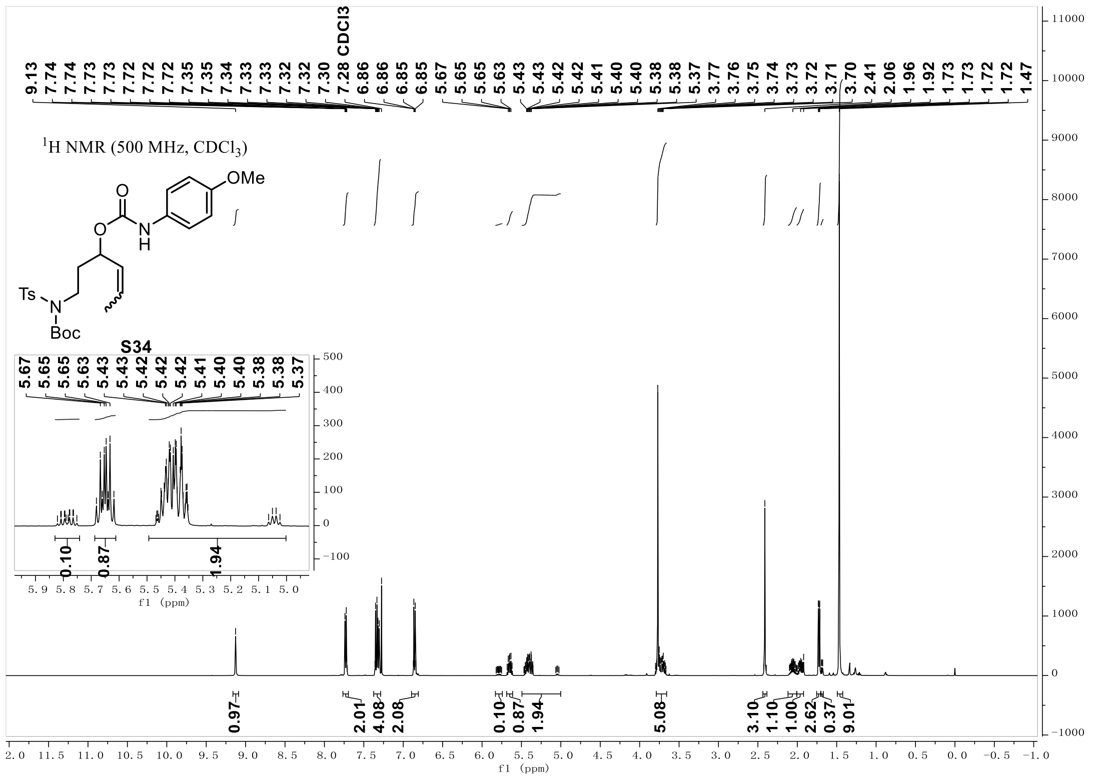

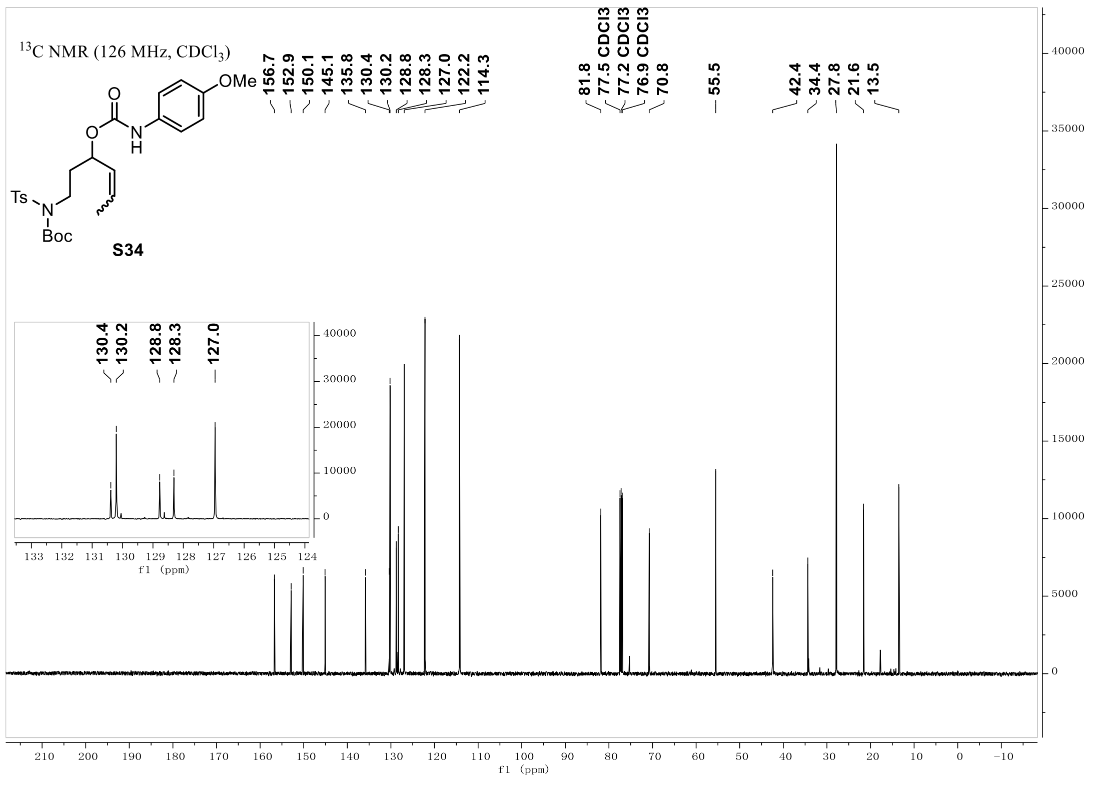

Supplementary Figure 89. ^{1}H NMR and ^{13}C NMR spectra of compound S34. The product was isolated as a 8:1 mixture of *Z*/*E* isomers.

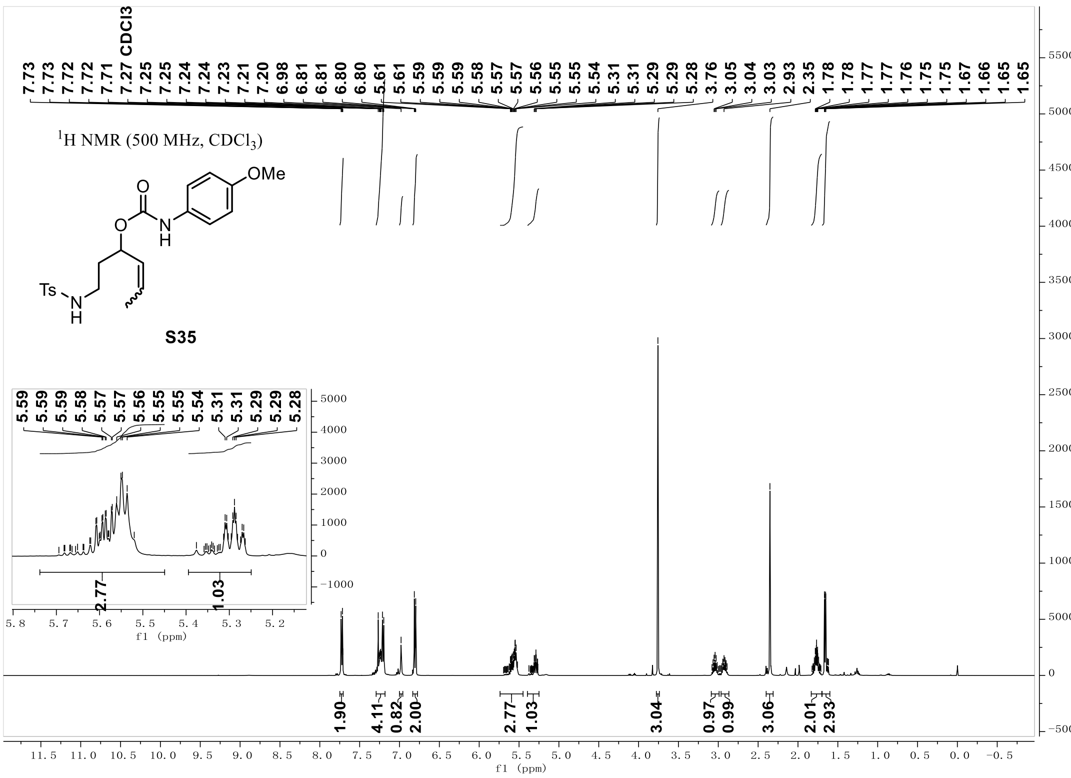

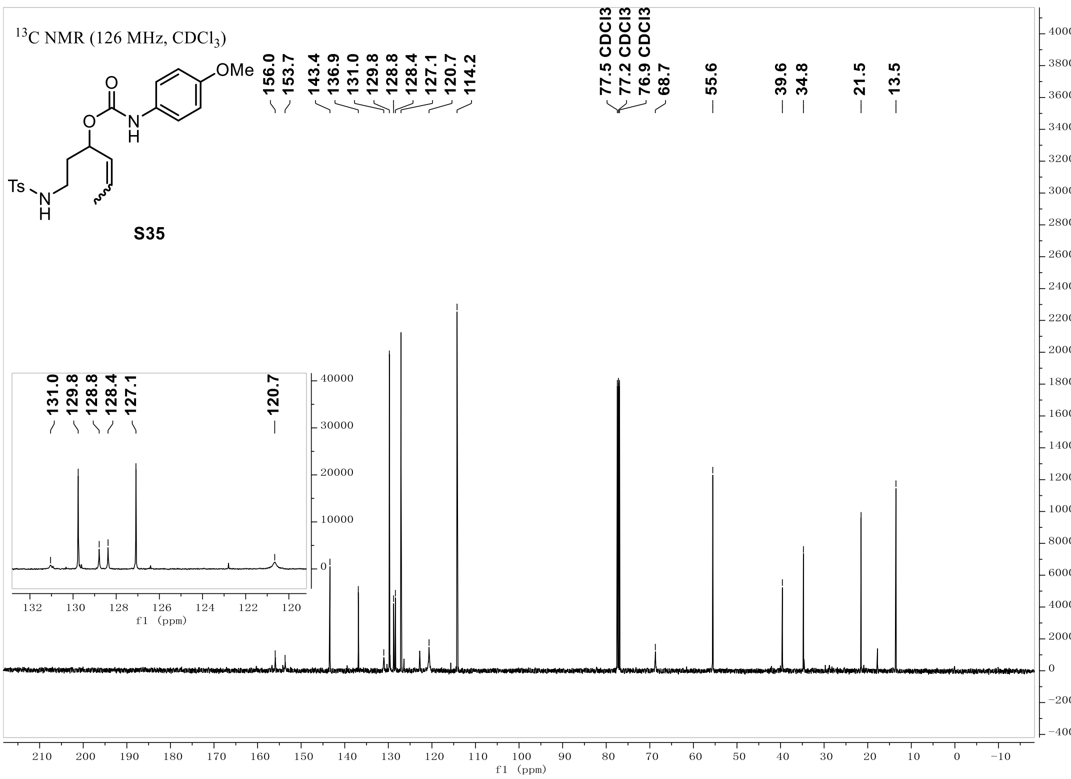

Supplementary Figure 90. ^{1}H NMR and ^{13}C NMR spectra of compound S35. The product was isolated as a 8:1 mixture of *Z*/*E* isomers.

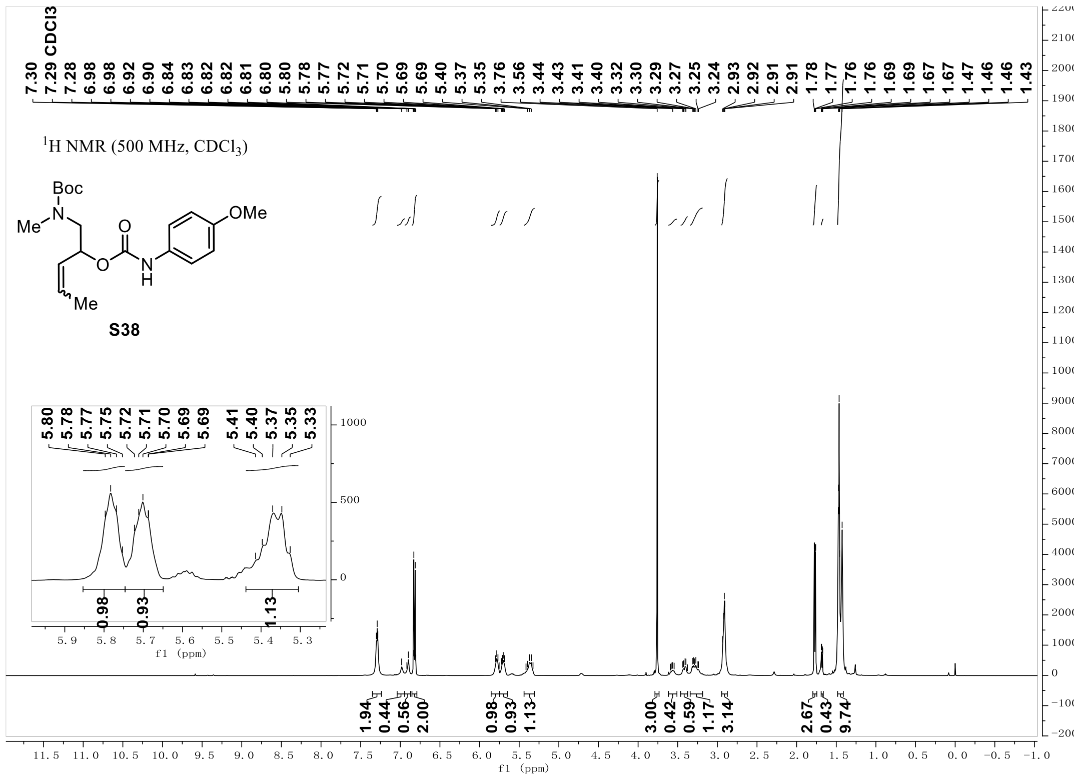

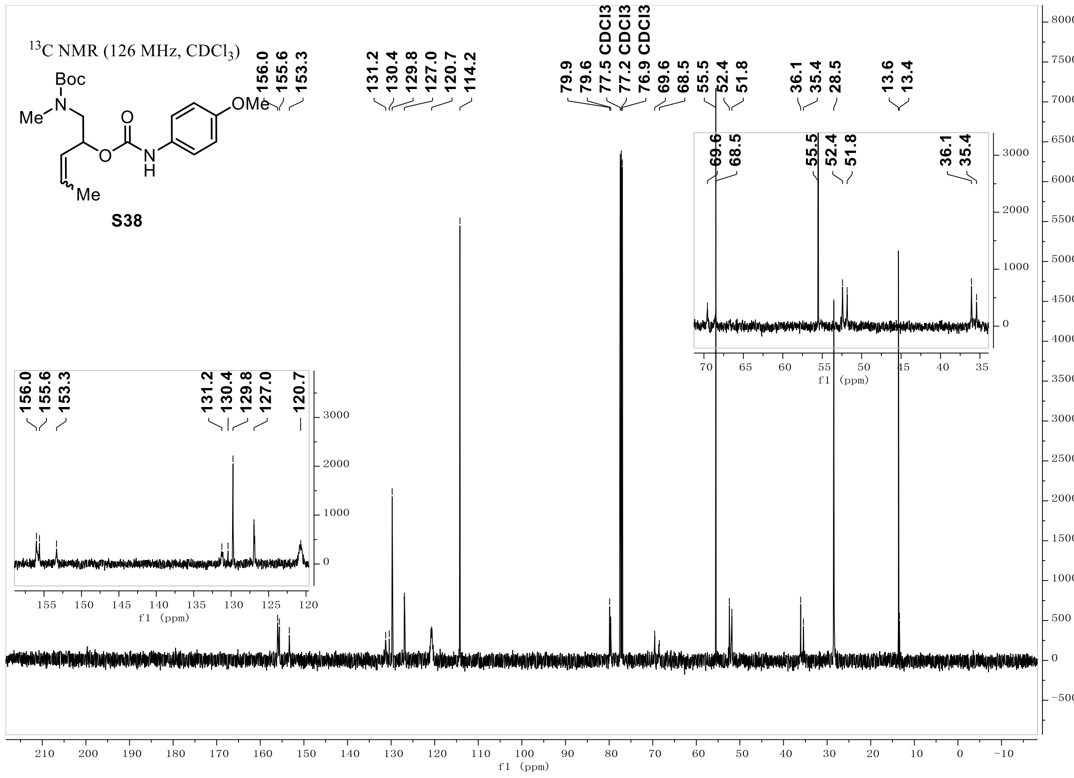

Supplementary Figure 91. ^{1}H NMR and ^{13}C NMR spectra of compound S38. The product was isolated as a 3:2 mixture of rotary isomers with 6:1 *Z*/*E*.

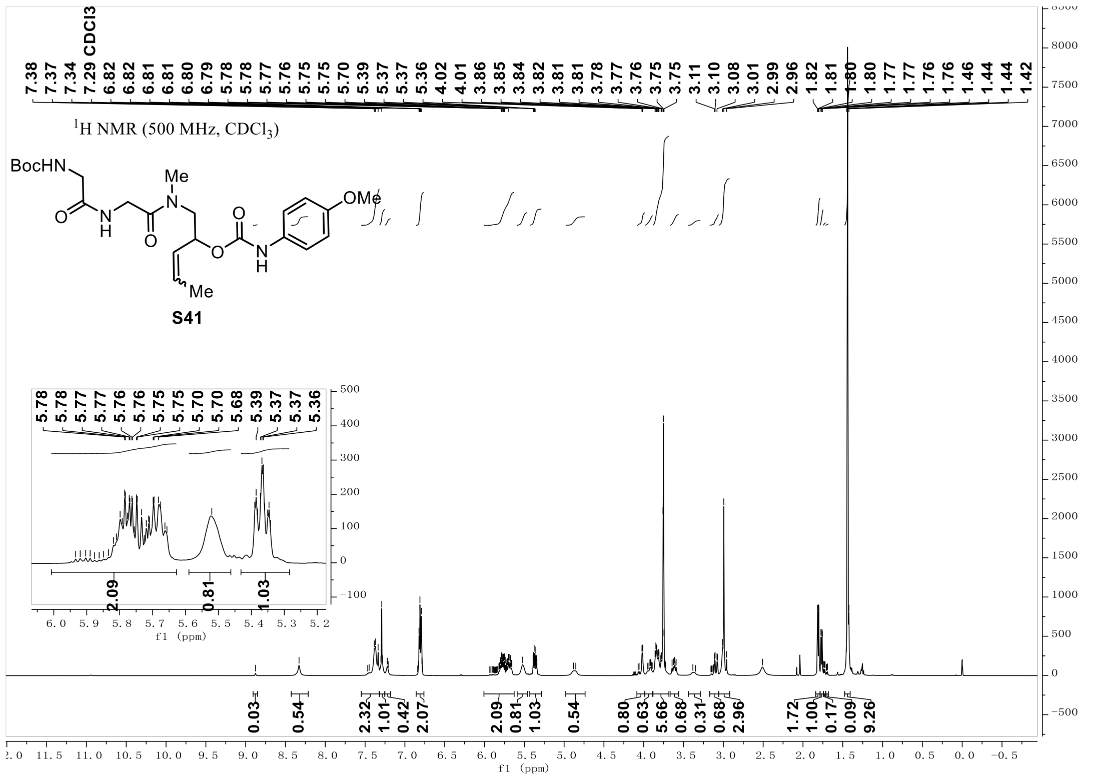

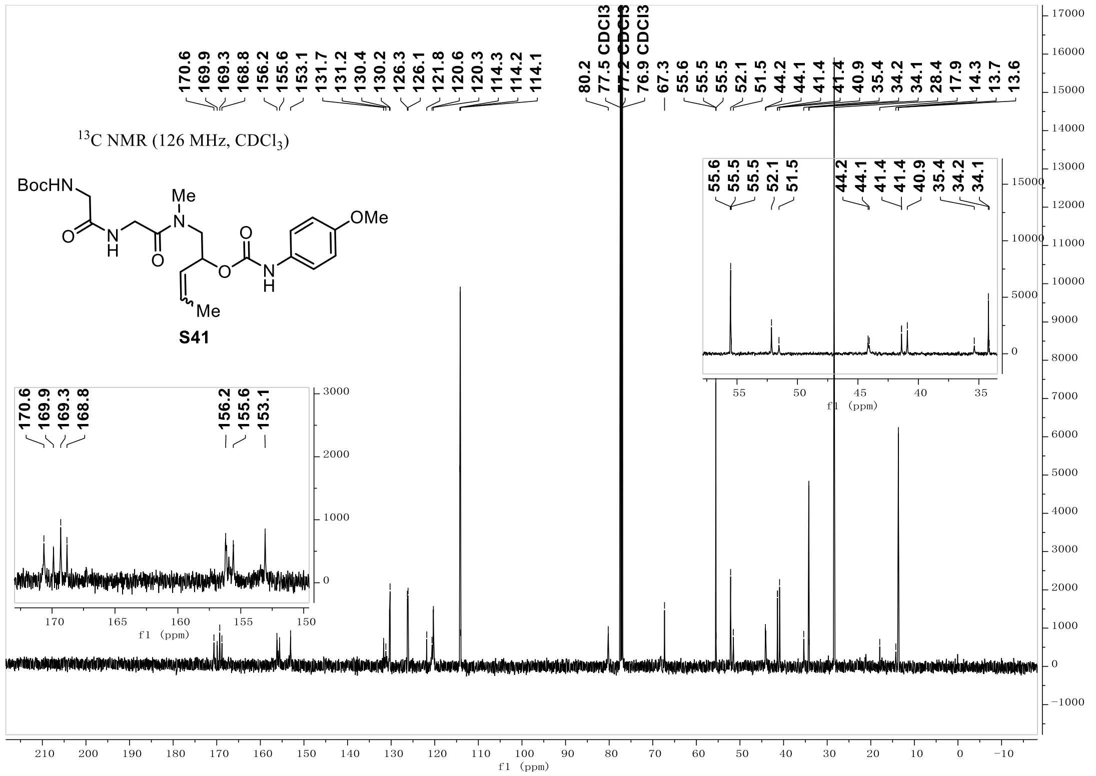

Supplementary Figure 92. ^{1}H NMR and ^{13}C NMR spectra of compound S41. The produvt was isolated as a 3:2 mixture of rotary isomers with 10:1 *Z*/*E*.

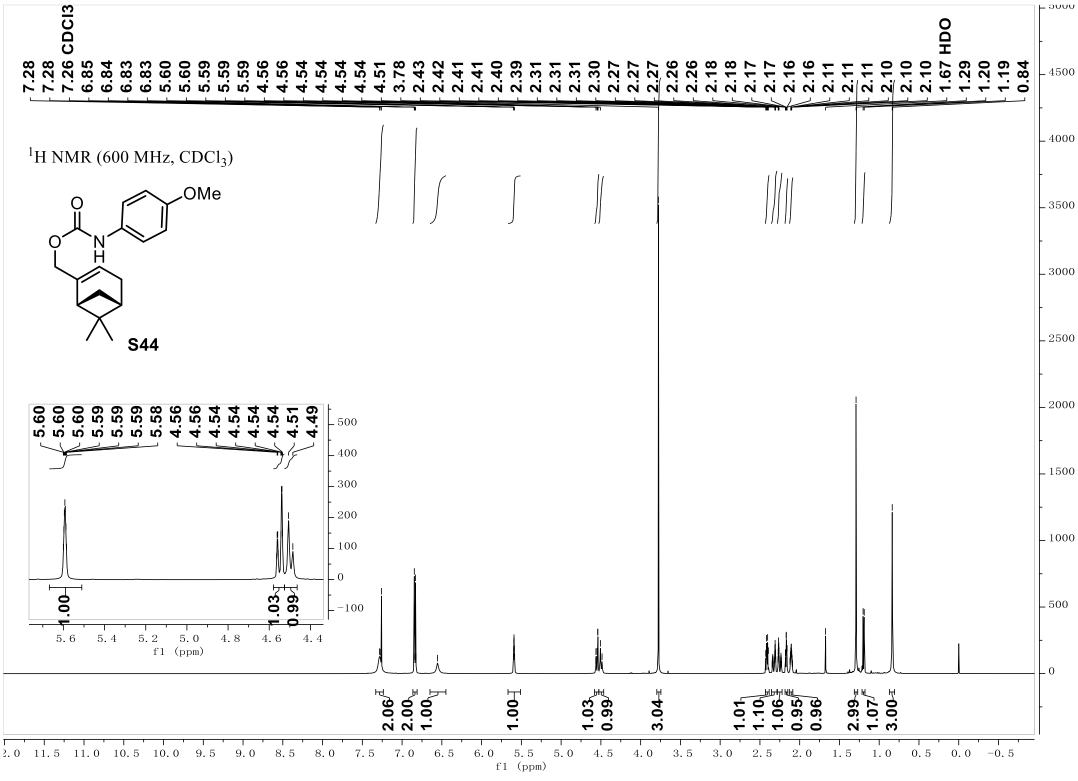

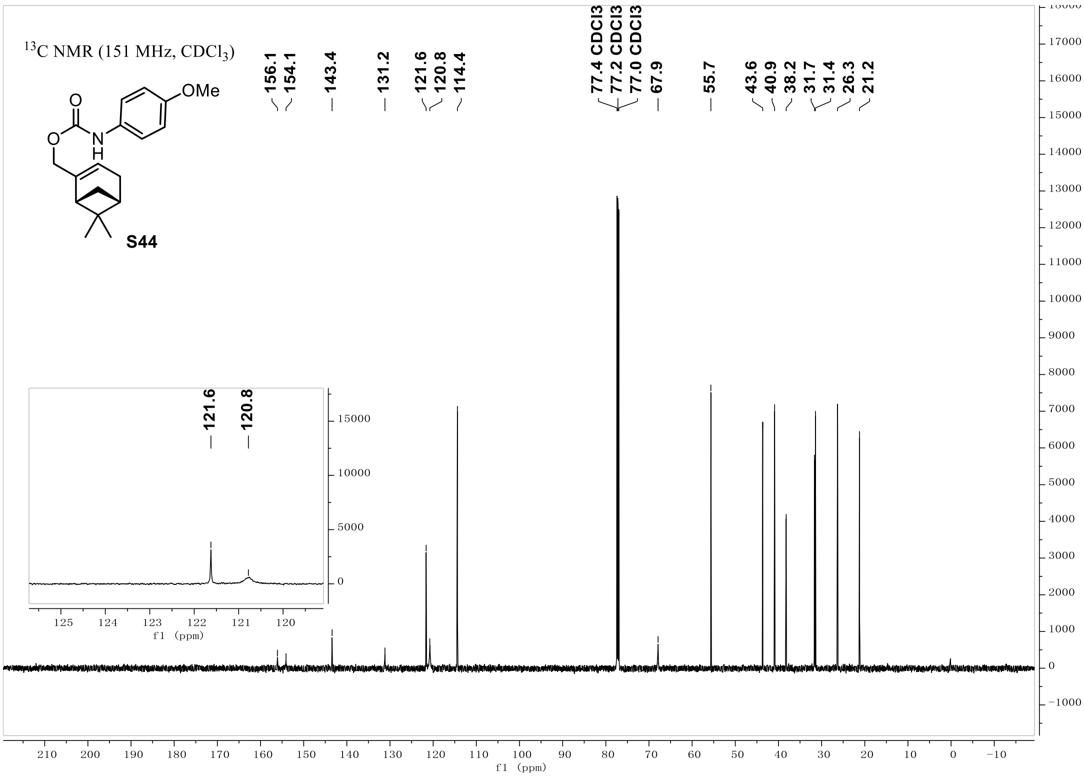

Supplementary Figure 93. ^{1}H NMR and ^{13}C NMR spectra of compound S44.

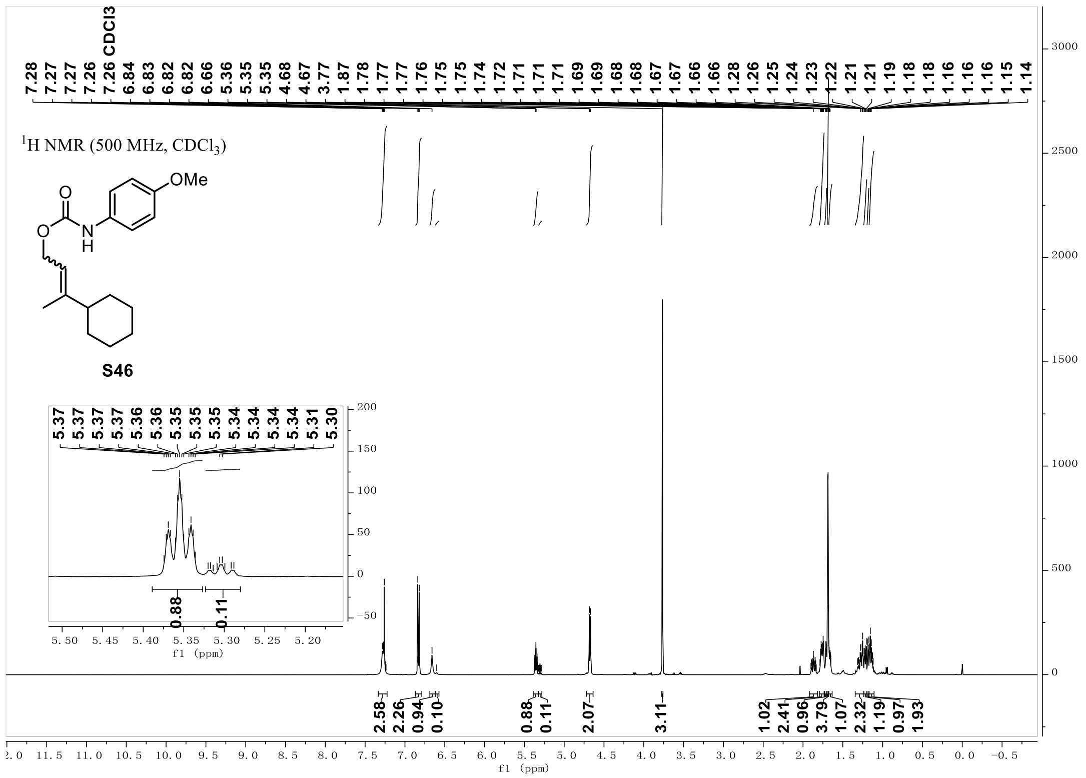

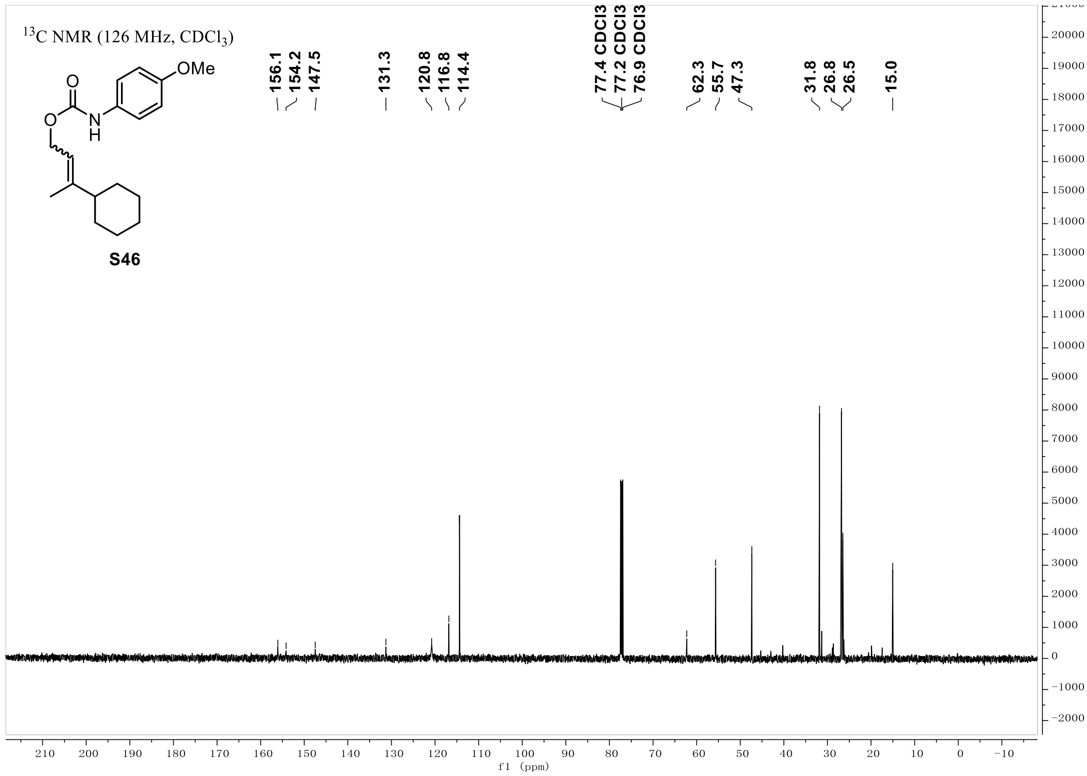

Supplementary Figure 94. ^{1}H NMR and ^{13}C NMR spectra of compound S46.

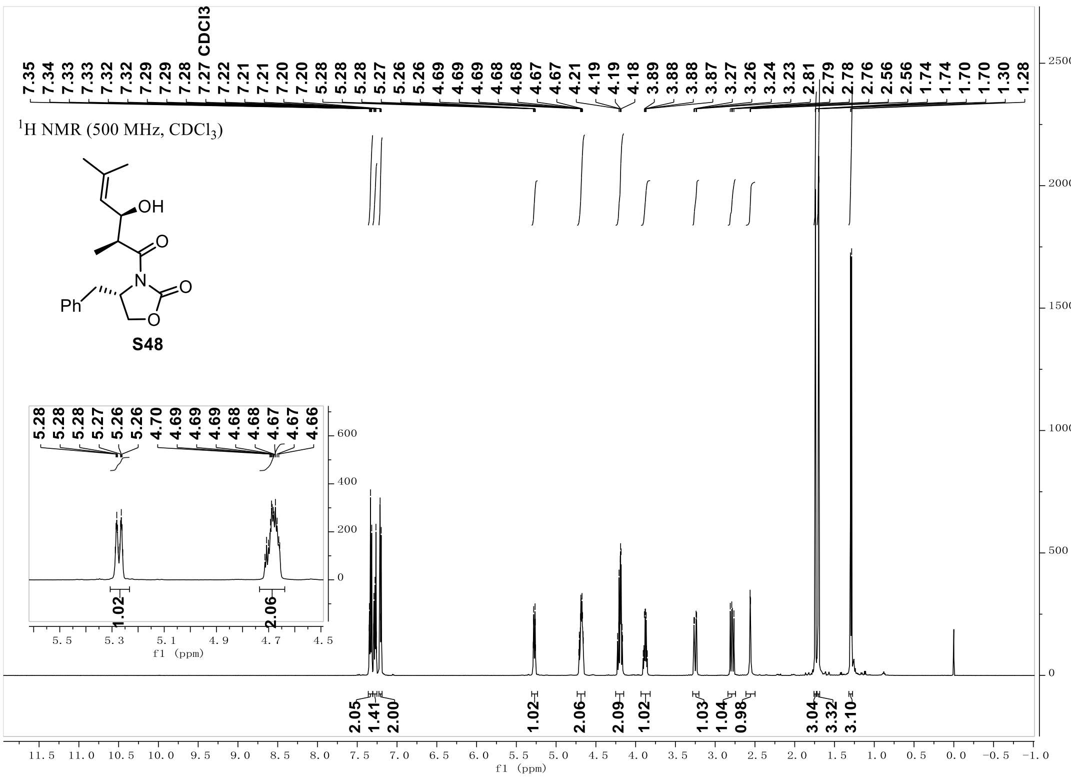

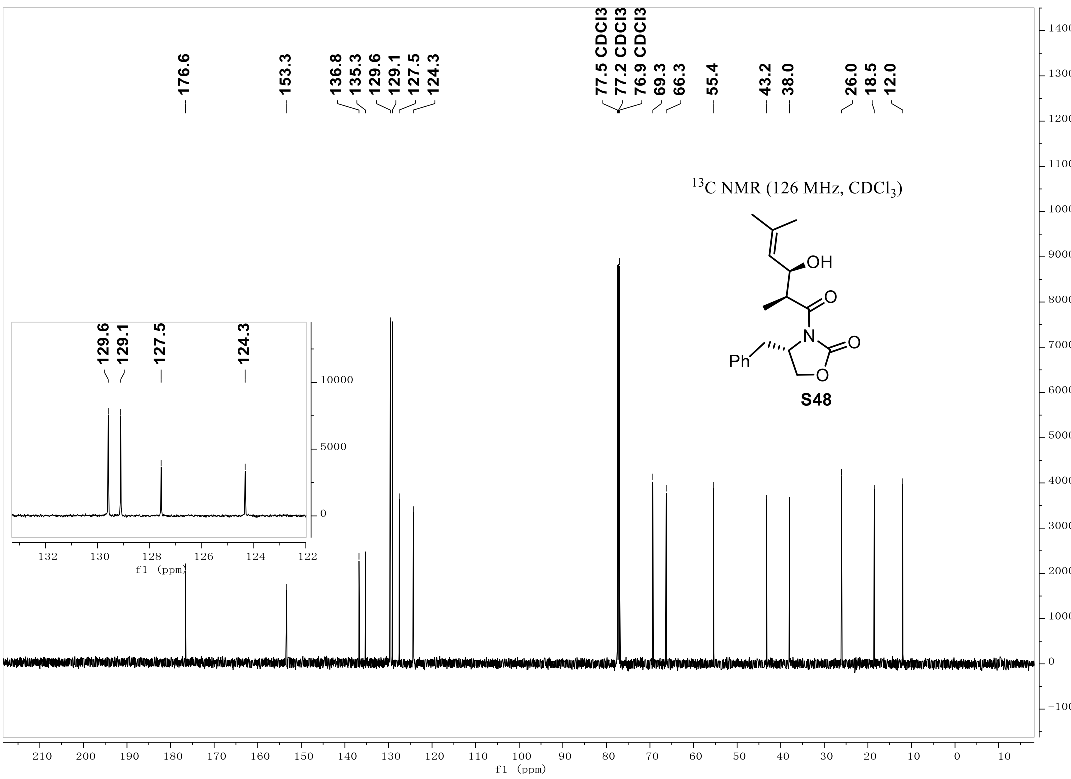

Supplementary Figure 95. ^{1}H NMR and ^{13}C NMR spectra of compound S48.

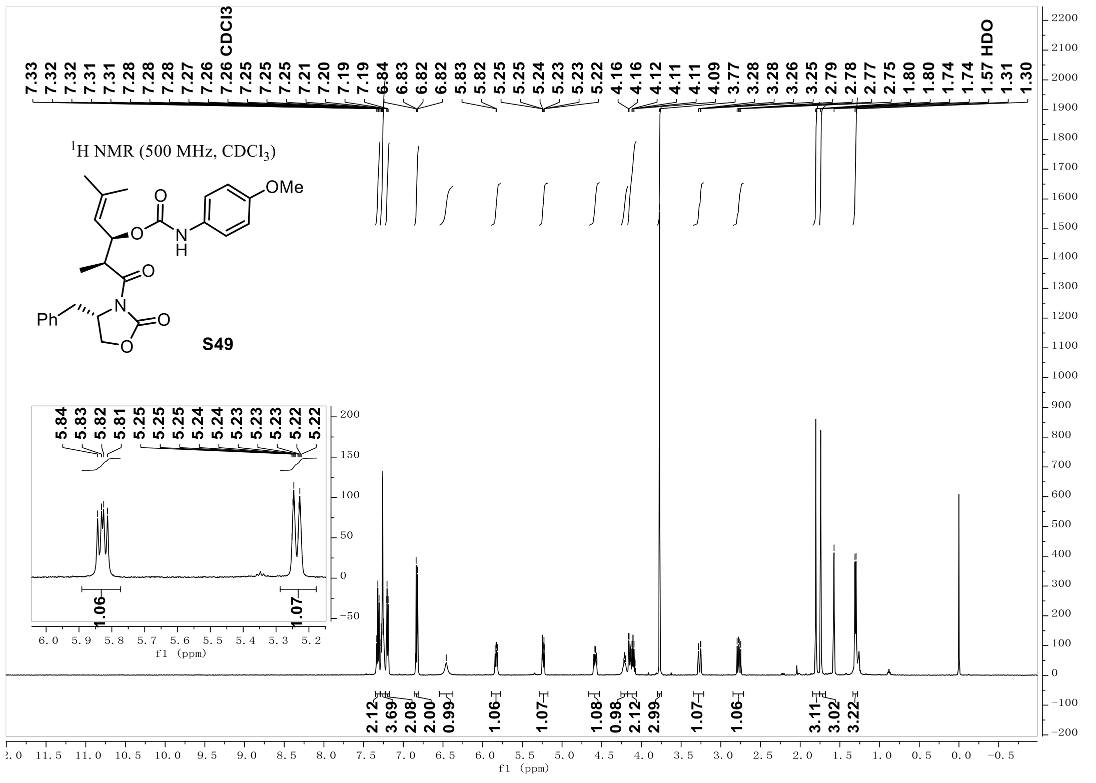

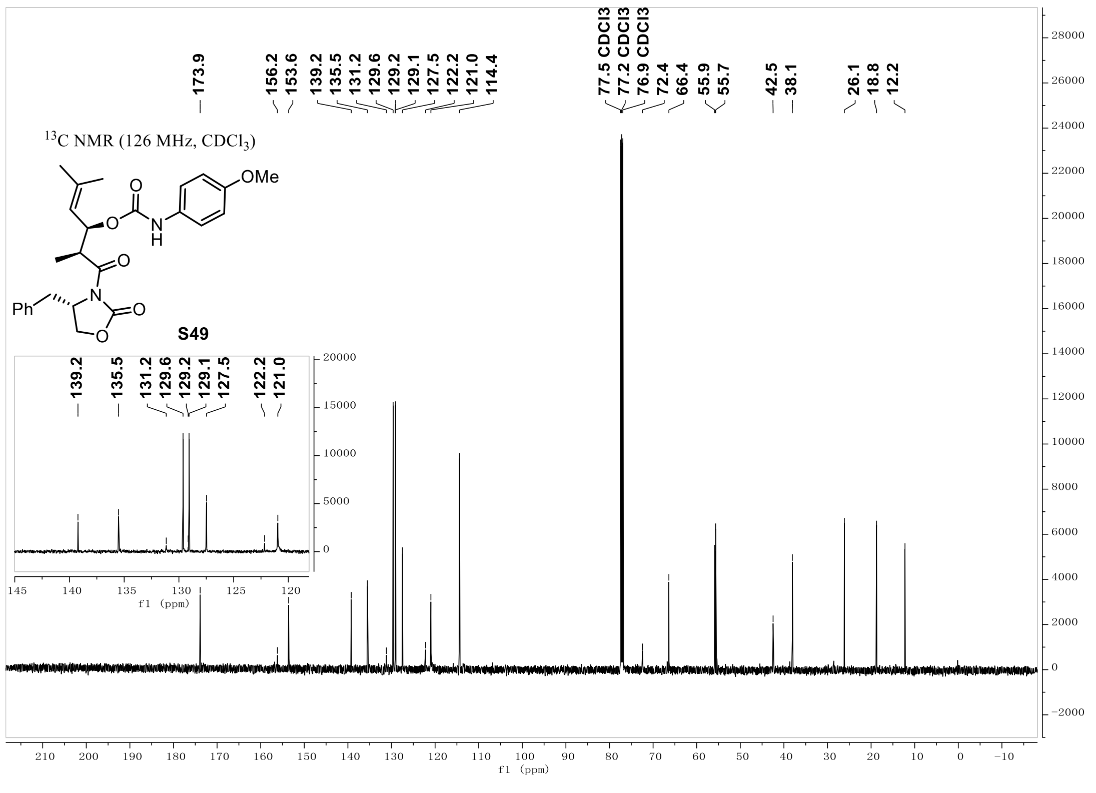

Supplementary Figure 96. ^{1}H NMR and ^{13}C NMR spectra of compound S49.

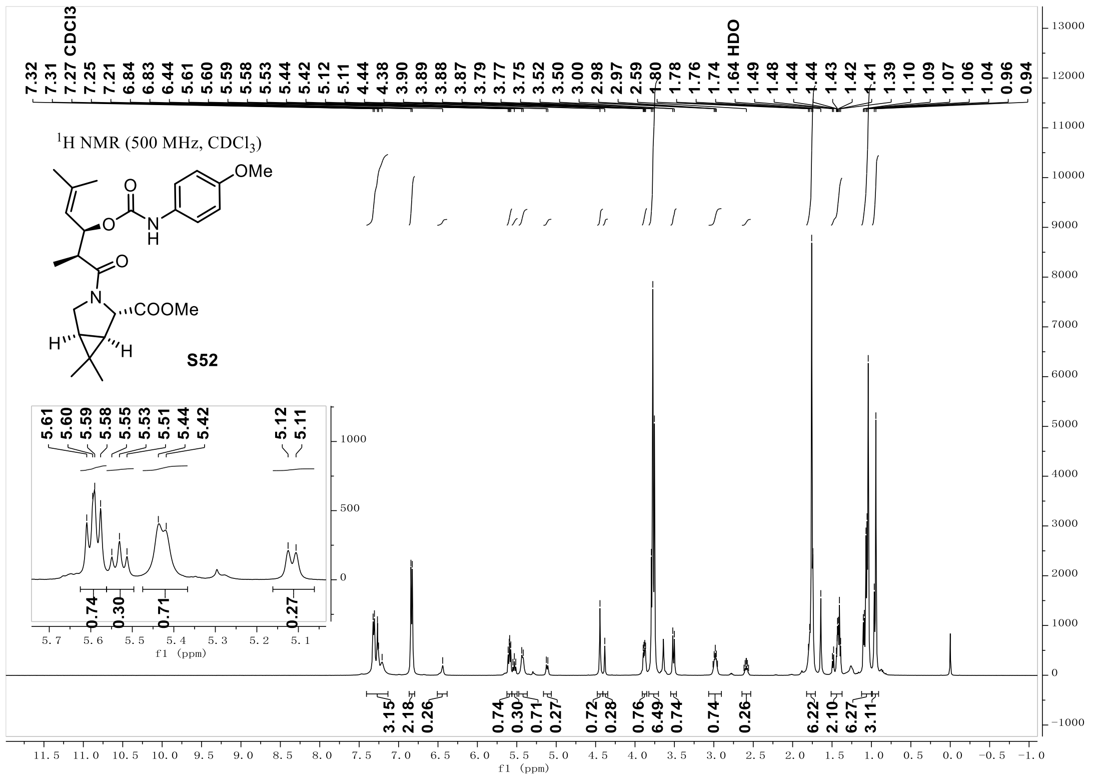

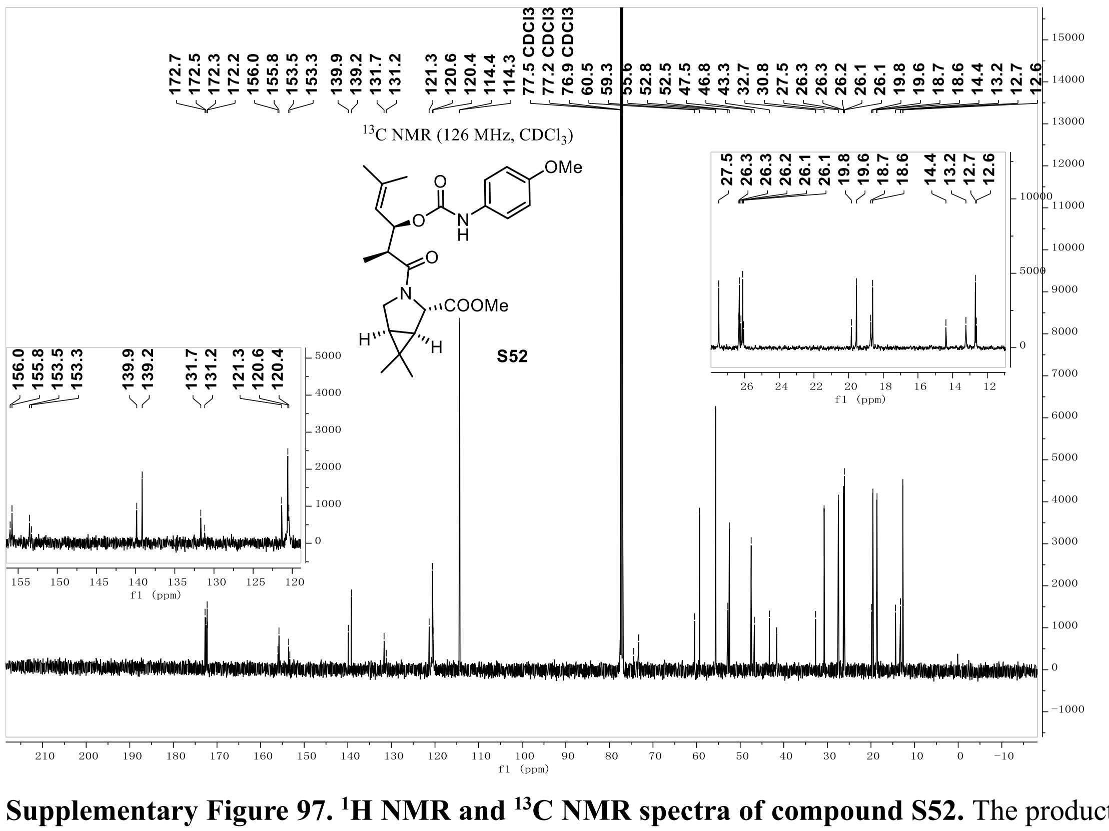

Supplementary Figure 97. ^{1}H NMR and ^{13}C NMR spectra of compound S52. The product was isolated as a 7:3 mixture of rotary isomers.

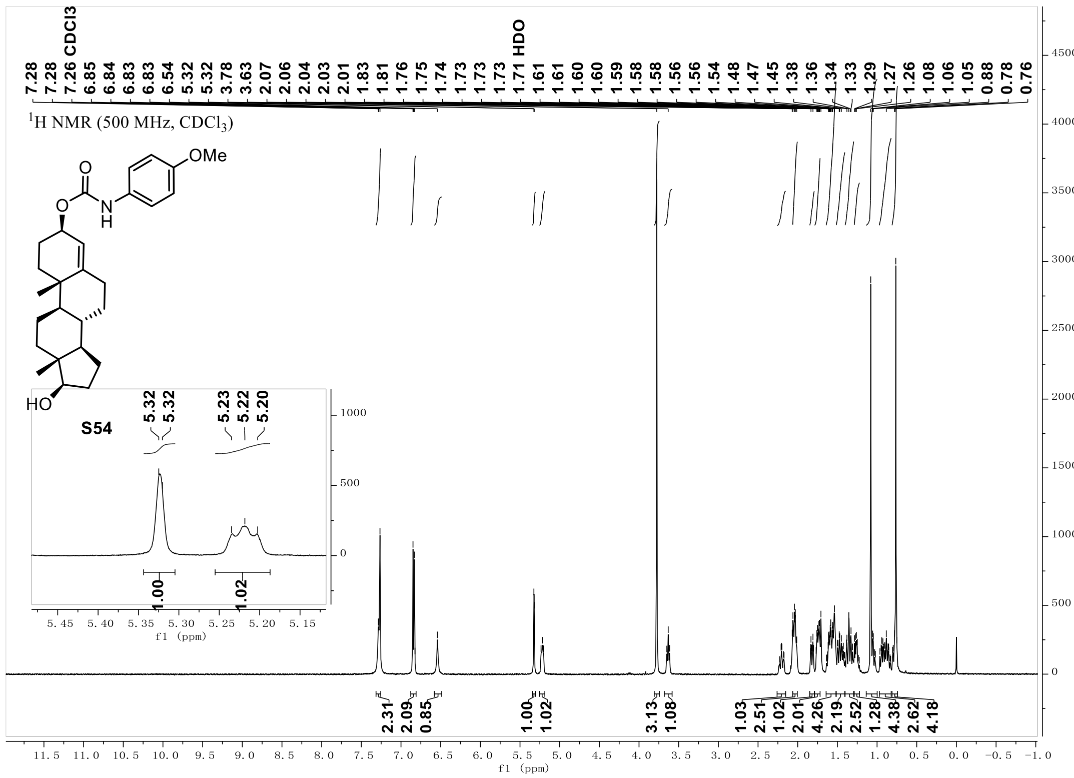

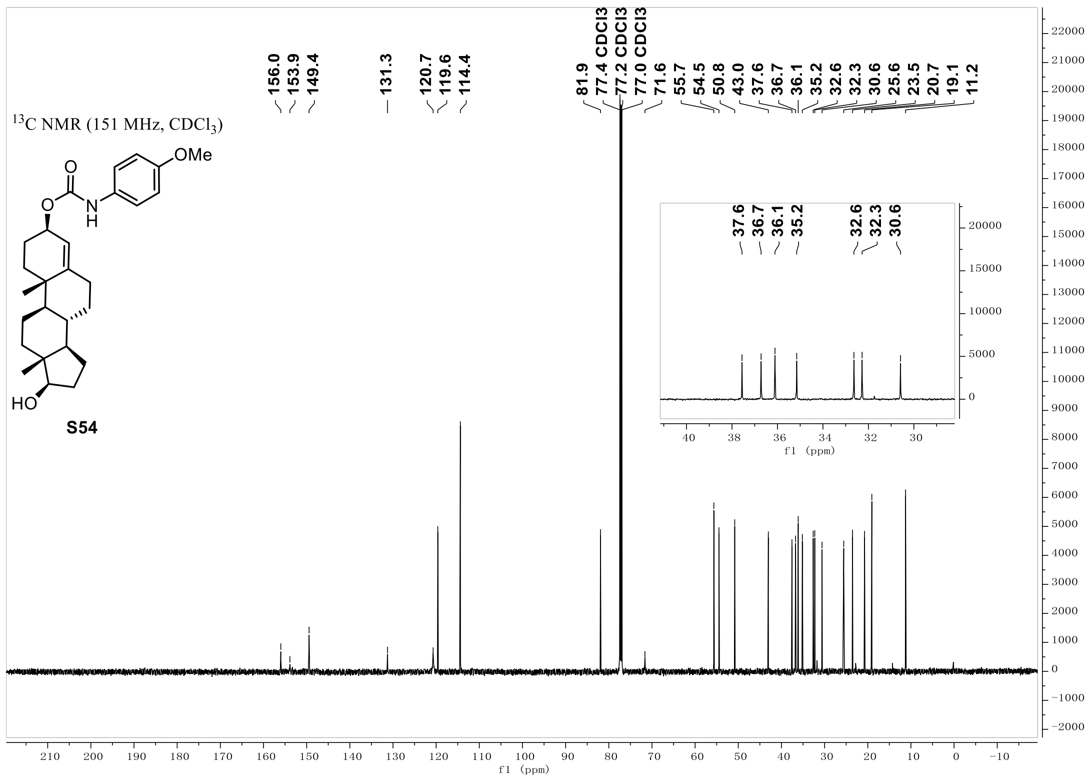

Supplementary Figure 98. ^{1}H NMR and ^{13}C NMR spectra of compound S54.

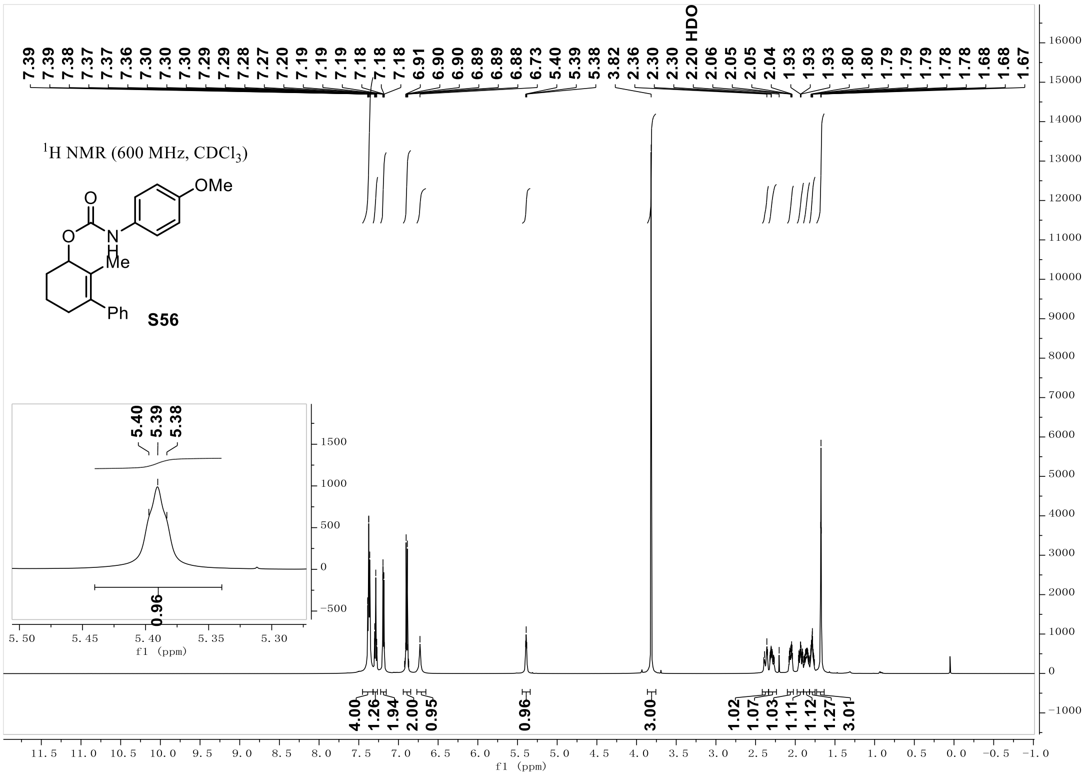

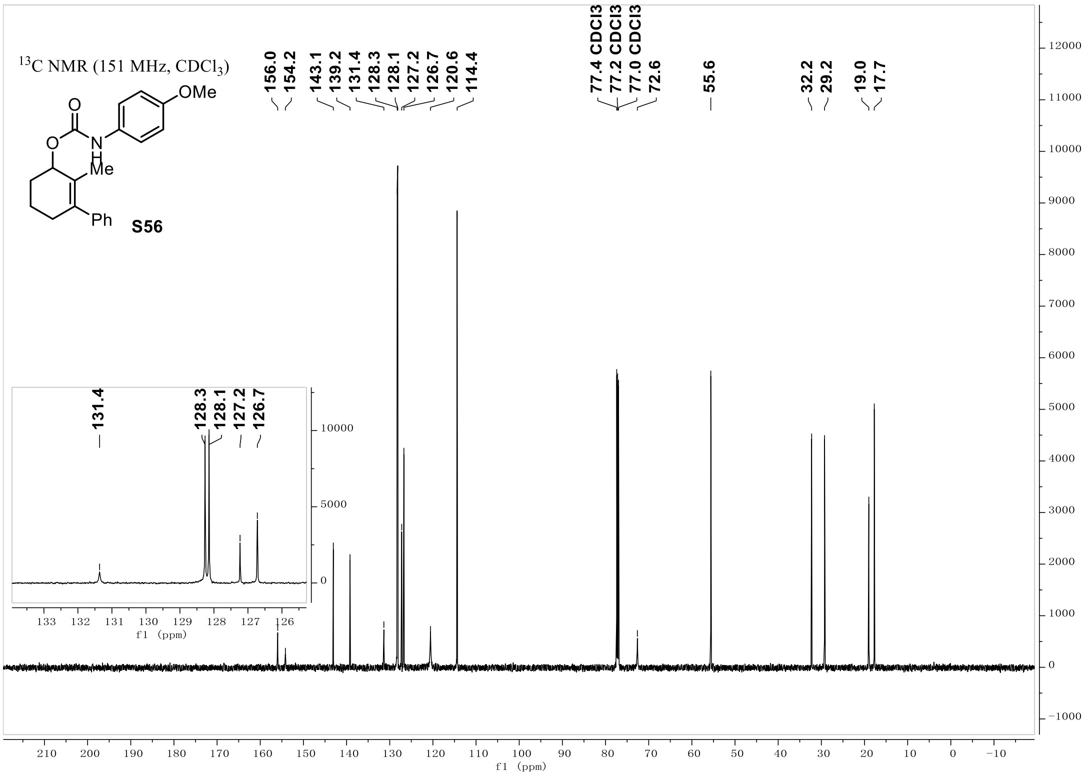

Supplementary Figure 99. ^{1}H NMR and ^{13}C NMR spectra of compound S56.

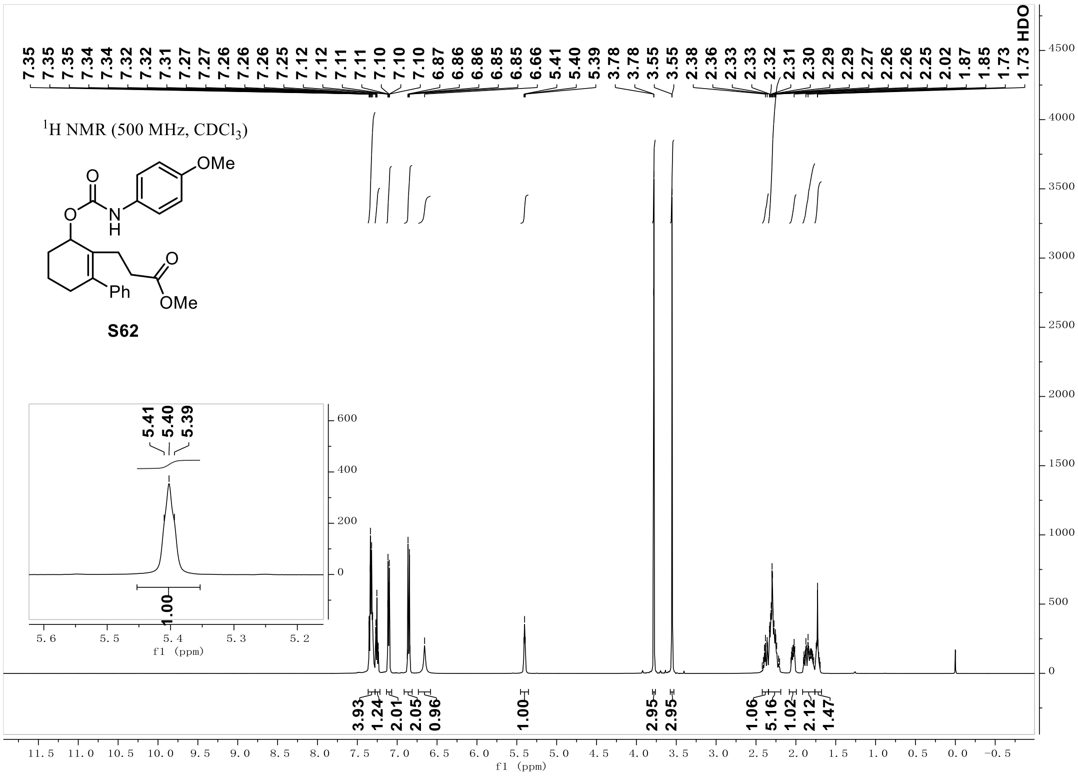

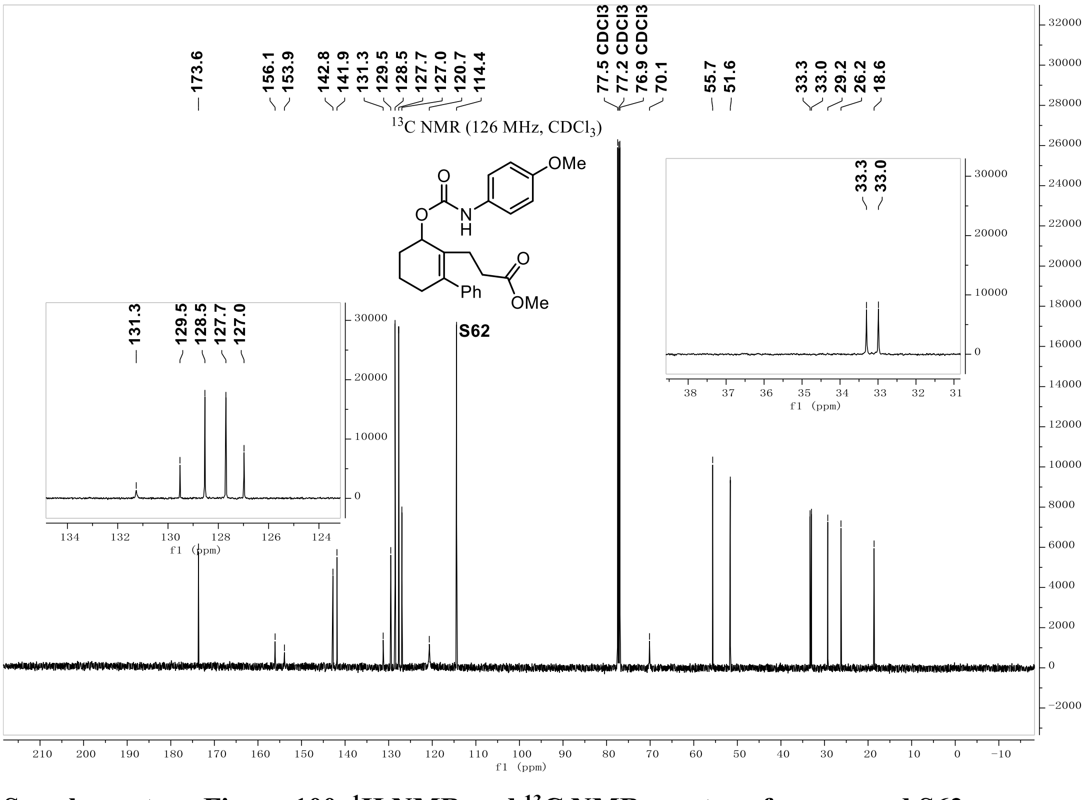

Supplementary Figure 100. ^{1}H NMR and ^{13}C NMR spectra of compound S62.

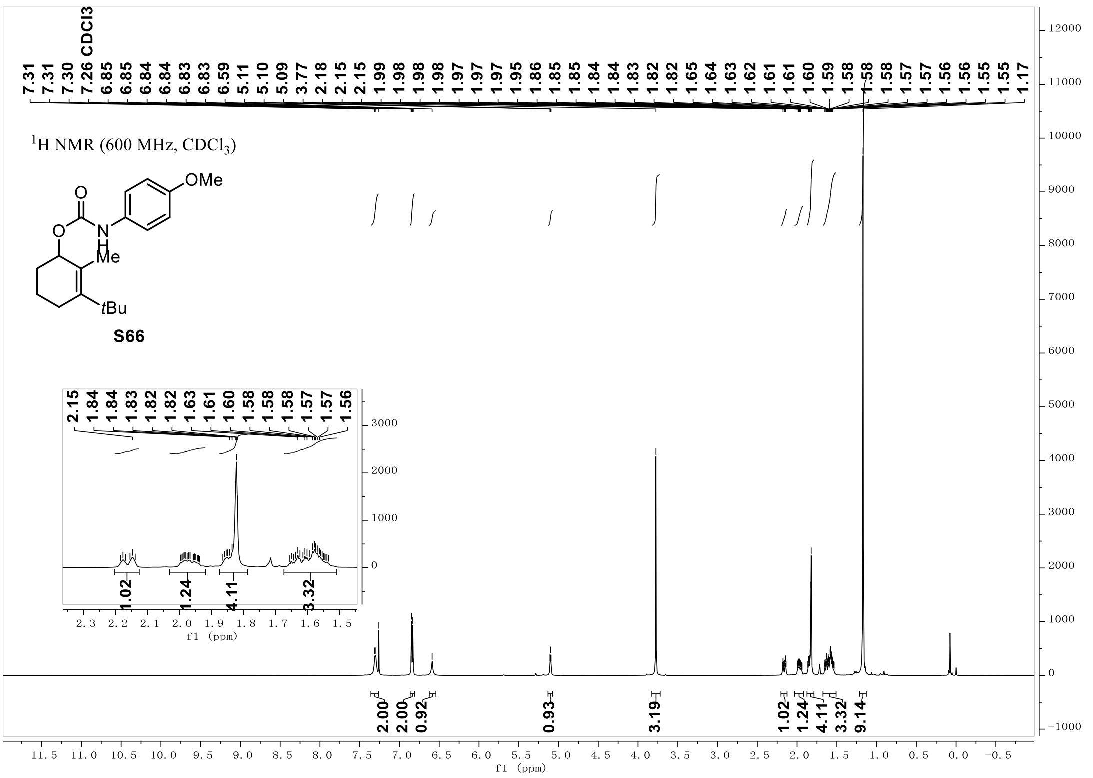

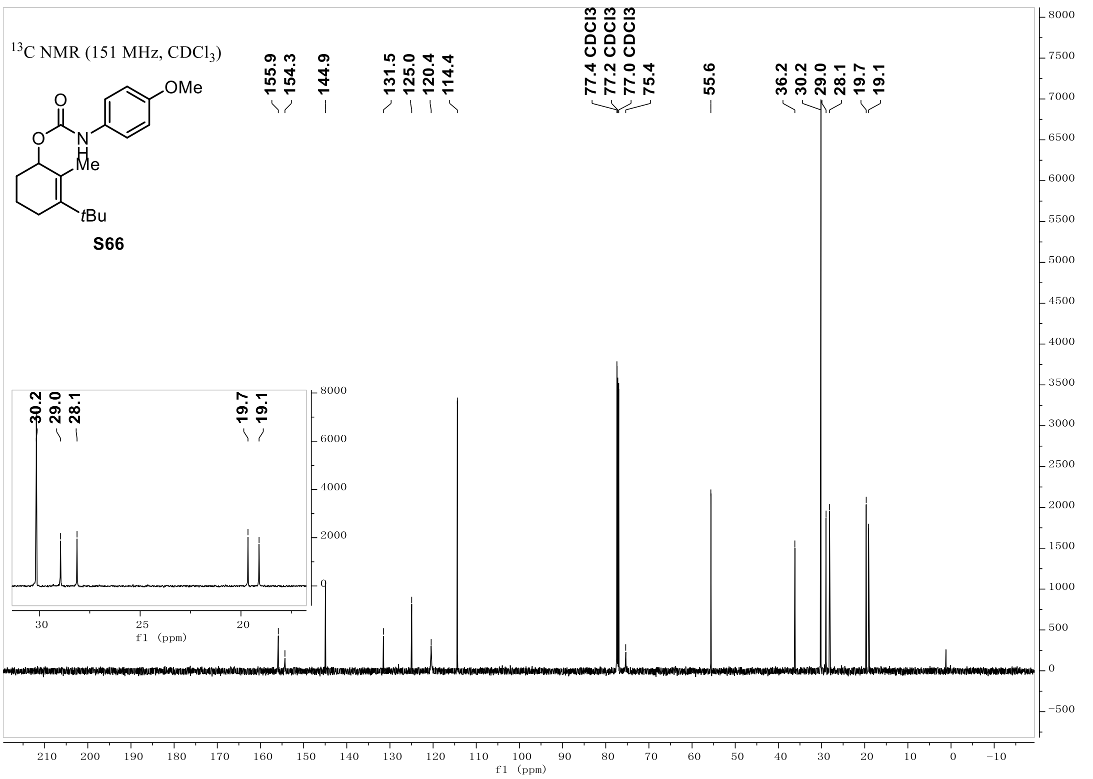

Supplementary Figure 101. ^{1}H NMR and ^{13}C NMR spectra of compound S66.

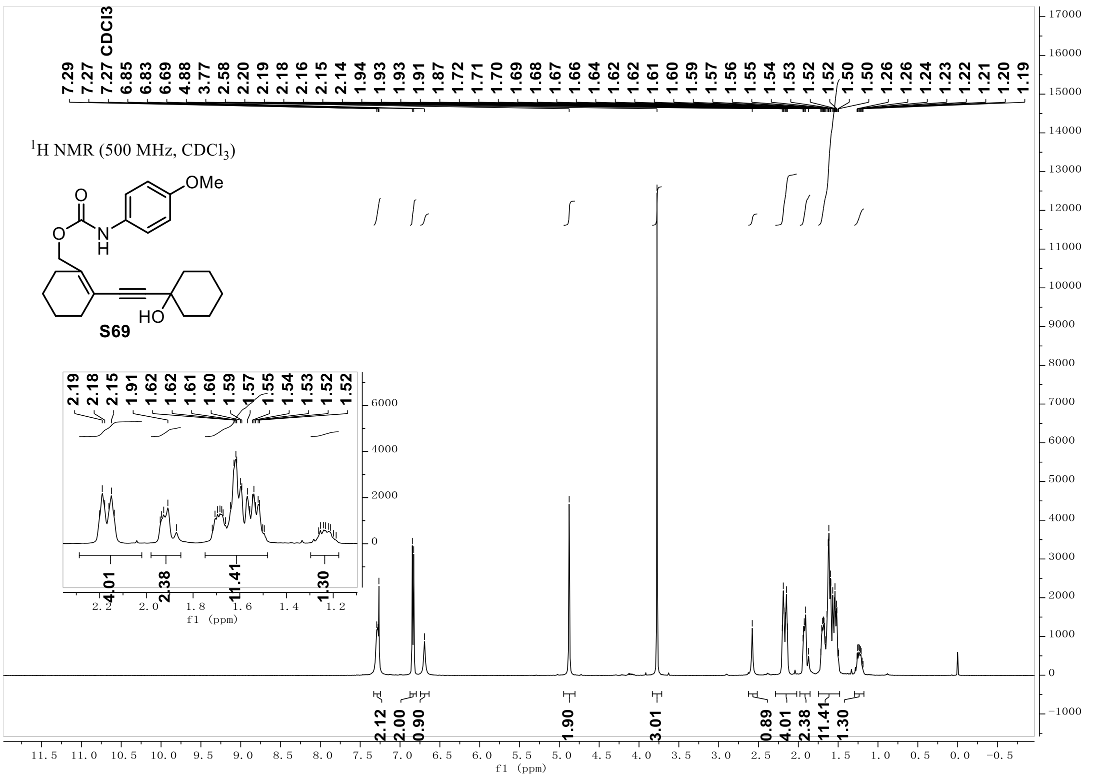

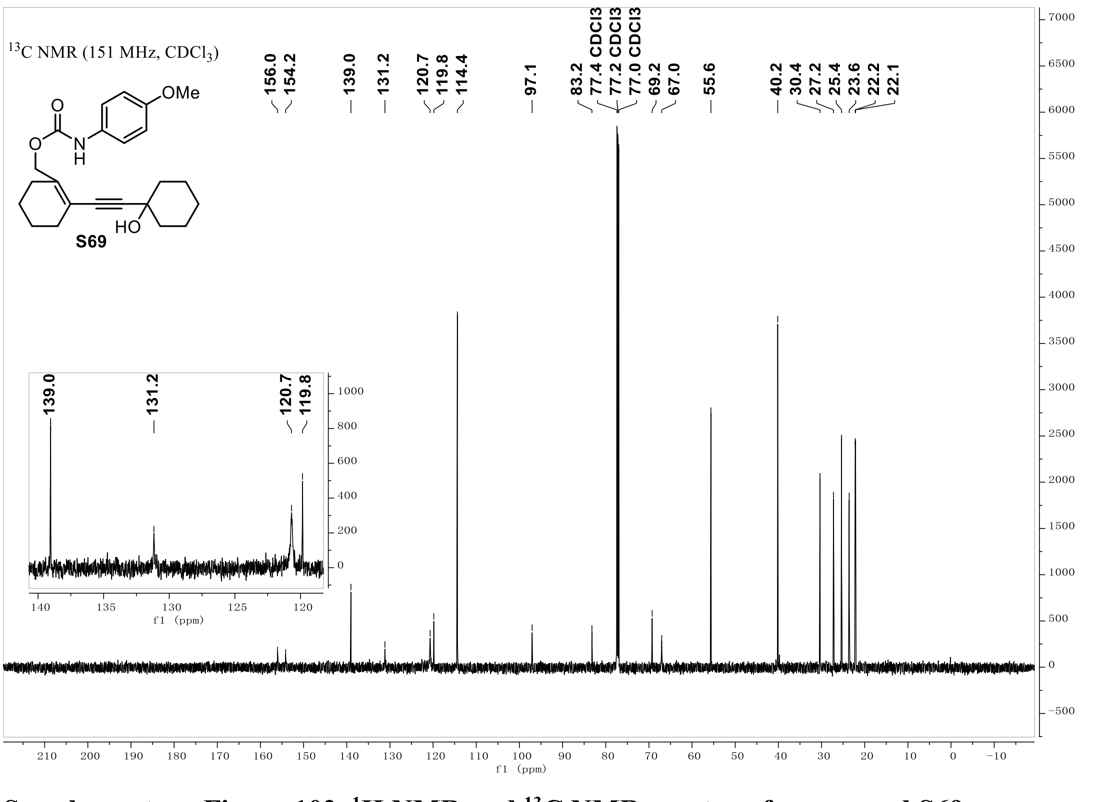

Supplementary Figure 102. ^{1}H NMR and ^{13}C NMR spectra of compound S69.

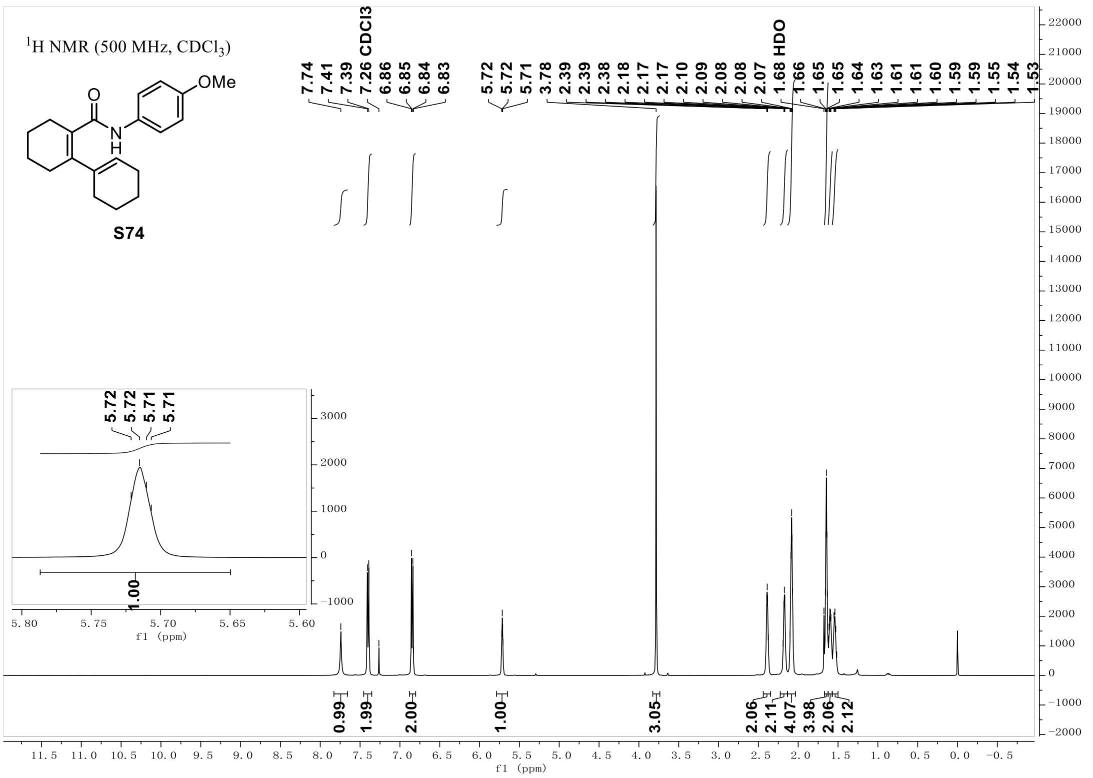

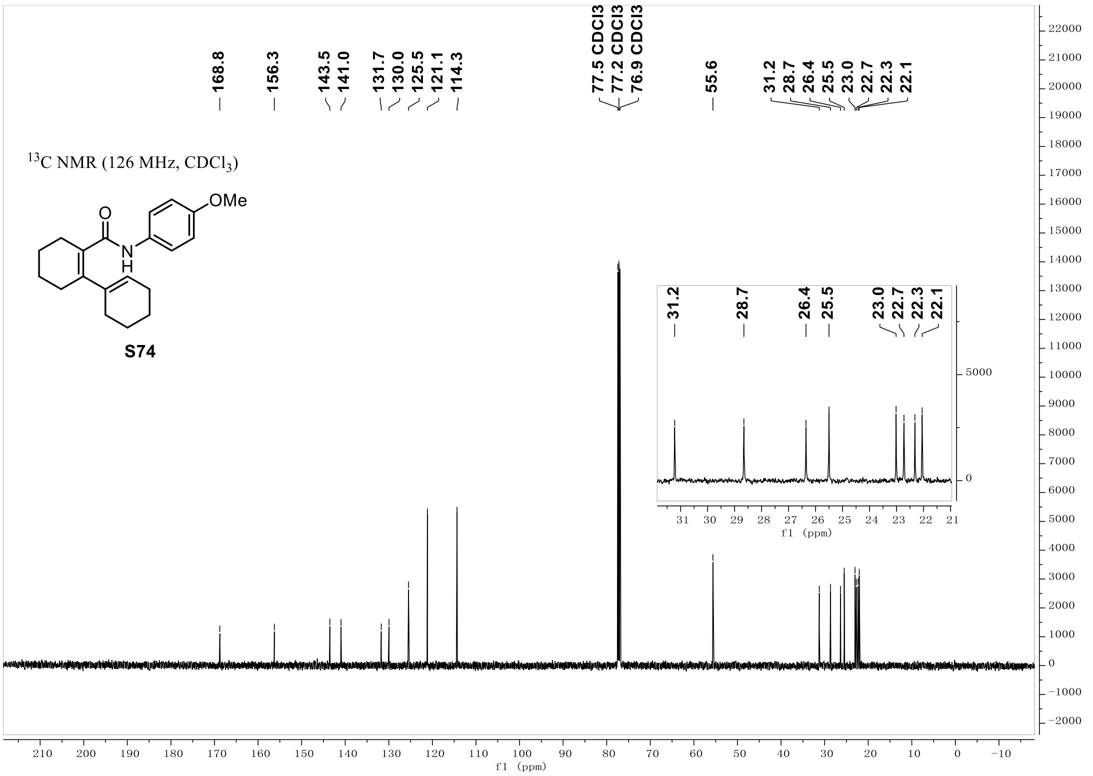

Supplementary Figure 103. ^{1}H NMR and ^{13}C NMR spectra of compound S74.

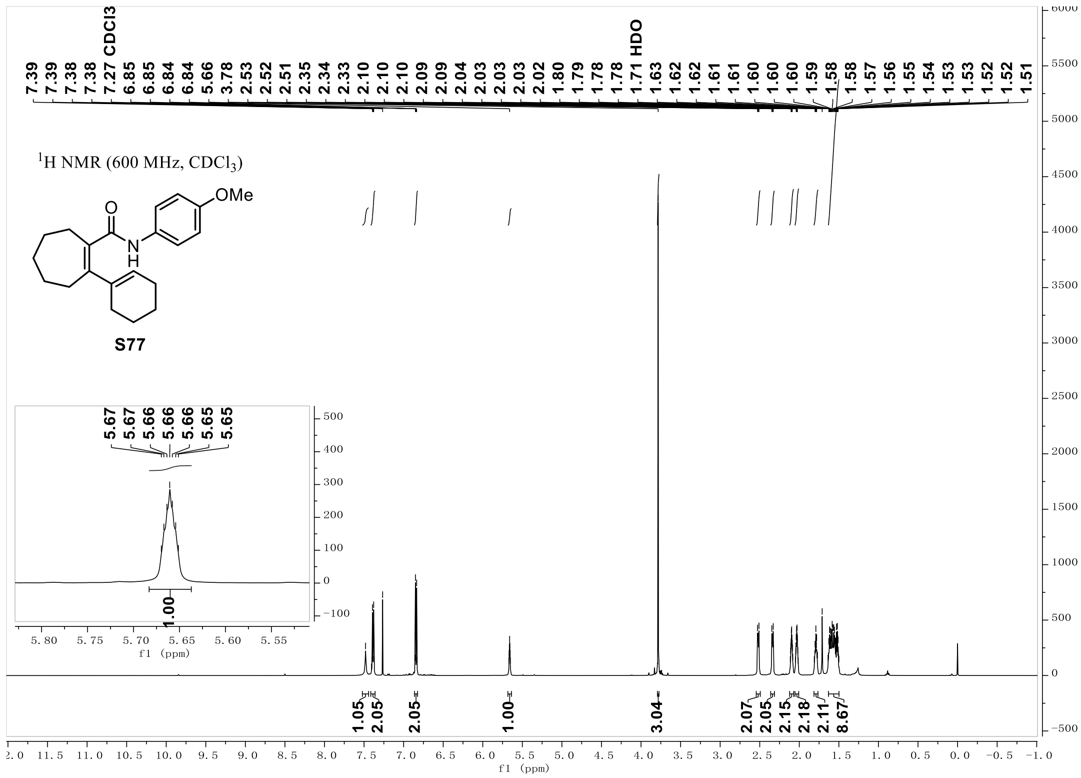

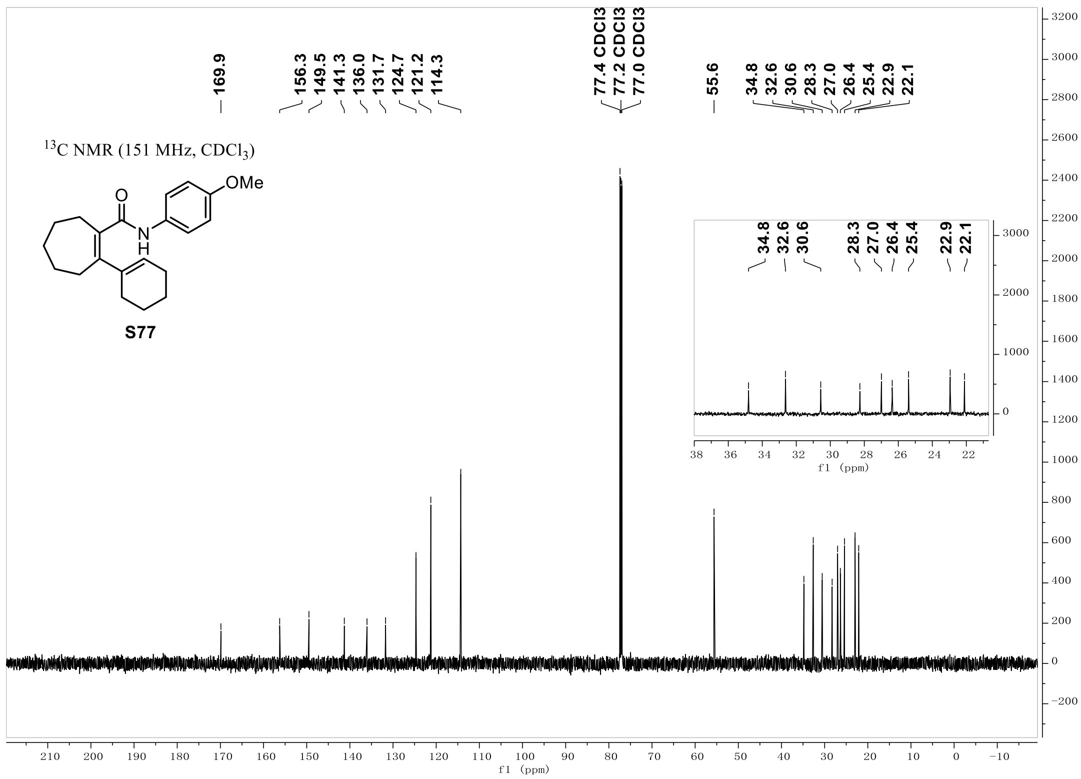

Supplementary Figure 104. ^{1}H NMR and ^{13}C NMR spectra of compound S77.

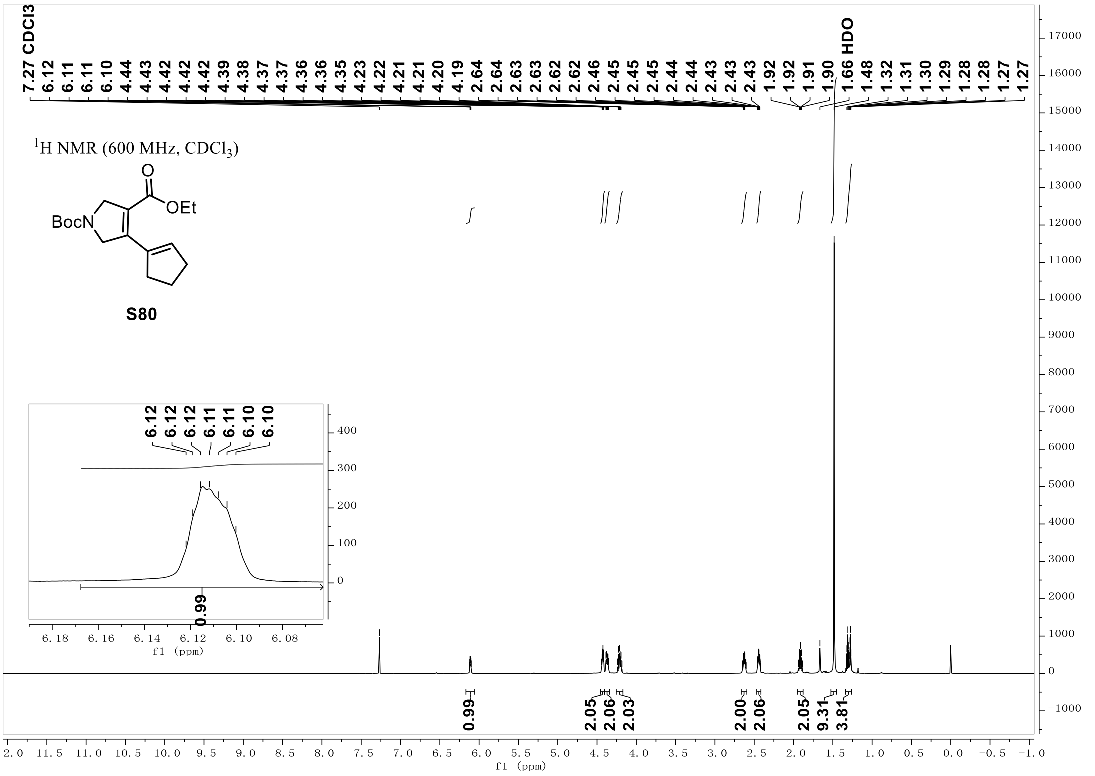

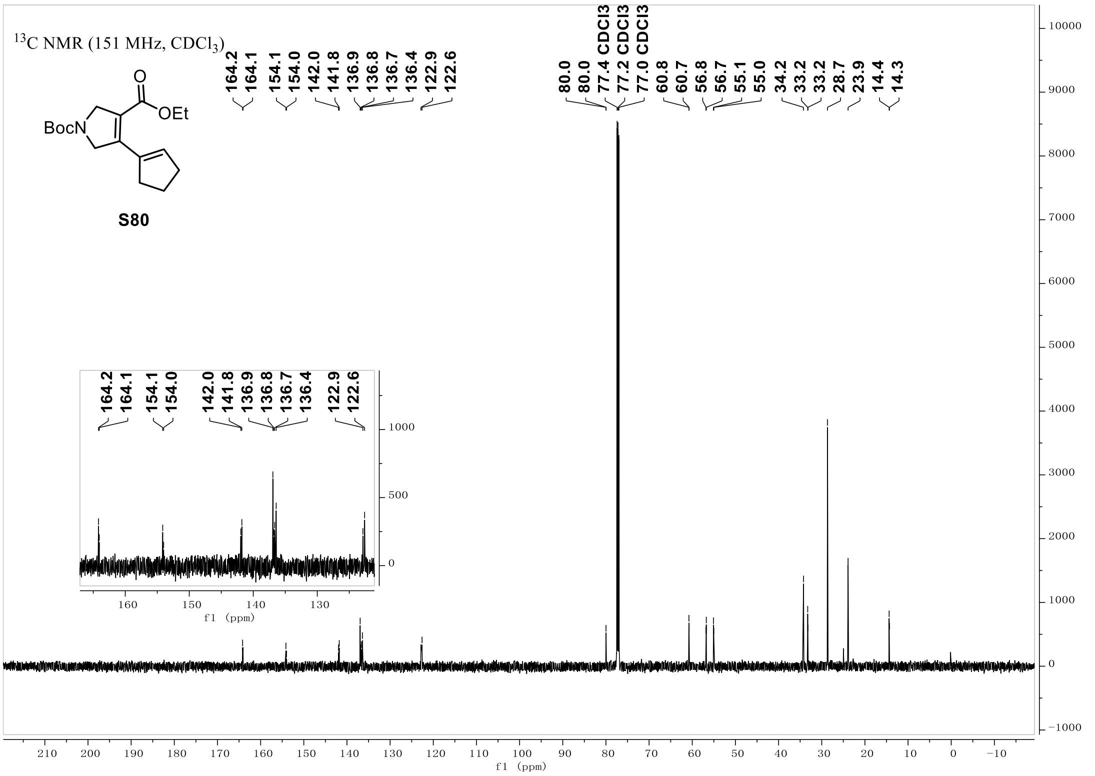

Supplementary Figure 105. ^{1}H NMR and ^{13}C NMR spectra of compound S80.

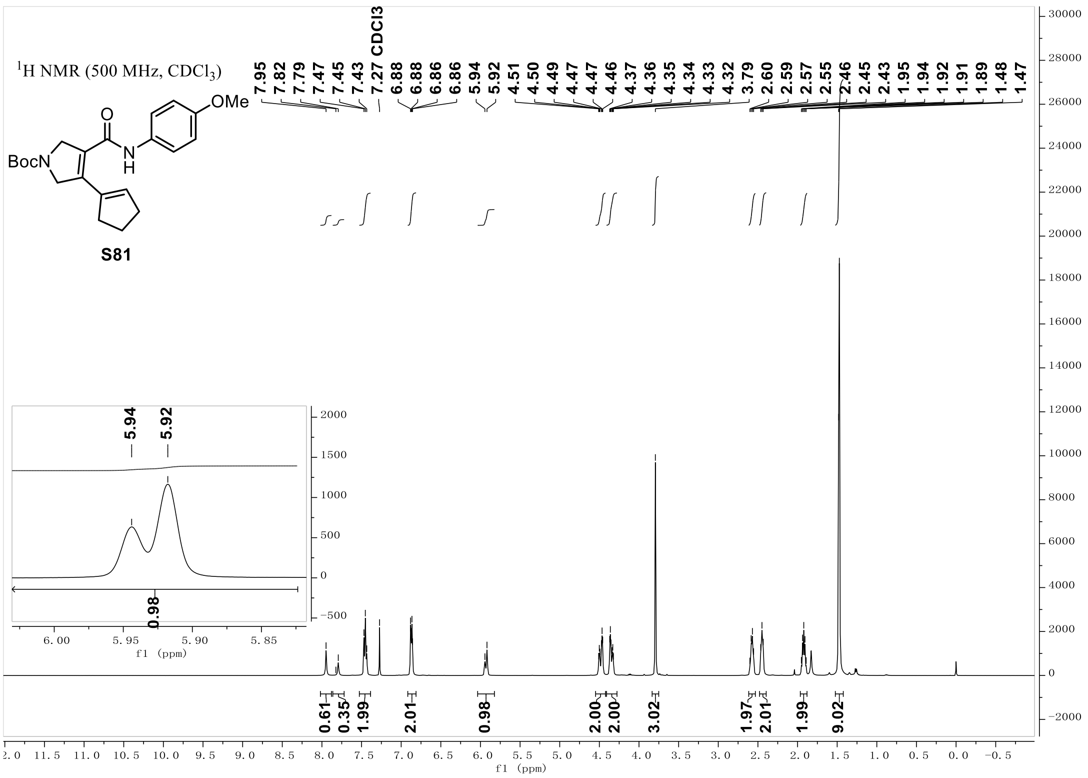

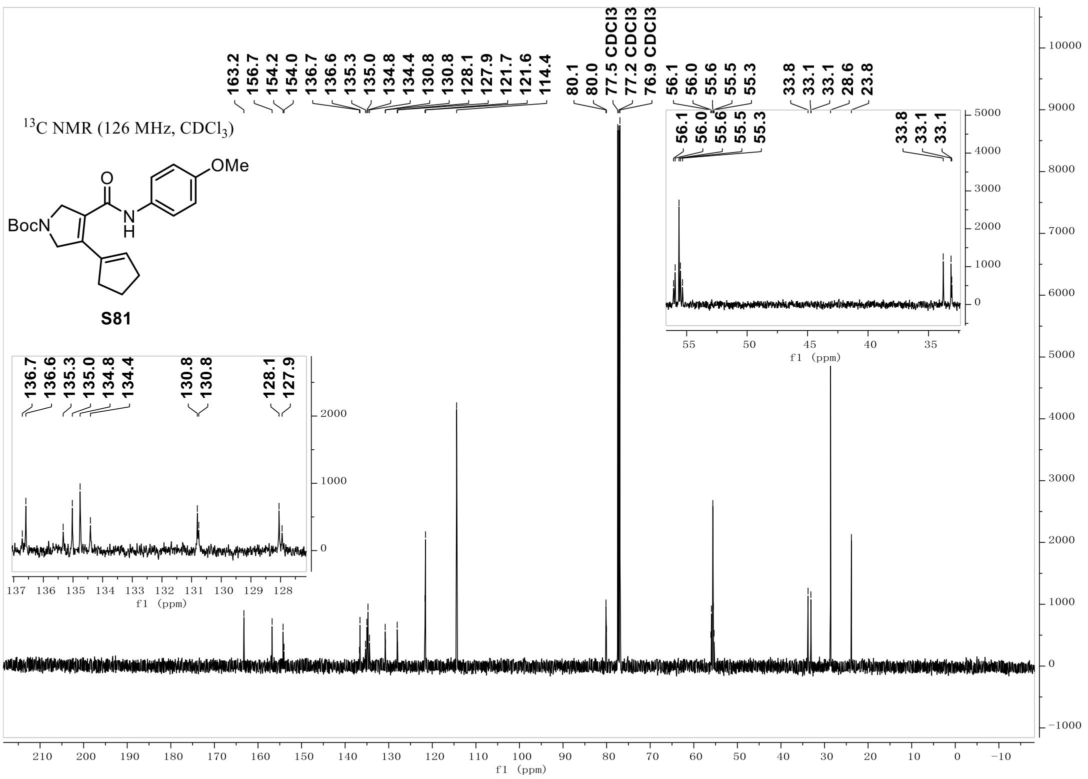

Supplementary Figure 106. ^{1}H NMR and ^{13}C NMR spectra of compound S81.

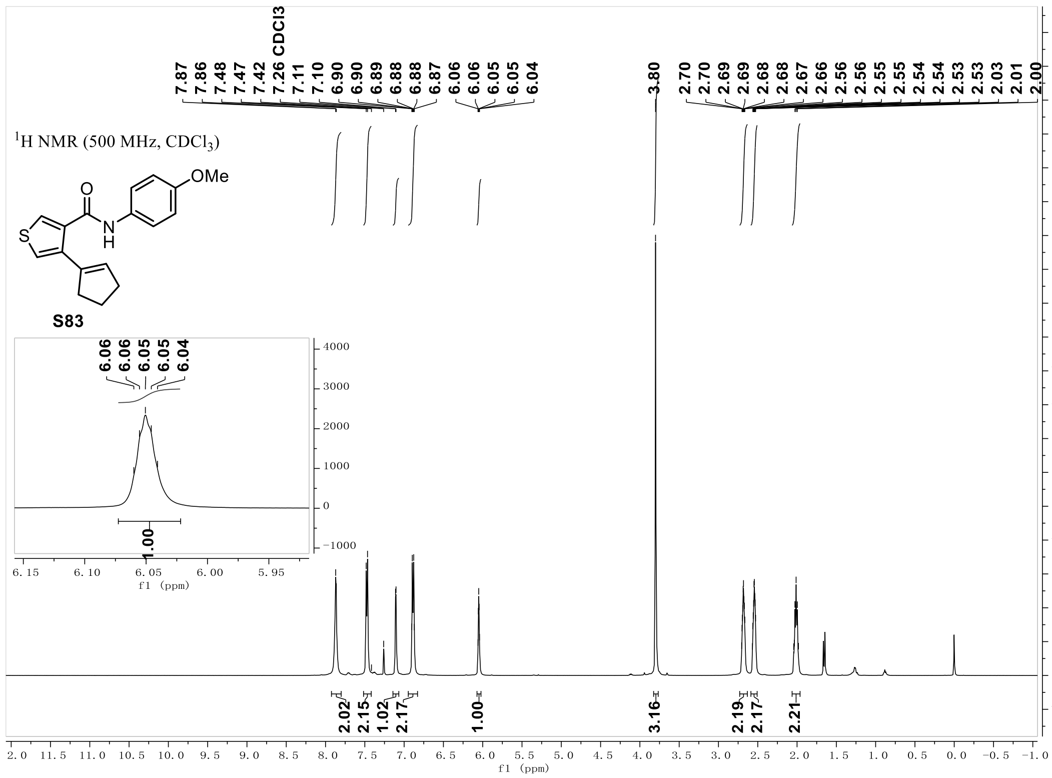

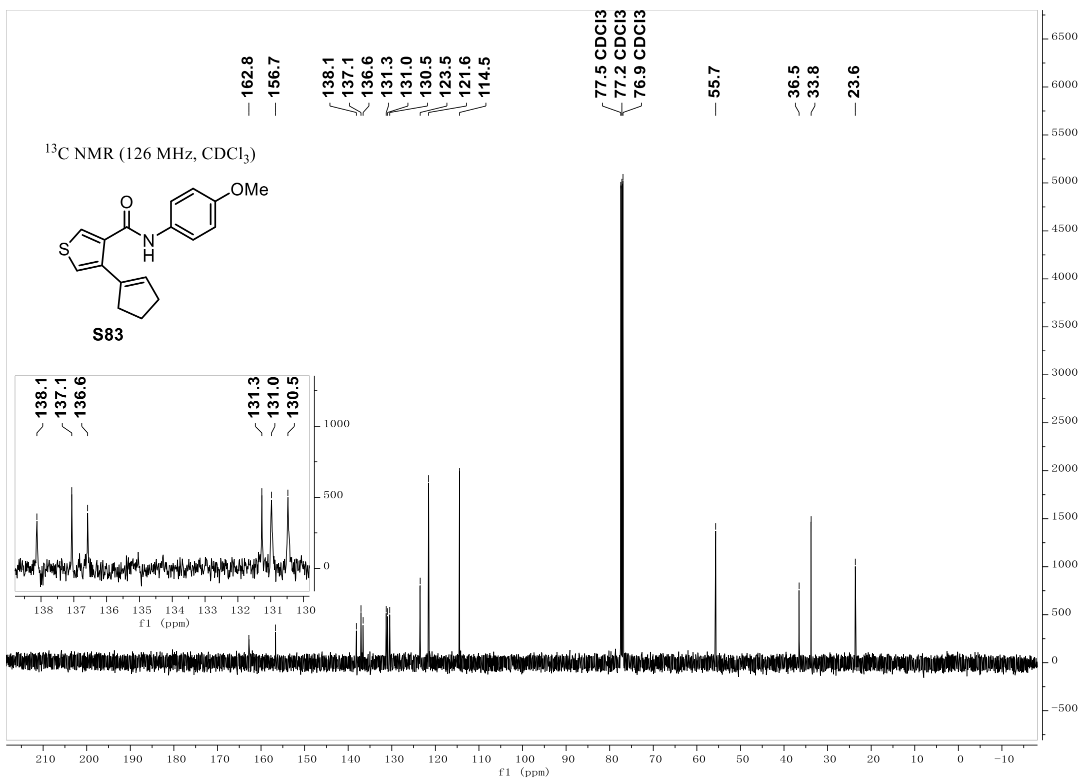

Supplementary Figure 107. ^{1}H NMR and ^{13}C NMR spectra of compound S83.

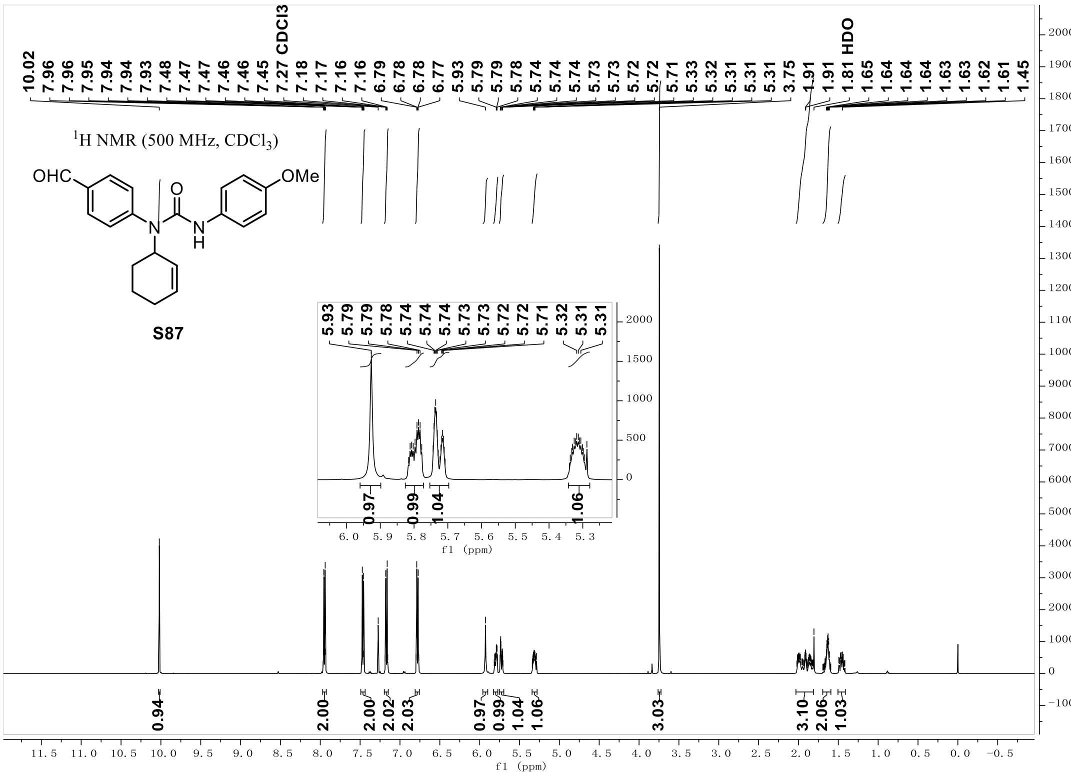

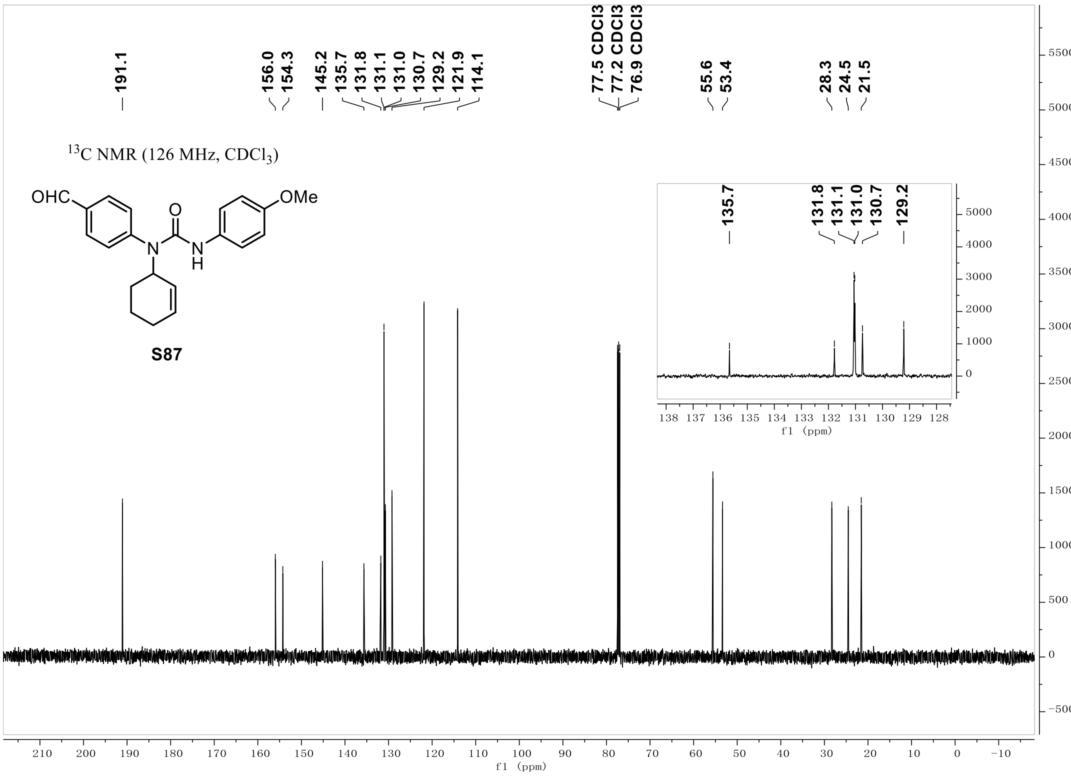

Supplementary Figure 108. ^{1}H NMR and ^{13}C NMR spectra of compound S87.

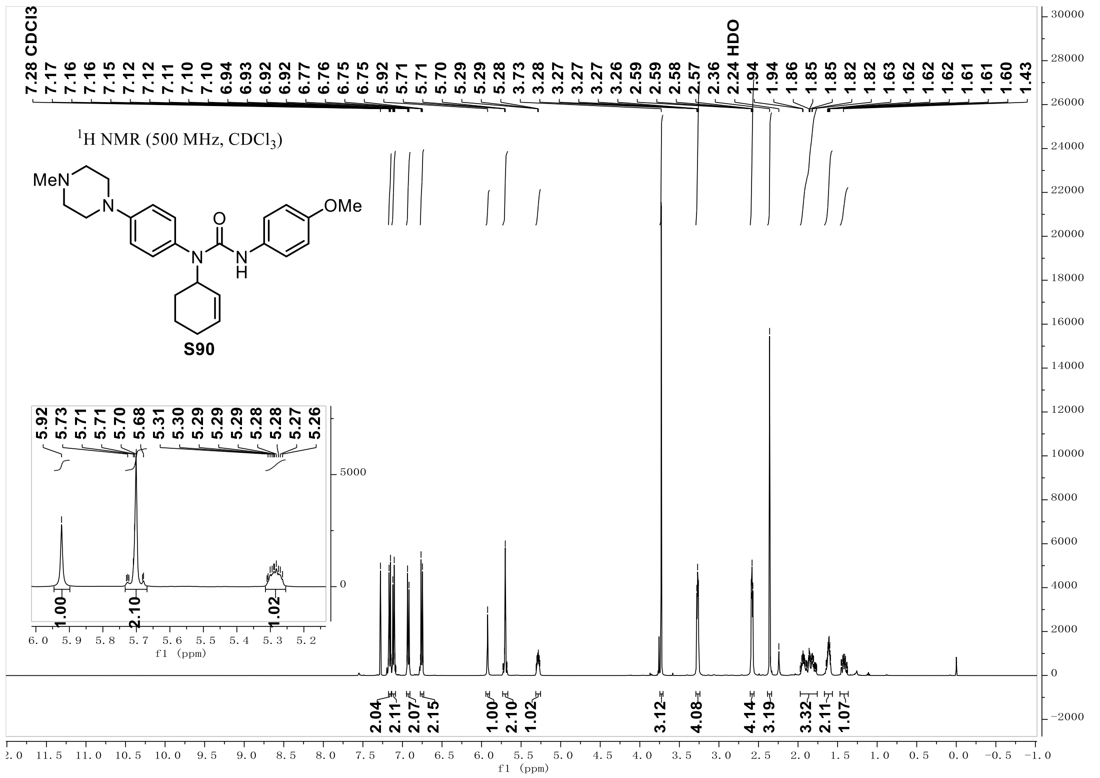

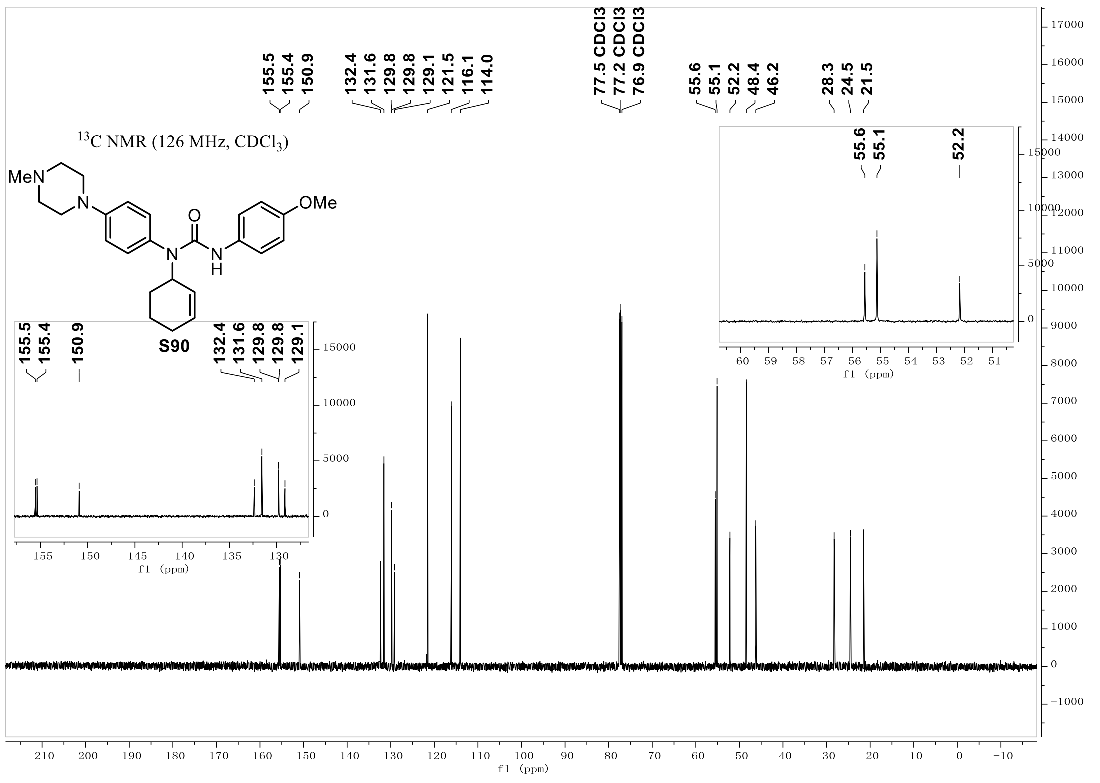

Supplementary Figure 109. ^{1}H NMR and ^{13}C NMR spectra of compound S90.

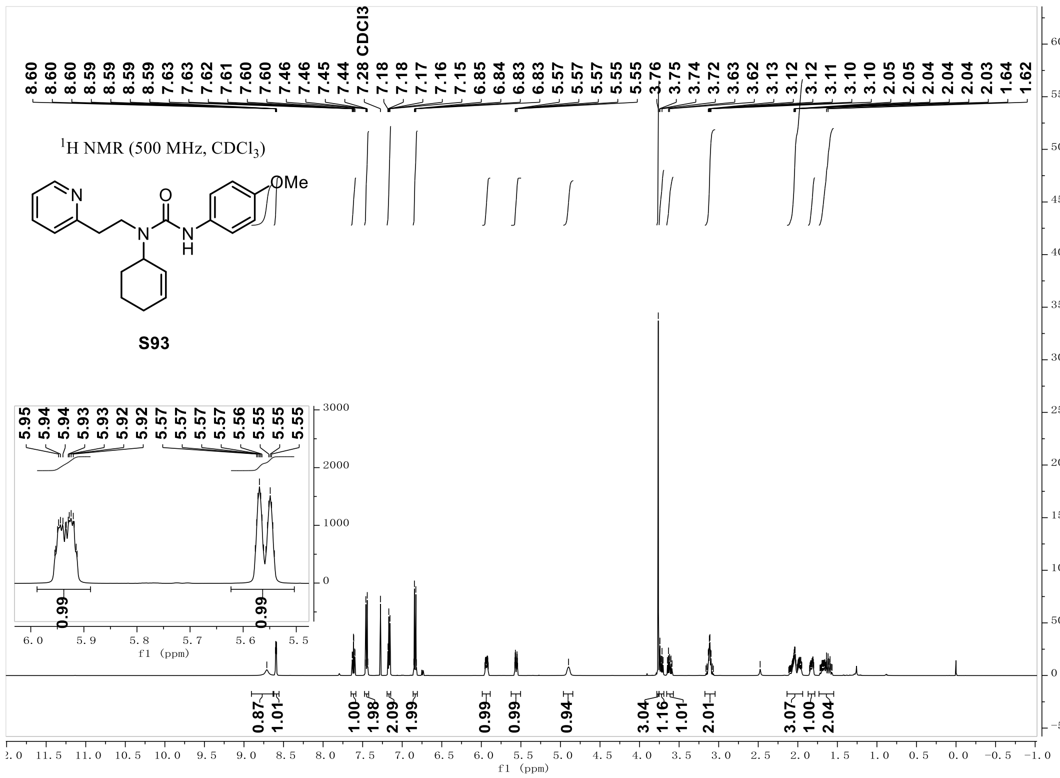

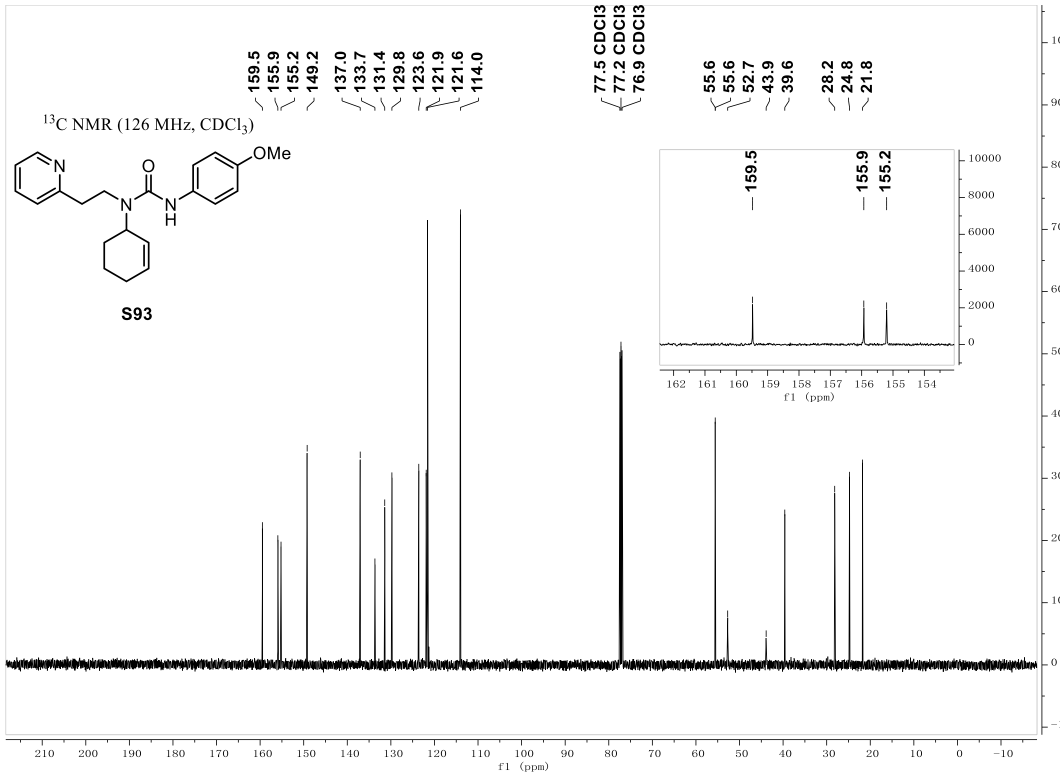

Supplementary Figure 110. ^{1}H NMR and ^{13}C NMR spectra of compound S93.

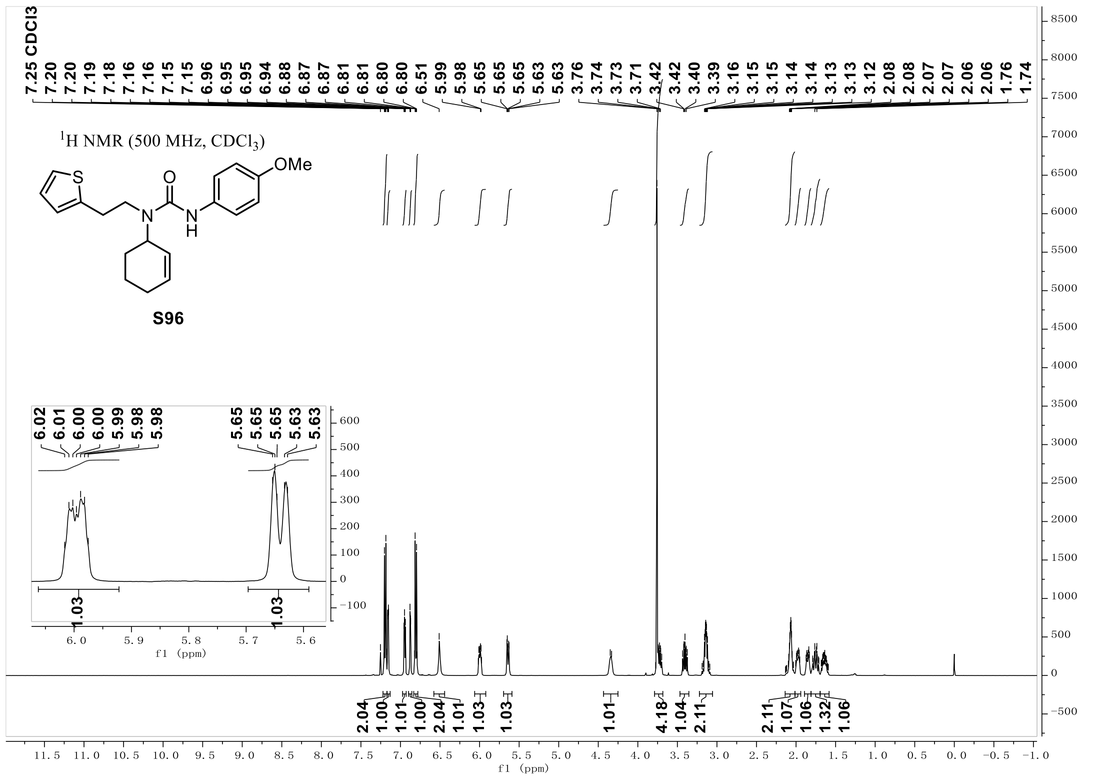

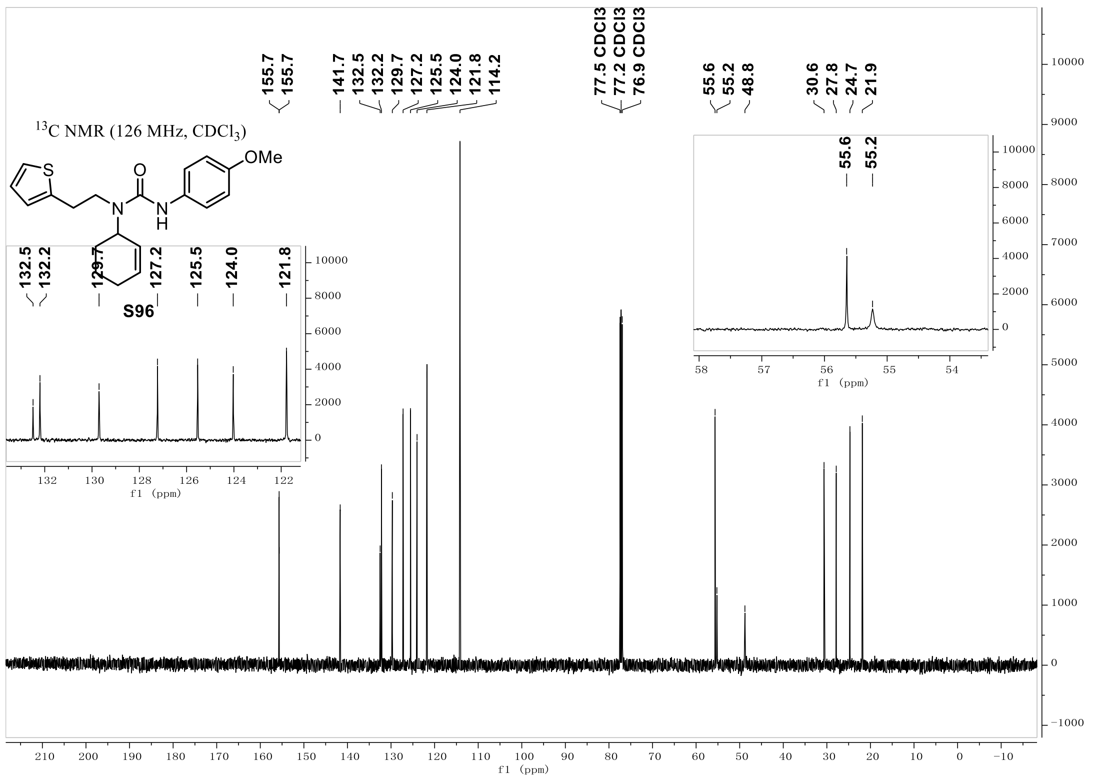

Supplementary Figure 111. ^{1}H NMR and ^{13}C NMR spectra of compound S96.

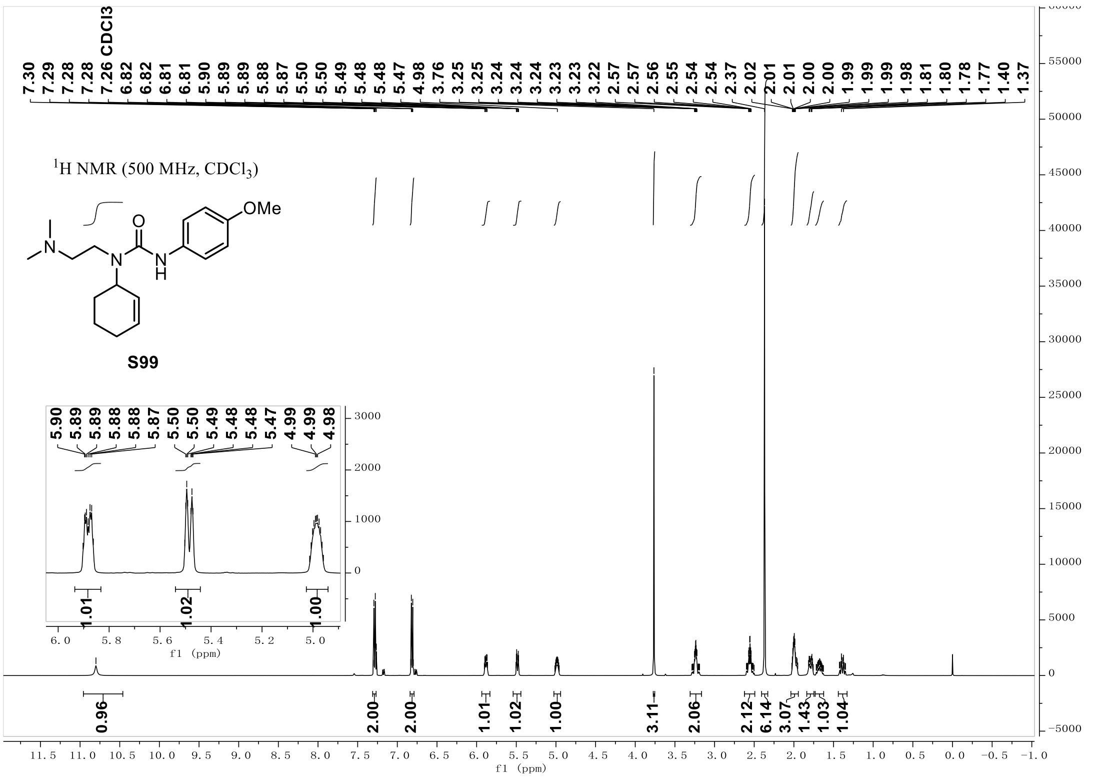

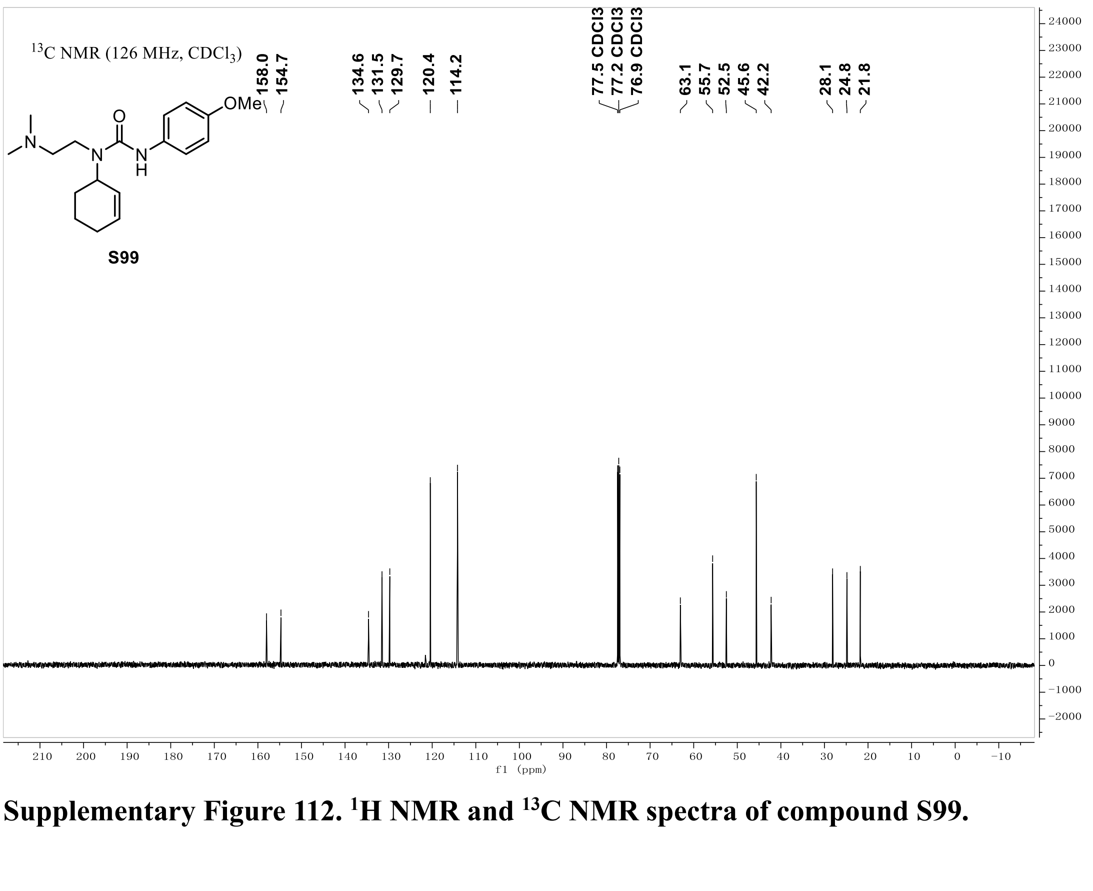

Supplementary Figure 112. ^{1}H NMR and ^{13}C NMR spectra of compound S99.

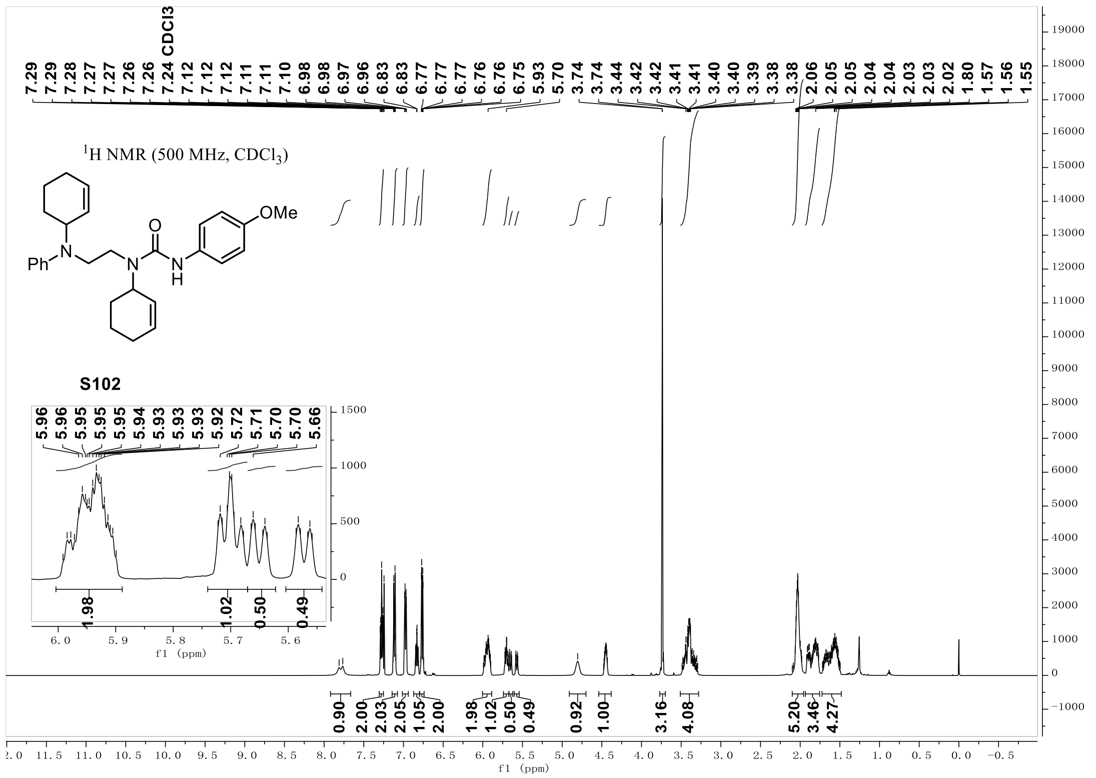

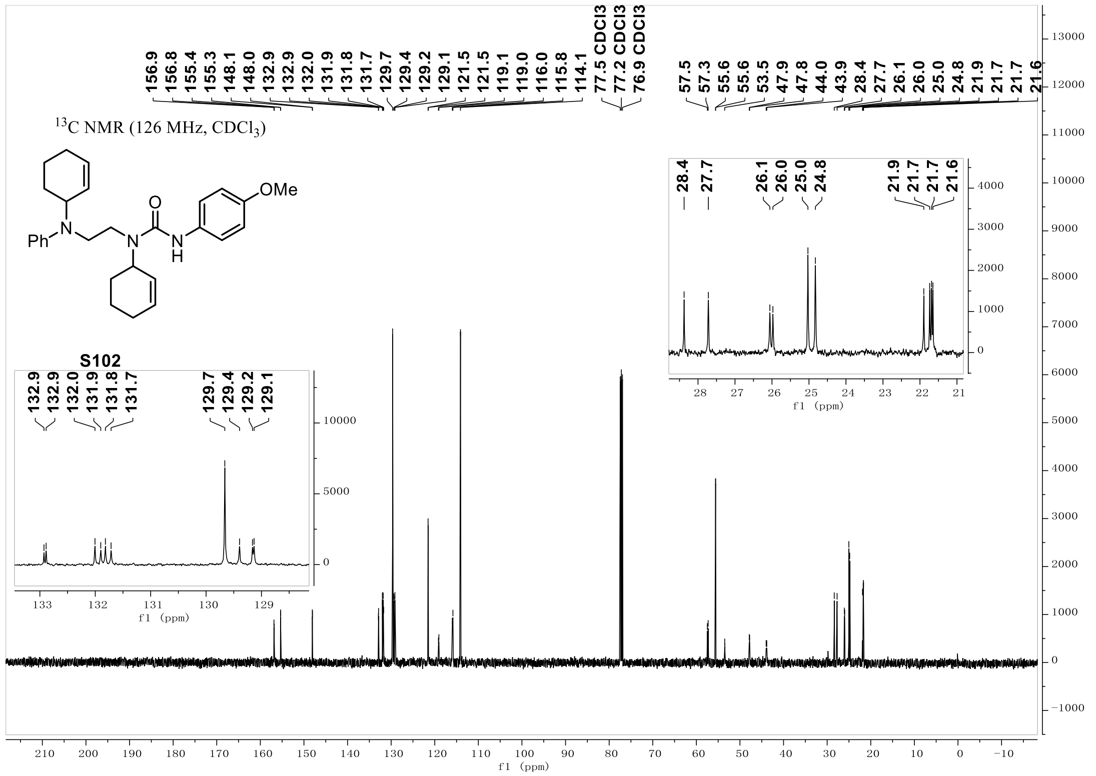

Supplementary Figure 113. ^{1}H NMR and ^{13}C NMR spectra of compound S102. The product was isolated as a 1:1 mixture of diastereisomers.

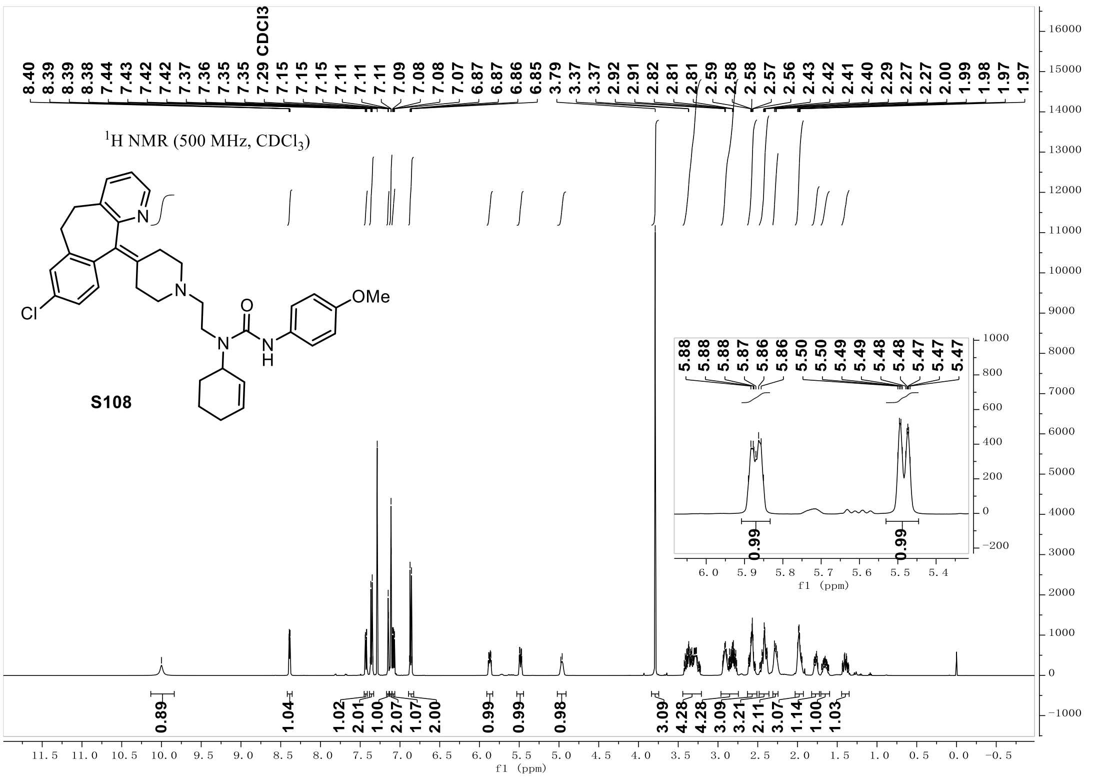

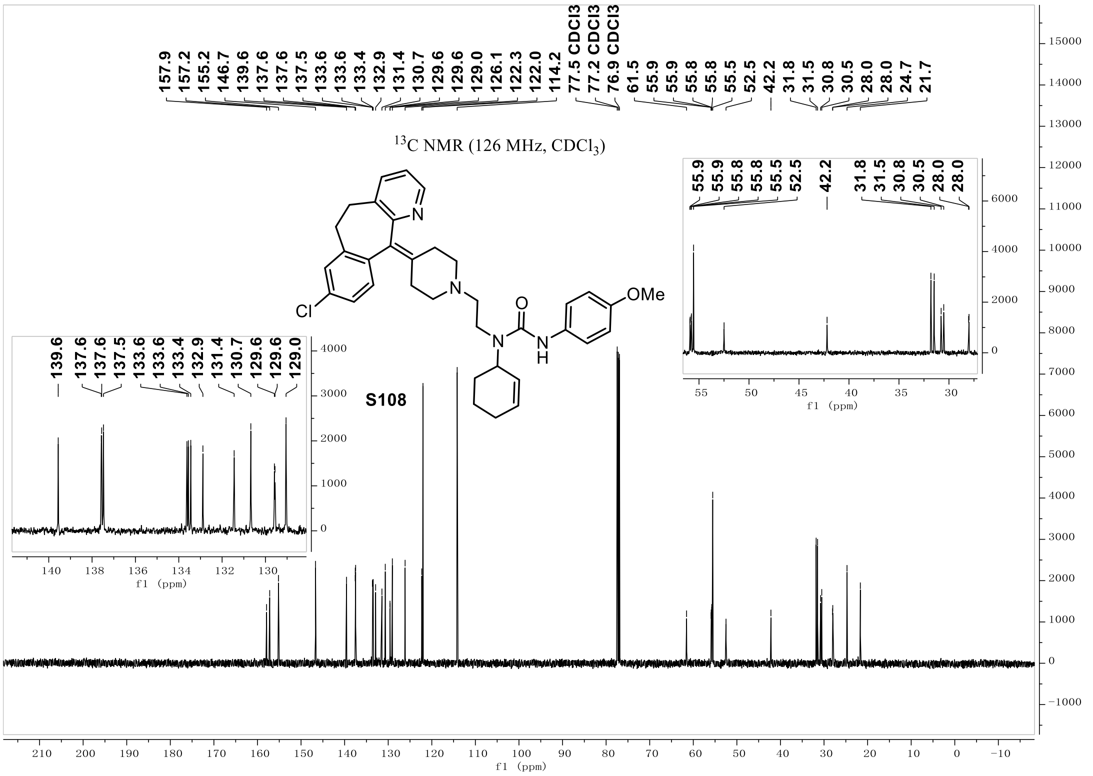

Supplementary Figure 114. ^{1}H NMR and ^{13}C NMR spectra of compound S108. The product was isolated as a 1:1 mixture of diastereomers.

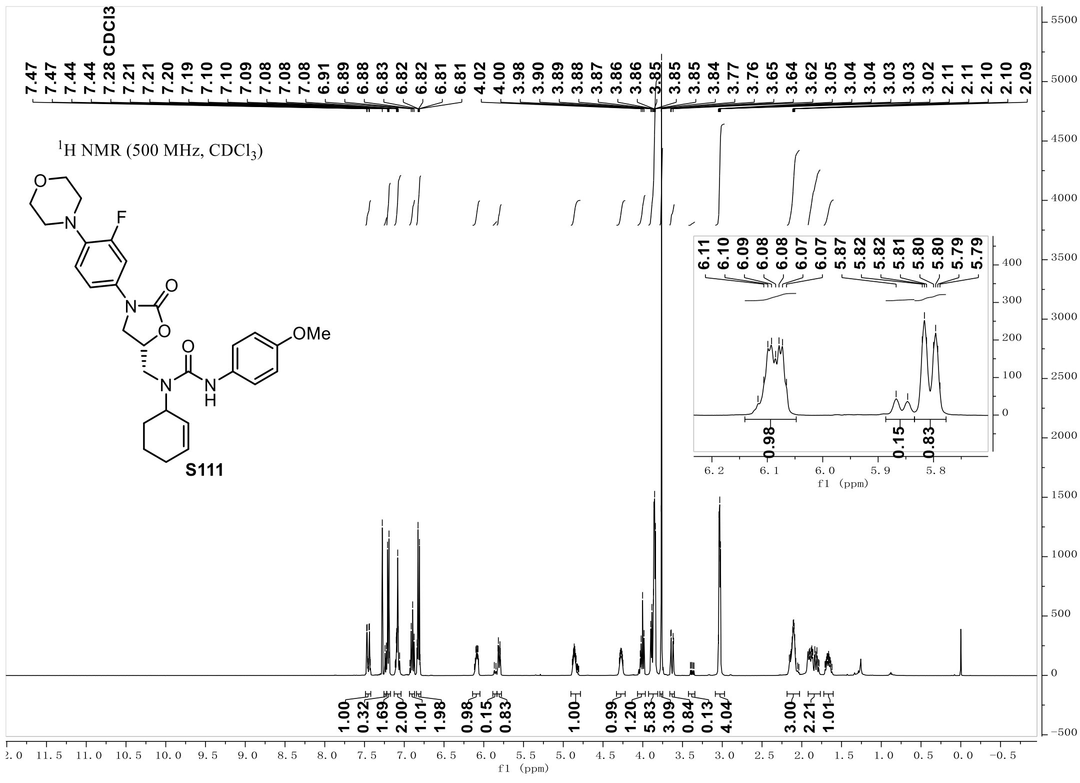

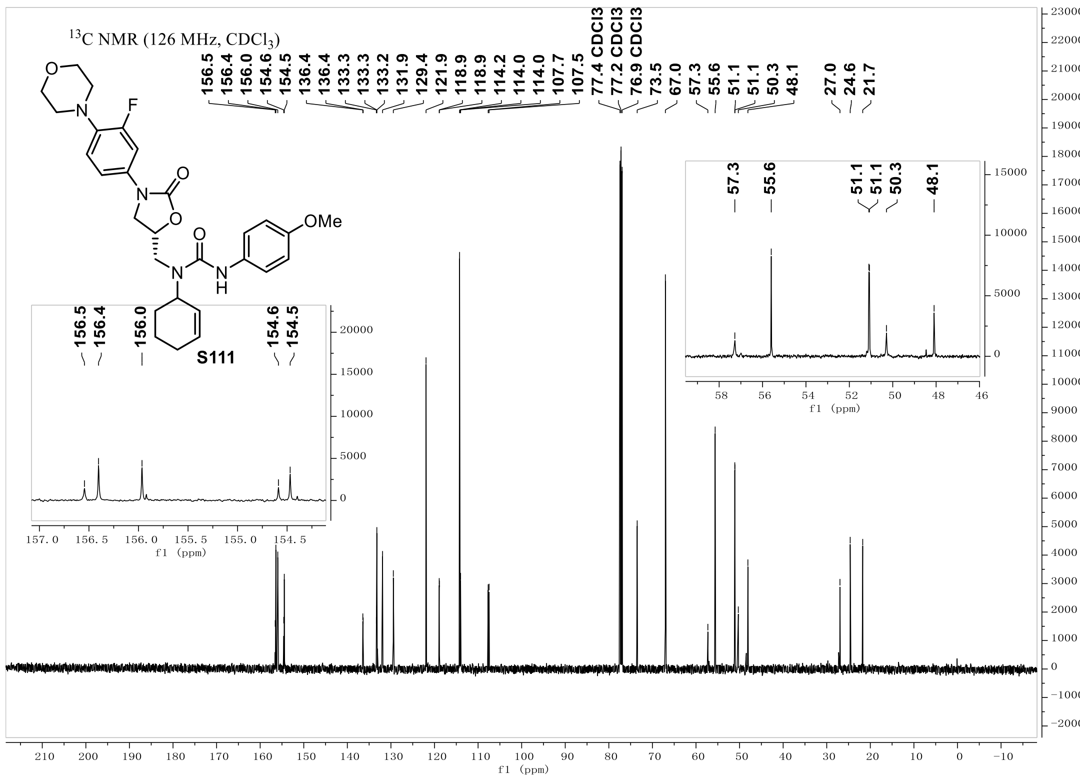

Supplementary Figure 115. ^{1}H NMR and ^{13}C NMR spectra of compound S111. The prouct was isolated as a 5.7:1 mixture of diastereomers.

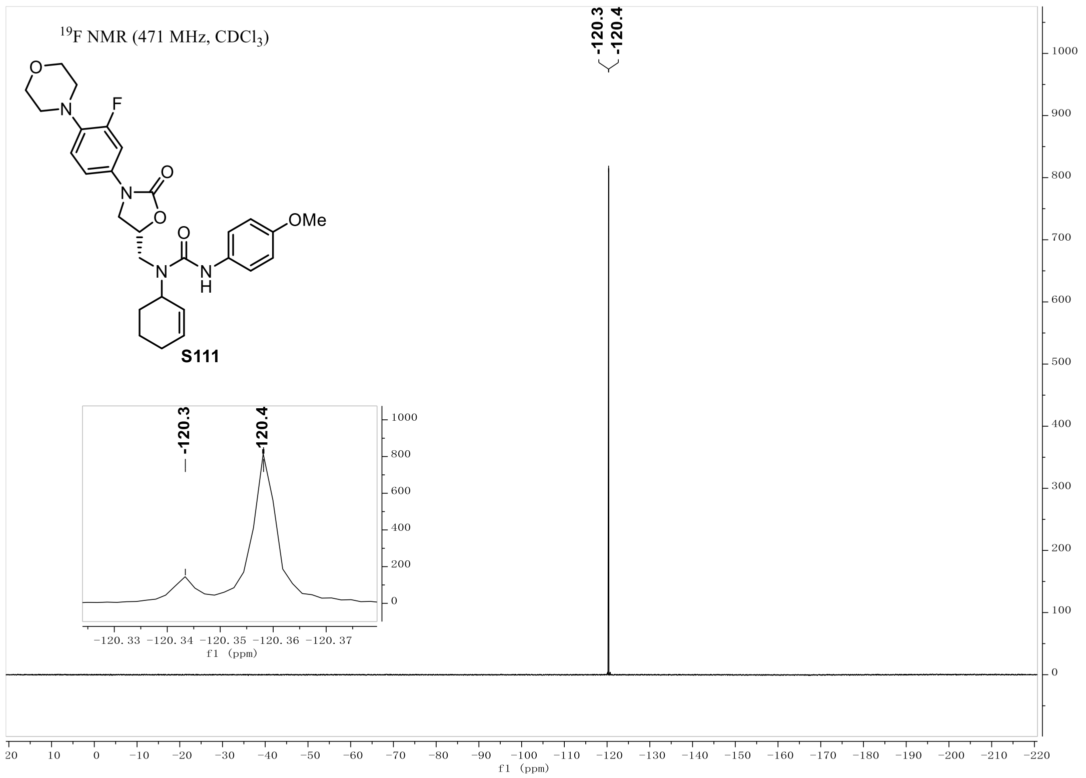

Supplementary Figure 116. ^{19}F NMR spectra of compound S111. The prouct was isolated as a 5.7:1 mixture of diastereomers.

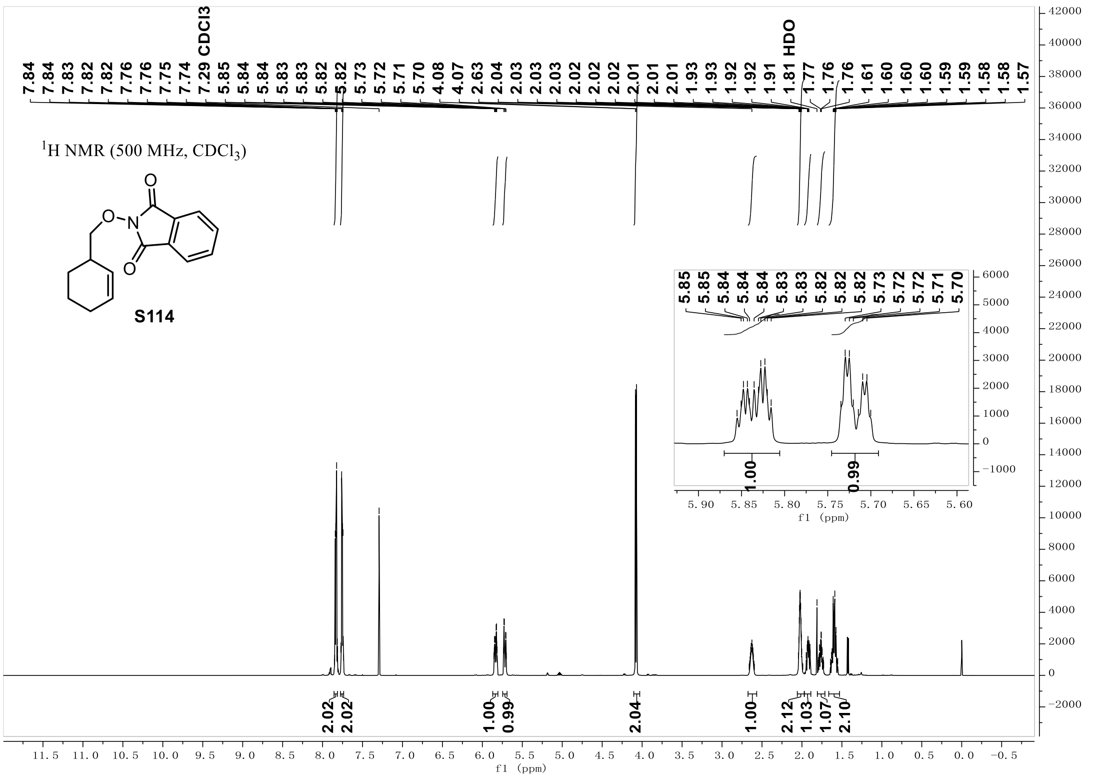

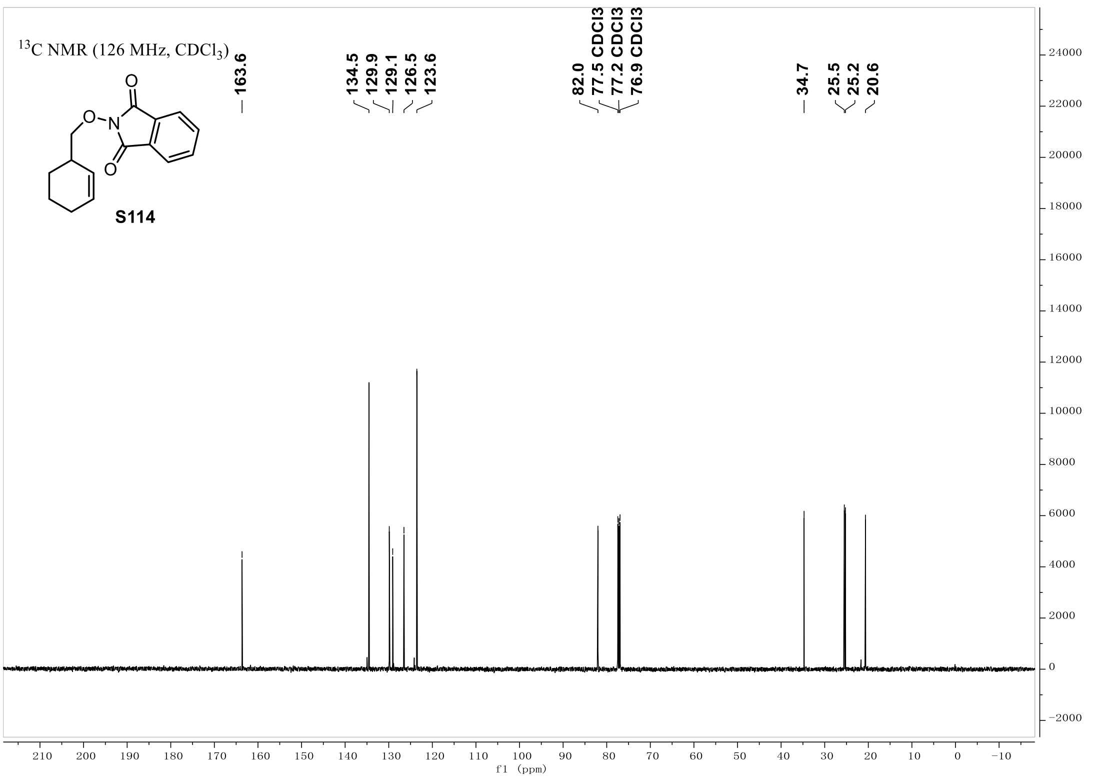

Supplementary Figure 117. ^{1}H NMR and ^{13}C NMR spectra of compound S114.

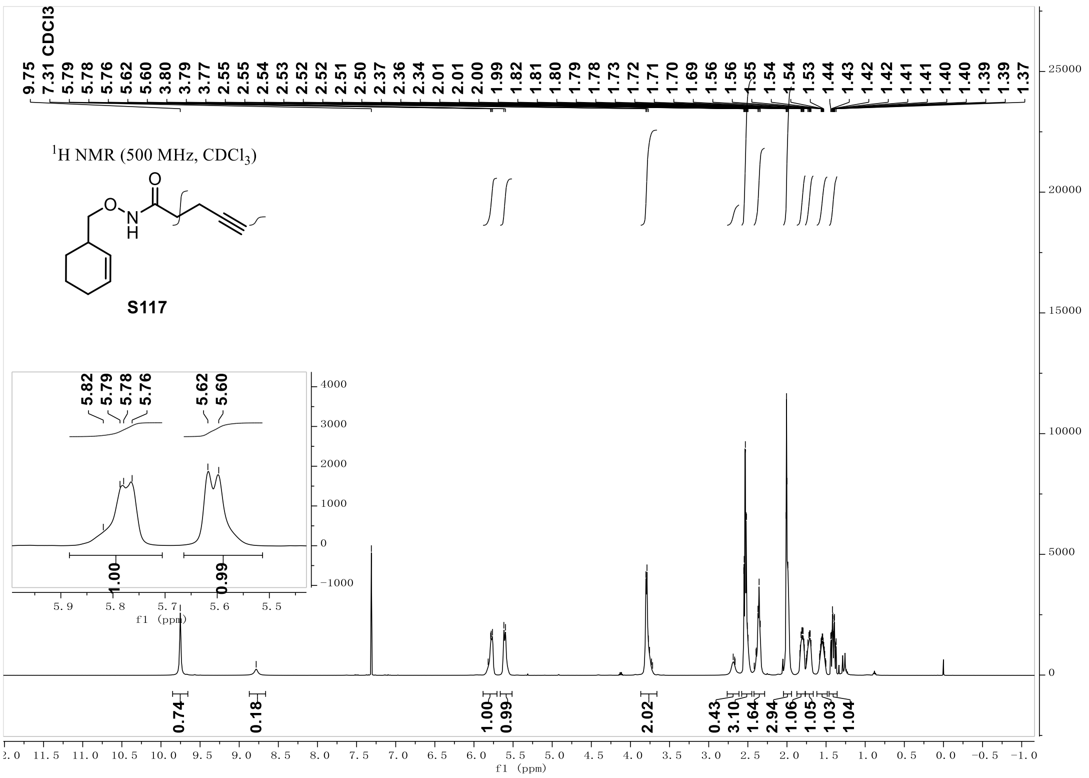

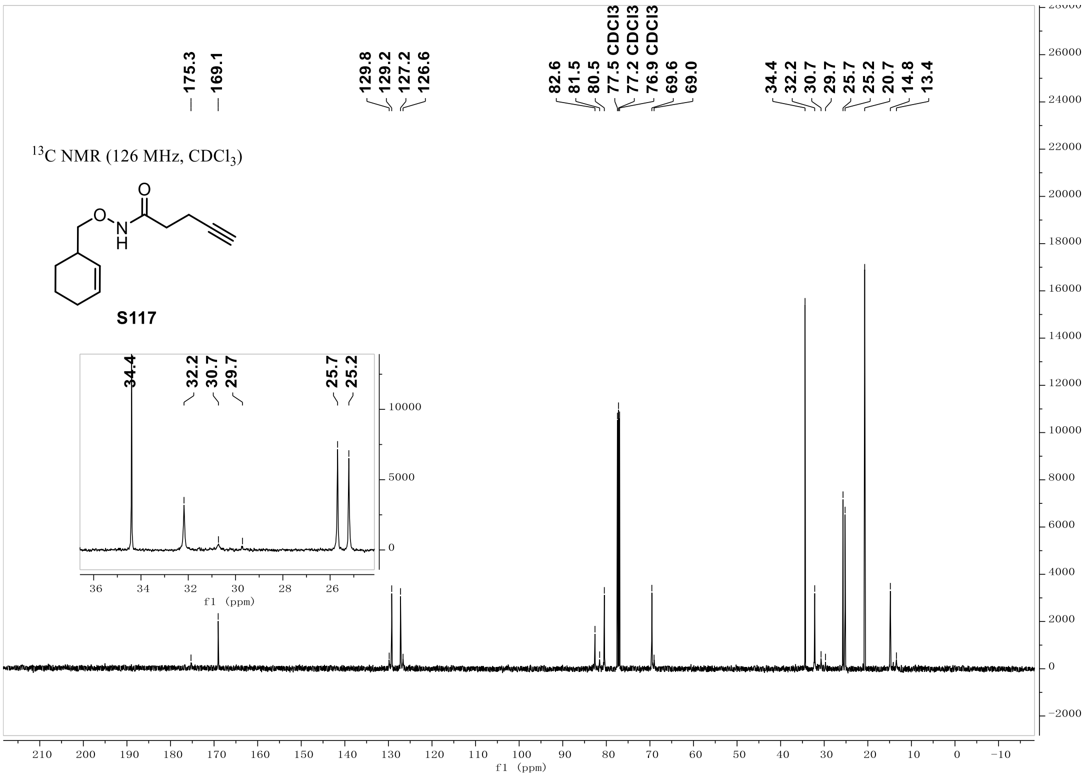

Supplementary Figure 118. ^{1}H NMR and ^{13}C NMR spectra of compound S117. The product was isolated as a 4:1 mixture of rotary isomers.

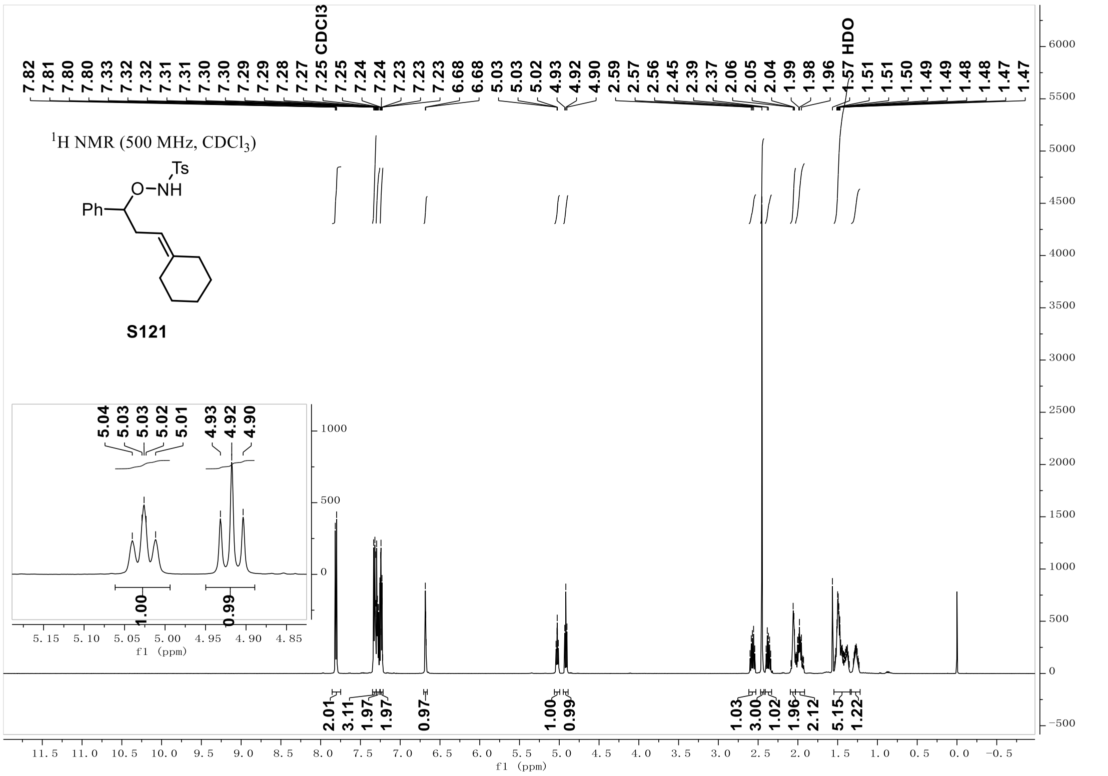

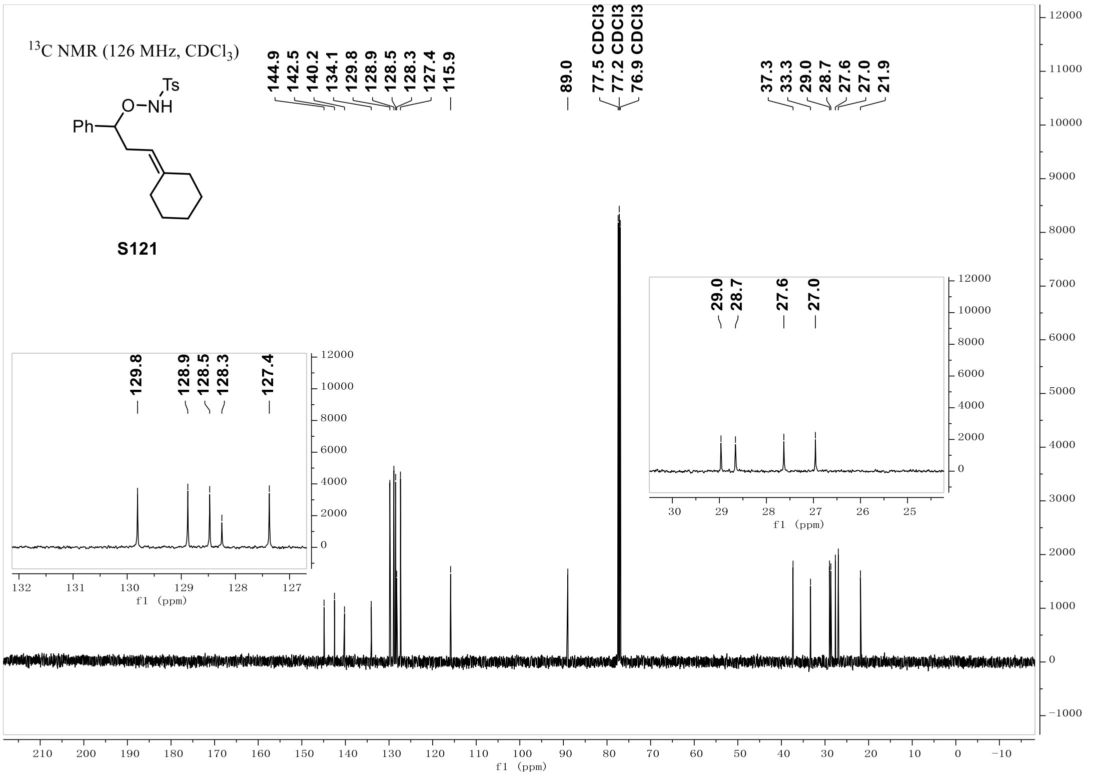

Supplementary Figure 119. ^{1}H NMR and ^{13}C NMR spectra of compound S121.

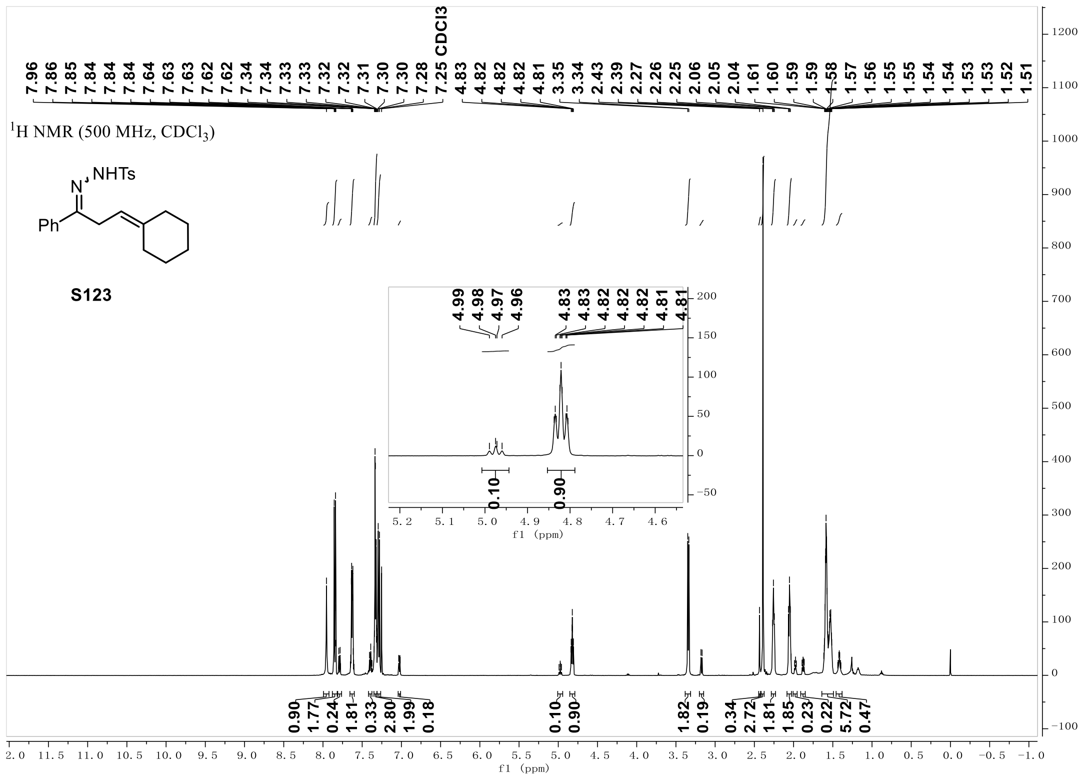

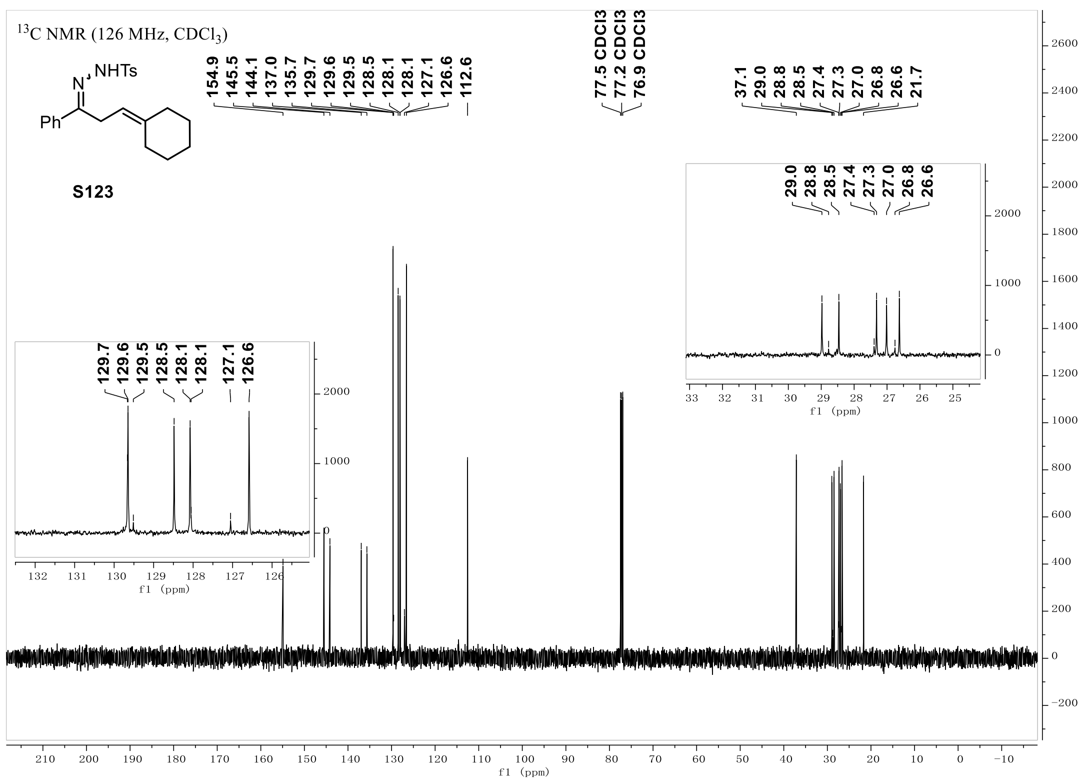

Supplementary Figure 120. ^{1}H NMR and ^{13}C NMR spectra of compound S123. The product was isolated a 1:9 mixture of *Z*/*E* isomers.

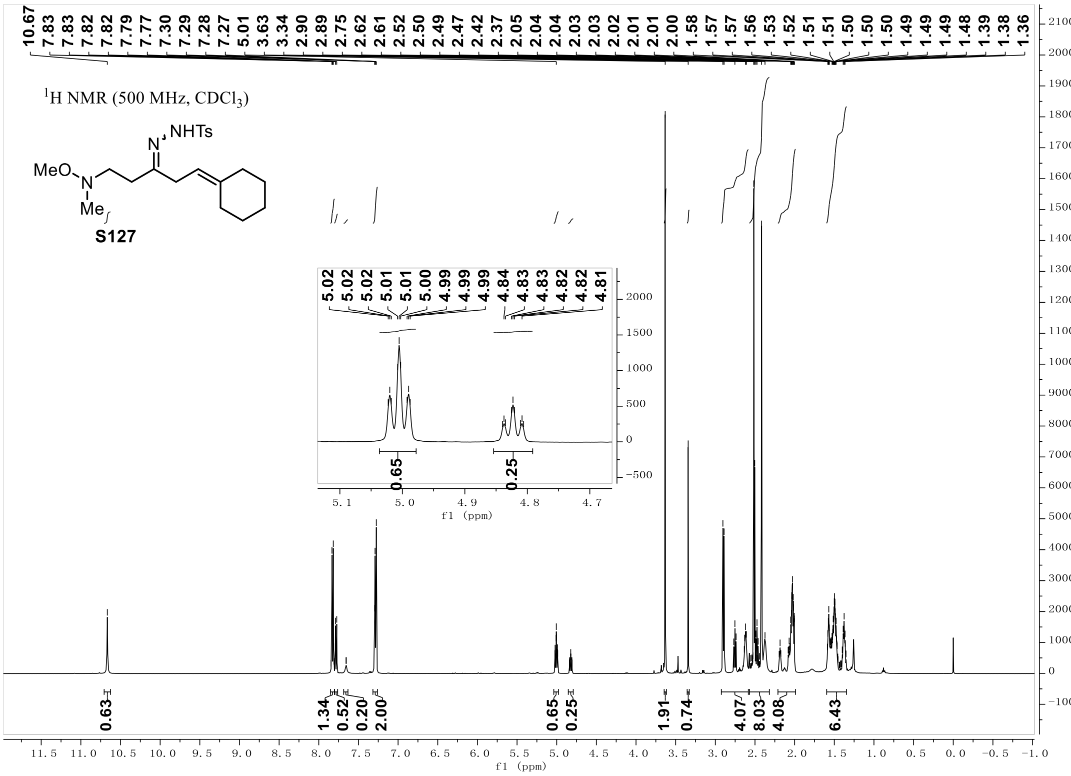

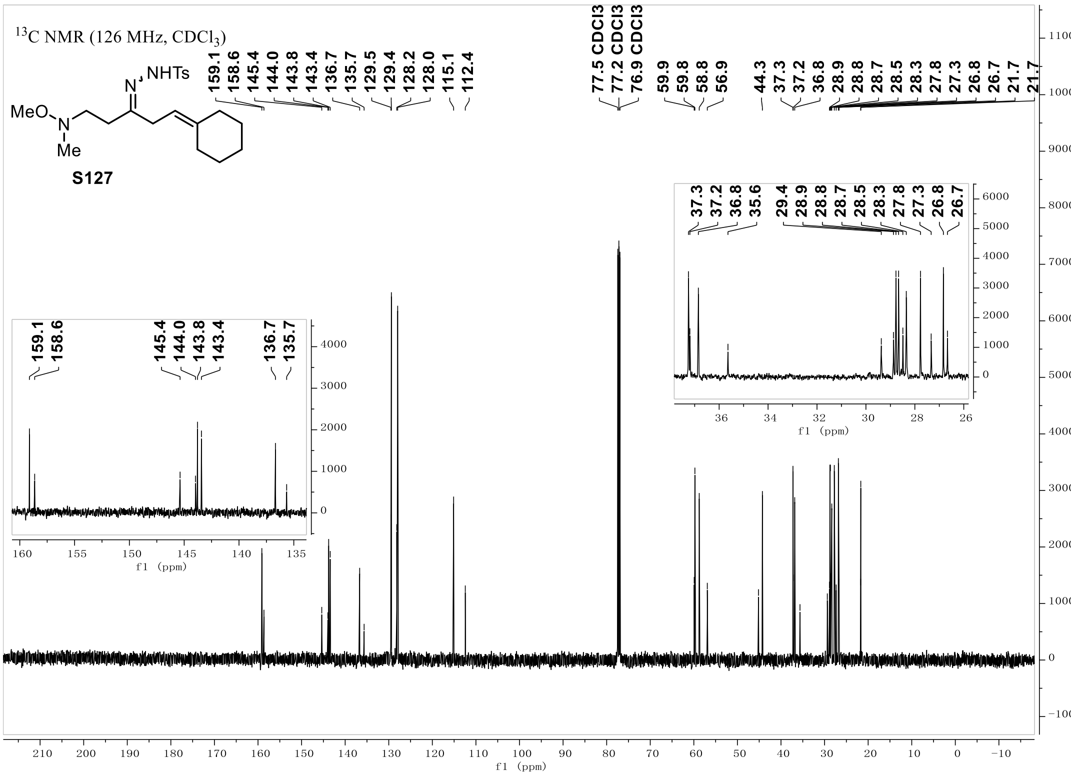

Supplementary Figure 121. ^{1}H NMR and ^{13}C NMR spectra of compound S127. The product was isolated as a 1:2.5 mixture of *Z*/*E* isomers.

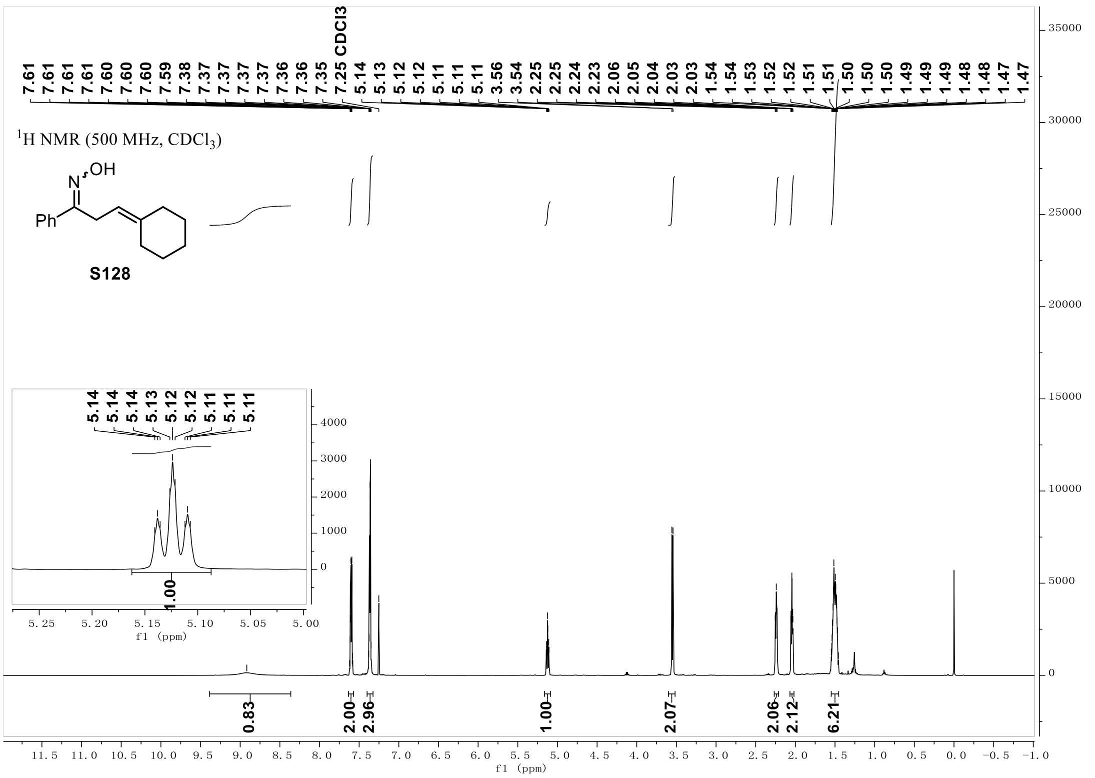

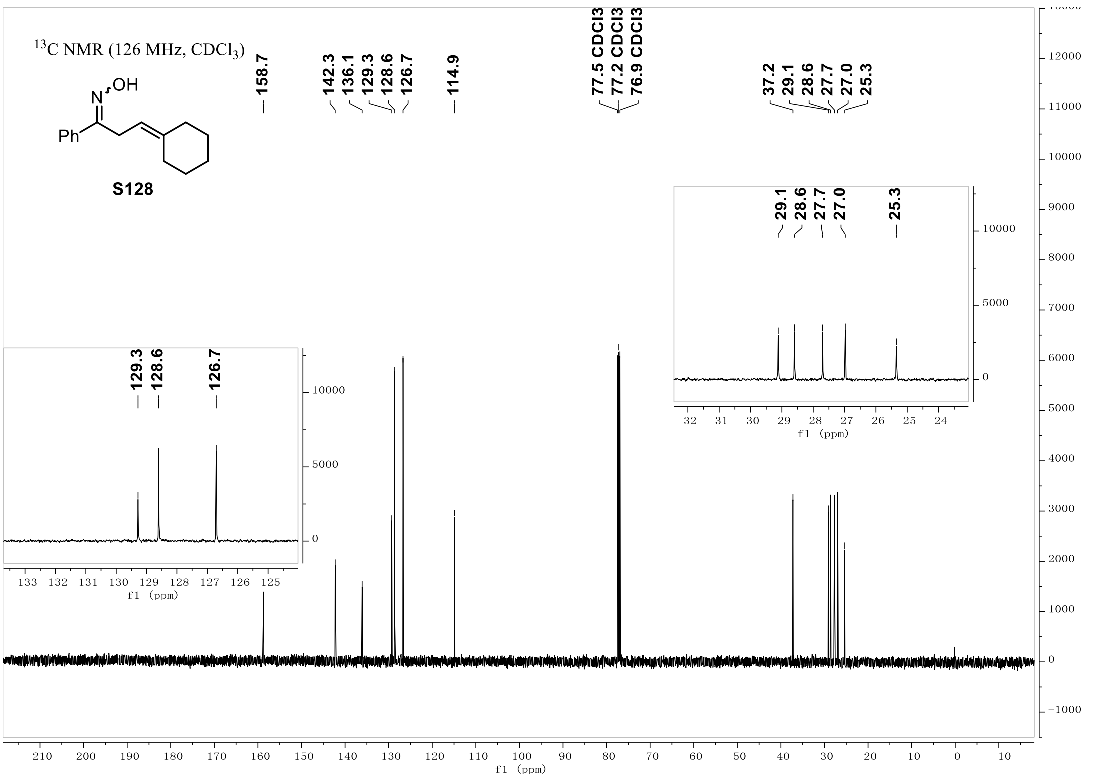

Supplementary Figure 122. ¹H NMR and ¹³C NMR spectra of compound S128.

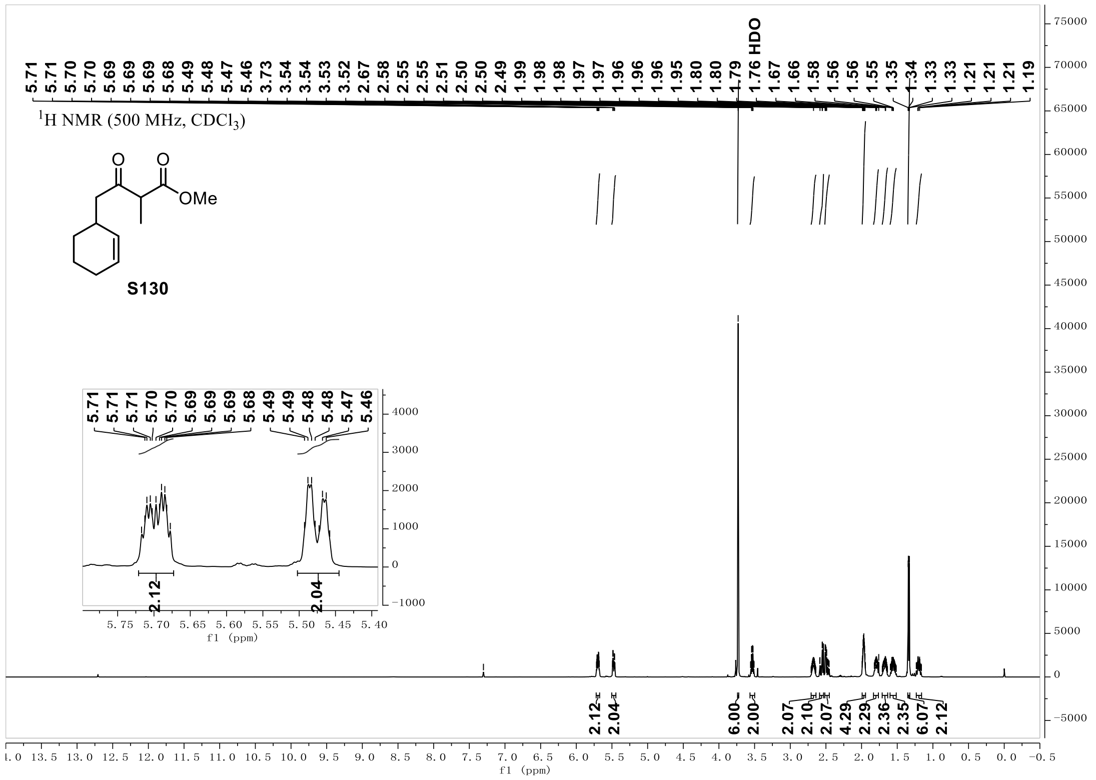

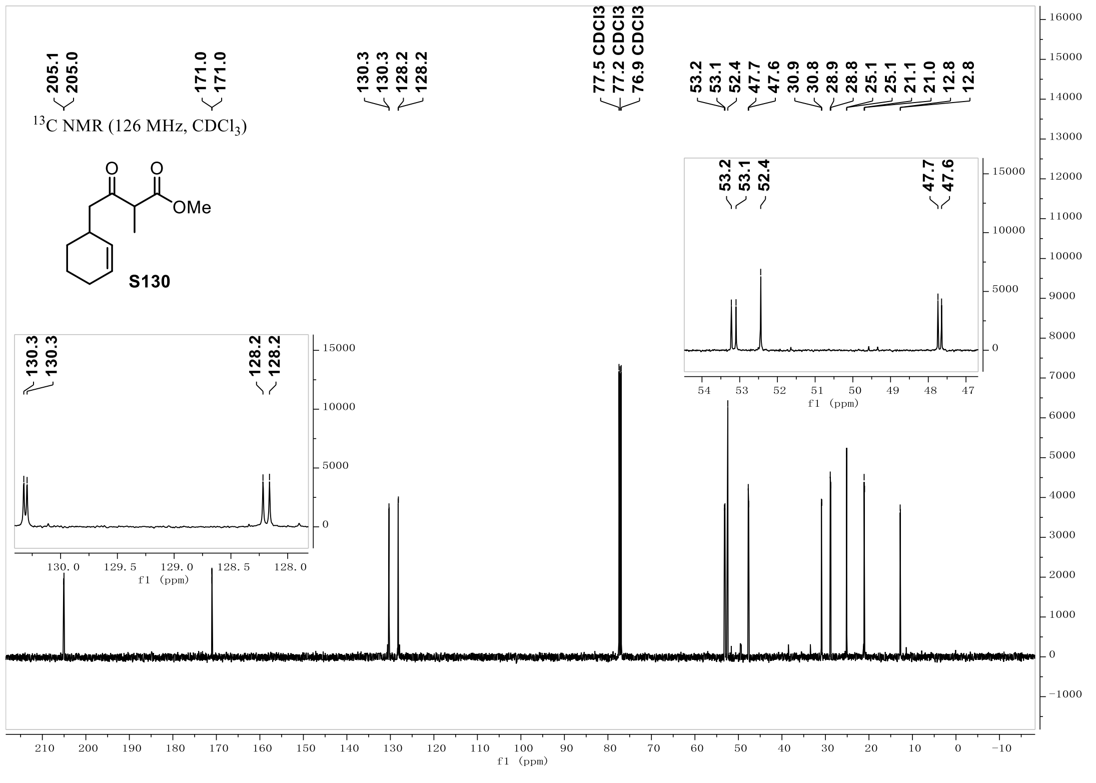

Supplementary Figure 123. ^{1}H NMR and ^{13}C NMR spectra of compound S130. The product was isolated as a 1:1 mixture of diastereomers and contained traces of enol form.

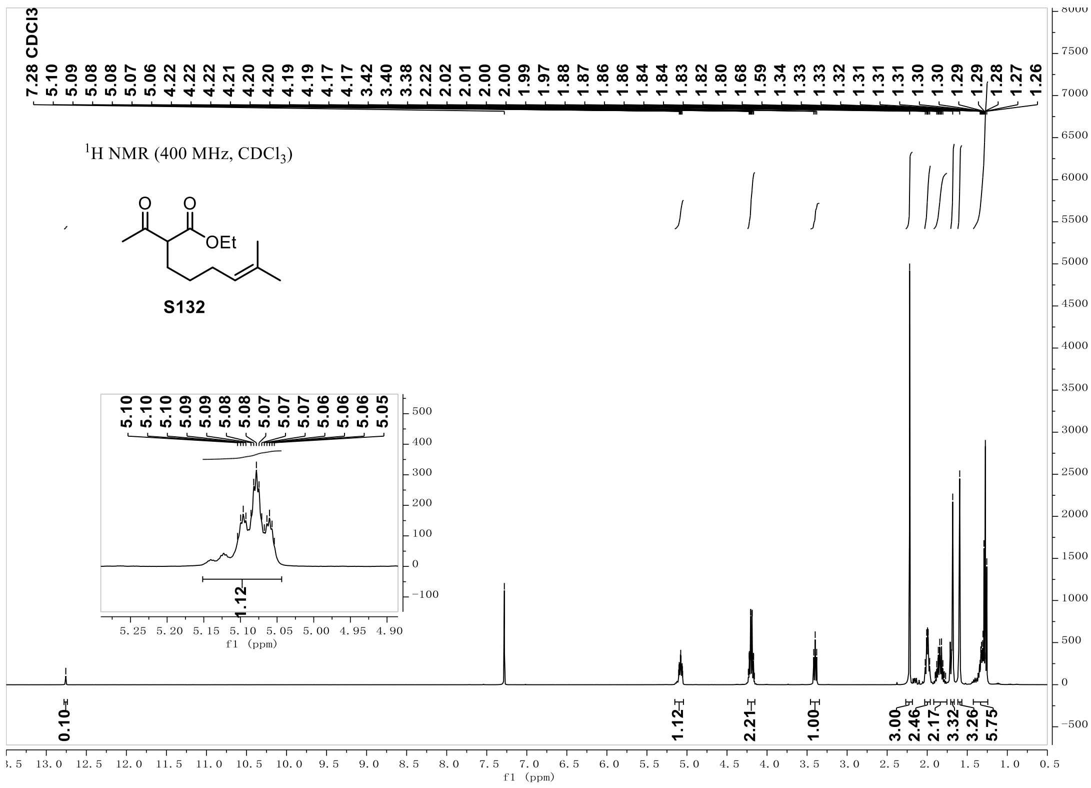

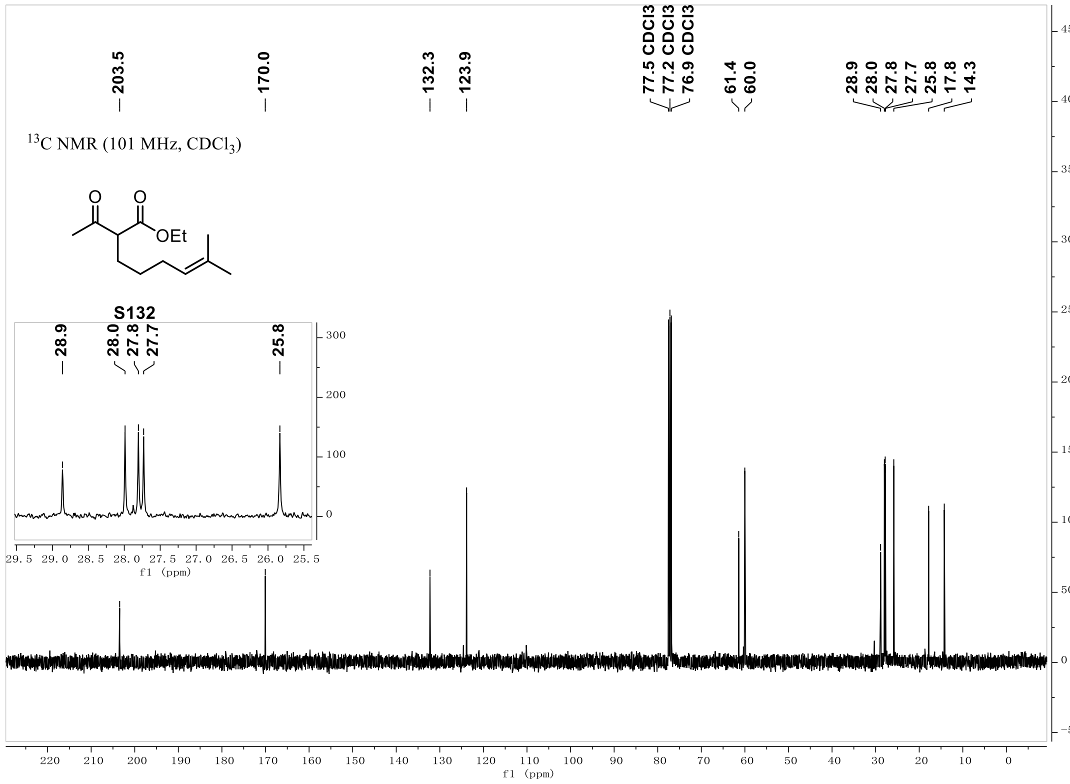

Supplementary Figure 124. ^{1}H NMR and ^{13}C NMR spectra of compound S132.

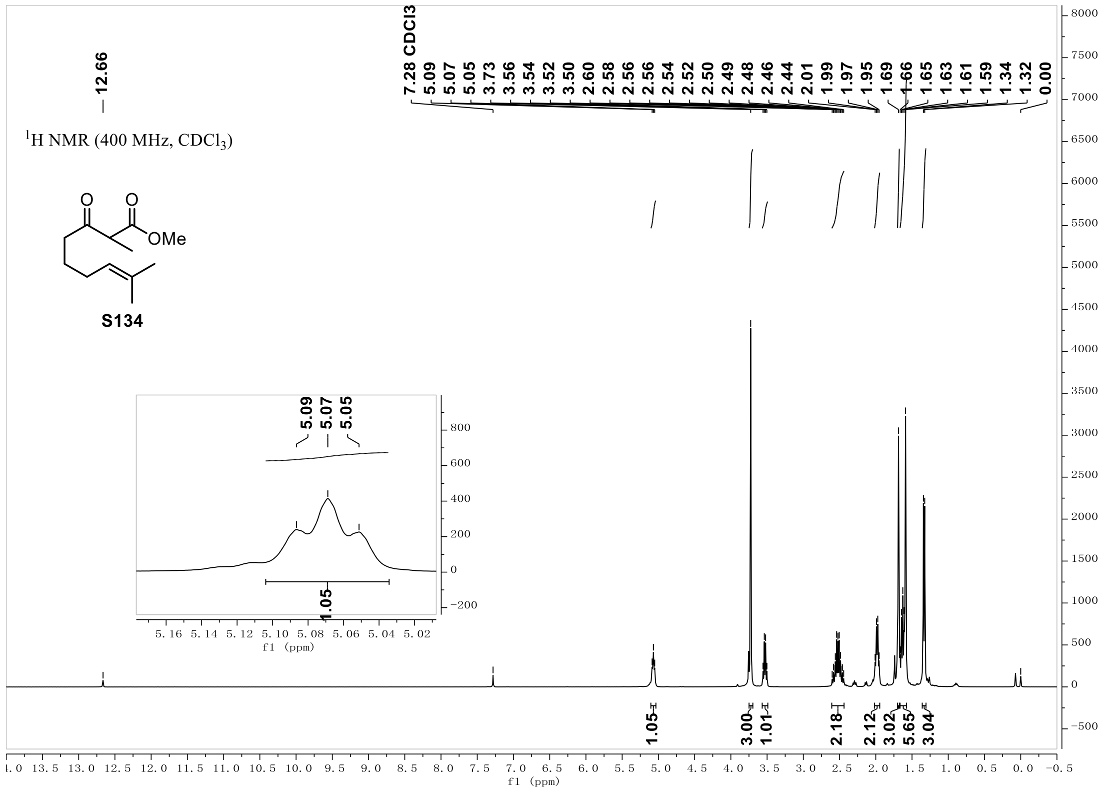

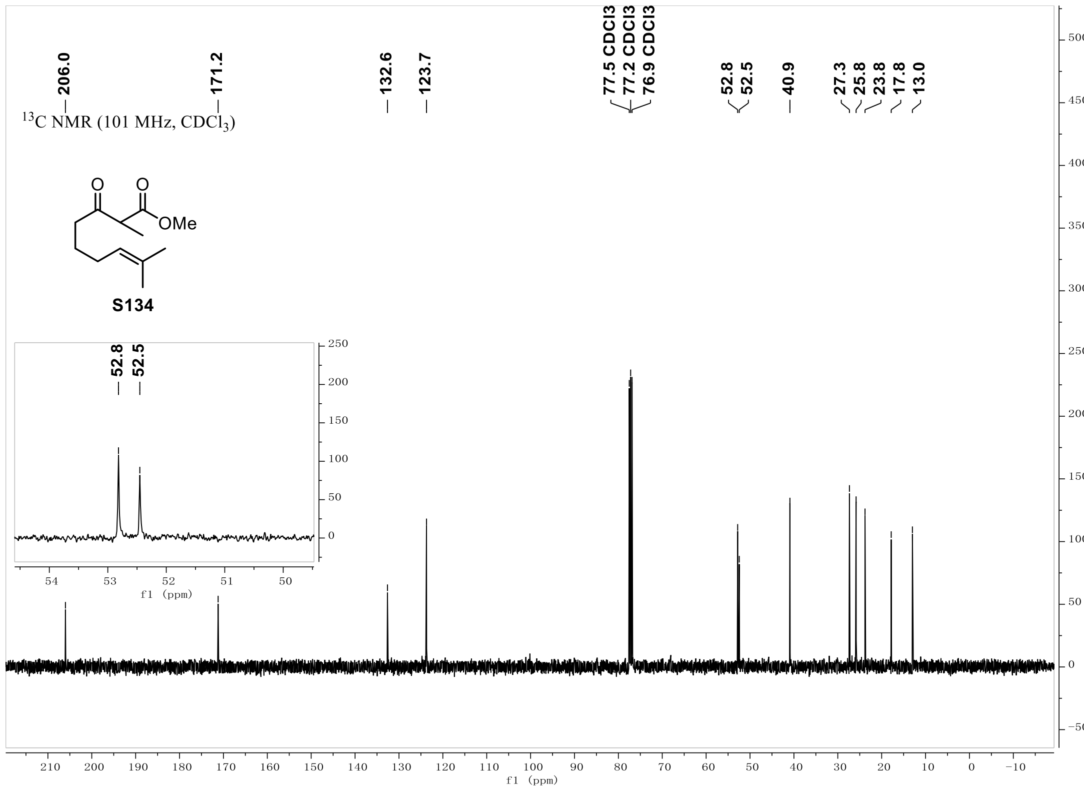

Supplementary Figure 125. ^{1}H NMR and ^{13}C NMR spectra of compound S134. The compound contained traces of enol form.

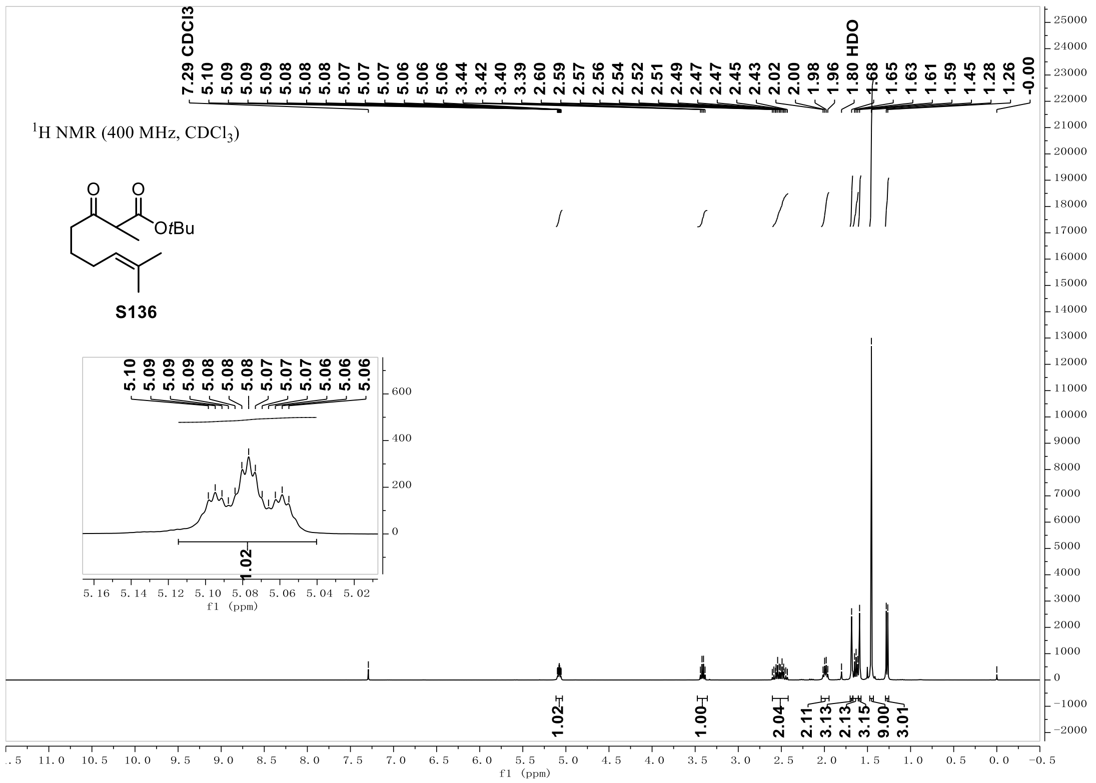

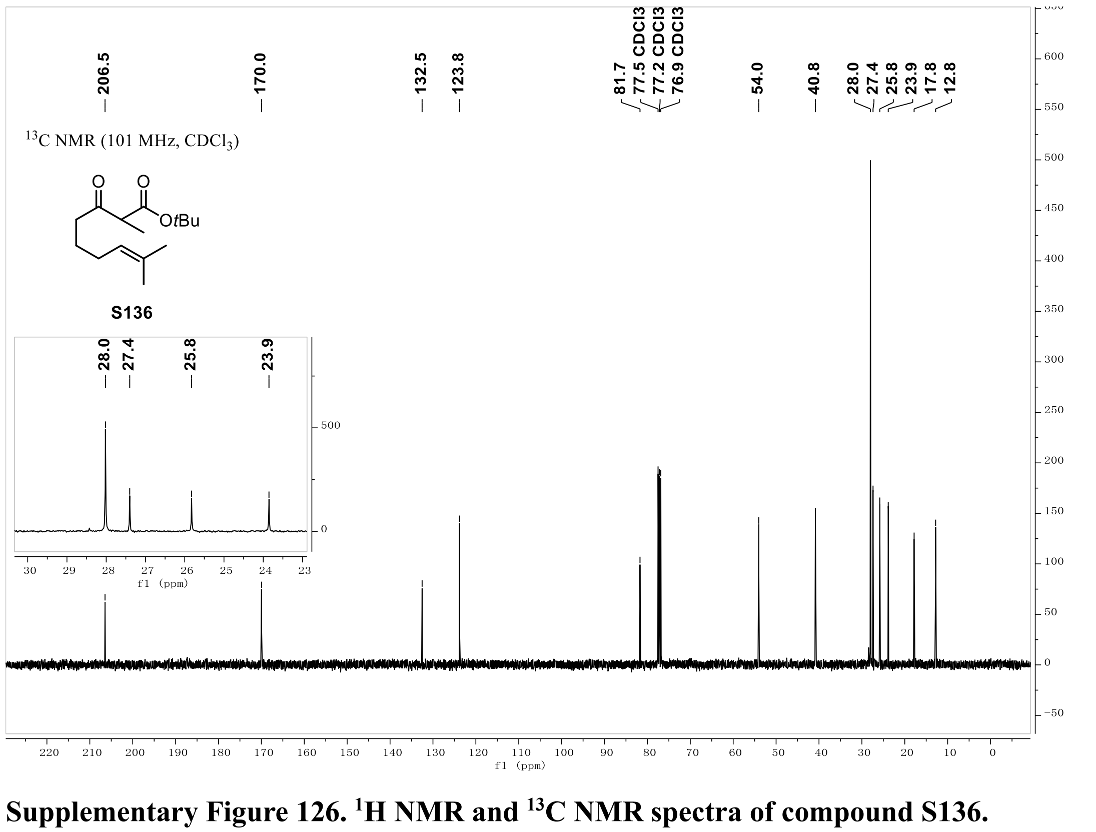

Supplementary Figure 126. ^{1}H NMR and ^{13}C NMR spectra of compound S136.

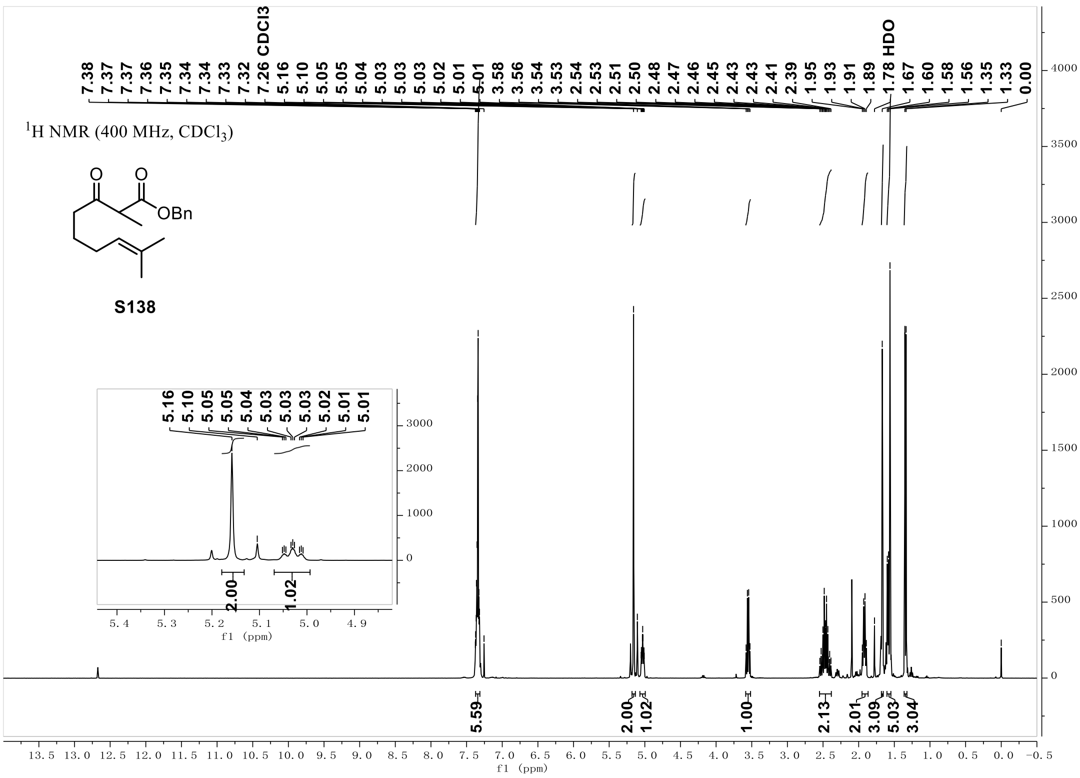

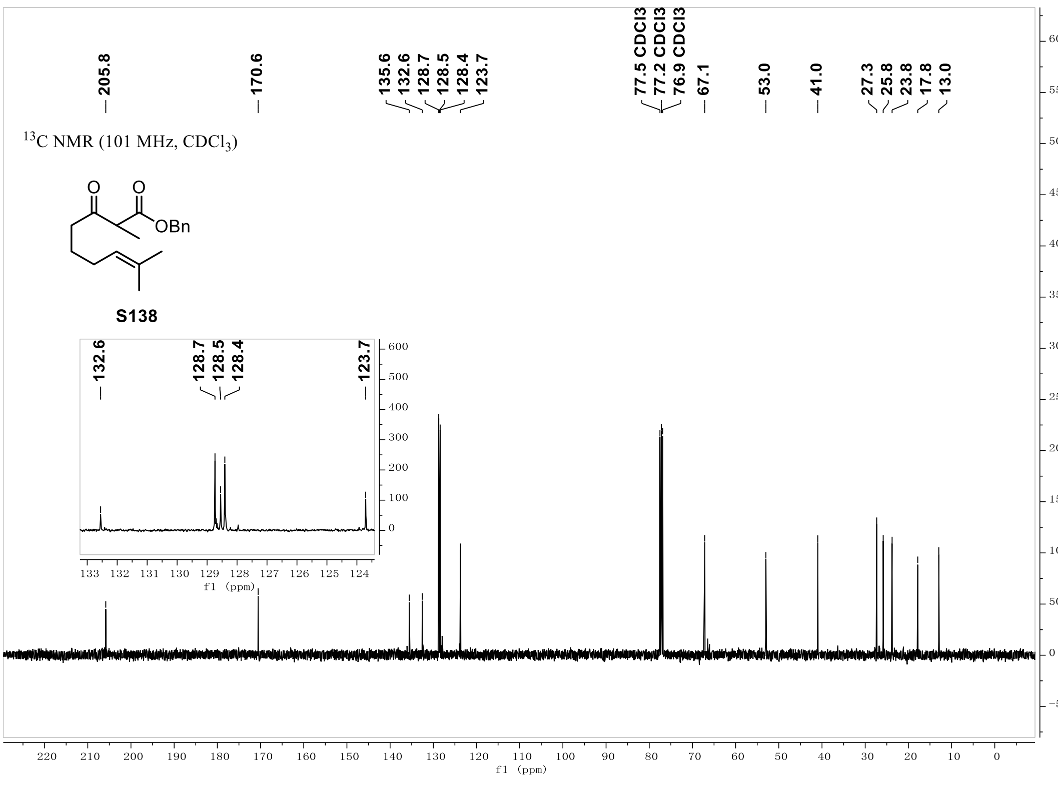

Supplementary Figure 127. ^{1}H NMR and ^{13}C NMR spectra of compound S138.

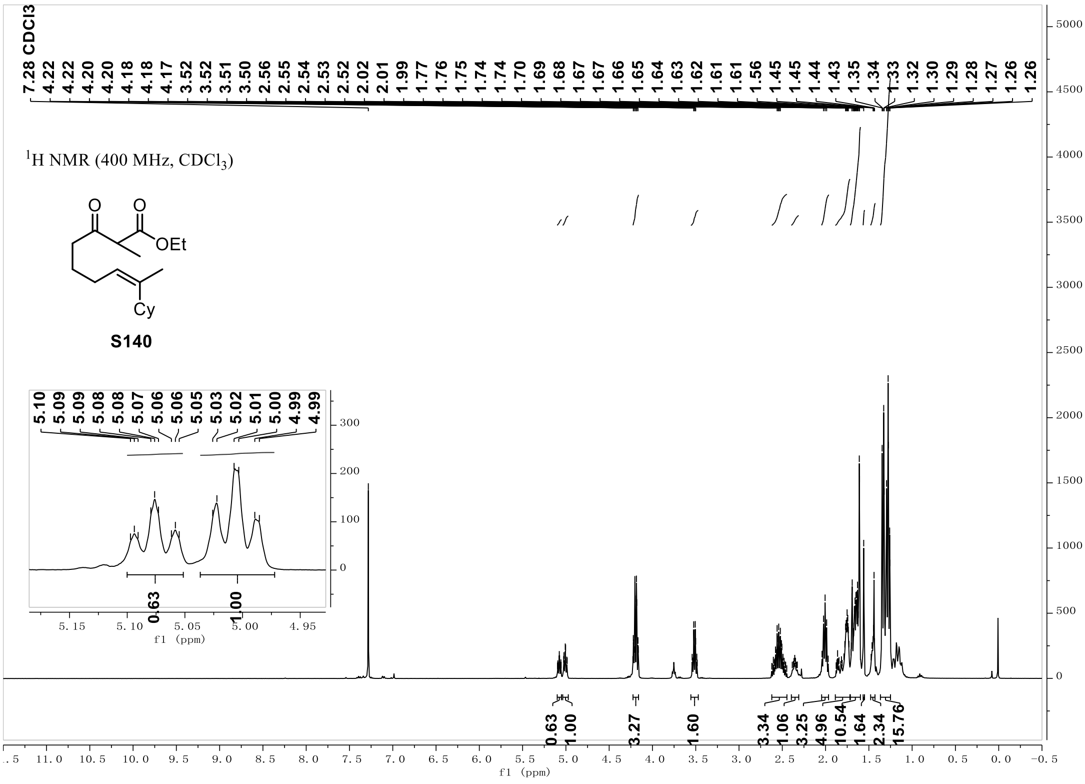

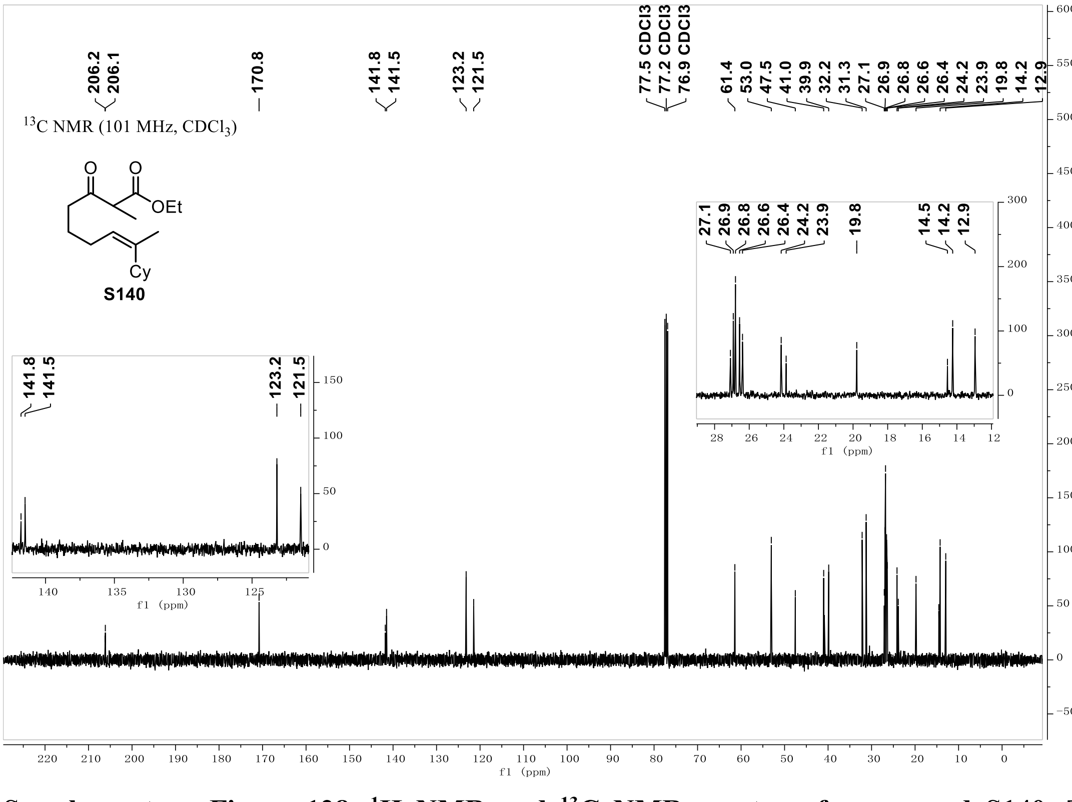

Supplementary Figure 128. ^{1}H NMR and ^{13}C NMR spectra of compound S140. The product was isolated as a 1:0.6 mixture of *Z*/*E* isomers.

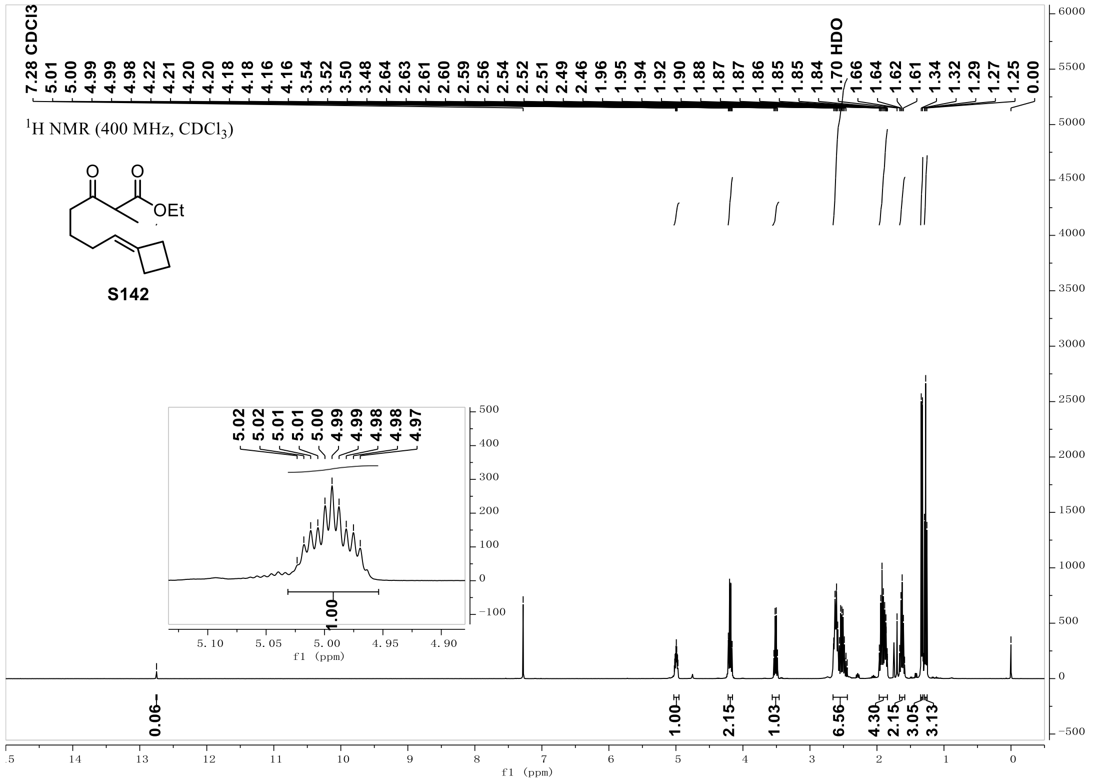

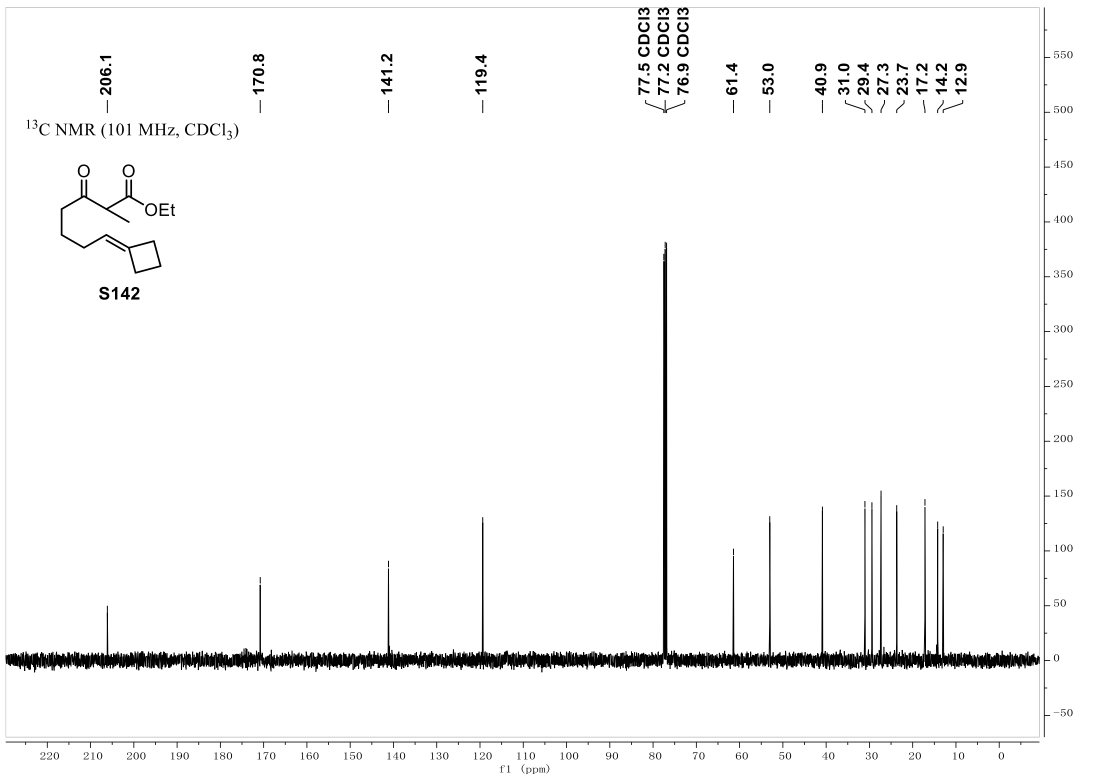

Supplementary Figure 129. ^{1}H NMR and ^{13}C NMR spectra of compound S142. The compound contained < 10% of enol form.

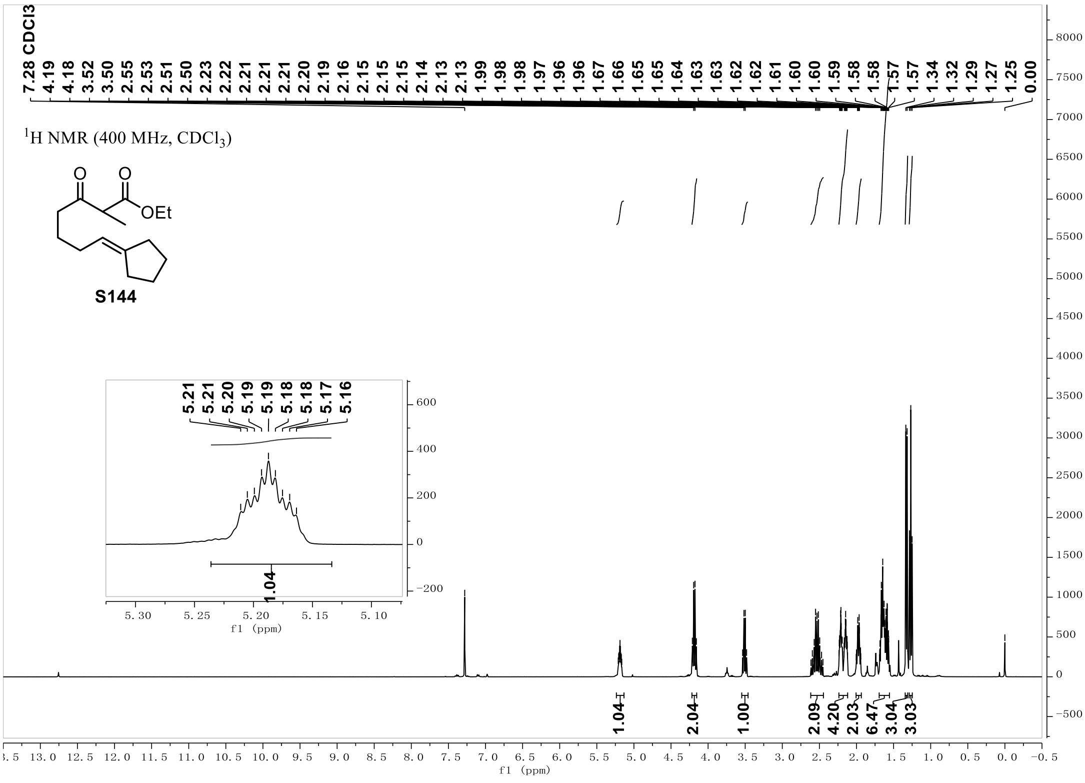

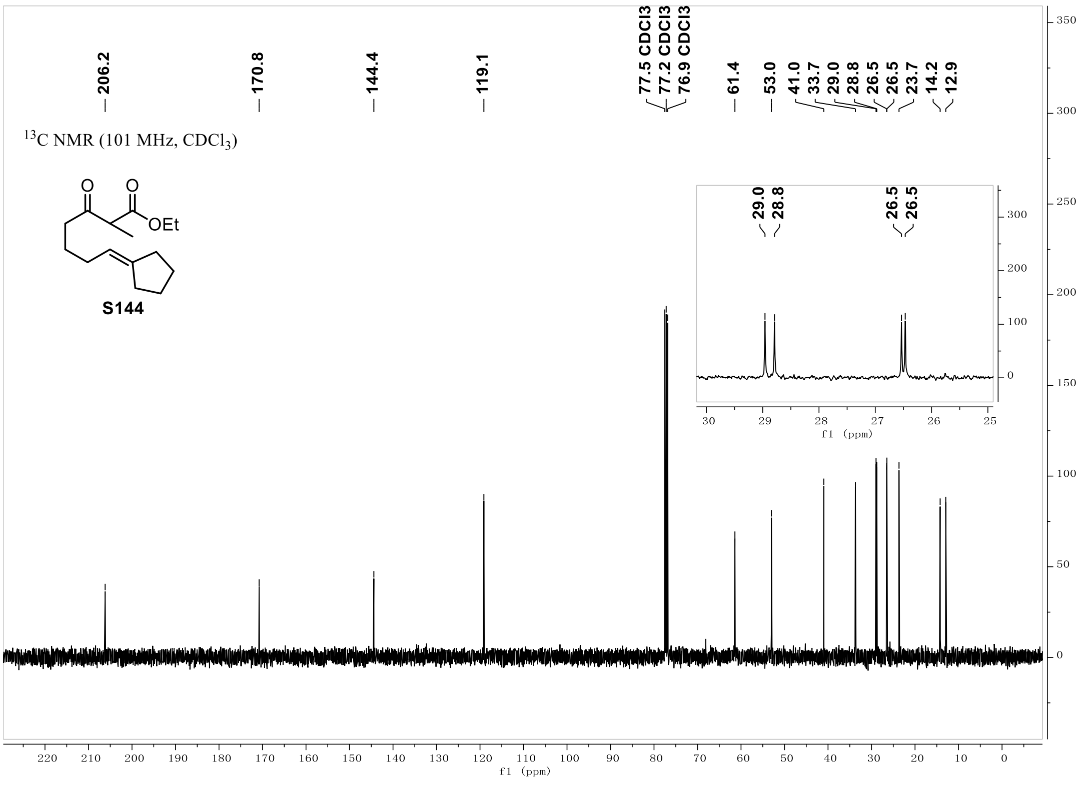

Supplementary Figure 130. ^{1}H NMR and ^{13}C NMR spectra of compound S144. The compound contained traces of enol form.

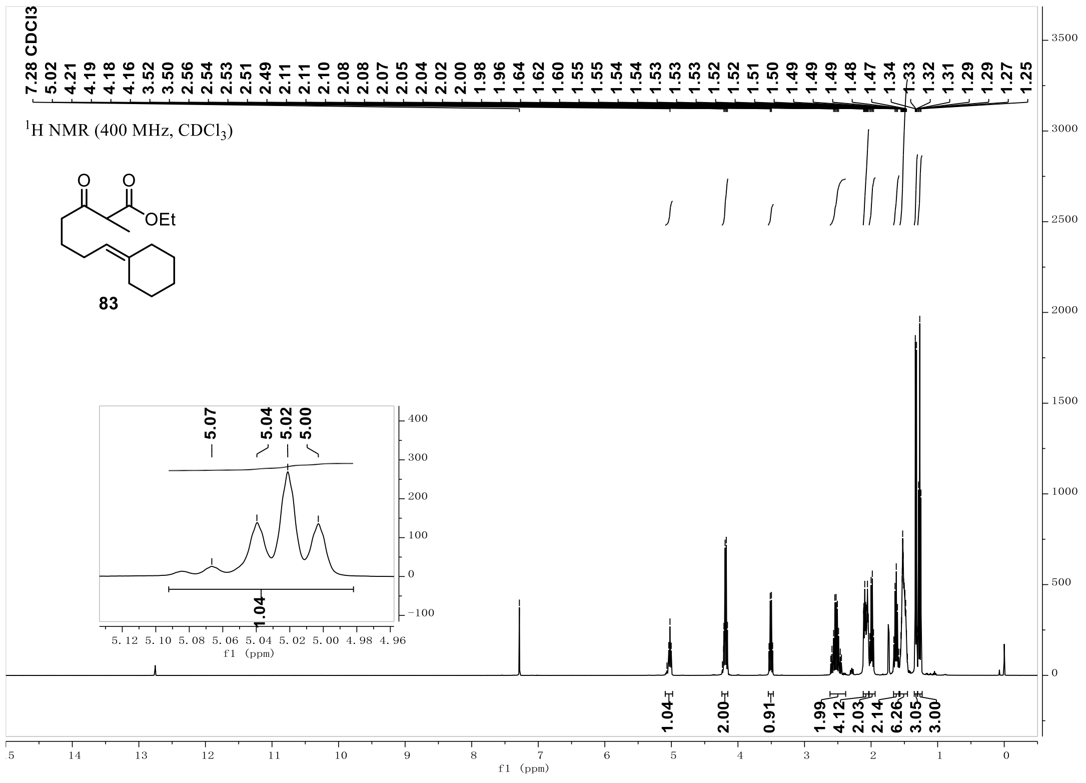

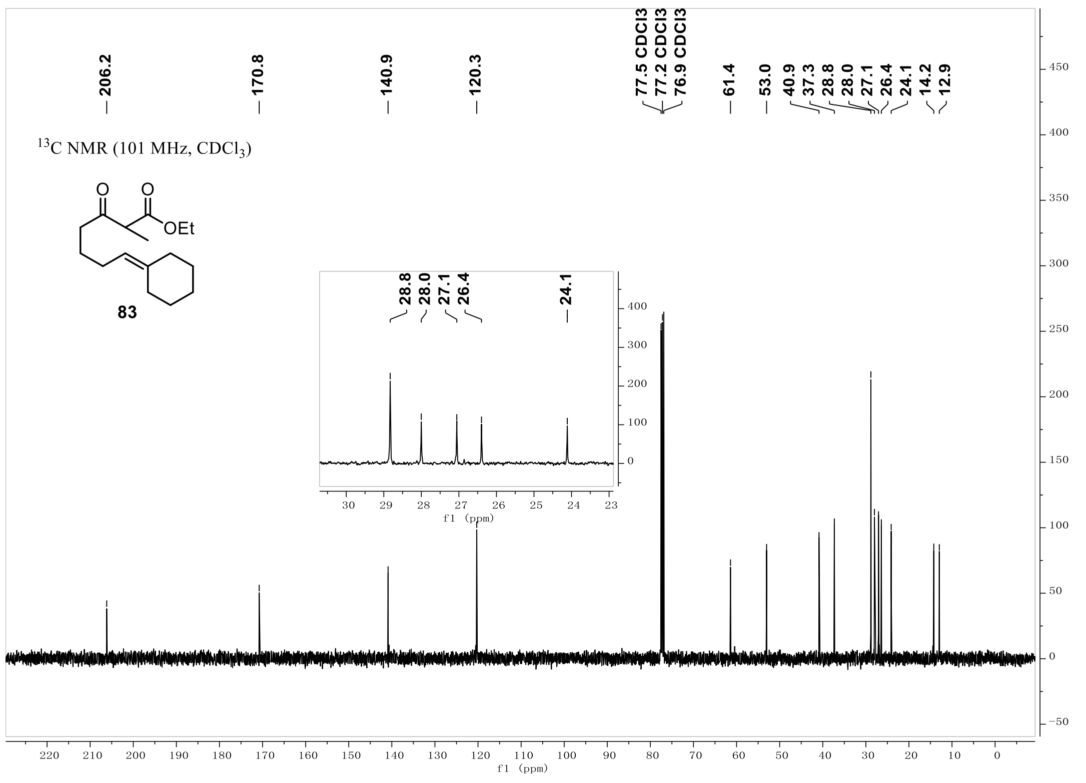

Supplementary Figure 131. ^{1}H NMR and ^{13}C NMR spectra of compound 83. The compound contained traces of enol form.

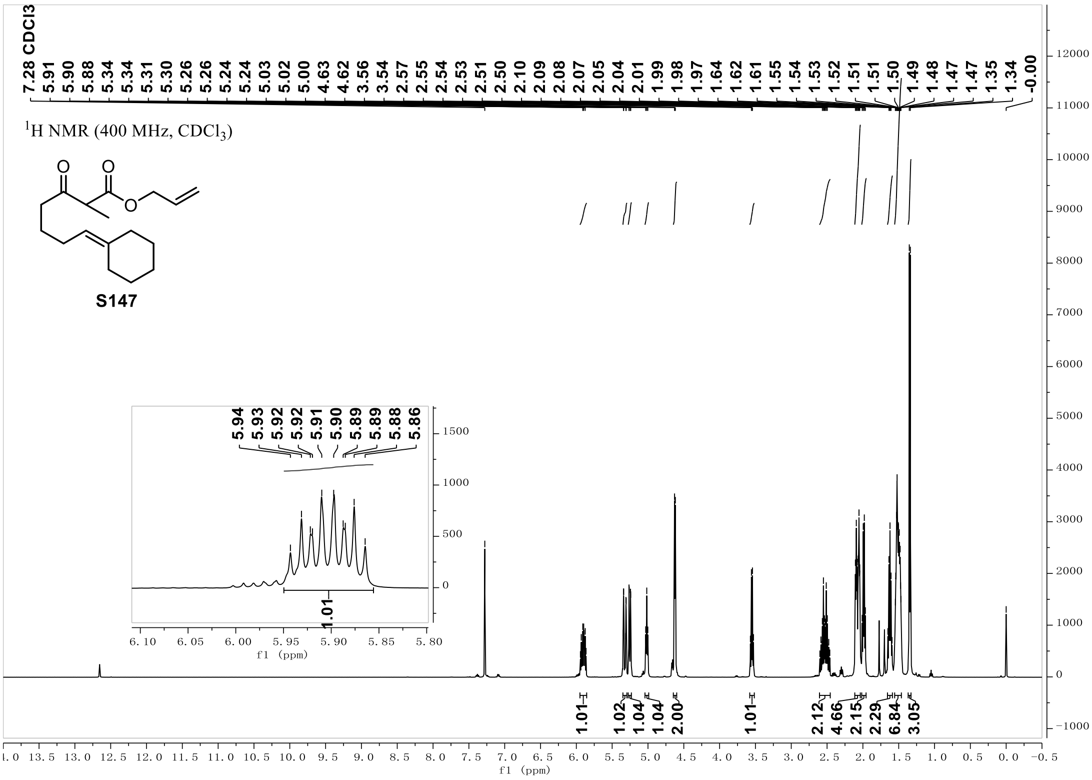

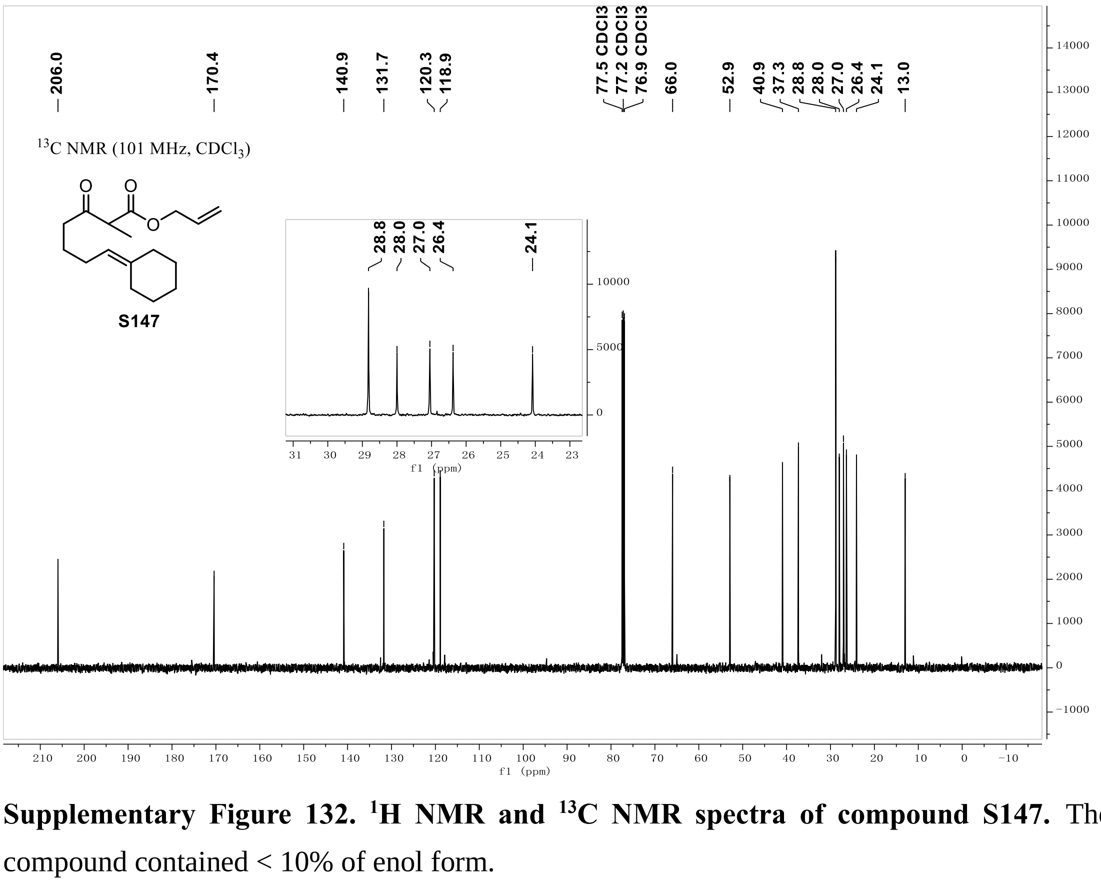

Supplementary Figure 132. ^{1}H NMR and ^{13}C NMR spectra of compound S147. The compound contained < 10% of enol form.

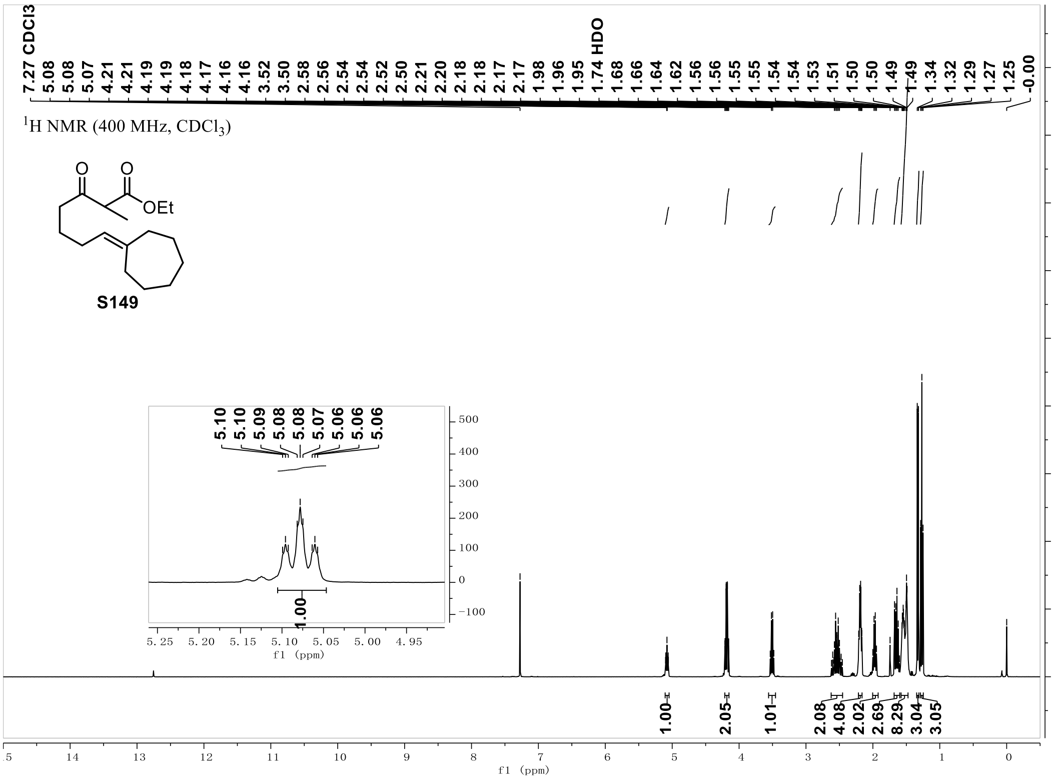

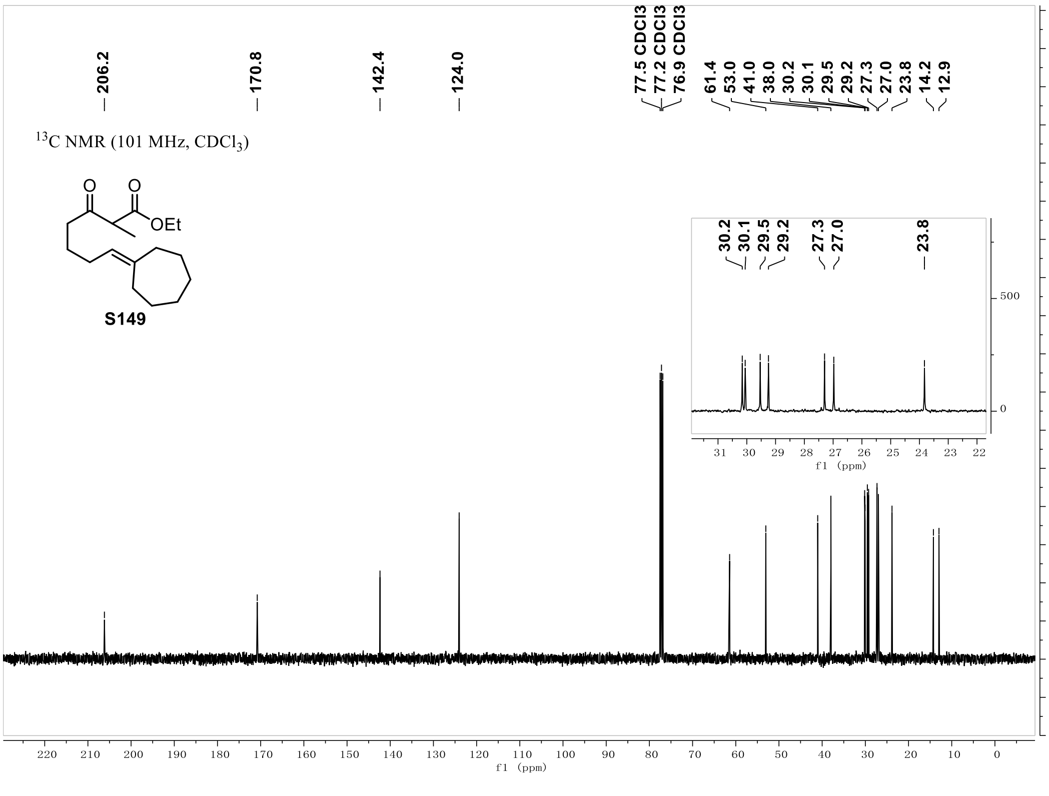

Supplementary Figure 133. ^{1}H NMR and ^{13}C NMR spectra of compound S149. The compound contained traces of enol form.

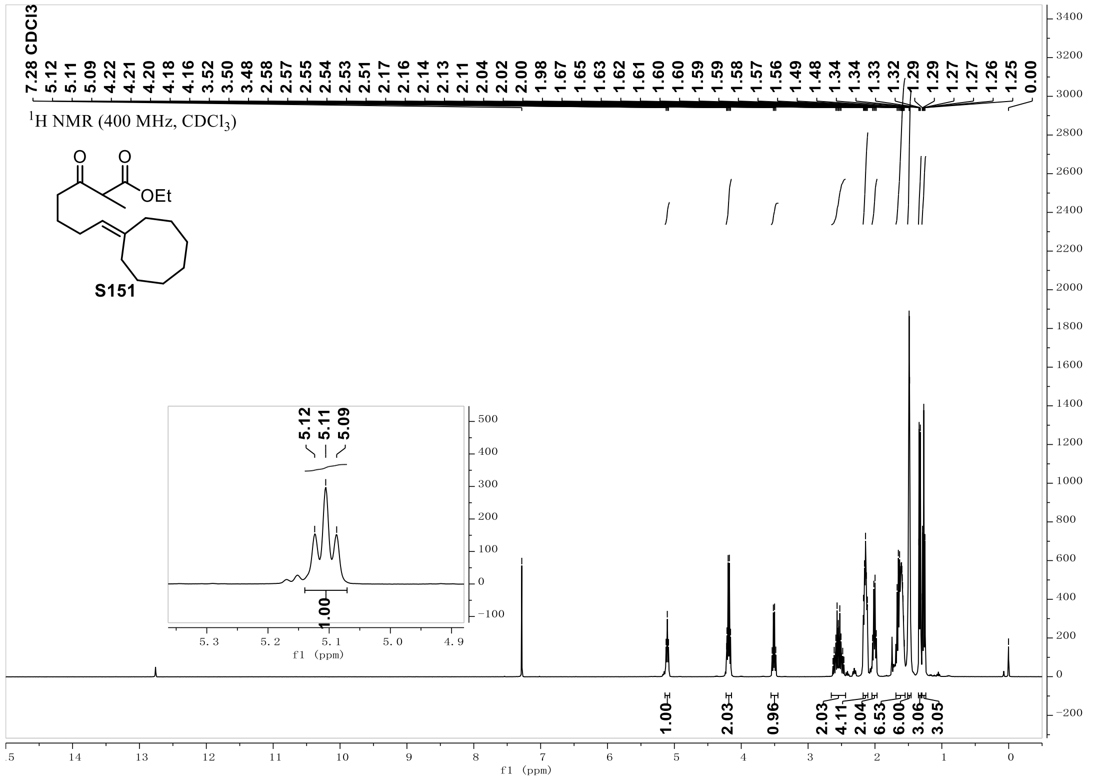

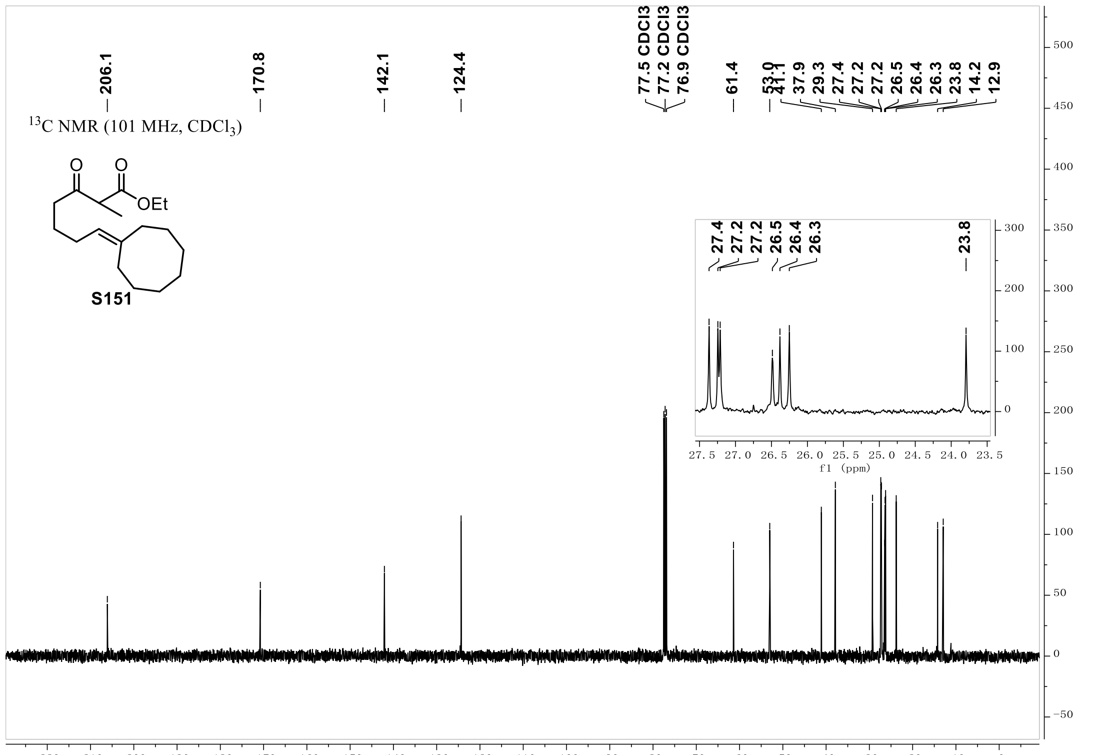

Supplementary Figure 134. ^{1}H NMR and ^{13}C NMR spectra of compound S151. The compound contained < 10% of enol form.

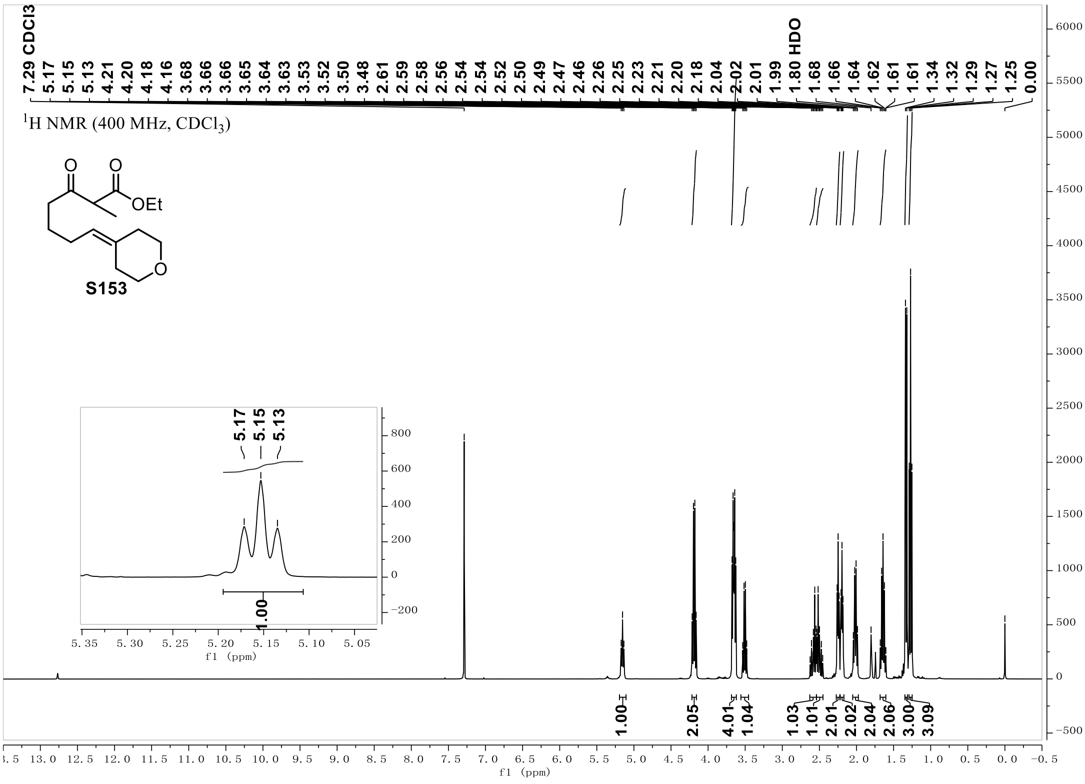

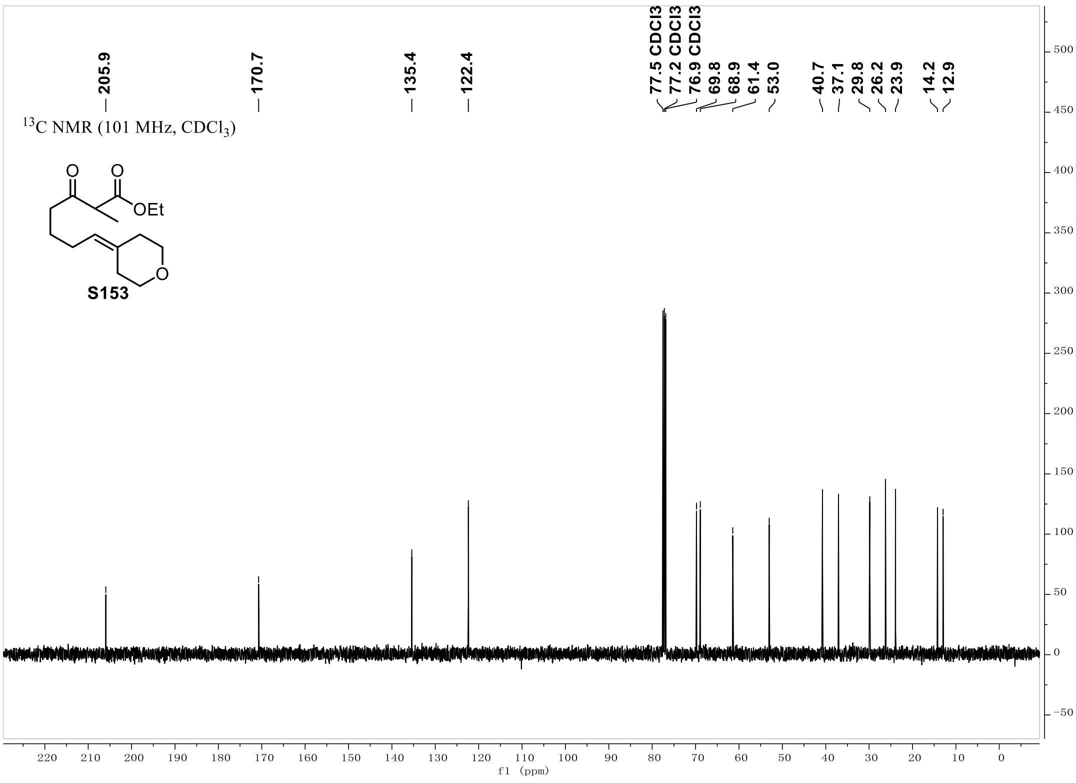

Supplementary Figure 135. ^{1}H NMR and ^{13}C NMR spectra of compound S153. The compound contained traces of enol form.

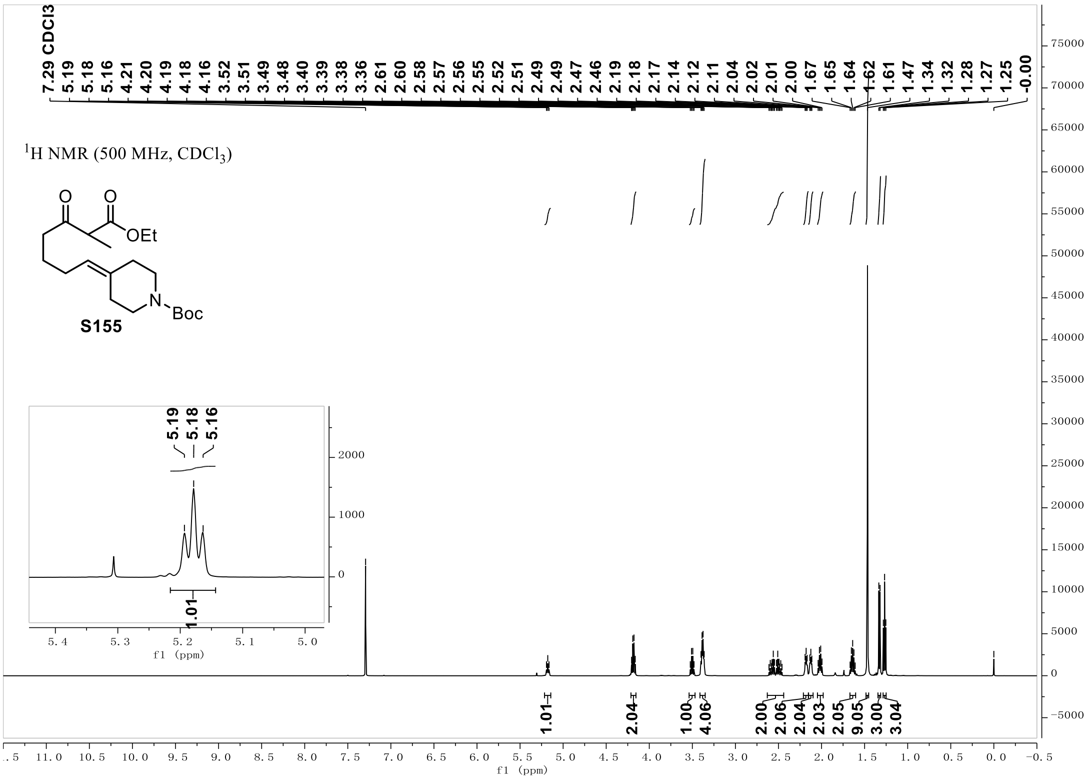

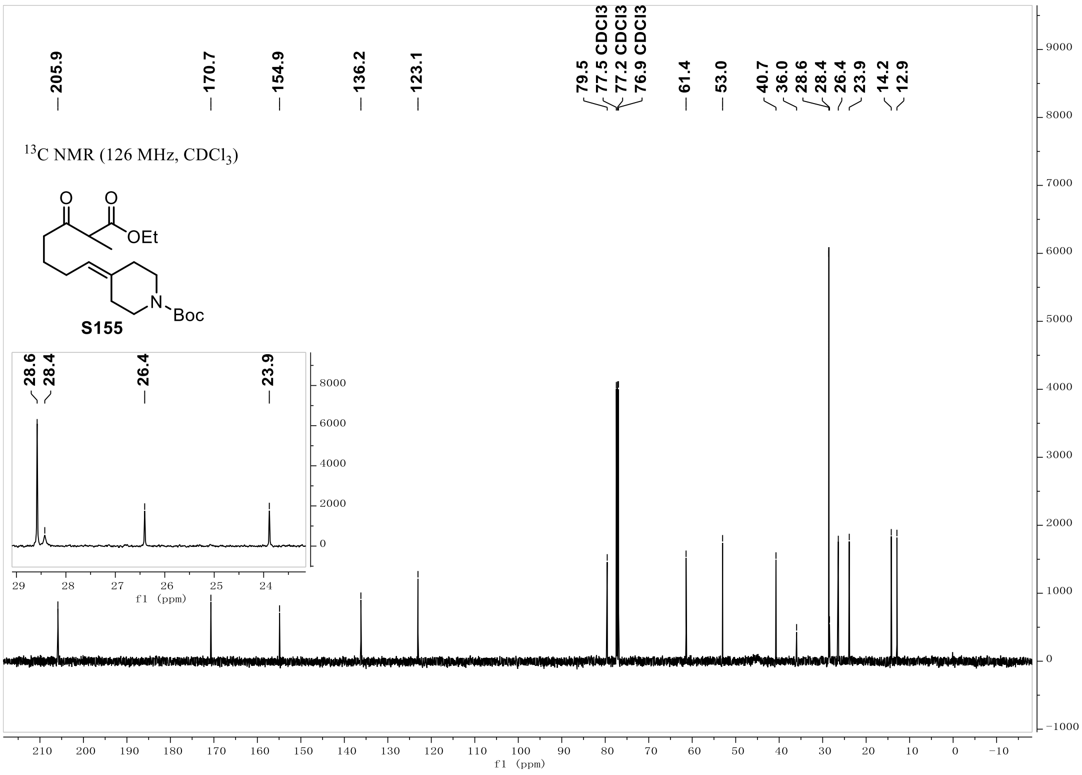

Supplementary Figure 136. ^{1}H NMR and ^{13}C NMR spectra of compound S155.

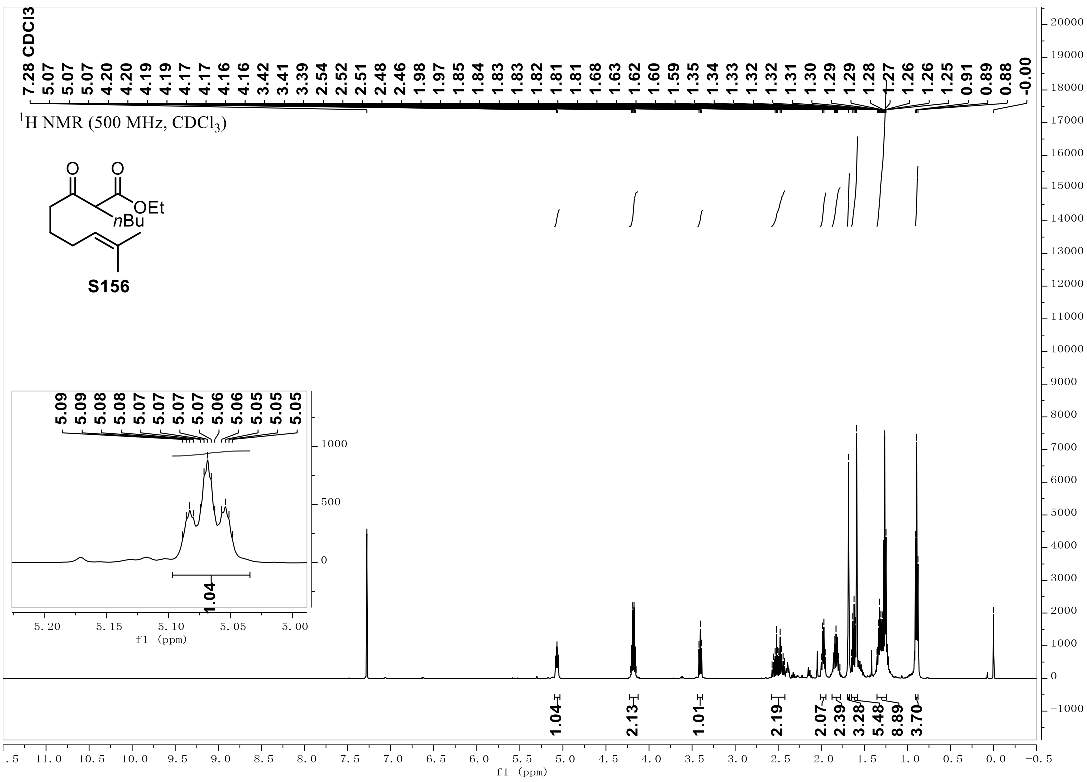

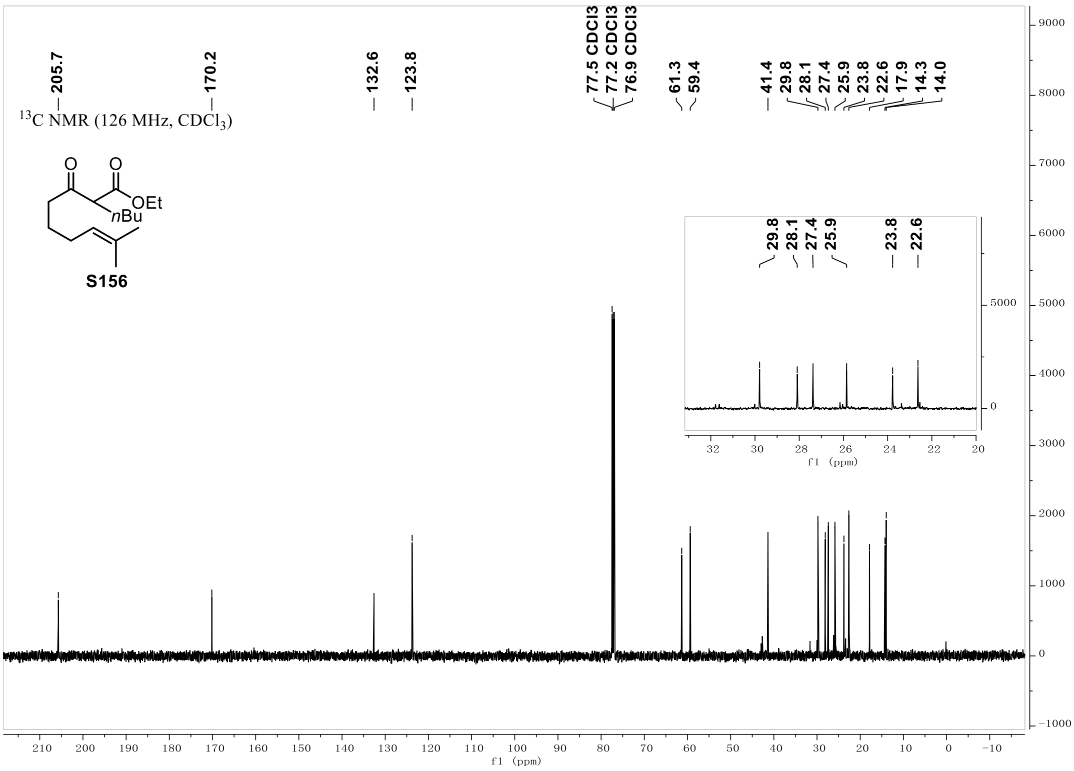

Supplementary Figure 137. ^{1}H NMR and ^{13}C NMR spectra of compound S156. The compound contained < 10% of enol form.

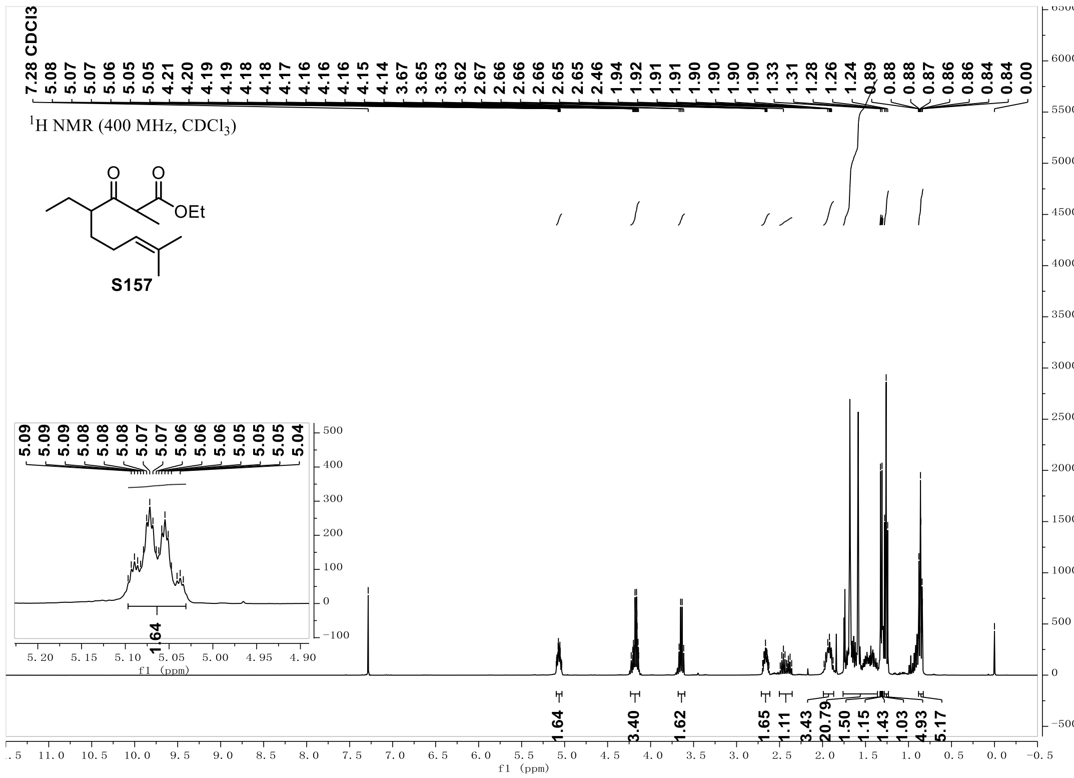

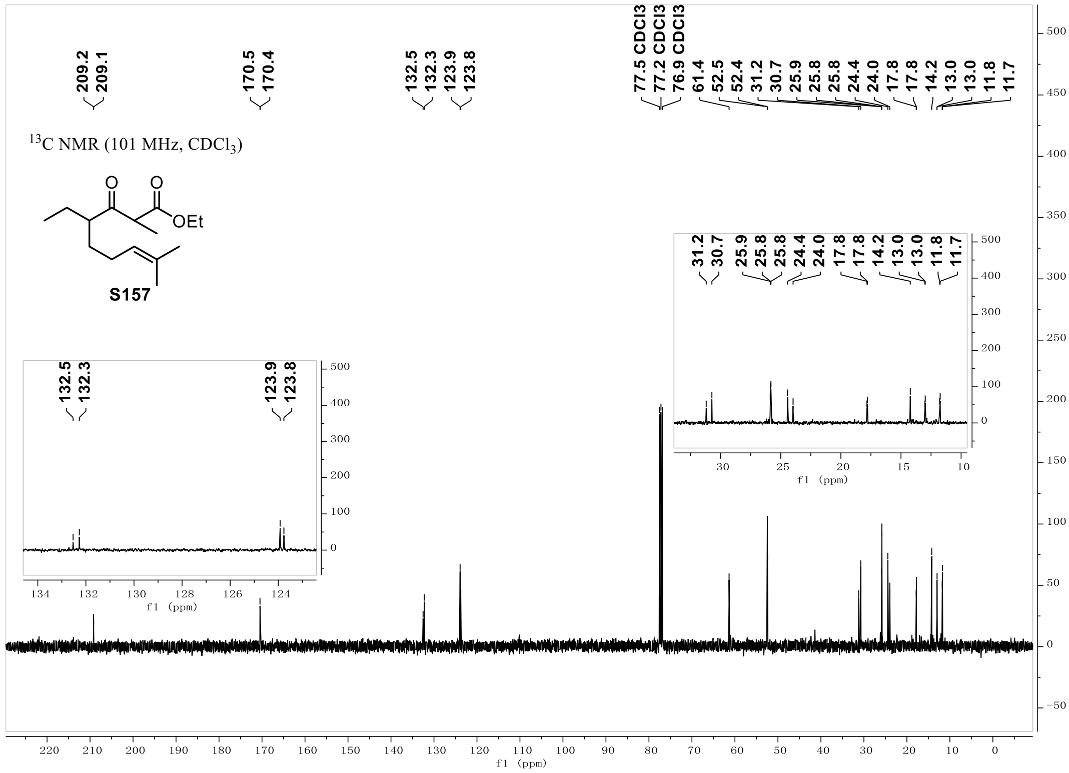

Supplementary Figure 138. ^{1}H NMR and ^{13}C NMR spectra of compound S157. The product was isolated as a 1:0.6 mixture of diastereoisomers.

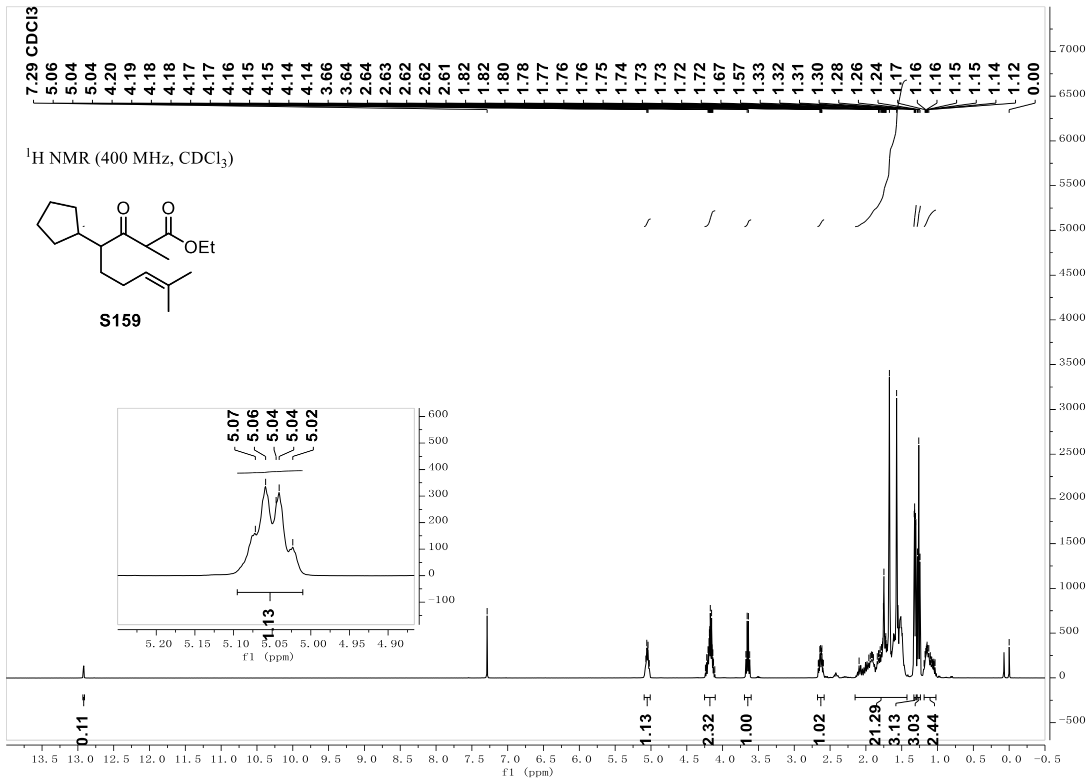

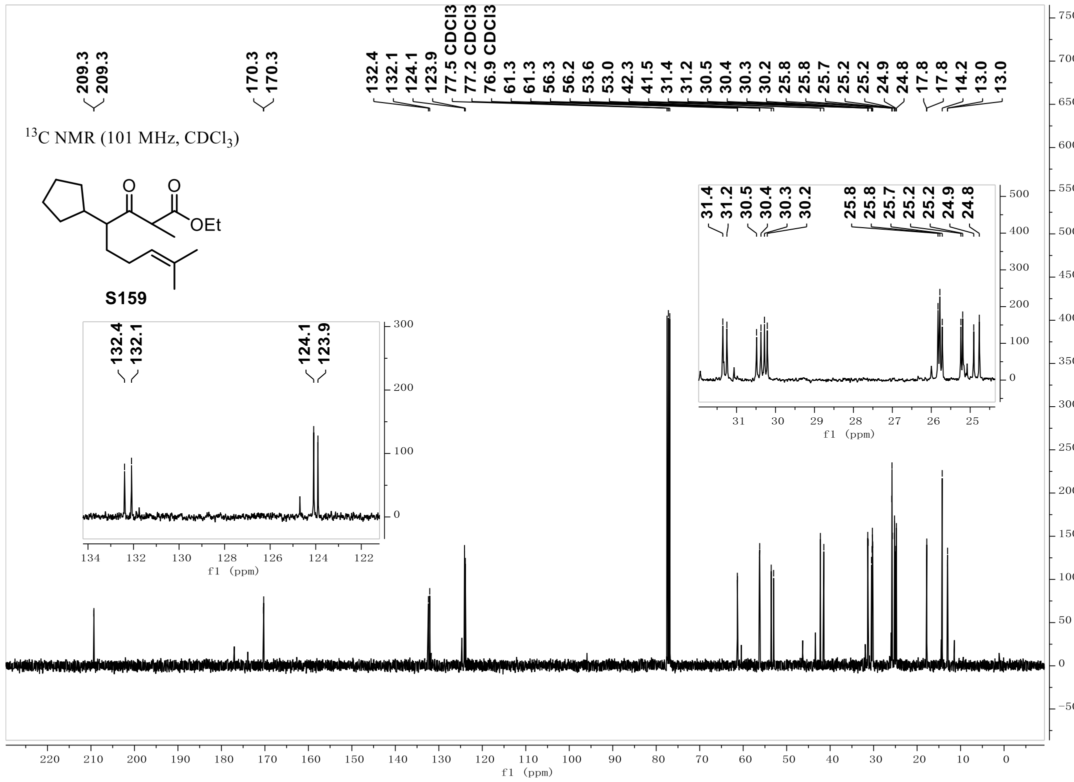

Supplementary Figure 139. ^{1}H NMR and ^{13}C NMR spectra of compound S159. The product was isolated as a 1:1 mixture of diastereoisomers.

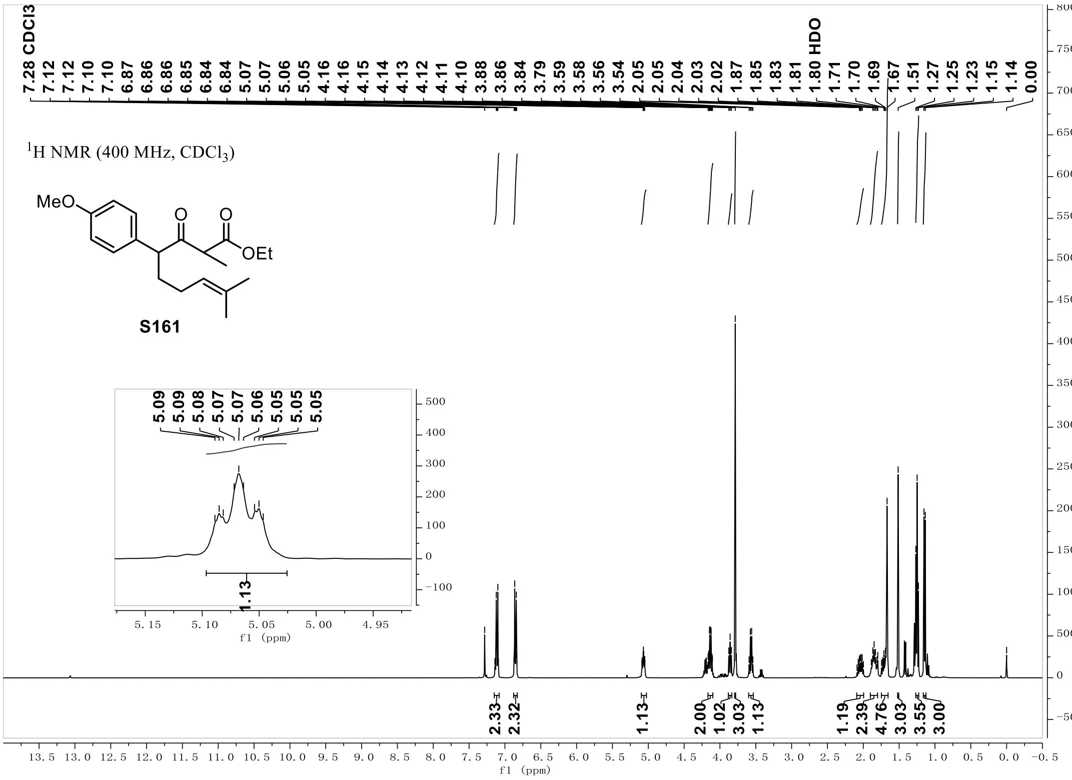

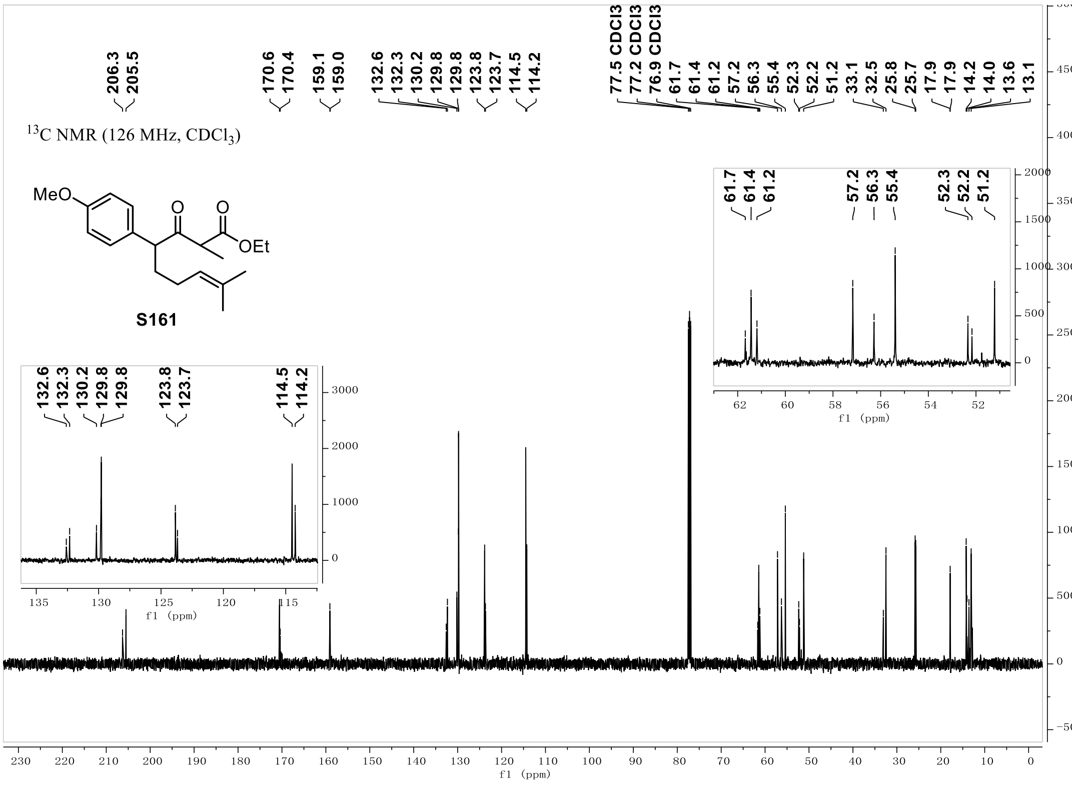

Supplementary Figure 140. ^{1}H NMR and ^{13}C NMR spectra of compound S161. The product was isolated as a 1:0.15 mixture of diastereoisomers and contained traces of enol form.

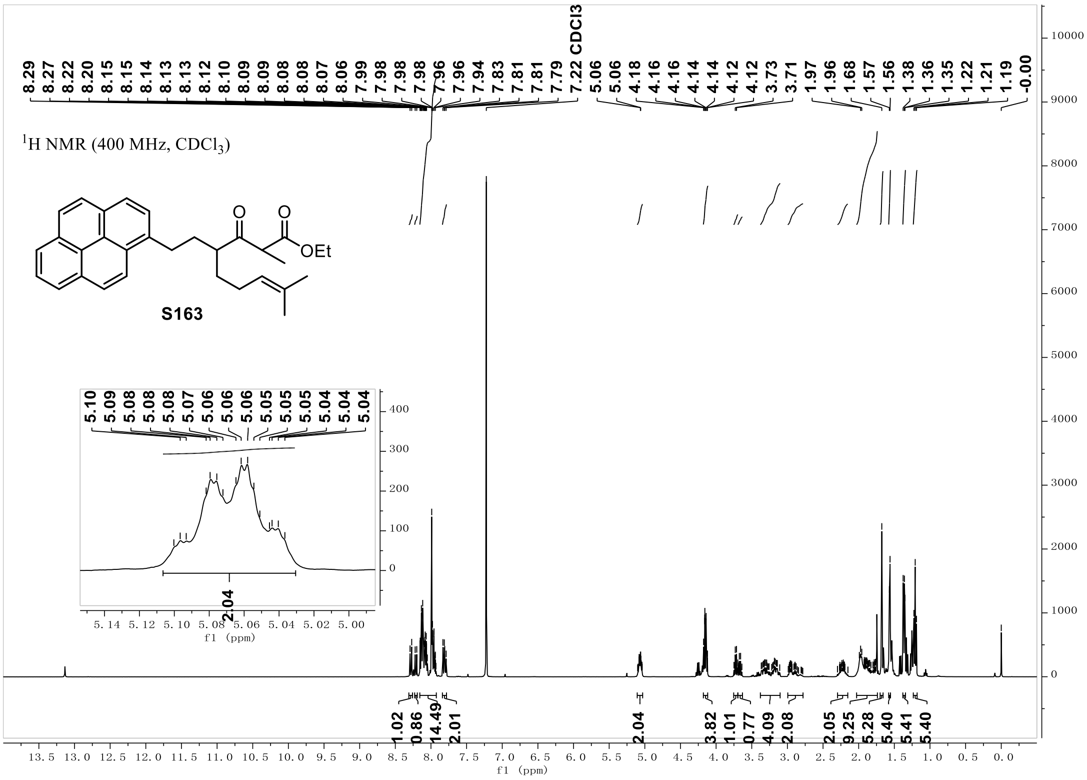

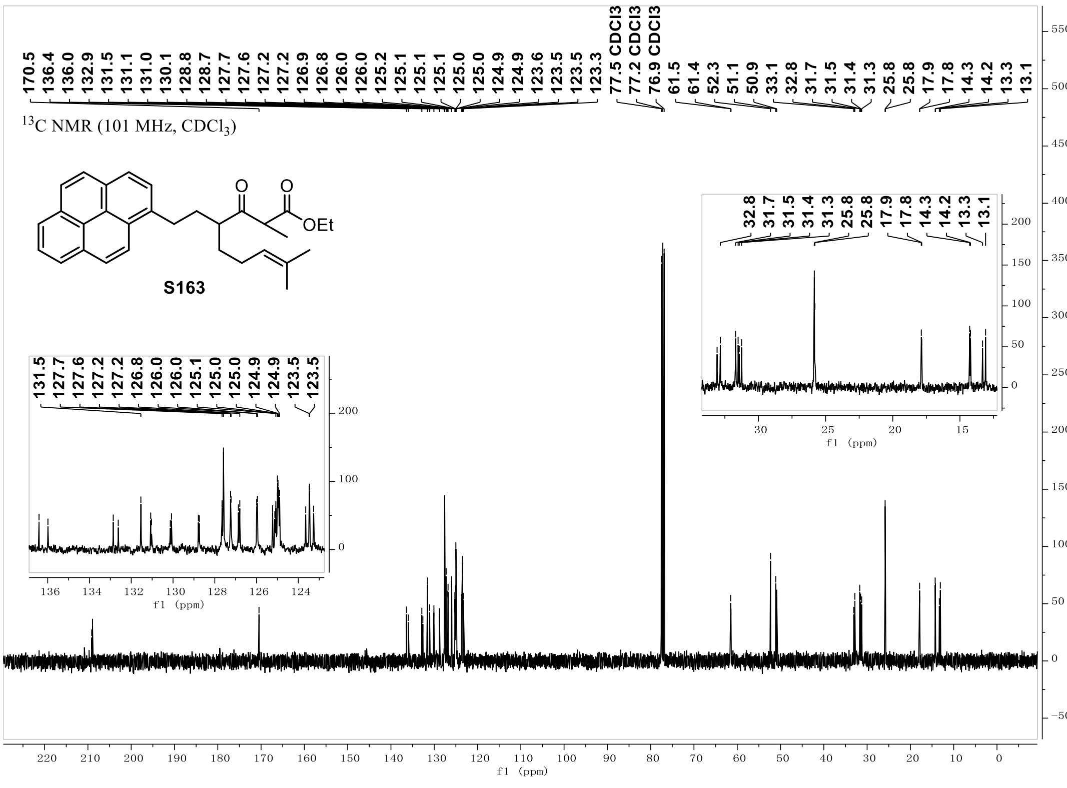

Supplementary Figure 141. ^{1}H NMR and ^{13}C NMR spectra of compound S163. The product was isolated as a 1:0.8 mixture of diastereoisomers and contained 14% of enol forms.

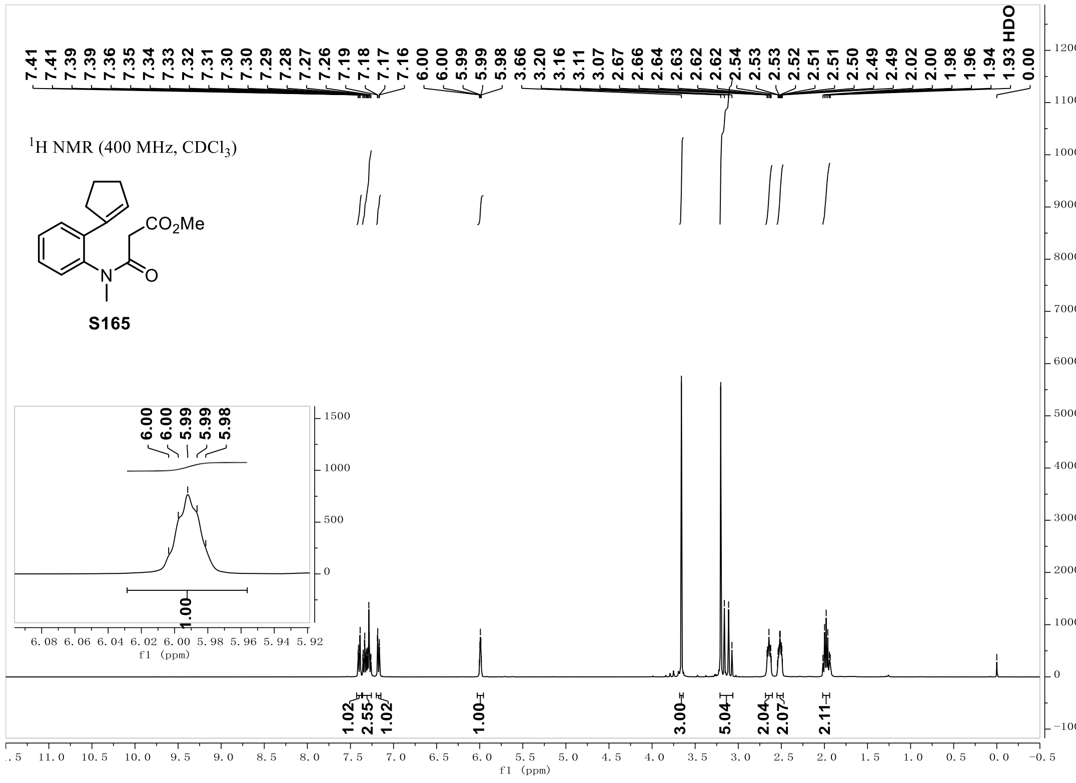

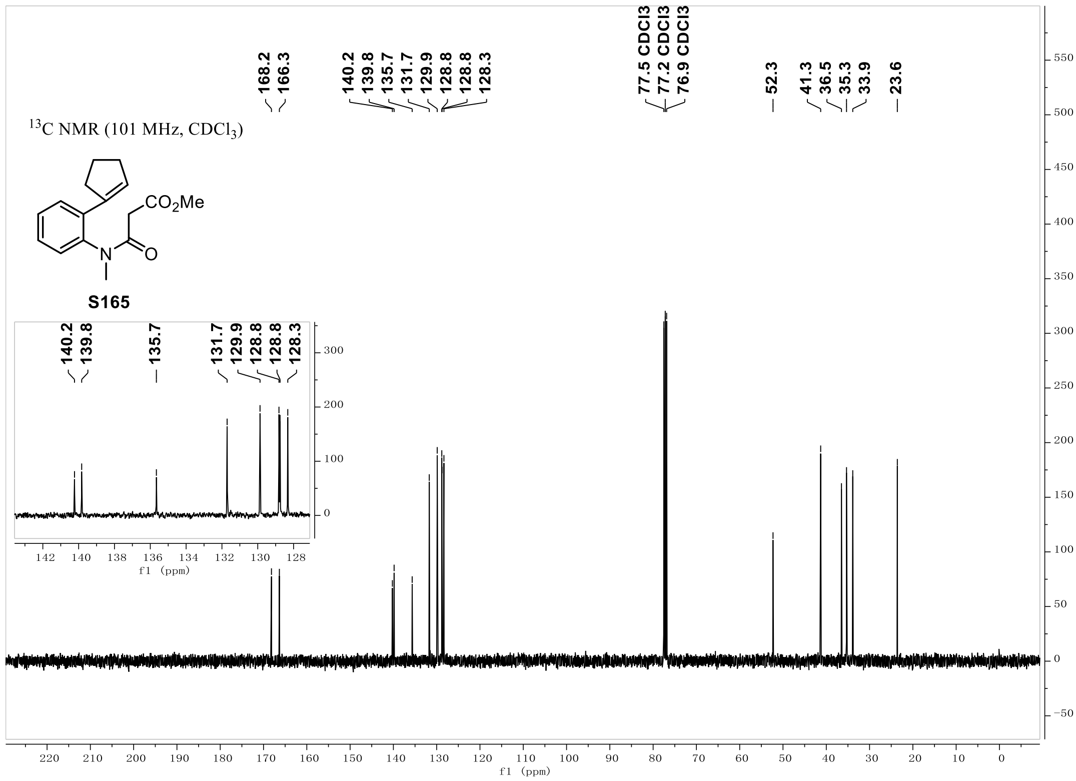

Supplementary Figure 142. ^{1}H NMR and ^{13}C NMR spectra of compound S165.

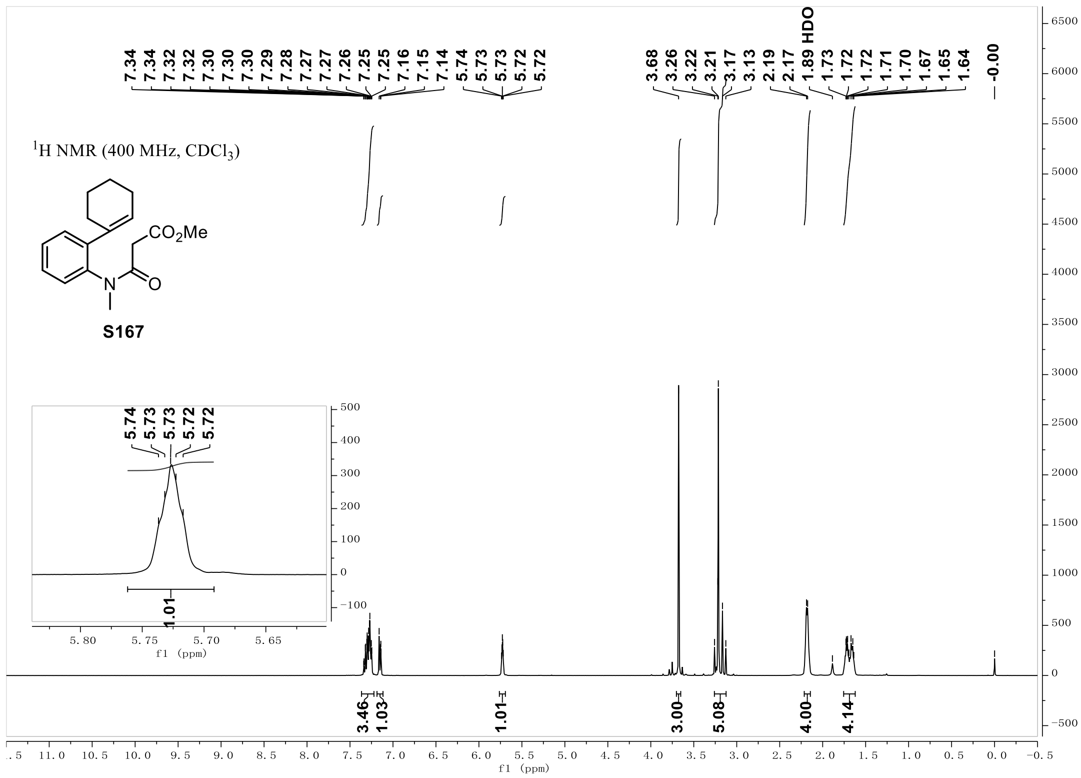

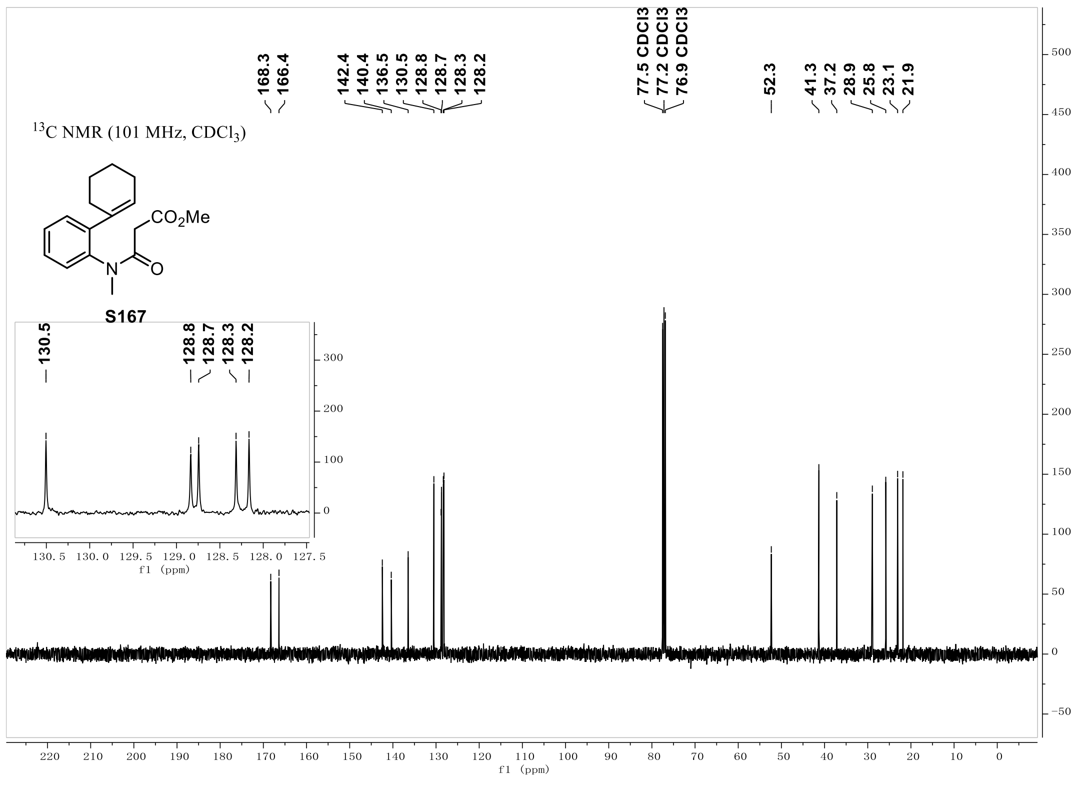

Supplementary Figure 143. ^{1}H NMR and ^{13}C NMR spectra of compound S167.

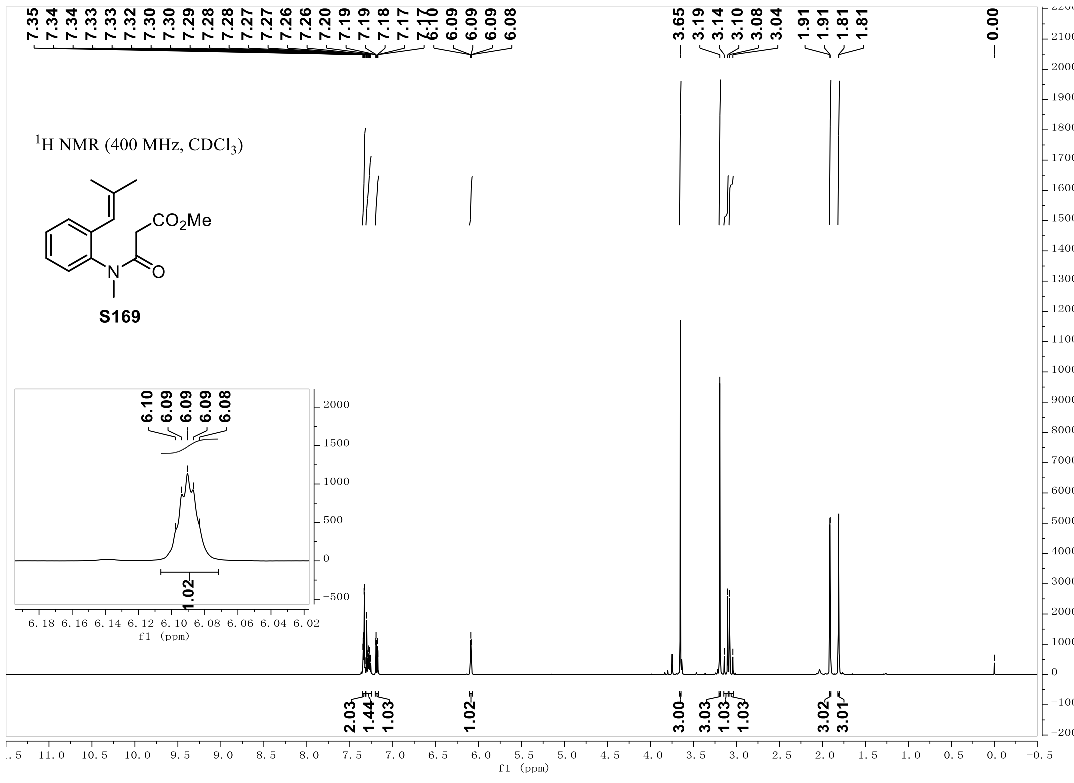

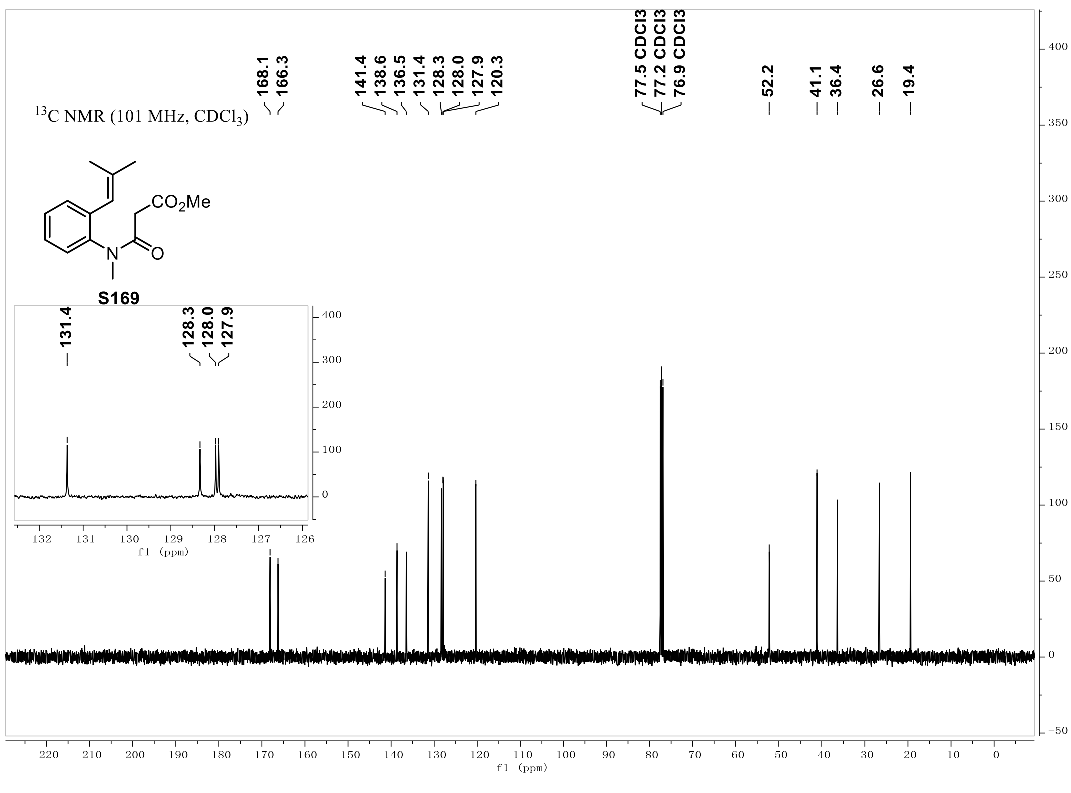

Supplementary Figure 144. ^{1}H NMR and ^{13}C NMR spectra of compound S169.

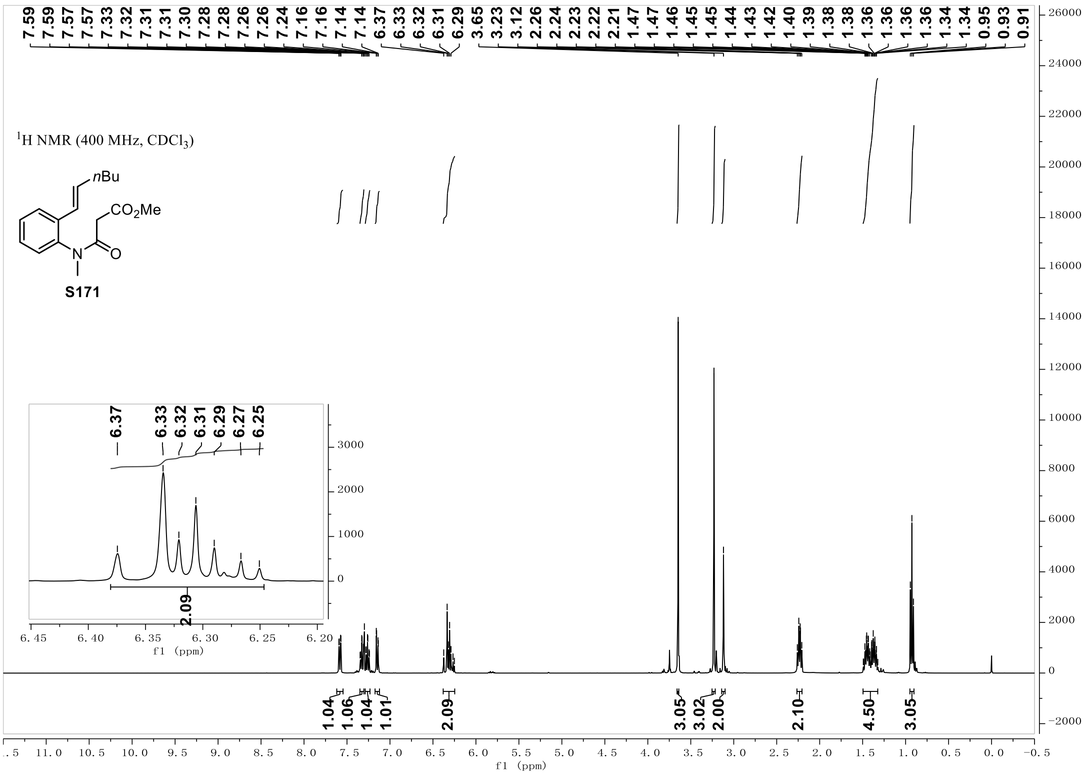

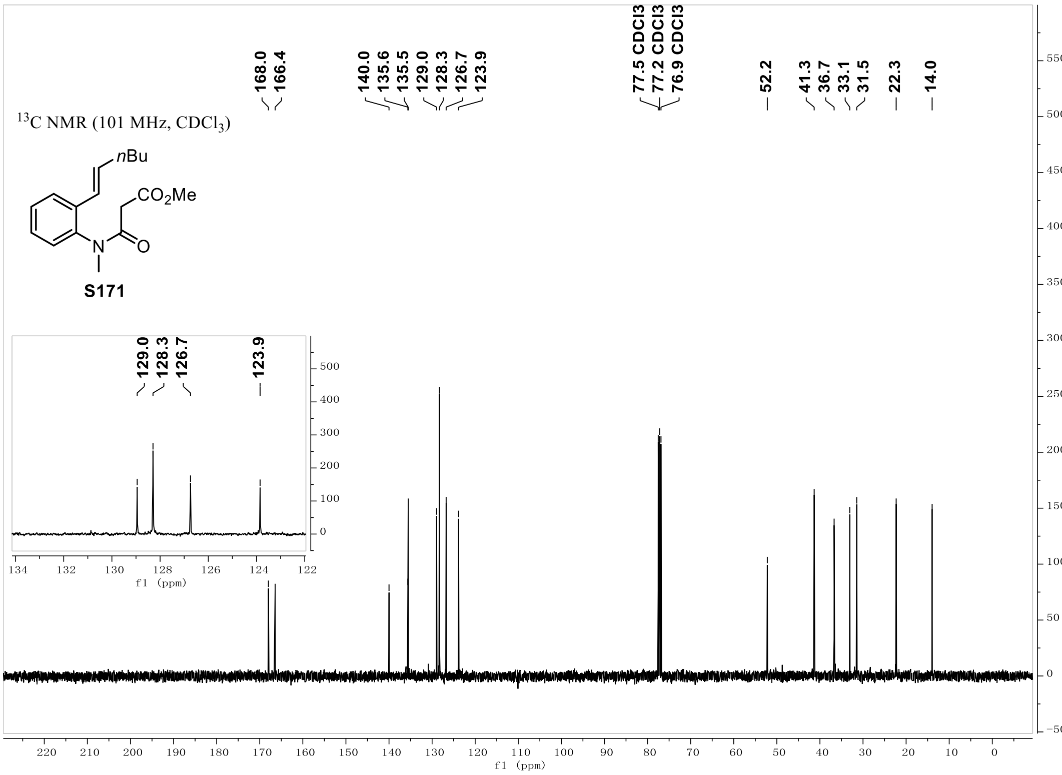

Supplementary Figure 145. ^{1}H NMR and ^{13}C NMR spectra of compound S171.

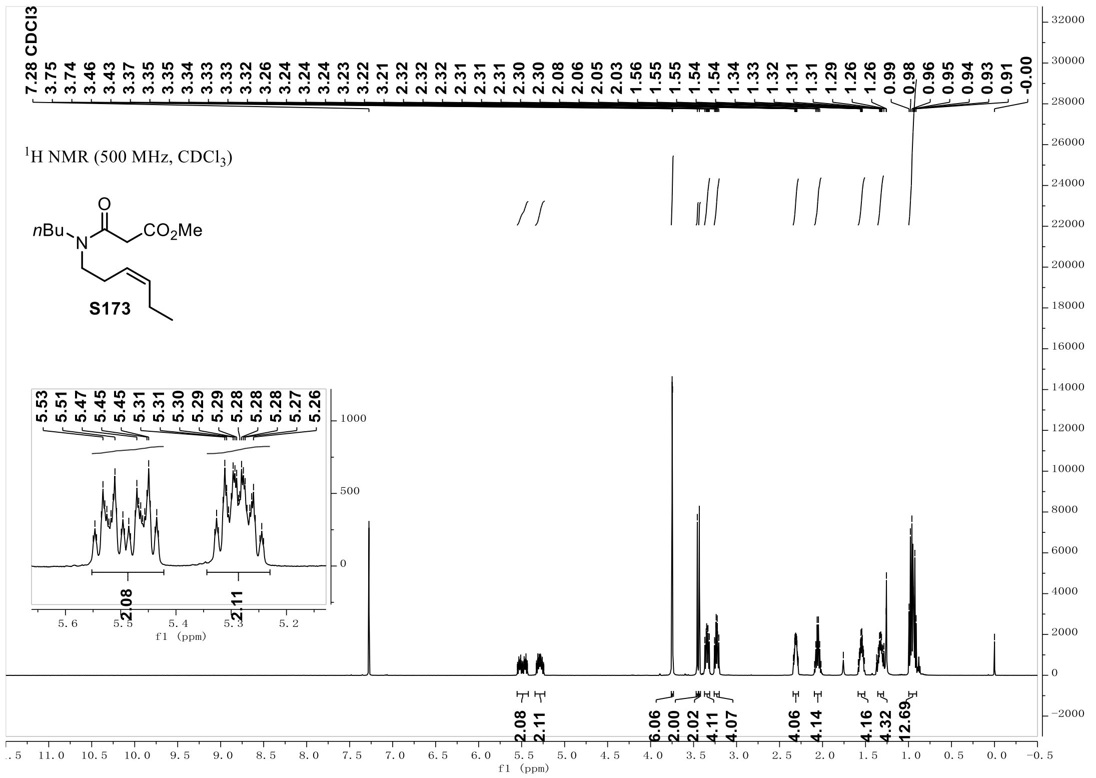

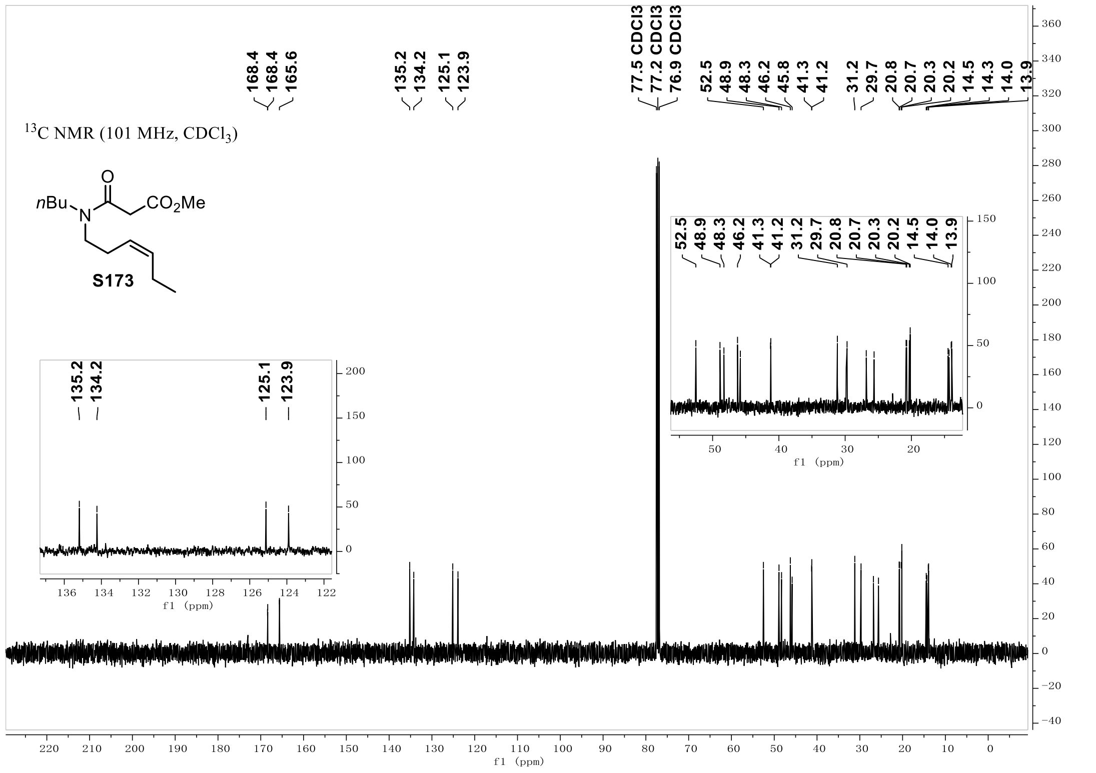

Supplementary Figure 146. ^{1}H NMR and ^{13}C NMR spectra of compound S173. The title compound was isolated as a 1:1 mixture of rotamers.

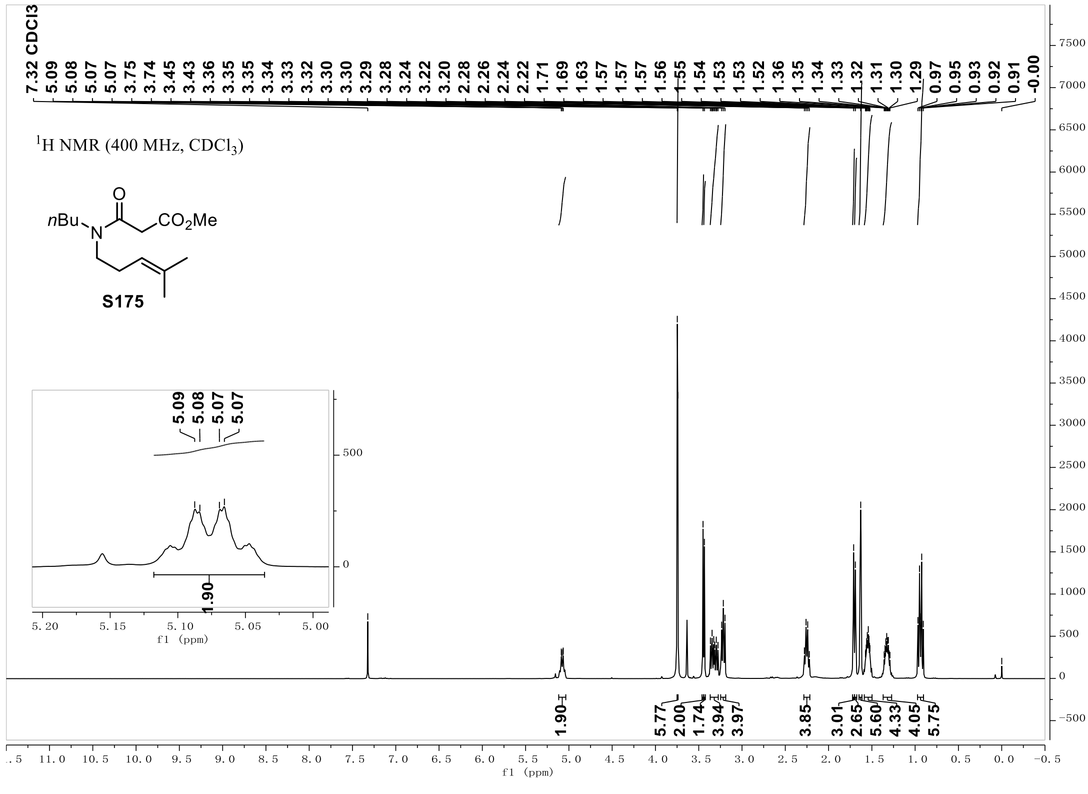

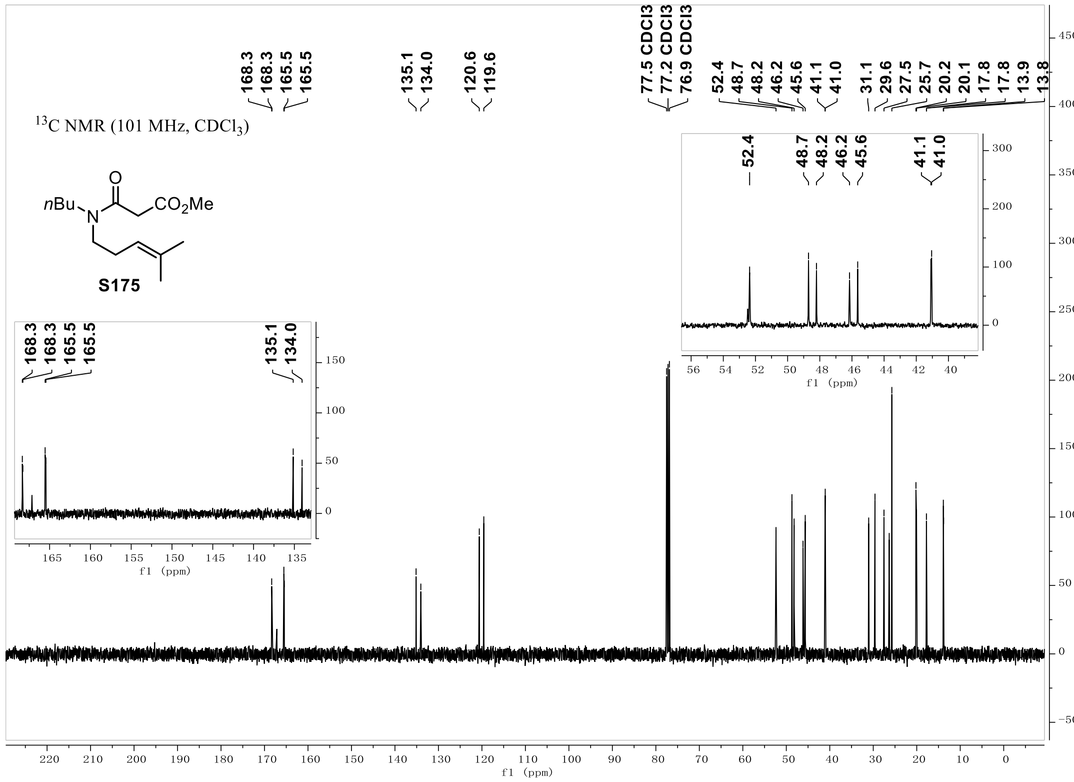

Supplementary Figure 147. ^{1}H NMR and ^{13}C NMR spectra of compound S175. The product was isolated as a 1:0.9 mixture of rotamers.

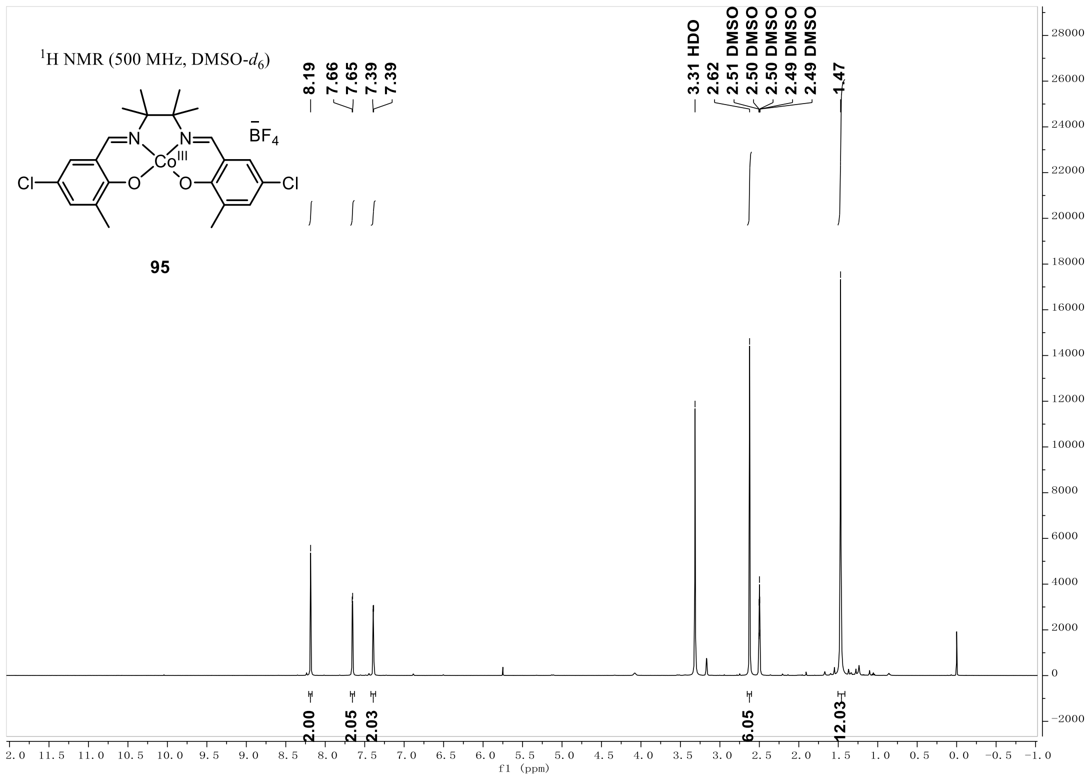

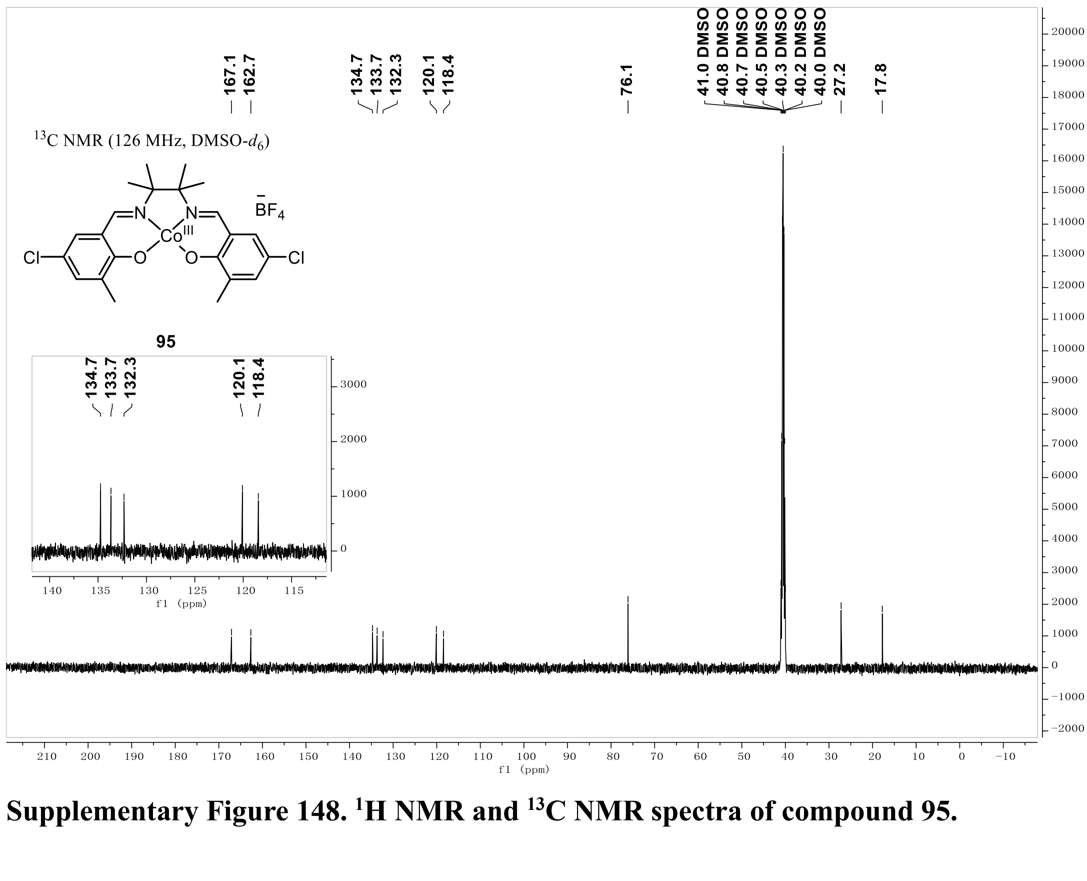

Supplementary Figure 148. ^{1}H NMR and ^{13}C NMR spectra of compound 95.

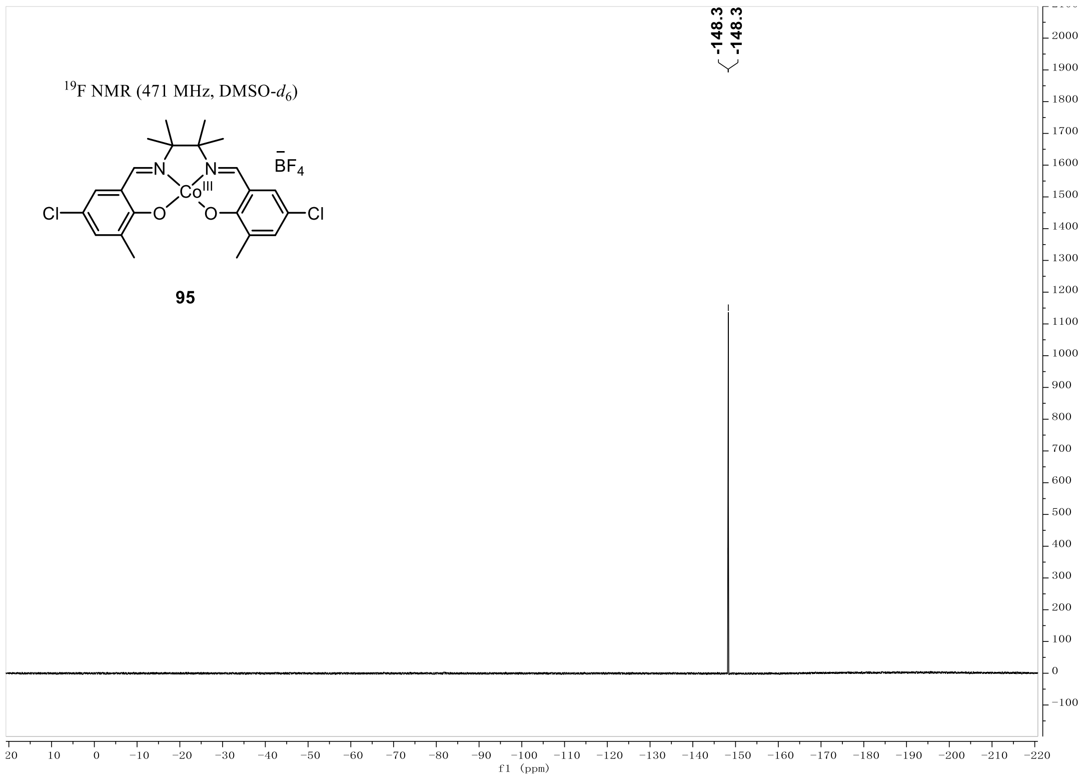

Supplementary Figure 149. ^{19}F NMR spectra of compound 95.

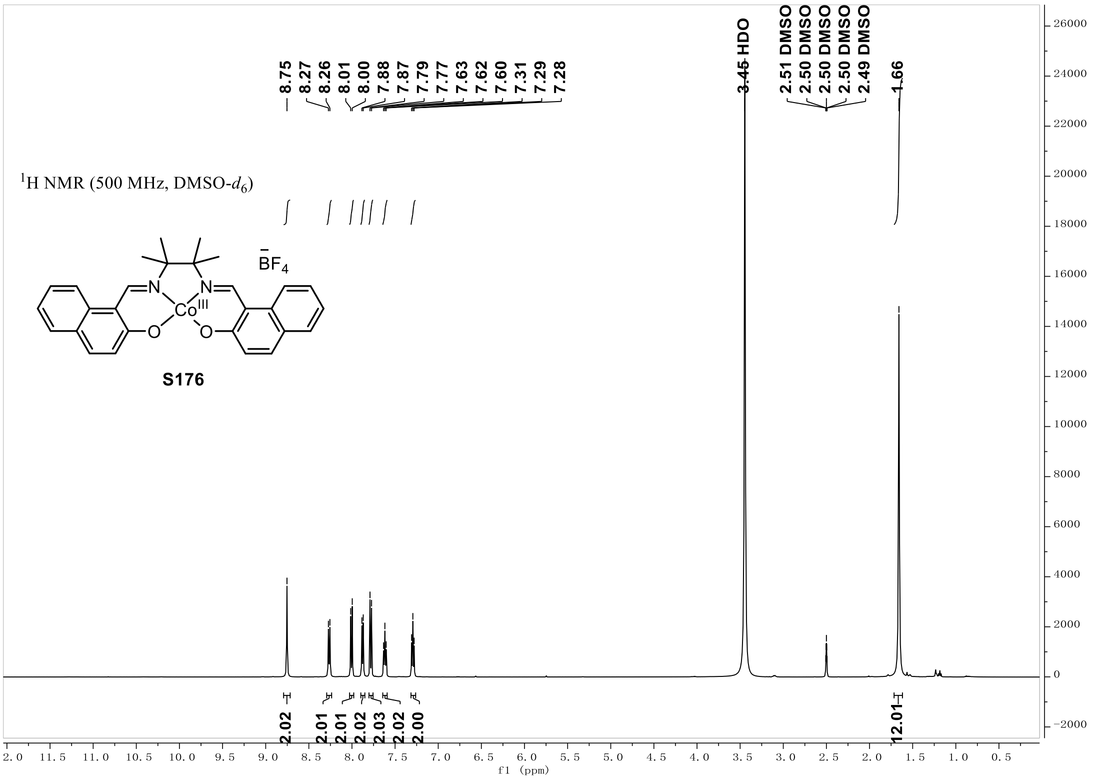

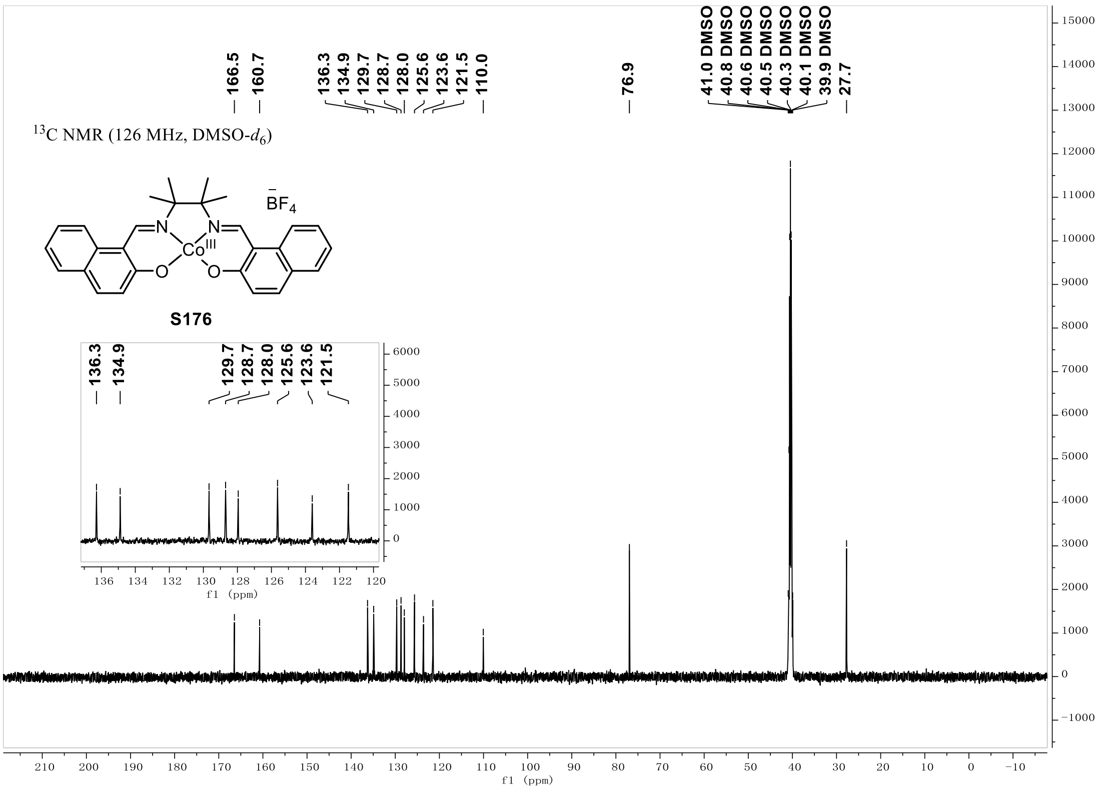

Supplementary Figure 150. ^1H NMR and ^{13}C NMR spectra of compound S176.

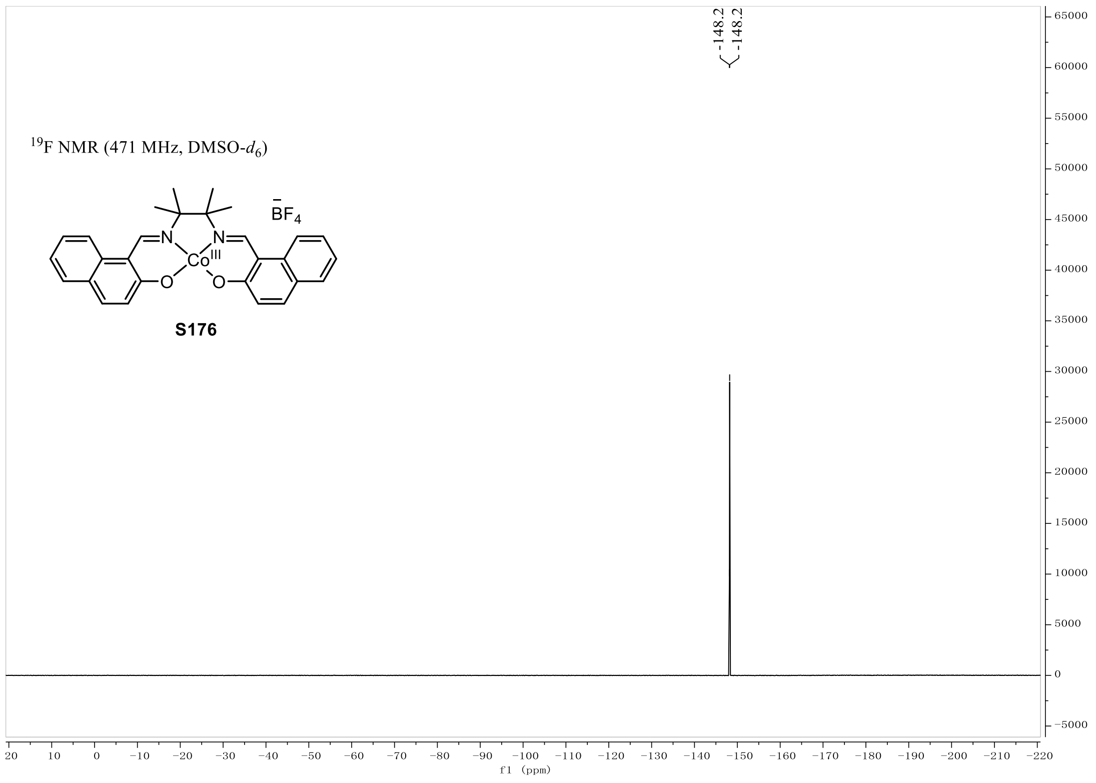

Supplementary Figure 151. ^{19}F NMR spectra of compound S176.

Supplementary References

1. Zhao, Y.; Truhlar, D. G. The M06 suite of density functionals for main group thermochemistry, thermochemical kinetics, noncovalent interactions, excited states, and transition elements: two new functionals and systematic testing of four M06-class functionals and 12 other functionals. *Theor Chem Acc* **120**, 215–241 (2008).
2. Hariharan, P. C.; Pople, J. A. The influence of polarization functions on molecular orbital hydrogenation energies. *Theor Chim Acta* **28**, 213–222 (1973).
3. Hehre, W. J.; Ditchfield, R.; Pople, J. A. Self–Consistent Molecular Orbital Methods. XII. Further Extensions of Gaussian–Type Basis Sets for Use in Molecular Orbital Studies of Organic Molecules. *J. Chem. Phys.* **56**, 2257–2261 (1972).
4. Weigend, F. Accurate Coulomb-fitting basis sets for H to Rn. *Phys. Chem. Chem. Phys.* **8**, 1057–1065 (2006).
5. Mardirossian, N.; Head-Gordon, M. How Accurate Are the Minnesota Density Functionals for Noncovalent Interactions, Isomerization Energies, Thermochemistry, and Barrier Heights Involving Molecules Composed of Main-Group Elements? *J. Chem. Theory Comput.* **12**, 4303–4325 (2016).
6. Mitra, H.; Roy, T. K. Comprehensive Benchmark Results for the Accuracy of Basis Sets for Anharmonic Molecular Vibrations. *J. Phys. Chem. A* **124**, 9203–9221 (2020).
7. Frisch, M. J.; Trucks, G. W.; Schlegel, H. B.; Scuseria, G. E.; Robb, M. A.; Cheeseman, J. R.; Scalmani, G.; Barone, V.; Petersson, G. A.; Nakatsuji, H.; Li, X.; Caricato, M.; Marenich, A. V.; Bloino, J.; Janesko, B. G.; Gomperts, R.; Mennucci, B.; Hratchian, H. P.; Ortiz, J. V.; Izmaylov, A. F.; Sonnenberg, J. L.; Williams; Ding, F.; Lipparini, F.; Egidi, F.; Goings, J.; Peng, B.; Petrone, A.; Henderson, T.; Ranasinghe, D.; Zakrzewski, V. G.; Gao, J.; Rega, N.; Zheng, G.; Liang, W.; Hada, M.; Ehara, M.; Toyota, K.; Fukuda, R.; Hasegawa, J.; Ishida, M.; Nakajima, T.; Honda, Y.; Kitao, O.; Nakai, H.; Vreven, T.; Throssell, K.; Montgomery Jr., J. A.; Peralta, J. E.; Ogliaro, F.; Bearpark, M. J.; Heyd, J. J.; Brothers, E. N.; Kudin, K. N.; Staroverov, V. N.; Keith, T. A.; Kobayashi, R.; Normand, J.; Raghavachari, K.; Rendell, A. P.; Burant, J. C.; Iyengar, S. S.; Tomasi, J.; Cossi, M.; Millam, J. M.; Klene, M.; Adamo, C.; Cammi, R.; Ochterski, J. W.; Martin, R. L.; Morokuma, K.; Farkas, O.; Foresman, J. B.; Fox, D. J. *Gaussian 16 Rev. C.01*, Wallingford, CT (2016).
8. Ye, K.-Y., McCallum, T. & Lin, S. Bimetallic Radical Redox-Relay Catalysis for the Isomerization of Epoxides to Allylic Alcohols. *J. Am. Chem. Soc.* **141**, 9548–9554 (2019).
9. Xiong, P., *et al.* Copper-Catalyzed Intramolecular Oxidative Amination of Unactivated Internal Alkenes. **22**, 4379–4383 (2016).
10. Xiong, P., Xu, H.-H. & Xu, H.-C. Metal- and Reagent-Free Intramolecular Oxidative Amination of Tri- and Tetrasubstituted Alkenes. *J. Am. Chem. Soc.* **139**, 2956–2959 (2017).
11. Kates, S.A., Dombroski, M.A. & Snider, B.B. Manganese(III)-based oxidative free-radical cyclization of unsaturated .beta.-keto esters, 1,3-diketones, and malonate diesters. *J. Org. Chem.* **55**, 2427–2436 (1990).

12. Zhu, L., *et al.* Electrocatalytic Generation of Amidyl Radicals for Olefin Hydroamidation: Use of Solvent Effects to Enable Anilide Oxidation. *Angew. Chem. Int. Ed.* **55**, 2226–2229 (2016).
13. Raghavan, S. & Samanta, P.K. Stereoselective Synthesis of the C13–C28 Subunit of (–)-Laulimalide Utilizing an α-Chlorosulfide Intermediate. *Synlett.* **24**, 1983–1987 (2013).
14. Li, J.-Q., *et al.* Asymmetric Hydrogenation of Allylic Alcohols Using Ir–N,P-Complexes. *ACS Catal.* **6**, 8342–8349 (2016).
15. Cirujano, F.G., *et al.* Boosting the Catalytic Performance of Metal–Organic Frameworks for Steroid Transformations by Confinement within a Mesoporous Scaffold. *Angew. Chem. Int. Ed.* **56**, 13302–13306 (2017).
16. Kita, Y., *et al.* Remarkable Effect of Aluminum Reagents on Rearrangements of Epoxy Acylates via Stable Cation Intermediates and Its Application to the Synthesis of (S)-(+)-Sporochnol A. *J. Org. Chem.* **66**, 8779–8786 (2001).
17. Zhu, C., Wang, D., Zhao, Y., Sun, W.-Y. & Shi, Z. Enantioselective Palladium-Catalyzed Intramolecular α-Arylative Desymmetrization of 1,3-Diketones. *J. Am. Chem. Soc.* **139**, 16486–16489 (2017).
18. Levine, S.R., Krout, M.R. & Stoltz, B.M. Catalytic Enantioselective Approach to the Eudesmane Sesquiterpenoids: Total Synthesis of (+)-Carissone. *Org. Lett.* **11**, 289–292 (2009).
19. Tharra, P. & Baire, B. Mild Approach to 2-Acylfurans via Intercepted Meyer–Schuster Rearrangement of 6-Hydroxyhex-2-en-4-ynals. *J. Org. Chem.* **80**, 8314–8328 (2015).
20. Patel, H.H., Prater, M.B., Squire, S.O. & Sigman, M.S. Formation of Chiral Allylic Ethers via an Enantioselective Palladium-Catalyzed Alkenylation of Acyclic Enol Ethers. *J. Am. Chem. Soc.* **140**, 5895–5898 (2018).
21. Hu, Q.-F., Gao, T.-T., Shi, Y.-J., Lei, Q. & Yu, L.-T. Palladium-catalyzed intramolecular C–H arylation of 2-halo-N-Boc-N-arylbenzamides for the synthesis of N–H phenanthridinones. *RSC Adv.* **8**, 13879–13890 (2018).
22. Lovinger, G.J., Aparece, M.D. & Morken, J.P. Pd-Catalyzed Conjunctive Cross-Coupling between Grignard-Derived Boron "Ate" Complexes and C(sp^2) Halides or Triflates: NaOTf as a Grignard Activator and Halide Scavenger. *J. Am. Chem. Soc.* **139**, 3153–3160 (2017).
23. Newhouse, T.R., Kaib, P.S.J., Gross, A.W. & Corey, E.J. Versatile Approaches for the Synthesis of Fused-Ring γ-Lactones Utilizing Cyclopropane Intermediates. *Org. Lett.* **15**, 1591–1593 (2013).
24. Boyer, F.-D. & Hanna, I. Synthesis of the Tricyclic Core of Colchicine via a Dienyne Tandem Ring-Closing Metathesis Reaction. *Org. Lett.* **9**, 2293–2295 (2007).
25. Arlt, A., Benson, S., Schulthoff, S., Gabor, B. & Fürstner, A. A Total Synthesis of Spirastrellolide A Methyl Ester. *Chem. Eur. J.* **19**, 3596–3608 (2013).
26. Hou, Z.-W., Yan, H., Song, J.-S. & Xu, H.-C. Electrochemical Synthesis of (Aza)indolines via Dehydrogenative [3+2] Annulation: Application to Total Synthesis of (±)-Hinckdentine A. *Chin. J. Chem.* **36**, 909–915 (2018).
27. Shevick, S.L., Obradors, C. & Shenvi, R.A. Mechanistic Interrogation of Co/Ni-Dual Catalyzed Hydroarylation. *J. Am. Chem. Soc.* **140**, 12056–12068 (2018).
